# High mRNA expression of LY6 gene family is associated with overall survival outcome in pancreatic ductal adenocarcinoma

**DOI:** 10.18632/oncotarget.27880

**Published:** 2021-02-02

**Authors:** Eric Russ, Krithika Bhuvaneshwar, Guisong Wang, Benjamin Jin, Michele M. Gage, Subha Madhavan, Yuriy Gusev, Geeta Upadhyay

**Affiliations:** ^1^Department of Pathology, Uniformed Services University, Bethesda, MD, USA; ^2^Innovation Center for Biomedical Informatics, Georgetown University Medical Center, Washington DC, USA; ^3^Murtha Cancer Center/Research Program, Department of Surgery, Uniformed Services University of the Health Sciences, Bethesda, MD, USA; ^4^The Henry M. Jackson Foundation for the Advancement of Military Medicine Inc, Bethesda, MD, USA; ^5^Walter Reed Navy Military Medical Center, Department of Surgery, Uniformed Services University, Bethesda, MD, USA

**Keywords:** LY6 genes, pancreatic cancer, immune cells, survival outcome

## Abstract

Pancreatic cancer ranks one of the worst in overall survival outcome with a 5 year survival rate being less than 10%. Pancreatic cancer faces unique challenges in its diagnosis and treatment, such as the lack of clinically validated biomarkers and the immensely immunosuppressive tumor microenvironment. Recently, the LY6 gene family has received increasing attention for its multi-faceted roles in cancer development, stem cell maintenance, immunomodulation, and association with more aggressive and hard-to-treat cancers. A detailed study of mRNA expression of LY6 gene family and its association with overall survival (OS) outcome in pancreatic cancers is lacking. We used publicly available clinical datasets to analyze the mRNA expression of a set of LY6 genes and its effect on OS outcome in the context of the tumor microenvironment and immunomodulation. We used web-based tools Kaplan-Meier Plotter, cBioPortal, Oncomine and R-programming to analyze copy number alterations, mRNA expression and its association with OS outcome in pancreatic cancer. These analyses demonstrated that high expression of LY6 genes is associated with OS and disease free survival (DFS) outcome. High expression of LY6 genes and their association with OS outcome is dependent on the composition of tumor microenvironment. Considering that LY6 proteins are anchored to the outer cell membrane or secreted, making them readily accessible, these findings highlight the potential of LY6 family members in the future of pancreatic cancer diagnosis and treatment.

## INTRODUCTION

Pancreatic cancer is a high-risk malignant neoplasm with a 5-year survival rate of less than 10%. The number of new cases and deaths of pancreatic cancer worldwide in 2018 was 458,918 and 432,242, respectively. In 2020, the projected number of incidences and deaths of pancreatic cancer patients in the United States is 57,600 and 47,050, respectively. Pancreatic ductal adenocarcinoma (PDAC) is the most common type of pancreatic cancer, accounting for more than 90% of all pancreatic cancer diagnoses [1, 2]. The poor prognosis of PDAC is due to several challenges, especially the lack of manifestation screening biomarkers, leading to most pancreatic cancer diagnoses being made in the later, more aggressive stages.

New biomarkers and therapeutic targets of PDAC are urgently needed. Currently, carbohydrate antigen (CA) 19-9 is the sole clinically approved serum biomarker for pancreatic cancer [3]. CA-19-9 is only used for disease monitoring due to a lack of specificity and sensitivity for the early diagnosis of asymptomatic cases. PDAC tends to have strong stem cell-like properties, causing pancreatic cancer to be relatively resistant to traditional chemotherapeutic strategies [4]. Mechanistically, mutations in the KRAS gene has been well described in PDAC, but this advanced knowledge has not resulted in therapeutic innovations due to multiple factors [1, 2]. KRAS protein is relatively small and lacks deep binding pockets, making it difficult to target with inhibitory drugs. KRAS is an intracellular protein and cannot be targeted with immunotherapy options such as antibodies or CAR-T cells. Because of these limitations, KRAS has been viewed as a difficult protein to develop targeted therapies [5, 6]. PDAC is presented with a highly immunosuppressive tumor microenvironment, leading to challenges in immunotherapy treatments such as the administration of checkpoint inhibitors [7–9]. Thus, there is a critical need for novel biomarkers relevant to therapeutic outcomes and novel therapeutic targets in PDAC.

Recently, the human lymphocyte antigen-6 (LY6) gene family has received increasing attention for its multi-faceted roles in cancer development, stem cell maintenance, immunomodulation, and association with more aggressive and hard-to-treat cancers [2, 10, 11]. The LY6 gene family is located on chromosomes 6, 8, 11, and 19. The LY6 family members in human chromosome 8 include PSCA, LY6K, SLURP1, LYPD2, SLURP2, LY6D, GML, LY6E, LY6L, LY6H, and GPIHBP1, mapped at 8q24.3 locus [10]. Somatic amplification of 8q has been suggested to be one of the most prevalent copy number gains in cancer [12–14]. LY6D, LY6E, LY6H, and LY6K have increased mRNA expression in tumor tissues of ovarian, colorectal, gastric, breast, lung, bladder, brain, cervical, esophageal, head and neck, and pancreatic cancer compared to adjacent normal tissues. The increased mRNA expression of LY6D, LY6E, LY6H and LY6K is associated with poor outcome in ovarian, colorectal, gastric, breast, lung, bladder or brain and CNS [2]. Increased levels of LY6A/E (Sca-1) has been reported to promote breast tumorigenesis via disruption of TGF-β signaling [15]. However, whether LY6 family members can be a biomarker for PDAC diagnosis, prognosis, and therapy remains elusive.

In this report, we analyzed the mRNA expression profile of 30 LY6 genes located on chromosomes 6, 8, 11 and 19 using the public The Cancer Genome Atlas (TCGA) dataset of 177 PDAC patients and their association with overall survival outcome. We used publicly available online tools Kaplan-Meier Plotter (KM Plotter), cBioPortal, Oncomine and survMisc R package [16–18]. Herein, we demonstrate differential associations of LY6 family members with patient survival outcome to discuss the potential influence of our identified proteins on the tumor microenvironment, immunomodulation, and stem cell maintenance. Although the function of many LY6 family members is yet to be established, several of our identified proteins have known roles in tumorigenesis and may be suitable candidate biomarkers for the diagnosis, prognosis, and treatment of PDAC.

## RESULTS

### High expression of LY6 mRNA was associated with overall survival outcome in pancreatic ductal carcinoma

We used Kaplan-Meier (KM) plotter web tool (https://kmplot.com) to see if LY6 gene expression was significantly associated with overall survival (OS) in pancreatic ductal adenocarcinoma (PDAC) [18]. We found 21 out of 30 queried genes were significantly associated with OS outcome in PDAC. We found that high mRNA expression of 17 LY6 genes - PSCA, SLURP1, LYPD2, LY6D, GML, Ly6E and LY6L on chromosome 8; LYPD4, PLAUR, LYPD5 on chromosome 19; PATE1, PATE2, PATE3 and CD59 on chromosome 11and LY6G6C, LY6G6D and LY6G6F on chromosome 6 to be significantly associated with poor OS outcome. The hazard ratios for these genes ranged from 1.68 to 2.99 indicating that patients with high mRNA expression of genes had approximately 1.6 to 3 times the risk of death compared to patients with low mRNA expression. We found high mRNA expression of 4 LY6 genes - LY6H on chromosome 8; PINLYP on chromosome 19; and LY6G5C and LY6G5B on chromosome 6 were significantly associated with good OS outcome (Supplementary Table 1 and Supplementary Figure 1).

**Figure 1:**
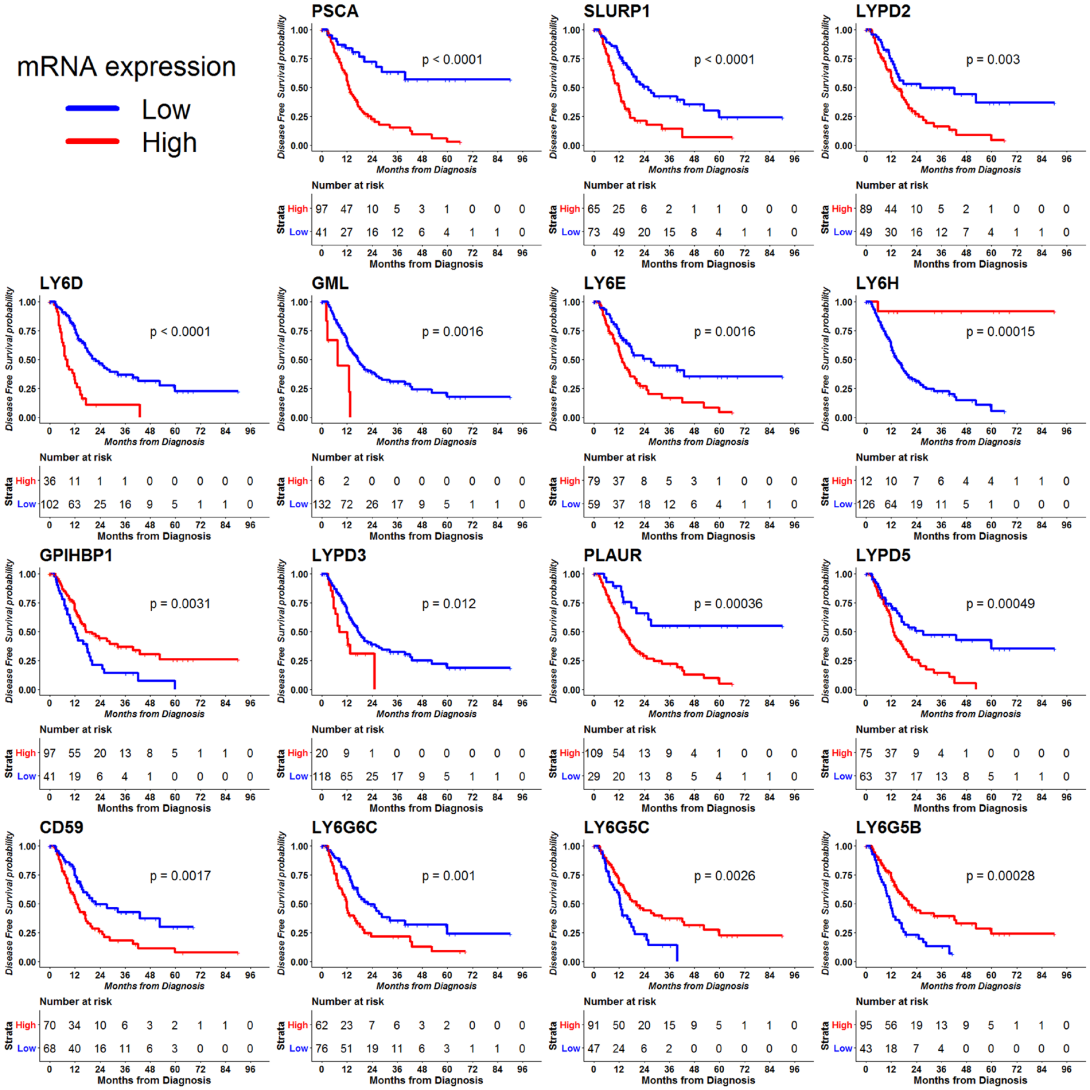
Disease free survival (DFS) outcome for 30 LY6 genes was analyzed using the RNA-seq data using the TCGA data. The clinical and expression data were accessed through the cgdsr package, R programming. The optimal cutoff for mRNA expression was determined using the method implemented in the survMisc R package. High mRNA expression of 15 genes associated with DFS outcome of PDAC. Note: KM plot for GML gene expression has only 6 patients in high expression, however this was included based on *p* value < 0.05.

We explored the association between RNA-seq data from Pancreatic Adenocarcinoma from TCGA with disease free survival (DFS) outcome using R-programming. We found that 16 out of the 30 LY6 genes were significantly associated with DFS outcome in PDAC. We found that high mRNA expression of 12 LY6 genes - PSCA, SLURP1, LYPD2, LY6D, GML and LY6E on chromosome 8; LYPD3, PLAUR, LYPD5 on chromosome 19; CD59 on chromosome 11 and LY6G6C on chromosome 6 to be significantly associated with poor DFS outcome. We found that high mRNA expression of 4 LY6 genes - LY6H and GPIHBP1 on chromosome 8; and LY6G5C and LY6G5B on chromosome 6 were significantly associated with good OS outcome (Figure 1).

We found that 13 LY6 genes were commonly associated with both OS and DFS outcome. We found high expression of 10 LY6 genes - PSCA, SLURP1, LYPD2, LY6D, GML, LY6E on chromosome 8; PLAUR and LYPD5 on chromosome 19; CD59 on chromosome 11 and LY6G6C on chromosome 6 were commonly associated with poor OS and DFS outcomes. We found high expression of 3 LY6 genes LY6H on chromosome 8; LY6G5C and LY6G5B on chromosome 6 were commonly associated with good OS and DFS outcomes (Supplementary Table 1 and Figure 1).

### High expression of LY6 mRNA expression and their association with overall survival outcome was dependent on cell type content of the tumors

Tumor microenvironment is composed of various cell types. The enrichment of specific cellular contents in a tumor microenvironment may play an important role as to how the tumor will progress or respond to therapeutic interventions. To estimate if inherent cellular content plays a role in the association of LY6 gene expression with the OS outcome, we used the restricted analysis feature of Kaplan-Meier Plotter tool. This feature allowed us to observe the OS outcome in patient samples with enriched or decreased cellular content of PDAC. Mesenchymal stem cells (MSCs) in tumor microenvironment have shown to be responsible for increased tumor metastasis and immune tolerance of tumors [19–21]. We studied how the LY6 genes affected OS outcome in PDAC patients based on the MSCs status in the tumor samples. We found high mRNA expression of LY6D, SLURP1, CD59, PSCA, PATE2, LY6G6F, LYPD5, LY6E, PATE1, LYPD2, LY6G6D, PATE3, LYPD4, and GML was significantly associated with poor OS outcome only in the MSC enriched patient population. High expression of LY6G6C and GML was associated with poor OS outcome in the MSC enriched population. High expression of LY6G6C and GML was associated with good OS outcome in the MSC decreased population. LY6G5C was associated with good OS outcome only in the MSC enriched population. Ly6G5B was associated with good OS outcome independent of MSC status. Ly6H, PINLYP was associated with good OS outcome only in MSC enriched population. TEX101 was associated with good OS outcome only in MSC decreased population (Table 1 and Supplementary Figure 2).

**Table 1:** High expression of LY6 genes and its association with overall survival outcome (OS) in TCGA dataset of PDAC patient population based on mesenchymal stem cells (MSC) status, as observed by using KM plotter tool

Gene	MSC enriched population			MSC decreased population		
	OS	*p* value	FDR	OS	*p* value	FDR
LY6D	Poor	0.00002	1%	NS	0.11	100%
SLURP1	Poor	0.00002	1%	NS	0.18	100%
PSCA	Poor	0.00005	1%	NS	0.33	100%
LY6G5C	Good	0.00005	2%	NS	0.16	100%
CD59	Poor	0.00007	2%	NS	0.22	100%
LY6G6F	Poor	0.00037	10%	NS	0.39	100%
PATE1	Poor	0.00061	20%	NA	NA	NA
PATE2	Poor	0.00130	50%	NS	0.21	100%
LY6G6D	Poor	0.00160	10%	NS	0.17	100%
LY6E	Poor	0.00290	50%	NS	0.26	100%
LY6G5B	Good	0.00300	50%	Good	0.041	50%
LY6G6C	Poor	0.00410	20%	Good	0.046	50%
LYPD5	Poor	0.00480	50%	NS	0.13	100%
LYPD2	Poor	0.00520	50%	NS	0.14	100%
PATE3	Poor	0.00640	50%	NS	0.59	100%
LY6H	Good	0.00670	50%	NS	0.091	100%
PINLYP	Good	0.00680	50%	NS	0.077	100%
LYPD4	Poor	0.02100	50%	NS	0.15	100%
LY6L	Poor	0.02200	50%	Good	0.036	50%
GML	Poor	0.03700	50%	NS	0.33	100%
TEX101	NS	0.12000	100%	Good	0.033	50%

LY6 gene expression with NS association in both categories are not displayed. The table is arranged in increasing *p* value for MSC enriched population. For the original KM plots, sample sizes in MSCs enriched and decreased population and other details, please refer to Supplementary Figure 2. NS: Not Significant, *p* < 0.05; OS: Overall Survival, FDR: False Discovery Rate, HR: Hazard Ratio.

Regulatory T cells (Treg cells) play important roles in suppressing immune responses in the tumor microenvironment. They can inhibit cytotoxic T-cell lymphocytes (CTL) from attacking the cancer cells or infiltrating the tumor microenvironment. We observed that high expression of LY6D, SLURP1, PSCA, LY6G6C, LY6E, LYPD5, LY6G6D, PATE2, and PATE1 were significantly associated with poor OS outcome independent of the Treg status. High expression of LY6G5C and LY6G5B were associated good OS outcome independent of Treg status. CD59, LY6G6F, and PATE3 were associated with poor OS outcome only in Treg decreased population. LYPD2 and PLAUR were only associated with poor OS outcome only in Treg enriched population. (Table 2 and Supplementary Figure 3).

**Table 2:** High expression of LY6 genes and its association with overall survival outcome (OS) in TCGA dataset of PDAC patient population based on T-regulatory cells (Treg) status, as observed by using KM plotter tool

Gene	Treg enriched population			Treg decreased population		
	OS	*p* value	FDR	OS	*p* value	FDR
LY6D	Poor	0.00002	1%	Poor	0.00220	20%
SLURP1	Poor	0.00007	1%	Poor	0.00360	>50%
PSCA	Poor	0.00030	2%	Poor	0.01760	>50%
LY6G6C	Poor	0.00210	20%	Poor	0.00860	>50%
LYPD2	Poor	0.00220	20%	NS	0.09210	100%
LY6G5C	Good	0.00230	50%	Good	0.00030	5%
LY6G5B	Good	0.00290	10%	Good	0.00790	>50%
PLAUR	Poor	0.00390	50%	NS	0.20220	100%
LY6E	Poor	0.00810	50%	Poor	0.02210	>50%
LYPD5	Poor	0.01340	>50%	Poor	0.00770	50%
LY6G6D	Poor	0.02650	100%	Poor	0.04400	>50%
PATE2	Poor	0.02800	>50%	Poor	0.00200	50%
PATE1	Poor	0.03690	>50%	Poor	0.01290	>50%
LY6G6F	NS	0.05030	100%	Poor	0.00540	>50%
CD59	NS	0.07340	100%	Poor	0.00004	1%
PATE3	NS	0.16470	100%	Poor	0.02020	>50%

LY6 gene expression with NS association in both categories are not displayed. The table is arranged in increasing *p* value for Treg enriched population. For the original KM plots, sample sizes in T-reg enriched and decreased population and other details, please refer to Supplementary Figure 3. NS: Not Significant, *p* < 0.05; OS: Overall Survival, FDR: False Discovery Rate, HR: Hazard Ratio.

CD8 positive T-cells are major defense against cancer. Tumors with infiltrated CD8 positive T-cells are termed as inflamed or hot tumors [22]. High expression of PSCA, LY6D, SLURP1, PATE2, LYPD5, PATE1, and LY6G6F was associated with poor OS outcome independent of CD8 positive T-cells status. High expression of LY6G6C and LY6G5B was associated with good OS outcome independent of CD8 positive T-cells status. High expression of PLAUR, LY6E, PATE3, LYPD2, and CD177 was associated with poor OS outcome only in CD8 enriched population. High expression of LY6H was associated with good OS outcome only in CD8 enriched population. High expression of LY6G6C, LY6G6D, CD59, and LYPD4 was associated with poor OS outcome only in CD8 decreased population. High expression of LYPD3 was associated with poor OS outcome in CD8 enriched and with good OS outcome in CD8 decreased population (Table 3 and Supplementary Figure 4).

**Table 3:** High expression of LY6 genes and its association with overall survival outcome (OS) in TCGA dataset of PDAC patient population based on CD8 positive (CD8+) status, as observed by using KM plotter tool

Gene	CD8+ enriched population			CD8+ decreased population		
	OS	*p* value	FDR	OS	*p* value	FDR
LY6D	Poor	0.000074	1%	Poor	0.0016	20%
SLURP1	Poor	0.0012	20%	Poor	0.0017	50%
PATE2	Poor	0.0013	20%	Poor	0.011	50%
LYPD5	Poor	0.0016	10%	Poor	0.035	50%
LY6G5C	Good	0.0022	10%	Good	0.0026	20%
PSCA	Poor	0.005	50%	Poor	0.0034	50%
LYPD3	Poor	0.0092	50%	Good	0.009	50%
PATE1	Poor	0.0095	50%	Poor	0.022	50%
PLAUR	Poor	0.01	50%	NS	0.21	100%
LY6G5B	Good	0.011	50%	Good	0.0049	50%
LY6E	Poor	0.015	50%	NS	0.082	100%
LY6H	Good	0.019	50%	NS	0.078	100%
LYPD2	Poor	0.02	50%	NS	0.097	100%
CD177	Poor	0.023	50%	NS	0.27	100%
LY6G6F	Poor	0.038	50%	Poor	0.0064	50%
LY6L	NS	0.049	50%	NS	0.13	100%
LY6G6C	NS	0.057	100%	Poor	0.015	50%
LY6G6D	NS	0.07	100%	Poor	0.014	50%
CD59	NS	0.1	100%	Poor	0.0014	20%
LYPD4	NS	0.21	100%	Poor	0.044	50%

LY6 gene expression with NS association in both categories are not displayed. The table is arranged in increasing *p* value for CD8+ enriched population. For the original KM plots, sample sizes in in CD8+ cells enriched and decreased population and other details, please refer to Supplementary Figure 4. NS: Not Significant, *p* < 0.05; OS: Overall Survival, FDR: False Discovery Rate, HR: Hazard Ratio.

Macrophages play an important role in pancreatic beta cell function, pancreatic tissue homeostasis and pancreatic cancer [23, 24]. High expression of LY6D, CD59, and SLURP1 was associated with poor OS outcome and LY6G5B and LY6G5C was associated with good OS outcome independent of macrophage status. High expression of PATE2 was associated with poor OS outcome in macrophage enriched population. SPACA4, LYPD4, LYPD3, and LY6L was associated with good OS outcome in macrophages enriched population and with poor OS outcome in macrophages decreased population. High expression of LY6H and LY6K was associated with good OS outcome only in macrophages decreased population. High expression of PSCA, TEX101, CD177, LY6G6D, PATE1, LY6E, LY6G6F, LYPD2, PATE3, LYPD5, GML and LY6G6C was associated with poor OS outcome in macrophages decreased population (Table 4 and Supplementary Figure 5).

**Table 4:** High expression of LY6 genes and its association with overall survival outcome (OS) in TCGA dataset of PDAC patient population based on macrophages status, as observed by using KM plotter tool

Gene	Macrophages enriched population			Macrophages decreased population		
	OS	*p* value	FDR	OS	*p* value	FDR
LY6D	Poor	0.00017	2%	Poor	0.00001	1%
LY6G5B	Good	0.00090	10%%	Good	0.04400	50%
CD59	Poor	0.00890	50%	Poor	0.00030	5%
PATE2	Poor	0.00970	50%	NS	0.10000	100%
SLURP1	Poor	0.01700	50%	Poor	0.00000	1%
SPACA4	Good	0.01900	50%	Poor	0.02900	50%
LYPD4	Good	0.02100	50%	Poor	0.00350	50%
LY6G5C	Good	0.02700	50%	Good	0.00002	1%
LYPD3	Good	0.02900	50%	Poor	0.03500	50%
LY6L	Good	0.04100	50%	Poor	0.00330	20%
PLAUR	NS	0.06900	100%	Poor	0.00092	10%
LY6H	NS	0.07800	100%	Good	0.00070	10%
PSCA	NS	0.09500	100%	Poor	0.00000	1%
TEX101	NS	0.09600	100%	Poor	0.00990	50%
CD177	NS	0.12000	100%	Poor	0.00790	50%
LY6G6D	NS	0.12000	100%	Poor	0.01400	50%
PATE1	NS	0.13000	100%	Poor	0.01200	50%
LY6K	NS	0.14000	100%	Good	0.00470	50%
LY6E	NS	0.14000	100%	Poor	0.00014	1%
LY6G6F	NS	0.18000	100%	Poor	0.00140	20%
LYPD2	NS	0.27000	100%	Poor	0.00006	1%
PATE3	NS	0.29000	100%	Poor	0.01200	50%
LYPD5	NS	0.35000	100%	Poor	0.00001	1%
GML	NS	0.45000	100%	Poor	0.02800	50%
LY6G6C	NS	0.48000	100%	Poor	0.00018	2%

LY6 gene expression with NS association in both categories are not displayed. The table is arranged in increasing *p* value for macrophages enriched population. For the original KM plots, sample sizes in macrophages enriched and decreased population and other details, please refer to Supplementary Figure 5. NS: Not Significant, *p* < 0.05; OS: Overall Survival, FDR: False Discovery Rate, HR: Hazard Ratio.

Natural killer T-cell (NKT cells) have the characteristics of natural killer (NK) cells and T-cells [25, 26]. High expression of LY6D, SLURP1, LYPD5, PSCA, CD59, PATE2, LY6G6F and LY6E was significantly associated with poor OS outcome independent of NKT status. High expression of LY6G5C, LY6H, and LY6G5B was significantly associated with good OS outcome independent of NKT population. High expression of LYPD2, PLAUR, LY6G6C, and CD177 was significantly associated with poor OS outcome only in the NKT enriched population. High expression of PATE4 was associated with good OS outcome in NKT enriched and poor OS outcome in NKT decreased population. High expression of LYPD4, PATE3, PATE1, and LY6G6D was associated with poor OS outcome only in NKT decreased patient population (Table 5 and Supplementary Figure 6).

**Table 5:** High expression of LY6 genes and its association with overall survival outcome (OS) in TCGA dataset of PDAC patient population based on NKT cell status, as observed by using KM plotter tool

Gene	NKT enriched population			NKT decreased population		
	OS	*p* value	FDR	OS	*p* value	FDR
LY6D	Poor	0.00009	2%	Poor	0.00100	20%
SLURP1	Poor	0.00010	2%	Poor	0.00330	50%
LY6G5C	Good	0.00030	2%	Good	0.00490	50%
LYPD2	Poor	0.00035	5%	NS	0.26000	100%
LYPD5	Poor	0.00170	20%	Poor	0.04700	50%
PSCA	Poor	0.00410	20%	Poor	0.00260	50%
PATE4	Good	0.00700	50%	Poor	0.01100	50%
CD59	Poor	0.00750	50%	Poor	0.00310	20%
LY6H	Good	0.00850	50%	Good	0.00900	50%
PLAUR	Poor	0.00990	50%	NS	0.42000	100%
LY6K	Good	0.01100	50%	NS	0.40000	100%
LY6G5B	Good	0.01400	50%	Good	0.00340	50%
LY6G6C	Poor	0.01500	50%	NS	0.17000	100%
PATE2	Poor	0.01600	50%	Poor	0.01100	50%
CD177	Poor	0.01700	50%	NS	0.33000	100%
LY6G6F	Poor	0.01700	50%	Poor	0.00660	50%
LY6E	Poor	0.03400	50%	Poor	0.00700	50%
LYPD4	NS	0.06300	100%	Poor	0.01900	50%
PATE3	Good	0.06900	100%	Poor	0.03400	50%
PATE1	NS	0.08700	100%	Poor	0.00650	50%
LY6G6D	NS	0.29000	100%	Poor	0.00160	20%

LY6 gene expression with NS association in both categories are not displayed. The table is arranged in increasing *p* value for NKT enriched population. For the original KM plots, sample sizes in NKT cells enriched and decreased population and other details, please refer to Supplementary Figure 6. NS: Not Significant, *p* < 0.05; OS: Overall Survival, FDR: False Discovery Rate, HR: Hazard Ratio.

CD4 positive T-cells are helper memory T-cells which can activate cytotoxic T-cells, natural killer T-cells, B-cells and macrophages to activate immune responses [27, 28]. High expression of LY6D, LY6E, LY6L, LYPD4, LYPD5, and SLURP1 was significantly associated with poor OS outcome independent of CD4+ memory T-cell status. High expression of LY6G5B was strongly associated with good OS outcome independent of CD4+ memory T-cell status. High expression of PATE1 was associated with good OS outcome in CD4+ enriched and poor OS outcome in CD4+ decreased population. High expression of PATE3, LYPD2, GML, PSCA, PATE2, LY6G6D, CD59, and LY6G6C was associated with poor OS outcome only in the CD4+decraesed population. High expression of LY6H, and LY6G5C was associated with good OS outcome in CD4+ decreased population (Table 6 and Supplementary Figure 7).

**Table 6:** High expression of LY6 genes and its association with overall survival outcome (OS) in TCGA dataset of PDAC patient population based on CD4+ memory T-cell status, as observed by using KM plotter tool

Gene	CD4+ enriched population			CD4+ decreased population		
	OS	*p* value	FDR	OS	*p* value	FDR
LY6D	Poor	0.000048	1%	Poor	0.0001	5%
LY6E	Poor	0.0012	10%	Poor	0.0248	>50%
LY6L	Poor	0.0037	20%	Poor	0.0056	>50%
LYPD4	Poor	0.0038	20%	Poor	0.003	>50%
LY6G5B	Good	0.0062	>50%	Good	0.009	50%
LYPD5	Poor	0.0142	>50%	Poor	0.0044	50%
SLURP1	Poor	0.0328	>50%	Poor	0.000013	1%
PATE1	Good	0.0465	>50%	Poor	0.0007	10%
PATE3	NS	0.061	100%	Poor	0.0052	>50%
LYPD2	NS	0.0854	100%	Poor	0.0041	>50%
GML	NS	0.1008	100%	Poor	0.0112	>50%
PSCA	NS	0.1014	100%	Poor	0.000084	2%
PATE2	NS	0.1275	100%	Poor	0.0004	20%
LY6G6D	NS	0.1351	100%	Poor	0.0016	10%
CD59	NS	0.1934	100%	Poor	0.0006	10%
LY6H	NS	0.214	100%	Good	0.006	50%
LY6G5C	NS	0.277	100%	Good	0.00006	2%
LY6G6C	NS	0.4561	100%	Poor	0.0037	50%

LY6 gene expression with NS association in both categories are not displayed. The table is arranged in increasing *p* value for CD4+ memory T-cells enriched population. For the original KM plots, sample sizes in CD4+ memory T-cell enriched and decreased population and other details, please refer to Supplementary Figure 7. NS: Not Significant, *p* < 0.05; OS: Overall Survival, FDR: False Discovery Rate, HR: Hazard Ratio.

Presence of B-cells have been demonstrated to correlate with good prognosis in many different cancers [29–31]. Interestingly, LY6D has been shown to be involved in B-cell differentiation [32, 33]. High expression of LY6D, LY6E, PSCA, SLURP1, LYPD5, and PATE2 was strongly associated with poor outcome independent of B-cell status. High expression of LY6G5B and LY6G5C was associated with good OS outcome independent of B-cell status. High expression of PLAUR, LYPD4, LY6G6C, LYPD2, CD59, LY6G6D, LY6L, GML, PATE3, and PATE1 was associated with poor OS outcome only in B-cell decreased population. High expression of LY6H was associated with good OS outcome only in B-cell decreased population. (Table 7 and Supplementary Figure 8).

**Table 7:** High expression of LY6 genes and its association with overall survival outcome (OS) in TCGA dataset of PDAC patient population based on B-cell status, as observed by using KM plotter tool

Gene	B cell enriched population			B cell decreased population		
	OS	*p* value	FDR	OS	*p* value	FDR
LY6D	Poor	0.00003	1%	Poor	0.0009	10%
LY6E	Poor	0.006	10%	Poor	0.0078	>50%
PSCA	Poor	0.0152	100%	Poor	0.0005	5%
LY6G5B	Good	0.0213	>50%	Good	0.0017	10%
SLURP1	Poor	0.0242	>50%	Poor	0.000009	1%
LY6G5C	Good	0.0283	100%	Good	0.0001	5%
LYPD5	Poor	0.0355	>50%	Poor	0.003	50%
PLAUR	NS	0.0433	100%	Poor	0.0644	>50%
PATE2	Poor	0.0488	100%	Poor	0.0014	50%
LYPD4	NS	0.0525	20%	Poor	0.009	>50%
LY6G6C	NS	0.055	100%	Poor	0.0078	>50%
LYPD2	NS	0.0967	100%	Poor	0.0021	50%
CD59	NS	0.1004	100%	Poor	0.0000068	1%
LY6G6D	NS	0.1892	100%	Poor	0.0038	>50%
LY6L	NS	0.2433	20%	Poor	0.043	>50%
LY6H	NS	0.3599	100%	Good	0.0007	20%
GML	NS	0.3734	100%	Poor	0.0385	>50%
PATE3	NS	0.4032	100%	Poor	0.0261	>50%
PATE1	NS	0.6189	>50%	Poor	0.0039	50%

LY6 gene expression with NS association in both categories are not displayed. The table is arranged in increasing *p* value for B-cells enriched population. For the original KM plots, sample sizes in B-cells enriched and decreased population and other details, please refer to Supplementary Figure 8. NS: Not Significant, *p* < 0.05; OS: Overall Survival, FDR: False Discovery Rate, HR: Hazard Ratio.

### LY6 DNA were amplified in pancreatic ductal carcinoma

**Figure 2:**
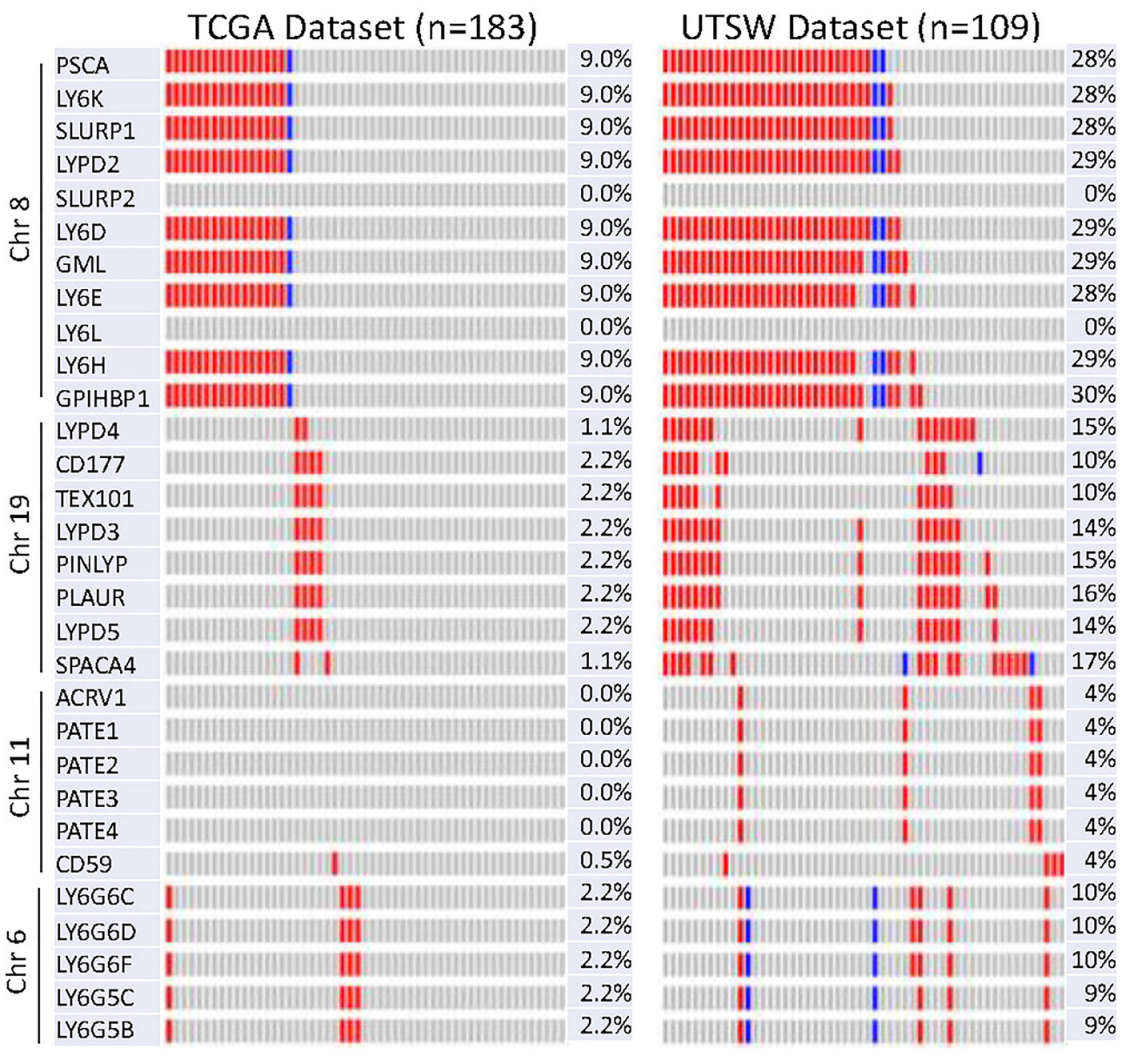
Oncoprint of copy number variation of 30 LY6 genes in TCGA and UTSW dataset using cBioPortal. Each column represents a patient/sample. DNA amplification in red and DNA deep deletion in blue are depicted in the indicated genes.

LY6 genes have been reported to be upregulated in multiple cancers [11, 34]. To test whether LY6 gene family members are amplified in PDAC, we assessed copy number variation data including DNA amplification and deep deletions from TCGA dataset (*n* = 177) and Pancreatic Cancer UTSW dataset (*n* = 109) hosted on cBioPortal tool [35]. We observed that LY6 genes located on the chromosome 8q24.3, PSCA, LY6K, SLURP1, LYPD2, LY6D, GML, LY6E, LY6H, and GPIHBP1 were co-amplified in most 9 to 28% of PDAC cases in TCGA and UTSW datasets. Interestingly, the cluster of LY6 genes which expressed on same genetic location were co-amplified in the same patients. The deep deletions in LY6 genes were only observed in few cases (Figure 2).

### LY6 mRNA expression is upregulated in cancer compared to normal tissues

**Figure 3:**
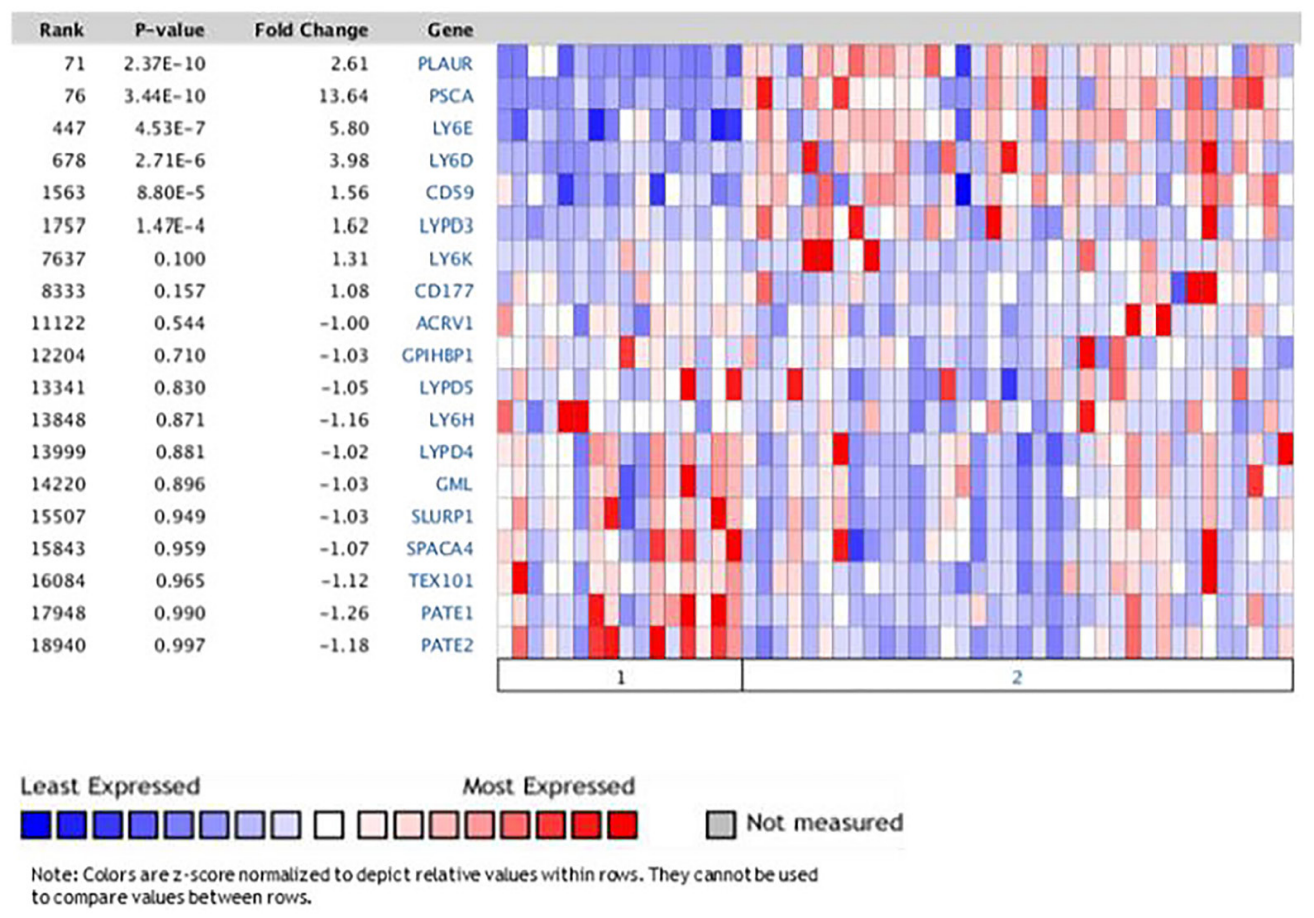
Oncomine tool showed significant increase expression of LY6 genes in pancreatic tumor vs normal tissues in Pei dataset comparing normal vs tumor PDAC tissue. (1) Normal Pancreas (16 cases); (2) Pancreatic Carcinoma (36 cases).

The differential expression of LY6 genes between the tumor tissue and the normal tissue will promote the understanding of their potentials as prognosis and therapeutic biomarkers. We evaluated 30 LY6 genes in the Pei dataset of pancreatic tumors versus normal tissues hosted at Oncomine. LY6D, LY6E, PLAUR, PSCA, CD59 and LYPD3 mRNA expression was significantly increased in the PDAC tumor tissues than normal adjacent tissues (*p* < 0.01) (Figure 3).

## DISCUSSION

The first step towards research and development of pancreatic cancer treatment will be to identify and define the novel tumor-specific biomarkers. Mutations in the oncogenic KRAS gene occur in over 90% of patients and are viewed as driving force of pancreatic cancer [5]. However, a history of detailed knowledge in the KRAS mechanistic pathway has not yet led to a clinical breakthrough in the treatment of PDAC [5, 6]. With a five-year survival rate of less than 10%, there is an urgent need for innovative treatment strategies. Compared to other solid malignancies, challenges in pancreatic cancer include the immensely immunosuppressive tumor microenvironment, in addition to the presence of a dense desmoplastic barrier, which limits the diffusion of therapeutic drugs and the infiltration of immunotherapy-based anti-tumor immune cells [5–8]. An increased understanding of the key molecular pathways unique to pancreatic cancer which contribute to its immunosuppressive and stem cell-like properties is required to develop novel and successful therapeutic strategies against pancreatic cancer. Herein, we analyze the expression of LY6 gene family and its association with OS outcome in clinical samples of PDAC.

### LY6 gene expression in the context of tumor microenvironment

#### Mesenchymal stems cell (MSC) enriched pancreatic tumors

MSC enriched tumors showed the strongest association between high expression of LY6D/SLURP1/PSCA/CD59 and low overall survival outcome in PDAC. PDAC tumors with low MSC did not show significant association with high LY6 gene expression to OS outcome. This observation suggests that LY6 gene expression and its association with OS outcome is specifically relevant in the presence of MSCs in the pancreatic tumor microenvironment. MSCs are instrumental in providing the immunosuppressive tumor microenvironment. They can suppress CD4 and CD8 positive T-cells. MSCs can secrete various growth factors which can regulate gene expression directly on cancer cells [36]. It remains to be seen if the LY6 gene expression is associated with a direct immunosuppressive environment due to presence of MSCs.

#### Immune cell enriched pancreatic tumors

We observed that high expression of many LY6 genes were associated significantly with lower OS in PDAC population enriched for Treg, CD8, macrophages, NKT, B-cells and CD4+ immune cells. However, a single pattern of association did not emerge for each of the LY6 genes, suggesting the LY6 genes may be differentially regulated. In contrast to other LY6 genes, high expression for CD59 was strongly associated with lower OS in PDAC population decreased for CD8, Treg, macrophages, NKT, B-cells and CD4+ immune cells. This observation suggests that CD59 is associated with tumor environments which present with lower immune cell infiltrates [37]. The overexpression of CD59 in pancreatic cancer has major consequences on the tumor microenvironment and was previously shown to be required for stem cell evasion of complement surveillance, a biological mechanism for eliminating cancer stem cells in epithelial cancer [37].

We observed that increased mRNA of SLURP1 was associated with lower OS outcome in pancreatic cancer. This observation was in agreement with public data from The Human protein Atlas data which showed that high expression of mRNA for SLURP1 is associated with lower OS. This observation, however was in conflict with a previously reported finding that high expression of SLURP1 protein is associated with higher OS outcome in pancreatic cancer [38]. Further studies are required to understand the role of SLURP1 mRNA and protein in pancreatic cancer and its association with OS outcome in PDAC. *In vivo* evidence indicates that SLURP1 is a major component of maintaining immune privilege through inhibiting leukocytic binding and infiltration in a corneal model, suggesting that SLURP1 can also serve as a potent inhibitor of immune activity [39, 40]. However this has not been tested in tumor models, it is plausible that SLURP1 can contribute to pancreatic cancer’s immunosuppressive tumor microenvironment and suppress anti-tumor immune responses.

### LY6 proteins and maintenance of proliferation and stem cell-like properties

LY6D, LY6E, PSCA, and PLAUR, known markers tumorigenesis and cancer cell maintenance, were significantly associated with lower overall survival outcome in our pancreatic cancer analysis [10, 11]. LY6D is a GPI-anchored member of the LY6 family with a recently established association with aggressive cancers and poor patient outcome [10, 11]. Under normal physiological conditions, LY6D is most commonly used as a marker of early B cell lineage; however, in response to genotoxic stressors such as radiation and chemotherapy, LY6D expression is upregulated in numerous cancer types and is suggested to contribute to distant metastasis in breast cancer [41–43]. LY6D serves as a marker of luminal progenitors with bi-lineage capacity and intrinsic castration-resistant properties in prostate cancer [44]. Together, our analysis and previous research suggests that LY6D expression may indicate a more aggressive pancreatic cancer phenotype and it would therefore be beneficial to explore the precise mechanism of action for LY6D to determine its therapeutic potential.

LY6E, a GPI-anchored member of the LY6 family, has recently been implicated as a driver of tumorigenesis and stem cell maintenance through inhibiting expression of the tumor suppressor PTEN and inducing the upregulation of the HIF-1 pathway [45, 46]. Notably, inhibition of LY6E with siRNA was shown to restore PTEN expression, induce G1-S phase cell cycle arrest, and increase apoptosis in gastric cancer, suggesting that LY6E’s inhibition may be enough to cause anti-tumor effects in some cancers [46]. On a clinical level, high LY6E expression correlates with poor overall patient survival in various malignant tumors such as those of gastric, breast, head and neck, lung, bladder, brain, and skin origin [11, 45]. Importantly, in pancreatic cancer specifically, LY6E was suggested to be a marker for cancer cells with stem cell properties and was used in addition to the stem cell markers TACSTD1 and CD44 to establish a sorting technique to obtain clonal colony-forming pancreatic cancer stem cells [47].

GPI-anchored LY6 family member PSCA is most commonly attributed to prostate stem cells and prostate cancer; however, its overexpression in pancreatic cancer and limited expression in normal pancreatic cells is also well recognized [48, 49]. PSCA can be a target of immunotherapy strategies such as anti-PSCA antibody and anti-PSCA CAR-T cell therapy, with a clinical trial currently underway for the latter [6]. However, PSCA’s function in pancreatic cancer is still unknown and requires further research.

### Gene copy number amplifications of LY6 proteins on chromosome 8

LY6D, LY6E, LY6H, LY6K, PSCA, SLURP1, LYPD2, and GPIHP1 had co-amplifications of their gene copy numbers as seen on cBioPortal in TCGA dataset While increased DNA copy number does not necessarily indicate increased levels of protein expression, the mRNA expression data for these genes was found to be relatively high in the patients with gene copy number amplifications, suggesting that an amplified copy number for LY6 genes on chromosome 8 increases LY6 gene expression. Understanding what causes both an amplification of LY6 gene copy number and an overexpression of LY6 genes on chromosome 8 may reveal important insight into the molecular phenotype of this subset of pancreatic cancer patients.

### The “good” LY6 genes

High expression of LY6G5B, LY6G5C, and LY6G6C was associated with high OS outcome. These LY6 genes are located among the MHC class III proteins and are known as MHC-linked LY6 genes. Although the precise functions of the LY6 family members LY6G5B, LY6G5C, LY6G6C are unknown, they are suggested to be located on filopodia and secreted proteins with binding potential to the cell surface and may be involved in cell signaling. Interestingly, potential ligands for LY6G5C, LY6G6C were found to be present on K562 cells, an undifferentiated megakaryocyte cell line, among a panel of cell lines, indicating a potential role in hematopoietic cell differentiation [50, 51]. It is unclear how these genes are associated with higher OS outcome. It is likely that cell-cell interaction may play important role in LY6 signaling.

### Concluding remarks

LY6 family proteins are either attached to the outer cell surface through a GPI-anchor or are secreted into the extracellular matrix, making them relatively accessible for drug inhibition or immunotherapy targeting. In this report, we sought to analyze the mRNA expression of LY6 gene family and its association with overall survival (OS) outcome in pancreatic cancer patients. We focused our exploration on thirty LY6 family genes scattered on various chromosomes [2, 10, 11]. We found that high expression of sixteen LY6 family members significantly associated with lower OS outcome and high expression of four genes significantly associated with higher OS outcome. Future research is required to translate our DNA and mRNA analysis into a proteomic and molecular interaction context. With further research, these findings may lead to potential successful screening markers for PDAC, as well as new, personalized targeted therapy or guidance of current standard chemotherapy regimens based on LY6 gene expression.

## MATERIALS AND METHODS

### Overall survival (OS) outcome analysis

The Kaplan-Meier (KM) plotter (https://kmplot.com/analysis/) online web-based tools allows users to observe the association of mRNA expression to over survival outcome with or the context of tumor microenvironment cellular composition [18]. For our analysis, we selected PDAC dataset on 177 patients among the list of pan cancer dataset (TCGA collection) in the gene expression RNA sequencing (RNA-seq) tab on the homepage of KM plotter. This dataset was used to visualize the association of mRNA expression of the LY6 gene family with overall survival (OS) outcome in PDAC. The number of patients in low and high expression of indicated gene is displayed in the Supplementary Table 1. To estimate if inherent cellular content plays a role in the association of LY6 gene expression with the OS outcome, we used the restricted analysis feature of the KM Plotter tool. This feature allowed us to observe the OS outcome in patient samples with enriched or decreased cellular content which included mesenchymal stem cell, CD8+ T cells, macrophages, NK T cells, CD4+ memory T cells, regulatory T cells, and B cells. The number of patients in the various subgroups for low expressing genes were in the range of 43 to 127 and for high expressing genes were 51 to 134. The exact number of patients in each subgroup can be found in Supplementary Figure 1 through 8, which corresponds to the data presented in Supplementary Table 1, Tables 2–7.

### Disease free survival (DFS) analysis

RNA-seq normalized gene expression data at z-score level and the corresponding clinical data from the TCGA Pancreatic Adenocarcinoma cohort were downloaded from the cBioPortal (https://www.cbioportal.org/) [16, 17]. The TCGA dataset contained data from 179 patients, of which 139 had disease free survival (DFS) status, including one metastasis sample. Thus, 138 primary tumor samples were used for DFS analysis. DFS analysis was performed using the R statistical programming environment. We used the *cgdsr* R package (https://CRAN.R-project.org/package=cgdsr) in the R statistical programming software to download and query the data [52]. For each gene, the optimal cutoff into “low” and “high” expression was determined by inbuilt algorithm in the survMisc R package [53]. We used this data to explore the association of the LY6 genes with DFS outcome. The survival analysis and KM plots were done using the survMisc R package. Then the significance of the association of each gene with DFS outcome was reported using a log rank test with *p*-value < 0.05.

### Copy number alteration analysis

We analyzed the copy number alteration data from LY6 genes in TCGA PDAC in the cBioPortal, which included data for 183 patients and UTSW PDAC dataset, which contained data for 109 patients.

### Differential gene expression analysis

The Oncomine™ Platform (Thermo Fisher, Ann Arbor, MI) (https://www.oncomine.org [54]) was applied to assess the differential expression of LY6 genes between the pancreatic tumors and normal tissues from the Pei dataset [55]. Pei et al. performed differential gene expression analysis in 16 normal and 36 pancreatic cancer samples using a human genome U133 Plus 2.0 Array measuring 19574 genes, submitted as a public dataset in the NCBI Gene Expression Omnibus public repository GSE16515 [55].

## SUPPLEMENTARY MATERIALS






