# Supplementary material for: High mRNA expression of LY6 gene family is associated with overall survival outcome in pancreatic ductal adenocarcinoma
Source: Oncotarget. 2021 Feb 2;12(3):145–59. doi: 10.18632/oncotarget.27880 (PMC7869573; doi:10.18632/oncotarget.27880)
Supplement: Supplementary file 1 [file oncotarget-12-145-s001.pdf]

High mRNA expression of LY6 gene family is associated with overall survival outcome in pancreatic ductal adenocarcinoma

SUPPLEMENTARY MATERIALS

Supplementary Figure 1: KM plots and other raw data for the data depicted in Table 1. See Supplementary Figure

Supplementary Figure 2: KM plots and other raw data for the data depicted in Table 2. See Supplementary Figure

Supplementary Figure 3: KM plots and other raw data for the data depicted in Table 3. See Supplementary Figure

Supplementary Figure 4: KM plots and other raw data for the data depicted in Table 4. See Supplementary Figure

Supplementary Figure 5: KM plots and other raw data for the data depicted in Table 5. See Supplementary Figure

Supplementary Figure 6: KM plots and other raw data for the data depicted in Table 6. See Supplementary Figure

Supplementary Figure 7: KM plots and other raw data for the data depicted in Table 7. See Supplementary Figure

Supplementary Figure 8: KM plots and other raw data for the data depicted in Table 8. See Supplementary Figure

Supplementary Table 1: High mRNA expression of 30 LY6 genes and its association with OS outcome in the PDAC as seen in the TCGA dataset visualized by KM plotter tool. See Supplementary Table 1
