# Supplementary material for: High mRNA expression of LY6 gene family is associated with overall survival outcome in pancreatic ductal adenocarcinoma
Source: Oncotarget. 2021 Feb 2;12(3):145–59. doi: 10.18632/oncotarget.27880 (PMC7869573; doi:10.18632/oncotarget.27880)
Supplement: Supplementary file 2 [file oncotarget-12-145-s002.pdf]

Figure S1: KM plots and other raw data for the data depicted in Table 1

Pan-cancer

KM plotter | Home | Vote | Download | Updates | Contact

The desired RNAseq ID is valid: PSCA (-), LY6K (-), SLURP1 (-), LYPD2 (-), LY6D (-), GML (-), LY6E (-), LY6L (-), LY6H (-), GPIHBP1 (-), LYPD4 (-), CD177 (-), TEX101 (-), LYPD3 (-), PINLYP (-), PLAUR (-), LYPD5 (-), SPACA4 (-), ACRV1 (-), PATE1 (-), PATE2 (-), PATE3 (-), PATE4 (-), CD59 (-), LY6G6C (-), LY6G6D (-), LY6G6F (-), LY6G5C (-), LY6G5B (-),

**RNAseq ID:**	PSCA -
**Survival:**	OS
**Auto select best cutoff:**	checked
**Follow up threshold:**	all
**Censore at threshold:**	checked
**Compute median over entire database:**	false
**Cutoff value used in analysis:**	350
**Expression range of the probe:**	0 - 65661
**Invert HR values below 1:**	not checked

Restrictions

Tumor type: Pancreatic ductal adenocarcinoma

Restrict analysis to subtypes...

Stage:	all
Gender:	all
Race:	all
Grade:	all
Mutation burden:	all

Restrict analysis based on cellular content...

Basophils:	all
B-cells:	all
CD4+ memory T-cells:	all
CD8+ T-cells:	all
Eosinophils:	all
Macrophages:	all
Mesenchymal stem cells:	all
Natural killer T-cells:	all
Regulatory T-cells:	all
Type 1 T-helper cells:	all
Type 2 T-helper cells:	all

Results

P value: 6.0e-5
FDR: 1%

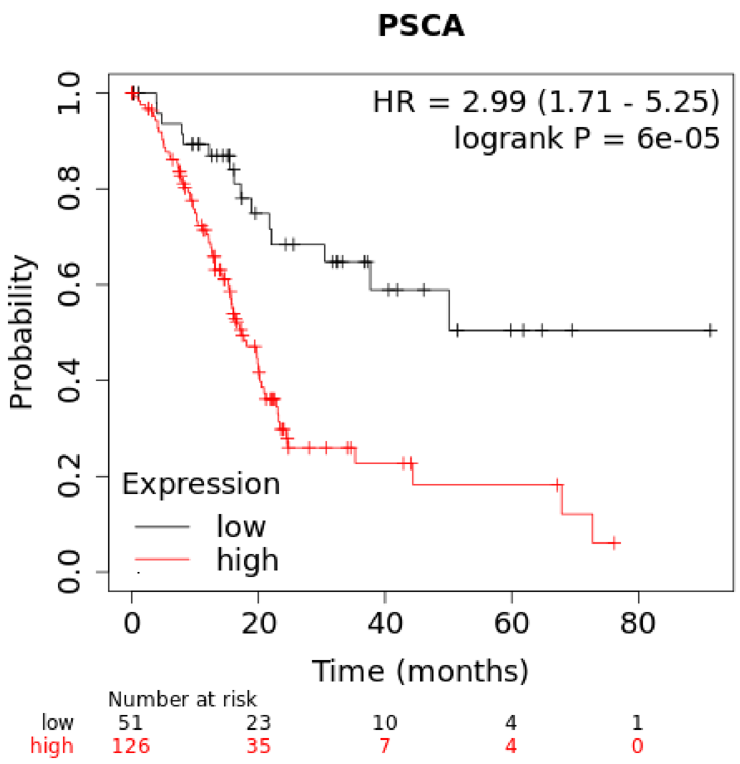

Click here to download the plot in TIFF format

Download plot as a PDF

Download p values vs. cutoff table

Upper quartile survival

Low expression cohort (months)	High expression cohort (months)
18.93	9.97

RNAseq ID: LY6K -
Survival: OS
Auto select best cutoff: checked
Follow up threshold: all
Censore at threshold: checked
Compute median over entire database: false
Cutoff value used in analysis: 6
Expression range of the probe: 0 - 1825
Invert HR values below 1: not checked

Restrictions

Tumor type: Pancreatic ductal adenocarcinoma

Restrict analysis to subtypes...

Stage: all
Gender: all
Race: all
Grade: all
Mutation burden: all

Restrict analysis based on cellular content...

Basophils: all

B-cells:	all
CD4+ memory T-cells:	all
CD8+ T-cells:	all
Eosinophils:	all
Macrophages:	all
Mesenchymal stem cells:	all
Natural killer T-cells:	all
Regulatory T-cells:	all
Type 1 T-helper cells:	all
Type 2 T-helper cells:	all

Results

P value: 0.1082
FDR: 100%

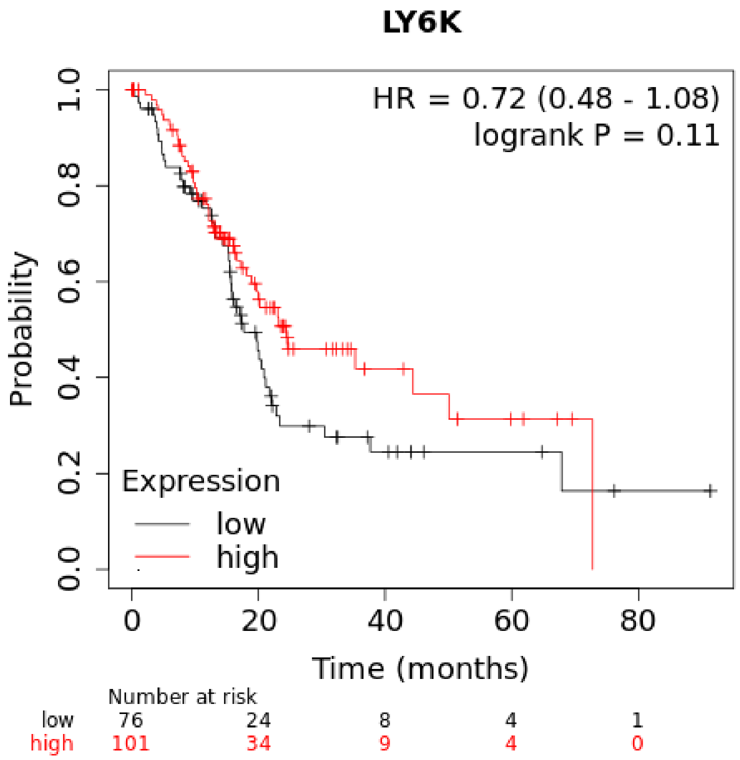

Click here to download the plot in TIFF format

Download plot as a PDF

Download p values vs. cutoff table

Median survival

Low expression cohort (months)	High expression cohort (months)
17.73	24.4

**RNAseq ID:**	SLURP1 -
**Survival:**	OS
**Auto select best cutoff:**	checked
**Follow up threshold:**	all
**Censore at threshold:**	checked
**Compute median over entire database:**	false
**Cutoff value used in analysis:**	1
**Expression range of the probe:**	0 - 279
**Invert HR values below 1:**	not checked

Restrictions

Tumor type: Pancreatic ductal adenocarcinoma

Restrict analysis to subtypes...

Stage:	all
Gender:	all
Race:	all
Grade:	all
Mutation burden:	all

Restrict analysis based on cellular content...

Basophils:	all
B-cells:	all
CD4+ memory T-cells:	all
CD8+ T-cells:	all
Eosinophils:	all
Macrophages:	all
Mesenchymal stem cells:	all
Natural killer T-cells:	all
Regulatory T-cells:	all
Type 1 T-helper cells:	all
Type 2 T-helper cells:	all

Results

P value: 3.9e-6
FDR: 1%

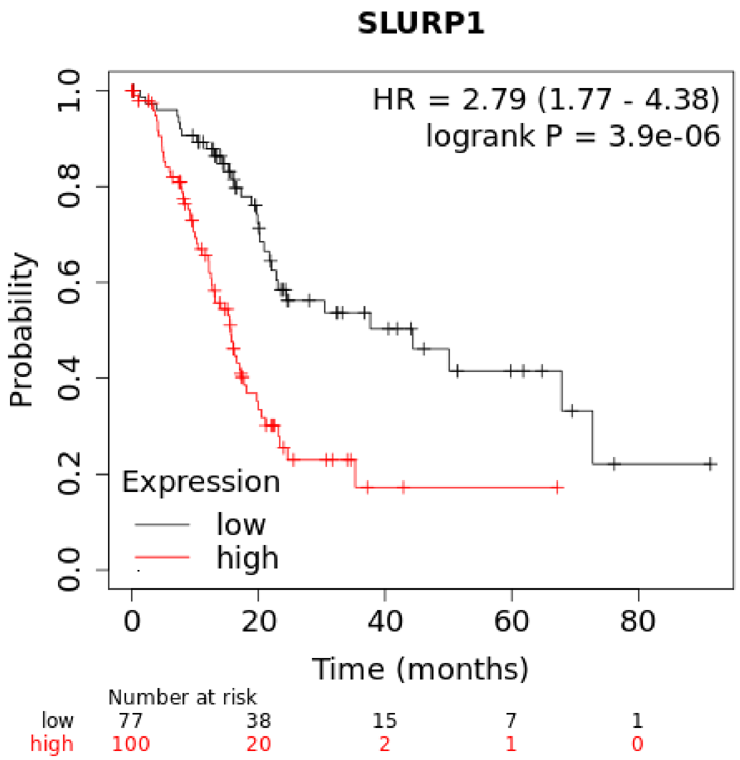

Click here to download the plot in TIFF format

Download plot as a PDF

Download p values vs. cutoff table

Median survival

Low expression cohort (months)	High expression cohort (months)
44.4	15.67

**RNAseq ID:**	LYPD2	-
**Survival:**	OS	
**Auto select best cutoff:**	checked	
**Follow up threshold:**	all	
**Censore at threshold:**	checked	
**Compute median over entire database:**	false	
**Cutoff value used in analysis:**	4	
**Expression range of the probe:**	0 - 4748	
**Invert HR values below 1:**	not checked	

Restrictions

Tumor type: Pancreatic ductal adenocarcinoma

Restrict analysis to subtypes...

Stage:	all
Gender:	all
Race:	all
Grade:	all
Mutation burden:	all

Restrict analysis based on cellular content...

Basophils:	all
B-cells:	all
CD4+ memory T-cells:	all
CD8+ T-cells:	all
Eosinophils:	all
Macrophages:	all
Mesenchymal stem cells:	all
Natural killer T-cells:	all
Regulatory T-cells:	all
Type 1 T-helper cells:	all
Type 2 T-helper cells:	all

Results

**P value:**	0.0034
**FDR:**	50%

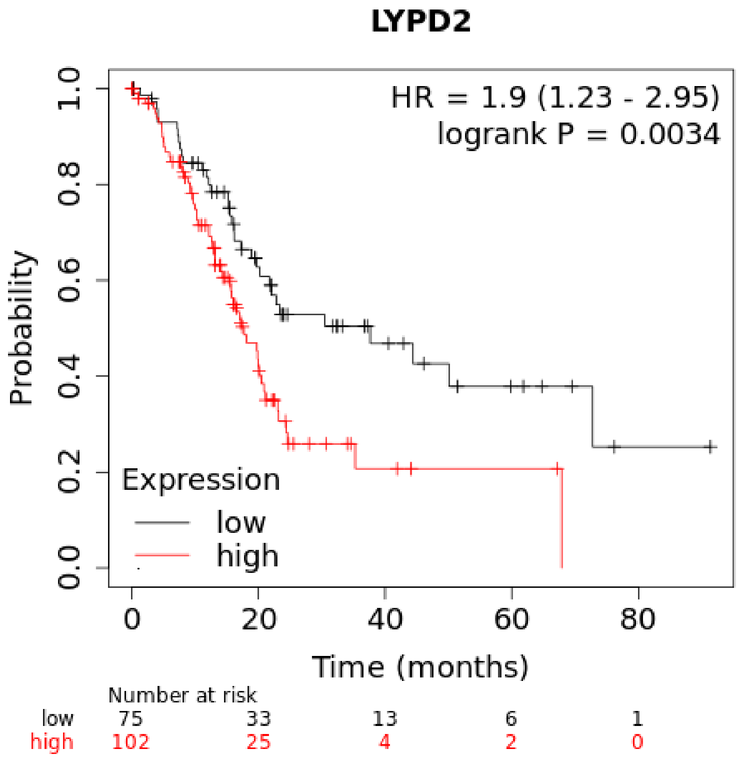

Click here to download the plot in TIFF format

Download plot as a PDF

Download p values vs. cutoff table

Median survival

Low expression cohort (months)	High expression cohort (months)
37.67	17.73

**RNAseq ID:**	LY6D -
**Survival:**	OS
**Auto select best cutoff:**	checked
**Follow up threshold:**	all
**Censore at threshold:**	checked
**Compute median over entire database:**	false
**Cutoff value used in analysis:**	160
**Expression range of the probe:**	0 - 18030
**Invert HR values below 1:**	not checked

Restrictions

Tumor type: Pancreatic ductal adenocarcinoma

Restrict analysis to subtypes...

Stage:	all
Gender:	all
Race:	all
Grade:	all
Mutation burden:	all

Restrict analysis based on cellular content...

Basophils:	all

B-cells:	all
CD4+ memory T-cells:	all
CD8+ T-cells:	all
Eosinophils:	all
Macrophages:	all
Mesenchymal stem cells:	all
Natural killer T-cells:	all
Regulatory T-cells:	all
Type 1 T-helper cells:	all
Type 2 T-helper cells:	all

Results

P value: 2.0e-6
FDR: 1%

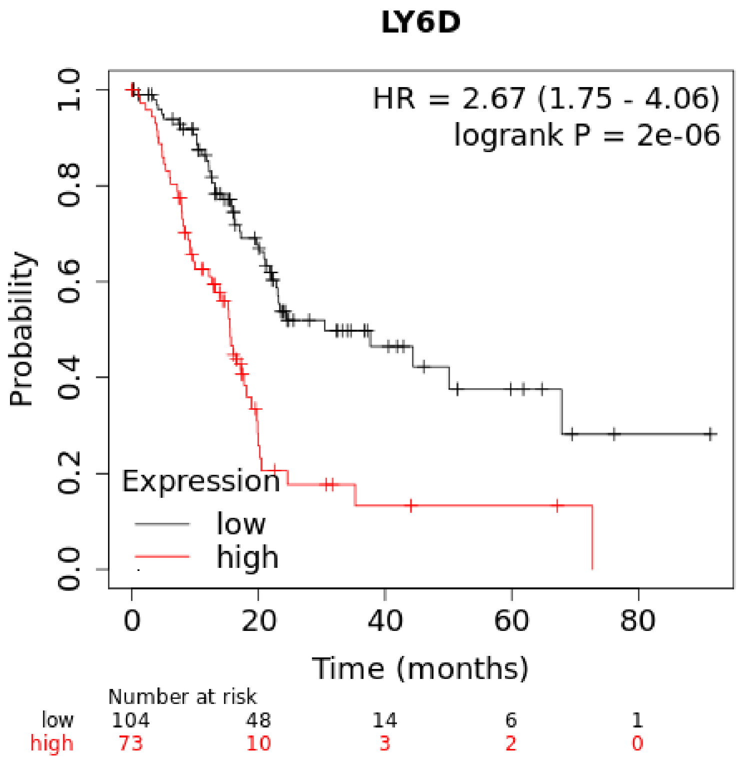

Click here to download the plot in TIFF format

Download plot as a PDF

Download p values vs. cutoff table

Median survival

Low expression cohort (months)	High expression cohort (months)
30.43	15.57

**RNAseq ID:**	GML -
**Survival:**	OS
**Auto select best cutoff:**	checked
**Follow up threshold:**	all
**Censore at threshold:**	checked
**Compute median over entire database:**	false
**Cutoff value used in analysis:**	0
**Expression range of the probe:**	0 - 3
**Invert HR values below 1:**	not checked

Restrictions

Tumor type: Pancreatic ductal adenocarcinoma

Restrict analysis to subtypes...

Stage:	all
Gender:	all
Race:	all
Grade:	all
Mutation burden:	all

Restrict analysis based on cellular content...

Basophils:	all
B-cells:	all
CD4+ memory T-cells:	all
CD8+ T-cells:	all
Eosinophils:	all
Macrophages:	all
Mesenchymal stem cells:	all
Natural killer T-cells:	all
Regulatory T-cells:	all
Type 1 T-helper cells:	all
Type 2 T-helper cells:	all

Results

P value: 0.0387
FDR: over 50%

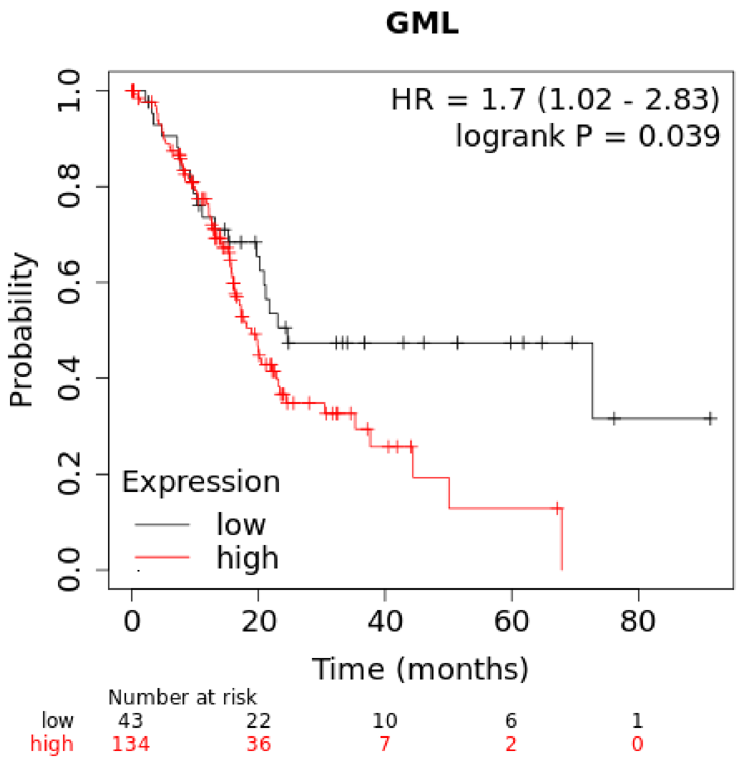

Click here to download the plot in TIFF format

Download plot as a PDF

Download p values vs. cutoff table

Median survival

Low expression cohort (months)	High expression cohort (months)
24.6	18.93

**RNAseq ID:**	LY6E -
**Survival:**	OS
**Auto select best cutoff:**	checked
**Follow up threshold:**	all
**Censore at threshold:**	checked
**Compute median over entire database:**	false
**Cutoff value used in analysis:**	10191
**Expression range of the probe:**	254 - 56404
**Invert HR values below 1:**	not checked

Restrictions

Tumor type: Pancreatic ductal adenocarcinoma

Restrict analysis to subtypes...

Stage:	all
Gender:	all
Race:	all
Grade:	all
Mutation burden:	all

Restrict analysis based on cellular content...

Basophils:	all
B-cells:	all
CD4+ memory T-cells:	all
CD8+ T-cells:	all
Eosinophils:	all
Macrophages:	all
Mesenchymal stem cells:	all
Natural killer T-cells:	all
Regulatory T-cells:	all
Type 1 T-helper cells:	all
Type 2 T-helper cells:	all

Results

P value: 0.0017
FDR: 20%

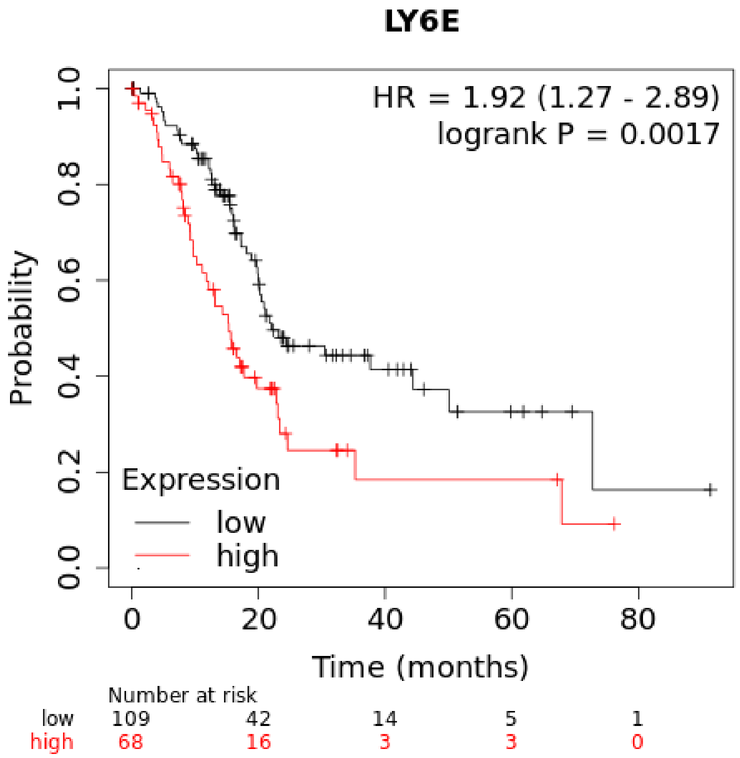

Click here to download the plot in TIFF format

Download plot as a PDF

Download p values vs. cutoff table

Median survival

Low expression cohort (months)	High expression cohort (months)
22.03	15.33

**RNAseq ID:**	LY6L -
**Survival:**	OS
**Auto select best cutoff:**	checked
**Follow up threshold:**	all
**Censore at threshold:**	checked
**Compute median over entire database:**	false
**Cutoff value used in analysis:**	0
**Expression range of the probe:**	0 - 8
**Invert HR values below 1:**	not checked

Restrictions

Tumor type: Pancreatic ductal adenocarcinoma

Restrict analysis to subtypes...

Stage:	all
Gender:	all
Race:	all
Grade:	all
Mutation burden:	all

Restrict analysis based on cellular content...

Basophils:	all

B-cells:	all
CD4+ memory T-cells:	all
CD8+ T-cells:	all
Eosinophils:	all
Macrophages:	all
Mesenchymal stem cells:	all
Natural killer T-cells:	all
Regulatory T-cells:	all
Type 1 T-helper cells:	all
Type 2 T-helper cells:	all

Results

P value: 0.0189
FDR: over 50%

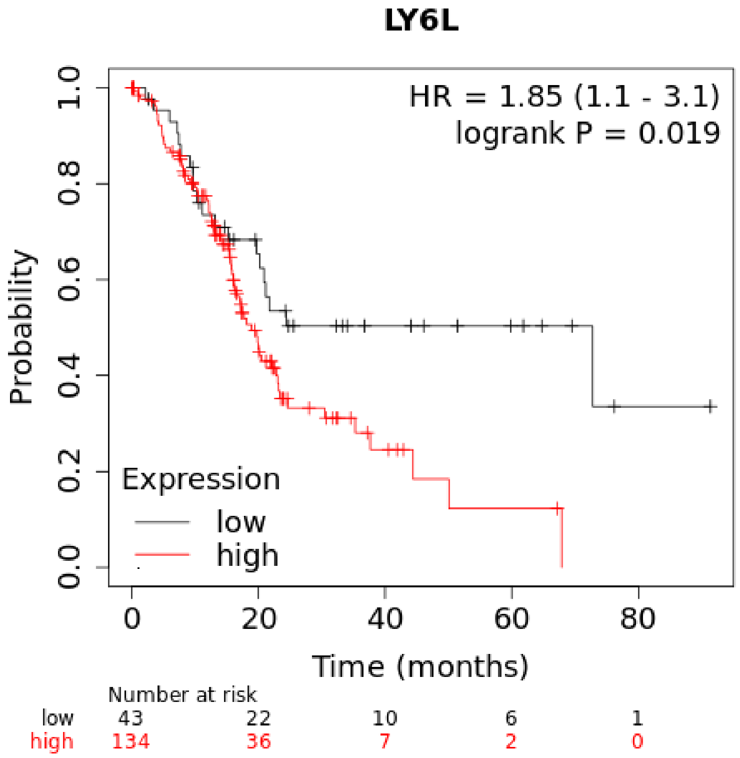

Click here to download the plot in TIFF format

Download plot as a PDF

Download p values vs. cutoff table

Median survival

Low expression cohort (months)	High expression cohort (months)
72.73	18.93

**RNAseq ID:**	LY6H -
**Survival:**	OS
**Auto select best cutoff:**	checked
**Follow up threshold:**	all
**Censore at threshold:**	checked
**Compute median over entire database:**	false
**Cutoff value used in analysis:**	39
**Expression range of the probe:**	1 - 9495
**Invert HR values below 1:**	not checked

Restrictions

Tumor type: Pancreatic ductal adenocarcinoma

Restrict analysis to subtypes...

Stage: all
Gender: all
Race: all
Grade: all
Mutation burden: all

Restrict analysis based on cellular content...

Basophils: all
B-cells: all
CD4+ memory T-cells: all
CD8+ T-cells: all
Eosinophils: all
Macrophages: all
Mesenchymal stem cells: all
Natural killer T-cells: all
Regulatory T-cells: all
Type 1 T-helper cells: all
Type 2 T-helper cells: all

Results

P value: 0.0037
FDR: 50%

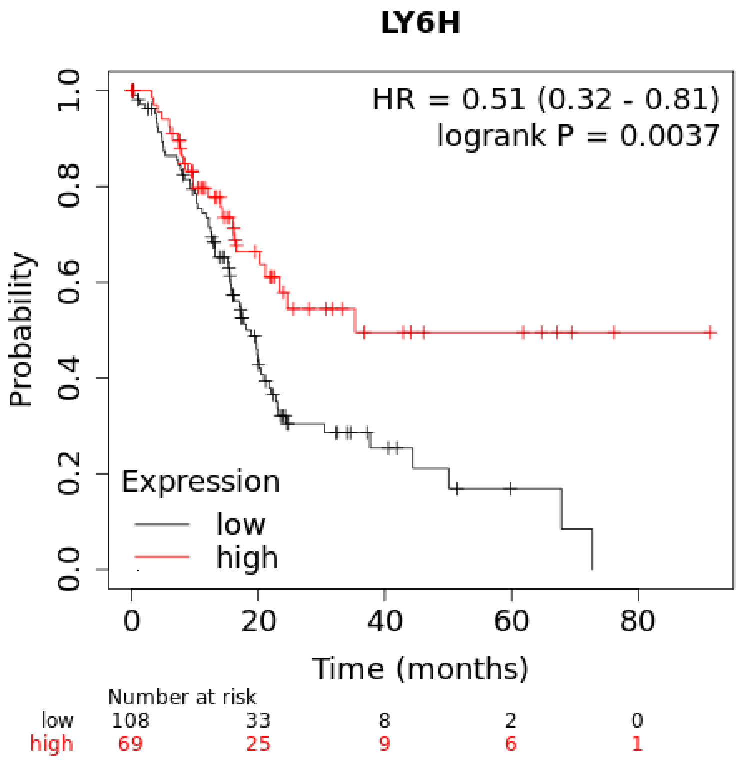

Click here to download the plot in TIFF format

Download plot as a PDF

Download p values vs. cutoff table

Median survival

Low expression cohort (months)	High expression cohort (months)
18.17	35.3

**RNAseq ID:**	GPIHBP1 -
**Survival:**	OS
**Auto select best cutoff:**	checked
**Follow up threshold:**	all
**Censore at threshold:**	checked
**Compute median over entire database:**	false
**Cutoff value used in analysis:**	32
**Expression range of the probe:**	5 - 344
**Invert HR values below 1:**	not checked

Restrictions

Tumor type: Pancreatic ductal adenocarcinoma

Restrict analysis to subtypes...

Stage:	all
Gender:	all
Race:	all
Grade:	all
Mutation burden:	all

Restrict analysis based on cellular content...

Basophils:	all
B-cells:	all
CD4+ memory T-cells:	all
CD8+ T-cells:	all
Eosinophils:	all
Macrophages:	all
Mesenchymal stem cells:	all
Natural killer T-cells:	all
Regulatory T-cells:	all
Type 1 T-helper cells:	all
Type 2 T-helper cells:	all

Results

P value: 0.1439
FDR: 100%

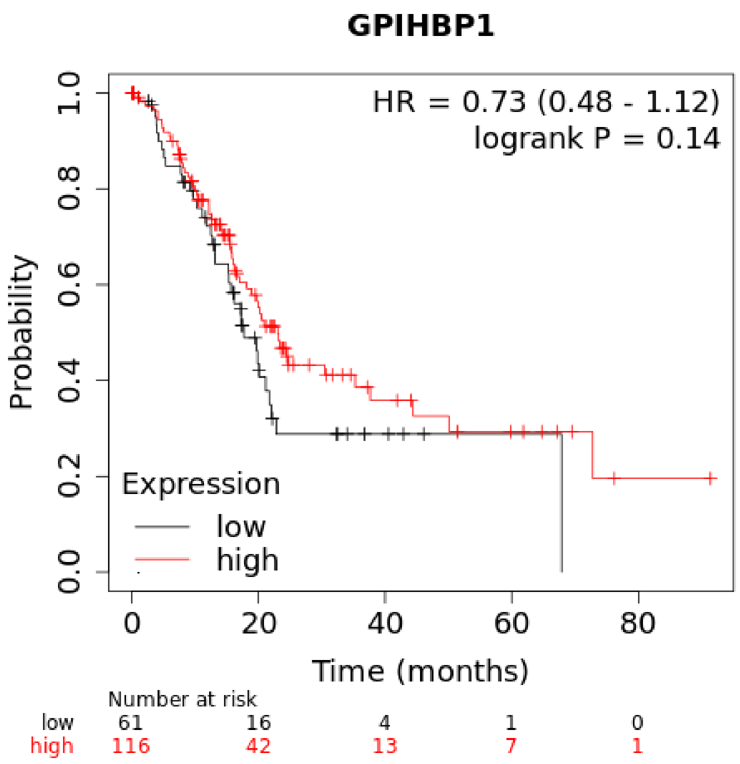

Click here to download the plot in TIFF format

Download plot as a PDF

Download p values vs. cutoff table

Median survival

Low expression cohort (months)	High expression cohort (months)
17.73	23.03

RNAseq ID: LYPD4 -
Survival: OS
Auto select best cutoff: checked
Follow up threshold: all
Censore at threshold: checked
Compute median over entire database: false
Cutoff value used in analysis: 0
Expression range of the probe: 0 - 18
Invert HR values below 1: not checked

Restrictions

Tumor type: Pancreatic ductal adenocarcinoma

Restrict analysis to subtypes...

Stage: all
Gender: all
Race: all
Grade: all
Mutation burden: all

Restrict analysis based on cellular content...

Basophils: all

B-cells:	all
CD4+ memory T-cells:	all
CD8+ T-cells:	all
Eosinophils:	all
Macrophages:	all
Mesenchymal stem cells:	all
Natural killer T-cells:	all
Regulatory T-cells:	all
Type 1 T-helper cells:	all
Type 2 T-helper cells:	all

Results

P value: 0.0387
FDR: over 50%

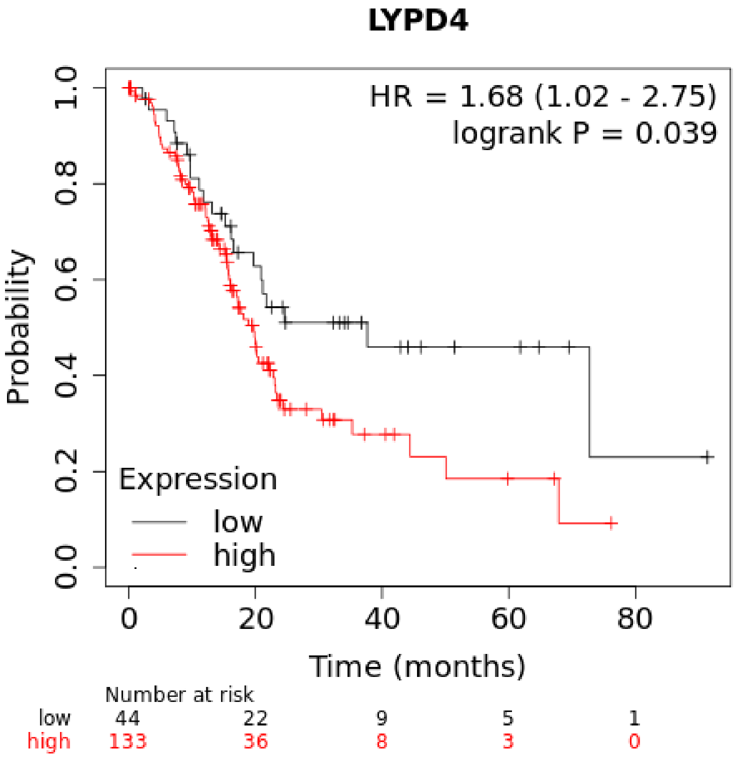

Click here to download the plot in TIFF format

Download plot as a PDF

Download p values vs. cutoff table

Median survival

Low expression cohort (months)	High expression cohort (months)
37.67	19.77

**RNAseq ID:**	CD177 -
**Survival:**	OS
**Auto select best cutoff:**	checked
**Follow up threshold:**	all
**Censore at threshold:**	checked
**Compute median over entire database:**	false
**Cutoff value used in analysis:**	18
**Expression range of the probe:**	0 - 6100
**Invert HR values below 1:**	not checked

Restrictions

Tumor type: Pancreatic ductal adenocarcinoma

Restrict analysis to subtypes...

Stage:	all
Gender:	all
Race:	all
Grade:	all
Mutation burden:	all

Restrict analysis based on cellular content...

Basophils:	all
B-cells:	all
CD4+ memory T-cells:	all
CD8+ T-cells:	all
Eosinophils:	all
Macrophages:	all
Mesenchymal stem cells:	all
Natural killer T-cells:	all
Regulatory T-cells:	all
Type 1 T-helper cells:	all
Type 2 T-helper cells:	all

Results

P value: 0.0938
FDR: 100%

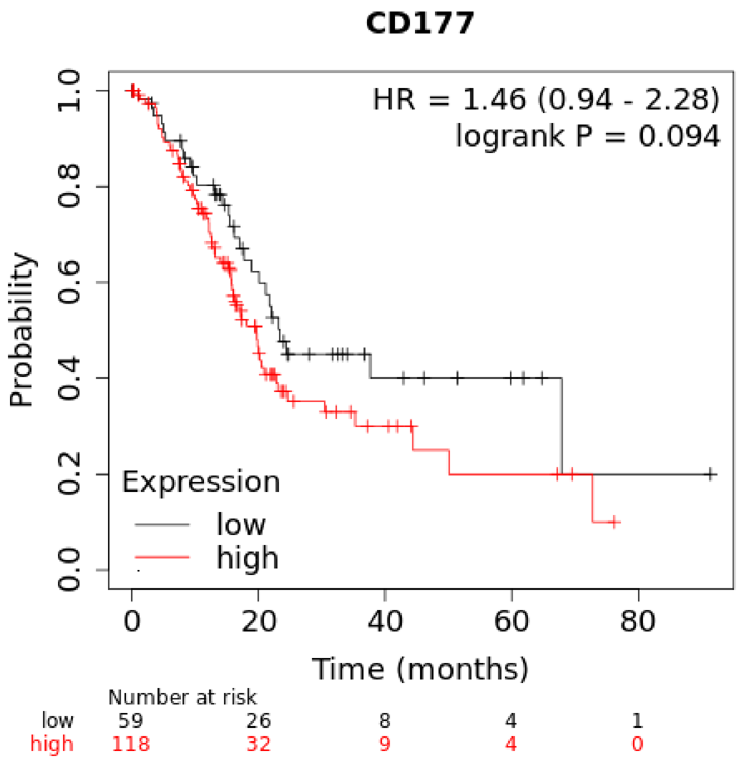

Click here to download the plot in TIFF format

Download plot as a PDF

Download p values vs. cutoff table

Median survival

Low expression cohort (months)	High expression cohort (months)
23.4	19.73

**RNAseq ID:**	TEX101 -
**Survival:**	OS
**Auto select best cutoff:**	checked
**Follow up threshold:**	all
**Censore at threshold:**	checked
**Compute median over entire database:**	false
**Cutoff value used in analysis:**	1
**Expression range of the probe:**	0 - 149
**Invert HR values below 1:**	not checked

Restrictions

Tumor type: Pancreatic ductal adenocarcinoma

Restrict analysis to subtypes...

Stage:	all
Gender:	all
Race:	all
Grade:	all
Mutation burden:	all

Restrict analysis based on cellular content...

Basophils:	all
B-cells:	all
CD4+ memory T-cells:	all
CD8+ T-cells:	all
Eosinophils:	all
Macrophages:	all
Mesenchymal stem cells:	all
Natural killer T-cells:	all
Regulatory T-cells:	all
Type 1 T-helper cells:	all
Type 2 T-helper cells:	all

Results

**P value:**	0.2076
**FDR:**	100%

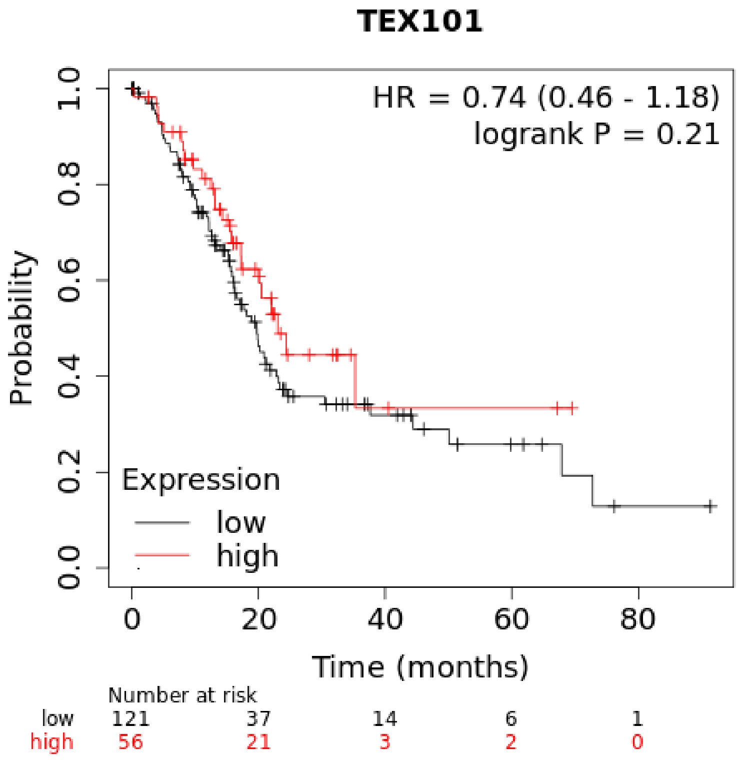

Click here to download the plot in TIFF format

Download plot as a PDF

Download p values vs. cutoff table

Median survival

Low expression cohort (months)	High expression cohort (months)
19.77	23.03

**RNAseq ID:**	LYPD3 -
**Survival:**	OS
**Auto select best cutoff:**	checked
**Follow up threshold:**	all
**Censore at threshold:**	checked
**Compute median over entire database:**	false
**Cutoff value used in analysis:**	144
**Expression range of the probe:**	9 - 7684
**Invert HR values below 1:**	not checked

Restrictions

Tumor type: Pancreatic ductal adenocarcinoma

Restrict analysis to subtypes...

Stage:	all
Gender:	all
Race:	all
Grade:	all
Mutation burden:	all

Restrict analysis based on cellular content...

Basophils:	all

B-cells:	all
CD4+ memory T-cells:	all
CD8+ T-cells:	all
Eosinophils:	all
Macrophages:	all
Mesenchymal stem cells:	all
Natural killer T-cells:	all
Regulatory T-cells:	all
Type 1 T-helper cells:	all
Type 2 T-helper cells:	all

Results

P value: 0.179
FDR: 100%

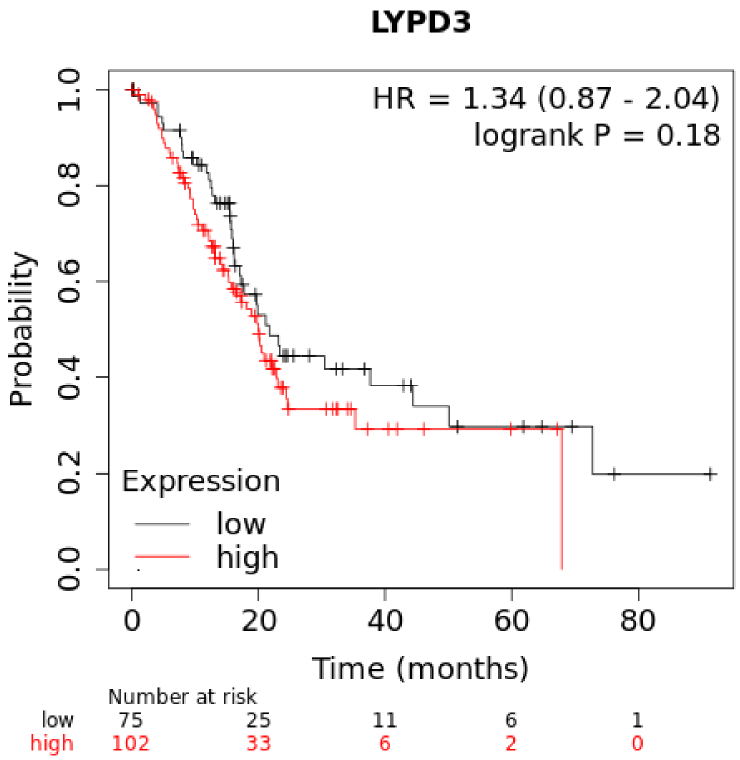

Click here to download the plot in TIFF format

Download plot as a PDF

Download p values vs. cutoff table

Median survival

Low expression cohort (months)	High expression cohort (months)
21.73	19.93

**RNAseq ID:**	PINLYP -
**Survival:**	OS
**Auto select best cutoff:**	checked
**Follow up threshold:**	all
**Censore at threshold:**	checked
**Compute median over entire database:**	false
**Cutoff value used in analysis:**	76
**Expression range of the probe:**	5 - 387
**Invert HR values below 1:**	not checked

Restrictions

Tumor type: Pancreatic ductal adenocarcinoma

Restrict analysis to subtypes...

Stage:	all
Gender:	all
Race:	all
Grade:	all
Mutation burden:	all

Restrict analysis based on cellular content...

Basophils:	all
B-cells:	all
CD4+ memory T-cells:	all
CD8+ T-cells:	all
Eosinophils:	all
Macrophages:	all
Mesenchymal stem cells:	all
Natural killer T-cells:	all
Regulatory T-cells:	all
Type 1 T-helper cells:	all
Type 2 T-helper cells:	all

Results

P value: 0.0359
FDR: over 50%

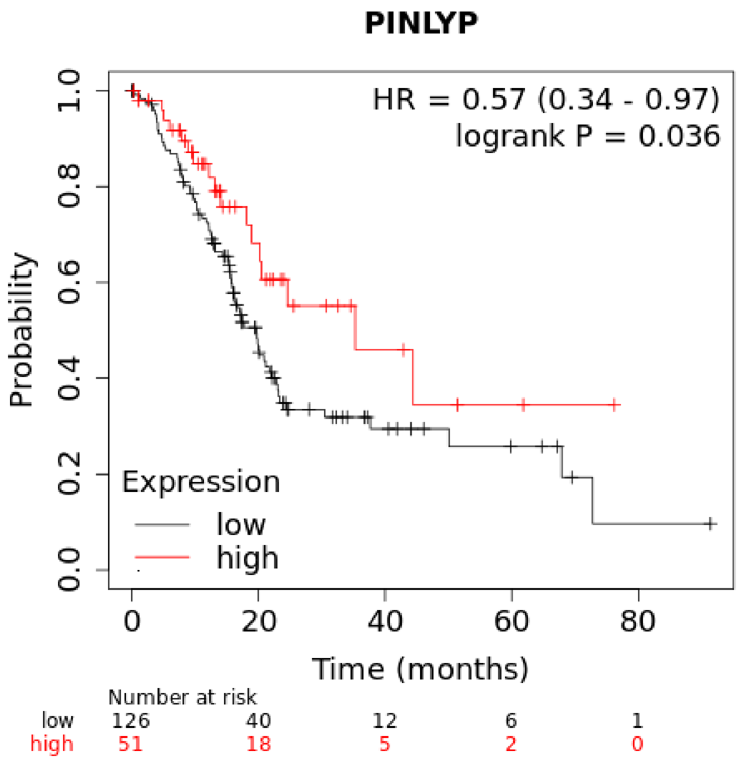

Click here to download the plot in TIFF format

Download plot as a PDF

Download p values vs. cutoff table

Median survival

Low expression cohort (months)	High expression cohort (months)
19.73	35.3

**RNAseq ID:**	PLAUR -
**Survival:**	OS
**Auto select best cutoff:**	checked
**Follow up threshold:**	all
**Censore at threshold:**	checked
**Compute median over entire database:**	false
**Cutoff value used in analysis:**	1750
**Expression range of the probe:**	47 - 18314
**Invert HR values below 1:**	not checked

Restrictions

Tumor type: Pancreatic ductal adenocarcinoma

Restrict analysis to subtypes...

Stage:	all
Gender:	all
Race:	all
Grade:	all
Mutation burden:	all

Restrict analysis based on cellular content...

Basophils:	all
B-cells:	all
CD4+ memory T-cells:	all
CD8+ T-cells:	all
Eosinophils:	all
Macrophages:	all
Mesenchymal stem cells:	all
Natural killer T-cells:	all
Regulatory T-cells:	all
Type 1 T-helper cells:	all
Type 2 T-helper cells:	all

Results

**P value:**	0.0099
**FDR:**	over 50%

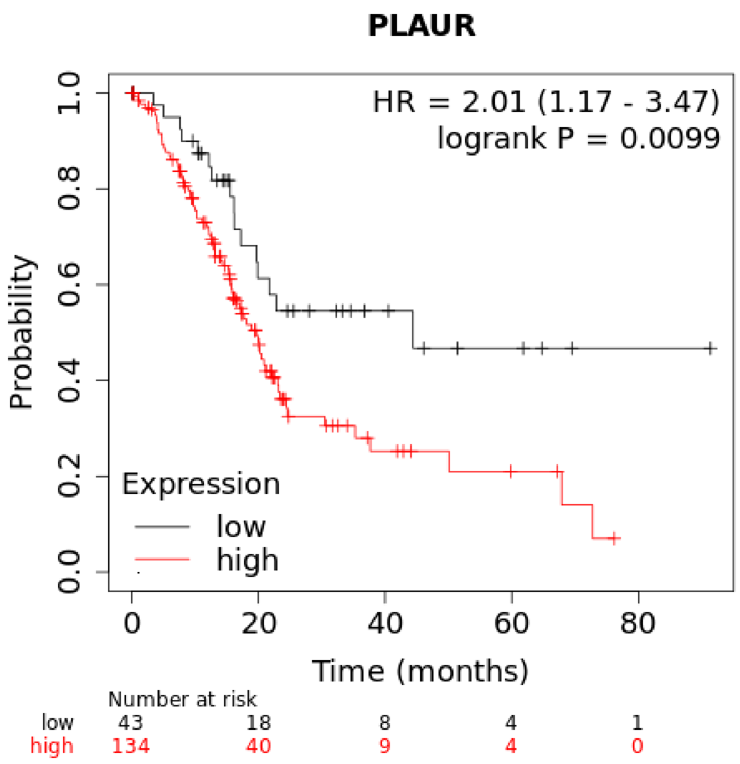

[Click here to download the plot in TIFF format]

[Download plot as a PDF]

[Download p values vs. cutoff table]

Median survival

Low expression cohort (months)	High expression cohort (months)
44.4	19.77

**RNAseq ID:**	LYPD5 -
**Survival:**	OS
**Auto select best cutoff:**	checked
**Follow up threshold:**	all
**Censore at threshold:**	checked
**Compute median over entire database:**	false
**Cutoff value used in analysis:**	78
**Expression range of the probe:**	1 - 578
**Invert HR values below 1:**	not checked

Restrictions

Tumor type: Pancreatic ductal adenocarcinoma

Restrict analysis to subtypes...

Stage:	all
Gender:	all
Race:	all
Grade:	all
Mutation burden:	all

Restrict analysis based on cellular content...

Basophils:	all

B-cells:	all
CD4+ memory T-cells:	all
CD8+ T-cells:	all
Eosinophils:	all
Macrophages:	all
Mesenchymal stem cells:	all
Natural killer T-cells:	all
Regulatory T-cells:	all
Type 1 T-helper cells:	all
Type 2 T-helper cells:	all

Results

P value: 0.0013
FDR: 10%

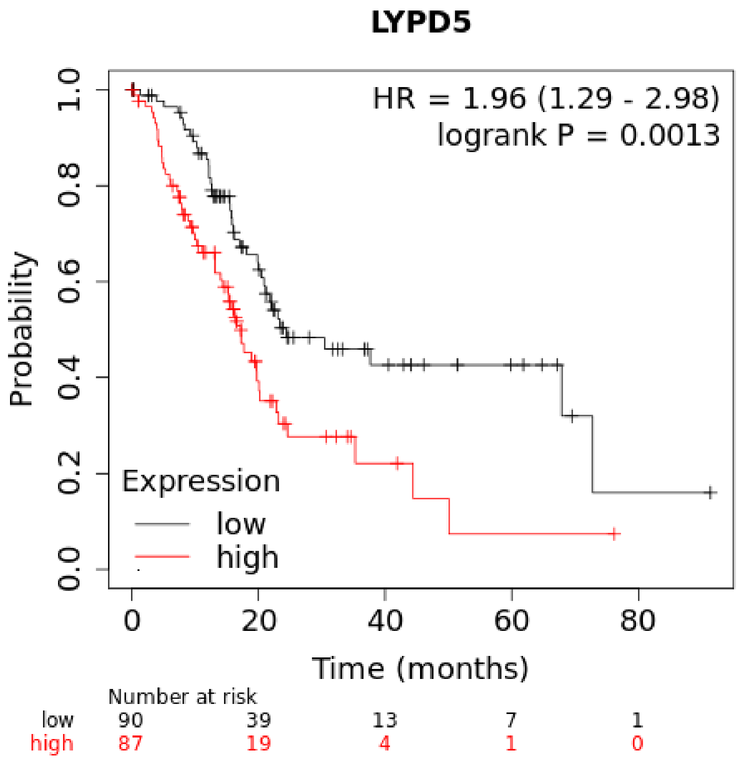

Click here to download the plot in TIFF format

Download plot as a PDF

Download p values vs. cutoff table

Median survival

Low expression cohort (months)	High expression cohort (months)
24.4	17.23

**RNAseq ID:**	SPACA4 -
**Survival:**	OS
**Auto select best cutoff:**	checked
**Follow up threshold:**	all
**Censore at threshold:**	checked
**Compute median over entire database:**	false
**Cutoff value used in analysis:**	19
**Expression range of the probe:**	0 - 206
**Invert HR values below 1:**	not checked

Restrictions

Tumor type: Pancreatic ductal adenocarcinoma

Restrict analysis to subtypes...

Stage:	all
Gender:	all
Race:	all
Grade:	all
Mutation burden:	all

Restrict analysis based on cellular content...

Basophils:	all
B-cells:	all
CD4+ memory T-cells:	all
CD8+ T-cells:	all
Eosinophils:	all
Macrophages:	all
Mesenchymal stem cells:	all
Natural killer T-cells:	all
Regulatory T-cells:	all
Type 1 T-helper cells:	all
Type 2 T-helper cells:	all

Results

P value: 0.2424
FDR: 100%

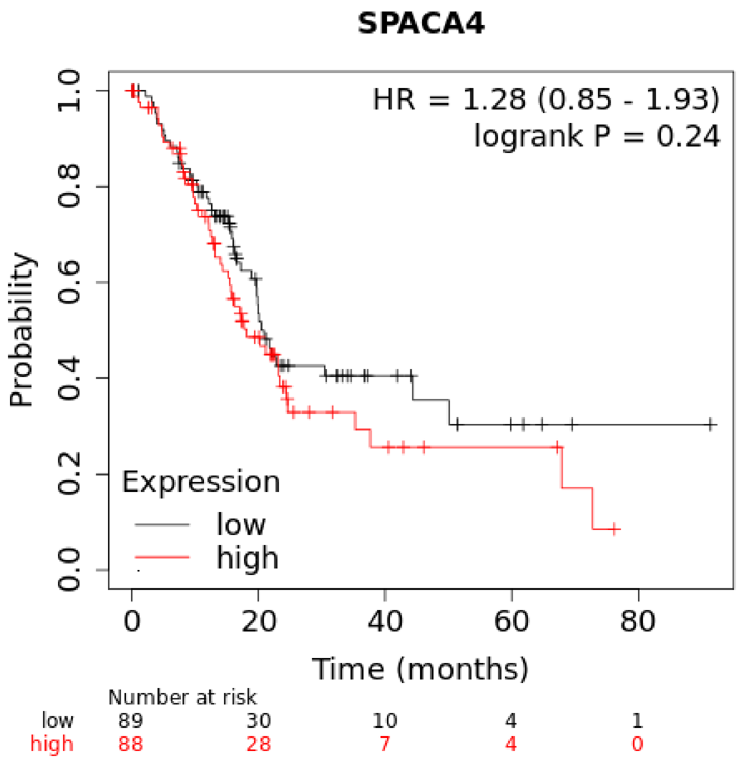

Click here to download the plot in TIFF format

Download plot as a PDF

Download p values vs. cutoff table

Median survival

Low expression cohort (months)	High expression cohort (months)
20.47	18.17

**RNAseq ID:**	ACRV1 -
**Survival:**	OS
**Auto select best cutoff:**	checked
**Follow up threshold:**	all
**Censore at threshold:**	checked
**Compute median over entire database:**	false
**Cutoff value used in analysis:**	4
**Expression range of the probe:**	0 - 71
**Invert HR values below 1:**	not checked

Restrictions

Tumor type: Pancreatic ductal adenocarcinoma

Restrict analysis to subtypes...

Stage:	all
Gender:	all
Race:	all
Grade:	all
Mutation burden:	all

Restrict analysis based on cellular content...

Basophils:	all
B-cells:	all
CD4+ memory T-cells:	all
CD8+ T-cells:	all
Eosinophils:	all
Macrophages:	all
Mesenchymal stem cells:	all
Natural killer T-cells:	all
Regulatory T-cells:	all
Type 1 T-helper cells:	all
Type 2 T-helper cells:	all

Results

**P value:**	0.0655
**FDR:**	100%

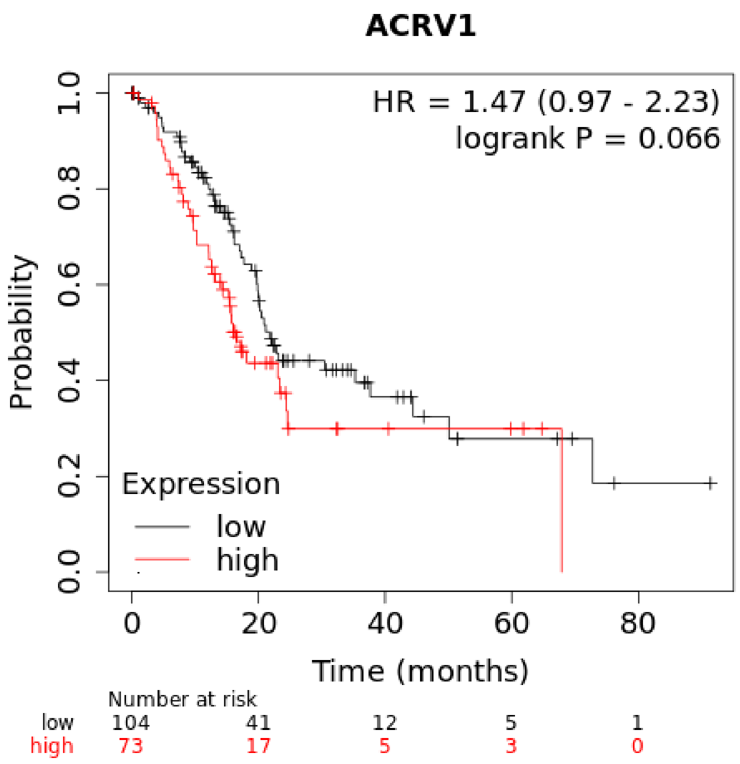

Click here to download the plot in TIFF format

Download plot as a PDF

Download p values vs. cutoff table

Median survival

Low expression cohort (months)	High expression cohort (months)
21.73	16.6

**RNAseq ID:**	PATE1 -
**Survival:**	OS
**Auto select best cutoff:**	checked
**Follow up threshold:**	all
**Censore at threshold:**	checked
**Compute median over entire database:**	false
**Cutoff value used in analysis:**	0
**Expression range of the probe:**	0 - 1
**Invert HR values below 1:**	not checked

Restrictions

Tumor type: Pancreatic ductal adenocarcinoma

Restrict analysis to subtypes...

Stage:	all
Gender:	all
Race:	all
Grade:	all
Mutation burden:	all

Restrict analysis based on cellular content...

Basophils:	all

B-cells:	all
CD4+ memory T-cells:	all
CD8+ T-cells:	all
Eosinophils:	all
Macrophages:	all
Mesenchymal stem cells:	all
Natural killer T-cells:	all
Regulatory T-cells:	all
Type 1 T-helper cells:	all
Type 2 T-helper cells:	all

Results

P value: 0.0021
FDR: 50%

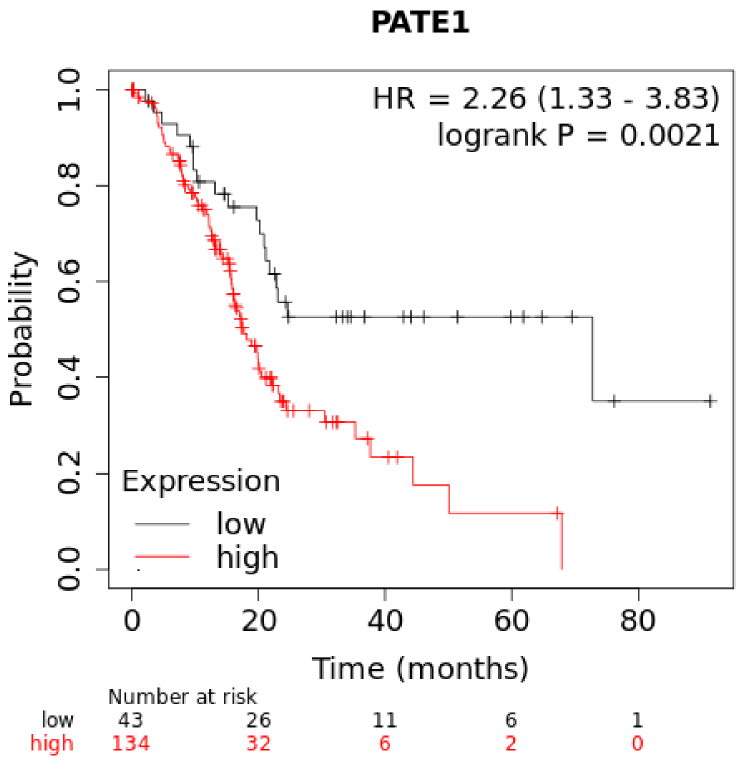

[Click here to download the plot in TIFF format]()

[Download plot as a PDF]()

[Download p values vs. cutoff table]()

Median survival

Low expression cohort (months)	High expression cohort (months)
72.73	17.73

**RNAseq ID:**	PATE2 -
**Survival:**	OS
**Auto select best cutoff:**	checked
**Follow up threshold:**	all
**Censore at threshold:**	checked
**Compute median over entire database:**	false
**Cutoff value used in analysis:**	1
**Expression range of the probe:**	0 - 7
**Invert HR values below 1:**	not checked

Restrictions

Tumor type: Pancreatic ductal adenocarcinoma

Restrict analysis to subtypes...

Stage:	all
Gender:	all
Race:	all
Grade:	all
Mutation burden:	all

Restrict analysis based on cellular content...

Basophils:	all
B-cells:	all
CD4+ memory T-cells:	all
CD8+ T-cells:	all
Eosinophils:	all
Macrophages:	all
Mesenchymal stem cells:	all
Natural killer T-cells:	all
Regulatory T-cells:	all
Type 1 T-helper cells:	all
Type 2 T-helper cells:	all

Results

P value: 0.0001
FDR: 5%

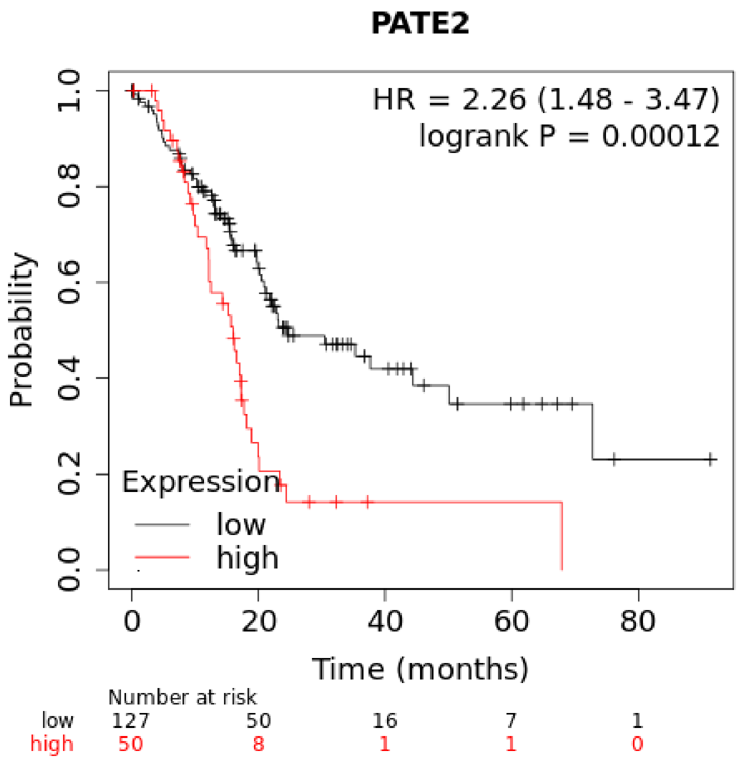

Click here to download the plot in TIFF format

Download plot as a PDF

Download p values vs. cutoff table

Median survival

Low expression cohort (months)	High expression cohort (months)
24.6	16.03

**RNAseq ID:**	PATE3 -
**Survival:**	OS
**Auto select best cutoff:**	checked
**Follow up threshold:**	all
**Censore at threshold:**	checked
**Compute median over entire database:**	false
**Cutoff value used in analysis:**	0
**Expression range of the probe:**	0 - 1
**Invert HR values below 1:**	not checked

Restrictions

Tumor type: Pancreatic ductal adenocarcinoma

Restrict analysis to subtypes...

Stage:	all
Gender:	all
Race:	all
Grade:	all
Mutation burden:	all

Restrict analysis based on cellular content...

Basophils:	all
B-cells:	all
CD4+ memory T-cells:	all
CD8+ T-cells:	all
Eosinophils:	all
Macrophages:	all
Mesenchymal stem cells:	all
Natural killer T-cells:	all
Regulatory T-cells:	all
Type 1 T-helper cells:	all
Type 2 T-helper cells:	all

Results

**P value:**	0.01
**FDR:**	over 50%

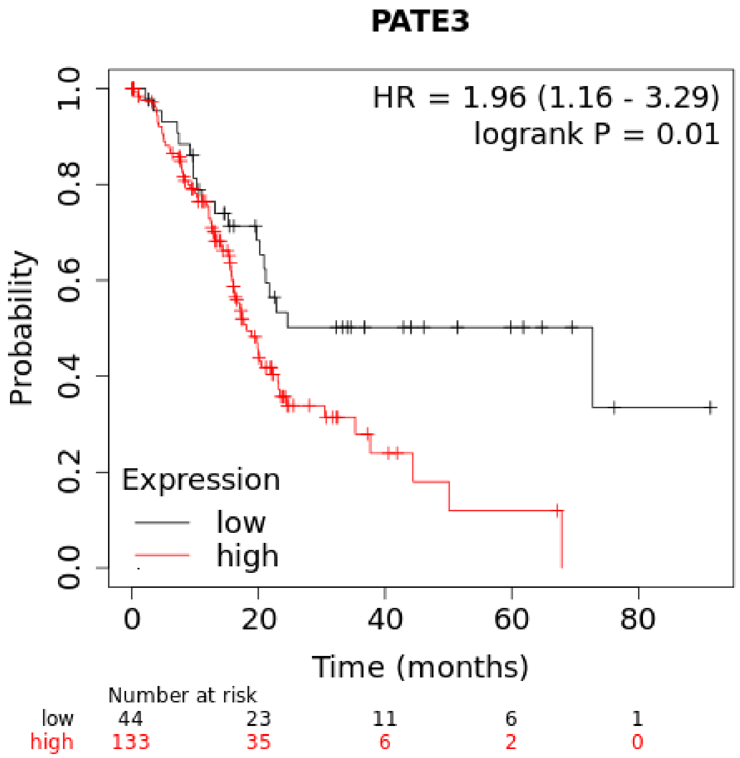

Click here to download the plot in TIFF format

Download plot as a PDF

Download p values vs. cutoff table

Median survival

Low expression cohort (months)	High expression cohort (months)
72.73	18.17

**RNAseq ID:**	PATE4 -
**Survival:**	OS
**Auto select best cutoff:**	checked
**Follow up threshold:**	all
**Censore at threshold:**	checked
**Compute median over entire database:**	false
**Cutoff value used in analysis:**	0
**Expression range of the probe:**	0 - 3
**Invert HR values below 1:**	not checked

Restrictions

Tumor type: Pancreatic ductal adenocarcinoma

Restrict analysis to subtypes...

Stage:	all
Gender:	all
Race:	all
Grade:	all
Mutation burden:	all

Restrict analysis based on cellular content...

Basophils:	all

B-cells:	all
CD4+ memory T-cells:	all
CD8+ T-cells:	all
Eosinophils:	all
Macrophages:	all
Mesenchymal stem cells:	all
Natural killer T-cells:	all
Regulatory T-cells:	all
Type 1 T-helper cells:	all
Type 2 T-helper cells:	all

Results

P value: 0.27
FDR: 100%

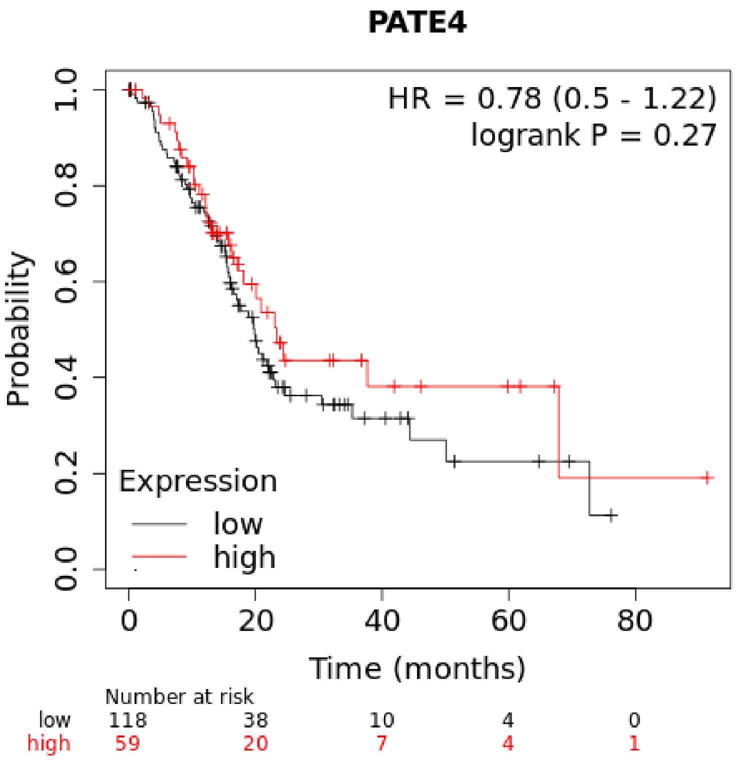

Click here to download the plot in TIFF format

Download plot as a PDF

Download p values vs. cutoff table

Median survival

Low expression cohort (months)	High expression cohort (months)
19.87	23.4

**RNAseq ID:**	CD59 -
**Survival:**	OS
**Auto select best cutoff:**	checked
**Follow up threshold:**	all
**Censore at threshold:**	checked
**Compute median over entire database:**	false
**Cutoff value used in analysis:**	17322
**Expression range of the probe:**	2610 - 39336
**Invert HR values below 1:**	not checked

Restrictions

Tumor type: Pancreatic ductal adenocarcinoma

Restrict analysis to subtypes...

Stage:	all
Gender:	all
Race:	all
Grade:	all
Mutation burden:	all

Restrict analysis based on cellular content...

Basophils:	all
B-cells:	all
CD4+ memory T-cells:	all
CD8+ T-cells:	all
Eosinophils:	all
Macrophages:	all
Mesenchymal stem cells:	all
Natural killer T-cells:	all
Regulatory T-cells:	all
Type 1 T-helper cells:	all
Type 2 T-helper cells:	all

Results

P value: 5.4e-5
FDR: 1%

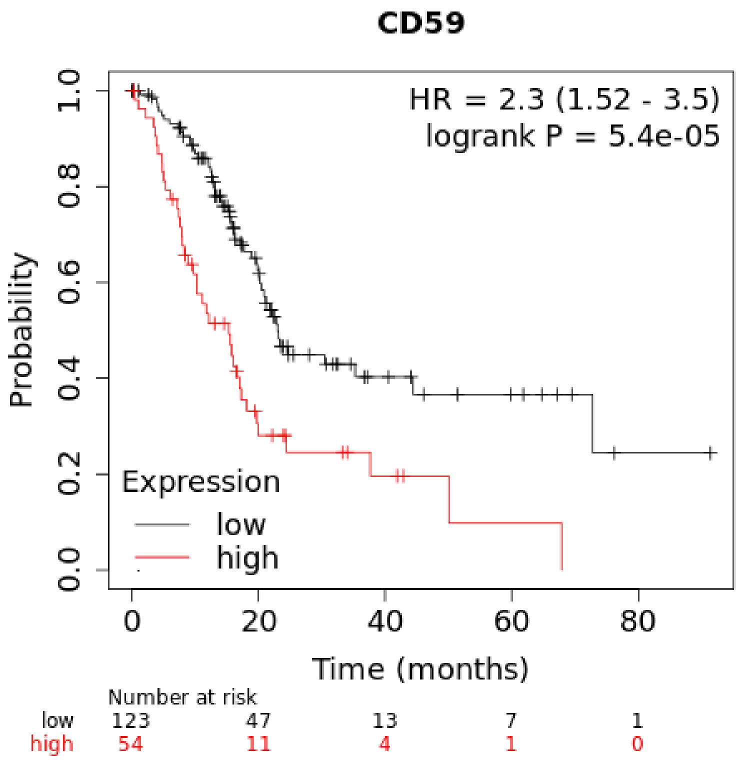

Click here to download the plot in TIFF format

Download plot as a PDF

Download p values vs. cutoff table

Median survival

Low expression cohort (months)	High expression cohort (months)
23.03	15.33

**RNAseq ID:**	LY6G6C	-
**Survival:**	OS	
**Auto select best cutoff:**	checked	
**Follow up threshold:**	all	
**Censore at threshold:**	checked	
**Compute median over entire database:**	false	
**Cutoff value used in analysis:**	13	
**Expression range of the probe:**	0 - 251	
**Invert HR values below 1:**	not checked	

Restrictions

Tumor type: Pancreatic ductal adenocarcinoma

Restrict analysis to subtypes...

Stage:	all
Gender:	all
Race:	all
Grade:	all
Mutation burden:	all

Restrict analysis based on cellular content...

Basophils:	all
B-cells:	all
CD4+ memory T-cells:	all
CD8+ T-cells:	all
Eosinophils:	all
Macrophages:	all
Mesenchymal stem cells:	all
Natural killer T-cells:	all
Regulatory T-cells:	all
Type 1 T-helper cells:	all
Type 2 T-helper cells:	all

Results

**P value:**	0.0065
**FDR:**	over 50%

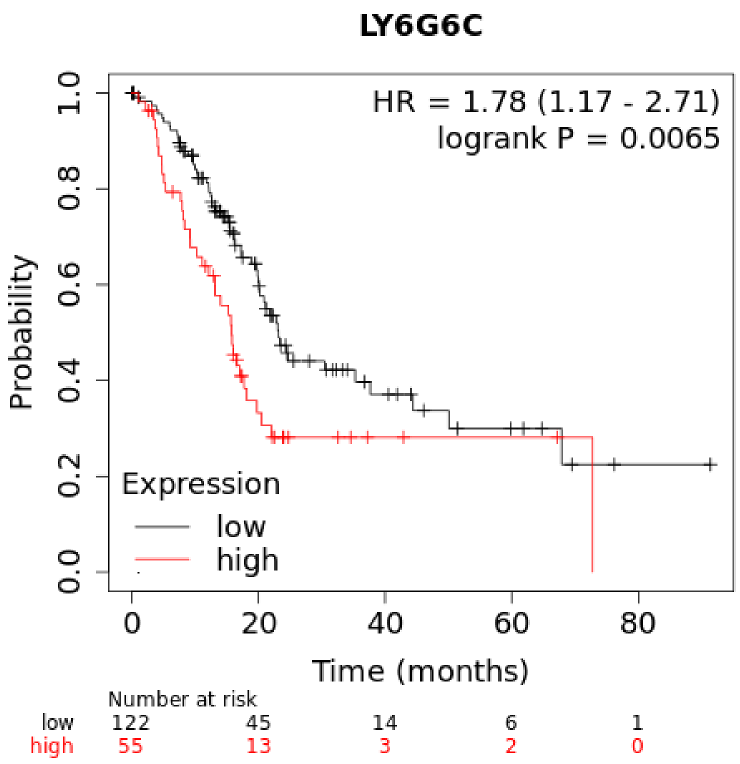

Click here to download the plot in TIFF format

Download plot as a PDF

Download p values vs. cutoff table

Median survival

Low expression cohort (months)	High expression cohort (months)
23.17	15.77

**RNAseq ID:**	LY6G6D -
**Survival:**	OS
**Auto select best cutoff:**	checked
**Follow up threshold:**	all
**Censore at threshold:**	checked
**Compute median over entire database:**	false
**Cutoff value used in analysis:**	0
**Expression range of the probe:**	0 - 2
**Invert HR values below 1:**	not checked

Restrictions

Tumor type: Pancreatic ductal adenocarcinoma

Restrict analysis to subtypes...

Stage:	all
Gender:	all
Race:	all
Grade:	all
Mutation burden:	all

Restrict analysis based on cellular content...

Basophils:	all

B-cells:	all
CD4+ memory T-cells:	all
CD8+ T-cells:	all
Eosinophils:	all
Macrophages:	all
Mesenchymal stem cells:	all
Natural killer T-cells:	all
Regulatory T-cells:	all
Type 1 T-helper cells:	all
Type 2 T-helper cells:	all

Results

P value: 0.0062
FDR: 50%

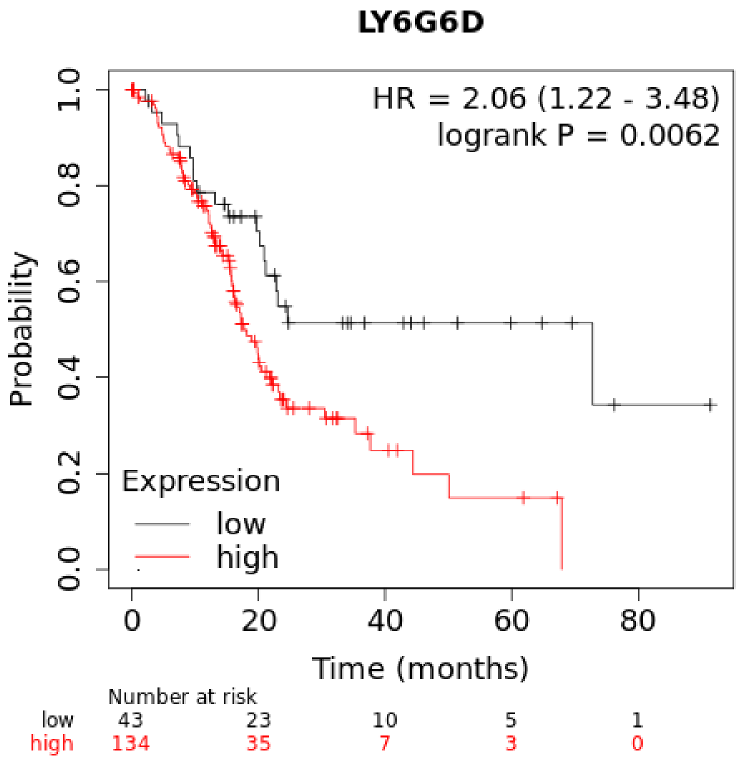

Click here to download the plot in TIFF format

Download plot as a PDF

Download p values vs. cutoff table

Median survival

Low expression cohort (months)	High expression cohort (months)
72.73	17.73

**RNAseq ID:**	LY6G6F -
**Survival:**	OS
**Auto select best cutoff:**	checked
**Follow up threshold:**	all
**Censore at threshold:**	checked
**Compute median over entire database:**	false
**Cutoff value used in analysis:**	0
**Expression range of the probe:**	0 - 5
**Invert HR values below 1:**	not checked

Restrictions

Tumor type: Pancreatic ductal adenocarcinoma

Restrict analysis to subtypes...

Stage:	all
Gender:	all
Race:	all
Grade:	all
Mutation burden:	all

Restrict analysis based on cellular content...

Basophils:	all
B-cells:	all
CD4+ memory T-cells:	all
CD8+ T-cells:	all
Eosinophils:	all
Macrophages:	all
Mesenchymal stem cells:	all
Natural killer T-cells:	all
Regulatory T-cells:	all
Type 1 T-helper cells:	all
Type 2 T-helper cells:	all

Results

P value: 0.0008
FDR: 20%

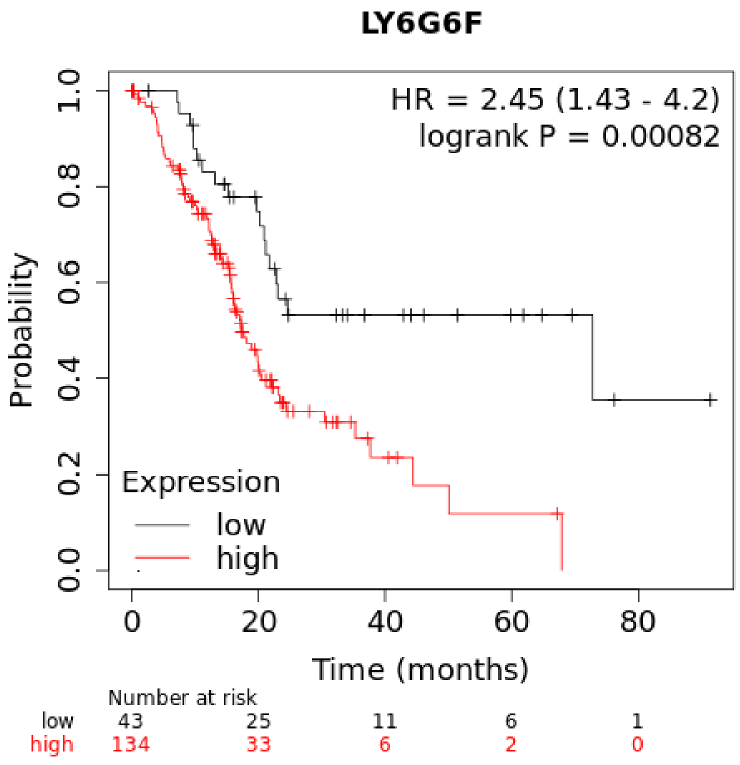

Click here to download the plot in TIFF format

Download plot as a PDF

Download p values vs. cutoff table

Median survival

Low expression cohort (months)	High expression cohort (months)
72.73	17.27

**RNAseq ID:**	LY6G5C -
**Survival:**	OS
**Auto select best cutoff:**	checked
**Follow up threshold:**	all
**Censore at threshold:**	checked
**Compute median over entire database:**	false
**Cutoff value used in analysis:**	49
**Expression range of the probe:**	15 - 586
**Invert HR values below 1:**	not checked

Restrictions

Tumor type: Pancreatic ductal adenocarcinoma

Restrict analysis to subtypes...

Stage:	all
Gender:	all
Race:	all
Grade:	all
Mutation burden:	all

Restrict analysis based on cellular content...

Basophils:	all
B-cells:	all
CD4+ memory T-cells:	all
CD8+ T-cells:	all
Eosinophils:	all
Macrophages:	all
Mesenchymal stem cells:	all
Natural killer T-cells:	all
Regulatory T-cells:	all
Type 1 T-helper cells:	all
Type 2 T-helper cells:	all

Results

**P value:**	2.0e-5
**FDR:**	1%

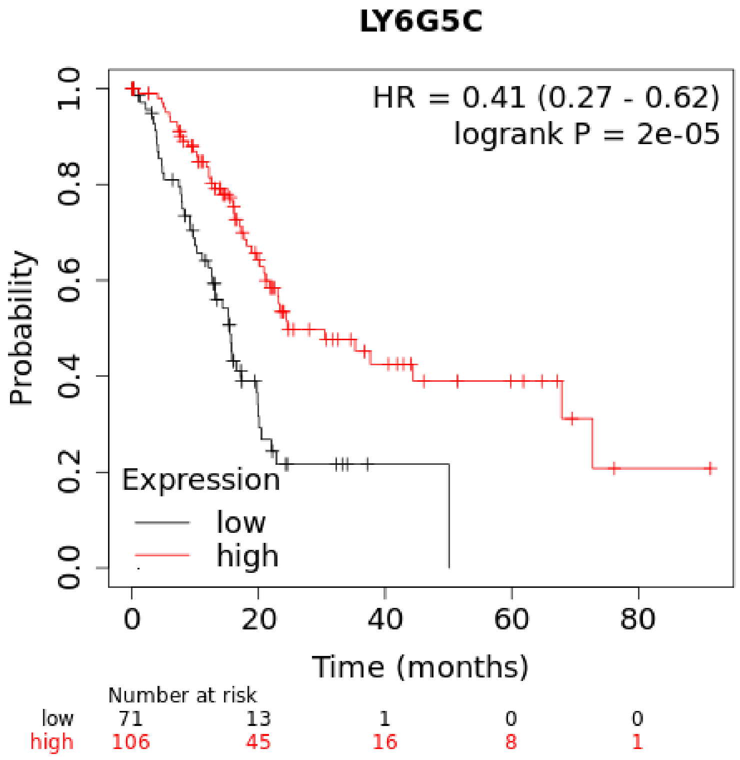

Click here to download the plot in TIFF format

Download plot as a PDF

Download p values vs. cutoff table

Median survival

Low expression cohort (months)	High expression cohort (months)
15.53	24.6

**RNAseq ID:**	LY6G5B -
**Survival:**	OS
**Auto select best cutoff:**	checked
**Follow up threshold:**	all
**Censore at threshold:**	checked
**Compute median over entire database:**	false
**Cutoff value used in analysis:**	37
**Expression range of the probe:**	3 - 192
**Invert HR values below 1:**	not checked

Restrictions

Tumor type: Pancreatic ductal adenocarcinoma

Restrict analysis to subtypes...

Stage:	all
Gender:	all
Race:	all
Grade:	all
Mutation burden:	all

Restrict analysis based on cellular content...

Basophils:	all

B-cells:	all
CD4+ memory T-cells:	all
CD8+ T-cells:	all
Eosinophils:	all
Macrophages:	all
Mesenchymal stem cells:	all
Natural killer T-cells:	all
Regulatory T-cells:	all
Type 1 T-helper cells:	all
Type 2 T-helper cells:	all

Results

P value: 0.0002
FDR: 5%

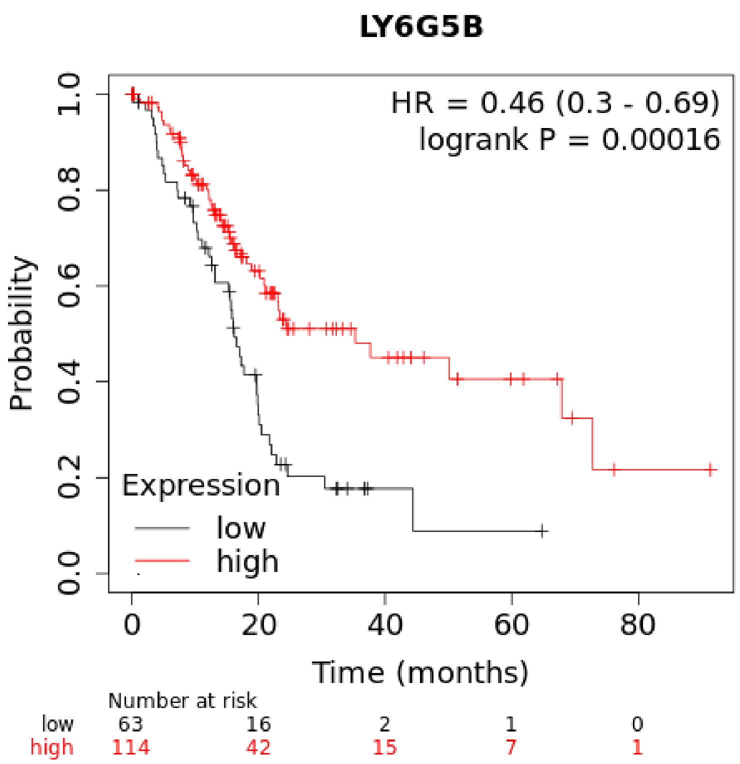

Click here to download the plot in TIFF format

Download plot as a PDF

Download p values vs. cutoff table

Median survival

Low expression cohort (months)	High expression cohort (months)
16.17	35.3

You can save the plots by right-clicking the image and then selecting "Save image as...". To generate a high resolution TIFF image, please adjust the "Settings" in the analysis page.

KMplot.com Copyright © 2009-2020 | Contact | Sitemap

Figure S2: KM plots and other raw data for the data depicted in Table 2

Pan-cancer

KM plotter | Home | Vote | Download | Updates | Contact

The desired RNAseq ID is valid: PSCA (-), LY6K (-), SLURP1 (-), LYPD2 (-), LY6D (-), GML (-), LY6E (-), LY6L (-), LY6H (-), GPIHBP1 (-), LYPD4 (-), CD177 (-), TEX101 (-), LYPD3 (-), PINLYP (-), PLAUR (-), LYPD5 (-), SPACA4 (-), ACRV1 (-), PATE1 (-), PATE2 (-), PATE3 (-), PATE4 (-), CD59 (-), LY6G6C (-), LY6G6D (-), LY6G6F (-), LY6G5C (-), LY6G5B (-),

**RNAseq ID:**	PSCA -
**Survival:**	OS
**Auto select best cutoff:**	checked
**Follow up threshold:**	all
**Censore at threshold:**	checked
**Compute median over entire database:**	false
**Cutoff value used in analysis:**	350
**Expression range of the probe:**	0 - 65661
**Invert HR values below 1:**	not checked

Restrictions

Tumor type: Pancreatic ductal adenocarcinoma

Restrict analysis to subtypes...

Stage:	all
Gender:	all
Race:	all
Grade:	all
Mutation burden:	all

Restrict analysis based on cellular content...

Basophils:	all
B-cells:	all
CD4+ memory T-cells:	all
CD8+ T-cells:	all
Eosinophils:	all
Macrophages:	all
Mesenchymal stem cells:	enriched
Natural killer T-cells:	all
Regulatory T-cells:	all
Type 1 T-helper cells:	all
Type 2 T-helper cells:	all

Results

P value: 5.4e-5
FDR: 1%

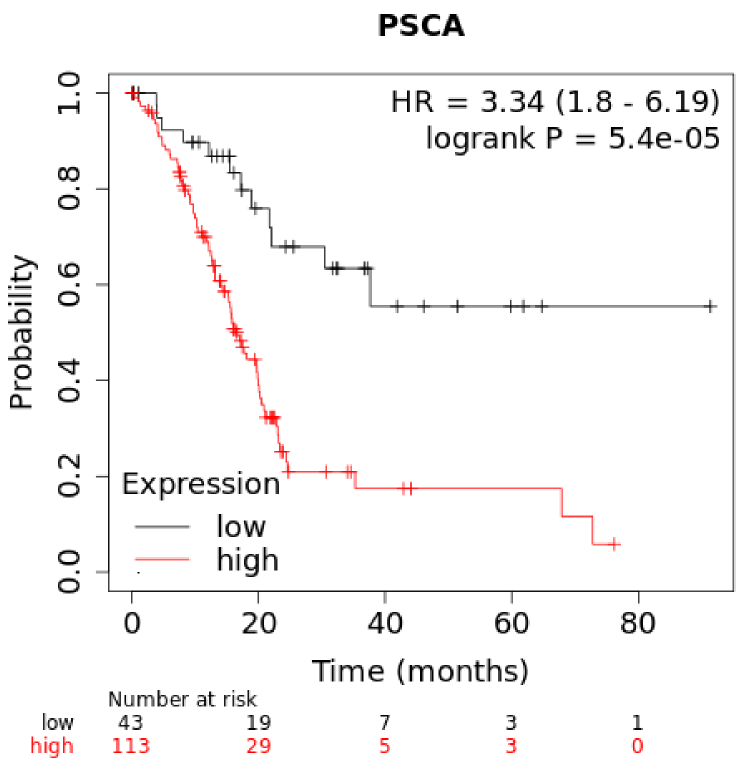

Click here to download the plot in TIFF format

Download plot as a PDF

Download p values vs. cutoff table

Upper quartile survival

Low expression cohort (months)	High expression cohort (months)
21.73	9.77

**RNAseq ID:**	LY6K -
**Survival:**	OS
**Auto select best cutoff:**	checked
**Follow up threshold:**	all
**Censore at threshold:**	checked
**Compute median over entire database:**	false
**Cutoff value used in analysis:**	10
**Expression range of the probe:**	0 - 1825
**Invert HR values below 1:**	not checked

Restrictions

Tumor type: Pancreatic ductal adenocarcinoma

Restrict analysis to subtypes...

Stage:	all
Gender:	all
Race:	all
Grade:	all
Mutation burden:	all

Restrict analysis based on cellular content...

Basophils:	all

B-cells:	all
CD4+ memory T-cells:	all
CD8+ T-cells:	all
Eosinophils:	all
Macrophages:	all
Mesenchymal stem cells:	enriched
Natural killer T-cells:	all
Regulatory T-cells:	all
Type 1 T-helper cells:	all
Type 2 T-helper cells:	all

Results

P value: 0.144
FDR: 100%

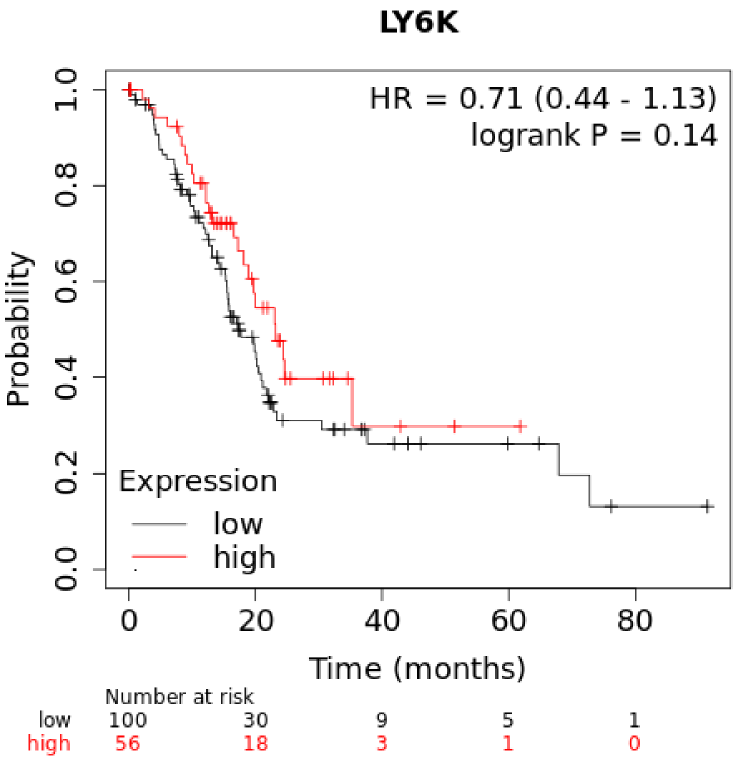

Click here to download the plot in TIFF format

Download plot as a PDF

Download p values vs. cutoff table

Median survival

Low expression cohort (months)	High expression cohort (months)
17.27	23.17

**RNAseq ID:**	SLURP1 -
**Survival:**	OS
**Auto select best cutoff:**	checked
**Follow up threshold:**	all
**Censore at threshold:**	checked
**Compute median over entire database:**	false
**Cutoff value used in analysis:**	1
**Expression range of the probe:**	0 - 185
**Invert HR values below 1:**	not checked

Restrictions

Tumor type: Pancreatic ductal adenocarcinoma

Restrict analysis to subtypes...

Stage:	all
Gender:	all
Race:	all
Grade:	all
Mutation burden:	all

Restrict analysis based on cellular content...

Basophils:	all
B-cells:	all
CD4+ memory T-cells:	all
CD8+ T-cells:	all
Eosinophils:	all
Macrophages:	all
Mesenchymal stem cells:	enriched
Natural killer T-cells:	all
Regulatory T-cells:	all
Type 1 T-helper cells:	all
Type 2 T-helper cells:	all

Results

P value: 2.2e-5
FDR: 1%

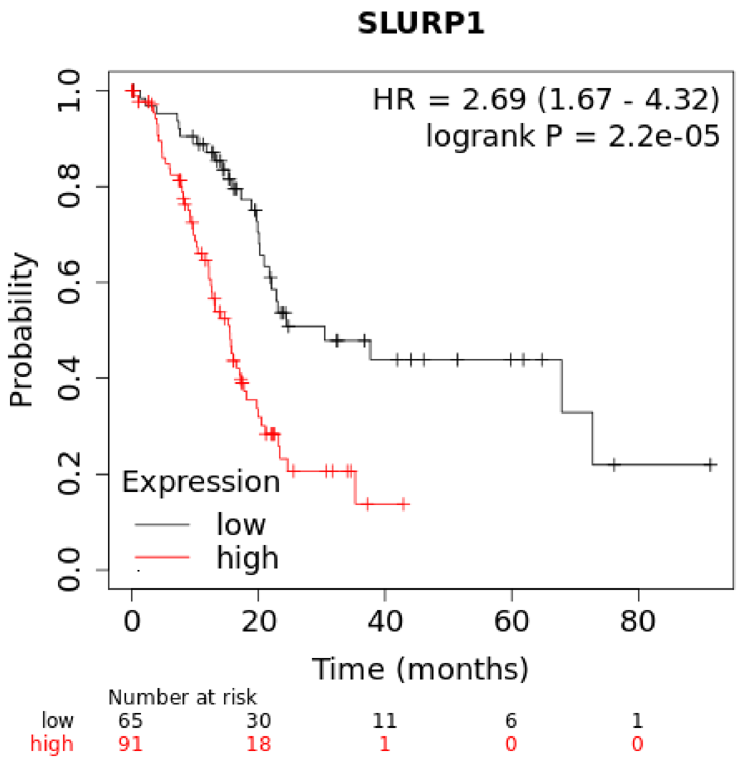

Click here to download the plot in TIFF format

Download plot as a PDF

Download p values vs. cutoff table

Median survival

Low expression cohort (months)	High expression cohort (months)
30.43	15.53

**RNAseq ID:**	LYPD2 -
**Survival:**	OS
**Auto select best cutoff:**	checked
**Follow up threshold:**	all
**Censore at threshold:**	checked
**Compute median over entire database:**	false
**Cutoff value used in analysis:**	4
**Expression range of the probe:**	0 - 4748
**Invert HR values below 1:**	not checked

Restrictions

Tumor type: Pancreatic ductal adenocarcinoma

Restrict analysis to subtypes...

Stage:	all
Gender:	all
Race:	all
Grade:	all
Mutation burden:	all

Restrict analysis based on cellular content...

Basophils:	all
B-cells:	all
CD4+ memory T-cells:	all
CD8+ T-cells:	all
Eosinophils:	all
Macrophages:	all
Mesenchymal stem cells:	enriched
Natural killer T-cells:	all
Regulatory T-cells:	all
Type 1 T-helper cells:	all
Type 2 T-helper cells:	all

Results

**P value:**	0.0052
**FDR:**	over 50%

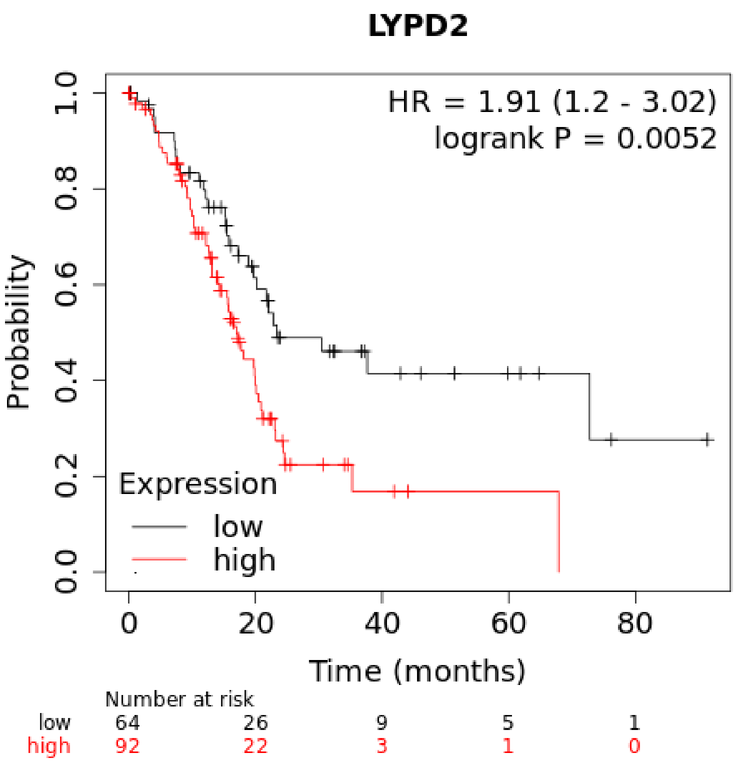

Click here to download the plot in TIFF format

Download plot as a PDF

Download p values vs. cutoff table

Median survival

Low expression cohort (months)	High expression cohort (months)
23.4	17.03

RNAseq ID: LY6D -
Survival: OS
Auto select best cutoff: checked
Follow up threshold: all
Censore at threshold: checked
Compute median over entire database: false
Cutoff value used in analysis: 160
Expression range of the probe: 0 - 16422
Invert HR values below 1: not checked

Restrictions

Tumor type: Pancreatic ductal adenocarcinoma

Restrict analysis to subtypes...

Stage: all
Gender: all
Race: all
Grade: all
Mutation burden: all

Restrict analysis based on cellular content...

Basophils: all

B-cells:	all
CD4+ memory T-cells:	all
CD8+ T-cells:	all
Eosinophils:	all
Macrophages:	all
Mesenchymal stem cells:	enriched
Natural killer T-cells:	all
Regulatory T-cells:	all
Type 1 T-helper cells:	all
Type 2 T-helper cells:	all

Results

P value: 2.0e-5
FDR: 1%

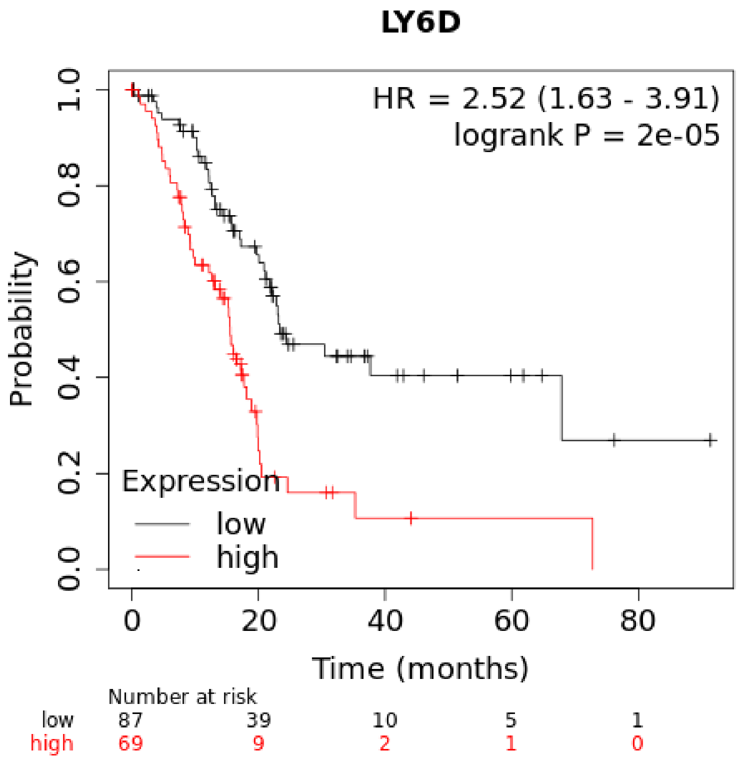

Click here to download the plot in TIFF format

Download plot as a PDF

Download p values vs. cutoff table

Median survival

Low expression cohort (months)	High expression cohort (months)
23.4	15.57

**RNAseq ID:**	GML -
**Survival:**	OS
**Auto select best cutoff:**	checked
**Follow up threshold:**	all
**Censore at threshold:**	checked
**Compute median over entire database:**	false
**Cutoff value used in analysis:**	0
**Expression range of the probe:**	0 - 2
**Invert HR values below 1:**	not checked

Restrictions

Tumor type: Pancreatic ductal adenocarcinoma

Restrict analysis to subtypes...

Stage:	all
Gender:	all
Race:	all
Grade:	all
Mutation burden:	all

Restrict analysis based on cellular content...

Basophils:	all
B-cells:	all
CD4+ memory T-cells:	all
CD8+ T-cells:	all
Eosinophils:	all
Macrophages:	all
Mesenchymal stem cells:	enriched
Natural killer T-cells:	all
Regulatory T-cells:	all
Type 1 T-helper cells:	all
Type 2 T-helper cells:	all

Results

P value: 0.0367
FDR: over 50%

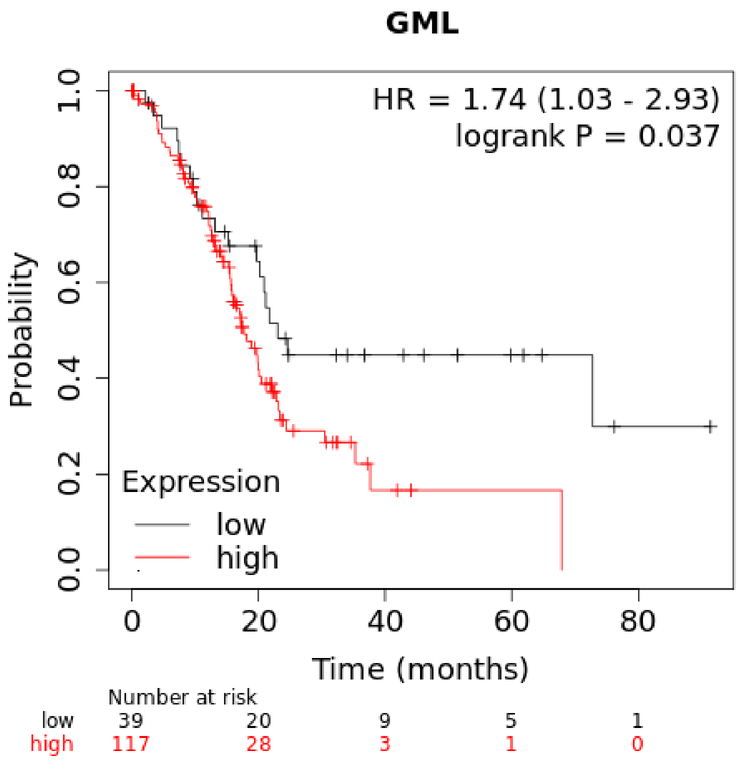

Click here to download the plot in TIFF format

Download plot as a PDF

Download p values vs. cutoff table

Median survival

Low expression cohort (months)	High expression cohort (months)
23.03	17.73

**RNAseq ID:**	LY6E	-
**Survival:**	OS	
**Auto select best cutoff:**	checked	
**Follow up threshold:**	all	
**Censore at threshold:**	checked	
**Compute median over entire database:**	false	
**Cutoff value used in analysis:**	10634	
**Expression range of the probe:**	508 - 56404	
**Invert HR values below 1:**	not checked	

Restrictions

Tumor type: Pancreatic ductal adenocarcinoma

Restrict analysis to subtypes...

Stage:	all
Gender:	all
Race:	all
Grade:	all
Mutation burden:	all

Restrict analysis based on cellular content...

Basophils:	all
B-cells:	all
CD4+ memory T-cells:	all
CD8+ T-cells:	all
Eosinophils:	all
Macrophages:	all
Mesenchymal stem cells:	enriched
Natural killer T-cells:	all
Regulatory T-cells:	all
Type 1 T-helper cells:	all
Type 2 T-helper cells:	all

Results

**P value:**	0.0029
**FDR:**	50%

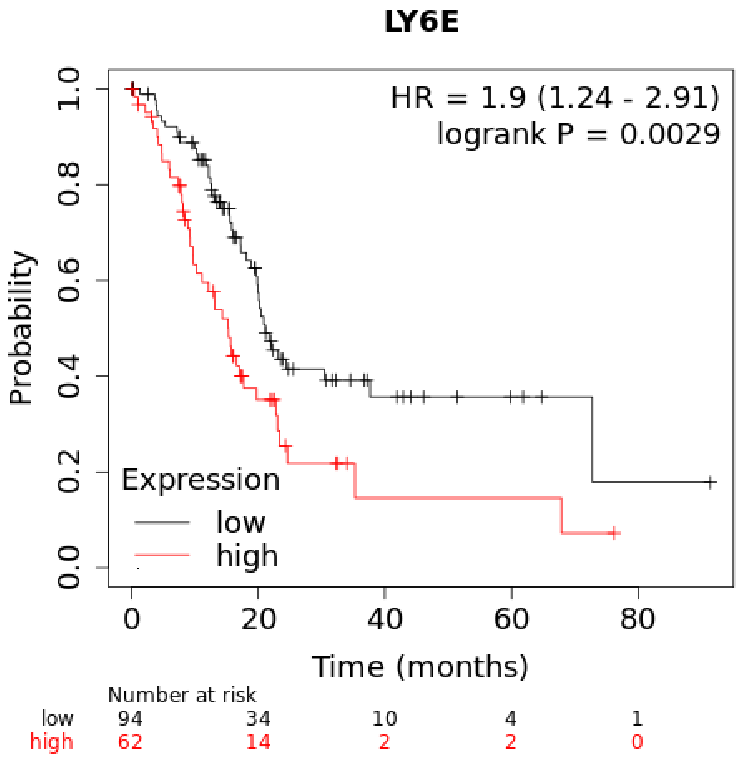

Click here to download the plot in TIFF format

Download plot as a PDF

Download p values vs. cutoff table

Median survival

Low expression cohort (months)	High expression cohort (months)
21.13	15.33

**RNAseq ID:**	LY6L -
**Survival:**	OS
**Auto select best cutoff:**	checked
**Follow up threshold:**	all
**Censore at threshold:**	checked
**Compute median over entire database:**	false
**Cutoff value used in analysis:**	0
**Expression range of the probe:**	0 - 6
**Invert HR values below 1:**	not checked

Restrictions

Tumor type: Pancreatic ductal adenocarcinoma

Restrict analysis to subtypes...

Stage:	all
Gender:	all
Race:	all
Grade:	all
Mutation burden:	all

Restrict analysis based on cellular content...

Basophils:	all

B-cells:	all
CD4+ memory T-cells:	all
CD8+ T-cells:	all
Eosinophils:	all
Macrophages:	all
Mesenchymal stem cells:	enriched
Natural killer T-cells:	all
Regulatory T-cells:	all
Type 1 T-helper cells:	all
Type 2 T-helper cells:	all

Results

P value: 0.0221
FDR: over 50%

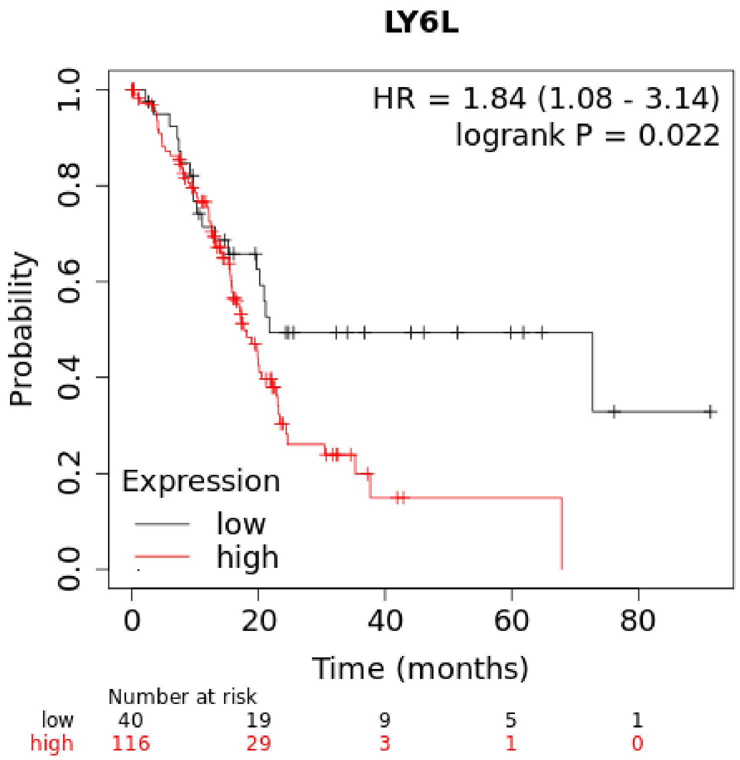

Click here to download the plot in TIFF format

Download plot as a PDF

Download p values vs. cutoff table

Median survival

Low expression cohort (months)	High expression cohort (months)
21.73	17.73

**RNAseq ID:**	LY6H -
**Survival:**	OS
**Auto select best cutoff:**	checked
**Follow up threshold:**	all
**Censore at threshold:**	checked
**Compute median over entire database:**	false
**Cutoff value used in analysis:**	21
**Expression range of the probe:**	1 - 9495
**Invert HR values below 1:**	not checked

Restrictions

Tumor type: Pancreatic ductal adenocarcinoma

Restrict analysis to subtypes...

Stage:	all
Gender:	all
Race:	all
Grade:	all
Mutation burden:	all

Restrict analysis based on cellular content...

Basophils:	all
B-cells:	all
CD4+ memory T-cells:	all
CD8+ T-cells:	all
Eosinophils:	all
Macrophages:	all
Mesenchymal stem cells:	enriched
Natural killer T-cells:	all
Regulatory T-cells:	all
Type 1 T-helper cells:	all
Type 2 T-helper cells:	all

Results

P value: 0.0067
FDR: 50%

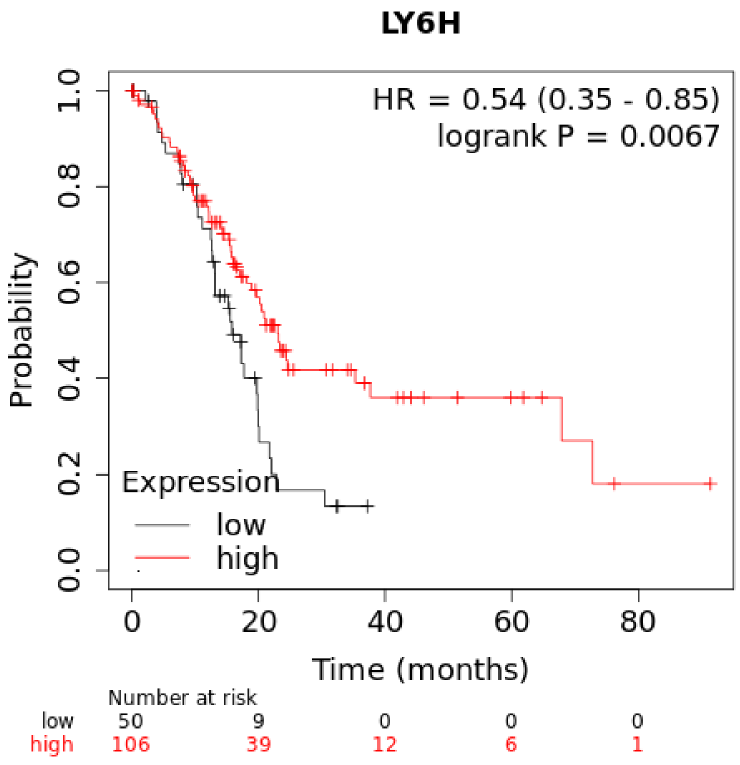

Click here to download the plot in TIFF format

Download plot as a PDF

Download p values vs. cutoff table

Median survival

Low expression cohort (months)	High expression cohort (months)
15.87	23.03

**RNAseq ID:**	GPIHBP1 -
**Survival:**	OS
**Auto select best cutoff:**	checked
**Follow up threshold:**	all
**Censore at threshold:**	checked
**Compute median over entire database:**	false
**Cutoff value used in analysis:**	32
**Expression range of the probe:**	5 - 273
**Invert HR values below 1:**	not checked

Restrictions

Tumor type: Pancreatic ductal adenocarcinoma

Restrict analysis to subtypes...

Stage:	all
Gender:	all
Race:	all
Grade:	all
Mutation burden:	all

Restrict analysis based on cellular content...

Basophils:	all
B-cells:	all
CD4+ memory T-cells:	all
CD8+ T-cells:	all
Eosinophils:	all
Macrophages:	all
Mesenchymal stem cells:	enriched
Natural killer T-cells:	all
Regulatory T-cells:	all
Type 1 T-helper cells:	all
Type 2 T-helper cells:	all

Results

**P value:**	0.3048
**FDR:**	100%

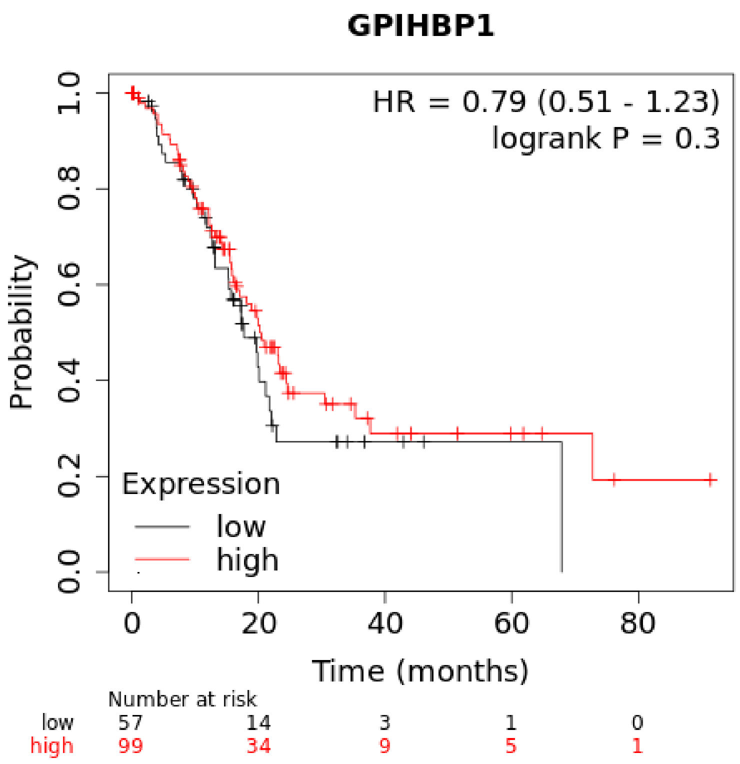

[Click here to download the plot in TIFF format]

[Download plot as a PDF]

[Download p values vs. cutoff table]

Median survival

Low expression cohort (months)	High expression cohort (months)
17.73	20.23

RNAseq ID: LYPD4 -
Survival: OS
Auto select best cutoff: checked
Follow up threshold: all
Censore at threshold: checked
Compute median over entire database: false
Cutoff value used in analysis: 0
Expression range of the probe: 0 - 18
Invert HR values below 1: not checked

Restrictions

Tumor type: Pancreatic ductal adenocarcinoma

Restrict analysis to subtypes...

Stage: all
Gender: all
Race: all
Grade: all
Mutation burden: all

Restrict analysis based on cellular content...

Basophils: all

B-cells: all
CD4+ memory T-cells: all
CD8+ T-cells: all
Eosinophils: all
Macrophages: all
Mesenchymal stem cells: enriched
Natural killer T-cells: all
Regulatory T-cells: all
Type 1 T-helper cells: all
Type 2 T-helper cells: all

Results

P value: 0.0212
FDR: over 50%

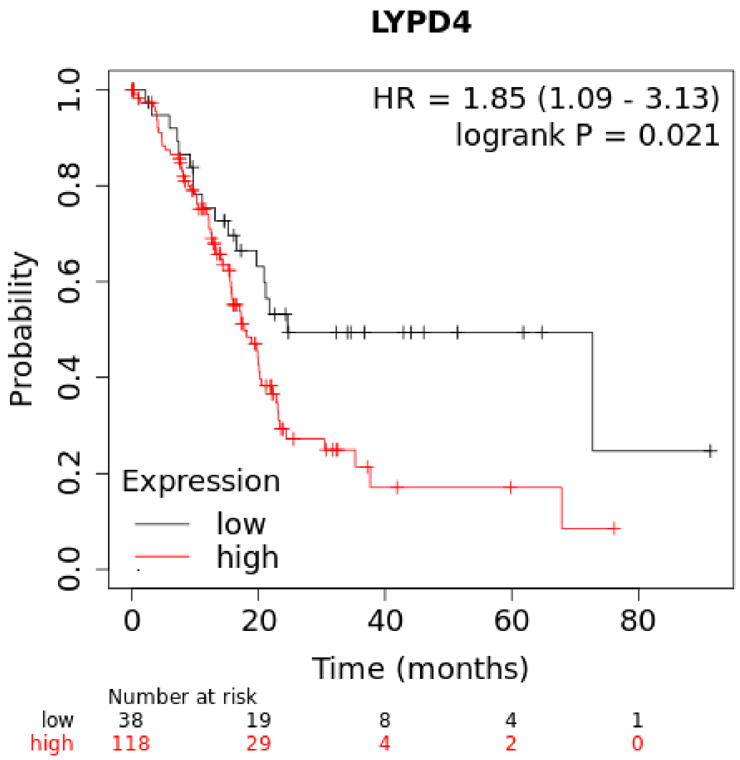

Click here to download the plot in TIFF format

Download plot as a PDF

Download p values vs. cutoff table

Median survival

Low expression cohort (months)	High expression cohort (months)
24.6	17.73

RNAseq ID: CD177 -
Survival: OS
Auto select best cutoff: checked
Follow up threshold: all
Censore at threshold: checked
Compute median over entire database: false
Cutoff value used in analysis: 18
Expression range of the probe: 0 - 6100
Invert HR values below 1: not checked

Restrictions

Tumor type: Pancreatic ductal adenocarcinoma

Restrict analysis to subtypes...

Stage: all
Gender: all
Race: all
Grade: all
Mutation burden: all

Restrict analysis based on cellular content...

Basophils: all
B-cells: all
CD4+ memory T-cells: all
CD8+ T-cells: all
Eosinophils: all
Macrophages: all
Mesenchymal stem cells: enriched
Natural killer T-cells: all
Regulatory T-cells: all
Type 1 T-helper cells: all
Type 2 T-helper cells: all

Results

P value: 0.1003
FDR: 100%

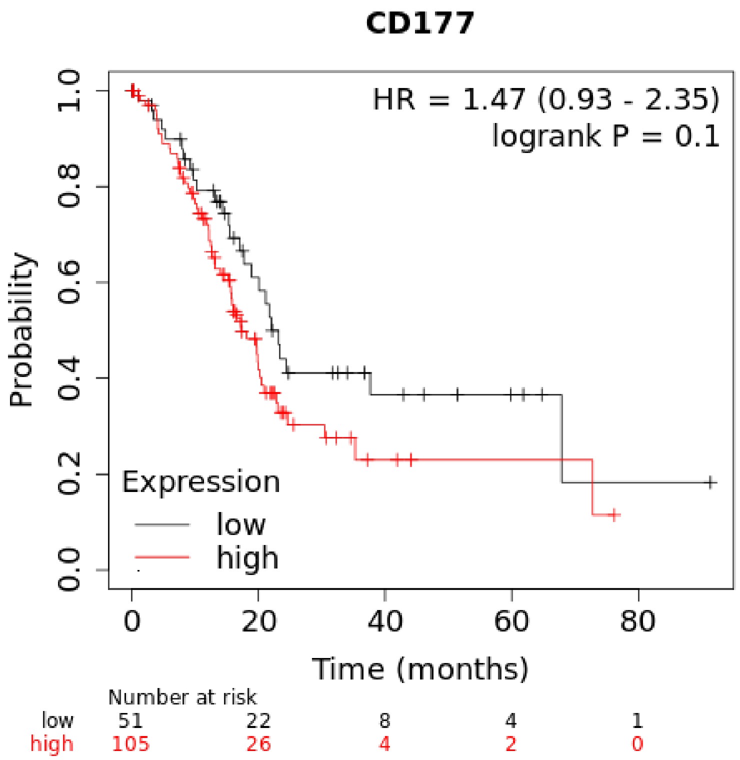

Click here to download the plot in TIFF format

Download plot as a PDF

Download p values vs. cutoff table

Median survival

Low expression cohort (months)	High expression cohort (months)
22.03	17.27

**RNAseq ID:**	TEX101 -
**Survival:**	OS
**Auto select best cutoff:**	checked
**Follow up threshold:**	all
**Censore at threshold:**	checked
**Compute median over entire database:**	false
**Cutoff value used in analysis:**	0
**Expression range of the probe:**	0 - 149
**Invert HR values below 1:**	not checked

Restrictions

Tumor type: Pancreatic ductal adenocarcinoma

Restrict analysis to subtypes...

Stage:	all
Gender:	all
Race:	all
Grade:	all
Mutation burden:	all

Restrict analysis based on cellular content...

Basophils:	all
B-cells:	all
CD4+ memory T-cells:	all
CD8+ T-cells:	all
Eosinophils:	all
Macrophages:	all
Mesenchymal stem cells:	enriched
Natural killer T-cells:	all
Regulatory T-cells:	all
Type 1 T-helper cells:	all
Type 2 T-helper cells:	all

Results

**P value:**	0.1153
**FDR:**	100%

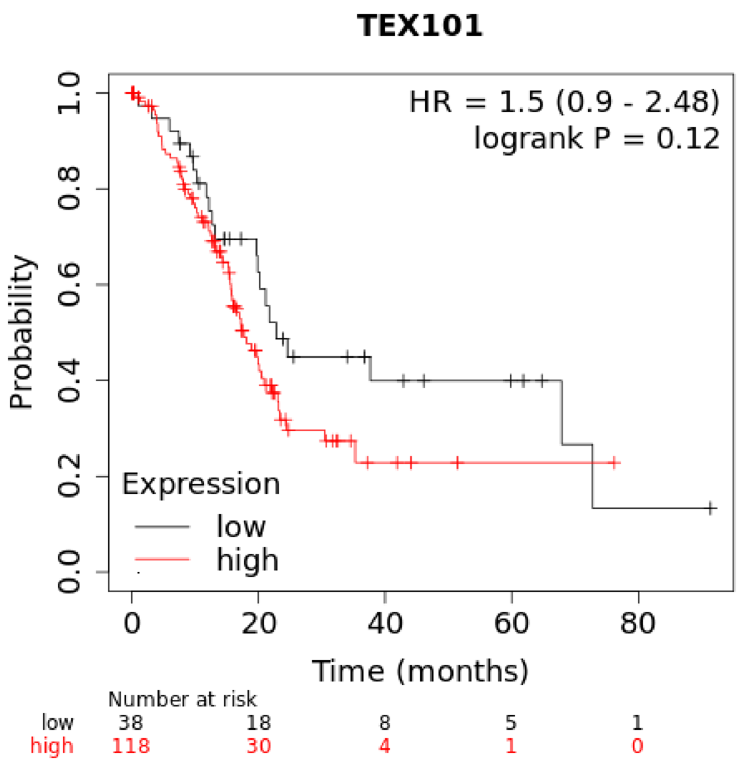

Click here to download the plot in TIFF format

Download plot as a PDF

Download p values vs. cutoff table

Median survival

Low expression cohort (months)	High expression cohort (months)
22.8	17.73

**RNAseq ID:**	LYPD3 -
**Survival:**	OS
**Auto select best cutoff:**	checked
**Follow up threshold:**	all
**Censore at threshold:**	checked
**Compute median over entire database:**	false
**Cutoff value used in analysis:**	144
**Expression range of the probe:**	9 - 7684
**Invert HR values below 1:**	not checked

Restrictions

Tumor type: Pancreatic ductal adenocarcinoma

Restrict analysis to subtypes...

Stage:	all
Gender:	all
Race:	all
Grade:	all
Mutation burden:	all

Restrict analysis based on cellular content...

Basophils:	all

B-cells:	all
CD4+ memory T-cells:	all
CD8+ T-cells:	all
Eosinophils:	all
Macrophages:	all
Mesenchymal stem cells:	enriched
Natural killer T-cells:	all
Regulatory T-cells:	all
Type 1 T-helper cells:	all
Type 2 T-helper cells:	all

Results

P value: 0.2587
FDR: 100%

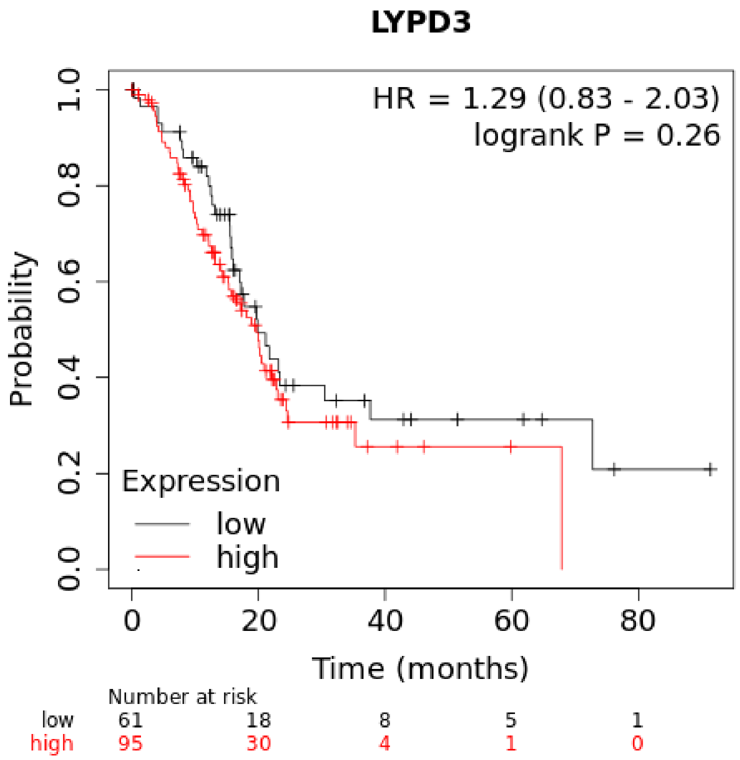

Click here to download the plot in TIFF format

Download plot as a PDF

Download p values vs. cutoff table

Median survival

Low expression cohort (months)	High expression cohort (months)
19.87	19.77

**RNAseq ID:**	PINLYP -
**Survival:**	OS
**Auto select best cutoff:**	checked
**Follow up threshold:**	all
**Censore at threshold:**	checked
**Compute median over entire database:**	false
**Cutoff value used in analysis:**	71
**Expression range of the probe:**	5 - 387
**Invert HR values below 1:**	not checked

Restrictions

Tumor type: Pancreatic ductal adenocarcinoma

Restrict analysis to subtypes...

Stage:	all
Gender:	all
Race:	all
Grade:	all
Mutation burden:	all

Restrict analysis based on cellular content...

Basophils:	all
B-cells:	all
CD4+ memory T-cells:	all
CD8+ T-cells:	all
Eosinophils:	all
Macrophages:	all
Mesenchymal stem cells:	enriched
Natural killer T-cells:	all
Regulatory T-cells:	all
Type 1 T-helper cells:	all
Type 2 T-helper cells:	all

Results

P value: 0.0068
FDR: over 50%

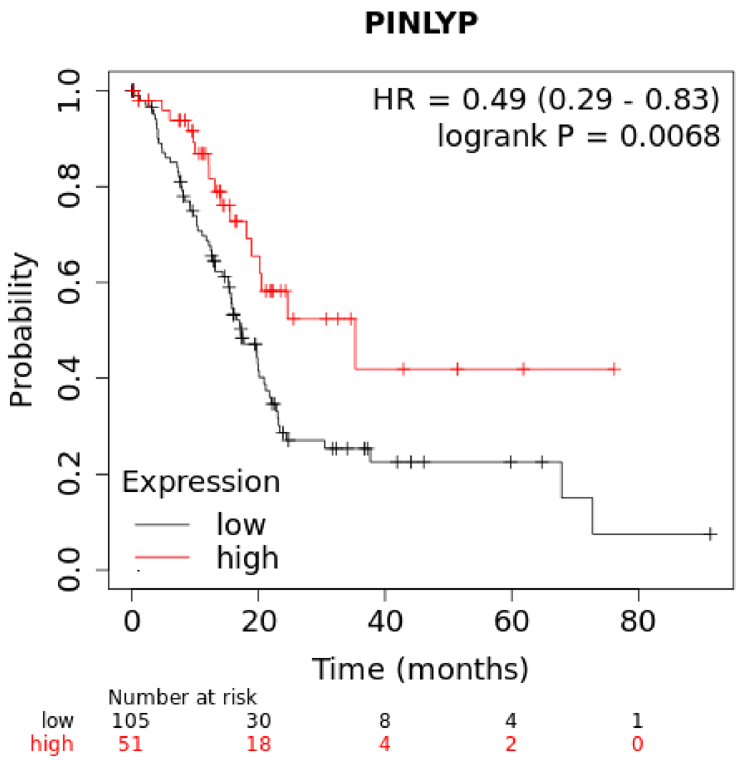

Click here to download the plot in TIFF format

Download plot as a PDF

Download p values vs. cutoff table

Median survival

Low expression cohort (months)	High expression cohort (months)
17.23	35.3

**RNAseq ID:**	PLAUR -
**Survival:**	OS
**Auto select best cutoff:**	checked
**Follow up threshold:**	all
**Censore at threshold:**	checked
**Compute median over entire database:**	false
**Cutoff value used in analysis:**	2095
**Expression range of the probe:**	55 - 18314
**Invert HR values below 1:**	not checked

Restrictions

Tumor type: Pancreatic ductal adenocarcinoma

Restrict analysis to subtypes...

Stage:	all
Gender:	all
Race:	all
Grade:	all
Mutation burden:	all

Restrict analysis based on cellular content...

Basophils:	all
B-cells:	all
CD4+ memory T-cells:	all
CD8+ T-cells:	all
Eosinophils:	all
Macrophages:	all
Mesenchymal stem cells:	enriched
Natural killer T-cells:	all
Regulatory T-cells:	all
Type 1 T-helper cells:	all
Type 2 T-helper cells:	all

Results

P value: 0.0594
FDR: 100%

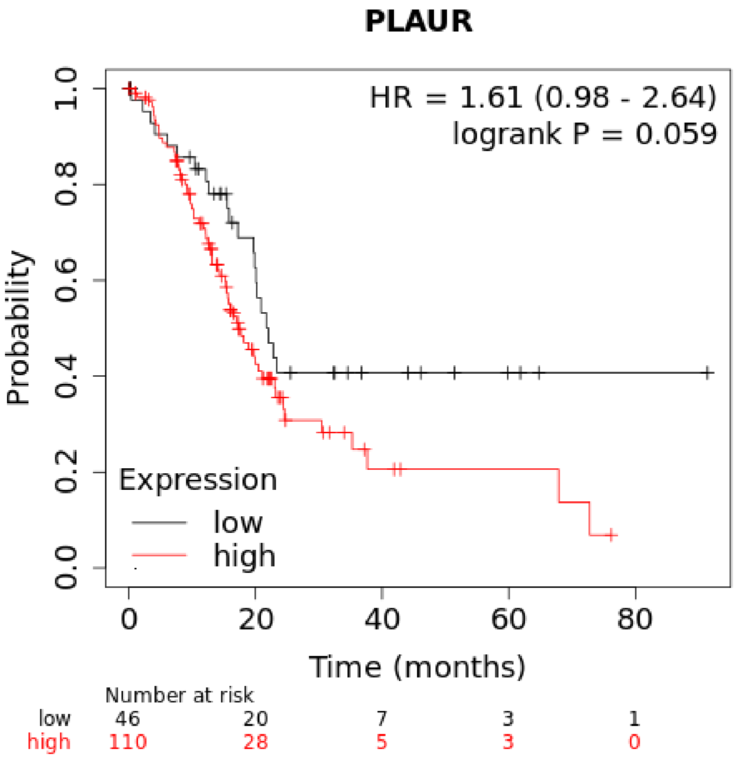

Click here to download the plot in TIFF format

Download plot as a PDF

Download p values vs. cutoff table

Median survival

Low expression cohort (months)	High expression cohort (months)
22.03	17.27

**RNAseq ID:**	LYPD5 -
**Survival:**	OS
**Auto select best cutoff:**	checked
**Follow up threshold:**	all
**Censore at threshold:**	checked
**Compute median over entire database:**	false
**Cutoff value used in analysis:**	78
**Expression range of the probe:**	5 - 578
**Invert HR values below 1:**	not checked

Restrictions

Tumor type: Pancreatic ductal adenocarcinoma

Restrict analysis to subtypes...

Stage:	all
Gender:	all
Race:	all
Grade:	all
Mutation burden:	all

Restrict analysis based on cellular content...

Basophils: all

B-cells:	all
CD4+ memory T-cells:	all
CD8+ T-cells:	all
Eosinophils:	all
Macrophages:	all
Mesenchymal stem cells:	enriched
Natural killer T-cells:	all
Regulatory T-cells:	all
Type 1 T-helper cells:	all
Type 2 T-helper cells:	all

Results

P value: 0.0048
FDR: 50%

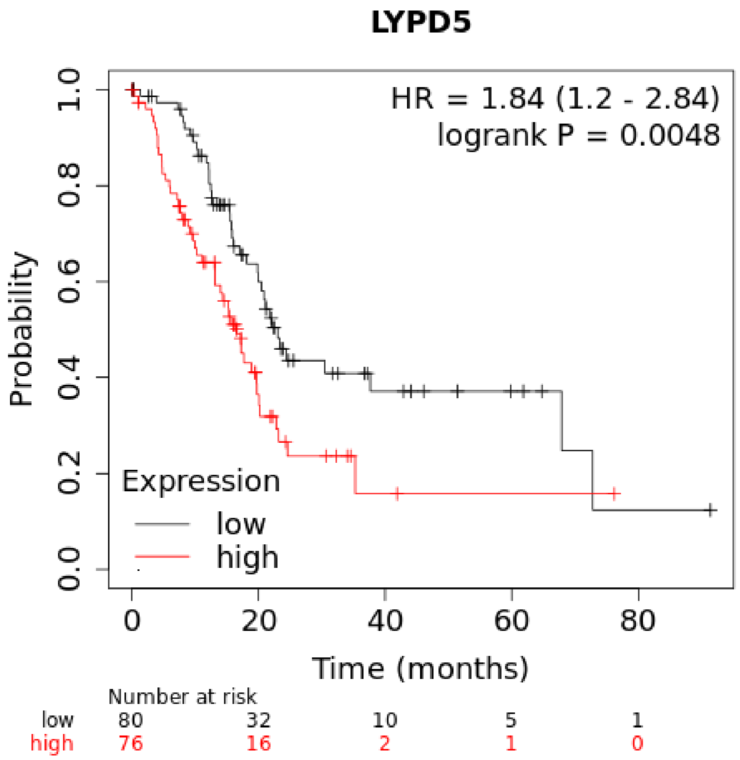

[Click here to download the plot in TIFF format]

[Download plot as a PDF]

[Download p values vs. cutoff table]

Median survival

Low expression cohort (months)	High expression cohort (months)
23.03	16.6

**RNAseq ID:**	SPACA4 -
**Survival:**	OS
**Auto select best cutoff:**	checked
**Follow up threshold:**	all
**Censore at threshold:**	checked
**Compute median over entire database:**	false
**Cutoff value used in analysis:**	19
**Expression range of the probe:**	0 - 206
**Invert HR values below 1:**	not checked

Restrictions

Tumor type: Pancreatic ductal adenocarcinoma

Restrict analysis to subtypes...

Stage:	all
Gender:	all
Race:	all
Grade:	all
Mutation burden:	all

Restrict analysis based on cellular content...

Basophils:	all
B-cells:	all
CD4+ memory T-cells:	all
CD8+ T-cells:	all
Eosinophils:	all
Macrophages:	all
Mesenchymal stem cells:	enriched
Natural killer T-cells:	all
Regulatory T-cells:	all
Type 1 T-helper cells:	all
Type 2 T-helper cells:	all

Results

P value: 0.1628
FDR: 100%

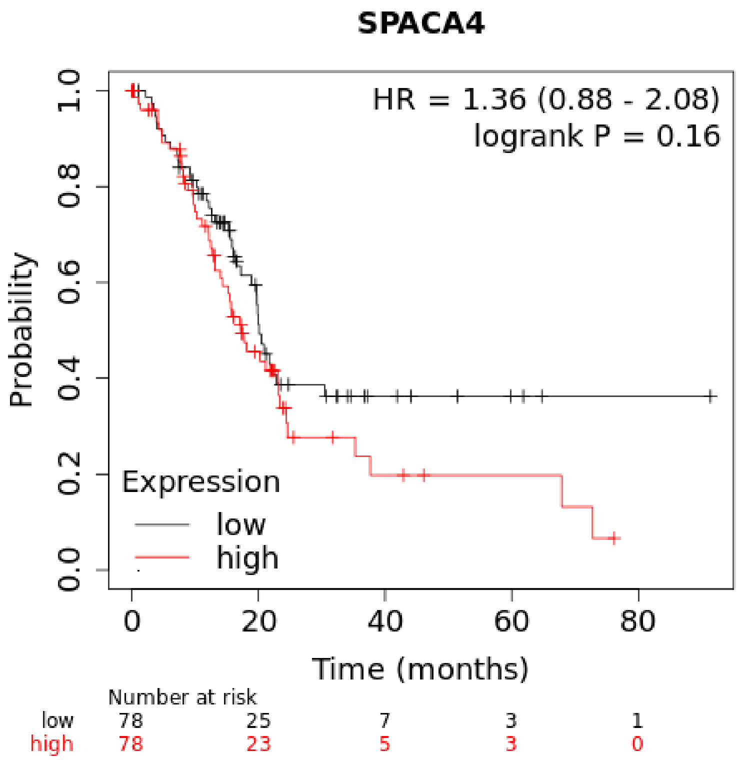

Click here to download the plot in TIFF format

Download plot as a PDF

Download p values vs. cutoff table

Median survival

Low expression cohort (months)	High expression cohort (months)
20.1	17.27

**RNAseq ID:**	ACRV1 -
**Survival:**	OS
**Auto select best cutoff:**	checked
**Follow up threshold:**	all
**Censore at threshold:**	checked
**Compute median over entire database:**	false
**Cutoff value used in analysis:**	6
**Expression range of the probe:**	0 - 71
**Invert HR values below 1:**	not checked

Restrictions

Tumor type: Pancreatic ductal adenocarcinoma

Restrict analysis to subtypes...

Stage:	all
Gender:	all
Race:	all
Grade:	all
Mutation burden:	all

Restrict analysis based on cellular content...

Basophils:	all
B-cells:	all
CD4+ memory T-cells:	all
CD8+ T-cells:	all
Eosinophils:	all
Macrophages:	all
Mesenchymal stem cells:	enriched
Natural killer T-cells:	all
Regulatory T-cells:	all
Type 1 T-helper cells:	all
Type 2 T-helper cells:	all

Results

P value: 0.1034
FDR: 100%

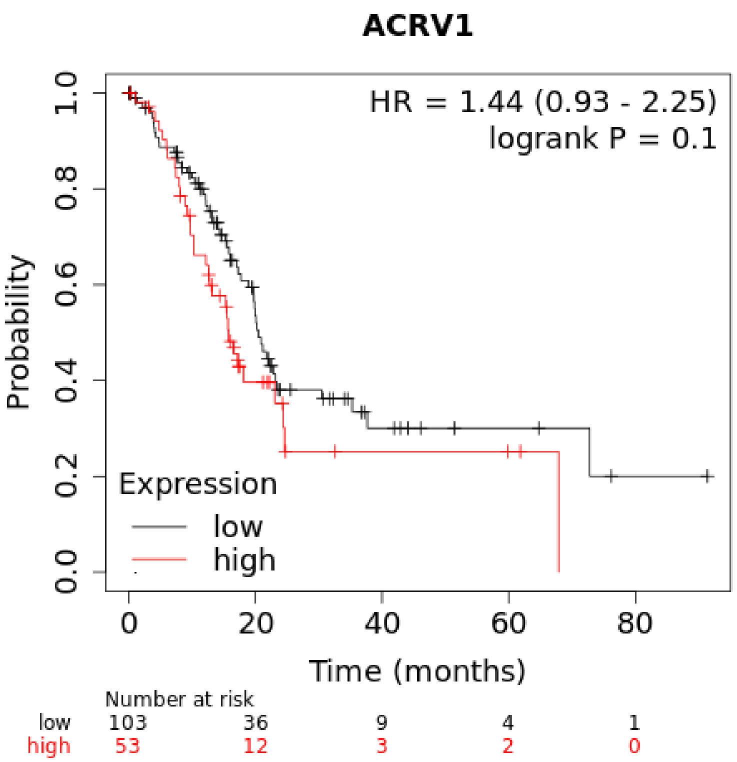

Click here to download the plot in TIFF format

Download plot as a PDF

Download p values vs. cutoff table

Median survival

Low expression cohort (months)	High expression cohort (months)
20.47	15.77

RNAseq ID: PATE1 -
Survival: OS
Auto select best cutoff: checked
Follow up threshold: all
Censore at threshold: checked
Compute median over entire database: false
Cutoff value used in analysis: 0
Expression range of the probe: 0 - 1
Invert HR values below 1: not checked

Restrictions

Tumor type: Pancreatic ductal adenocarcinoma

Restrict analysis to subtypes...

Stage: all
Gender: all
Race: all
Grade: all
Mutation burden: all

Restrict analysis based on cellular content...

Basophils: all

B-cells:	all
CD4+ memory T-cells:	all
CD8+ T-cells:	all
Eosinophils:	all
Macrophages:	all
Mesenchymal stem cells:	enriched
Natural killer T-cells:	all
Regulatory T-cells:	all
Type 1 T-helper cells:	all
Type 2 T-helper cells:	all

Results

P value: 0.0006
FDR: 20%

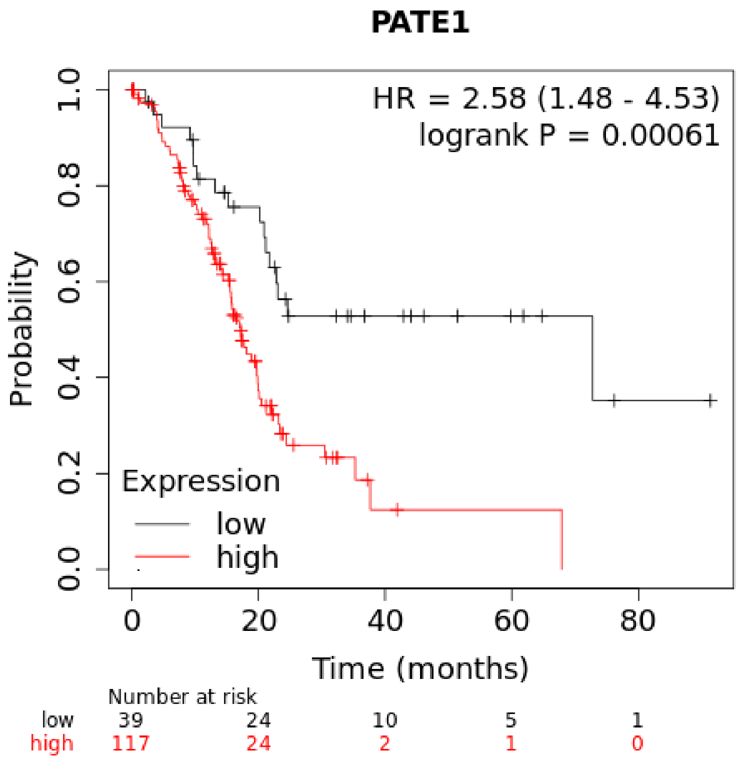

Click here to download the plot in TIFF format

Download plot as a PDF

Download p values vs. cutoff table

Median survival

Low expression cohort (months)	High expression cohort (months)
72.73	17.23

**RNAseq ID:**	PATE2 -
**Survival:**	OS
**Auto select best cutoff:**	checked
**Follow up threshold:**	all
**Censore at threshold:**	checked
**Compute median over entire database:**	false
**Cutoff value used in analysis:**	1
**Expression range of the probe:**	0 - 7
**Invert HR values below 1:**	not checked

Restrictions

Tumor type: Pancreatic ductal adenocarcinoma

Restrict analysis to subtypes...

Stage:	all
Gender:	all
Race:	all
Grade:	all
Mutation burden:	all

Restrict analysis based on cellular content...

Basophils:	all
B-cells:	all
CD4+ memory T-cells:	all
CD8+ T-cells:	all
Eosinophils:	all
Macrophages:	all
Mesenchymal stem cells:	enriched
Natural killer T-cells:	all
Regulatory T-cells:	all
Type 1 T-helper cells:	all
Type 2 T-helper cells:	all

Results

P value: 0.0013
FDR: 50%

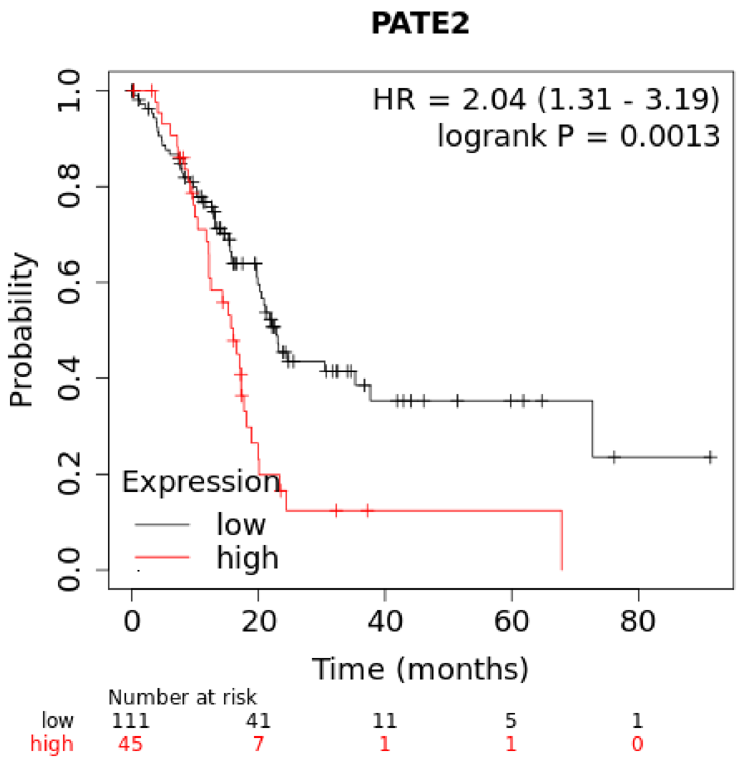

Click here to download the plot in TIFF format

Download plot as a PDF

Download p values vs. cutoff table

Median survival

Low expression cohort (months)	High expression cohort (months)
22.8	16.03

**RNAseq ID:**	PATE3 -
**Survival:**	OS
**Auto select best cutoff:**	checked
**Follow up threshold:**	all
**Censore at threshold:**	checked
**Compute median over entire database:**	false
**Cutoff value used in analysis:**	0
**Expression range of the probe:**	0 - 1
**Invert HR values below 1:**	not checked

Restrictions

Tumor type: Pancreatic ductal adenocarcinoma

Restrict analysis to subtypes...

Stage:	all
Gender:	all
Race:	all
Grade:	all
Mutation burden:	all

Restrict analysis based on cellular content...

Basophils:	all
B-cells:	all
CD4+ memory T-cells:	all
CD8+ T-cells:	all
Eosinophils:	all
Macrophages:	all
Mesenchymal stem cells:	enriched
Natural killer T-cells:	all
Regulatory T-cells:	all
Type 1 T-helper cells:	all
Type 2 T-helper cells:	all

Results

P value: 0.0064
FDR: over 50%

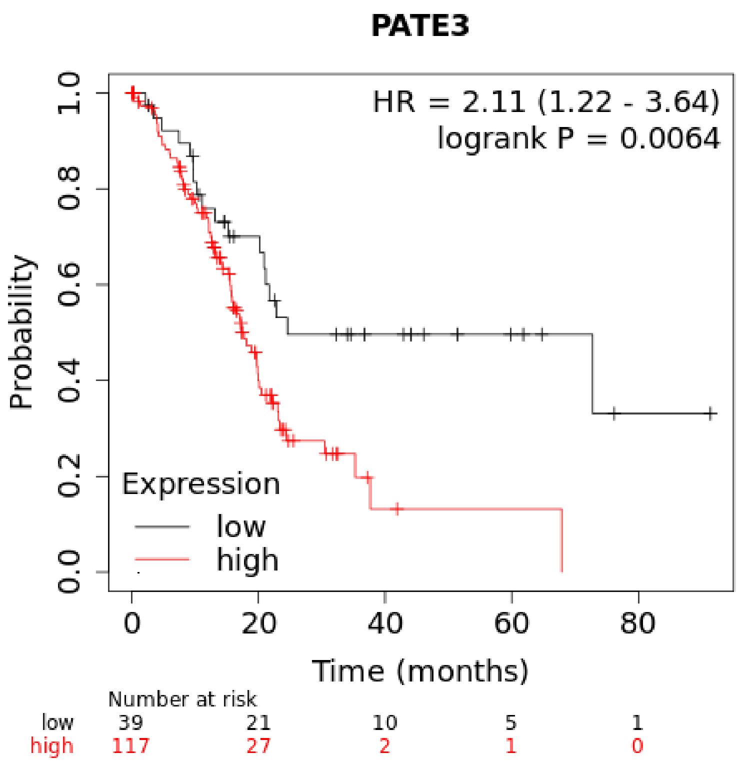

Click here to download the plot in TIFF format

Download plot as a PDF

Download p values vs. cutoff table

Median survival

Low expression cohort (months)	High expression cohort (months)
24.6	17.73

**RNAseq ID:**	PATE4 -
**Survival:**	OS
**Auto select best cutoff:**	checked
**Follow up threshold:**	all
**Censore at threshold:**	checked
**Compute median over entire database:**	false
**Cutoff value used in analysis:**	0
**Expression range of the probe:**	0 - 3
**Invert HR values below 1:**	not checked

Restrictions

Tumor type: Pancreatic ductal adenocarcinoma

Restrict analysis to subtypes...

Stage:	all
Gender:	all
Race:	all
Grade:	all
Mutation burden:	all

Restrict analysis based on cellular content...

Basophils:	all

B-cells:	all
CD4+ memory T-cells:	all
CD8+ T-cells:	all
Eosinophils:	all
Macrophages:	all
Mesenchymal stem cells:	enriched
Natural killer T-cells:	all
Regulatory T-cells:	all
Type 1 T-helper cells:	all
Type 2 T-helper cells:	all

Results

P value: 0.1682
FDR: 100%

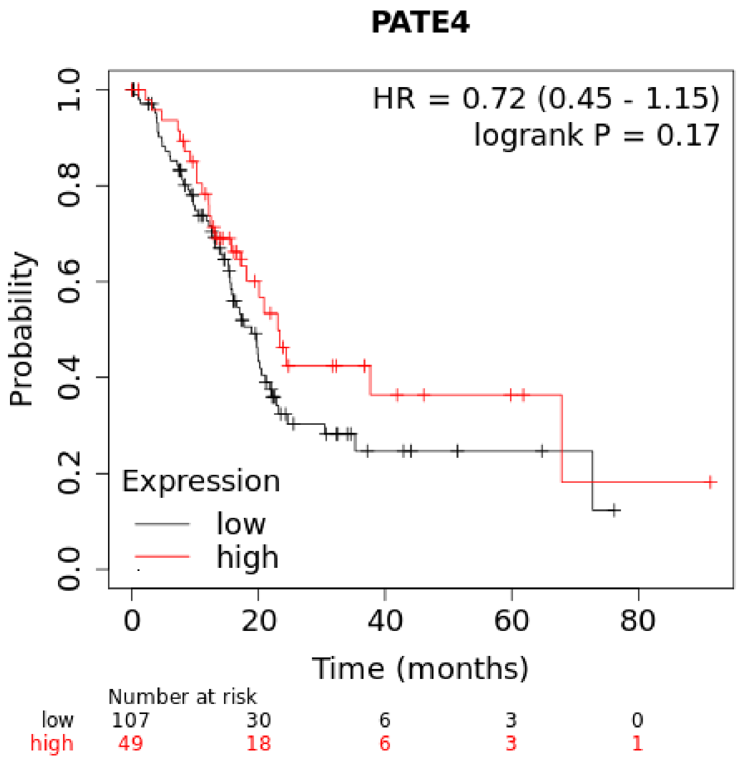

Click here to download the plot in TIFF format

Download plot as a PDF

Download p values vs. cutoff table

Median survival

Low expression cohort (months)	High expression cohort (months)
18.93	23.03

**RNAseq ID:**	CD59 -
**Survival:**	OS
**Auto select best cutoff:**	checked
**Follow up threshold:**	all
**Censore at threshold:**	checked
**Compute median over entire database:**	false
**Cutoff value used in analysis:**	17322
**Expression range of the probe:**	4436 - 39336
**Invert HR values below 1:**	not checked

Restrictions

Tumor type: Pancreatic ductal adenocarcinoma

Restrict analysis to subtypes...

Stage:	all
Gender:	all
Race:	all
Grade:	all
Mutation burden:	all

Restrict analysis based on cellular content...

Basophils:	all
B-cells:	all
CD4+ memory T-cells:	all
CD8+ T-cells:	all
Eosinophils:	all
Macrophages:	all
Mesenchymal stem cells:	enriched
Natural killer T-cells:	all
Regulatory T-cells:	all
Type 1 T-helper cells:	all
Type 2 T-helper cells:	all

Results

P value: 6.9e-5
FDR: 2%

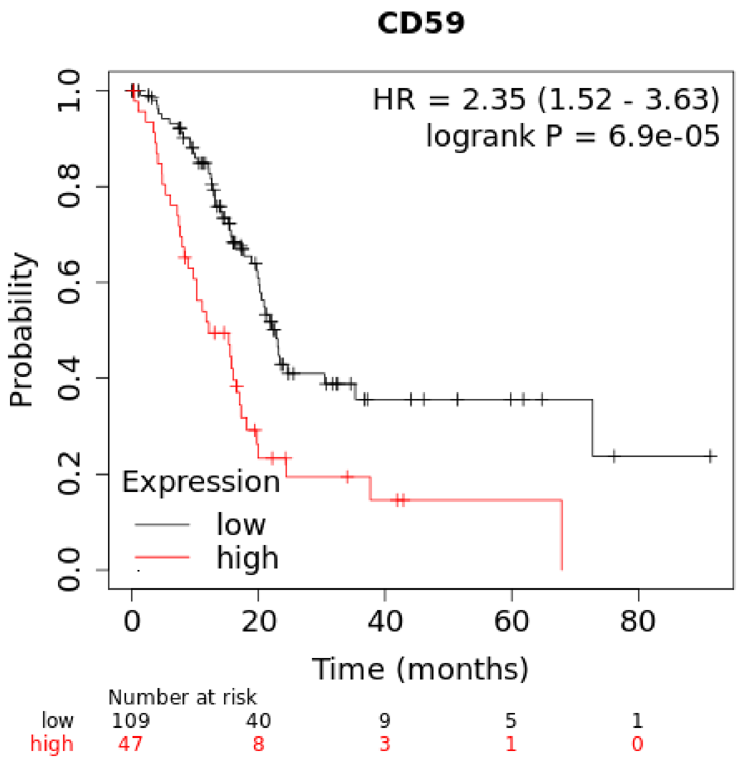

Click here to download the plot in TIFF format

Download plot as a PDF

Download p values vs. cutoff table

Median survival

Low expression cohort (months)	High expression cohort (months)
22.8	12.2

**RNAseq ID:**	LY6G6C -
**Survival:**	OS
**Auto select best cutoff:**	checked
**Follow up threshold:**	all
**Censore at threshold:**	checked
**Compute median over entire database:**	false
**Cutoff value used in analysis:**	8
**Expression range of the probe:**	0 - 251
**Invert HR values below 1:**	not checked

Restrictions

Tumor type: Pancreatic ductal adenocarcinoma

Restrict analysis to subtypes...

Stage:	all
Gender:	all
Race:	all
Grade:	all
Mutation burden:	all

Restrict analysis based on cellular content...

Basophils:	all
B-cells:	all
CD4+ memory T-cells:	all
CD8+ T-cells:	all
Eosinophils:	all
Macrophages:	all
Mesenchymal stem cells:	enriched
Natural killer T-cells:	all
Regulatory T-cells:	all
Type 1 T-helper cells:	all
Type 2 T-helper cells:	all

Results

**P value:**	0.0041
**FDR:**	20%

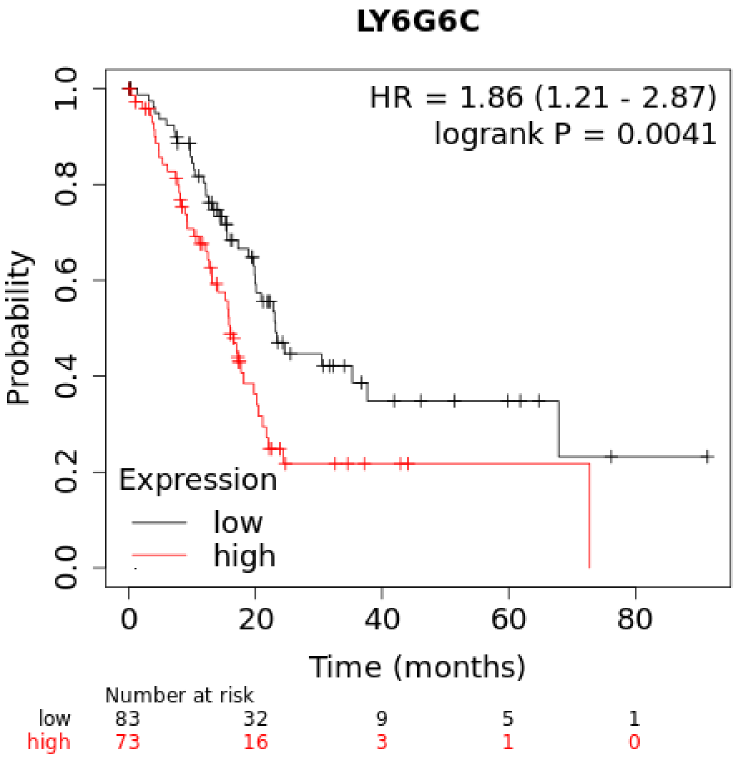

Click here to download the plot in TIFF format

Download plot as a PDF

Download p values vs. cutoff table

Median survival

Low expression cohort (months)	High expression cohort (months)
23.17	16.03

**RNAseq ID:**	LY6G6D -
**Survival:**	OS
**Auto select best cutoff:**	checked
**Follow up threshold:**	all
**Censore at threshold:**	checked
**Compute median over entire database:**	false
**Cutoff value used in analysis:**	0
**Expression range of the probe:**	0 - 2
**Invert HR values below 1:**	not checked

Restrictions

Tumor type: Pancreatic ductal adenocarcinoma

Restrict analysis to subtypes...

Stage:	all
Gender:	all
Race:	all
Grade:	all
Mutation burden:	all

Restrict analysis based on cellular content...

Basophils:	all

B-cells:	all
CD4+ memory T-cells:	all
CD8+ T-cells:	all
Eosinophils:	all
Macrophages:	all
Mesenchymal stem cells:	enriched
Natural killer T-cells:	all
Regulatory T-cells:	all
Type 1 T-helper cells:	all
Type 2 T-helper cells:	all

Results

P value: 0.0016
FDR: 10%

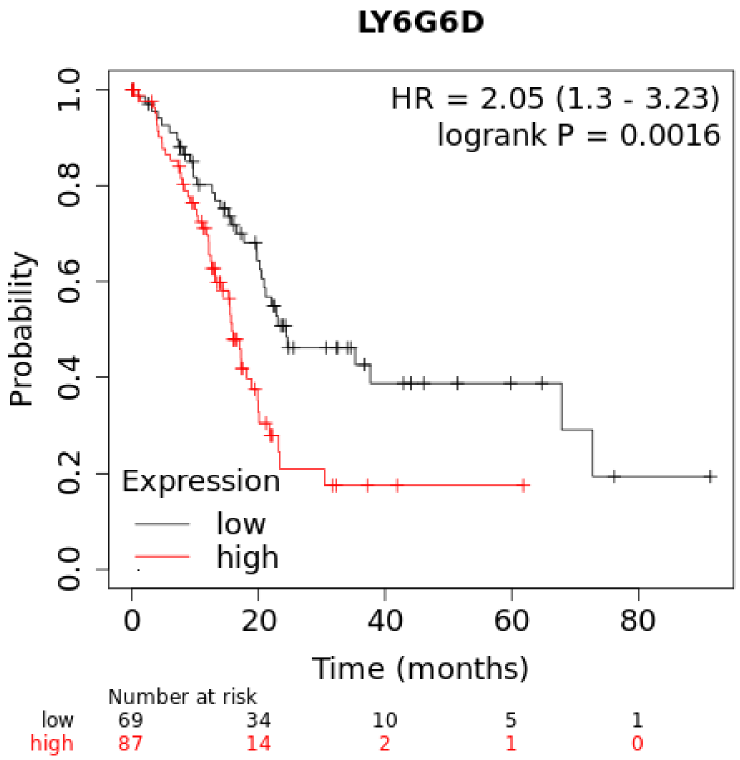

Click here to download the plot in TIFF format

Download plot as a PDF

Download p values vs. cutoff table

Median survival

Low expression cohort (months)	High expression cohort (months)
24.4	15.87

**RNAseq ID:**	LY6G6F -
**Survival:**	OS
**Auto select best cutoff:**	checked
**Follow up threshold:**	all
**Censore at threshold:**	checked
**Compute median over entire database:**	false
**Cutoff value used in analysis:**	0
**Expression range of the probe:**	0 - 5
**Invert HR values below 1:**	not checked

Restrictions

Tumor type: Pancreatic ductal adenocarcinoma

Restrict analysis to subtypes...

Stage:	all
Gender:	all
Race:	all
Grade:	all
Mutation burden:	all

Restrict analysis based on cellular content...

Basophils:	all
B-cells:	all
CD4+ memory T-cells:	all
CD8+ T-cells:	all
Eosinophils:	all
Macrophages:	all
Mesenchymal stem cells:	enriched
Natural killer T-cells:	all
Regulatory T-cells:	all
Type 1 T-helper cells:	all
Type 2 T-helper cells:	all

Results

P value: 0.0004
FDR: 10%

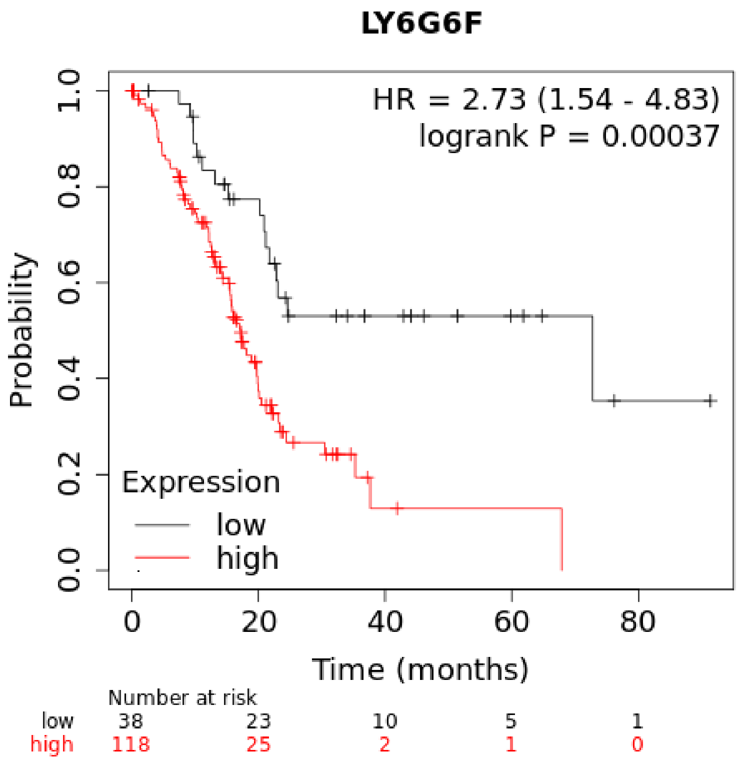

Click here to download the plot in TIFF format

Download plot as a PDF

Download p values vs. cutoff table

Median survival

Low expression cohort (months)	High expression cohort (months)
72.73	17.23

**RNAseq ID:**	LY6G5C -
**Survival:**	OS
**Auto select best cutoff:**	checked
**Follow up threshold:**	all
**Censore at threshold:**	checked
**Compute median over entire database:**	false
**Cutoff value used in analysis:**	49
**Expression range of the probe:**	15 - 253
**Invert HR values below 1:**	not checked

Restrictions

Tumor type: Pancreatic ductal adenocarcinoma

Restrict analysis to subtypes...

Stage:	all
Gender:	all
Race:	all
Grade:	all
Mutation burden:	all

Restrict analysis based on cellular content...

Basophils:	all
B-cells:	all
CD4+ memory T-cells:	all
CD8+ T-cells:	all
Eosinophils:	all
Macrophages:	all
Mesenchymal stem cells:	enriched
Natural killer T-cells:	all
Regulatory T-cells:	all
Type 1 T-helper cells:	all
Type 2 T-helper cells:	all

Results

P value: 5.4e-5
FDR: 2%

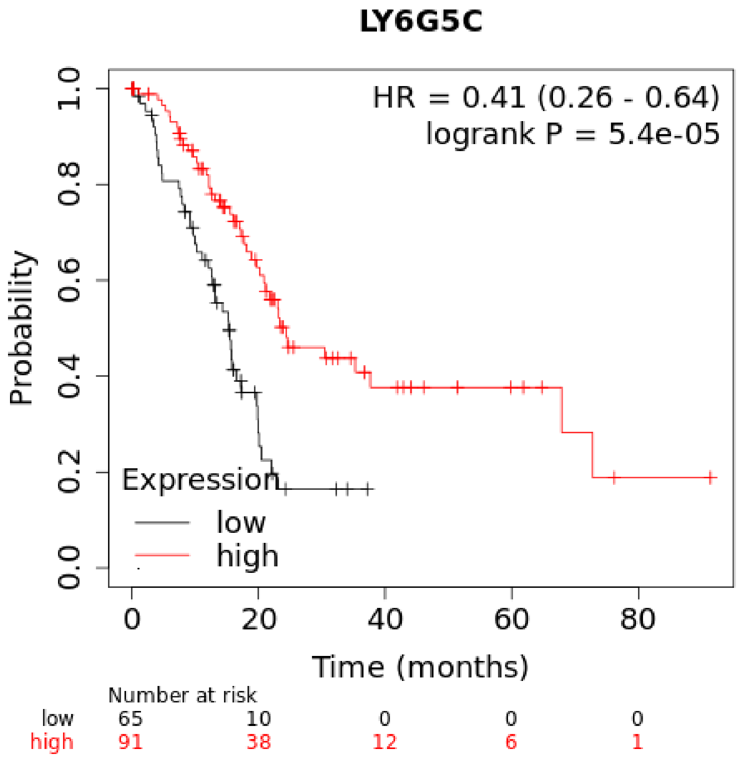

Click here to download the plot in TIFF format

Download plot as a PDF

Download p values vs. cutoff table

Median survival

Low expression cohort (months)	High expression cohort (months)
15.33	24.4

RNAseq ID: LY6G5B -
Survival: OS
Auto select best cutoff: checked
Follow up threshold: all
Censore at threshold: checked
Compute median over entire database: false
Cutoff value used in analysis: 37
Expression range of the probe: 3 - 192
Invert HR values below 1: not checked

Restrictions

Tumor type: Pancreatic ductal adenocarcinoma

Restrict analysis to subtypes...

Stage: all
Gender: all
Race: all
Grade: all
Mutation burden: all

Restrict analysis based on cellular content...

Basophils: all

B-cells:	all
CD4+ memory T-cells:	all
CD8+ T-cells:	all
Eosinophils:	all
Macrophages:	all
Mesenchymal stem cells:	enriched
Natural killer T-cells:	all
Regulatory T-cells:	all
Type 1 T-helper cells:	all
Type 2 T-helper cells:	all

Results

P value: 0.003
FDR: over 50%

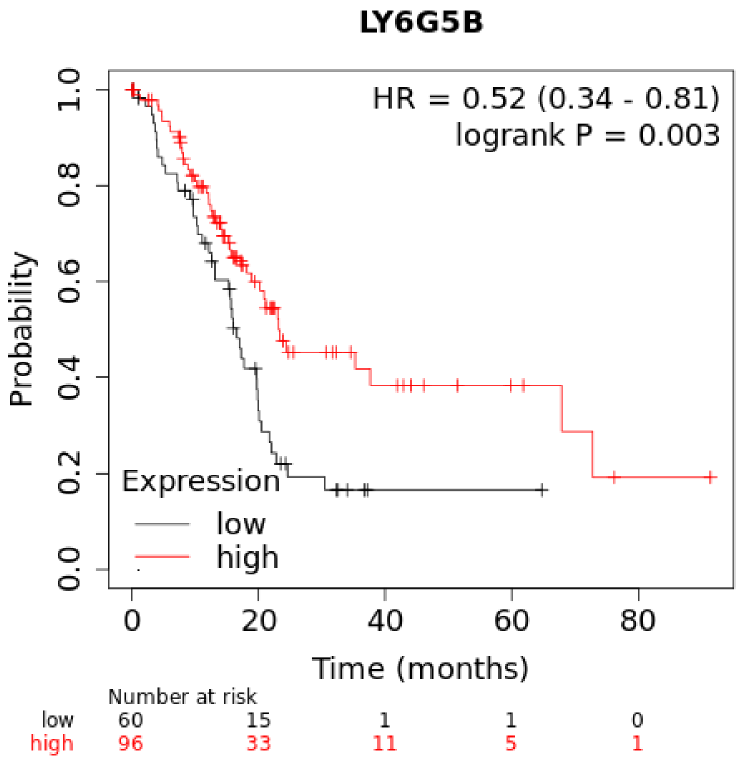

Click here to download the plot in TIFF format

Download plot as a PDF

Download p values vs. cutoff table

Median survival

Low expression cohort (months)	High expression cohort (months)
16.6	23.17

You can save the plots by right-clicking the image and then selecting "Save image as...". To generate a high resolution TIFF image, please adjust the "Settings" in the analysis page.

KMplot.com Copyright © 2009-2020 | Contact | Sitemap

Pan-cancer

KM plotter | Home | Vote | Download | Updates | Contact

The desired RNAseq ID is valid: PSCA (-), LY6K (-), SLURP1 (-), LYPD2 (-), LY6D (-), GML (-), LY6E (-), LY6L (-), LY6H (-), GPIHBP1 (-), LYPD4 (-), CD177 (-), TEX101 (-), LYPD3 (-), PINLYP (-), PLAUR (-), LYPD5 (-), SPACA4 (-), ACRV1 (-), PATE1 (-), PATE2 (-), PATE3 (-), PATE4 (-), CD59 (-), LY6G6C (-), LY6G6D (-), LY6G6F (-), LY6G5C (-), LY6G5B (-),

**RNAseq ID:**	PSCA -
**Survival:**	OS
**Auto select best cutoff:**	checked
**Follow up threshold:**	all
**Censore at threshold:**	checked
**Compute median over entire database:**	false
**Cutoff value used in analysis:**	2128
**Expression range of the probe:**	0 - 43084
**Invert HR values below 1:**	not checked

Restrictions

Tumor type: Pancreatic ductal adenocarcinoma

Restrict analysis to subtypes...

Stage:	all
Gender:	all
Race:	all
Grade:	all
Mutation burden:	all

Restrict analysis based on cellular content...

Basophils:	all
B-cells:	all
CD4+ memory T-cells:	all
CD8+ T-cells:	all
Eosinophils:	all
Macrophages:	all
Mesenchymal stem cells:	decreased
Natural killer T-cells:	all
Regulatory T-cells:	all
Type 1 T-helper cells:	all
Type 2 T-helper cells:	all

Results

P value: 0.326
FDR: 100%

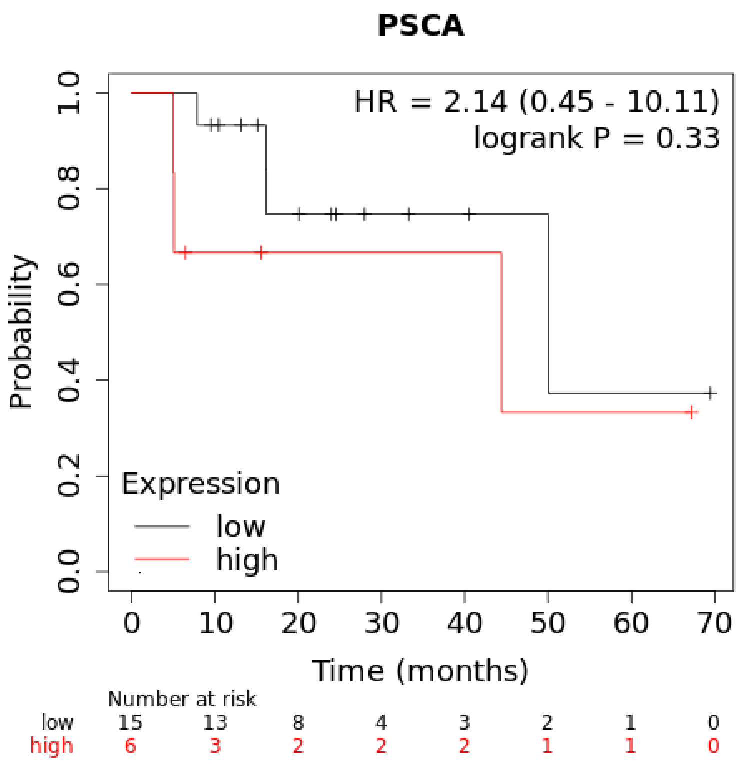

Click here to download the plot in TIFF format

Download plot as a PDF

Download p values vs. cutoff table

Median survival

Low expression cohort (months)	High expression cohort (months)
50.07	44.4

RNAseq ID: LY6K -
Survival: OS
Auto select best cutoff: checked
Follow up threshold: all
Censore at threshold: checked
Compute median over entire database: false
Cutoff value used in analysis: 6
Expression range of the probe: 1 - 248
Invert HR values below 1: not checked

Restrictions

Tumor type: Pancreatic ductal adenocarcinoma

Restrict analysis to subtypes...

Stage: all
Gender: all
Race: all
Grade: all
Mutation burden: all

Restrict analysis based on cellular content...

Basophils: all

B-cells:	all
CD4+ memory T-cells:	all
CD8+ T-cells:	all
Eosinophils:	all
Macrophages:	all
Mesenchymal stem cells:	decreased
Natural killer T-cells:	all
Regulatory T-cells:	all
Type 1 T-helper cells:	all
Type 2 T-helper cells:	all

Results

P value: 0.4699
FDR: 100%

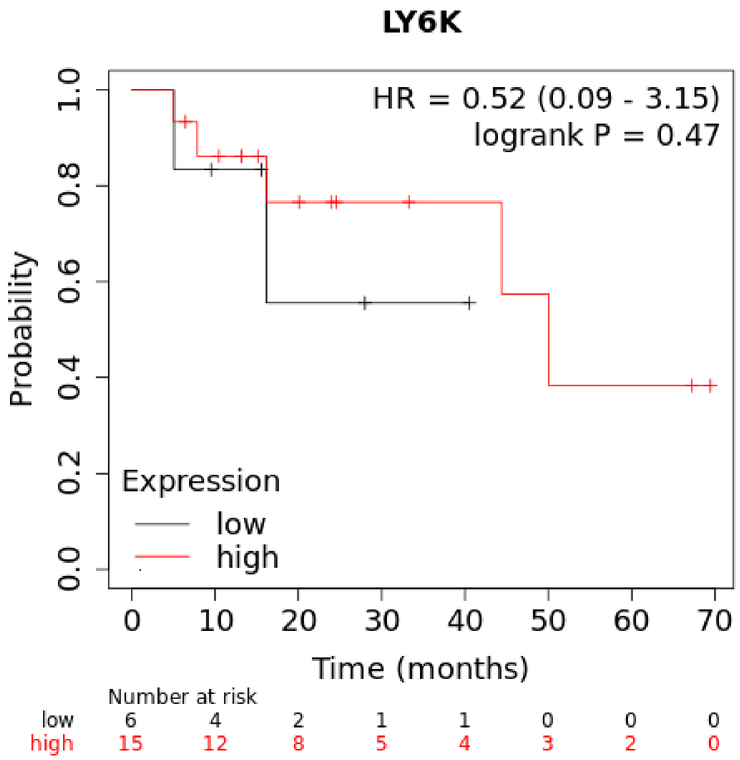

Click here to download the plot in TIFF format

Download plot as a PDF

Download p values vs. cutoff table

Upper quartile survival

Low expression cohort (months)	High expression cohort (months)
16.17	44.4

**RNAseq ID:**	SLURP1 -
**Survival:**	OS
**Auto select best cutoff:**	checked
**Follow up threshold:**	all
**Censore at threshold:**	checked
**Compute median over entire database:**	false
**Cutoff value used in analysis:**	0
**Expression range of the probe:**	0 - 279
**Invert HR values below 1:**	not checked

Restrictions

Tumor type: Pancreatic ductal adenocarcinoma

Restrict analysis to subtypes...

Stage:	all
Gender:	all
Race:	all
Grade:	all
Mutation burden:	all

Restrict analysis based on cellular content...

Basophils:	all
B-cells:	all
CD4+ memory T-cells:	all
CD8+ T-cells:	all
Eosinophils:	all
Macrophages:	all
Mesenchymal stem cells:	decreased
Natural killer T-cells:	all
Regulatory T-cells:	all
Type 1 T-helper cells:	all
Type 2 T-helper cells:	all

Results

P value: 0.1796
FDR: 100%

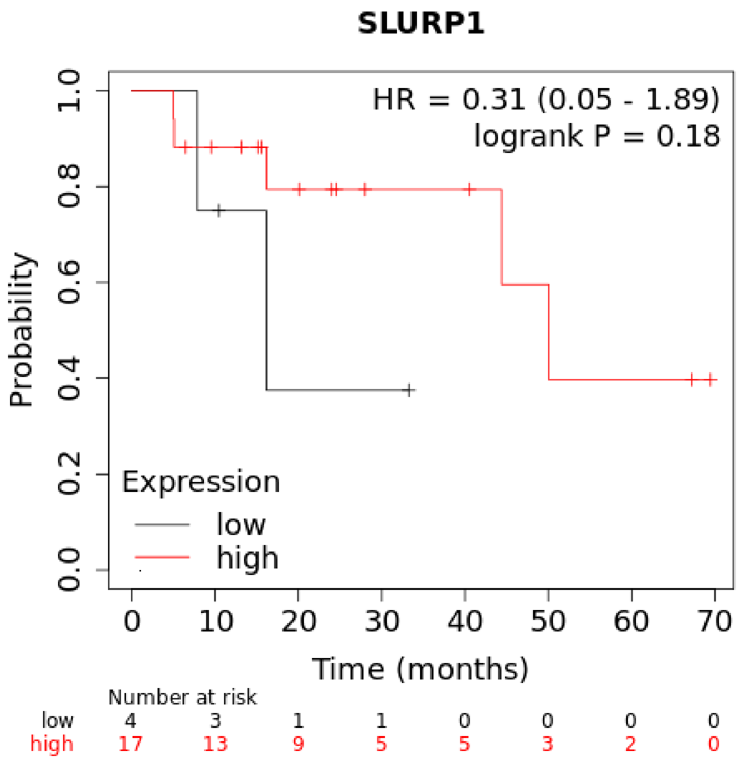

Click here to download the plot in TIFF format

Download plot as a PDF

Download p values vs. cutoff table

Median survival

Low expression cohort (months)	High expression cohort (months)
16.17	50.07

**RNAseq ID:**	LYPD2 -
**Survival:**	OS
**Auto select best cutoff:**	checked
**Follow up threshold:**	all
**Censore at threshold:**	checked
**Compute median over entire database:**	false
**Cutoff value used in analysis:**	0
**Expression range of the probe:**	0 - 2799
**Invert HR values below 1:**	not checked

Restrictions

Tumor type: Pancreatic ductal adenocarcinoma

Restrict analysis to subtypes...

Stage:	all
Gender:	all
Race:	all
Grade:	all
Mutation burden:	all

Restrict analysis based on cellular content...

Basophils:	all
B-cells:	all
CD4+ memory T-cells:	all
CD8+ T-cells:	all
Eosinophils:	all
Macrophages:	all
Mesenchymal stem cells:	decreased
Natural killer T-cells:	all
Regulatory T-cells:	all
Type 1 T-helper cells:	all
Type 2 T-helper cells:	all

Results

**P value:**	0.1434
**FDR:**	100%

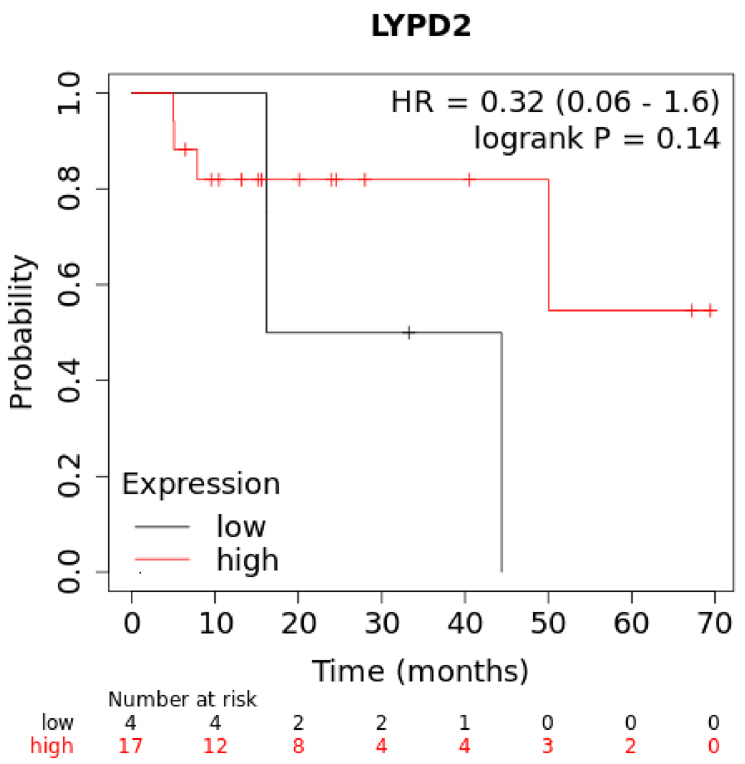

Click here to download the plot in TIFF format

Download plot as a PDF

Download p values vs. cutoff table

Upper quartile survival

Low expression cohort (months)	High expression cohort (months)
16.2	50.07

**RNAseq ID:**	LY6D -
**Survival:**	OS
**Auto select best cutoff:**	checked
**Follow up threshold:**	all
**Censore at threshold:**	checked
**Compute median over entire database:**	false
**Cutoff value used in analysis:**	29
**Expression range of the probe:**	0 - 18030
**Invert HR values below 1:**	not checked

Restrictions

Tumor type: Pancreatic ductal adenocarcinoma

Restrict analysis to subtypes...

Stage:	all
Gender:	all
Race:	all
Grade:	all
Mutation burden:	all

Restrict analysis based on cellular content...

Basophils:	all

B-cells:	all
CD4+ memory T-cells:	all
CD8+ T-cells:	all
Eosinophils:	all
Macrophages:	all
Mesenchymal stem cells:	decreased
Natural killer T-cells:	all
Regulatory T-cells:	all
Type 1 T-helper cells:	all
Type 2 T-helper cells:	all

Results

P value: 0.1077
FDR: 100%

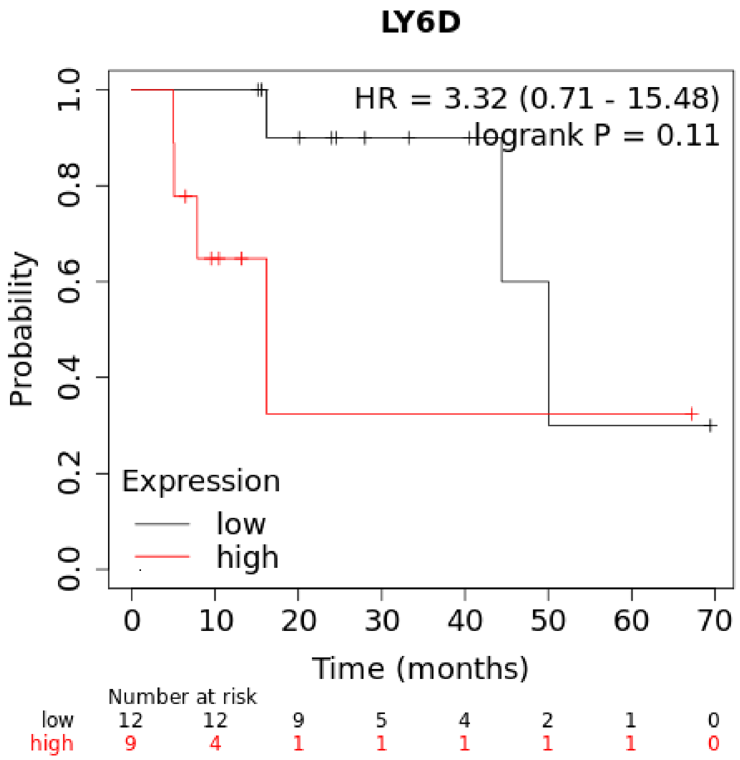

Click here to download the plot in TIFF format

Download plot as a PDF

Download p values vs. cutoff table

Median survival

Low expression cohort (months)	High expression cohort (months)
50.07	16.2

**RNAseq ID:**	GML -
**Survival:**	OS
**Auto select best cutoff:**	checked
**Follow up threshold:**	all
**Censore at threshold:**	checked
**Compute median over entire database:**	false
**Cutoff value used in analysis:**	0
**Expression range of the probe:**	0 - 3
**Invert HR values below 1:**	not checked

Restrictions

Tumor type: Pancreatic ductal adenocarcinoma

Restrict analysis to subtypes...

Stage:	all
Gender:	all
Race:	all
Grade:	all
Mutation burden:	all

Restrict analysis based on cellular content...

Basophils:	all
B-cells:	all
CD4+ memory T-cells:	all
CD8+ T-cells:	all
Eosinophils:	all
Macrophages:	all
Mesenchymal stem cells:	decreased
Natural killer T-cells:	all
Regulatory T-cells:	all
Type 1 T-helper cells:	all
Type 2 T-helper cells:	all

Results

P value: 0.3276
FDR: 100%

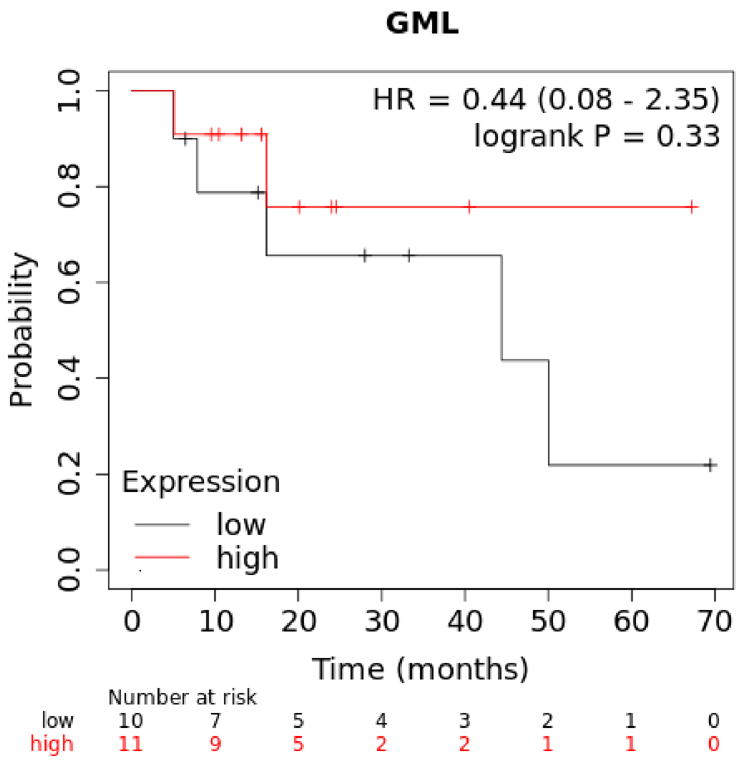

Click here to download the plot in TIFF format

Download plot as a PDF

Download p values vs. cutoff table

Median survival

Low expression cohort (months)	High expression cohort (months)
NA	NA

**RNAseq ID:**	LY6E	-
**Survival:**	OS	
**Auto select best cutoff:**	checked	
**Follow up threshold:**	all	
**Censore at threshold:**	checked	
**Compute median over entire database:**	false	
**Cutoff value used in analysis:**	5903	
**Expression range of the probe:**	254 - 34190	
**Invert HR values below 1:**	not checked	

Restrictions

Tumor type: Pancreatic ductal adenocarcinoma

Restrict analysis to subtypes...

Stage:	all
Gender:	all
Race:	all
Grade:	all
Mutation burden:	all

Restrict analysis based on cellular content...

Basophils:	all
B-cells:	all
CD4+ memory T-cells:	all
CD8+ T-cells:	all
Eosinophils:	all
Macrophages:	all
Mesenchymal stem cells:	decreased
Natural killer T-cells:	all
Regulatory T-cells:	all
Type 1 T-helper cells:	all
Type 2 T-helper cells:	all

Results

**P value:**	0.2644
**FDR:**	100%

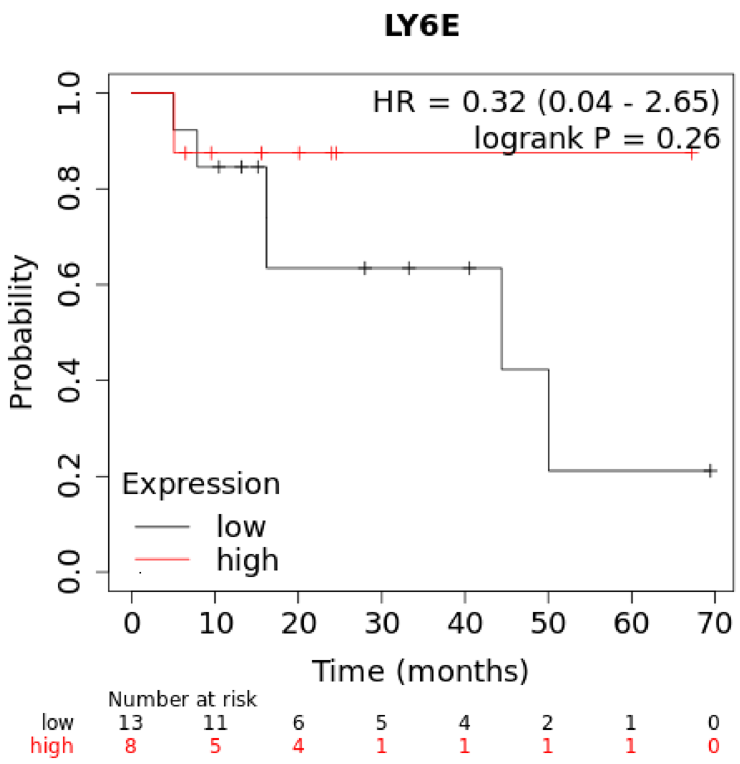

Click here to download the plot in TIFF format

Download plot as a PDF

Download p values vs. cutoff table

Median survival

Low expression cohort (months)	High expression cohort (months)
NA	NA

**RNAseq ID:**	LY6L -
**Survival:**	OS
**Auto select best cutoff:**	checked
**Follow up threshold:**	all
**Censore at threshold:**	checked
**Compute median over entire database:**	false
**Cutoff value used in analysis:**	0
**Expression range of the probe:**	0 - 8
**Invert HR values below 1:**	not checked

Restrictions

Tumor type: Pancreatic ductal adenocarcinoma

Restrict analysis to subtypes...

Stage:	all
Gender:	all
Race:	all
Grade:	all
Mutation burden:	all

Restrict analysis based on cellular content...

Basophils:	all

B-cells:	all
CD4+ memory T-cells:	all
CD8+ T-cells:	all
Eosinophils:	all
Macrophages:	all
Mesenchymal stem cells:	decreased
Natural killer T-cells:	all
Regulatory T-cells:	all
Type 1 T-helper cells:	all
Type 2 T-helper cells:	all

Results

P value: 0.0362
FDR: over 50%

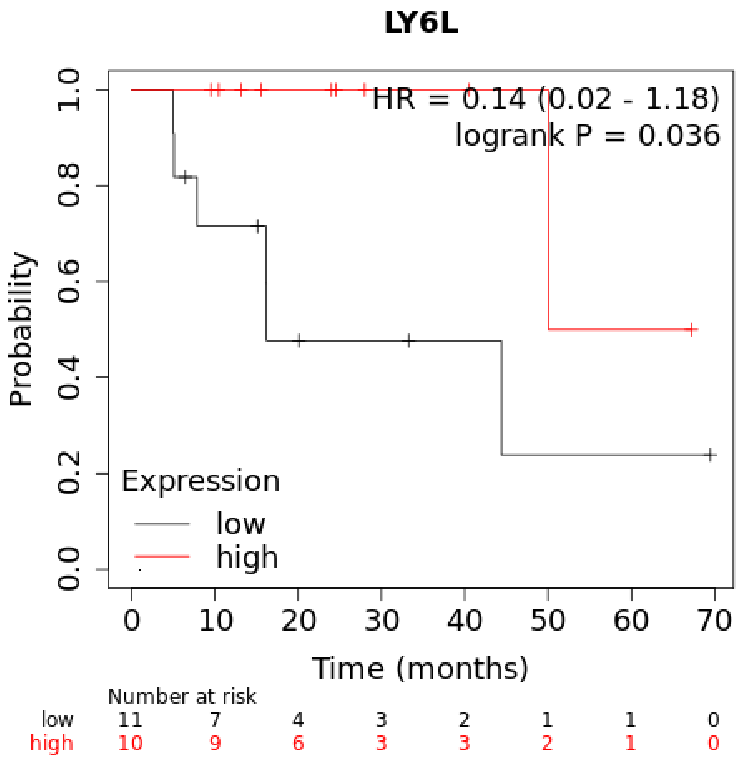

Click here to download the plot in TIFF format

Download plot as a PDF

Download p values vs. cutoff table

Upper quartile survival

Low expression cohort (months)	High expression cohort (months)
7.87	50.07

**RNAseq ID:**	LY6H -
**Survival:**	OS
**Auto select best cutoff:**	checked
**Follow up threshold:**	all
**Censore at threshold:**	checked
**Compute median over entire database:**	false
**Cutoff value used in analysis:**	85
**Expression range of the probe:**	5 - 6037
**Invert HR values below 1:**	not checked

Restrictions

Tumor type: Pancreatic ductal adenocarcinoma

Restrict analysis to subtypes...

Stage:	all
Gender:	all
Race:	all
Grade:	all
Mutation burden:	all

Restrict analysis based on cellular content...

Basophils:	all
B-cells:	all
CD4+ memory T-cells:	all
CD8+ T-cells:	all
Eosinophils:	all
Macrophages:	all
Mesenchymal stem cells:	decreased
Natural killer T-cells:	all
Regulatory T-cells:	all
Type 1 T-helper cells:	all
Type 2 T-helper cells:	all

Results

P value: 0.0906
FDR: 100%

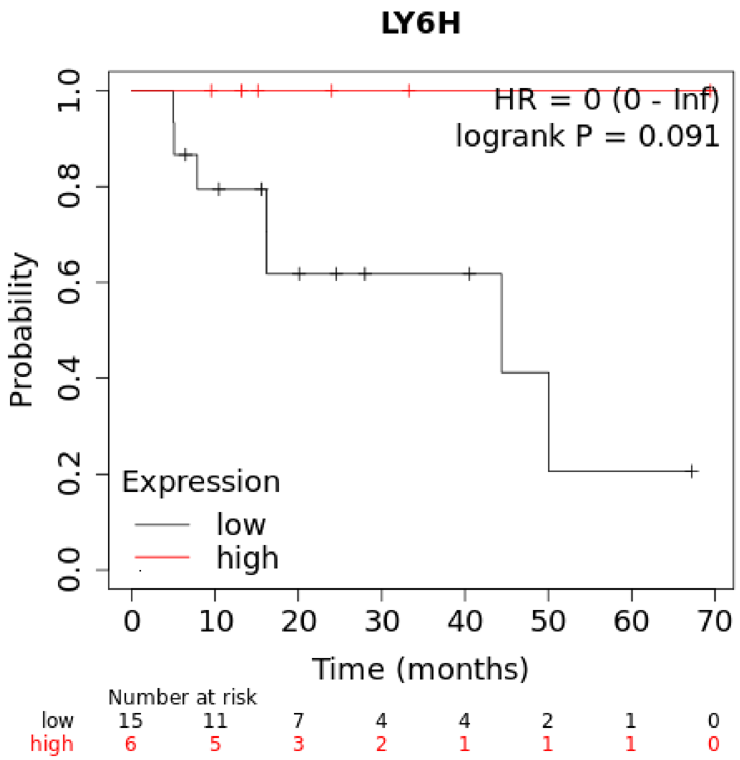

Click here to download the plot in TIFF format

Download plot as a PDF

Download p values vs. cutoff table

Median survival

Low expression cohort (months)	High expression cohort (months)
NA	NA

**RNAseq ID:**	GPIHBP1 -
**Survival:**	OS
**Auto select best cutoff:**	checked
**Follow up threshold:**	all
**Censore at threshold:**	checked
**Compute median over entire database:**	false
**Cutoff value used in analysis:**	86
**Expression range of the probe:**	7 - 344
**Invert HR values below 1:**	not checked

Restrictions

Tumor type: Pancreatic ductal adenocarcinoma

Restrict analysis to subtypes...

Stage:	all
Gender:	all
Race:	all
Grade:	all
Mutation burden:	all

Restrict analysis based on cellular content...

Basophils:	all
B-cells:	all
CD4+ memory T-cells:	all
CD8+ T-cells:	all
Eosinophils:	all
Macrophages:	all
Mesenchymal stem cells:	decreased
Natural killer T-cells:	all
Regulatory T-cells:	all
Type 1 T-helper cells:	all
Type 2 T-helper cells:	all

Results

**P value:**	0.1188
**FDR:**	100%

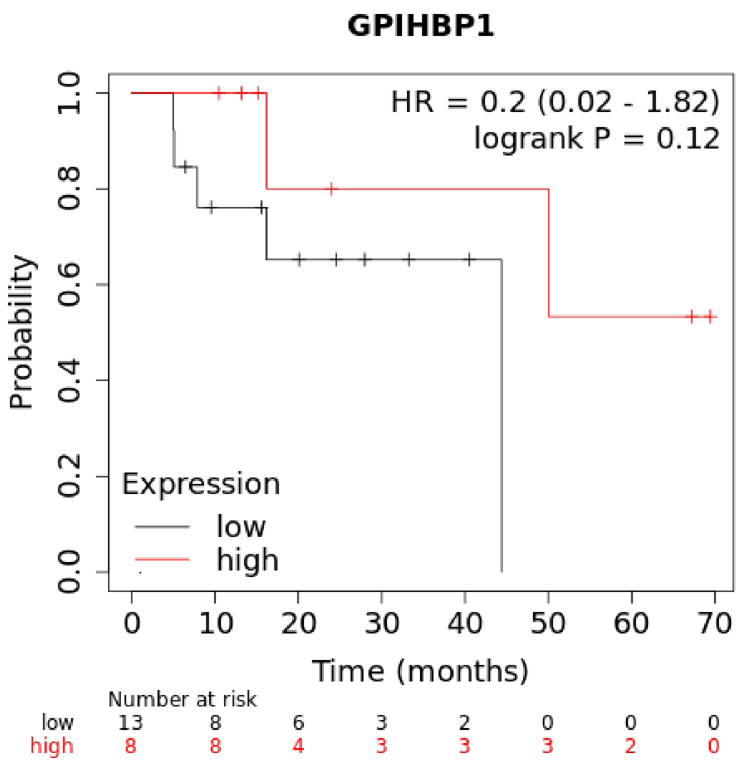

Click here to download the plot in TIFF format

Download plot as a PDF

Download p values vs. cutoff table

Upper quartile survival

Low expression cohort (months)	High expression cohort (months)
16.17	50.07

**RNAseq ID:**	LYPD4 -
**Survival:**	OS
**Auto select best cutoff:**	checked
**Follow up threshold:**	all
**Censore at threshold:**	checked
**Compute median over entire database:**	false
**Cutoff value used in analysis:**	0
**Expression range of the probe:**	0 - 4
**Invert HR values below 1:**	not checked

Restrictions

Tumor type: Pancreatic ductal adenocarcinoma

Restrict analysis to subtypes...

Stage:	all
Gender:	all
Race:	all
Grade:	all
Mutation burden:	all

Restrict analysis based on cellular content...

Basophils:	all

B-cells:	all
CD4+ memory T-cells:	all
CD8+ T-cells:	all
Eosinophils:	all
Macrophages:	all
Mesenchymal stem cells:	decreased
Natural killer T-cells:	all
Regulatory T-cells:	all
Type 1 T-helper cells:	all
Type 2 T-helper cells:	all

Results

P value: 0.1547
FDR: 100%

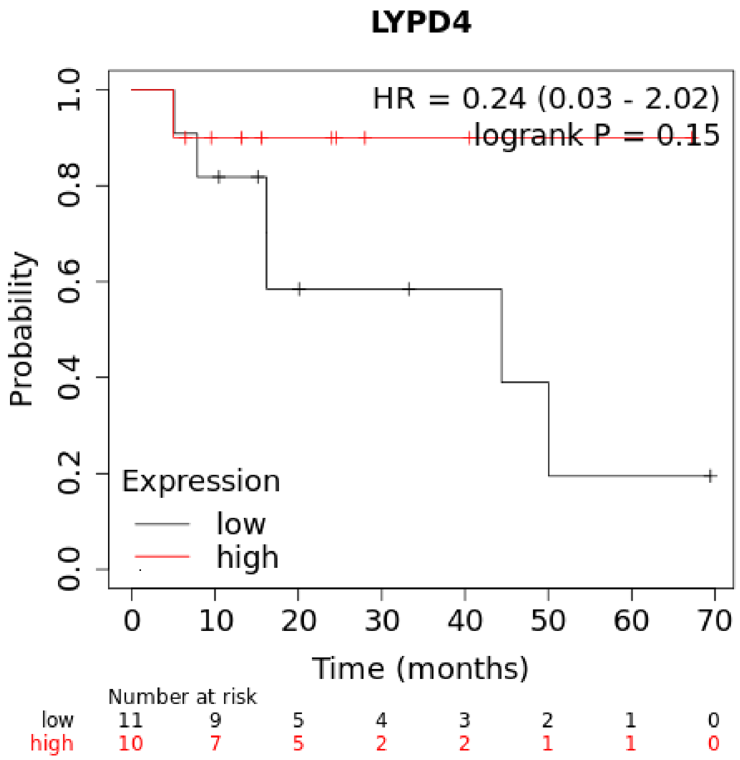

Click here to download the plot in TIFF format

Download plot as a PDF

Download p values vs. cutoff table

Median survival

Low expression cohort (months)	High expression cohort (months)
NA	NA

**RNAseq ID:**	CD177 -
**Survival:**	OS
**Auto select best cutoff:**	checked
**Follow up threshold:**	all
**Censore at threshold:**	checked
**Compute median over entire database:**	false
**Cutoff value used in analysis:**	38
**Expression range of the probe:**	0 - 1414
**Invert HR values below 1:**	not checked

Restrictions

Tumor type: Pancreatic ductal adenocarcinoma

Restrict analysis to subtypes...

Stage: all
Gender: all
Race: all
Grade: all
Mutation burden: all

Restrict analysis based on cellular content...

Basophils: all
B-cells: all
CD4+ memory T-cells: all
CD8+ T-cells: all
Eosinophils: all
Macrophages: all
Mesenchymal stem cells: decreased
Natural killer T-cells: all
Regulatory T-cells: all
Type 1 T-helper cells: all
Type 2 T-helper cells: all

Results

P value: 0.1129
FDR: 100%

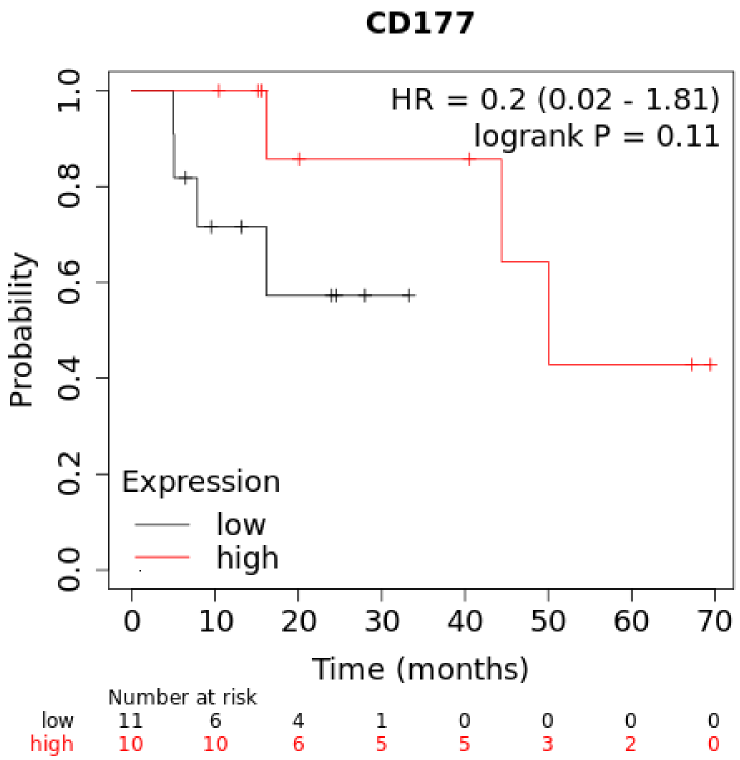

Click here to download the plot in TIFF format

Download plot as a PDF

Download p values vs. cutoff table

Upper quartile survival

Low expression cohort (months)	High expression cohort (months)
7.87	44.4

**RNAseq ID:**	TEX101 -
**Survival:**	OS
**Auto select best cutoff:**	checked
**Follow up threshold:**	all
**Censore at threshold:**	checked
**Compute median over entire database:**	false
**Cutoff value used in analysis:**	1
**Expression range of the probe:**	0 - 11
**Invert HR values below 1:**	not checked

Restrictions

Tumor type: Pancreatic ductal adenocarcinoma

Restrict analysis to subtypes...

Stage:	all
Gender:	all
Race:	all
Grade:	all
Mutation burden:	all

Restrict analysis based on cellular content...

Basophils:	all
B-cells:	all
CD4+ memory T-cells:	all
CD8+ T-cells:	all
Eosinophils:	all
Macrophages:	all
Mesenchymal stem cells:	decreased
Natural killer T-cells:	all
Regulatory T-cells:	all
Type 1 T-helper cells:	all
Type 2 T-helper cells:	all

Results

**P value:**	0.0335
**FDR:**	over 50%

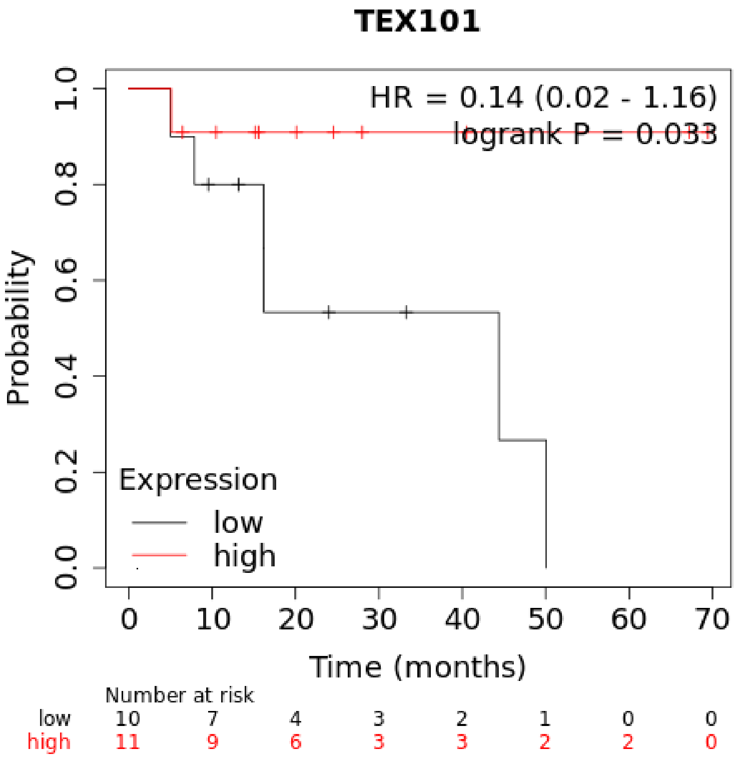

[Click here to download the plot in TIFF format]

[Download plot as a PDF]

[Download p values vs. cutoff table]

Median survival

Low expression cohort (months)	High expression cohort (months)
NA	NA

**RNAseq ID:**	LYPD3 -
**Survival:**	OS
**Auto select best cutoff:**	checked
**Follow up threshold:**	all
**Censore at threshold:**	checked
**Compute median over entire database:**	false
**Cutoff value used in analysis:**	94
**Expression range of the probe:**	16 - 598
**Invert HR values below 1:**	not checked

Restrictions

Tumor type: Pancreatic ductal adenocarcinoma

Restrict analysis to subtypes...

Stage:	all
Gender:	all
Race:	all
Grade:	all
Mutation burden:	all

Restrict analysis based on cellular content...

Basophils:	all

B-cells:	all
CD4+ memory T-cells:	all
CD8+ T-cells:	all
Eosinophils:	all
Macrophages:	all
Mesenchymal stem cells:	decreased
Natural killer T-cells:	all
Regulatory T-cells:	all
Type 1 T-helper cells:	all
Type 2 T-helper cells:	all

Results

P value: 0.0668
FDR: 100%

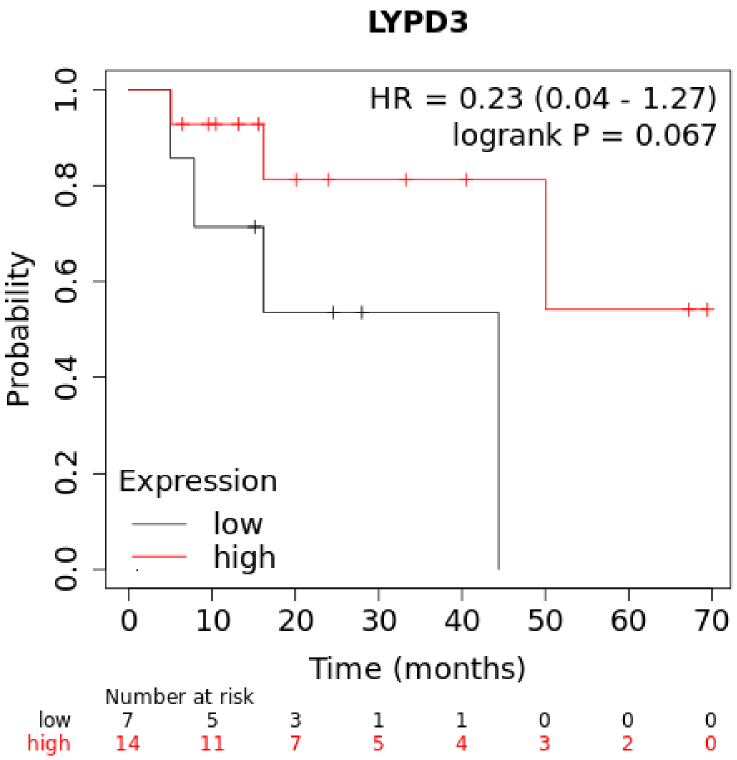

Click here to download the plot in TIFF format

Download plot as a PDF

Download p values vs. cutoff table

Upper quartile survival

Low expression cohort (months)	High expression cohort (months)
7.87	50.07

**RNAseq ID:**	PINLYP -
**Survival:**	OS
**Auto select best cutoff:**	checked
**Follow up threshold:**	all
**Censore at threshold:**	checked
**Compute median over entire database:**	false
**Cutoff value used in analysis:**	72
**Expression range of the probe:**	6 - 134
**Invert HR values below 1:**	not checked

Restrictions

Tumor type: Pancreatic ductal adenocarcinoma

Restrict analysis to subtypes...

Stage:	all
Gender:	all
Race:	all
Grade:	all
Mutation burden:	all

Restrict analysis based on cellular content...

Basophils:	all
B-cells:	all
CD4+ memory T-cells:	all
CD8+ T-cells:	all
Eosinophils:	all
Macrophages:	all
Mesenchymal stem cells:	decreased
Natural killer T-cells:	all
Regulatory T-cells:	all
Type 1 T-helper cells:	all
Type 2 T-helper cells:	all

Results

P value: 0.0775
FDR: 100%

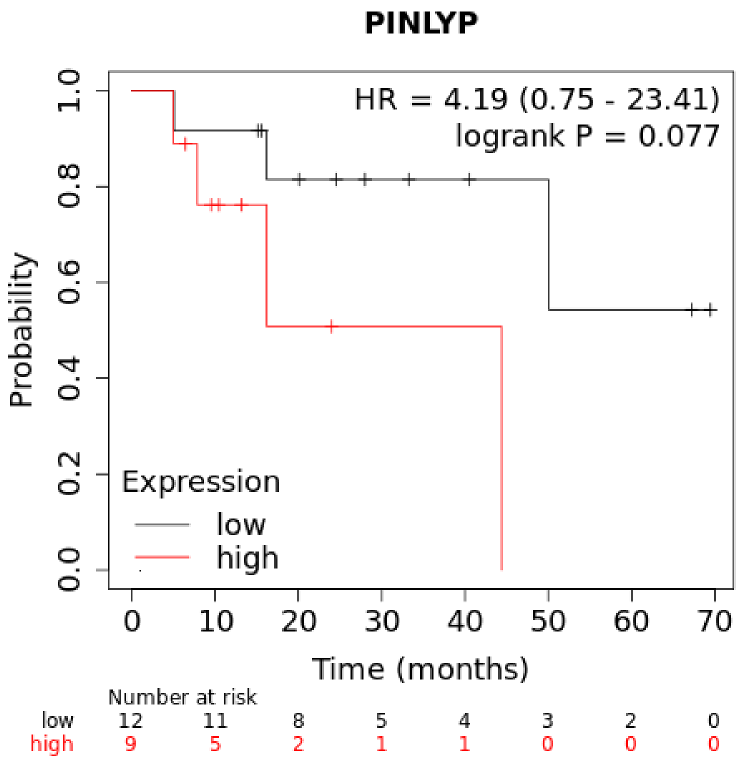

Click here to download the plot in TIFF format

Download plot as a PDF

Download p values vs. cutoff table

Upper quartile survival

Low expression cohort (months)	High expression cohort (months)
50.07	16.2

**RNAseq ID:**	PLAUR -
**Survival:**	OS
**Auto select best cutoff:**	checked
**Follow up threshold:**	all
**Censore at threshold:**	checked
**Compute median over entire database:**	false
**Cutoff value used in analysis:**	2606
**Expression range of the probe:**	47 - 14016
**Invert HR values below 1:**	not checked

Restrictions

Tumor type: Pancreatic ductal adenocarcinoma

Restrict analysis to subtypes...

Stage:	all
Gender:	all
Race:	all
Grade:	all
Mutation burden:	all

Restrict analysis based on cellular content...

Basophils:	all
B-cells:	all
CD4+ memory T-cells:	all
CD8+ T-cells:	all
Eosinophils:	all
Macrophages:	all
Mesenchymal stem cells:	decreased
Natural killer T-cells:	all
Regulatory T-cells:	all
Type 1 T-helper cells:	all
Type 2 T-helper cells:	all

Results

P value: 0.1369
FDR: 100%

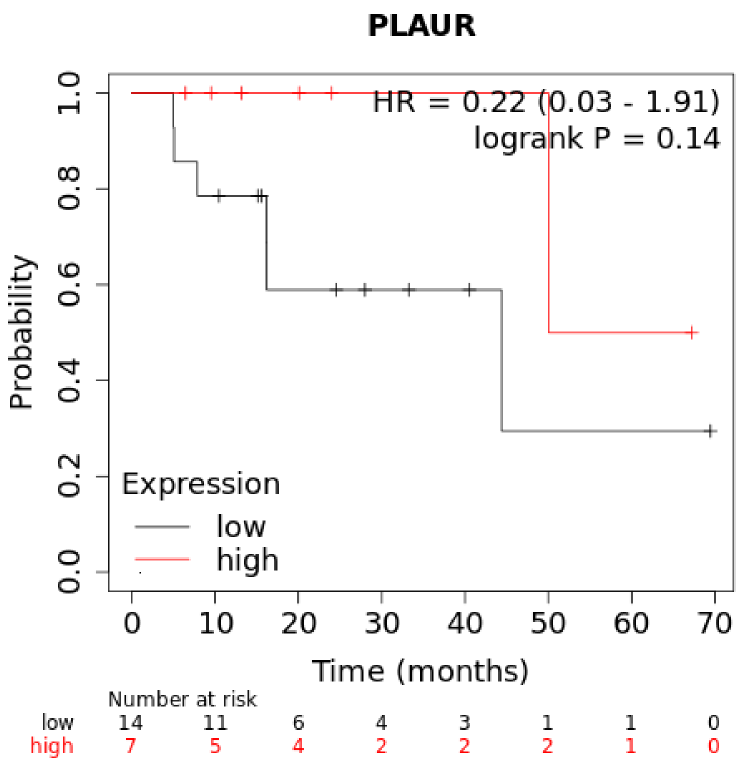

Click here to download the plot in TIFF format

Download plot as a PDF

Download p values vs. cutoff table

Upper quartile survival

Low expression cohort (months)	High expression cohort (months)
16.17	50.07

**RNAseq ID:**	LYPD5 -
**Survival:**	OS
**Auto select best cutoff:**	checked
**Follow up threshold:**	all
**Censore at threshold:**	checked
**Compute median over entire database:**	false
**Cutoff value used in analysis:**	83
**Expression range of the probe:**	1 - 419
**Invert HR values below 1:**	not checked

Restrictions

Tumor type: Pancreatic ductal adenocarcinoma

Restrict analysis to subtypes...

Stage:	all
Gender:	all
Race:	all
Grade:	all
Mutation burden:	all

Restrict analysis based on cellular content...

Basophils:	all

B-cells:	all
CD4+ memory T-cells:	all
CD8+ T-cells:	all
Eosinophils:	all
Macrophages:	all
Mesenchymal stem cells:	decreased
Natural killer T-cells:	all
Regulatory T-cells:	all
Type 1 T-helper cells:	all
Type 2 T-helper cells:	all

Results

P value: 0.1327
FDR: 100%

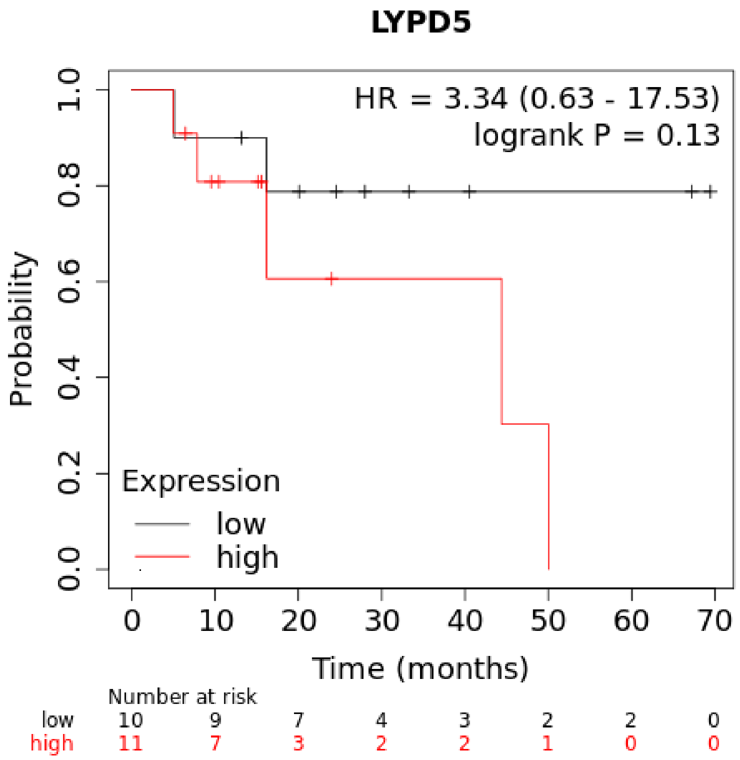

Click here to download the plot in TIFF format

Download plot as a PDF

Download p values vs. cutoff table

Median survival

Low expression cohort (months)	High expression cohort (months)
NA	NA

**RNAseq ID:**	SPACA4 -
**Survival:**	OS
**Auto select best cutoff:**	checked
**Follow up threshold:**	all
**Censore at threshold:**	checked
**Compute median over entire database:**	false
**Cutoff value used in analysis:**	5
**Expression range of the probe:**	1 - 204
**Invert HR values below 1:**	not checked

Restrictions

Tumor type: Pancreatic ductal adenocarcinoma

Restrict analysis to subtypes...

Stage:	all
Gender:	all
Race:	all
Grade:	all
Mutation burden:	all

Restrict analysis based on cellular content...

Basophils:	all
B-cells:	all
CD4+ memory T-cells:	all
CD8+ T-cells:	all
Eosinophils:	all
Macrophages:	all
Mesenchymal stem cells:	decreased
Natural killer T-cells:	all
Regulatory T-cells:	all
Type 1 T-helper cells:	all
Type 2 T-helper cells:	all

Results

P value: 0.1558
FDR: 100%

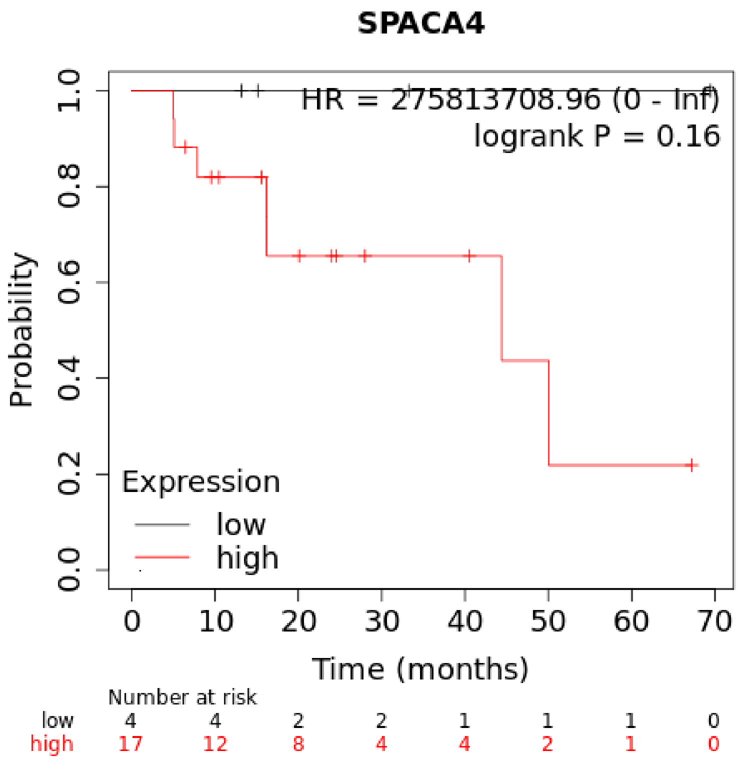

Click here to download the plot in TIFF format

Download plot as a PDF

Download p values vs. cutoff table

Median survival

Low expression cohort (months)	High expression cohort (months)
NA	NA

**RNAseq ID:**	ACRV1 -
**Survival:**	OS
**Auto select best cutoff:**	checked
**Follow up threshold:**	all
**Censore at threshold:**	checked
**Compute median over entire database:**	false
**Cutoff value used in analysis:**	2
**Expression range of the probe:**	0 - 12
**Invert HR values below 1:**	not checked

Restrictions

Tumor type: Pancreatic ductal adenocarcinoma

Restrict analysis to subtypes...

Stage:	all
Gender:	all
Race:	all
Grade:	all
Mutation burden:	all

Restrict analysis based on cellular content...

Basophils:	all
B-cells:	all
CD4+ memory T-cells:	all
CD8+ T-cells:	all
Eosinophils:	all
Macrophages:	all
Mesenchymal stem cells:	decreased
Natural killer T-cells:	all
Regulatory T-cells:	all
Type 1 T-helper cells:	all
Type 2 T-helper cells:	all

Results

**P value:**	0.0952
**FDR:**	100%

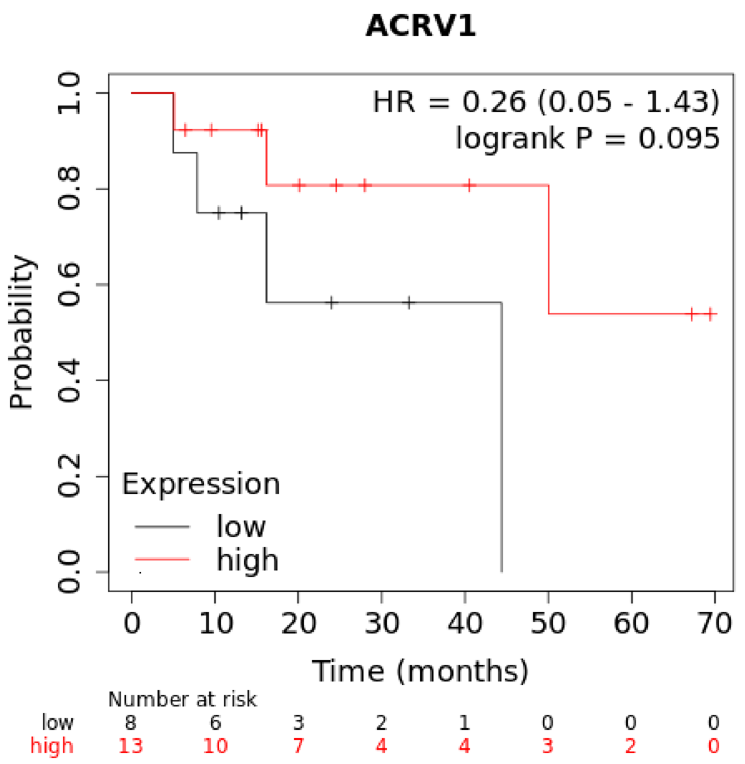

Click here to download the plot in TIFF format

Download plot as a PDF

Download p values vs. cutoff table

Upper quartile survival

Low expression cohort (months)	High expression cohort (months)
16.2	50.07

**RNAseq ID:**	PATE1 -
**Survival:**	OS
**Auto select best cutoff:**	checked
**Follow up threshold:**	all
**Censore at threshold:**	checked
**Compute median over entire database:**	false
**Cutoff value used in analysis:**	0
**Expression range of the probe:**	0 - 0
**Invert HR values below 1:**	not checked

Restrictions

Tumor type: Pancreatic ductal adenocarcinoma

Restrict analysis to subtypes...

Stage:	all
Gender:	all
Race:	all
Grade:	all
Mutation burden:	all

Restrict analysis based on cellular content...

Basophils:	all

B-cells:	all
CD4+ memory T-cells:	all
CD8+ T-cells:	all
Eosinophils:	all
Macrophages:	all
Mesenchymal stem cells:	decreased
Natural killer T-cells:	all
Regulatory T-cells:	all
Type 1 T-helper cells:	all
Type 2 T-helper cells:	all

Results

P value: 0.0589
FDR: 100%

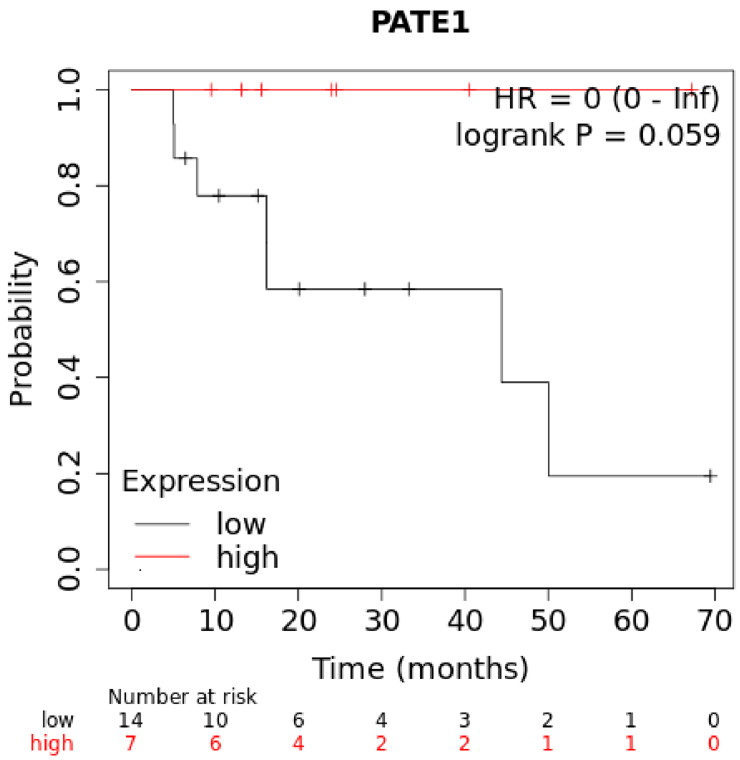

Click here to download the plot in TIFF format

Download plot as a PDF

Download p values vs. cutoff table

Median survival

Low expression cohort (months)	High expression cohort (months)
NA	NA

**RNAseq ID:**	PATE2 -
**Survival:**	OS
**Auto select best cutoff:**	checked
**Follow up threshold:**	all
**Censore at threshold:**	checked
**Compute median over entire database:**	false
**Cutoff value used in analysis:**	0
**Expression range of the probe:**	0 - 2
**Invert HR values below 1:**	not checked

Restrictions

Tumor type: Pancreatic ductal adenocarcinoma

Restrict analysis to subtypes...

Stage:	all
Gender:	all
Race:	all
Grade:	all
Mutation burden:	all

Restrict analysis based on cellular content...

Basophils:	all
B-cells:	all
CD4+ memory T-cells:	all
CD8+ T-cells:	all
Eosinophils:	all
Macrophages:	all
Mesenchymal stem cells:	decreased
Natural killer T-cells:	all
Regulatory T-cells:	all
Type 1 T-helper cells:	all
Type 2 T-helper cells:	all

Results

P value: 0.2071
FDR: 100%

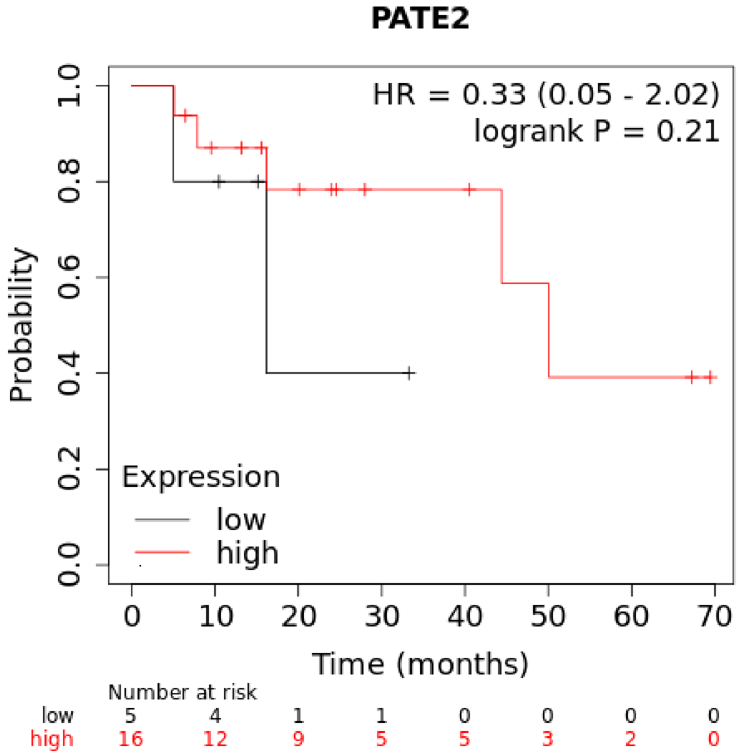

Click here to download the plot in TIFF format

Download plot as a PDF

Download p values vs. cutoff table

Median survival

Low expression cohort (months)	High expression cohort (months)
16.17	50.07

**RNAseq ID:**	PATE3 -
**Survival:**	OS
**Auto select best cutoff:**	checked
**Follow up threshold:**	all
**Censore at threshold:**	checked
**Compute median over entire database:**	false
**Cutoff value used in analysis:**	0
**Expression range of the probe:**	0 - 1
**Invert HR values below 1:**	not checked

Restrictions

Tumor type: Pancreatic ductal adenocarcinoma

Restrict analysis to subtypes...

Stage:	all
Gender:	all
Race:	all
Grade:	all
Mutation burden:	all

Restrict analysis based on cellular content...

Basophils:	all
B-cells:	all
CD4+ memory T-cells:	all
CD8+ T-cells:	all
Eosinophils:	all
Macrophages:	all
Mesenchymal stem cells:	decreased
Natural killer T-cells:	all
Regulatory T-cells:	all
Type 1 T-helper cells:	all
Type 2 T-helper cells:	all

Results

P value: 0.0589
FDR: 100%

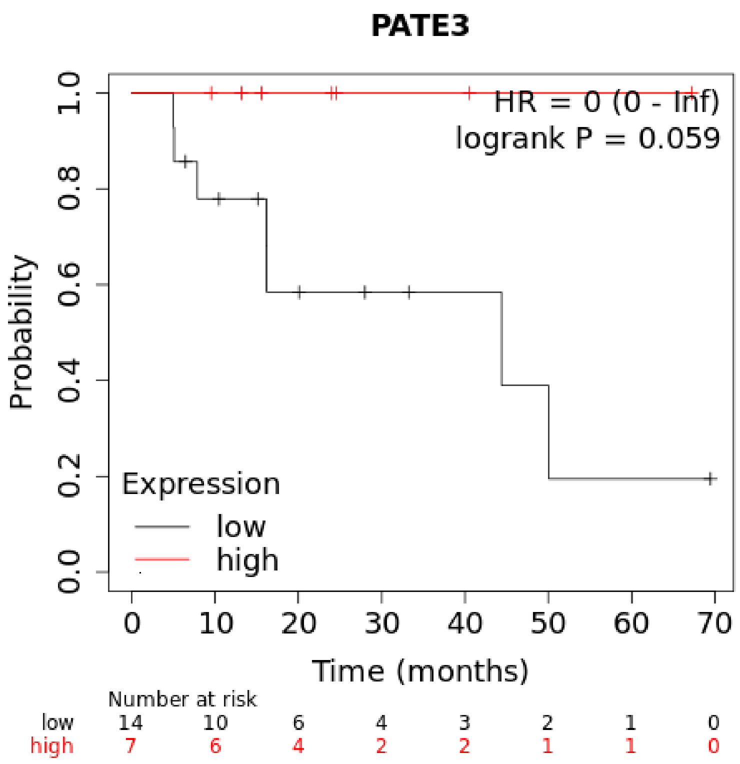

Click here to download the plot in TIFF format

Download plot as a PDF

Download p values vs. cutoff table

Median survival

Low expression cohort (months)	High expression cohort (months)
NA	NA

**RNAseq ID:**	PATE4 -
**Survival:**	OS
**Auto select best cutoff:**	checked
**Follow up threshold:**	all
**Censore at threshold:**	checked
**Compute median over entire database:**	false
**Cutoff value used in analysis:**	0
**Expression range of the probe:**	0 - 1
**Invert HR values below 1:**	not checked

Restrictions

Tumor type: Pancreatic ductal adenocarcinoma

Restrict analysis to subtypes...

Stage:	all
Gender:	all
Race:	all
Grade:	all
Mutation burden:	all

Restrict analysis based on cellular content...

Basophils:	all

B-cells:	all
CD4+ memory T-cells:	all
CD8+ T-cells:	all
Eosinophils:	all
Macrophages:	all
Mesenchymal stem cells:	decreased
Natural killer T-cells:	all
Regulatory T-cells:	all
Type 1 T-helper cells:	all
Type 2 T-helper cells:	all

Results

P value: 0.3468
FDR: 100%

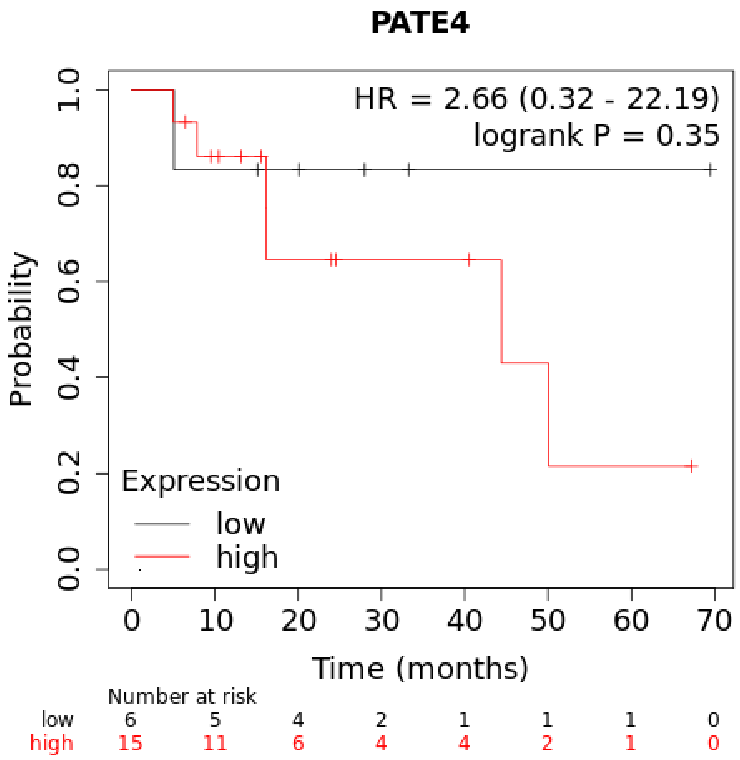

Click here to download the plot in TIFF format

Download plot as a PDF

Download p values vs. cutoff table

Median survival

Low expression cohort (months)	High expression cohort (months)
NA	NA

**RNAseq ID:**	CD59 -
**Survival:**	OS
**Auto select best cutoff:**	checked
**Follow up threshold:**	all
**Censore at threshold:**	checked
**Compute median over entire database:**	false
**Cutoff value used in analysis:**	14483
**Expression range of the probe:**	2610 - 22224
**Invert HR values below 1:**	not checked

Restrictions

Tumor type: Pancreatic ductal adenocarcinoma

Restrict analysis to subtypes...

Stage:	all
Gender:	all
Race:	all
Grade:	all
Mutation burden:	all

Restrict analysis based on cellular content...

Basophils:	all
B-cells:	all
CD4+ memory T-cells:	all
CD8+ T-cells:	all
Eosinophils:	all
Macrophages:	all
Mesenchymal stem cells:	decreased
Natural killer T-cells:	all
Regulatory T-cells:	all
Type 1 T-helper cells:	all
Type 2 T-helper cells:	all

Results

P value: 0.2232
FDR: 100%

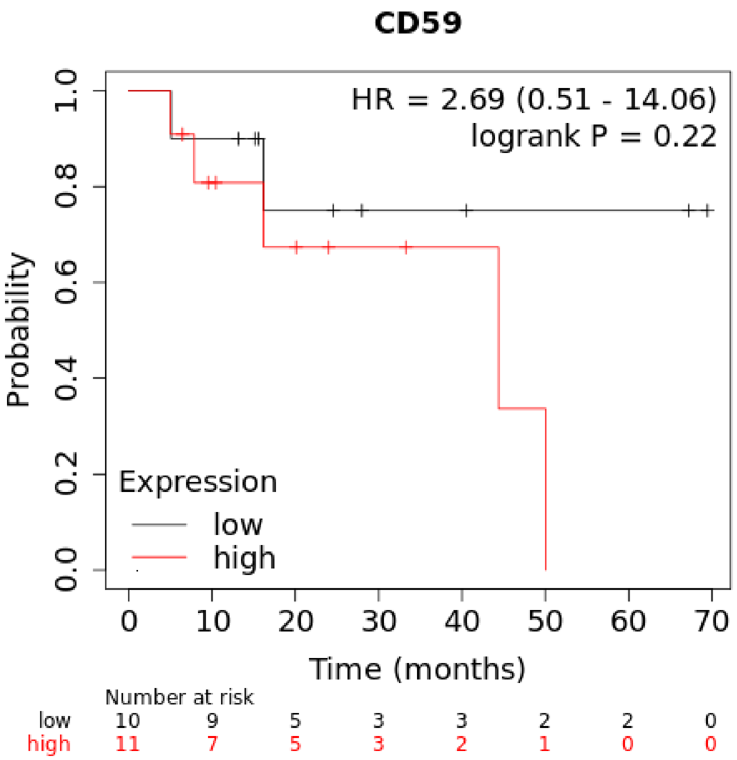

Click here to download the plot in TIFF format

Download plot as a PDF

Download p values vs. cutoff table

Median survival

Low expression cohort (months)	High expression cohort (months)
NA	NA

**RNAseq ID:**	LY6G6C -
**Survival:**	OS
**Auto select best cutoff:**	checked
**Follow up threshold:**	all
**Censore at threshold:**	checked
**Compute median over entire database:**	false
**Cutoff value used in analysis:**	3
**Expression range of the probe:**	0 - 42
**Invert HR values below 1:**	not checked

Restrictions

Tumor type: Pancreatic ductal adenocarcinoma

Restrict analysis to subtypes...

Stage:	all
Gender:	all
Race:	all
Grade:	all
Mutation burden:	all

Restrict analysis based on cellular content...

Basophils:	all
B-cells:	all
CD4+ memory T-cells:	all
CD8+ T-cells:	all
Eosinophils:	all
Macrophages:	all
Mesenchymal stem cells:	decreased
Natural killer T-cells:	all
Regulatory T-cells:	all
Type 1 T-helper cells:	all
Type 2 T-helper cells:	all

Results

**P value:**	0.0465
**FDR:**	over 50%

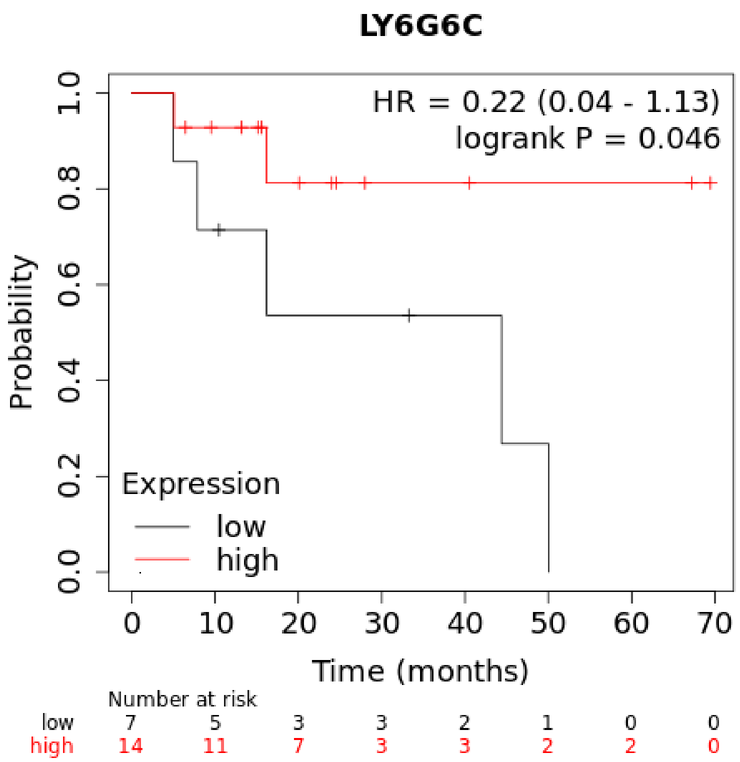

Click here to download the plot in TIFF format

Download plot as a PDF

Download p values vs. cutoff table

Median survival

Low expression cohort (months)	High expression cohort (months)
NA	NA

**RNAseq ID:**	LY6G6D -
**Survival:**	OS
**Auto select best cutoff:**	checked
**Follow up threshold:**	all
**Censore at threshold:**	checked
**Compute median over entire database:**	false
**Cutoff value used in analysis:**	0
**Expression range of the probe:**	0 - 1
**Invert HR values below 1:**	not checked

Restrictions

Tumor type: Pancreatic ductal adenocarcinoma

Restrict analysis to subtypes...

Stage:	all
Gender:	all
Race:	all
Grade:	all
Mutation burden:	all

Restrict analysis based on cellular content...

Basophils:	all

B-cells:	all
CD4+ memory T-cells:	all
CD8+ T-cells:	all
Eosinophils:	all
Macrophages:	all
Mesenchymal stem cells:	decreased
Natural killer T-cells:	all
Regulatory T-cells:	all
Type 1 T-helper cells:	all
Type 2 T-helper cells:	all

Results

P value: 0.1651
FDR: 100%

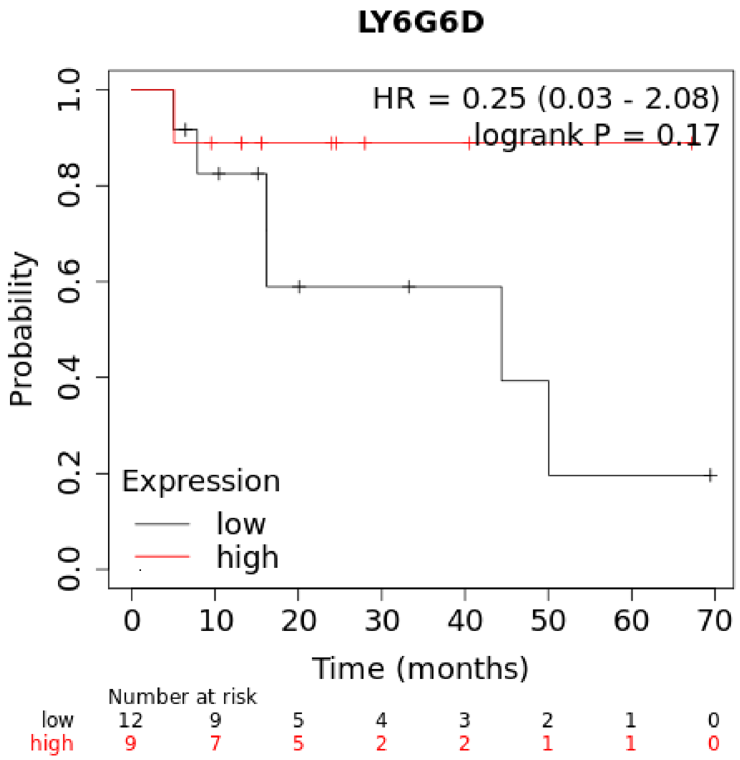

Click here to download the plot in TIFF format

Download plot as a PDF

Download p values vs. cutoff table

Median survival

Low expression cohort (months)	High expression cohort (months)
NA	NA

**RNAseq ID:**	LY6G6F -
**Survival:**	OS
**Auto select best cutoff:**	checked
**Follow up threshold:**	all
**Censore at threshold:**	checked
**Compute median over entire database:**	false
**Cutoff value used in analysis:**	0
**Expression range of the probe:**	0 - 1
**Invert HR values below 1:**	not checked

Restrictions

Tumor type: Pancreatic ductal adenocarcinoma

Restrict analysis to subtypes...

Stage: all
Gender: all
Race: all
Grade: all
Mutation burden: all

Restrict analysis based on cellular content...

Basophils: all
B-cells: all
CD4+ memory T-cells: all
CD8+ T-cells: all
Eosinophils: all
Macrophages: all
Mesenchymal stem cells: decreased
Natural killer T-cells: all
Regulatory T-cells: all
Type 1 T-helper cells: all
Type 2 T-helper cells: all

Results

P value: 0.3916
FDR: 100%

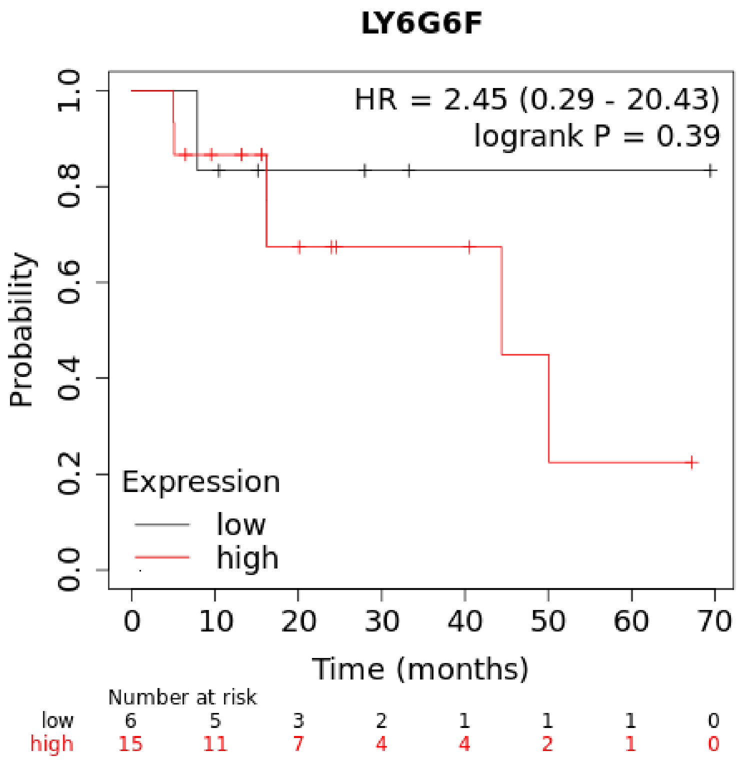

Click here to download the plot in TIFF format

Download plot as a PDF

Download p values vs. cutoff table

Median survival

Low expression cohort (months)	High expression cohort (months)
NA	NA

**RNAseq ID:**	LY6G5C -
**Survival:**	OS
**Auto select best cutoff:**	checked
**Follow up threshold:**	all
**Censore at threshold:**	checked
**Compute median over entire database:**	false
**Cutoff value used in analysis:**	58
**Expression range of the probe:**	31 - 586
**Invert HR values below 1:**	not checked

Restrictions

Tumor type: Pancreatic ductal adenocarcinoma

Restrict analysis to subtypes...

Stage:	all
Gender:	all
Race:	all
Grade:	all
Mutation burden:	all

Restrict analysis based on cellular content...

Basophils:	all
B-cells:	all
CD4+ memory T-cells:	all
CD8+ T-cells:	all
Eosinophils:	all
Macrophages:	all
Mesenchymal stem cells:	decreased
Natural killer T-cells:	all
Regulatory T-cells:	all
Type 1 T-helper cells:	all
Type 2 T-helper cells:	all

Results

**P value:**	0.1551
**FDR:**	100%

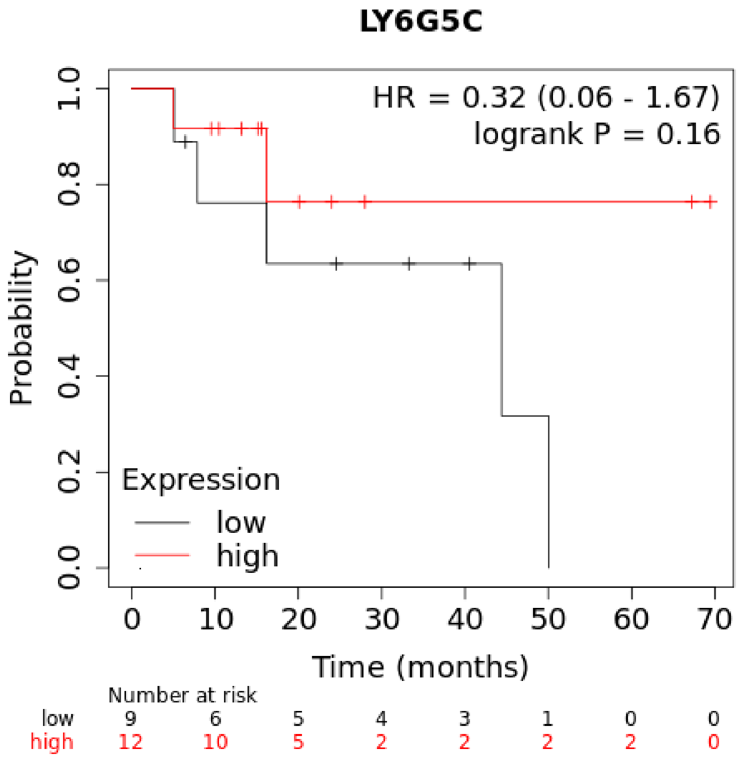

Click here to download the plot in TIFF format

Download plot as a PDF

Download p values vs. cutoff table

Median survival

Low expression cohort (months)	High expression cohort (months)
NA	NA

**RNAseq ID:**	LY6G5B -
**Survival:**	OS
**Auto select best cutoff:**	checked
**Follow up threshold:**	all
**Censore at threshold:**	checked
**Compute median over entire database:**	false
**Cutoff value used in analysis:**	67
**Expression range of the probe:**	20 - 173
**Invert HR values below 1:**	not checked

Restrictions

Tumor type: Pancreatic ductal adenocarcinoma

Restrict analysis to subtypes...

Stage:	all
Gender:	all
Race:	all
Grade:	all
Mutation burden:	all

Restrict analysis based on cellular content...

Basophils:	all

B-cells:	all
CD4+ memory T-cells:	all
CD8+ T-cells:	all
Eosinophils:	all
Macrophages:	all
Mesenchymal stem cells:	decreased
Natural killer T-cells:	all
Regulatory T-cells:	all
Type 1 T-helper cells:	all
Type 2 T-helper cells:	all

Results

P value: 0.0414
FDR: over 50%

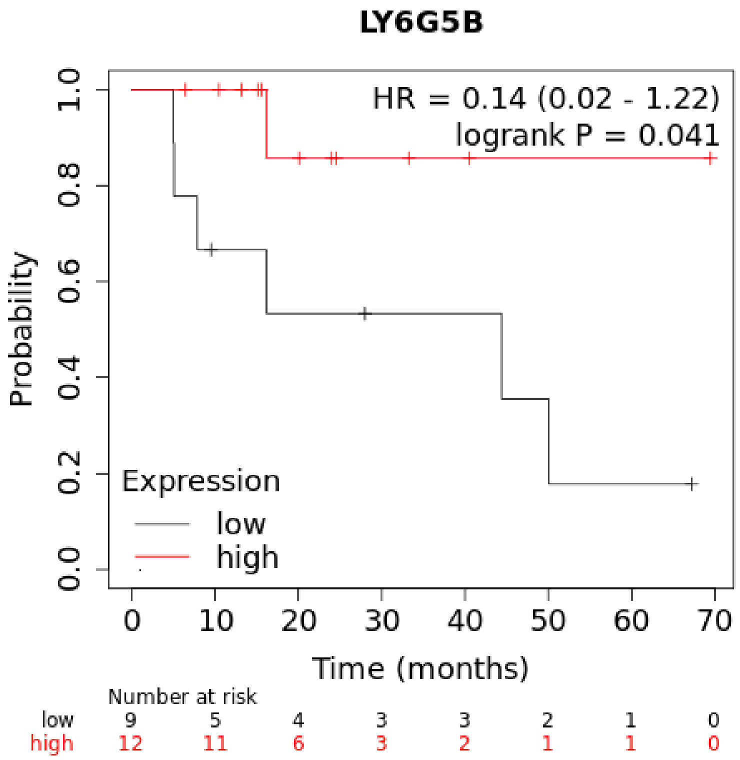

Click here to download the plot in TIFF format

Download plot as a PDF

Download p values vs. cutoff table

Median survival

Low expression cohort (months)	High expression cohort (months)
NA	NA

You can save the plots by right-clicking the image and then selecting "Save image as...". To generate a high resolution TIFF image, please adjust the "Settings" in the analysis page.

KMplot.com Copyright © 2009-2020 | Contact | Sitemap

Figure S3: KM plots and other raw data for the data depicted in Table 3

Pan-cancer

KM plotter | Home | Vote | Download | Updates | Contact

The desired RNAseq ID is valid: PSCA (-), LY6K (-), SLURP1 (-), LYPD2 (-), LY6D (-), GML (-), LY6E (-), LY6L (-), LY6H (-), GPIHBP1 (-), LYPD4 (-), CD177 (-), TEX101 (-), LYPD3 (-), PINLYP (-), PLAUR (-), LYPD5 (-), SPACA4 (-), ACRV1 (-), PATE1 (-), PATE2 (-), PATE3 (-), PATE4 (-), CD59 (-), LY6G6C (-), LY6G6D (-), LY6G6F (-), LY6G5C (-), LY6G5B (-),

**RNAseq ID:**	PSCA -
**Survival:**	OS
**Auto select best cutoff:**	checked
**Follow up threshold:**	all
**Censore at threshold:**	checked
**Compute median over entire database:**	false
**Cutoff value used in analysis:**	371
**Expression range of the probe:**	3 - 49321
**Invert HR values below 1:**	not checked

Restrictions

Tumor type: Pancreatic ductal adenocarcinoma

Restrict analysis to subtypes...

Stage:	all
Gender:	all
Race:	all
Grade:	all
Mutation burden:	all

Restrict analysis based on cellular content...

Basophils:	all
B-cells:	all
CD4+ memory T-cells:	all
CD8+ T-cells:	all
Eosinophils:	all
Macrophages:	all
Mesenchymal stem cells:	all
Natural killer T-cells:	all
Regulatory T-cells:	enriched
Type 1 T-helper cells:	all
Type 2 T-helper cells:	all

Results

P value: 0.0003
FDR: 2%

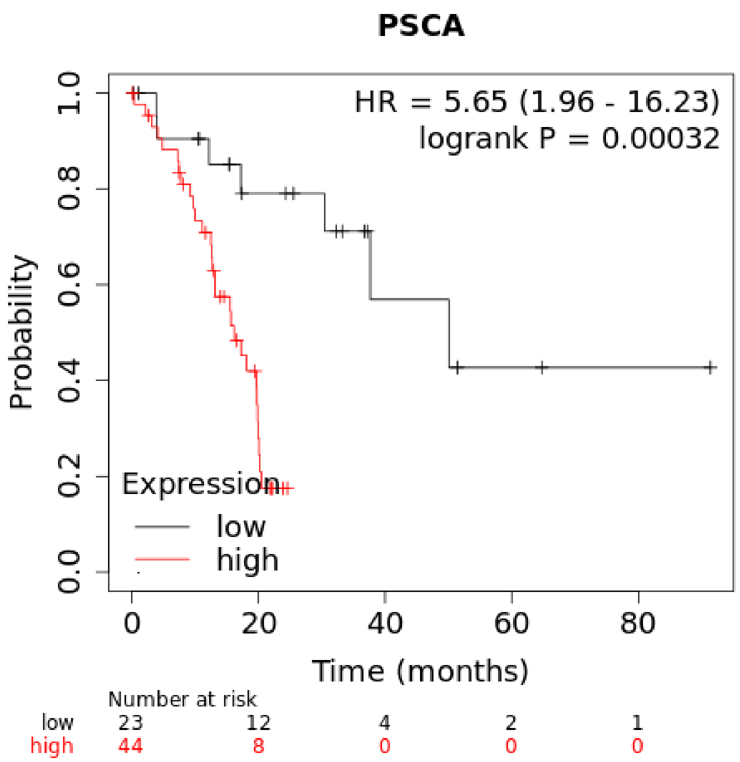

Click here to download the plot in TIFF format

Download plot as a PDF

Download p values vs. cutoff table

Median survival

Low expression cohort (months)	High expression cohort (months)
50.07	16.2

**RNAseq ID:**	LY6K -
**Survival:**	OS
**Auto select best cutoff:**	checked
**Follow up threshold:**	all
**Censore at threshold:**	checked
**Compute median over entire database:**	false
**Cutoff value used in analysis:**	4
**Expression range of the probe:**	0 - 917
**Invert HR values below 1:**	not checked

Restrictions

Tumor type: Pancreatic ductal adenocarcinoma

Restrict analysis to subtypes...

Stage:	all
Gender:	all
Race:	all
Grade:	all
Mutation burden:	all

Restrict analysis based on cellular content...

Basophils:	all

B-cells:	all
CD4+ memory T-cells:	all
CD8+ T-cells:	all
Eosinophils:	all
Macrophages:	all
Mesenchymal stem cells:	all
Natural killer T-cells:	all
Regulatory T-cells:	enriched
Type 1 T-helper cells:	all
Type 2 T-helper cells:	all

Results

P value: 0.2185
FDR: 100%

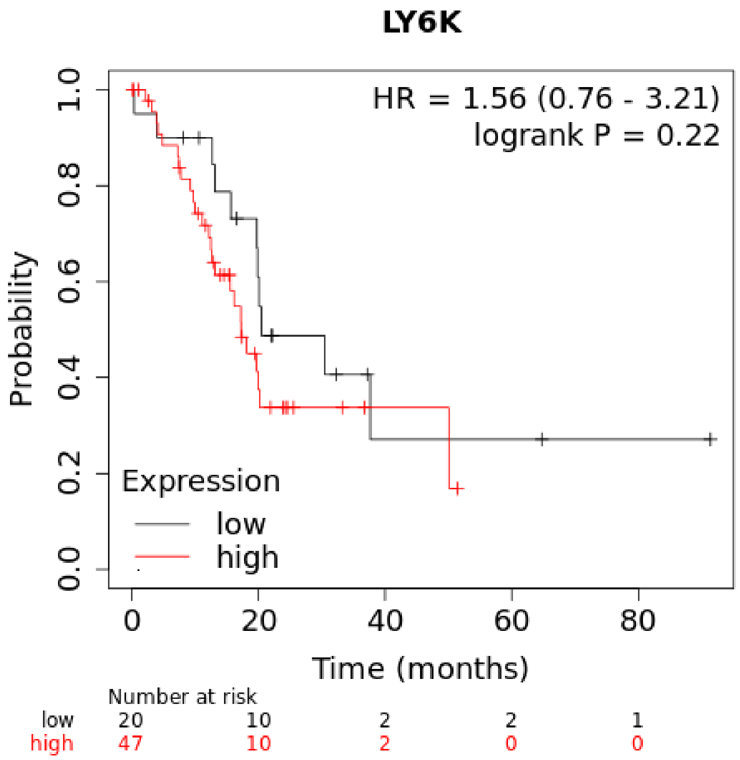

Click here to download the plot in TIFF format

Download plot as a PDF

Download p values vs. cutoff table

Median survival

Low expression cohort (months)	High expression cohort (months)
20.47	17.27

**RNAseq ID:**	SLURP1 -
**Survival:**	OS
**Auto select best cutoff:**	checked
**Follow up threshold:**	all
**Censore at threshold:**	checked
**Compute median over entire database:**	false
**Cutoff value used in analysis:**	1
**Expression range of the probe:**	0 - 42
**Invert HR values below 1:**	not checked

Restrictions

Tumor type: Pancreatic ductal adenocarcinoma

Restrict analysis to subtypes...

Stage:	all
Gender:	all
Race:	all
Grade:	all
Mutation burden:	all

Restrict analysis based on cellular content...

Basophils:	all
B-cells:	all
CD4+ memory T-cells:	all
CD8+ T-cells:	all
Eosinophils:	all
Macrophages:	all
Mesenchymal stem cells:	all
Natural killer T-cells:	all
Regulatory T-cells:	enriched
Type 1 T-helper cells:	all
Type 2 T-helper cells:	all

Results

P value: 7.0e-5
FDR: 1%

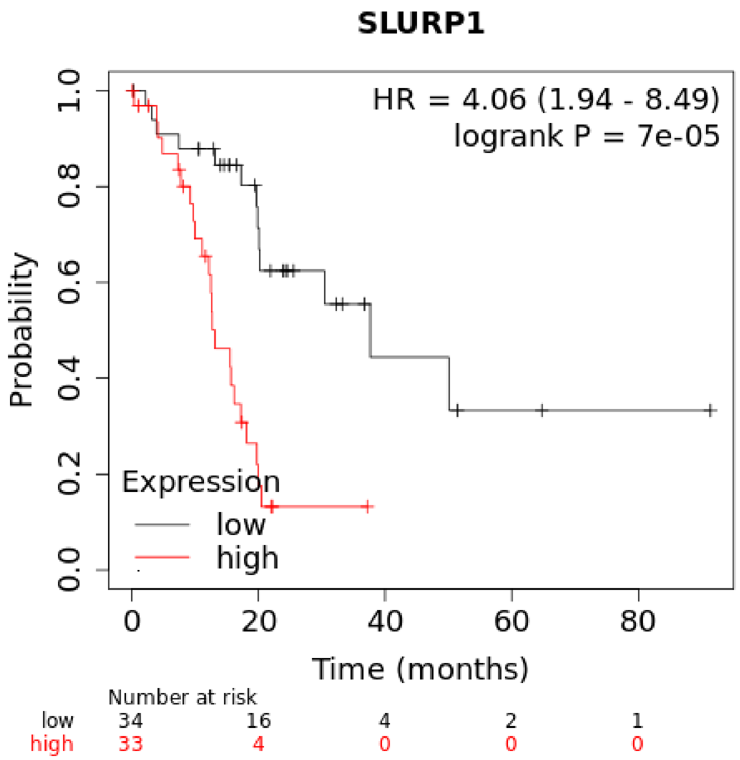

Click here to download the plot in TIFF format

Download plot as a PDF

Download p values vs. cutoff table

Median survival

Low expression cohort (months)	High expression cohort (months)
37.67	13.13

**RNAseq ID:**	LYPD2 -
**Survival:**	OS
**Auto select best cutoff:**	checked
**Follow up threshold:**	all
**Censore at threshold:**	checked
**Compute median over entire database:**	false
**Cutoff value used in analysis:**	5
**Expression range of the probe:**	0 - 4748
**Invert HR values below 1:**	not checked

Restrictions

Tumor type: Pancreatic ductal adenocarcinoma

Restrict analysis to subtypes...

Stage:	all
Gender:	all
Race:	all
Grade:	all
Mutation burden:	all

Restrict analysis based on cellular content...

Basophils:	all
B-cells:	all
CD4+ memory T-cells:	all
CD8+ T-cells:	all
Eosinophils:	all
Macrophages:	all
Mesenchymal stem cells:	all
Natural killer T-cells:	all
Regulatory T-cells:	enriched
Type 1 T-helper cells:	all
Type 2 T-helper cells:	all

Results

**P value:**	0.0022
**FDR:**	20%

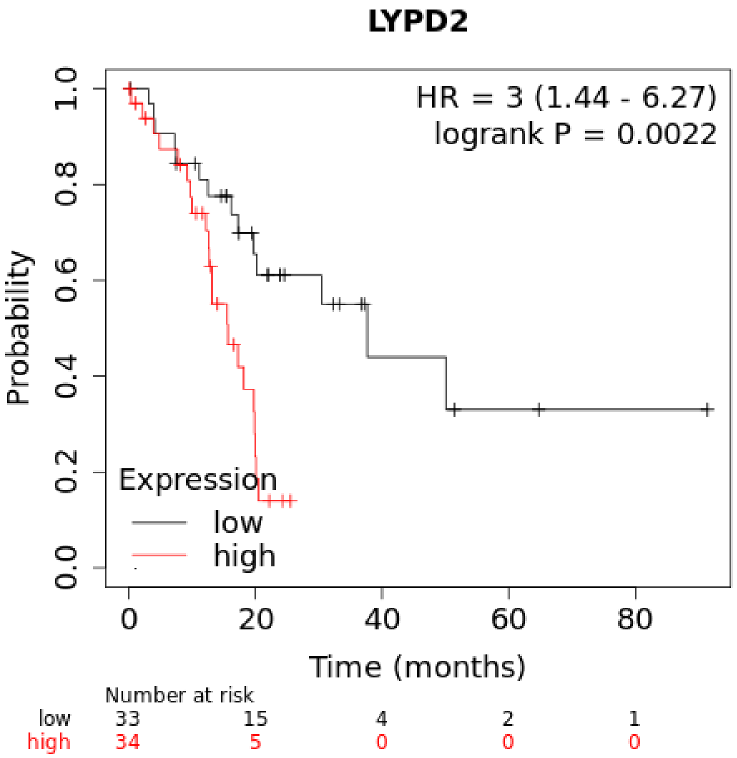

Click here to download the plot in TIFF format

Download plot as a PDF

Download p values vs. cutoff table

Median survival

Low expression cohort (months)	High expression cohort (months)
37.67	15.67

**RNAseq ID:**	LY6D -
**Survival:**	OS
**Auto select best cutoff:**	checked
**Follow up threshold:**	all
**Censore at threshold:**	checked
**Compute median over entire database:**	false
**Cutoff value used in analysis:**	315
**Expression range of the probe:**	0 - 6226
**Invert HR values below 1:**	not checked

Restrictions

Tumor type: Pancreatic ductal adenocarcinoma

Restrict analysis to subtypes...

Stage:	all
Gender:	all
Race:	all
Grade:	all
Mutation burden:	all

Restrict analysis based on cellular content...

Basophils:	all

B-cells:	all
CD4+ memory T-cells:	all
CD8+ T-cells:	all
Eosinophils:	all
Macrophages:	all
Mesenchymal stem cells:	all
Natural killer T-cells:	all
Regulatory T-cells:	enriched
Type 1 T-helper cells:	all
Type 2 T-helper cells:	all

Results

P value: 2.2e-5
FDR: 1%

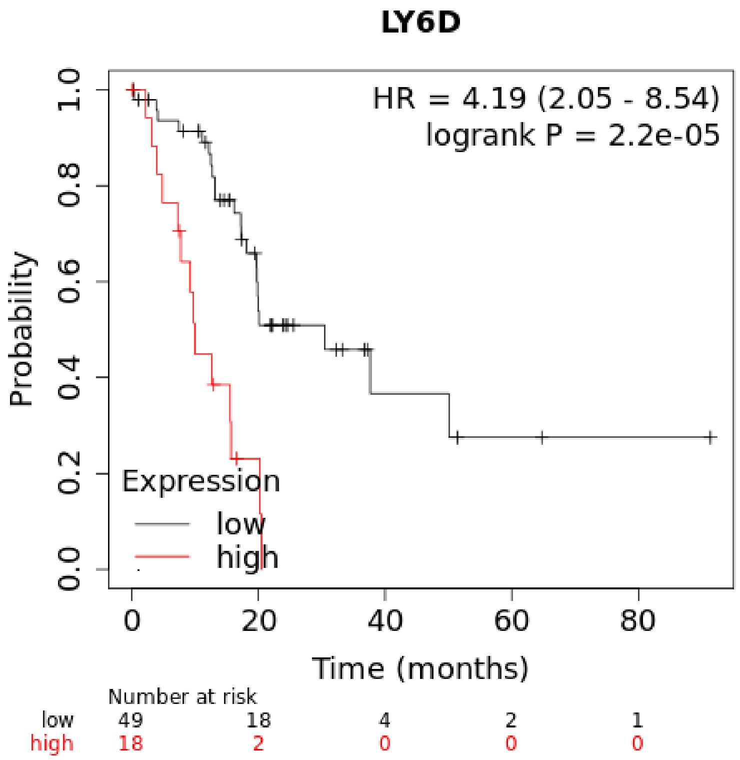

Click here to download the plot in TIFF format

Download plot as a PDF

Download p values vs. cutoff table

Median survival

Low expression cohort (months)	High expression cohort (months)
30.43	9.97

**RNAseq ID:**	GML -
**Survival:**	OS
**Auto select best cutoff:**	checked
**Follow up threshold:**	all
**Censore at threshold:**	checked
**Compute median over entire database:**	false
**Cutoff value used in analysis:**	0
**Expression range of the probe:**	0 - 3
**Invert HR values below 1:**	not checked

Restrictions

Tumor type: Pancreatic ductal adenocarcinoma

Restrict analysis to subtypes...

Stage:	all
Gender:	all
Race:	all
Grade:	all
Mutation burden:	all

Restrict analysis based on cellular content...

Basophils:	all
B-cells:	all
CD4+ memory T-cells:	all
CD8+ T-cells:	all
Eosinophils:	all
Macrophages:	all
Mesenchymal stem cells:	all
Natural killer T-cells:	all
Regulatory T-cells:	enriched
Type 1 T-helper cells:	all
Type 2 T-helper cells:	all

Results

P value: 0.1656
FDR: 100%

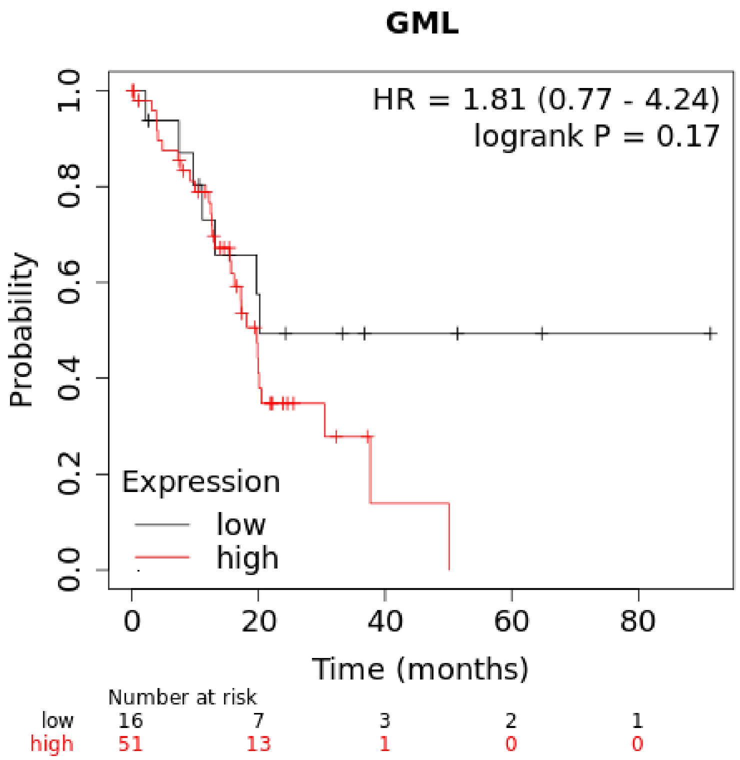

Click here to download the plot in TIFF format

Download plot as a PDF

Download p values vs. cutoff table

Median survival

Low expression cohort (months)	High expression cohort (months)
20.23	19.77

**RNAseq ID:**	LY6E -
**Survival:**	OS
**Auto select best cutoff:**	checked
**Follow up threshold:**	all
**Censore at threshold:**	checked
**Compute median over entire database:**	false
**Cutoff value used in analysis:**	10637
**Expression range of the probe:**	254 - 52613
**Invert HR values below 1:**	not checked

Restrictions

Tumor type: Pancreatic ductal adenocarcinoma

Restrict analysis to subtypes...

Stage:	all
Gender:	all
Race:	all
Grade:	all
Mutation burden:	all

Restrict analysis based on cellular content...

Basophils:	all
B-cells:	all
CD4+ memory T-cells:	all
CD8+ T-cells:	all
Eosinophils:	all
Macrophages:	all
Mesenchymal stem cells:	all
Natural killer T-cells:	all
Regulatory T-cells:	enriched
Type 1 T-helper cells:	all
Type 2 T-helper cells:	all

Results

**P value:**	0.0081
**FDR:**	50%

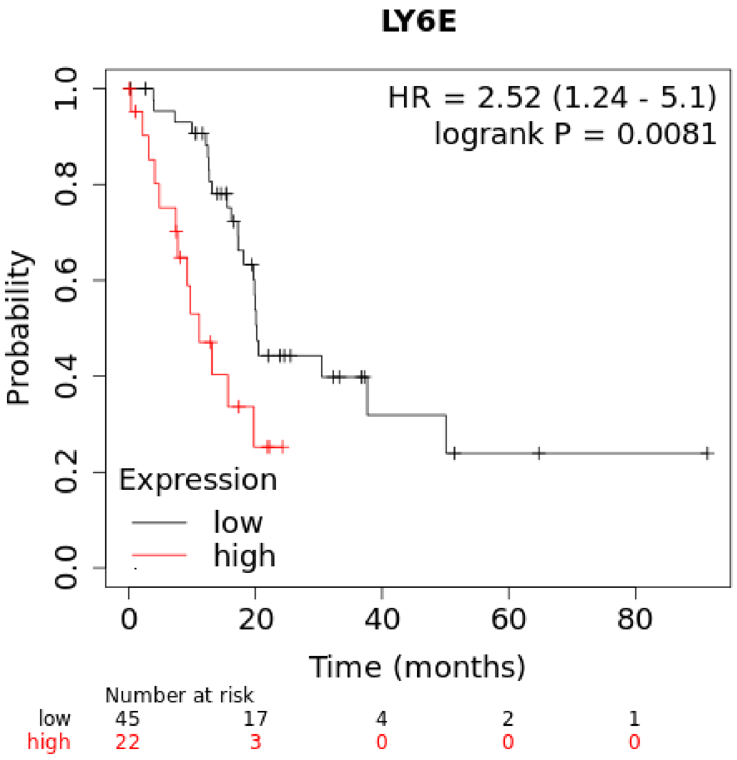

Click here to download the plot in TIFF format

Download plot as a PDF

Download p values vs. cutoff table

Median survival

Low expression cohort (months)	High expression cohort (months)
20.23	11.13

RNAseq ID: LY6L -
Survival: OS
Auto select best cutoff: checked
Follow up threshold: all
Censore at threshold: checked
Compute median over entire database: false
Cutoff value used in analysis: 0
Expression range of the probe: 0 - 2
Invert HR values below 1: not checked

Restrictions

Tumor type: Pancreatic ductal adenocarcinoma

Restrict analysis to subtypes...

Stage: all
Gender: all
Race: all
Grade: all
Mutation burden: all

Restrict analysis based on cellular content...

Basophils: all

B-cells: all
CD4+ memory T-cells: all
CD8+ T-cells: all
Eosinophils: all
Macrophages: all
Mesenchymal stem cells: all
Natural killer T-cells: all
Regulatory T-cells: enriched
Type 1 T-helper cells: all
Type 2 T-helper cells: all

Results

P value: 0.1656
FDR: 100%

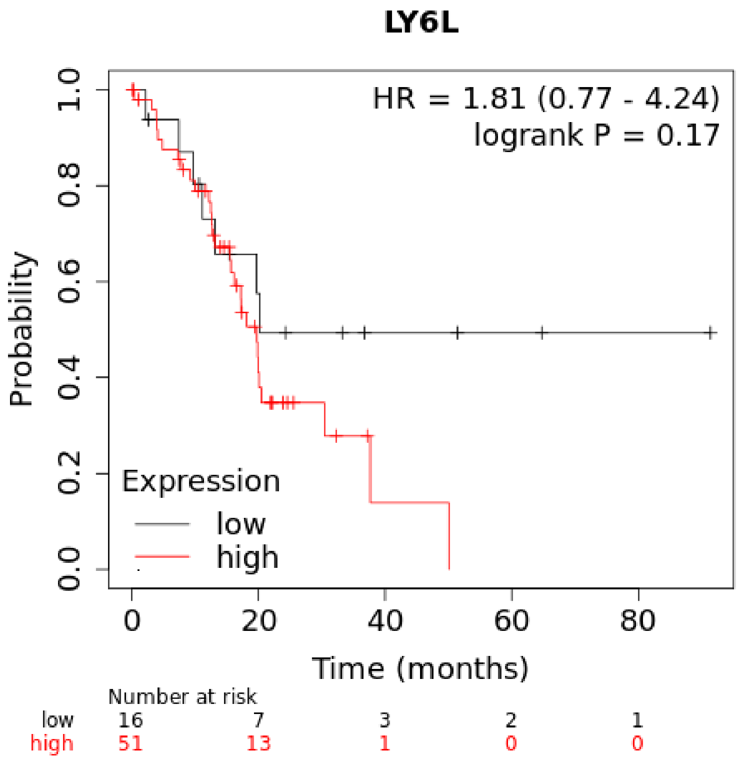

Click here to download the plot in TIFF format

Download plot as a PDF

Download p values vs. cutoff table

Median survival

Low expression cohort (months)	High expression cohort (months)
20.23	19.77

RNAseq ID: LY6H -
Survival: OS
Auto select best cutoff: checked
Follow up threshold: all
Censore at threshold: checked
Compute median over entire database: false
Cutoff value used in analysis: 39
Expression range of the probe: 2 - 6634
Invert HR values below 1: not checked

Restrictions

Tumor type: Pancreatic ductal adenocarcinoma

Restrict analysis to subtypes...

Stage:	all
Gender:	all
Race:	all
Grade:	all
Mutation burden:	all

Restrict analysis based on cellular content...

Basophils:	all
B-cells:	all
CD4+ memory T-cells:	all
CD8+ T-cells:	all
Eosinophils:	all
Macrophages:	all
Mesenchymal stem cells:	all
Natural killer T-cells:	all
Regulatory T-cells:	enriched
Type 1 T-helper cells:	all
Type 2 T-helper cells:	all

Results

P value: 0.0024
FDR: 20%

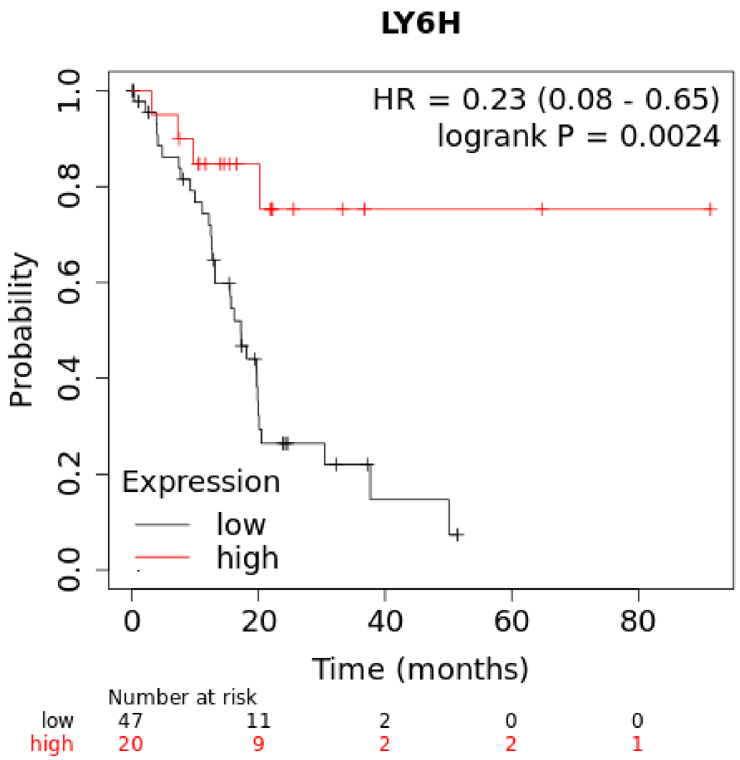

Click here to download the plot in TIFF format

Download plot as a PDF

Download p values vs. cutoff table

Median survival

Low expression cohort (months)	High expression cohort (months)
NA	NA

**RNAseq ID:**	GPIHBP1 -
**Survival:**	OS
**Auto select best cutoff:**	checked
**Follow up threshold:**	all
**Censore at threshold:**	checked
**Compute median over entire database:**	false
**Cutoff value used in analysis:**	26
**Expression range of the probe:**	5 - 323
**Invert HR values below 1:**	not checked

Restrictions

Tumor type: Pancreatic ductal adenocarcinoma

Restrict analysis to subtypes...

Stage:	all
Gender:	all
Race:	all
Grade:	all
Mutation burden:	all

Restrict analysis based on cellular content...

Basophils:	all
B-cells:	all
CD4+ memory T-cells:	all
CD8+ T-cells:	all
Eosinophils:	all
Macrophages:	all
Mesenchymal stem cells:	all
Natural killer T-cells:	all
Regulatory T-cells:	enriched
Type 1 T-helper cells:	all
Type 2 T-helper cells:	all

Results

**P value:**	0.0321
**FDR:**	over 50%

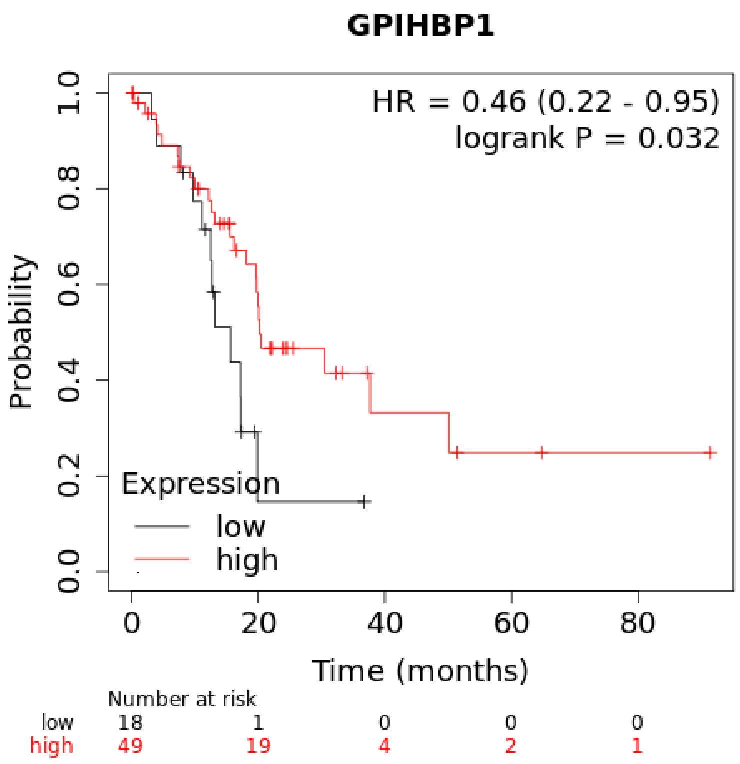

Click here to download the plot in TIFF format

Download plot as a PDF

Download p values vs. cutoff table

Median survival

Low expression cohort (months)	High expression cohort (months)
15.67	20.23

**RNAseq ID:**	LYPD4 -
**Survival:**	OS
**Auto select best cutoff:**	checked
**Follow up threshold:**	all
**Censore at threshold:**	checked
**Compute median over entire database:**	false
**Cutoff value used in analysis:**	0
**Expression range of the probe:**	0 - 11
**Invert HR values below 1:**	not checked

Restrictions

Tumor type: Pancreatic ductal adenocarcinoma

Restrict analysis to subtypes...

Stage:	all
Gender:	all
Race:	all
Grade:	all
Mutation burden:	all

Restrict analysis based on cellular content...

Basophils:	all

B-cells:	all
CD4+ memory T-cells:	all
CD8+ T-cells:	all
Eosinophils:	all
Macrophages:	all
Mesenchymal stem cells:	all
Natural killer T-cells:	all
Regulatory T-cells:	enriched
Type 1 T-helper cells:	all
Type 2 T-helper cells:	all

Results

P value: 0.0662
FDR: 100%

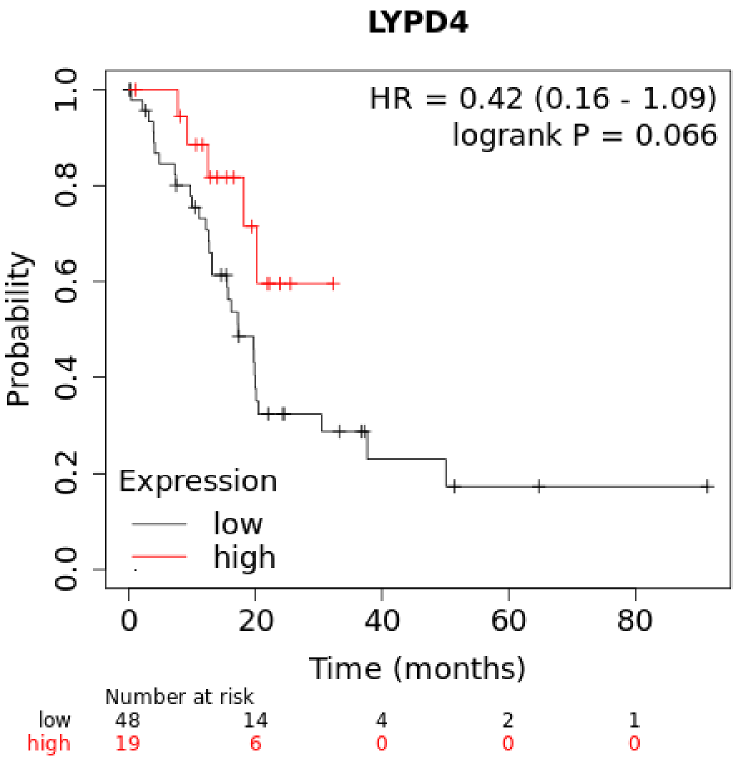

Click here to download the plot in TIFF format

Download plot as a PDF

Download p values vs. cutoff table

Upper quartile survival

Low expression cohort (months)	High expression cohort (months)
11.13	18.17

**RNAseq ID:**	CD177 -
**Survival:**	OS
**Auto select best cutoff:**	checked
**Follow up threshold:**	all
**Censore at threshold:**	checked
**Compute median over entire database:**	false
**Cutoff value used in analysis:**	21
**Expression range of the probe:**	0 - 3766
**Invert HR values below 1:**	not checked

Restrictions

Tumor type: Pancreatic ductal adenocarcinoma

Restrict analysis to subtypes...

Stage:	all
Gender:	all
Race:	all
Grade:	all
Mutation burden:	all

Restrict analysis based on cellular content...

Basophils:	all
B-cells:	all
CD4+ memory T-cells:	all
CD8+ T-cells:	all
Eosinophils:	all
Macrophages:	all
Mesenchymal stem cells:	all
Natural killer T-cells:	all
Regulatory T-cells:	enriched
Type 1 T-helper cells:	all
Type 2 T-helper cells:	all

Results

P value: 0.0085
FDR: 50%

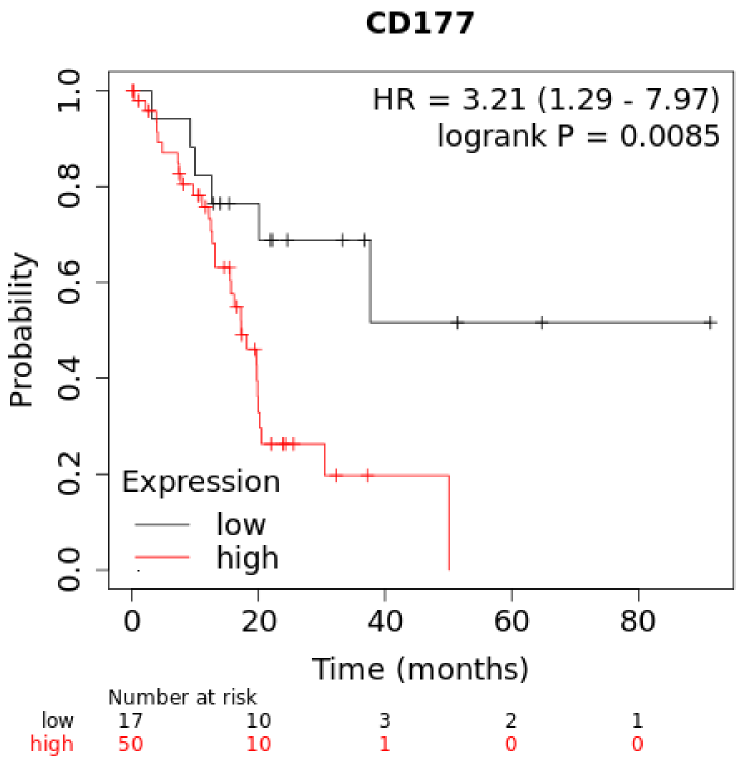

Click here to download the plot in TIFF format

Download plot as a PDF

Download p values vs. cutoff table

Upper quartile survival

Low expression cohort (months)	High expression cohort (months)
20.1	12.2

**RNAseq ID:**	TEX101 -
**Survival:**	OS
**Auto select best cutoff:**	checked
**Follow up threshold:**	all
**Censore at threshold:**	checked
**Compute median over entire database:**	false
**Cutoff value used in analysis:**	2
**Expression range of the probe:**	0 - 20
**Invert HR values below 1:**	not checked

Restrictions

Tumor type: Pancreatic ductal adenocarcinoma

Restrict analysis to subtypes...

Stage:	all
Gender:	all
Race:	all
Grade:	all
Mutation burden:	all

Restrict analysis based on cellular content...

Basophils:	all
B-cells:	all
CD4+ memory T-cells:	all
CD8+ T-cells:	all
Eosinophils:	all
Macrophages:	all
Mesenchymal stem cells:	all
Natural killer T-cells:	all
Regulatory T-cells:	enriched
Type 1 T-helper cells:	all
Type 2 T-helper cells:	all

Results

**P value:**	0.0484
**FDR:**	over 50%

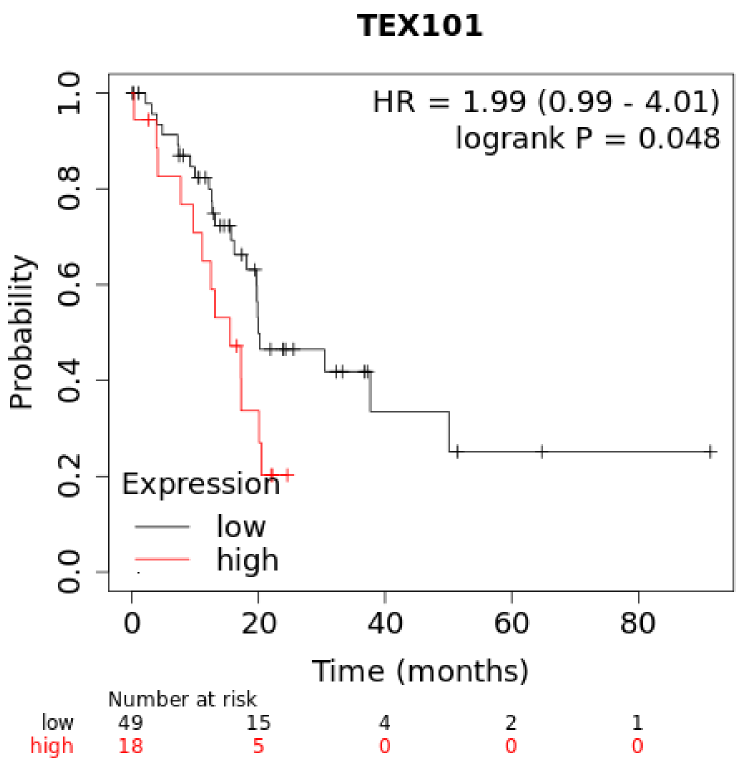

Click here to download the plot in TIFF format

Download plot as a PDF

Download p values vs. cutoff table

Median survival

Low expression cohort (months)	High expression cohort (months)
19.93	15.57

**RNAseq ID:**	LYPD3 -
**Survival:**	OS
**Auto select best cutoff:**	checked
**Follow up threshold:**	all
**Censore at threshold:**	checked
**Compute median over entire database:**	false
**Cutoff value used in analysis:**	95
**Expression range of the probe:**	16 - 7684
**Invert HR values below 1:**	not checked

Restrictions

Tumor type: Pancreatic ductal adenocarcinoma

Restrict analysis to subtypes...

Stage:	all
Gender:	all
Race:	all
Grade:	all
Mutation burden:	all

Restrict analysis based on cellular content...

Basophils:	all

B-cells:	all
CD4+ memory T-cells:	all
CD8+ T-cells:	all
Eosinophils:	all
Macrophages:	all
Mesenchymal stem cells:	all
Natural killer T-cells:	all
Regulatory T-cells:	enriched
Type 1 T-helper cells:	all
Type 2 T-helper cells:	all

Results

P value: 0.0053
FDR: 50%

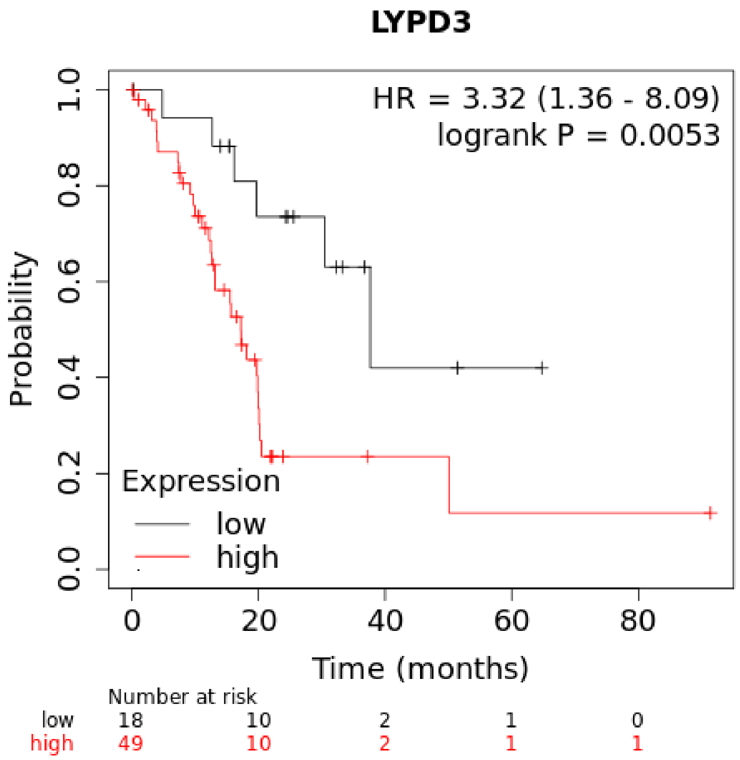

Click here to download the plot in TIFF format

Download plot as a PDF

Download p values vs. cutoff table

Median survival

Low expression cohort (months)	High expression cohort (months)
37.67	17.23

**RNAseq ID:**	PINLYP -
**Survival:**	OS
**Auto select best cutoff:**	checked
**Follow up threshold:**	all
**Censore at threshold:**	checked
**Compute median over entire database:**	false
**Cutoff value used in analysis:**	71
**Expression range of the probe:**	13 - 198
**Invert HR values below 1:**	not checked

Restrictions

Tumor type: Pancreatic ductal adenocarcinoma

Restrict analysis to subtypes...

Stage:	all
Gender:	all
Race:	all
Grade:	all
Mutation burden:	all

Restrict analysis based on cellular content...

Basophils:	all
B-cells:	all
CD4+ memory T-cells:	all
CD8+ T-cells:	all
Eosinophils:	all
Macrophages:	all
Mesenchymal stem cells:	all
Natural killer T-cells:	all
Regulatory T-cells:	enriched
Type 1 T-helper cells:	all
Type 2 T-helper cells:	all

Results

P value: 0.0929
FDR: 100%

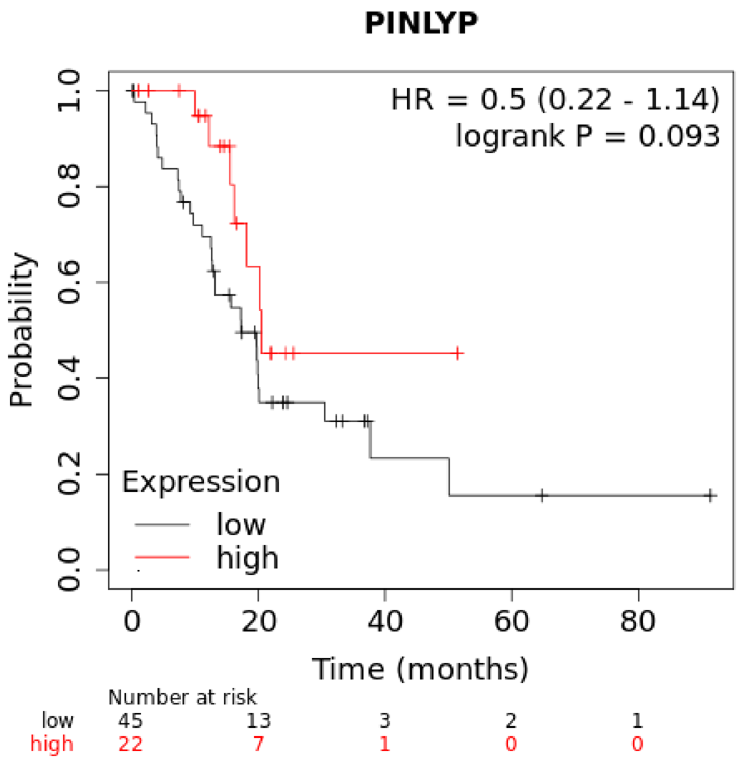

Click here to download the plot in TIFF format

Download plot as a PDF

Download p values vs. cutoff table

Median survival

Low expression cohort (months)	High expression cohort (months)
17.27	20.47

**RNAseq ID:**	PLAUR -
**Survival:**	OS
**Auto select best cutoff:**	checked
**Follow up threshold:**	all
**Censore at threshold:**	checked
**Compute median over entire database:**	false
**Cutoff value used in analysis:**	1655
**Expression range of the probe:**	47 - 18314
**Invert HR values below 1:**	not checked

Restrictions

Tumor type: Pancreatic ductal adenocarcinoma

Restrict analysis to subtypes...

Stage:	all
Gender:	all
Race:	all
Grade:	all
Mutation burden:	all

Restrict analysis based on cellular content...

Basophils:	all
B-cells:	all
CD4+ memory T-cells:	all
CD8+ T-cells:	all
Eosinophils:	all
Macrophages:	all
Mesenchymal stem cells:	all
Natural killer T-cells:	all
Regulatory T-cells:	enriched
Type 1 T-helper cells:	all
Type 2 T-helper cells:	all

Results

P value: 0.0039
FDR: 50%

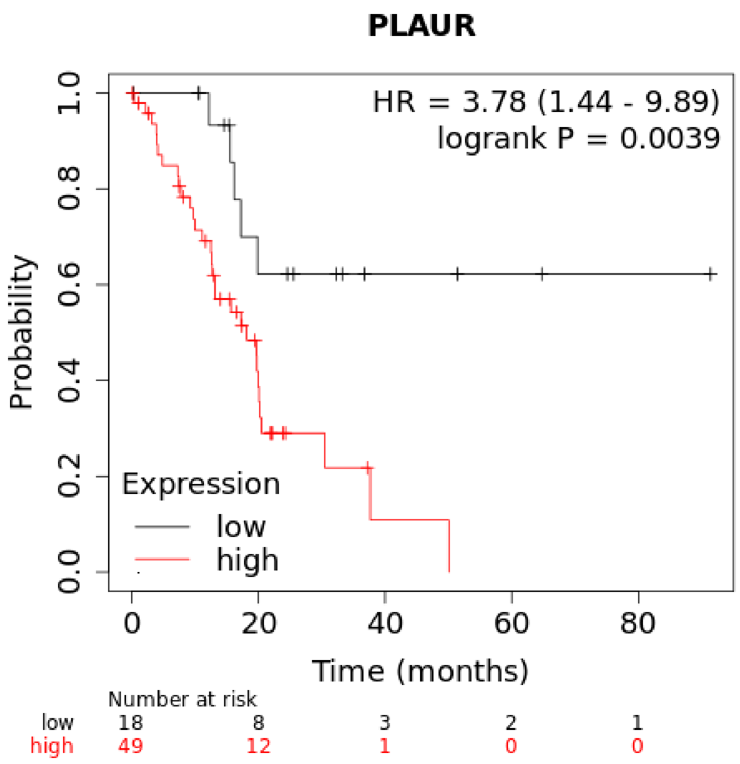

Click here to download the plot in TIFF format

Download plot as a PDF

Download p values vs. cutoff table

Upper quartile survival

Low expression cohort (months)	High expression cohort (months)
17.23	9.77

**RNAseq ID:**	LYPD5 -
**Survival:**	OS
**Auto select best cutoff:**	checked
**Follow up threshold:**	all
**Censore at threshold:**	checked
**Compute median over entire database:**	false
**Cutoff value used in analysis:**	58
**Expression range of the probe:**	1 - 245
**Invert HR values below 1:**	not checked

Restrictions

Tumor type: Pancreatic ductal adenocarcinoma

Restrict analysis to subtypes...

Stage:	all
Gender:	all
Race:	all
Grade:	all
Mutation burden:	all

Restrict analysis based on cellular content...

Basophils:	all

B-cells:	all
CD4+ memory T-cells:	all
CD8+ T-cells:	all
Eosinophils:	all
Macrophages:	all
Mesenchymal stem cells:	all
Natural killer T-cells:	all
Regulatory T-cells:	enriched
Type 1 T-helper cells:	all
Type 2 T-helper cells:	all

Results

P value: 0.0134
FDR: over 50%

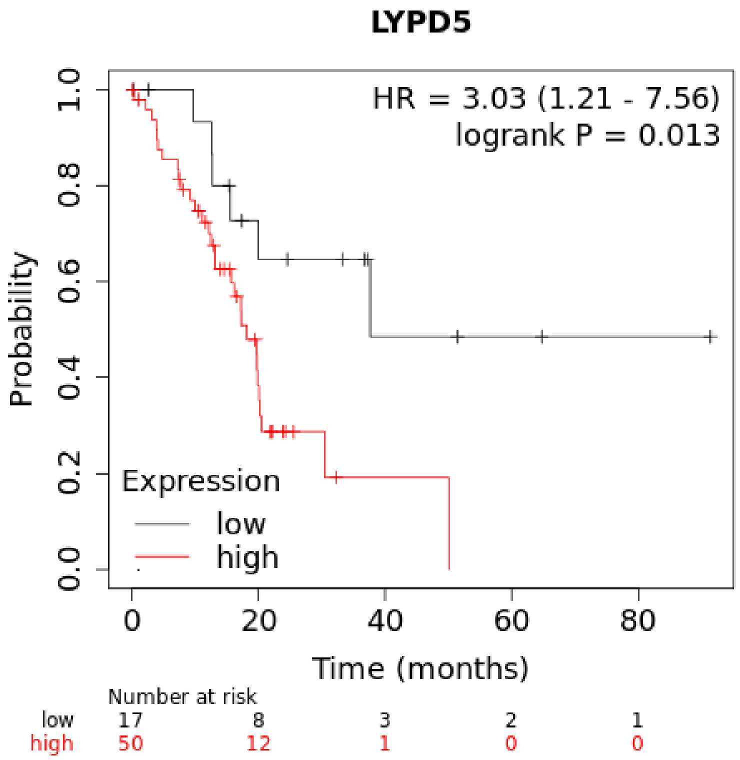

Click here to download the plot in TIFF format

Download plot as a PDF

Download p values vs. cutoff table

Median survival

Low expression cohort (months)	High expression cohort (months)
37.67	18.17

**RNAseq ID:**	SPACA4 -
**Survival:**	OS
**Auto select best cutoff:**	checked
**Follow up threshold:**	all
**Censore at threshold:**	checked
**Compute median over entire database:**	false
**Cutoff value used in analysis:**	23
**Expression range of the probe:**	1 - 204
**Invert HR values below 1:**	not checked

Restrictions

Tumor type: Pancreatic ductal adenocarcinoma

Restrict analysis to subtypes...

Stage:	all
Gender:	all
Race:	all
Grade:	all
Mutation burden:	all

Restrict analysis based on cellular content...

Basophils:	all
B-cells:	all
CD4+ memory T-cells:	all
CD8+ T-cells:	all
Eosinophils:	all
Macrophages:	all
Mesenchymal stem cells:	all
Natural killer T-cells:	all
Regulatory T-cells:	enriched
Type 1 T-helper cells:	all
Type 2 T-helper cells:	all

Results

P value: 0.3551
FDR: 100%

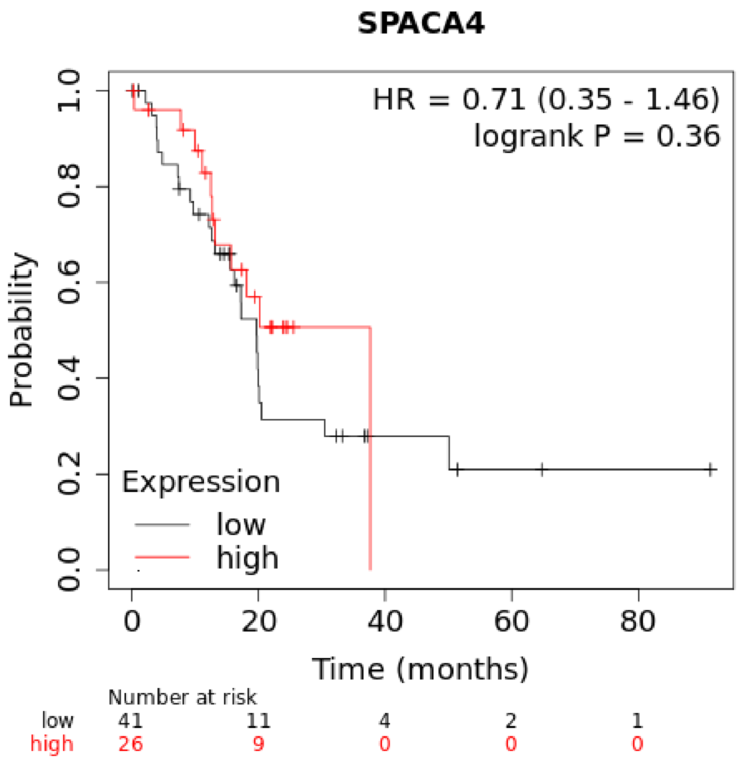

Click here to download the plot in TIFF format

Download plot as a PDF

Download p values vs. cutoff table

Median survival

Low expression cohort (months)	High expression cohort (months)
19.73	37.67

**RNAseq ID:**	ACRV1 -
**Survival:**	OS
**Auto select best cutoff:**	checked
**Follow up threshold:**	all
**Censore at threshold:**	checked
**Compute median over entire database:**	false
**Cutoff value used in analysis:**	3
**Expression range of the probe:**	0 - 26
**Invert HR values below 1:**	not checked

Restrictions

Tumor type: Pancreatic ductal adenocarcinoma

Restrict analysis to subtypes...

Stage:	all
Gender:	all
Race:	all
Grade:	all
Mutation burden:	all

Restrict analysis based on cellular content...

Basophils:	all
B-cells:	all
CD4+ memory T-cells:	all
CD8+ T-cells:	all
Eosinophils:	all
Macrophages:	all
Mesenchymal stem cells:	all
Natural killer T-cells:	all
Regulatory T-cells:	enriched
Type 1 T-helper cells:	all
Type 2 T-helper cells:	all

Results

**P value:**	0.1166
**FDR:**	100%

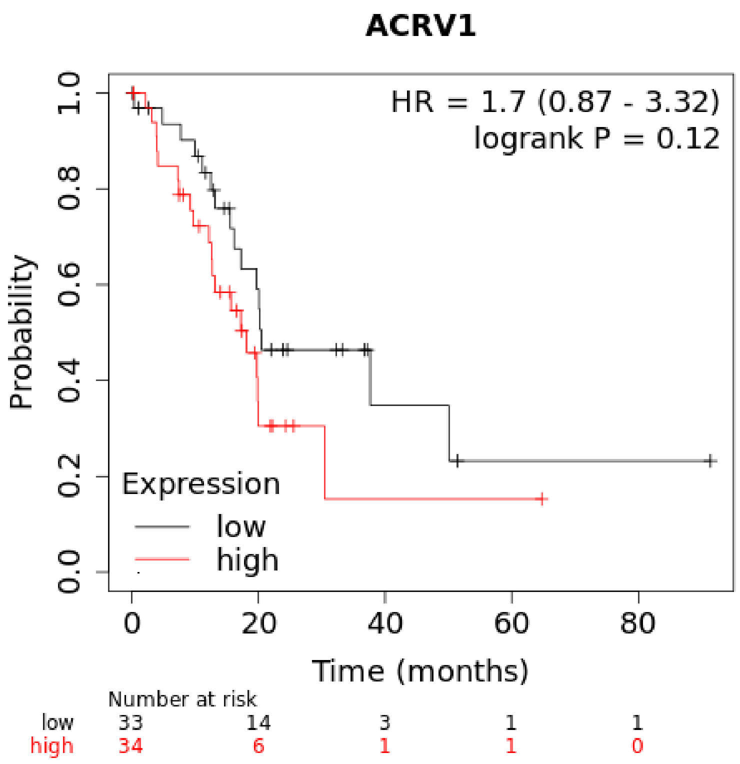

Click here to download the plot in TIFF format

Download plot as a PDF

Download p values vs. cutoff table

Median survival

Low expression cohort (months)	High expression cohort (months)
20.47	18.17

**RNAseq ID:**	PATE1 -
**Survival:**	OS
**Auto select best cutoff:**	checked
**Follow up threshold:**	all
**Censore at threshold:**	checked
**Compute median over entire database:**	false
**Cutoff value used in analysis:**	0
**Expression range of the probe:**	0 - 1
**Invert HR values below 1:**	not checked

Restrictions

Tumor type: Pancreatic ductal adenocarcinoma

Restrict analysis to subtypes...

Stage:	all
Gender:	all
Race:	all
Grade:	all
Mutation burden:	all

Restrict analysis based on cellular content...

Basophils:	all

B-cells:	all
CD4+ memory T-cells:	all
CD8+ T-cells:	all
Eosinophils:	all
Macrophages:	all
Mesenchymal stem cells:	all
Natural killer T-cells:	all
Regulatory T-cells:	enriched
Type 1 T-helper cells:	all
Type 2 T-helper cells:	all

Results

P value: 0.0369
FDR: over 50%

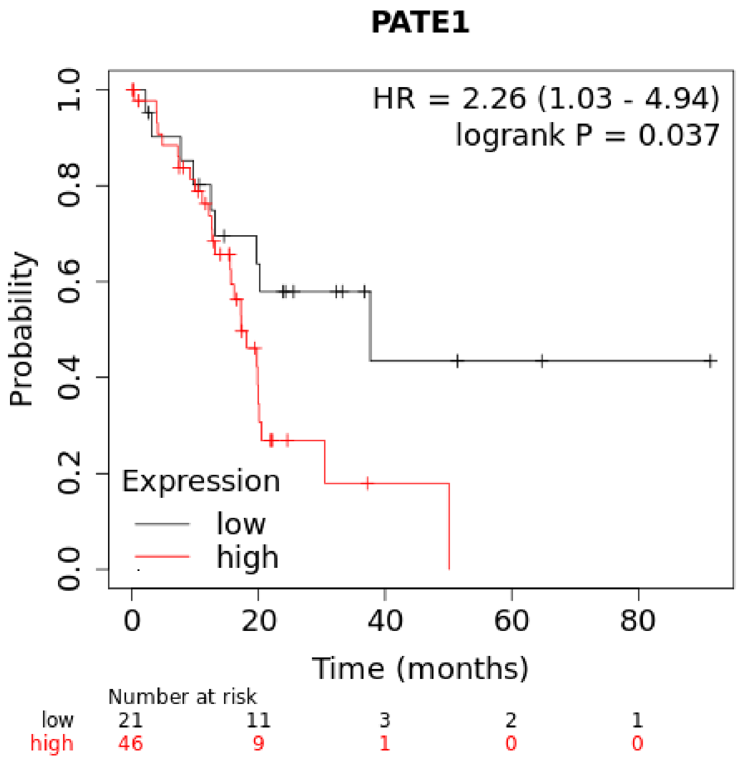

Click here to download the plot in TIFF format

Download plot as a PDF

Download p values vs. cutoff table

Median survival

Low expression cohort (months)	High expression cohort (months)
37.67	17.27

**RNAseq ID:**	PATE2 -
**Survival:**	OS
**Auto select best cutoff:**	checked
**Follow up threshold:**	all
**Censore at threshold:**	checked
**Compute median over entire database:**	false
**Cutoff value used in analysis:**	1
**Expression range of the probe:**	0 - 3
**Invert HR values below 1:**	not checked

Restrictions

Tumor type: Pancreatic ductal adenocarcinoma

Restrict analysis to subtypes...

Stage: all
Gender: all
Race: all
Grade: all
Mutation burden: all

Restrict analysis based on cellular content...

Basophils: all
B-cells: all
CD4+ memory T-cells: all
CD8+ T-cells: all
Eosinophils: all
Macrophages: all
Mesenchymal stem cells: all
Natural killer T-cells: all
Regulatory T-cells: enriched
Type 1 T-helper cells: all
Type 2 T-helper cells: all

Results

P value: 0.028
FDR: over 50%

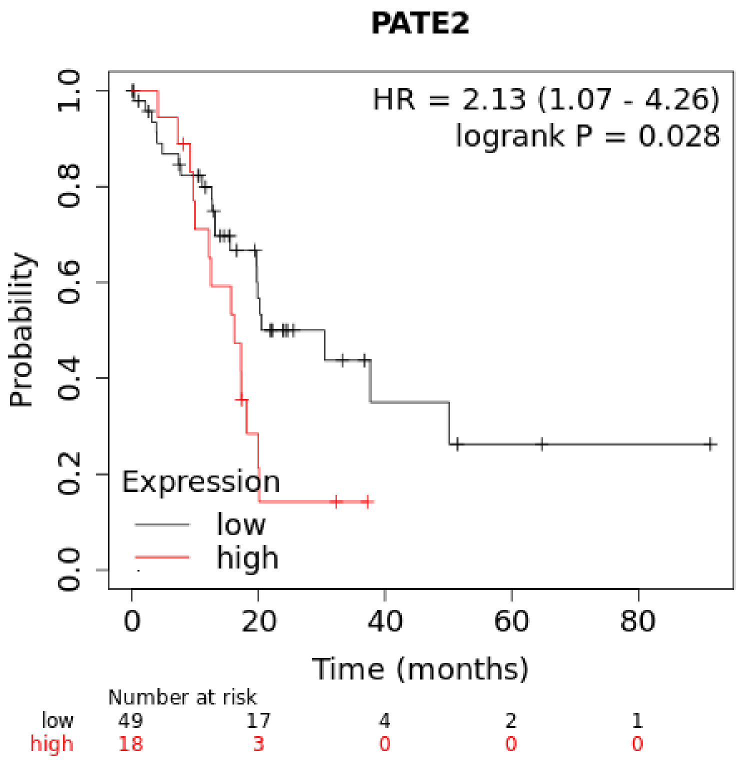

[Click here to download the plot in TIFF format]

[Download plot as a PDF]

[Download p values vs. cutoff table]

Median survival

Low expression cohort (months)	High expression cohort (months)
30.43	16.2

**RNAseq ID:**	PATE3 -
**Survival:**	OS
**Auto select best cutoff:**	checked
**Follow up threshold:**	all
**Censore at threshold:**	checked
**Compute median over entire database:**	false
**Cutoff value used in analysis:**	0
**Expression range of the probe:**	0 - 1
**Invert HR values below 1:**	not checked

Restrictions

Tumor type: Pancreatic ductal adenocarcinoma

Restrict analysis to subtypes...

Stage:	all
Gender:	all
Race:	all
Grade:	all
Mutation burden:	all

Restrict analysis based on cellular content...

Basophils:	all
B-cells:	all
CD4+ memory T-cells:	all
CD8+ T-cells:	all
Eosinophils:	all
Macrophages:	all
Mesenchymal stem cells:	all
Natural killer T-cells:	all
Regulatory T-cells:	enriched
Type 1 T-helper cells:	all
Type 2 T-helper cells:	all

Results

**P value:**	0.1647
**FDR:**	100%

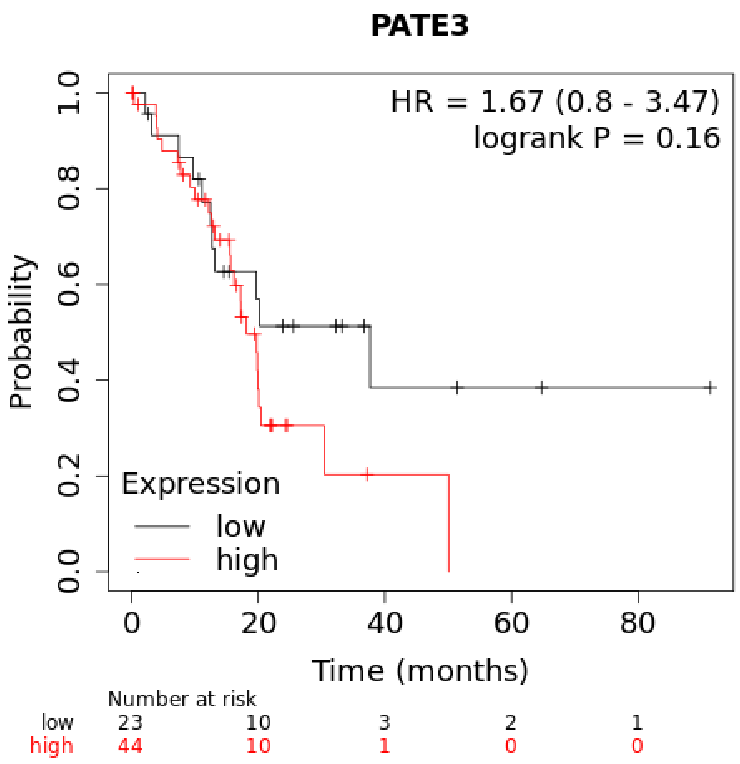

Click here to download the plot in TIFF format

Download plot as a PDF

Download p values vs. cutoff table

Median survival

Low expression cohort (months)	High expression cohort (months)
37.67	18.17

**RNAseq ID:**	PATE4 -
**Survival:**	OS
**Auto select best cutoff:**	checked
**Follow up threshold:**	all
**Censore at threshold:**	checked
**Compute median over entire database:**	false
**Cutoff value used in analysis:**	0
**Expression range of the probe:**	0 - 2
**Invert HR values below 1:**	not checked

Restrictions

Tumor type: Pancreatic ductal adenocarcinoma

Restrict analysis to subtypes...

Stage:	all
Gender:	all
Race:	all
Grade:	all
Mutation burden:	all

Restrict analysis based on cellular content...

Basophils:	all

B-cells:	all
CD4+ memory T-cells:	all
CD8+ T-cells:	all
Eosinophils:	all
Macrophages:	all
Mesenchymal stem cells:	all
Natural killer T-cells:	all
Regulatory T-cells:	enriched
Type 1 T-helper cells:	all
Type 2 T-helper cells:	all

Results

P value: 0.2102
FDR: 100%

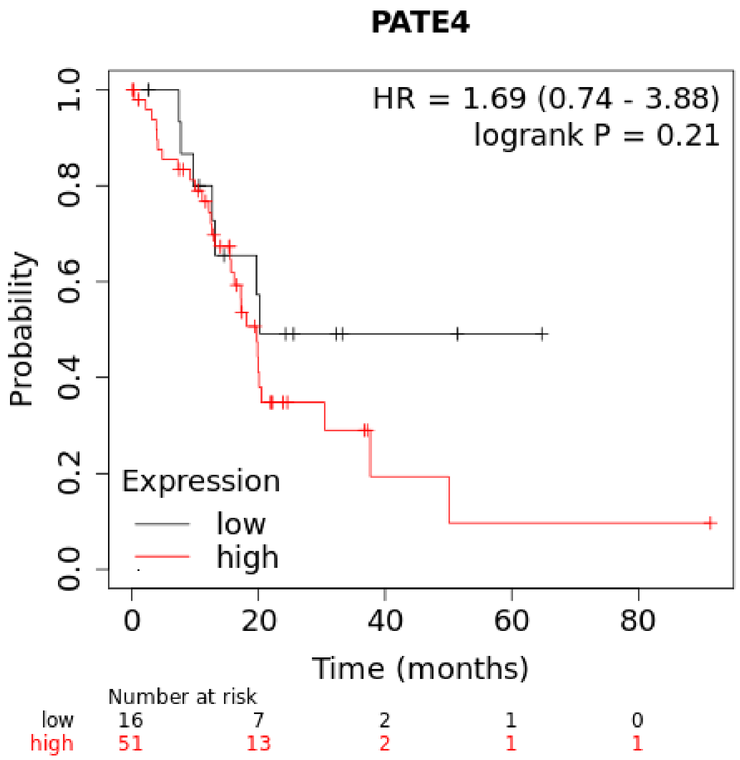

Click here to download the plot in TIFF format

Download plot as a PDF

Download p values vs. cutoff table

Median survival

Low expression cohort (months)	High expression cohort (months)
20.23	19.77

**RNAseq ID:**	CD59 -
**Survival:**	OS
**Auto select best cutoff:**	checked
**Follow up threshold:**	all
**Censore at threshold:**	checked
**Compute median over entire database:**	false
**Cutoff value used in analysis:**	17604
**Expression range of the probe:**	5969 - 30352
**Invert HR values below 1:**	not checked

Restrictions

Tumor type: Pancreatic ductal adenocarcinoma

Restrict analysis to subtypes...

Stage:	all
Gender:	all
Race:	all
Grade:	all
Mutation burden:	all

Restrict analysis based on cellular content...

Basophils:	all
B-cells:	all
CD4+ memory T-cells:	all
CD8+ T-cells:	all
Eosinophils:	all
Macrophages:	all
Mesenchymal stem cells:	all
Natural killer T-cells:	all
Regulatory T-cells:	enriched
Type 1 T-helper cells:	all
Type 2 T-helper cells:	all

Results

P value: 0.0734
FDR: 100%

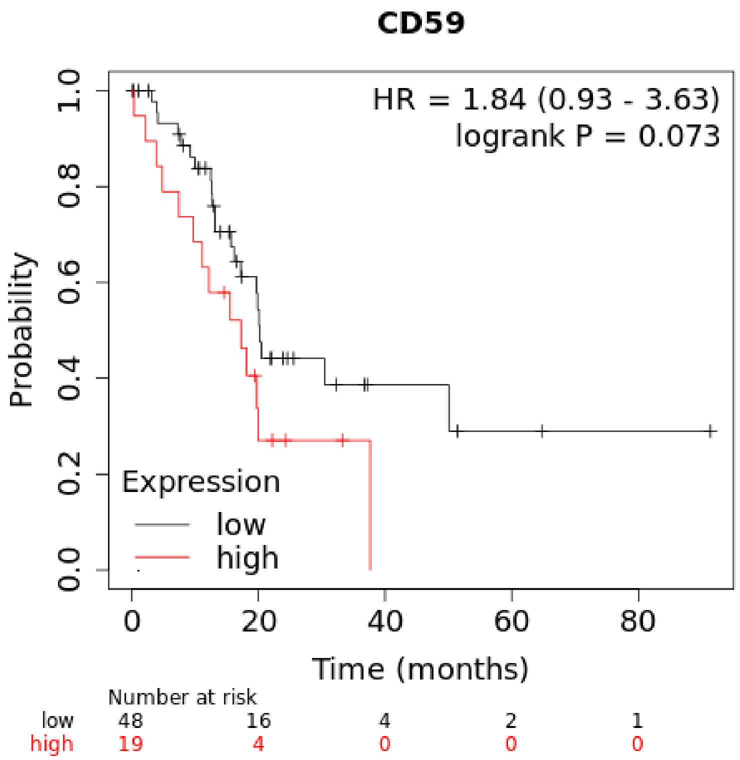

Click here to download the plot in TIFF format

Download plot as a PDF

Download p values vs. cutoff table

Median survival

Low expression cohort (months)	High expression cohort (months)
20.23	17.27

**RNAseq ID:**	LY6G6C -
**Survival:**	OS
**Auto select best cutoff:**	checked
**Follow up threshold:**	all
**Censore at threshold:**	checked
**Compute median over entire database:**	false
**Cutoff value used in analysis:**	3
**Expression range of the probe:**	0 - 140
**Invert HR values below 1:**	not checked

Restrictions

Tumor type: Pancreatic ductal adenocarcinoma

Restrict analysis to subtypes...

Stage:	all
Gender:	all
Race:	all
Grade:	all
Mutation burden:	all

Restrict analysis based on cellular content...

Basophils:	all
B-cells:	all
CD4+ memory T-cells:	all
CD8+ T-cells:	all
Eosinophils:	all
Macrophages:	all
Mesenchymal stem cells:	all
Natural killer T-cells:	all
Regulatory T-cells:	enriched
Type 1 T-helper cells:	all
Type 2 T-helper cells:	all

Results

**P value:**	0.0021
**FDR:**	20%

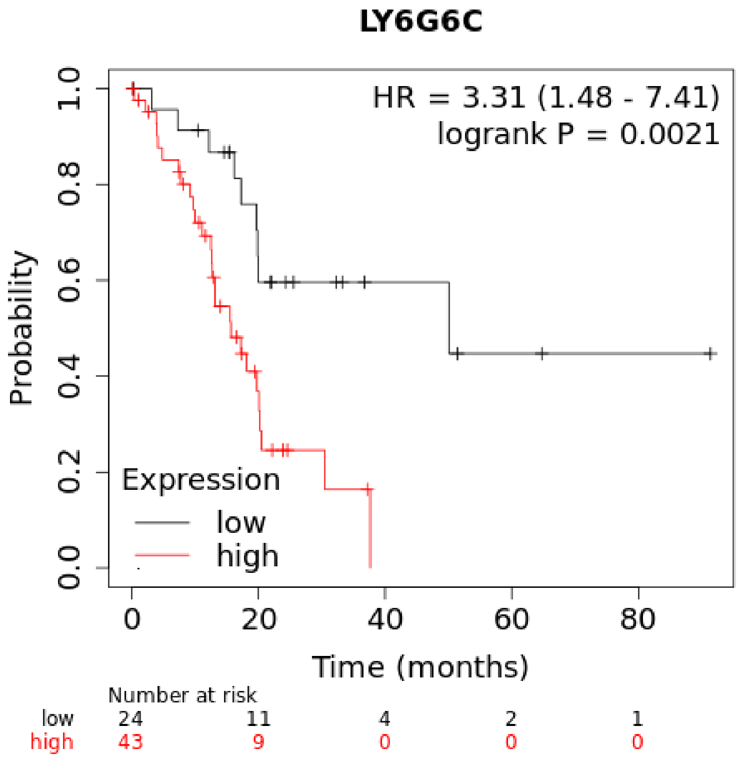

Click here to download the plot in TIFF format

Download plot as a PDF

Download p values vs. cutoff table

Median survival

Low expression cohort (months)	High expression cohort (months)
50.07	15.67

**RNAseq ID:**	LY6G6D -
**Survival:**	OS
**Auto select best cutoff:**	checked
**Follow up threshold:**	all
**Censore at threshold:**	checked
**Compute median over entire database:**	false
**Cutoff value used in analysis:**	0
**Expression range of the probe:**	0 - 1
**Invert HR values below 1:**	not checked

Restrictions

Tumor type: Pancreatic ductal adenocarcinoma

Restrict analysis to subtypes...

Stage:	all
Gender:	all
Race:	all
Grade:	all
Mutation burden:	all

Restrict analysis based on cellular content...

Basophils: all

B-cells:	all
CD4+ memory T-cells:	all
CD8+ T-cells:	all
Eosinophils:	all
Macrophages:	all
Mesenchymal stem cells:	all
Natural killer T-cells:	all
Regulatory T-cells:	enriched
Type 1 T-helper cells:	all
Type 2 T-helper cells:	all

Results

P value: 0.0265
FDR: over 50%

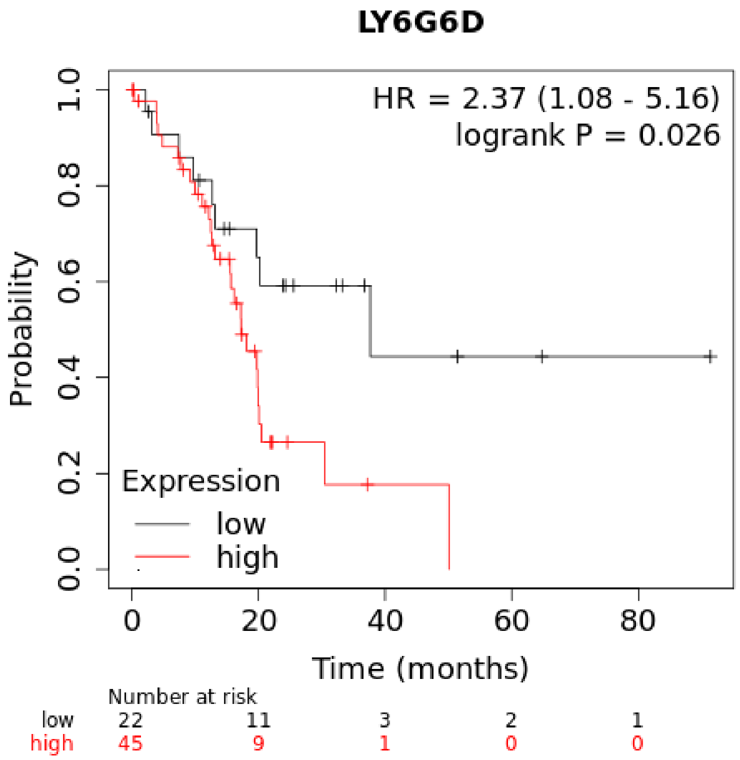

Click here to download the plot in TIFF format

Download plot as a PDF

Download p values vs. cutoff table

Median survival

Low expression cohort (months)	High expression cohort (months)
37.67	17.27

**RNAseq ID:**	LY6G6F -
**Survival:**	OS
**Auto select best cutoff:**	checked
**Follow up threshold:**	all
**Censore at threshold:**	checked
**Compute median over entire database:**	false
**Cutoff value used in analysis:**	0
**Expression range of the probe:**	0 - 2
**Invert HR values below 1:**	not checked

Restrictions

Tumor type: Pancreatic ductal adenocarcinoma

Restrict analysis to subtypes...

Stage:	all
Gender:	all
Race:	all
Grade:	all
Mutation burden:	all

Restrict analysis based on cellular content...

Basophils:	all
B-cells:	all
CD4+ memory T-cells:	all
CD8+ T-cells:	all
Eosinophils:	all
Macrophages:	all
Mesenchymal stem cells:	all
Natural killer T-cells:	all
Regulatory T-cells:	enriched
Type 1 T-helper cells:	all
Type 2 T-helper cells:	all

Results

P value: 0.0503
FDR: 100%

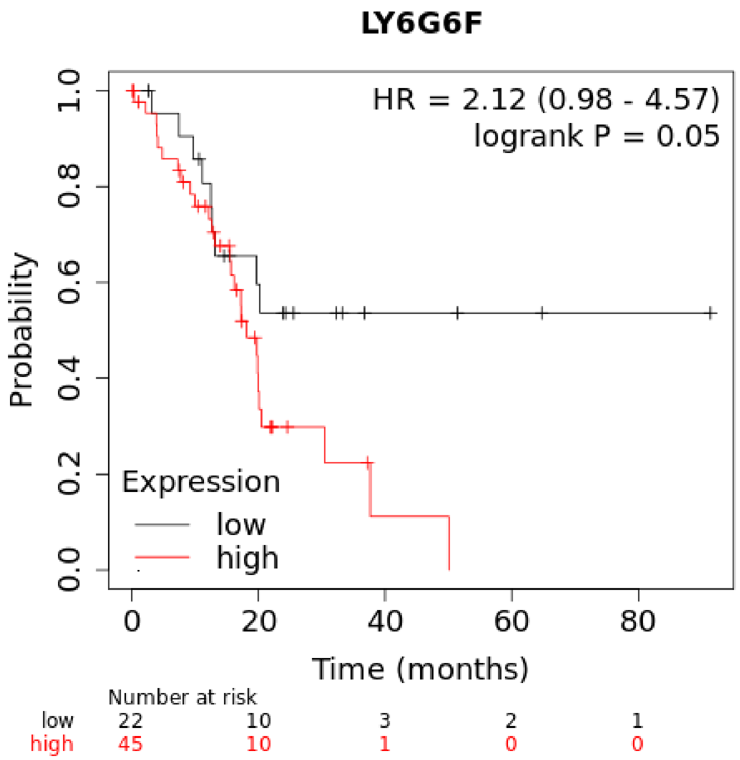

Click here to download the plot in TIFF format

Download plot as a PDF

Download p values vs. cutoff table

Upper quartile survival

Low expression cohort (months)	High expression cohort (months)
12.7	12.2

**RNAseq ID:**	LY6G5C -
**Survival:**	OS
**Auto select best cutoff:**	checked
**Follow up threshold:**	all
**Censore at threshold:**	checked
**Compute median over entire database:**	false
**Cutoff value used in analysis:**	38
**Expression range of the probe:**	17 - 253
**Invert HR values below 1:**	not checked

Restrictions

Tumor type: Pancreatic ductal adenocarcinoma

Restrict analysis to subtypes...

Stage:	all
Gender:	all
Race:	all
Grade:	all
Mutation burden:	all

Restrict analysis based on cellular content...

Basophils:	all
B-cells:	all
CD4+ memory T-cells:	all
CD8+ T-cells:	all
Eosinophils:	all
Macrophages:	all
Mesenchymal stem cells:	all
Natural killer T-cells:	all
Regulatory T-cells:	enriched
Type 1 T-helper cells:	all
Type 2 T-helper cells:	all

Results

**P value:**	0.0023
**FDR:**	50%

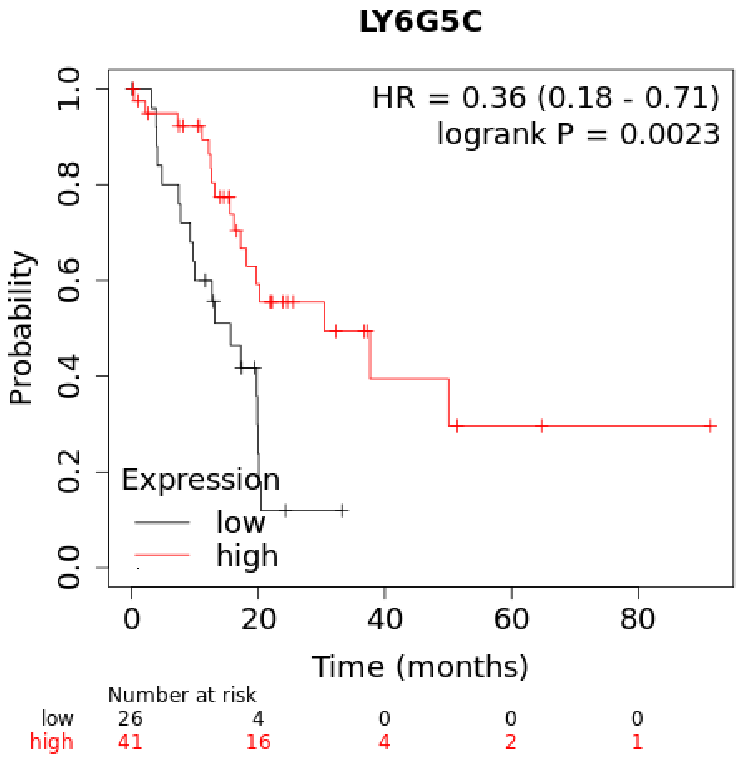

Click here to download the plot in TIFF format

Download plot as a PDF

Download p values vs. cutoff table

Median survival

Low expression cohort (months)	High expression cohort (months)
15.67	30.43

**RNAseq ID:**	LY6G5B -
**Survival:**	OS
**Auto select best cutoff:**	checked
**Follow up threshold:**	all
**Censore at threshold:**	checked
**Compute median over entire database:**	false
**Cutoff value used in analysis:**	56
**Expression range of the probe:**	6 - 144
**Invert HR values below 1:**	not checked

Restrictions

Tumor type: Pancreatic ductal adenocarcinoma

Restrict analysis to subtypes...

Stage:	all
Gender:	all
Race:	all
Grade:	all
Mutation burden:	all

Restrict analysis based on cellular content...

Basophils:	all

B-cells:	all
CD4+ memory T-cells:	all
CD8+ T-cells:	all
Eosinophils:	all
Macrophages:	all
Mesenchymal stem cells:	all
Natural killer T-cells:	all
Regulatory T-cells:	enriched
Type 1 T-helper cells:	all
Type 2 T-helper cells:	all

Results

P value: 0.0029
FDR: 10%

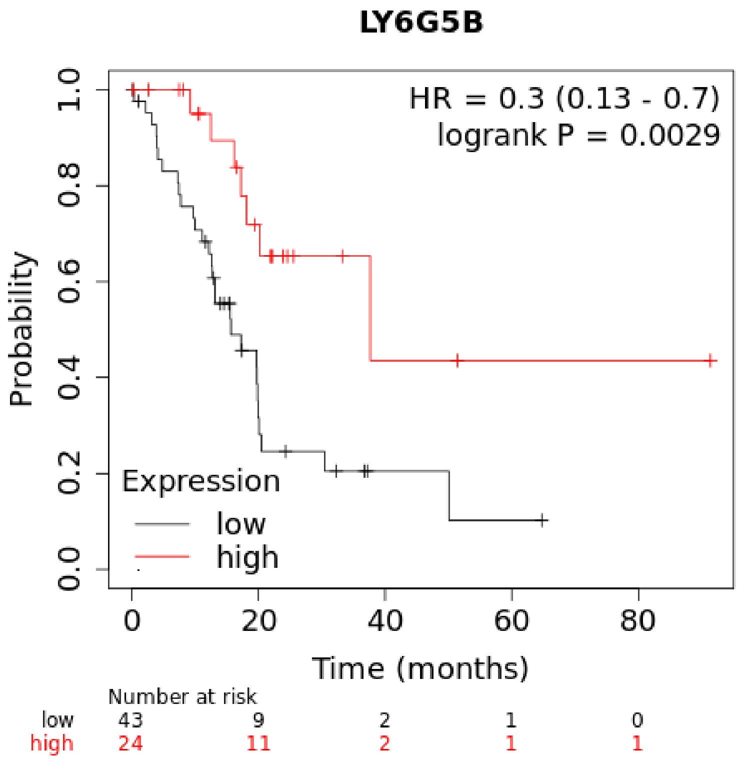

Click here to download the plot in TIFF format

Download plot as a PDF

Download p values vs. cutoff table

Median survival

Low expression cohort (months)	High expression cohort (months)
15.67	37.67

You can save the plots by right-clicking the image and then selecting "Save image as...". To generate a high resolution TIFF image, please adjust the "Settings" in the analysis page.

KMplot.com Copyright © 2009-2020 | Contact | Sitemap

Pan-cancer

KM plotter | Home | Vote | Download | Updates | Contact

The desired RNAseq ID is valid: PSCA (-), LY6K (-), SLURP1 (-), LYPD2 (-), LY6D (-), GML (-), LY6E (-), LY6L (-), LY6H (-), GPIHBP1 (-), LYPD4 (-), CD177 (-), TEX101 (-), LYPD3 (-), PINLYP (-), PLAUR (-), LYPD5 (-), SPACA4 (-), ACRV1 (-), PATE1 (-), PATE2 (-), PATE3 (-), PATE4 (-), CD59 (-), LY6G6C (-), LY6G6D (-), LY6G6F (-), LY6G5C (-), LY6G5B (-),

**RNAseq ID:**	PSCA -
**Survival:**	OS
**Auto select best cutoff:**	checked
**Follow up threshold:**	all
**Censore at threshold:**	checked
**Compute median over entire database:**	false
**Cutoff value used in analysis:**	350
**Expression range of the probe:**	0 - 65661
**Invert HR values below 1:**	not checked

Restrictions

Tumor type: Pancreatic ductal adenocarcinoma

Restrict analysis to subtypes...

Stage:	all
Gender:	all
Race:	all
Grade:	all
Mutation burden:	all

Restrict analysis based on cellular content...

Basophils:	all
B-cells:	all
CD4+ memory T-cells:	all
CD8+ T-cells:	all
Eosinophils:	all
Macrophages:	all
Mesenchymal stem cells:	all
Natural killer T-cells:	all
Regulatory T-cells:	decreased
Type 1 T-helper cells:	all
Type 2 T-helper cells:	all

Results

P value: 0.0176
FDR: over 50%

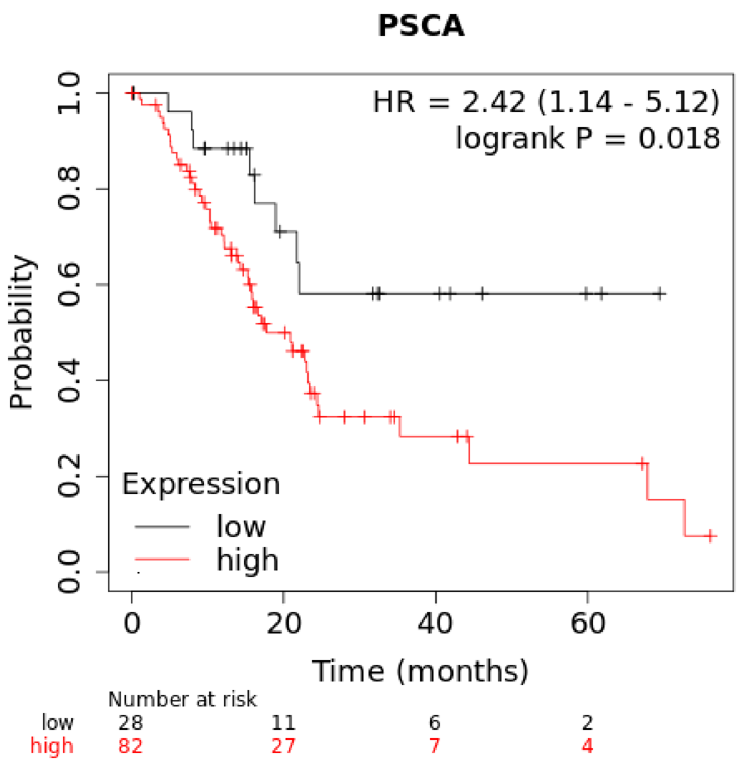

Click here to download the plot in TIFF format

Download plot as a PDF

Download p values vs. cutoff table

Upper quartile survival

Low expression cohort (months)	High expression cohort (months)
18.93	10.27

RNAseq ID: LY6K -
Survival: OS
Auto select best cutoff: checked
Follow up threshold: all
Censore at threshold: checked
Compute median over entire database: false
Cutoff value used in analysis: 6
Expression range of the probe: 0 - 1825
Invert HR values below 1: not checked

Restrictions

Tumor type: Pancreatic ductal adenocarcinoma

Restrict analysis to subtypes...

Stage: all
Gender: all
Race: all
Grade: all
Mutation burden: all

Restrict analysis based on cellular content...

Basophils: all

B-cells:	all
CD4+ memory T-cells:	all
CD8+ T-cells:	all
Eosinophils:	all
Macrophages:	all
Mesenchymal stem cells:	all
Natural killer T-cells:	all
Regulatory T-cells:	decreased
Type 1 T-helper cells:	all
Type 2 T-helper cells:	all

Results

P value: 0.0369
FDR: over 50%

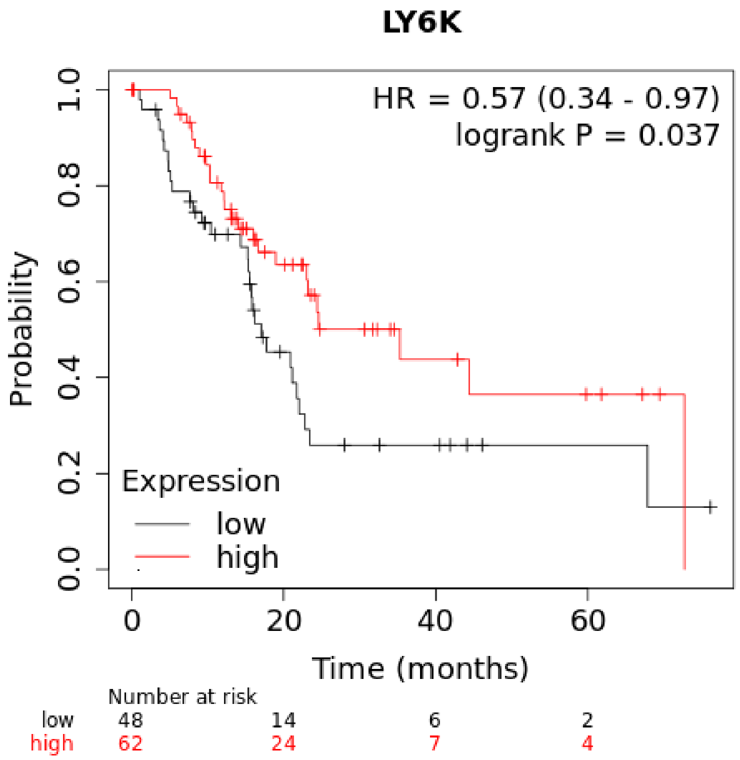

Click here to download the plot in TIFF format

Download plot as a PDF

Download p values vs. cutoff table

Median survival

Low expression cohort (months)	High expression cohort (months)
17.03	35.3

**RNAseq ID:**	SLURP1 -
**Survival:**	OS
**Auto select best cutoff:**	checked
**Follow up threshold:**	all
**Censore at threshold:**	checked
**Compute median over entire database:**	false
**Cutoff value used in analysis:**	1
**Expression range of the probe:**	0 - 279
**Invert HR values below 1:**	not checked

Restrictions

Tumor type: Pancreatic ductal adenocarcinoma

Restrict analysis to subtypes...

Stage:	all
Gender:	all
Race:	all
Grade:	all
Mutation burden:	all

Restrict analysis based on cellular content...

Basophils:	all
B-cells:	all
CD4+ memory T-cells:	all
CD8+ T-cells:	all
Eosinophils:	all
Macrophages:	all
Mesenchymal stem cells:	all
Natural killer T-cells:	all
Regulatory T-cells:	decreased
Type 1 T-helper cells:	all
Type 2 T-helper cells:	all

Results

P value: 0.0036
FDR: over 50%

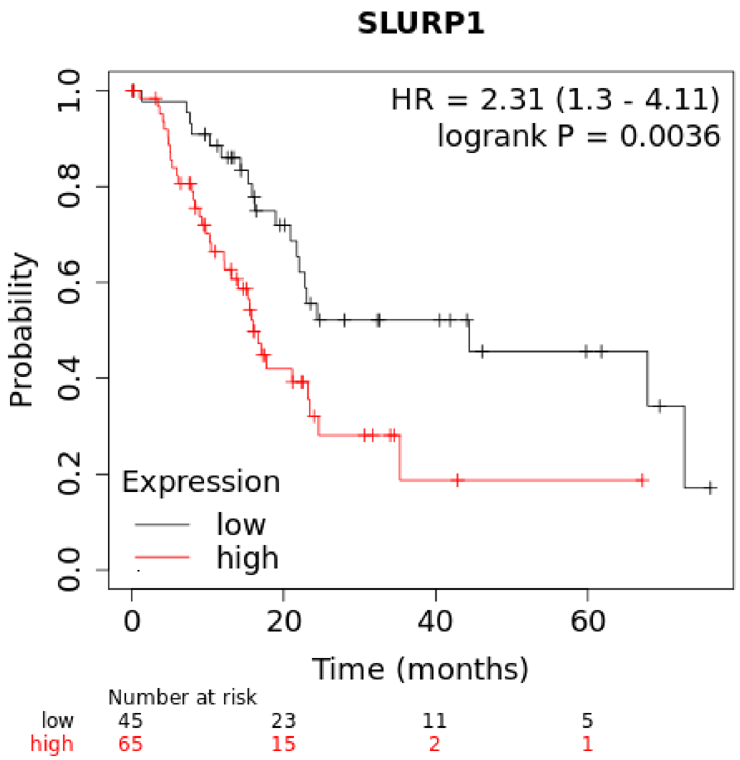

Click here to download the plot in TIFF format

Download plot as a PDF

Download p values vs. cutoff table

Median survival

Low expression cohort (months)	High expression cohort (months)
44.4	16.03

**RNAseq ID:**	LYPD2	-
**Survival:**	OS	
**Auto select best cutoff:**	checked	
**Follow up threshold:**	all	
**Censore at threshold:**	checked	
**Compute median over entire database:**	false	
**Cutoff value used in analysis:**	39	
**Expression range of the probe:**	0 - 2799	
**Invert HR values below 1:**	not checked	

Restrictions

Tumor type: Pancreatic ductal adenocarcinoma

Restrict analysis to subtypes...

Stage:	all
Gender:	all
Race:	all
Grade:	all
Mutation burden:	all

Restrict analysis based on cellular content...

Basophils:	all
B-cells:	all
CD4+ memory T-cells:	all
CD8+ T-cells:	all
Eosinophils:	all
Macrophages:	all
Mesenchymal stem cells:	all
Natural killer T-cells:	all
Regulatory T-cells:	decreased
Type 1 T-helper cells:	all
Type 2 T-helper cells:	all

Results

**P value:**	0.0921
**FDR:**	100%

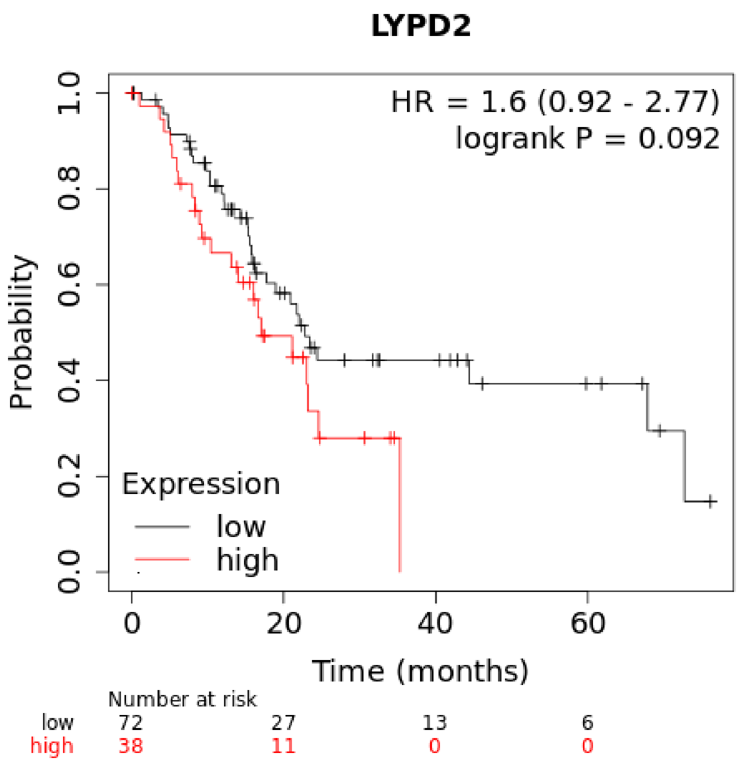

Click here to download the plot in TIFF format

Download plot as a PDF

Download p values vs. cutoff table

Median survival

Low expression cohort (months)	High expression cohort (months)
22.8	17.03

**RNAseq ID:**	LY6D -
**Survival:**	OS
**Auto select best cutoff:**	checked
**Follow up threshold:**	all
**Censore at threshold:**	checked
**Compute median over entire database:**	false
**Cutoff value used in analysis:**	642
**Expression range of the probe:**	0 - 18030
**Invert HR values below 1:**	not checked

Restrictions

Tumor type: Pancreatic ductal adenocarcinoma

Restrict analysis to subtypes...

Stage:	all
Gender:	all
Race:	all
Grade:	all
Mutation burden:	all

Restrict analysis based on cellular content...

Basophils:	all

B-cells:	all
CD4+ memory T-cells:	all
CD8+ T-cells:	all
Eosinophils:	all
Macrophages:	all
Mesenchymal stem cells:	all
Natural killer T-cells:	all
Regulatory T-cells:	decreased
Type 1 T-helper cells:	all
Type 2 T-helper cells:	all

Results

P value: 0.0022
FDR: 20%

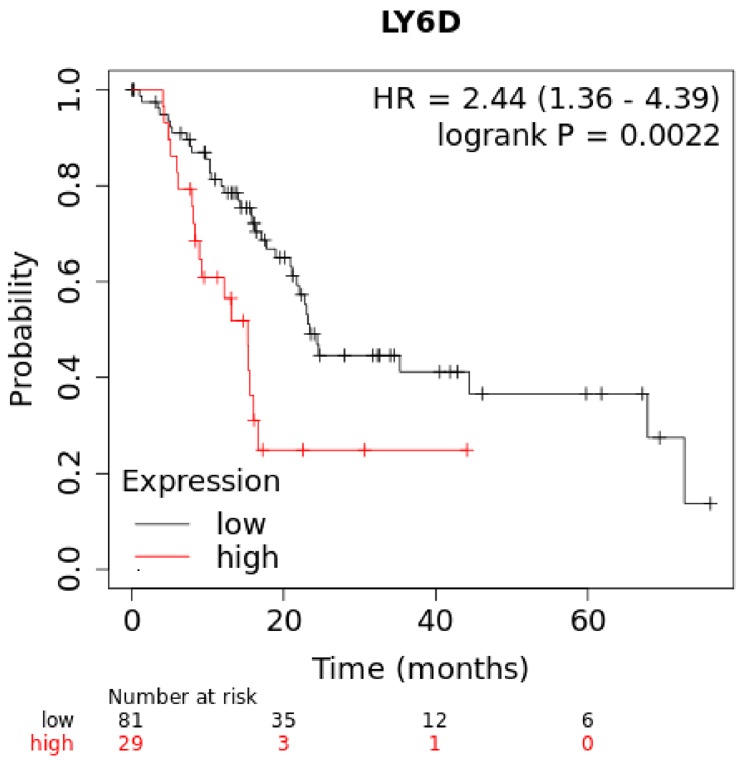

Click here to download the plot in TIFF format

Download plot as a PDF

Download p values vs. cutoff table

Median survival

Low expression cohort (months)	High expression cohort (months)
23.4	15.27

**RNAseq ID:**	GML -
**Survival:**	OS
**Auto select best cutoff:**	checked
**Follow up threshold:**	all
**Censore at threshold:**	checked
**Compute median over entire database:**	false
**Cutoff value used in analysis:**	0
**Expression range of the probe:**	0 - 2
**Invert HR values below 1:**	not checked

Restrictions

Tumor type: Pancreatic ductal adenocarcinoma

Restrict analysis to subtypes...

Stage:	all
Gender:	all
Race:	all
Grade:	all
Mutation burden:	all

Restrict analysis based on cellular content...

Basophils:	all
B-cells:	all
CD4+ memory T-cells:	all
CD8+ T-cells:	all
Eosinophils:	all
Macrophages:	all
Mesenchymal stem cells:	all
Natural killer T-cells:	all
Regulatory T-cells:	decreased
Type 1 T-helper cells:	all
Type 2 T-helper cells:	all

Results

P value: 0.0754
FDR: 100%

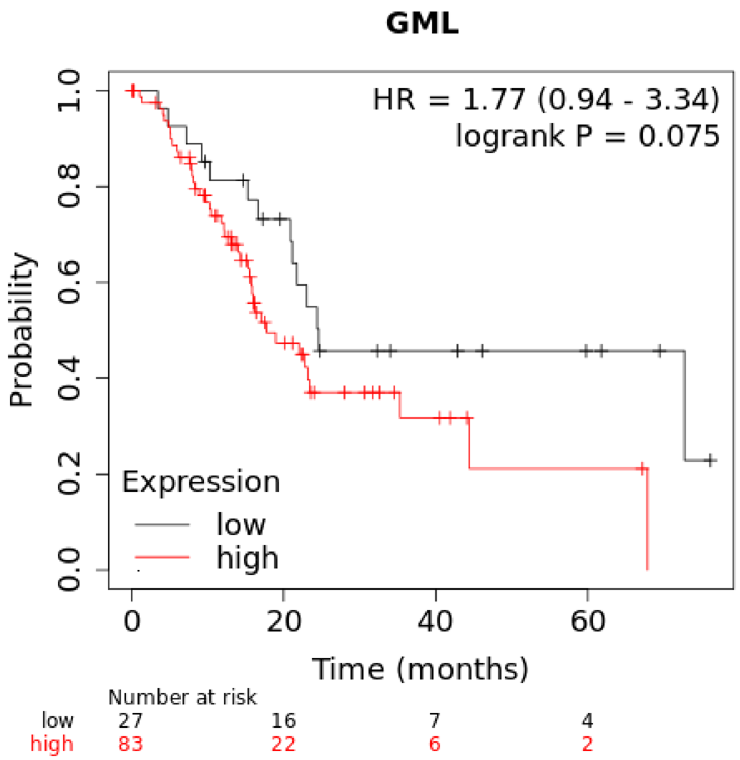

Click here to download the plot in TIFF format

Download plot as a PDF

Download p values vs. cutoff table

Median survival

Low expression cohort (months)	High expression cohort (months)
24.6	17.73

**RNAseq ID:**	LY6E -
**Survival:**	OS
**Auto select best cutoff:**	checked
**Follow up threshold:**	all
**Censore at threshold:**	checked
**Compute median over entire database:**	false
**Cutoff value used in analysis:**	10191
**Expression range of the probe:**	504 - 56404
**Invert HR values below 1:**	not checked

Restrictions

Tumor type: Pancreatic ductal adenocarcinoma

Restrict analysis to subtypes...

Stage:	all
Gender:	all
Race:	all
Grade:	all
Mutation burden:	all

Restrict analysis based on cellular content...

Basophils:	all
B-cells:	all
CD4+ memory T-cells:	all
CD8+ T-cells:	all
Eosinophils:	all
Macrophages:	all
Mesenchymal stem cells:	all
Natural killer T-cells:	all
Regulatory T-cells:	decreased
Type 1 T-helper cells:	all
Type 2 T-helper cells:	all

Results

**P value:**	0.0221
**FDR:**	over 50%

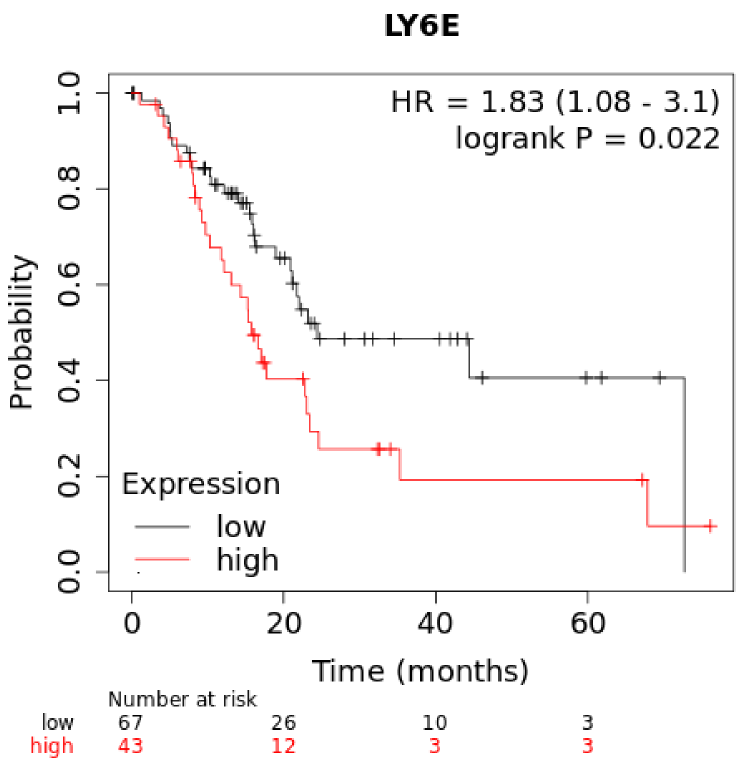

Click here to download the plot in TIFF format

Download plot as a PDF

Download p values vs. cutoff table

Median survival

Low expression cohort (months)	High expression cohort (months)
24.4	15.77

**RNAseq ID:**	LY6L -
**Survival:**	OS
**Auto select best cutoff:**	checked
**Follow up threshold:**	all
**Censore at threshold:**	checked
**Compute median over entire database:**	false
**Cutoff value used in analysis:**	0
**Expression range of the probe:**	0 - 8
**Invert HR values below 1:**	not checked

Restrictions

Tumor type: Pancreatic ductal adenocarcinoma

Restrict analysis to subtypes...

Stage:	all
Gender:	all
Race:	all
Grade:	all
Mutation burden:	all

Restrict analysis based on cellular content...

Basophils:	all

B-cells:	all
CD4+ memory T-cells:	all
CD8+ T-cells:	all
Eosinophils:	all
Macrophages:	all
Mesenchymal stem cells:	all
Natural killer T-cells:	all
Regulatory T-cells:	decreased
Type 1 T-helper cells:	all
Type 2 T-helper cells:	all

Results

P value: 0.1474
FDR: 100%

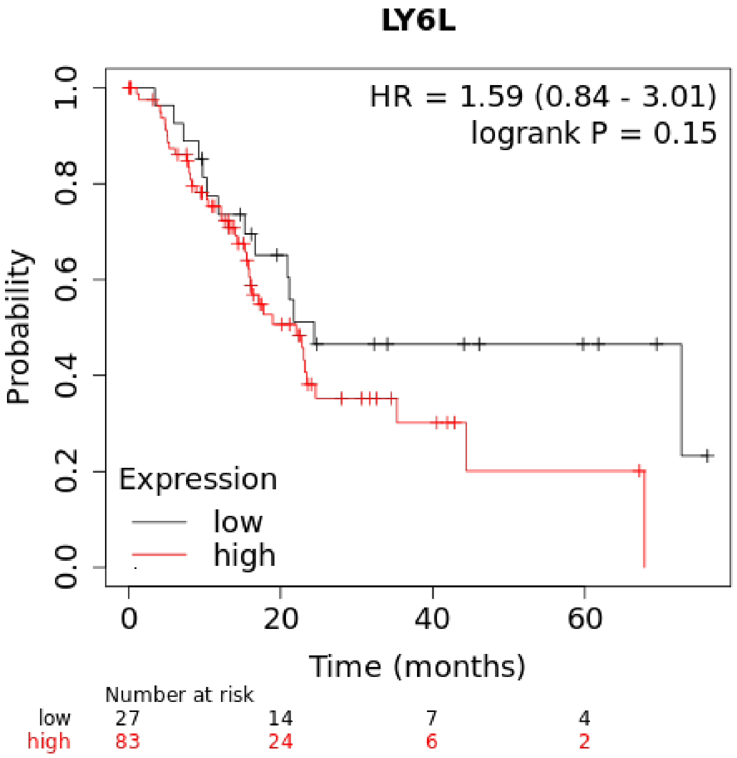

Click here to download the plot in TIFF format

Download plot as a PDF

Download p values vs. cutoff table

Median survival

Low expression cohort (months)	High expression cohort (months)
24.4	22.03

**RNAseq ID:**	LY6H -
**Survival:**	OS
**Auto select best cutoff:**	checked
**Follow up threshold:**	all
**Censore at threshold:**	checked
**Compute median over entire database:**	false
**Cutoff value used in analysis:**	28
**Expression range of the probe:**	1 - 9495
**Invert HR values below 1:**	not checked

Restrictions

Tumor type: Pancreatic ductal adenocarcinoma

Restrict analysis to subtypes...

Stage:	all
Gender:	all
Race:	all
Grade:	all
Mutation burden:	all

Restrict analysis based on cellular content...

Basophils:	all
B-cells:	all
CD4+ memory T-cells:	all
CD8+ T-cells:	all
Eosinophils:	all
Macrophages:	all
Mesenchymal stem cells:	all
Natural killer T-cells:	all
Regulatory T-cells:	decreased
Type 1 T-helper cells:	all
Type 2 T-helper cells:	all

Results

P value: 0.1653
FDR: 100%

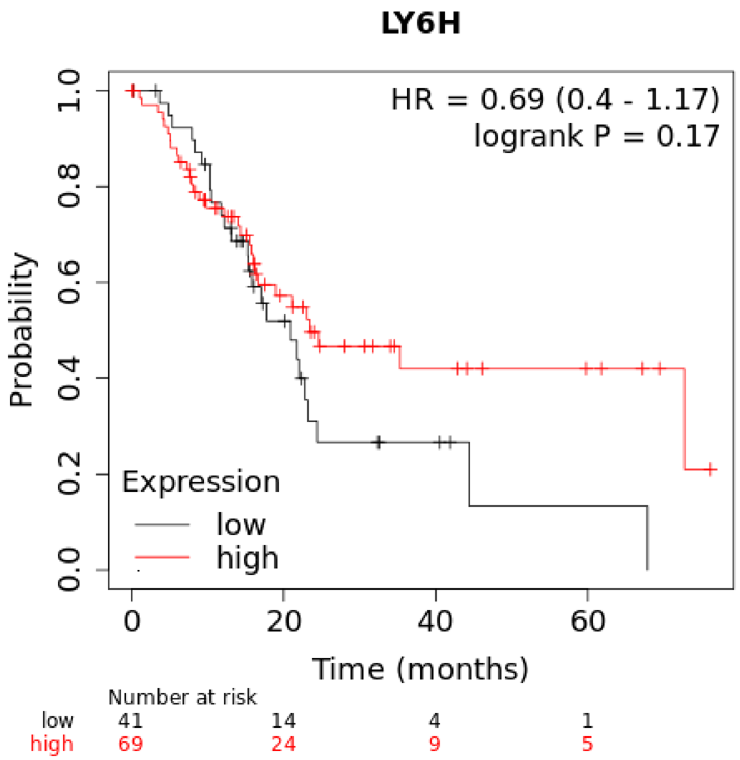

Click here to download the plot in TIFF format

Download plot as a PDF

Download p values vs. cutoff table

Median survival

Low expression cohort (months)	High expression cohort (months)
20.9	23.4

**RNAseq ID:**	GPIHBP1 -
**Survival:**	OS
**Auto select best cutoff:**	checked
**Follow up threshold:**	all
**Censore at threshold:**	checked
**Compute median over entire database:**	false
**Cutoff value used in analysis:**	63
**Expression range of the probe:**	7 - 344
**Invert HR values below 1:**	not checked

Restrictions

Tumor type: Pancreatic ductal adenocarcinoma

Restrict analysis to subtypes...

Stage:	all
Gender:	all
Race:	all
Grade:	all
Mutation burden:	all

Restrict analysis based on cellular content...

Basophils:	all
B-cells:	all
CD4+ memory T-cells:	all
CD8+ T-cells:	all
Eosinophils:	all
Macrophages:	all
Mesenchymal stem cells:	all
Natural killer T-cells:	all
Regulatory T-cells:	decreased
Type 1 T-helper cells:	all
Type 2 T-helper cells:	all

Results

P value: 0.2719
FDR: 100%

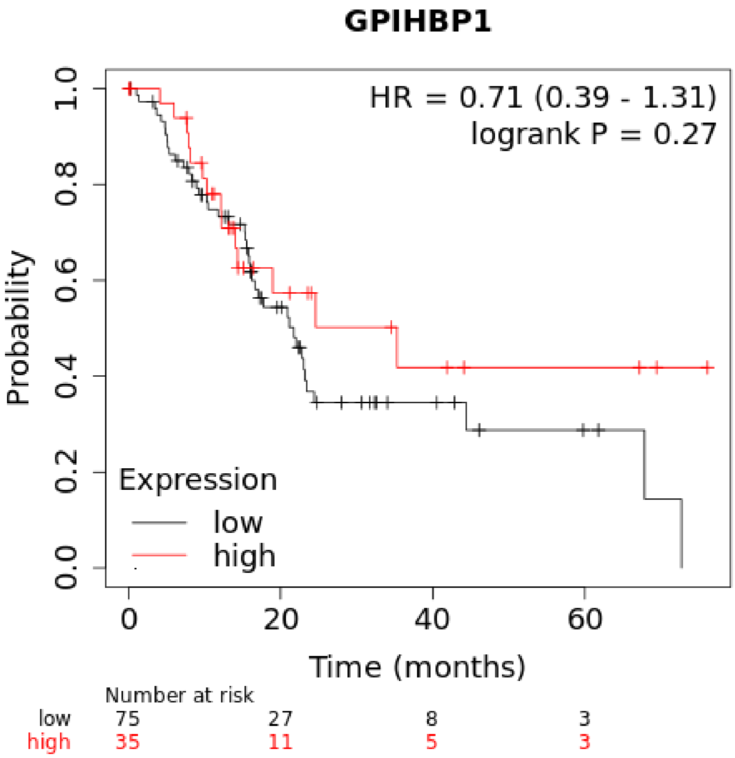

Click here to download the plot in TIFF format

Download plot as a PDF

Download p values vs. cutoff table

Median survival

Low expression cohort (months)	High expression cohort (months)
21.73	35.3

**RNAseq ID:**	LYPD4 -
**Survival:**	OS
**Auto select best cutoff:**	checked
**Follow up threshold:**	all
**Censore at threshold:**	checked
**Compute median over entire database:**	false
**Cutoff value used in analysis:**	0
**Expression range of the probe:**	0 - 18
**Invert HR values below 1:**	not checked

Restrictions

Tumor type: Pancreatic ductal adenocarcinoma

Restrict analysis to subtypes...

Stage:	all
Gender:	all
Race:	all
Grade:	all
Mutation burden:	all

Restrict analysis based on cellular content...

Basophils:	all

B-cells:	all
CD4+ memory T-cells:	all
CD8+ T-cells:	all
Eosinophils:	all
Macrophages:	all
Mesenchymal stem cells:	all
Natural killer T-cells:	all
Regulatory T-cells:	decreased
Type 1 T-helper cells:	all
Type 2 T-helper cells:	all

Results

P value: 0.0901
FDR: 100%

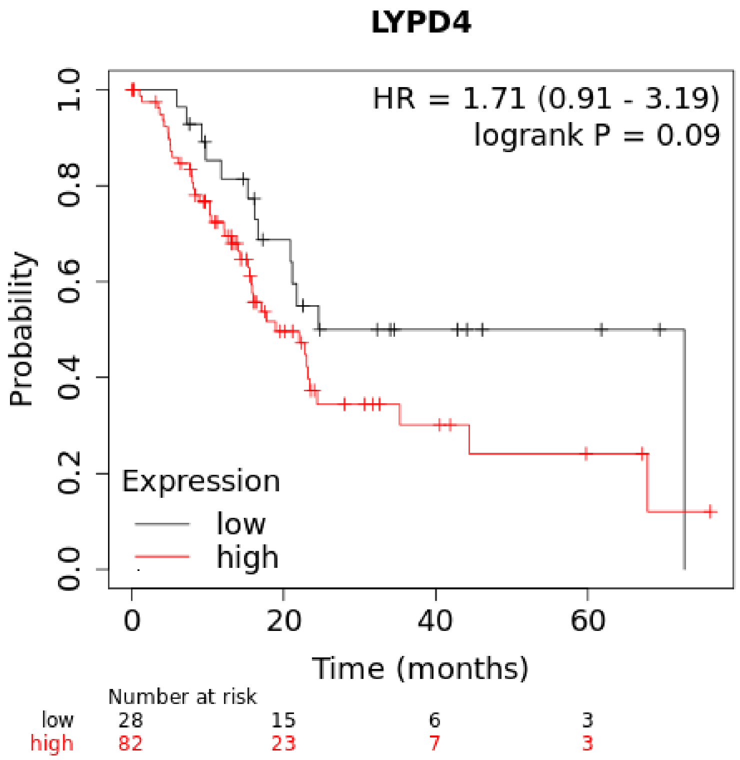

Click here to download the plot in TIFF format

Download plot as a PDF

Download p values vs. cutoff table

Median survival

Low expression cohort (months)	High expression cohort (months)
24.6	18.93

**RNAseq ID:**	CD177 -
**Survival:**	OS
**Auto select best cutoff:**	checked
**Follow up threshold:**	all
**Censore at threshold:**	checked
**Compute median over entire database:**	false
**Cutoff value used in analysis:**	58
**Expression range of the probe:**	0 - 6100
**Invert HR values below 1:**	not checked

Restrictions

Tumor type: Pancreatic ductal adenocarcinoma

Restrict analysis to subtypes...

Stage:	all
Gender:	all
Race:	all
Grade:	all
Mutation burden:	all

Restrict analysis based on cellular content...

Basophils:	all
B-cells:	all
CD4+ memory T-cells:	all
CD8+ T-cells:	all
Eosinophils:	all
Macrophages:	all
Mesenchymal stem cells:	all
Natural killer T-cells:	all
Regulatory T-cells:	decreased
Type 1 T-helper cells:	all
Type 2 T-helper cells:	all

Results

P value: 0.1375
FDR: 100%

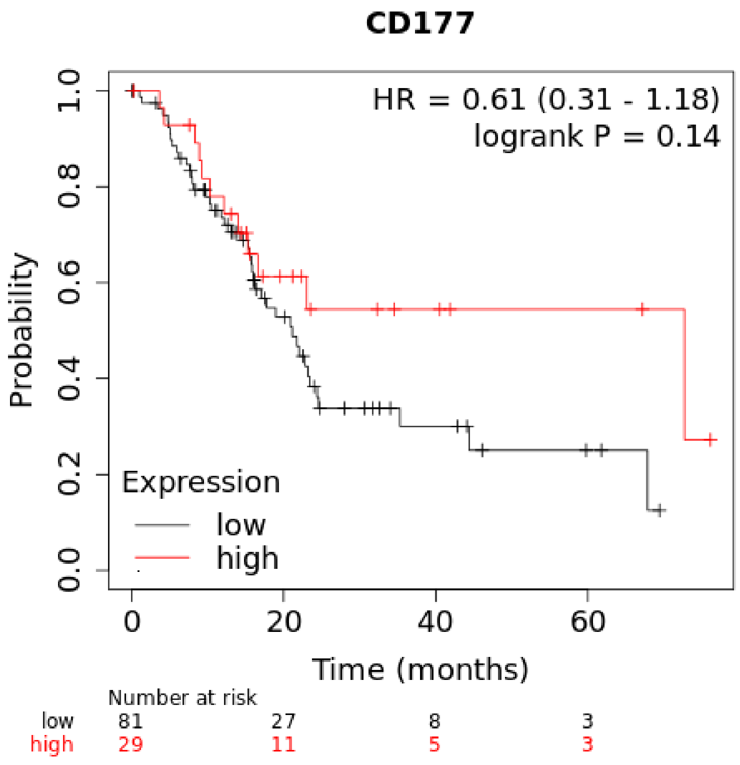

Click here to download the plot in TIFF format

Download plot as a PDF

Download p values vs. cutoff table

Median survival

Low expression cohort (months)	High expression cohort (months)
21.13	72.73

**RNAseq ID:**	TEX101 -
**Survival:**	OS
**Auto select best cutoff:**	checked
**Follow up threshold:**	all
**Censore at threshold:**	checked
**Compute median over entire database:**	false
**Cutoff value used in analysis:**	1
**Expression range of the probe:**	0 - 149
**Invert HR values below 1:**	not checked

Restrictions

Tumor type: Pancreatic ductal adenocarcinoma

Restrict analysis to subtypes...

Stage:	all
Gender:	all
Race:	all
Grade:	all
Mutation burden:	all

Restrict analysis based on cellular content...

Basophils:	all
B-cells:	all
CD4+ memory T-cells:	all
CD8+ T-cells:	all
Eosinophils:	all
Macrophages:	all
Mesenchymal stem cells:	all
Natural killer T-cells:	all
Regulatory T-cells:	decreased
Type 1 T-helper cells:	all
Type 2 T-helper cells:	all

Results

**P value:**	0.0098
**FDR:**	over 50%

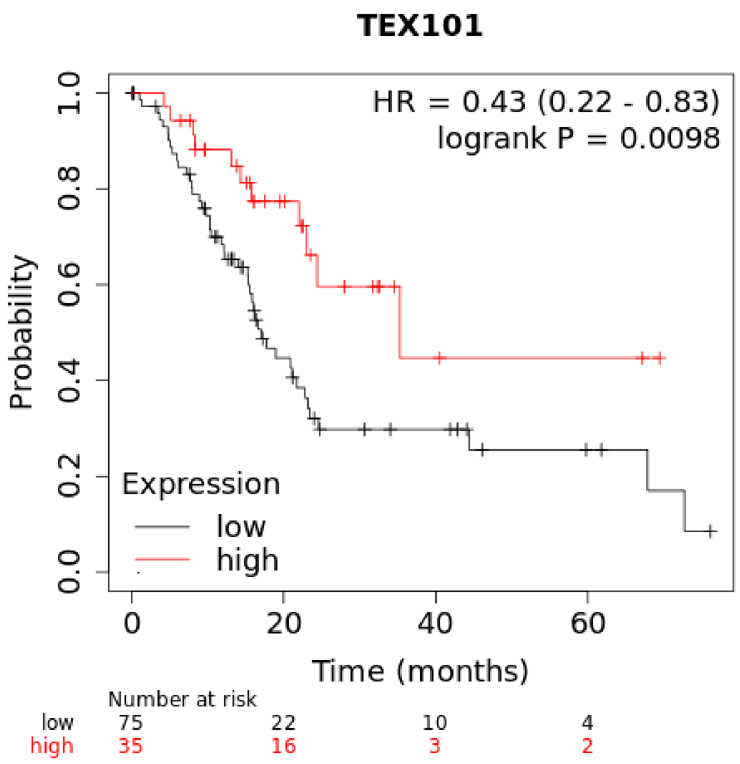

Click here to download the plot in TIFF format

Download plot as a PDF

Download p values vs. cutoff table

Median survival

Low expression cohort (months)	High expression cohort (months)
17.03	35.3

**RNAseq ID:**	LYPD3 -
**Survival:**	OS
**Auto select best cutoff:**	checked
**Follow up threshold:**	all
**Censore at threshold:**	checked
**Compute median over entire database:**	false
**Cutoff value used in analysis:**	102
**Expression range of the probe:**	9 - 3640
**Invert HR values below 1:**	not checked

Restrictions

Tumor type: Pancreatic ductal adenocarcinoma

Restrict analysis to subtypes...

Stage:	all
Gender:	all
Race:	all
Grade:	all
Mutation burden:	all

Restrict analysis based on cellular content...

Basophils:	all

B-cells: all
CD4+ memory T-cells: all
CD8+ T-cells: all
Eosinophils: all
Macrophages: all
Mesenchymal stem cells: all
Natural killer T-cells: all
Regulatory T-cells: decreased
Type 1 T-helper cells: all
Type 2 T-helper cells: all

Results

P value: 0.0941
FDR: 100%

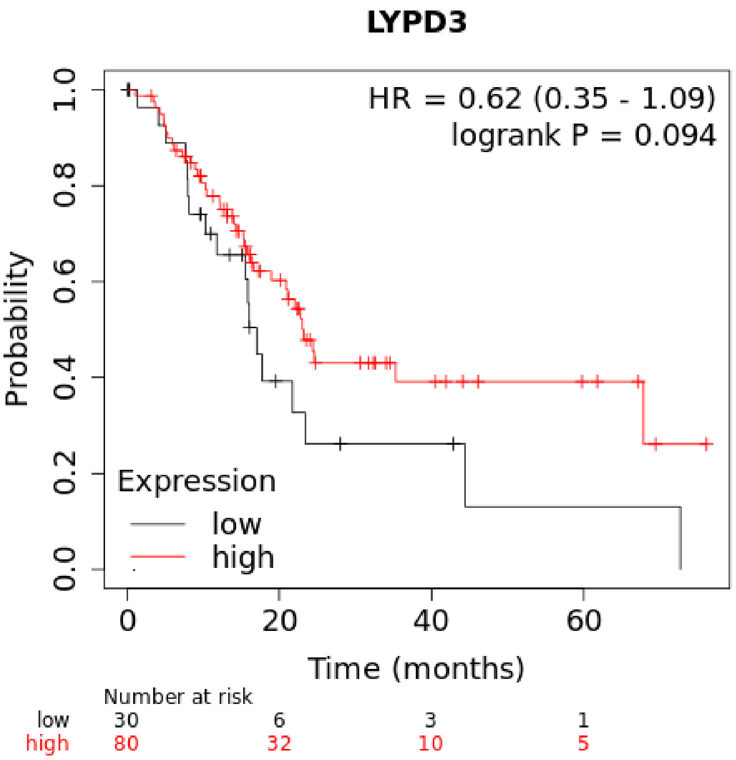

Click here to download the plot in TIFF format

Download plot as a PDF

Download p values vs. cutoff table

Median survival

Low expression cohort (months)	High expression cohort (months)
17.03	23.17

RNAseq ID: PINLYP -
Survival: OS
Auto select best cutoff: checked
Follow up threshold: all
Censore at threshold: checked
Compute median over entire database: false
Cutoff value used in analysis: 76
Expression range of the probe: 5 - 387
Invert HR values below 1: not checked

Restrictions

Tumor type: Pancreatic ductal adenocarcinoma

Restrict analysis to subtypes...

Stage: all
Gender: all
Race: all
Grade: all
Mutation burden: all

Restrict analysis based on cellular content...

Basophils: all
B-cells: all
CD4+ memory T-cells: all
CD8+ T-cells: all
Eosinophils: all
Macrophages: all
Mesenchymal stem cells: all
Natural killer T-cells: all
Regulatory T-cells: decreased
Type 1 T-helper cells: all
Type 2 T-helper cells: all

Results

P value: 0.1095
FDR: 100%

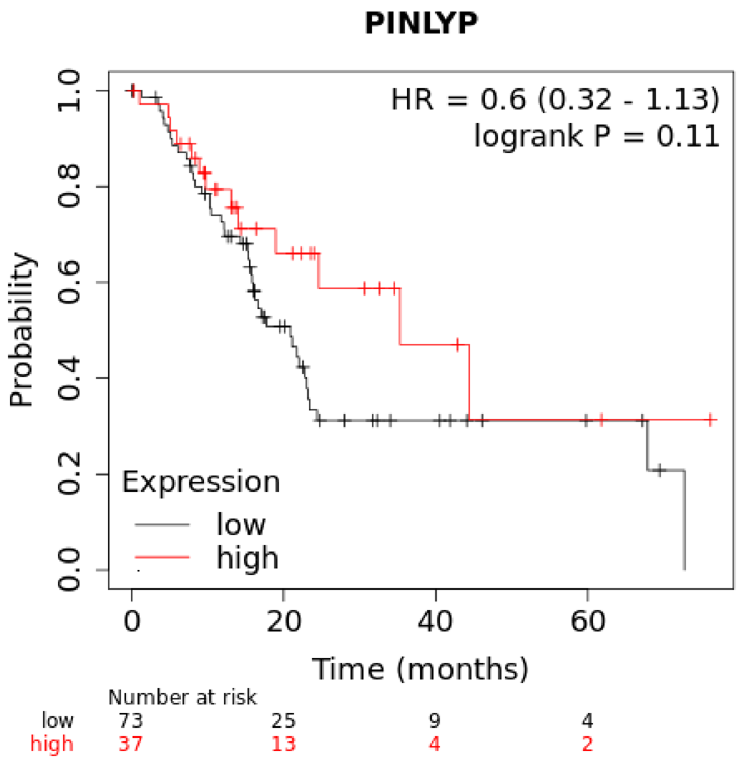

Click here to download the plot in TIFF format

Download plot as a PDF

Download p values vs. cutoff table

Median survival

Low expression cohort (months)	High expression cohort (months)
20.9	35.3

**RNAseq ID:**	PLAUR -
**Survival:**	OS
**Auto select best cutoff:**	checked
**Follow up threshold:**	all
**Censore at threshold:**	checked
**Compute median over entire database:**	false
**Cutoff value used in analysis:**	2095
**Expression range of the probe:**	55 - 14016
**Invert HR values below 1:**	not checked

Restrictions

Tumor type: Pancreatic ductal adenocarcinoma

Restrict analysis to subtypes...

Stage:	all
Gender:	all
Race:	all
Grade:	all
Mutation burden:	all

Restrict analysis based on cellular content...

Basophils:	all
B-cells:	all
CD4+ memory T-cells:	all
CD8+ T-cells:	all
Eosinophils:	all
Macrophages:	all
Mesenchymal stem cells:	all
Natural killer T-cells:	all
Regulatory T-cells:	decreased
Type 1 T-helper cells:	all
Type 2 T-helper cells:	all

Results

**P value:**	0.2022
**FDR:**	100%

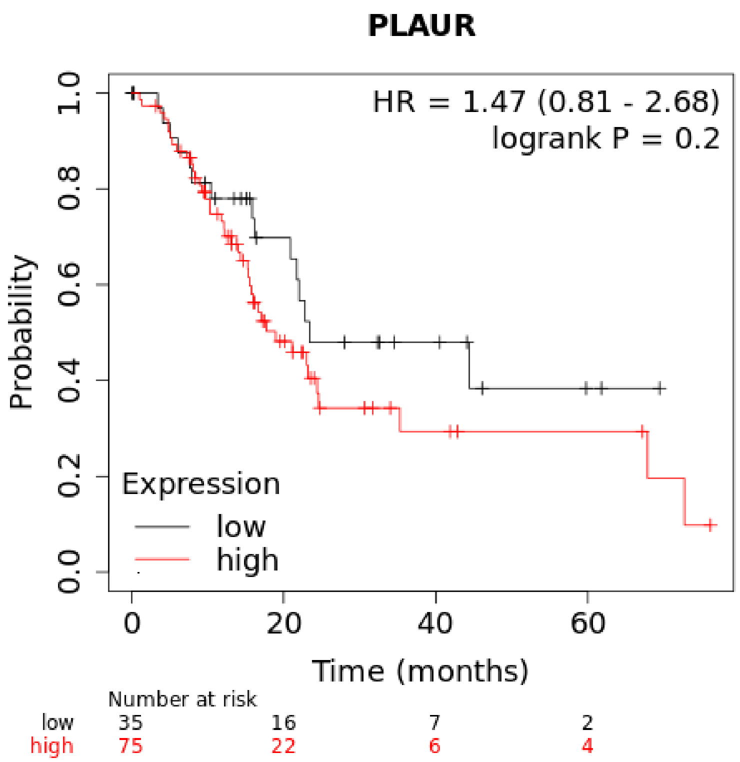

Click here to download the plot in TIFF format

Download plot as a PDF

Download p values vs. cutoff table

Median survival

Low expression cohort (months)	High expression cohort (months)
23.4	18.93

RNAseq ID: LYPD5 -
Survival: OS
Auto select best cutoff: checked
Follow up threshold: all
Censore at threshold: checked
Compute median over entire database: false
Cutoff value used in analysis: 74
Expression range of the probe: 5 - 578
Invert HR values below 1: not checked

Restrictions

Tumor type: Pancreatic ductal adenocarcinoma

Restrict analysis to subtypes...

Stage: all
Gender: all
Race: all
Grade: all
Mutation burden: all

Restrict analysis based on cellular content...

Basophils: all

B-cells:	all
CD4+ memory T-cells:	all
CD8+ T-cells:	all
Eosinophils:	all
Macrophages:	all
Mesenchymal stem cells:	all
Natural killer T-cells:	all
Regulatory T-cells:	decreased
Type 1 T-helper cells:	all
Type 2 T-helper cells:	all

Results

P value: 0.0077
FDR: 50%

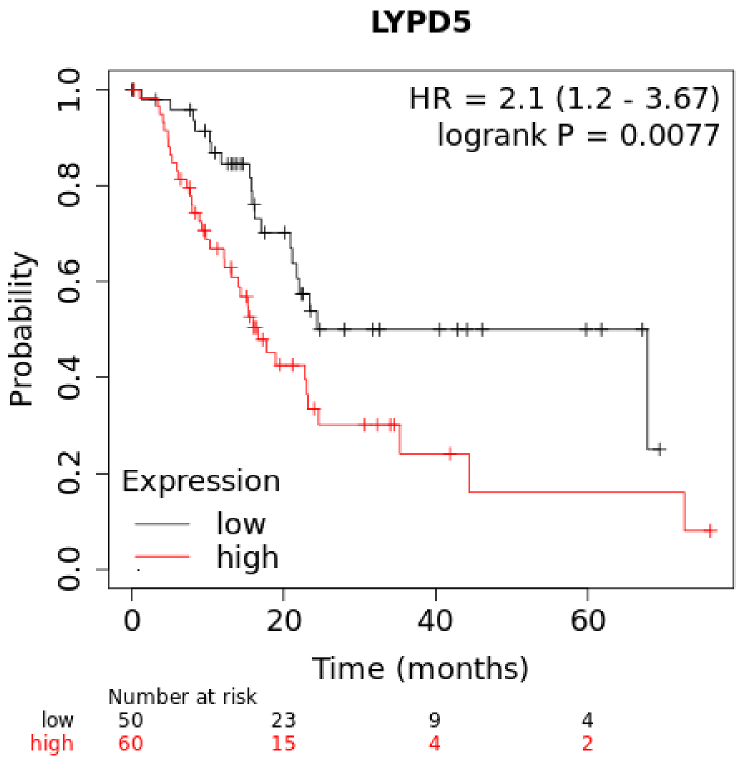

Click here to download the plot in TIFF format

Download plot as a PDF

Download p values vs. cutoff table

Median survival

Low expression cohort (months)	High expression cohort (months)
67.87	16.6

**RNAseq ID:**	SPACA4 -
**Survival:**	OS
**Auto select best cutoff:**	checked
**Follow up threshold:**	all
**Censore at threshold:**	checked
**Compute median over entire database:**	false
**Cutoff value used in analysis:**	19
**Expression range of the probe:**	0 - 206
**Invert HR values below 1:**	not checked

Restrictions

Tumor type: Pancreatic ductal adenocarcinoma

Restrict analysis to subtypes...

Stage:	all
Gender:	all
Race:	all
Grade:	all
Mutation burden:	all

Restrict analysis based on cellular content...

Basophils:	all
B-cells:	all
CD4+ memory T-cells:	all
CD8+ T-cells:	all
Eosinophils:	all
Macrophages:	all
Mesenchymal stem cells:	all
Natural killer T-cells:	all
Regulatory T-cells:	decreased
Type 1 T-helper cells:	all
Type 2 T-helper cells:	all

Results

P value: 0.1039
FDR: 100%

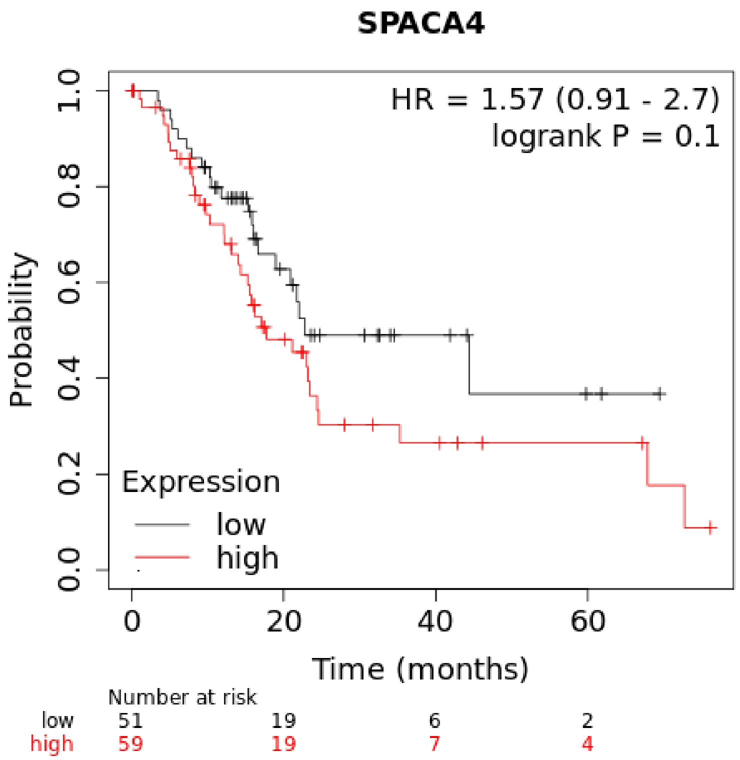

Click here to download the plot in TIFF format

Download plot as a PDF

Download p values vs. cutoff table

Median survival

Low expression cohort (months)	High expression cohort (months)
22.8	17.73

**RNAseq ID:**	ACRV1 -
**Survival:**	OS
**Auto select best cutoff:**	checked
**Follow up threshold:**	all
**Censore at threshold:**	checked
**Compute median over entire database:**	false
**Cutoff value used in analysis:**	6
**Expression range of the probe:**	0 - 71
**Invert HR values below 1:**	not checked

Restrictions

Tumor type: Pancreatic ductal adenocarcinoma

Restrict analysis to subtypes...

Stage:	all
Gender:	all
Race:	all
Grade:	all
Mutation burden:	all

Restrict analysis based on cellular content...

Basophils:	all
B-cells:	all
CD4+ memory T-cells:	all
CD8+ T-cells:	all
Eosinophils:	all
Macrophages:	all
Mesenchymal stem cells:	all
Natural killer T-cells:	all
Regulatory T-cells:	decreased
Type 1 T-helper cells:	all
Type 2 T-helper cells:	all

Results

**P value:**	0.1029
**FDR:**	100%

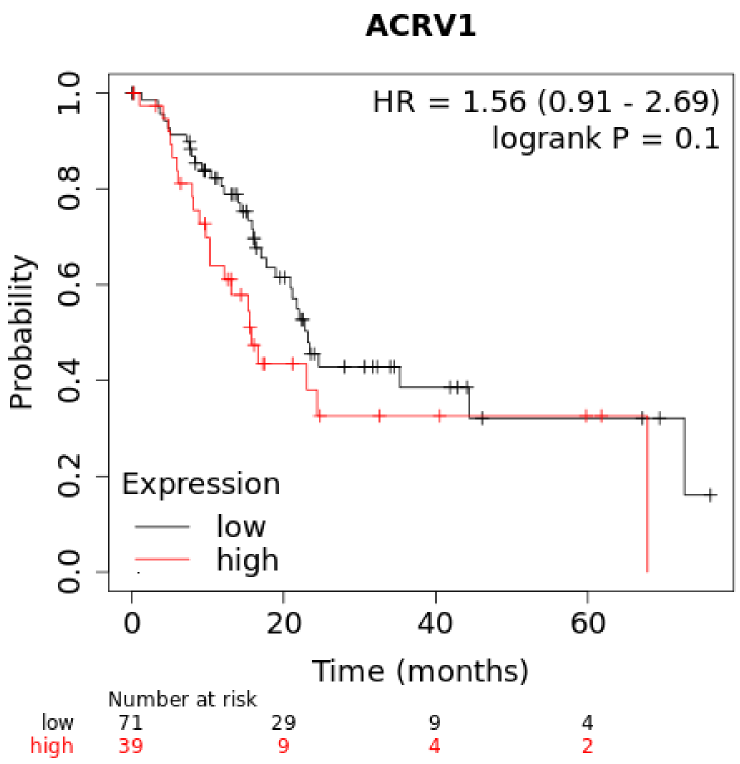

Click here to download the plot in TIFF format

Download plot as a PDF

Download p values vs. cutoff table

Median survival

Low expression cohort (months)	High expression cohort (months)
23.17	15.77

RNAseq ID: PATE1 -
Survival: OS
Auto select best cutoff: checked
Follow up threshold: all
Censore at threshold: checked
Compute median over entire database: false
Cutoff value used in analysis: 0
Expression range of the probe: 0 - 1
Invert HR values below 1: not checked

Restrictions

Tumor type: Pancreatic ductal adenocarcinoma

Restrict analysis to subtypes...

Stage: all
Gender: all
Race: all
Grade: all
Mutation burden: all

Restrict analysis based on cellular content...

Basophils: all

B-cells:	all
CD4+ memory T-cells:	all
CD8+ T-cells:	all
Eosinophils:	all
Macrophages:	all
Mesenchymal stem cells:	all
Natural killer T-cells:	all
Regulatory T-cells:	decreased
Type 1 T-helper cells:	all
Type 2 T-helper cells:	all

Results

P value: 0.0129
FDR: over 50%

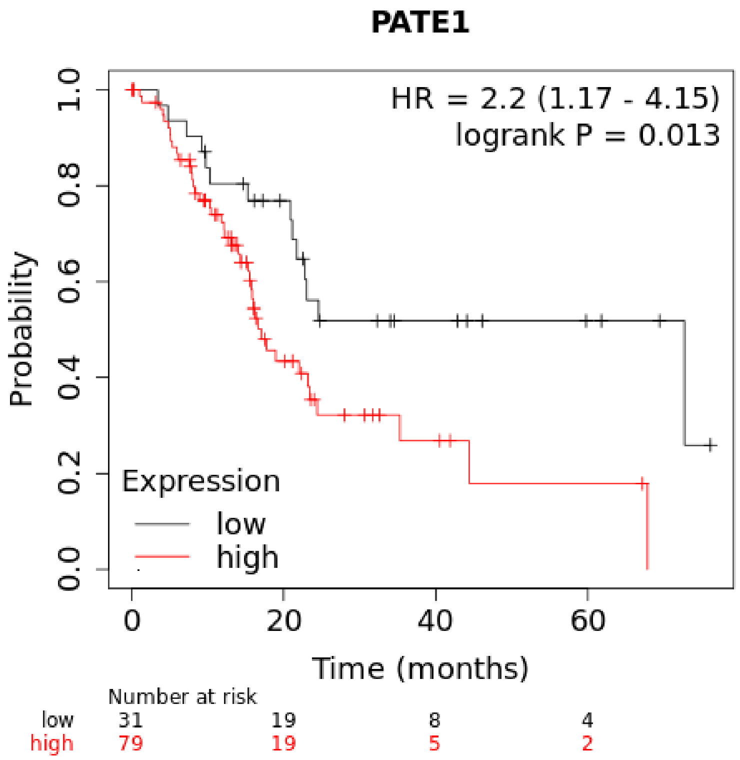

Click here to download the plot in TIFF format

Download plot as a PDF

Download p values vs. cutoff table

Median survival

Low expression cohort (months)	High expression cohort (months)
72.73	17.03

**RNAseq ID:**	PATE2 -
**Survival:**	OS
**Auto select best cutoff:**	checked
**Follow up threshold:**	all
**Censore at threshold:**	checked
**Compute median over entire database:**	false
**Cutoff value used in analysis:**	1
**Expression range of the probe:**	0 - 7
**Invert HR values below 1:**	not checked

Restrictions

Tumor type: Pancreatic ductal adenocarcinoma

Restrict analysis to subtypes...

Stage:	all
Gender:	all
Race:	all
Grade:	all
Mutation burden:	all

Restrict analysis based on cellular content...

Basophils:	all
B-cells:	all
CD4+ memory T-cells:	all
CD8+ T-cells:	all
Eosinophils:	all
Macrophages:	all
Mesenchymal stem cells:	all
Natural killer T-cells:	all
Regulatory T-cells:	decreased
Type 1 T-helper cells:	all
Type 2 T-helper cells:	all

Results

P value: 0.002
FDR: 50%

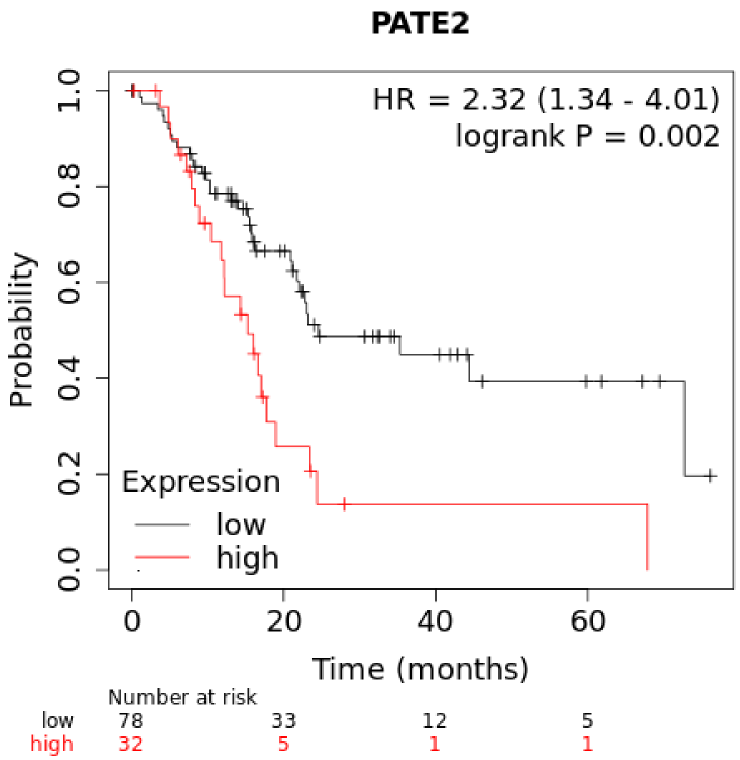

Click here to download the plot in TIFF format

Download plot as a PDF

Download p values vs. cutoff table

Median survival

Low expression cohort (months)	High expression cohort (months)
24.6	15.27

**RNAseq ID:**	PATE3 -
**Survival:**	OS
**Auto select best cutoff:**	checked
**Follow up threshold:**	all
**Censore at threshold:**	checked
**Compute median over entire database:**	false
**Cutoff value used in analysis:**	0
**Expression range of the probe:**	0 - 1
**Invert HR values below 1:**	not checked

Restrictions

Tumor type: Pancreatic ductal adenocarcinoma

Restrict analysis to subtypes...

Stage:	all
Gender:	all
Race:	all
Grade:	all
Mutation burden:	all

Restrict analysis based on cellular content...

Basophils:	all
B-cells:	all
CD4+ memory T-cells:	all
CD8+ T-cells:	all
Eosinophils:	all
Macrophages:	all
Mesenchymal stem cells:	all
Natural killer T-cells:	all
Regulatory T-cells:	decreased
Type 1 T-helper cells:	all
Type 2 T-helper cells:	all

Results

**P value:**	0.0202
**FDR:**	over 50%

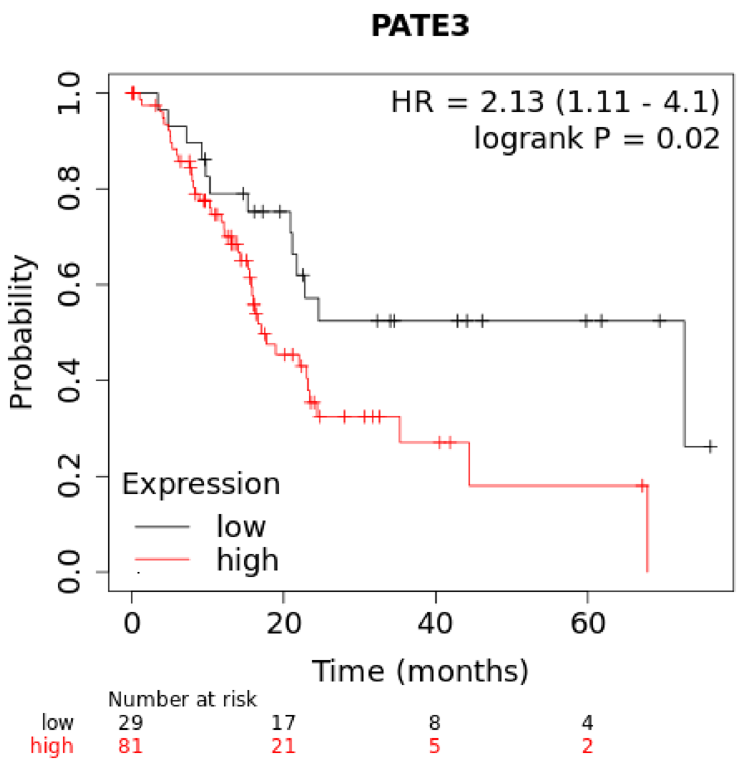

Click here to download the plot in TIFF format

Download plot as a PDF

Download p values vs. cutoff table

Median survival

Low expression cohort (months)	High expression cohort (months)
72.73	17.03

RNAseq ID: PATE4 -
Survival: OS
Auto select best cutoff: checked
Follow up threshold: all
Censore at threshold: checked
Compute median over entire database: false
Cutoff value used in analysis: 1
Expression range of the probe: 0 - 3
Invert HR values below 1: not checked

Restrictions

Tumor type: Pancreatic ductal adenocarcinoma

Restrict analysis to subtypes...

Stage: all
Gender: all
Race: all
Grade: all
Mutation burden: all

Restrict analysis based on cellular content...

Basophils: all

B-cells:	all
CD4+ memory T-cells:	all
CD8+ T-cells:	all
Eosinophils:	all
Macrophages:	all
Mesenchymal stem cells:	all
Natural killer T-cells:	all
Regulatory T-cells:	decreased
Type 1 T-helper cells:	all
Type 2 T-helper cells:	all

Results

P value: 0.385
FDR: 100%

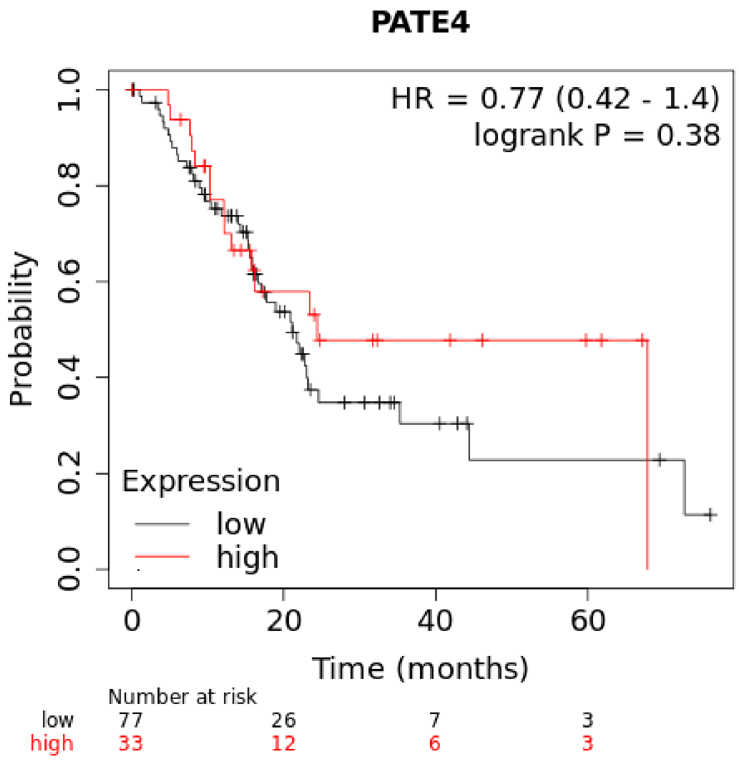

Click here to download the plot in TIFF format

Download plot as a PDF

Download p values vs. cutoff table

Median survival

Low expression cohort (months)	High expression cohort (months)
21.13	24.4

**RNAseq ID:**	CD59 -
**Survival:**	OS
**Auto select best cutoff:**	checked
**Follow up threshold:**	all
**Censore at threshold:**	checked
**Compute median over entire database:**	false
**Cutoff value used in analysis:**	17322
**Expression range of the probe:**	2610 - 39336
**Invert HR values below 1:**	not checked

Restrictions

Tumor type: Pancreatic ductal adenocarcinoma

Restrict analysis to subtypes...

Stage:	all
Gender:	all
Race:	all
Grade:	all
Mutation burden:	all

Restrict analysis based on cellular content...

Basophils:	all
B-cells:	all
CD4+ memory T-cells:	all
CD8+ T-cells:	all
Eosinophils:	all
Macrophages:	all
Mesenchymal stem cells:	all
Natural killer T-cells:	all
Regulatory T-cells:	decreased
Type 1 T-helper cells:	all
Type 2 T-helper cells:	all

Results

P value: 4.2e-5
FDR: 1%

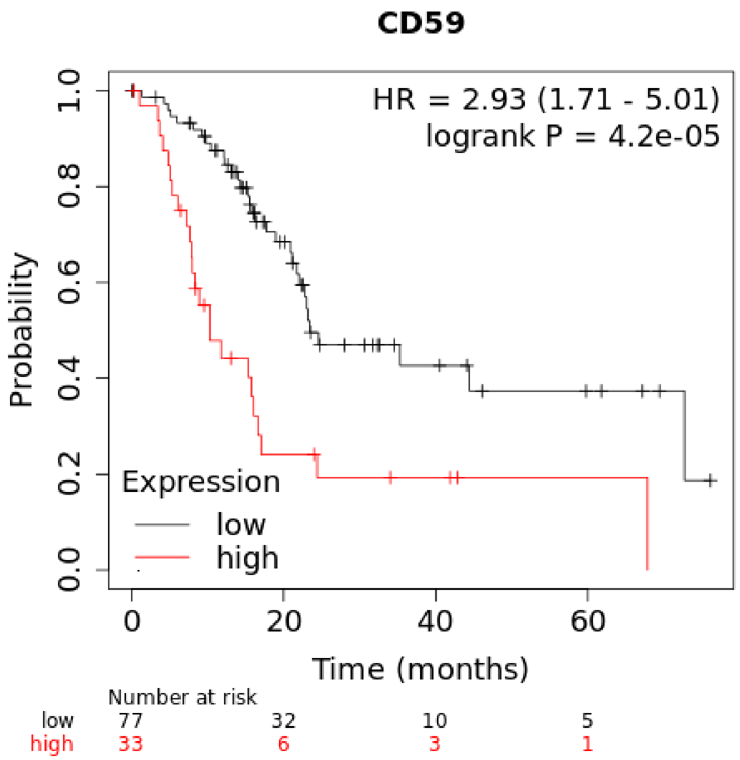

Click here to download the plot in TIFF format

Download plot as a PDF

Download p values vs. cutoff table

Median survival

Low expression cohort (months)	High expression cohort (months)
23.4	10.27

**RNAseq ID:**	LY6G6C -
**Survival:**	OS
**Auto select best cutoff:**	checked
**Follow up threshold:**	all
**Censore at threshold:**	checked
**Compute median over entire database:**	false
**Cutoff value used in analysis:**	13
**Expression range of the probe:**	0 - 251
**Invert HR values below 1:**	not checked

Restrictions

Tumor type: Pancreatic ductal adenocarcinoma

Restrict analysis to subtypes...

Stage:	all
Gender:	all
Race:	all
Grade:	all
Mutation burden:	all

Restrict analysis based on cellular content...

Basophils:	all
B-cells:	all
CD4+ memory T-cells:	all
CD8+ T-cells:	all
Eosinophils:	all
Macrophages:	all
Mesenchymal stem cells:	all
Natural killer T-cells:	all
Regulatory T-cells:	decreased
Type 1 T-helper cells:	all
Type 2 T-helper cells:	all

Results

**P value:**	0.0086
**FDR:**	over 50%

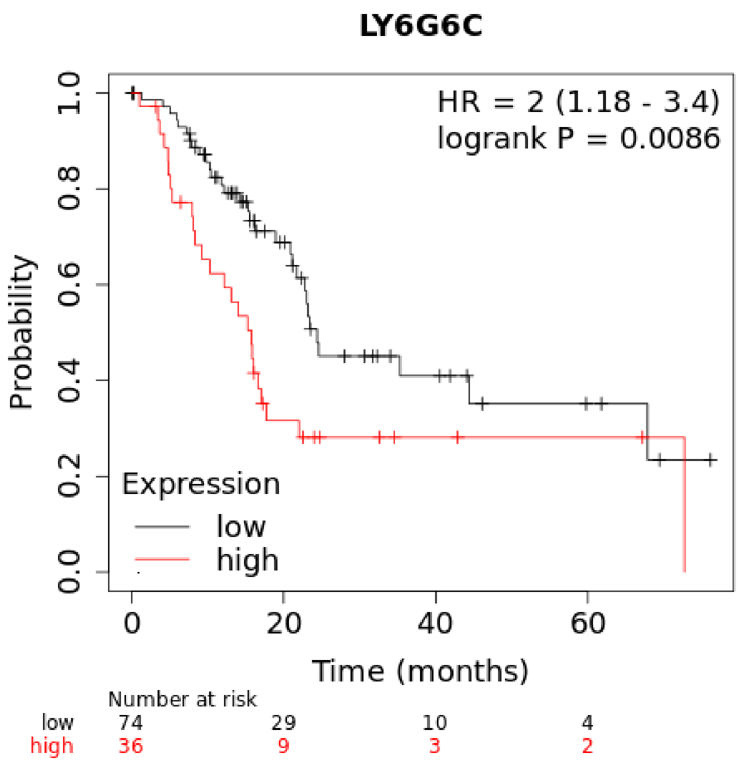

Click here to download the plot in TIFF format

Download plot as a PDF

Download p values vs. cutoff table

Median survival

Low expression cohort (months)	High expression cohort (months)
24.4	15.77

**RNAseq ID:**	LY6G6D -
**Survival:**	OS
**Auto select best cutoff:**	checked
**Follow up threshold:**	all
**Censore at threshold:**	checked
**Compute median over entire database:**	false
**Cutoff value used in analysis:**	0
**Expression range of the probe:**	0 - 2
**Invert HR values below 1:**	not checked

Restrictions

Tumor type: Pancreatic ductal adenocarcinoma

Restrict analysis to subtypes...

Stage:	all
Gender:	all
Race:	all
Grade:	all
Mutation burden:	all

Restrict analysis based on cellular content...

Basophils:	all

B-cells:	all
CD4+ memory T-cells:	all
CD8+ T-cells:	all
Eosinophils:	all
Macrophages:	all
Mesenchymal stem cells:	all
Natural killer T-cells:	all
Regulatory T-cells:	decreased
Type 1 T-helper cells:	all
Type 2 T-helper cells:	all

Results

P value: 0.044
FDR: over 50%

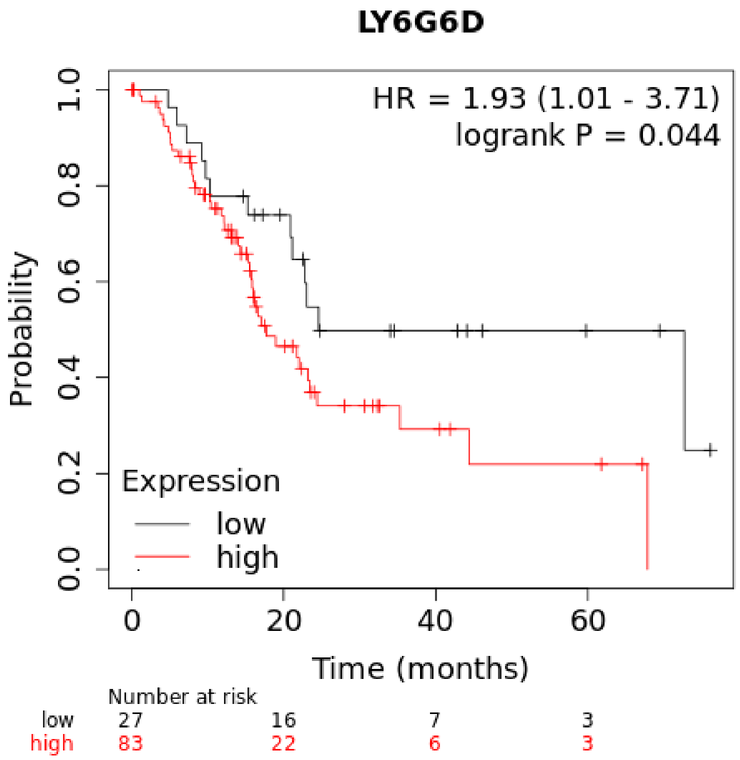

Click here to download the plot in TIFF format

Download plot as a PDF

Download p values vs. cutoff table

Median survival

Low expression cohort (months)	High expression cohort (months)
24.6	17.73

**RNAseq ID:**	LY6G6F -
**Survival:**	OS
**Auto select best cutoff:**	checked
**Follow up threshold:**	all
**Censore at threshold:**	checked
**Compute median over entire database:**	false
**Cutoff value used in analysis:**	0
**Expression range of the probe:**	0 - 5
**Invert HR values below 1:**	not checked

Restrictions

Tumor type: Pancreatic ductal adenocarcinoma

Restrict analysis to subtypes...

Stage: all
Gender: all
Race: all
Grade: all
Mutation burden: all

Restrict analysis based on cellular content...

Basophils: all
B-cells: all
CD4+ memory T-cells: all
CD8+ T-cells: all
Eosinophils: all
Macrophages: all
Mesenchymal stem cells: all
Natural killer T-cells: all
Regulatory T-cells: decreased
Type 1 T-helper cells: all
Type 2 T-helper cells: all

Results

P value: 0.0054
FDR: over 50%

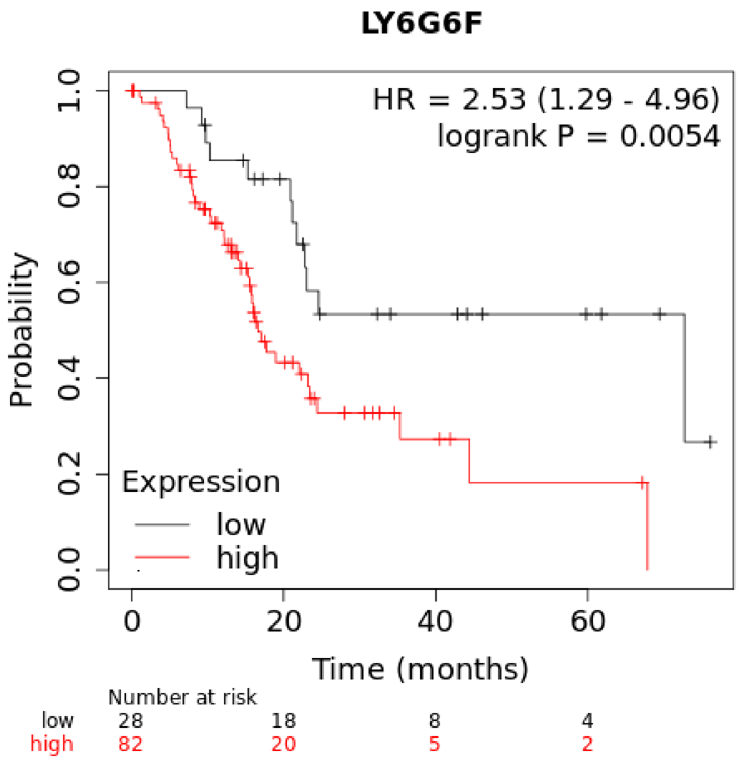

Click here to download the plot in TIFF format

Download plot as a PDF

Download p values vs. cutoff table

Median survival

Low expression cohort (months)	High expression cohort (months)
72.73	16.6

**RNAseq ID:**	LY6G5C -
**Survival:**	OS
**Auto select best cutoff:**	checked
**Follow up threshold:**	all
**Censore at threshold:**	checked
**Compute median over entire database:**	false
**Cutoff value used in analysis:**	49
**Expression range of the probe:**	15 - 586
**Invert HR values below 1:**	not checked

Restrictions

Tumor type: Pancreatic ductal adenocarcinoma

Restrict analysis to subtypes...

Stage:	all
Gender:	all
Race:	all
Grade:	all
Mutation burden:	all

Restrict analysis based on cellular content...

Basophils:	all
B-cells:	all
CD4+ memory T-cells:	all
CD8+ T-cells:	all
Eosinophils:	all
Macrophages:	all
Mesenchymal stem cells:	all
Natural killer T-cells:	all
Regulatory T-cells:	decreased
Type 1 T-helper cells:	all
Type 2 T-helper cells:	all

Results

**P value:**	0.0003
**FDR:**	5%

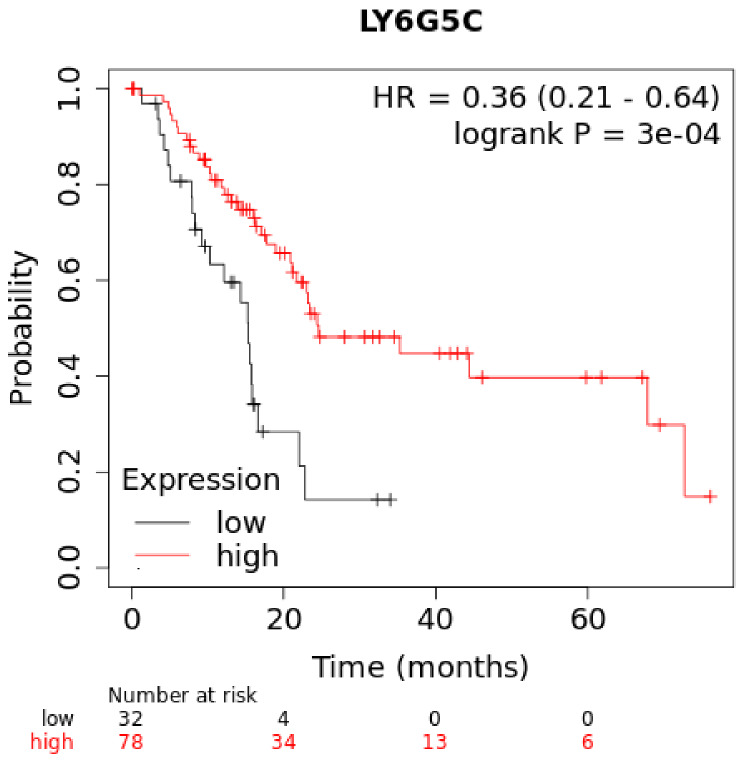

Click here to download the plot in TIFF format

Download plot as a PDF

Download p values vs. cutoff table

Median survival

Low expression cohort (months)	High expression cohort (months)
15.33	24.6

RNAseq ID: LY6G5B -
Survival: OS
Auto select best cutoff: checked
Follow up threshold: all
Censore at threshold: checked
Compute median over entire database: false
Cutoff value used in analysis: 37
Expression range of the probe: 3 - 192
Invert HR values below 1: not checked

Restrictions

Tumor type: Pancreatic ductal adenocarcinoma

Restrict analysis to subtypes...

Stage: all
Gender: all
Race: all
Grade: all
Mutation burden: all

Restrict analysis based on cellular content...

Basophils: all

B-cells:	all
CD4+ memory T-cells:	all
CD8+ T-cells:	all
Eosinophils:	all
Macrophages:	all
Mesenchymal stem cells:	all
Natural killer T-cells:	all
Regulatory T-cells:	decreased
Type 1 T-helper cells:	all
Type 2 T-helper cells:	all

Results

P value: 0.0079
FDR: over 50%

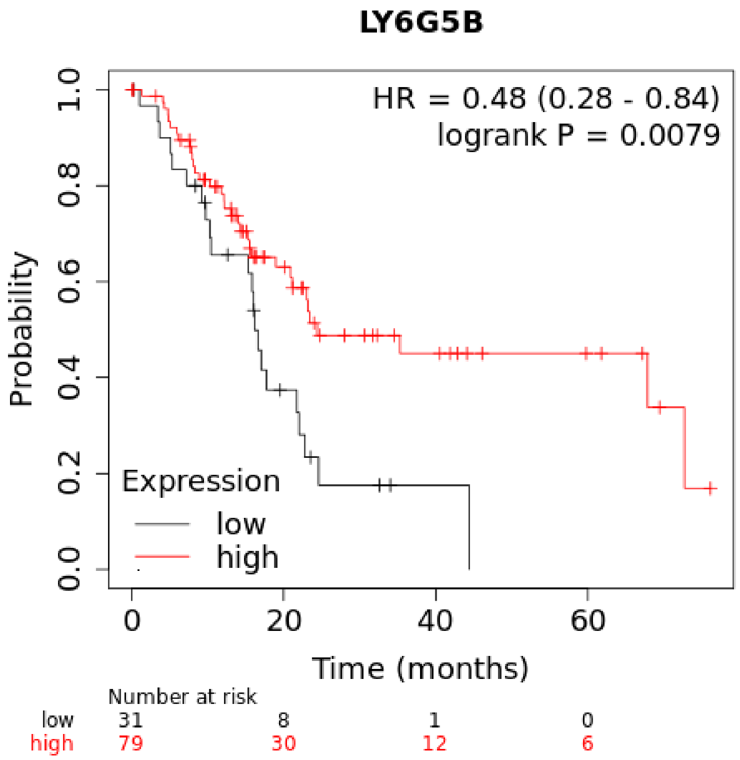

Click here to download the plot in TIFF format

Download plot as a PDF

Download p values vs. cutoff table

Median survival

Low expression cohort (months)	High expression cohort (months)
16.17	24.4

You can save the plots by right-clicking the image and then selecting "Save image as...". To generate a high resolution TIFF image, please adjust the "Settings" in the analysis page.

KMplot.com Copyright © 2009-2020 | Contact | Sitemap

Figure S4: KM plots and other raw data for the data depicted in Table 4

Pan-cancer

KM plotter | Home | Vote | Download | Updates | Contact

The desired RNAseq ID is valid: PSCA (-), LY6K (-), SLURP1 (-), LYPD2 (-), LY6D (-), GML (-), LY6E (-), LY6L (-), LY6H (-), GPIHBP1 (-), LYPD4 (-), CD177 (-), TEX101 (-), LYPD3 (-), PINLYP (-), PLAUR (-), LYPD5 (-), SPACA4 (-), ACRV1 (-), PATE1 (-), PATE2 (-), PATE3 (-), PATE4 (-), CD59 (-), LY6G6C (-), LY6G6D (-), LY6G6F (-), LY6G5C (-), LY6G5B (-),

**RNAseq ID:**	PSCA -
**Survival:**	OS
**Auto select best cutoff:**	checked
**Follow up threshold:**	all
**Censore at threshold:**	checked
**Compute median over entire database:**	false
**Cutoff value used in analysis:**	2365
**Expression range of the probe:**	0 - 31537
**Invert HR values below 1:**	not checked

Restrictions

Tumor type: Pancreatic ductal adenocarcinoma

Restrict analysis to subtypes...

Stage:	all
Gender:	all
Race:	all
Grade:	all
Mutation burden:	all

Restrict analysis based on cellular content...

Basophils:	all
B-cells:	all
CD4+ memory T-cells:	all
CD8+ T-cells:	enriched
Eosinophils:	all
Macrophages:	all
Mesenchymal stem cells:	all
Natural killer T-cells:	all
Regulatory T-cells:	all
Type 1 T-helper cells:	all
Type 2 T-helper cells:	all

Results

P value: 0.005
FDR: 50%

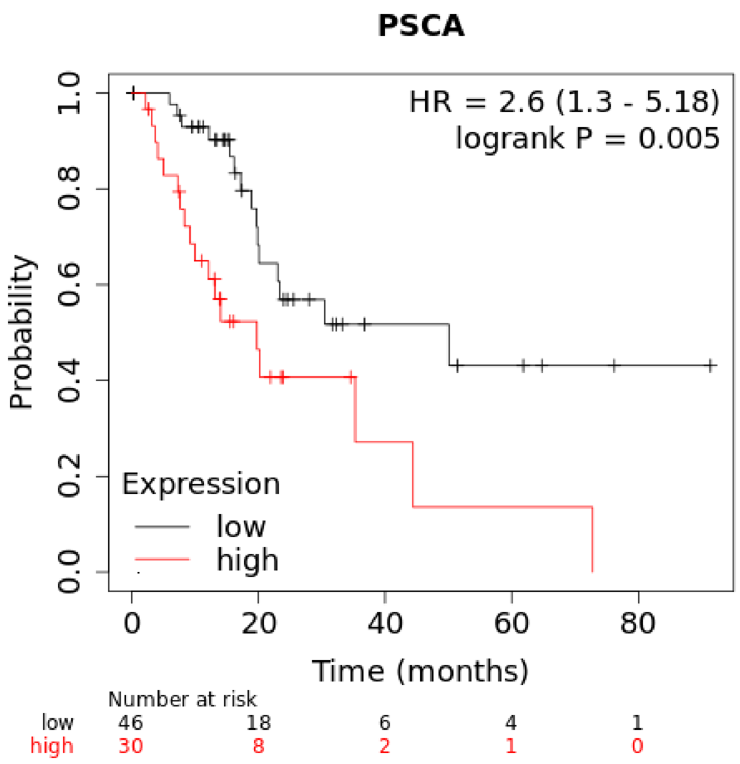

Click here to download the plot in TIFF format

Download plot as a PDF

Download p values vs. cutoff table

Median survival

Low expression cohort (months)	High expression cohort (months)
50.07	19.77

**RNAseq ID:**	LY6K -
**Survival:**	OS
**Auto select best cutoff:**	checked
**Follow up threshold:**	all
**Censore at threshold:**	checked
**Compute median over entire database:**	false
**Cutoff value used in analysis:**	5
**Expression range of the probe:**	0 - 1332
**Invert HR values below 1:**	not checked

Restrictions

Tumor type: Pancreatic ductal adenocarcinoma

Restrict analysis to subtypes...

Stage:	all
Gender:	all
Race:	all
Grade:	all
Mutation burden:	all

Restrict analysis based on cellular content...

Basophils:	all

B-cells:	all
CD4+ memory T-cells:	all
CD8+ T-cells:	enriched
Eosinophils:	all
Macrophages:	all
Mesenchymal stem cells:	all
Natural killer T-cells:	all
Regulatory T-cells:	all
Type 1 T-helper cells:	all
Type 2 T-helper cells:	all

Results

P value: 0.243
FDR: 100%

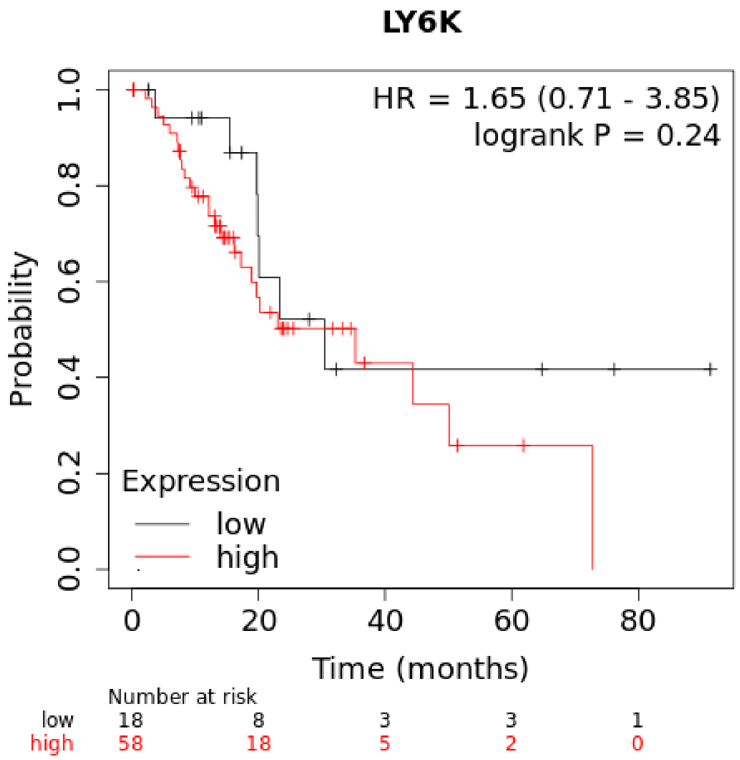

Click here to download the plot in TIFF format

Download plot as a PDF

Download p values vs. cutoff table

Median survival

Low expression cohort (months)	High expression cohort (months)
30.43	35.3

**RNAseq ID:**	SLURP1 -
**Survival:**	OS
**Auto select best cutoff:**	checked
**Follow up threshold:**	all
**Censore at threshold:**	checked
**Compute median over entire database:**	false
**Cutoff value used in analysis:**	1
**Expression range of the probe:**	0 - 150
**Invert HR values below 1:**	not checked

Restrictions

Tumor type: Pancreatic ductal adenocarcinoma

Restrict analysis to subtypes...

Stage:	all
Gender:	all
Race:	all
Grade:	all
Mutation burden:	all

Restrict analysis based on cellular content...

Basophils:	all
B-cells:	all
CD4+ memory T-cells:	all
CD8+ T-cells:	enriched
Eosinophils:	all
Macrophages:	all
Mesenchymal stem cells:	all
Natural killer T-cells:	all
Regulatory T-cells:	all
Type 1 T-helper cells:	all
Type 2 T-helper cells:	all

Results

P value: 0.0012
FDR: 20%

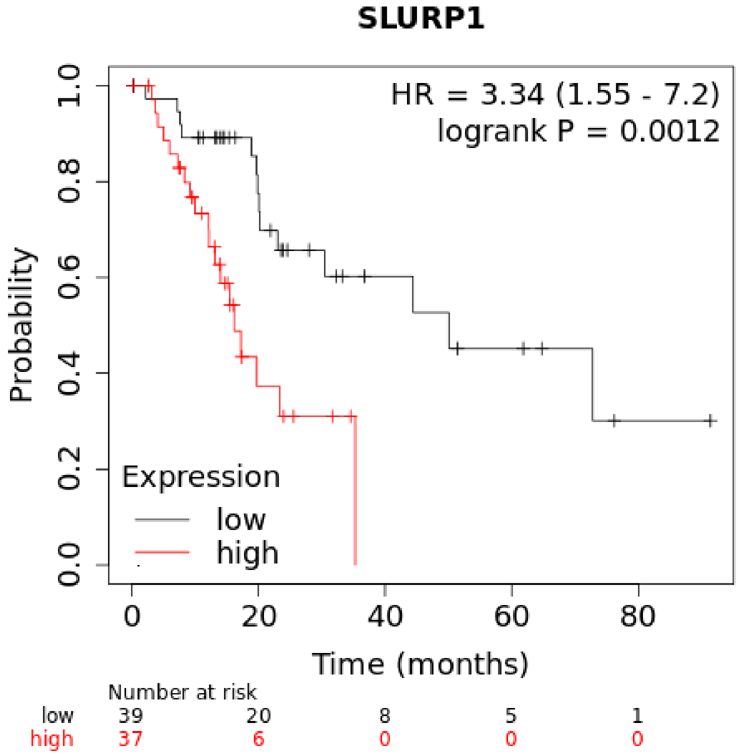

Click here to download the plot in TIFF format

Download plot as a PDF

Download p values vs. cutoff table

Median survival

Low expression cohort (months)	High expression cohort (months)
50.07	16.2

**RNAseq ID:**	LYPD2 -
**Survival:**	OS
**Auto select best cutoff:**	checked
**Follow up threshold:**	all
**Censore at threshold:**	checked
**Compute median over entire database:**	false
**Cutoff value used in analysis:**	2
**Expression range of the probe:**	0 - 2654
**Invert HR values below 1:**	not checked

Restrictions

Tumor type: Pancreatic ductal adenocarcinoma

Restrict analysis to subtypes...

Stage:	all
Gender:	all
Race:	all
Grade:	all
Mutation burden:	all

Restrict analysis based on cellular content...

Basophils:	all
B-cells:	all
CD4+ memory T-cells:	all
CD8+ T-cells:	enriched
Eosinophils:	all
Macrophages:	all
Mesenchymal stem cells:	all
Natural killer T-cells:	all
Regulatory T-cells:	all
Type 1 T-helper cells:	all
Type 2 T-helper cells:	all

Results

**P value:**	0.0199
**FDR:**	over 50%

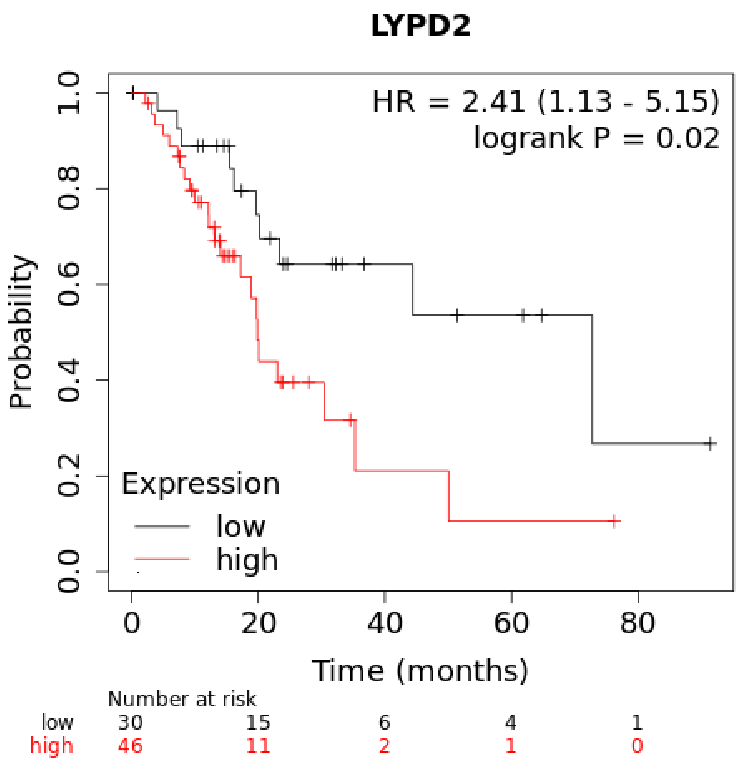

Click here to download the plot in TIFF format

Download plot as a PDF

Download p values vs. cutoff table

Median survival

Low expression cohort (months)	High expression cohort (months)
72.73	19.87

RNAseq ID: LY6D -
Survival: OS
Auto select best cutoff: checked
Follow up threshold: all
Censore at threshold: checked
Compute median over entire database: false
Cutoff value used in analysis: 181
Expression range of the probe: 0 - 8634
Invert HR values below 1: not checked

Restrictions

Tumor type: Pancreatic ductal adenocarcinoma

Restrict analysis to subtypes...

Stage: all
Gender: all
Race: all
Grade: all
Mutation burden: all

Restrict analysis based on cellular content...

Basophils: all

B-cells:	all
CD4+ memory T-cells:	all
CD8+ T-cells:	enriched
Eosinophils:	all
Macrophages:	all
Mesenchymal stem cells:	all
Natural killer T-cells:	all
Regulatory T-cells:	all
Type 1 T-helper cells:	all
Type 2 T-helper cells:	all

Results

P value: 7.4e-5
FDR: 1%

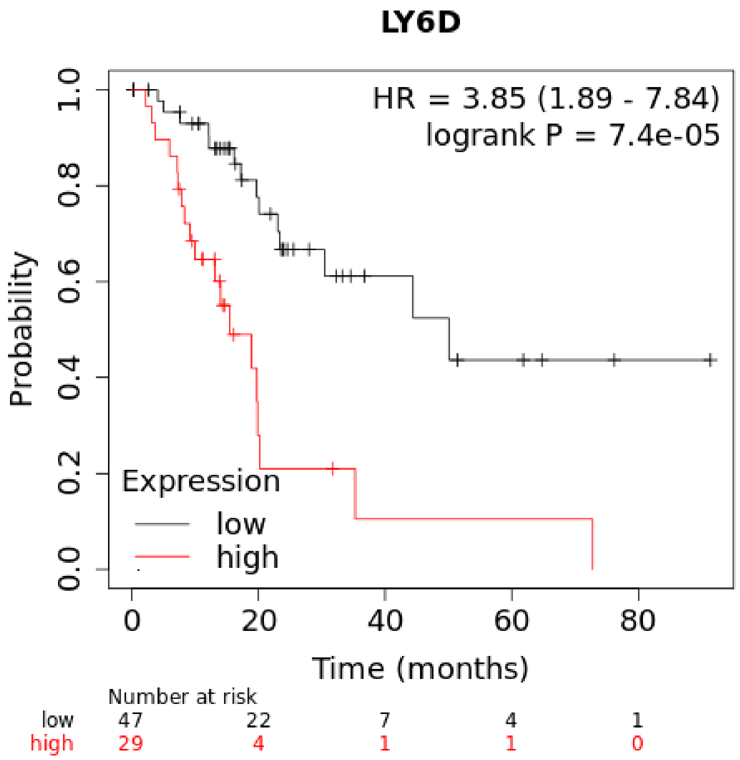

[Click here to download the plot in TIFF format]

[Download plot as a PDF]

[Download p values vs. cutoff table]

Median survival

Low expression cohort (months)	High expression cohort (months)
50.07	15.53

**RNAseq ID:**	GML -
**Survival:**	OS
**Auto select best cutoff:**	checked
**Follow up threshold:**	all
**Censore at threshold:**	checked
**Compute median over entire database:**	false
**Cutoff value used in analysis:**	0
**Expression range of the probe:**	0 - 3
**Invert HR values below 1:**	not checked

Restrictions

Tumor type: Pancreatic ductal adenocarcinoma

Restrict analysis to subtypes...

Stage:	all
Gender:	all
Race:	all
Grade:	all
Mutation burden:	all

Restrict analysis based on cellular content...

Basophils:	all
B-cells:	all
CD4+ memory T-cells:	all
CD8+ T-cells:	enriched
Eosinophils:	all
Macrophages:	all
Mesenchymal stem cells:	all
Natural killer T-cells:	all
Regulatory T-cells:	all
Type 1 T-helper cells:	all
Type 2 T-helper cells:	all

Results

P value: 0.0758
FDR: 100%

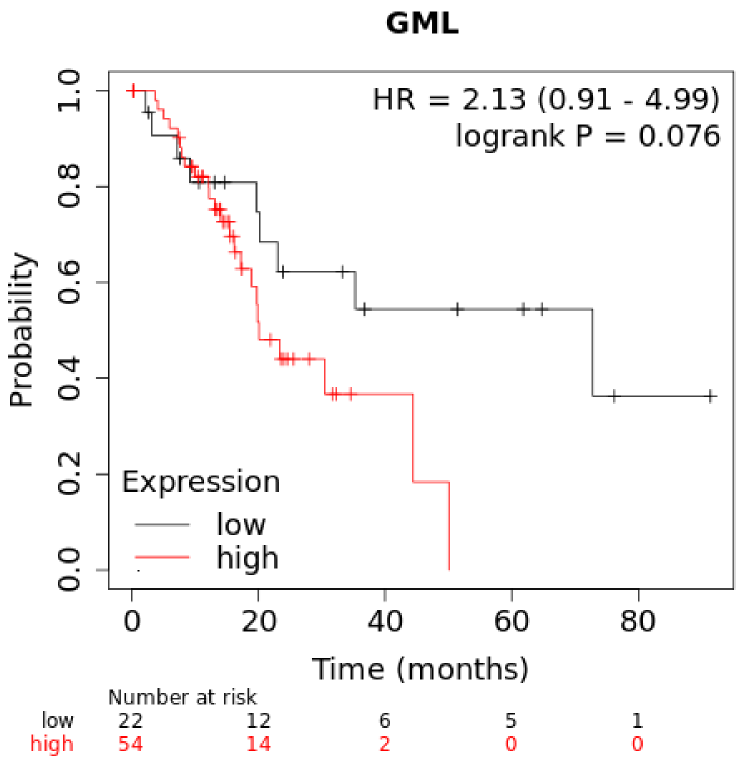

Click here to download the plot in TIFF format

Download plot as a PDF

Download p values vs. cutoff table

Median survival

Low expression cohort (months)	High expression cohort (months)
72.73	20.1

**RNAseq ID:**	LY6E -
**Survival:**	OS
**Auto select best cutoff:**	checked
**Follow up threshold:**	all
**Censore at threshold:**	checked
**Compute median over entire database:**	false
**Cutoff value used in analysis:**	5365
**Expression range of the probe:**	254 - 32064
**Invert HR values below 1:**	not checked

Restrictions

Tumor type: Pancreatic ductal adenocarcinoma

Restrict analysis to subtypes...

Stage:	all
Gender:	all
Race:	all
Grade:	all
Mutation burden:	all

Restrict analysis based on cellular content...

Basophils:	all
B-cells:	all
CD4+ memory T-cells:	all
CD8+ T-cells:	enriched
Eosinophils:	all
Macrophages:	all
Mesenchymal stem cells:	all
Natural killer T-cells:	all
Regulatory T-cells:	all
Type 1 T-helper cells:	all
Type 2 T-helper cells:	all

Results

**P value:**	0.0147
**FDR:**	50%

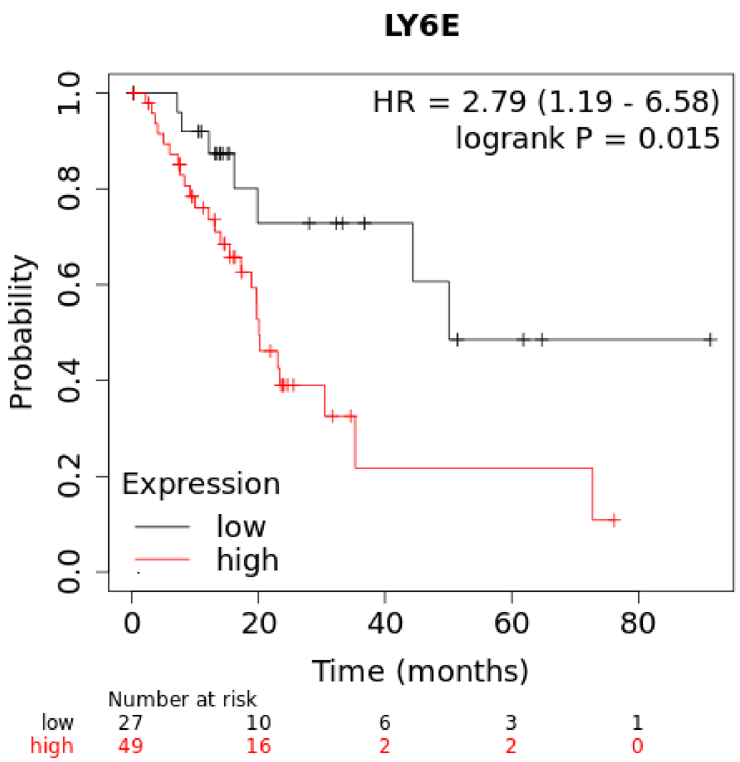

Click here to download the plot in TIFF format

Download plot as a PDF

Download p values vs. cutoff table

Median survival

Low expression cohort (months)	High expression cohort (months)
50.07	20.1

**RNAseq ID:**	LY6L -
**Survival:**	OS
**Auto select best cutoff:**	checked
**Follow up threshold:**	all
**Censore at threshold:**	checked
**Compute median over entire database:**	false
**Cutoff value used in analysis:**	0
**Expression range of the probe:**	0 - 8
**Invert HR values below 1:**	not checked

Restrictions

Tumor type: Pancreatic ductal adenocarcinoma

Restrict analysis to subtypes...

Stage:	all
Gender:	all
Race:	all
Grade:	all
Mutation burden:	all

Restrict analysis based on cellular content...

Basophils:	all

B-cells:	all
CD4+ memory T-cells:	all
CD8+ T-cells:	enriched
Eosinophils:	all
Macrophages:	all
Mesenchymal stem cells:	all
Natural killer T-cells:	all
Regulatory T-cells:	all
Type 1 T-helper cells:	all
Type 2 T-helper cells:	all

Results

P value: 0.049
FDR: over 50%

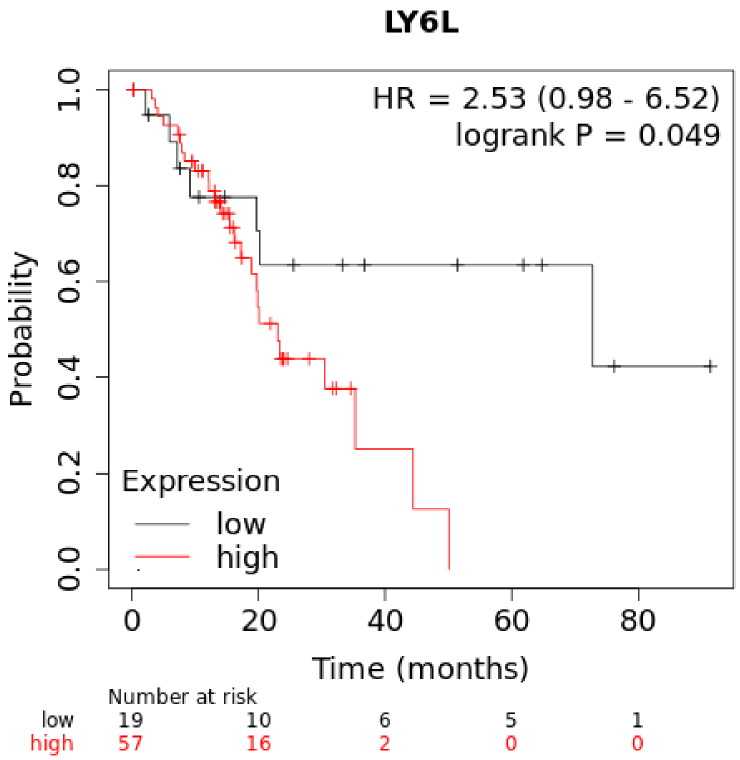

[Click here to download the plot in TIFF format]

[Download plot as a PDF]

[Download p values vs. cutoff table]

Median survival

Low expression cohort (months)	High expression cohort (months)
72.73	23.03

**RNAseq ID:**	LY6H -
**Survival:**	OS
**Auto select best cutoff:**	checked
**Follow up threshold:**	all
**Censore at threshold:**	checked
**Compute median over entire database:**	false
**Cutoff value used in analysis:**	63
**Expression range of the probe:**	5 - 9495
**Invert HR values below 1:**	not checked

Restrictions

Tumor type: Pancreatic ductal adenocarcinoma

Restrict analysis to subtypes...

Stage:	all
Gender:	all
Race:	all
Grade:	all
Mutation burden:	all

Restrict analysis based on cellular content...

Basophils:	all
B-cells:	all
CD4+ memory T-cells:	all
CD8+ T-cells:	enriched
Eosinophils:	all
Macrophages:	all
Mesenchymal stem cells:	all
Natural killer T-cells:	all
Regulatory T-cells:	all
Type 1 T-helper cells:	all
Type 2 T-helper cells:	all

Results

P value: 0.0189
FDR: over 50%

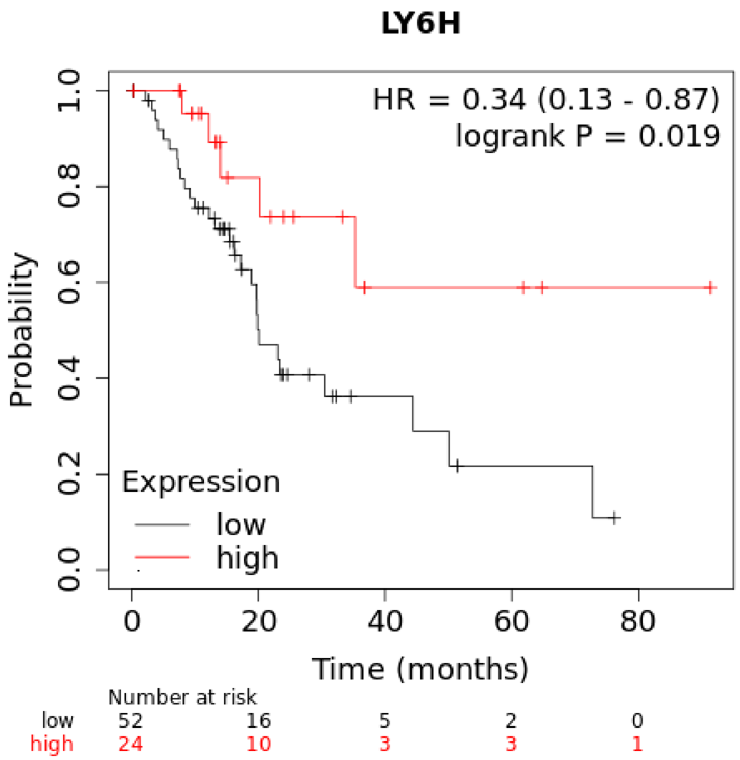

Click here to download the plot in TIFF format

Download plot as a PDF

Download p values vs. cutoff table

Upper quartile survival

Low expression cohort (months)	High expression cohort (months)
12.2	20.23

**RNAseq ID:**	GPIHBP1 -
**Survival:**	OS
**Auto select best cutoff:**	checked
**Follow up threshold:**	all
**Censore at threshold:**	checked
**Compute median over entire database:**	false
**Cutoff value used in analysis:**	42
**Expression range of the probe:**	12 - 323
**Invert HR values below 1:**	not checked

Restrictions

Tumor type: Pancreatic ductal adenocarcinoma

Restrict analysis to subtypes...

Stage:	all
Gender:	all
Race:	all
Grade:	all
Mutation burden:	all

Restrict analysis based on cellular content...

Basophils:	all
B-cells:	all
CD4+ memory T-cells:	all
CD8+ T-cells:	enriched
Eosinophils:	all
Macrophages:	all
Mesenchymal stem cells:	all
Natural killer T-cells:	all
Regulatory T-cells:	all
Type 1 T-helper cells:	all
Type 2 T-helper cells:	all

Results

**P value:**	0.1824
**FDR:**	100%

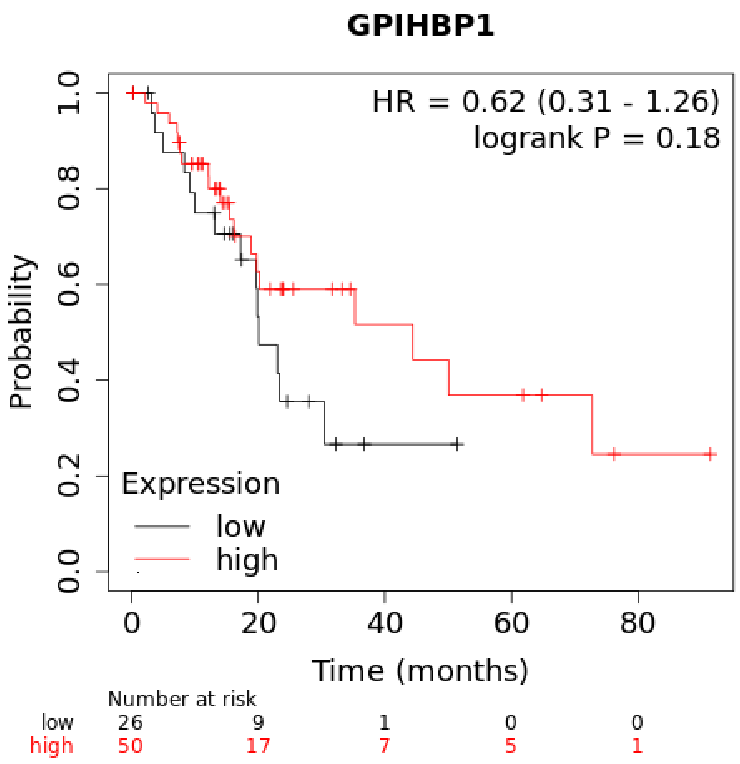

Click here to download the plot in TIFF format

Download plot as a PDF

Download p values vs. cutoff table

Median survival

Low expression cohort (months)	High expression cohort (months)
20.1	44.4

**RNAseq ID:**	LYPD4 -
**Survival:**	OS
**Auto select best cutoff:**	checked
**Follow up threshold:**	all
**Censore at threshold:**	checked
**Compute median over entire database:**	false
**Cutoff value used in analysis:**	0
**Expression range of the probe:**	0 - 4
**Invert HR values below 1:**	not checked

Restrictions

Tumor type: Pancreatic ductal adenocarcinoma

Restrict analysis to subtypes...

Stage:	all
Gender:	all
Race:	all
Grade:	all
Mutation burden:	all

Restrict analysis based on cellular content...

Basophils:	all

B-cells:	all
CD4+ memory T-cells:	all
CD8+ T-cells:	enriched
Eosinophils:	all
Macrophages:	all
Mesenchymal stem cells:	all
Natural killer T-cells:	all
Regulatory T-cells:	all
Type 1 T-helper cells:	all
Type 2 T-helper cells:	all

Results

P value: 0.2133
FDR: 100%

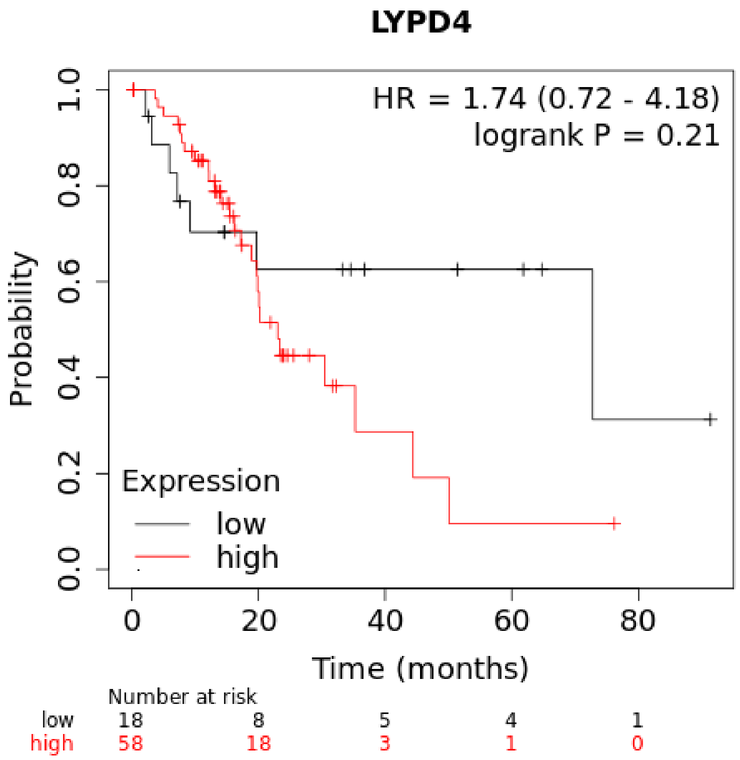

Click here to download the plot in TIFF format

Download plot as a PDF

Download p values vs. cutoff table

Median survival

Low expression cohort (months)	High expression cohort (months)
72.73	23.03

**RNAseq ID:**	CD177 -
**Survival:**	OS
**Auto select best cutoff:**	checked
**Follow up threshold:**	all
**Censore at threshold:**	checked
**Compute median over entire database:**	false
**Cutoff value used in analysis:**	14
**Expression range of the probe:**	0 - 6100
**Invert HR values below 1:**	not checked

Restrictions

Tumor type: Pancreatic ductal adenocarcinoma

Restrict analysis to subtypes...

Stage:	all
Gender:	all
Race:	all
Grade:	all
Mutation burden:	all

Restrict analysis based on cellular content...

Basophils:	all
B-cells:	all
CD4+ memory T-cells:	all
CD8+ T-cells:	enriched
Eosinophils:	all
Macrophages:	all
Mesenchymal stem cells:	all
Natural killer T-cells:	all
Regulatory T-cells:	all
Type 1 T-helper cells:	all
Type 2 T-helper cells:	all

Results

P value: 0.0233
FDR: over 50%

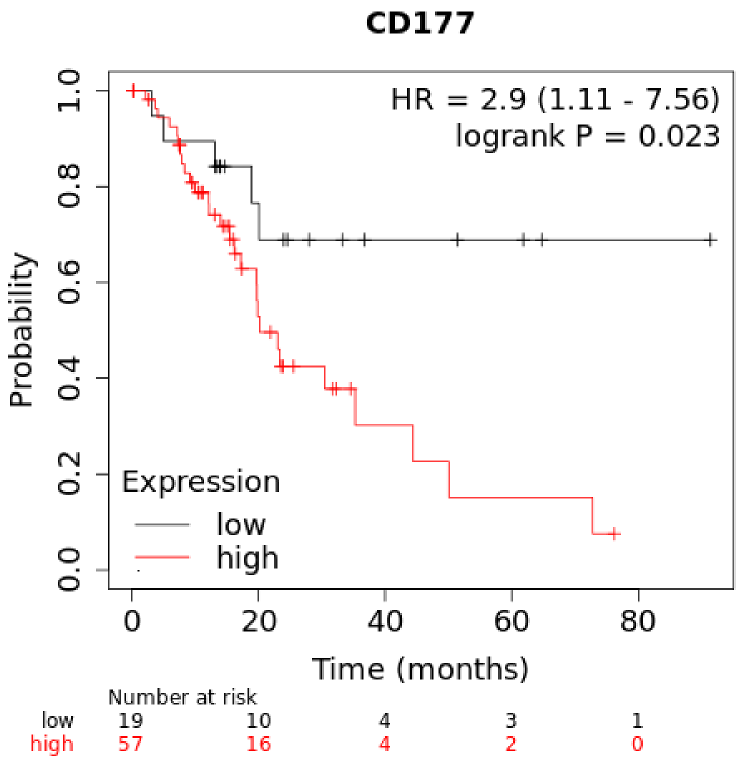

Click here to download the plot in TIFF format

Download plot as a PDF

Download p values vs. cutoff table

Upper quartile survival

Low expression cohort (months)	High expression cohort (months)
20.1	12.2

**RNAseq ID:**	TEX101 -
**Survival:**	OS
**Auto select best cutoff:**	checked
**Follow up threshold:**	all
**Censore at threshold:**	checked
**Compute median over entire database:**	false
**Cutoff value used in analysis:**	1
**Expression range of the probe:**	0 - 24
**Invert HR values below 1:**	not checked

Restrictions

Tumor type: Pancreatic ductal adenocarcinoma

Restrict analysis to subtypes...

Stage:	all
Gender:	all
Race:	all
Grade:	all
Mutation burden:	all

Restrict analysis based on cellular content...

Basophils:	all
B-cells:	all
CD4+ memory T-cells:	all
CD8+ T-cells:	enriched
Eosinophils:	all
Macrophages:	all
Mesenchymal stem cells:	all
Natural killer T-cells:	all
Regulatory T-cells:	all
Type 1 T-helper cells:	all
Type 2 T-helper cells:	all

Results

**P value:**	0.158
**FDR:**	100%

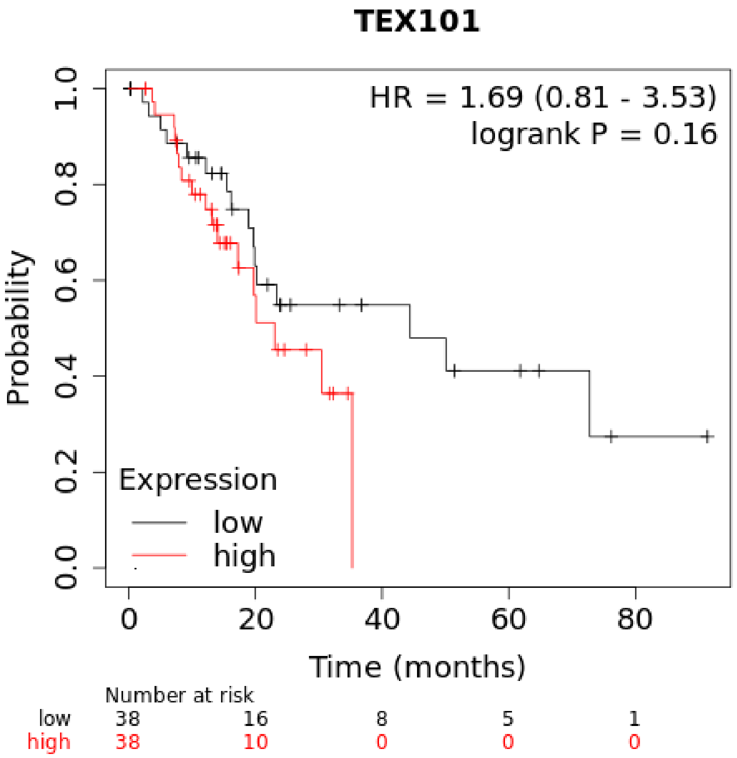

Click here to download the plot in TIFF format

Download plot as a PDF

Download p values vs. cutoff table

Median survival

Low expression cohort (months)	High expression cohort (months)
44.4	23.03

**RNAseq ID:**	LYPD3 -
**Survival:**	OS
**Auto select best cutoff:**	checked
**Follow up threshold:**	all
**Censore at threshold:**	checked
**Compute median over entire database:**	false
**Cutoff value used in analysis:**	148
**Expression range of the probe:**	16 - 1869
**Invert HR values below 1:**	not checked

Restrictions

Tumor type: Pancreatic ductal adenocarcinoma

Restrict analysis to subtypes...

Stage:	all
Gender:	all
Race:	all
Grade:	all
Mutation burden:	all

Restrict analysis based on cellular content...

Basophils: all

B-cells:	all
CD4+ memory T-cells:	all
CD8+ T-cells:	enriched
Eosinophils:	all
Macrophages:	all
Mesenchymal stem cells:	all
Natural killer T-cells:	all
Regulatory T-cells:	all
Type 1 T-helper cells:	all
Type 2 T-helper cells:	all

Results

P value: 0.0092
FDR: 50%

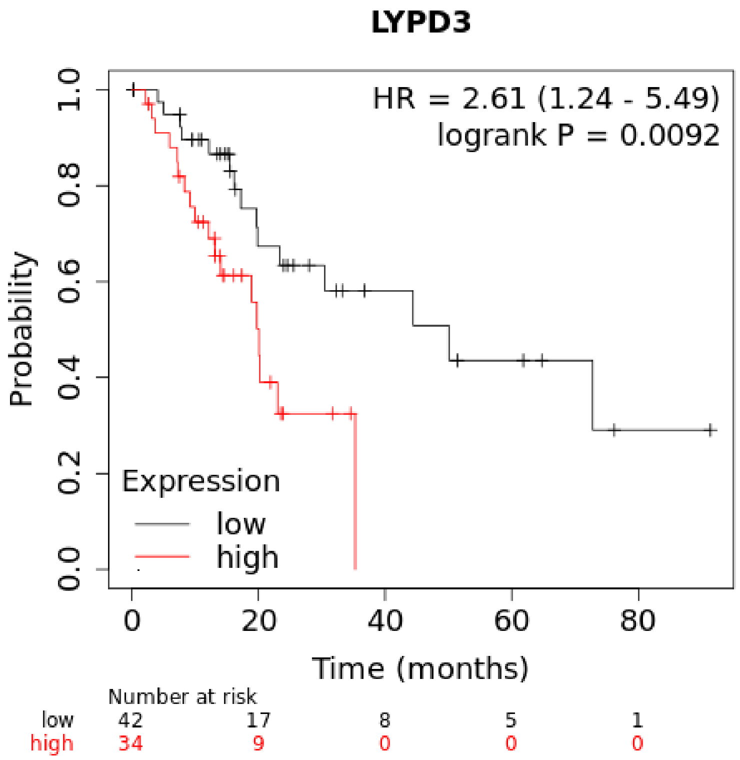

Click here to download the plot in TIFF format

Download plot as a PDF

Download p values vs. cutoff table

Median survival

Low expression cohort (months)	High expression cohort (months)
50.07	20.1

**RNAseq ID:**	PINLYP -
**Survival:**	OS
**Auto select best cutoff:**	checked
**Follow up threshold:**	all
**Censore at threshold:**	checked
**Compute median over entire database:**	false
**Cutoff value used in analysis:**	76
**Expression range of the probe:**	16 - 249
**Invert HR values below 1:**	not checked

Restrictions

Tumor type: Pancreatic ductal adenocarcinoma

Restrict analysis to subtypes...

Stage:	all
Gender:	all
Race:	all
Grade:	all
Mutation burden:	all

Restrict analysis based on cellular content...

Basophils:	all
B-cells:	all
CD4+ memory T-cells:	all
CD8+ T-cells:	enriched
Eosinophils:	all
Macrophages:	all
Mesenchymal stem cells:	all
Natural killer T-cells:	all
Regulatory T-cells:	all
Type 1 T-helper cells:	all
Type 2 T-helper cells:	all

Results

P value: 0.1735
FDR: 100%

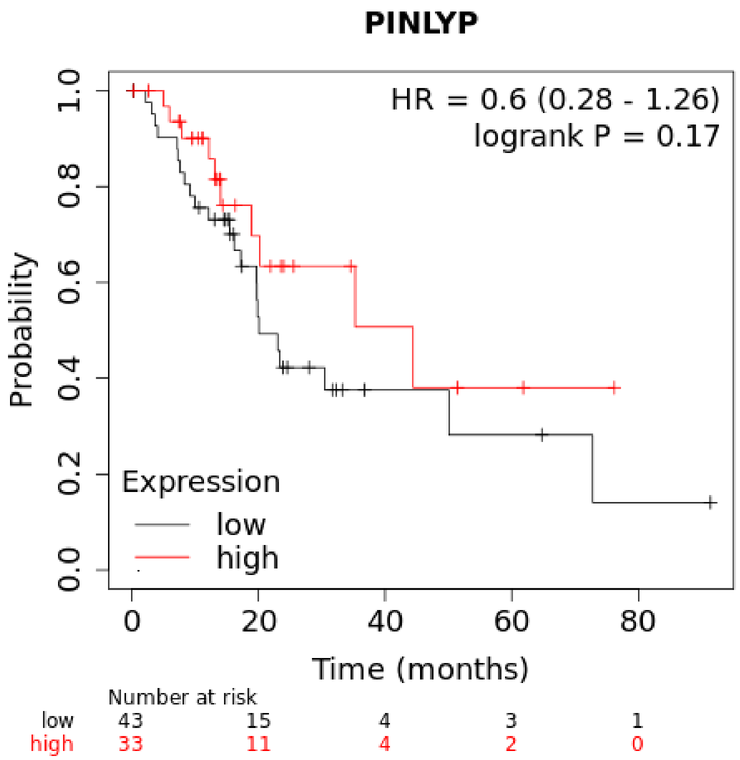

Click here to download the plot in TIFF format

Download plot as a PDF

Download p values vs. cutoff table

Median survival

Low expression cohort (months)	High expression cohort (months)
20.1	44.4

**RNAseq ID:**	PLAUR -
**Survival:**	OS
**Auto select best cutoff:**	checked
**Follow up threshold:**	all
**Censore at threshold:**	checked
**Compute median over entire database:**	false
**Cutoff value used in analysis:**	1325
**Expression range of the probe:**	47 - 7680
**Invert HR values below 1:**	not checked

Restrictions

Tumor type: Pancreatic ductal adenocarcinoma

Restrict analysis to subtypes...

Stage:	all
Gender:	all
Race:	all
Grade:	all
Mutation burden:	all

Restrict analysis based on cellular content...

Basophils:	all
B-cells:	all
CD4+ memory T-cells:	all
CD8+ T-cells:	enriched
Eosinophils:	all
Macrophages:	all
Mesenchymal stem cells:	all
Natural killer T-cells:	all
Regulatory T-cells:	all
Type 1 T-helper cells:	all
Type 2 T-helper cells:	all

Results

**P value:**	0.01
**FDR:**	over 50%

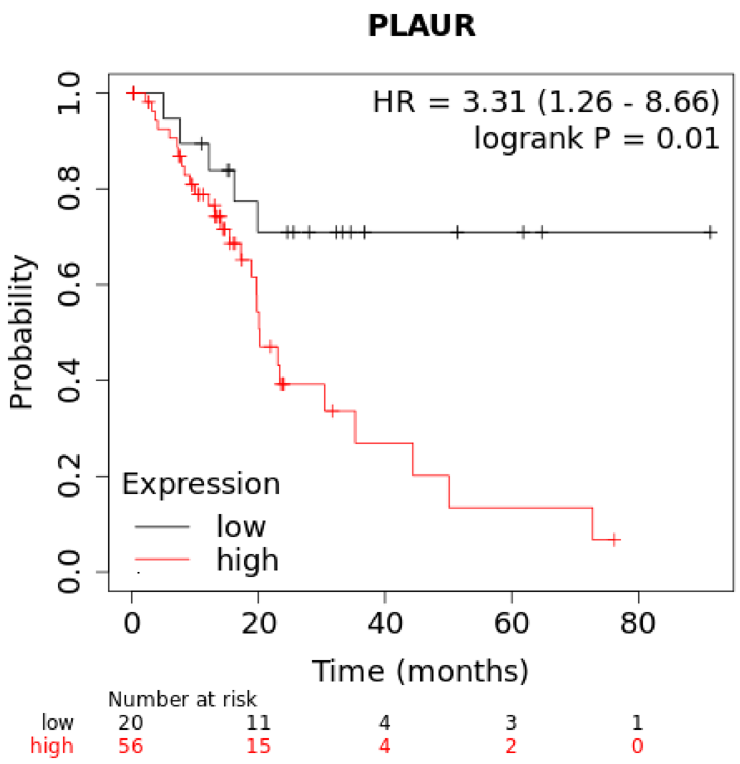

Click here to download the plot in TIFF format

Download plot as a PDF

Download p values vs. cutoff table

Upper quartile survival

Low expression cohort (months)	High expression cohort (months)
19.87	13.1

**RNAseq ID:**	LYPD5 -
**Survival:**	OS
**Auto select best cutoff:**	checked
**Follow up threshold:**	all
**Censore at threshold:**	checked
**Compute median over entire database:**	false
**Cutoff value used in analysis:**	73
**Expression range of the probe:**	1 - 578
**Invert HR values below 1:**	not checked

Restrictions

Tumor type: Pancreatic ductal adenocarcinoma

Restrict analysis to subtypes...

Stage:	all
Gender:	all
Race:	all
Grade:	all
Mutation burden:	all

Restrict analysis based on cellular content...

Basophils:	all

B-cells:	all
CD4+ memory T-cells:	all
CD8+ T-cells:	enriched
Eosinophils:	all
Macrophages:	all
Mesenchymal stem cells:	all
Natural killer T-cells:	all
Regulatory T-cells:	all
Type 1 T-helper cells:	all
Type 2 T-helper cells:	all

Results

P value: 0.0016
FDR: 10%

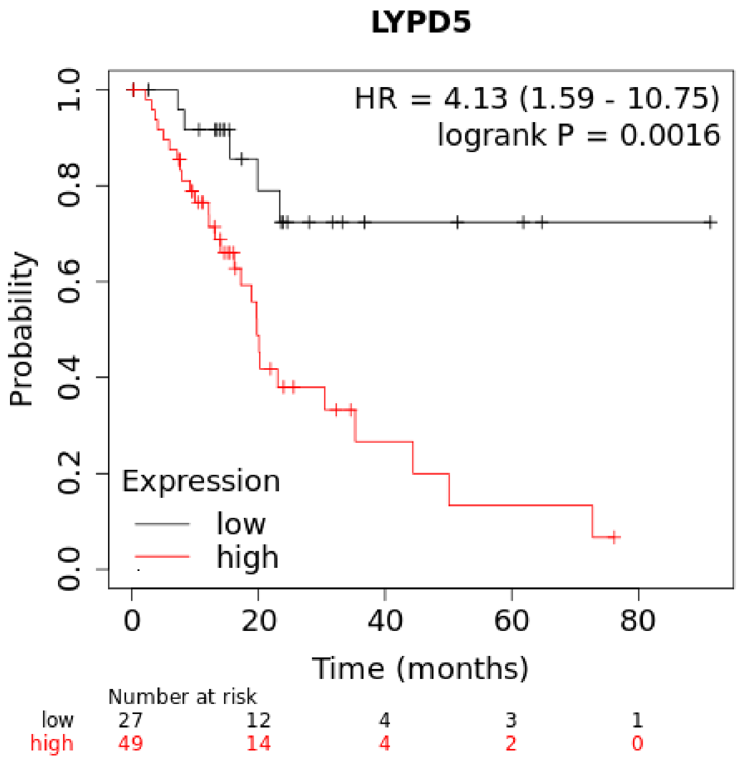

Click here to download the plot in TIFF format

Download plot as a PDF

Download p values vs. cutoff table

Upper quartile survival

Low expression cohort (months)	High expression cohort (months)
23.4	12.17

**RNAseq ID:**	SPACA4 -
**Survival:**	OS
**Auto select best cutoff:**	checked
**Follow up threshold:**	all
**Censore at threshold:**	checked
**Compute median over entire database:**	false
**Cutoff value used in analysis:**	26
**Expression range of the probe:**	0 - 204
**Invert HR values below 1:**	not checked

Restrictions

Tumor type: Pancreatic ductal adenocarcinoma

Restrict analysis to subtypes...

Stage:	all
Gender:	all
Race:	all
Grade:	all
Mutation burden:	all

Restrict analysis based on cellular content...

Basophils:	all
B-cells:	all
CD4+ memory T-cells:	all
CD8+ T-cells:	enriched
Eosinophils:	all
Macrophages:	all
Mesenchymal stem cells:	all
Natural killer T-cells:	all
Regulatory T-cells:	all
Type 1 T-helper cells:	all
Type 2 T-helper cells:	all

Results

P value: 0.1872
FDR: 100%

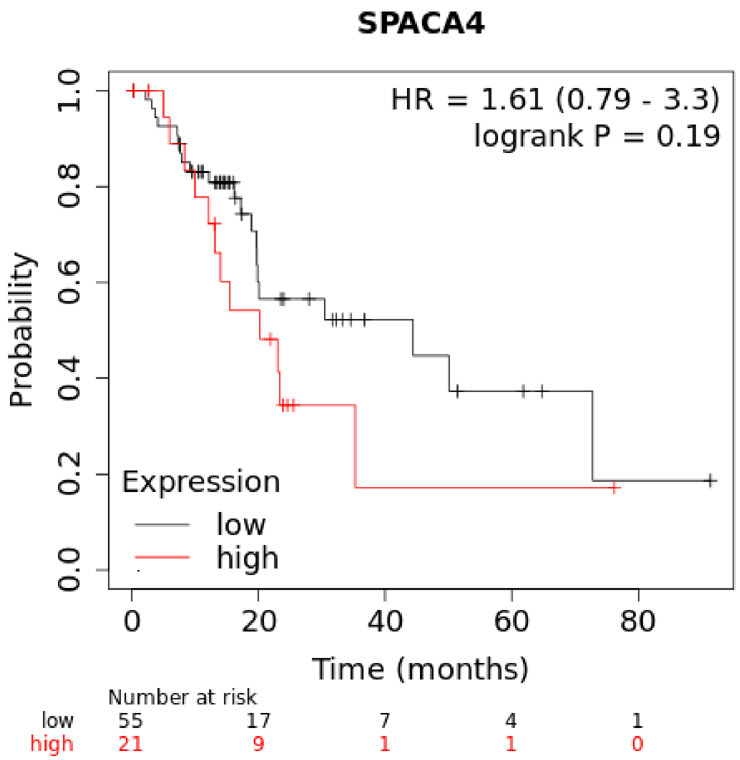

[Click here to download the plot in TIFF format]

[Download plot as a PDF]

[Download p values vs. cutoff table]

Median survival

Low expression cohort (months)	High expression cohort (months)
44.4	20.23

**RNAseq ID:**	ACRV1 -
**Survival:**	OS
**Auto select best cutoff:**	checked
**Follow up threshold:**	all
**Censore at threshold:**	checked
**Compute median over entire database:**	false
**Cutoff value used in analysis:**	3
**Expression range of the probe:**	0 - 15
**Invert HR values below 1:**	not checked

Restrictions

Tumor type: Pancreatic ductal adenocarcinoma

Restrict analysis to subtypes...

Stage:	all
Gender:	all
Race:	all
Grade:	all
Mutation burden:	all

Restrict analysis based on cellular content...

Basophils:	all
B-cells:	all
CD4+ memory T-cells:	all
CD8+ T-cells:	enriched
Eosinophils:	all
Macrophages:	all
Mesenchymal stem cells:	all
Natural killer T-cells:	all
Regulatory T-cells:	all
Type 1 T-helper cells:	all
Type 2 T-helper cells:	all

Results

**P value:**	0.093
**FDR:**	100%

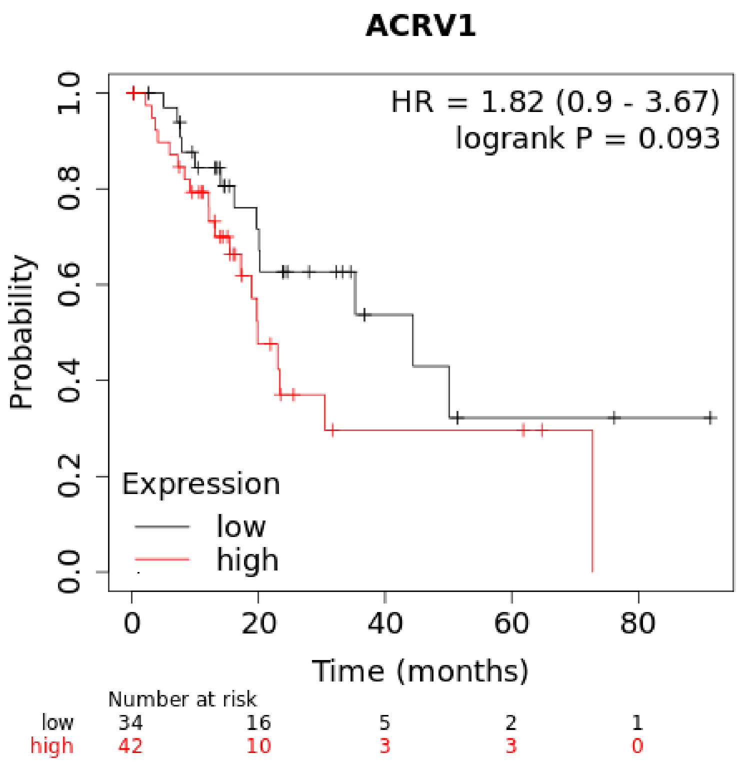

[Click here to download the plot in TIFF format]

[Download plot as a PDF]

[Download p values vs. cutoff table]

Median survival

Low expression cohort (months)	High expression cohort (months)
44.4	19.87

**RNAseq ID:**	PATE1 -
**Survival:**	OS
**Auto select best cutoff:**	checked
**Follow up threshold:**	all
**Censore at threshold:**	checked
**Compute median over entire database:**	false
**Cutoff value used in analysis:**	0
**Expression range of the probe:**	0 - 1
**Invert HR values below 1:**	not checked

Restrictions

Tumor type: Pancreatic ductal adenocarcinoma

Restrict analysis to subtypes...

Stage:	all
Gender:	all
Race:	all
Grade:	all
Mutation burden:	all

Restrict analysis based on cellular content...

Basophils:	all

B-cells:	all
CD4+ memory T-cells:	all
CD8+ T-cells:	enriched
Eosinophils:	all
Macrophages:	all
Mesenchymal stem cells:	all
Natural killer T-cells:	all
Regulatory T-cells:	all
Type 1 T-helper cells:	all
Type 2 T-helper cells:	all

Results

P value: 0.0095
FDR: over 50%

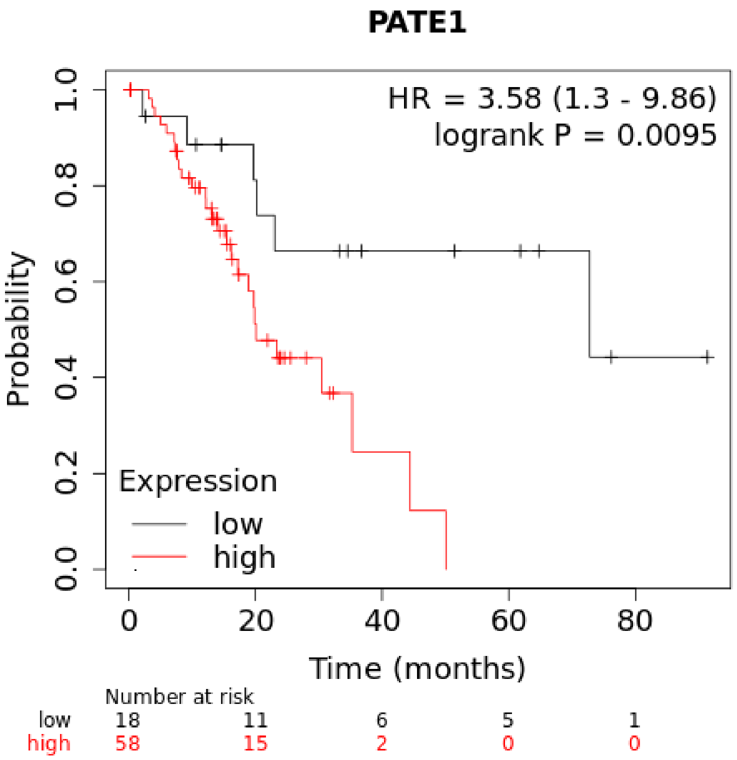

Click here to download the plot in TIFF format

Download plot as a PDF

Download p values vs. cutoff table

Median survival

Low expression cohort (months)	High expression cohort (months)
72.73	20.1

**RNAseq ID:**	PATE2 -
**Survival:**	OS
**Auto select best cutoff:**	checked
**Follow up threshold:**	all
**Censore at threshold:**	checked
**Compute median over entire database:**	false
**Cutoff value used in analysis:**	1
**Expression range of the probe:**	0 - 3
**Invert HR values below 1:**	not checked

Restrictions

Tumor type: Pancreatic ductal adenocarcinoma

Restrict analysis to subtypes...

Stage:	all
Gender:	all
Race:	all
Grade:	all
Mutation burden:	all

Restrict analysis based on cellular content...

Basophils:	all
B-cells:	all
CD4+ memory T-cells:	all
CD8+ T-cells:	enriched
Eosinophils:	all
Macrophages:	all
Mesenchymal stem cells:	all
Natural killer T-cells:	all
Regulatory T-cells:	all
Type 1 T-helper cells:	all
Type 2 T-helper cells:	all

Results

P value: 0.0013
FDR: 20%

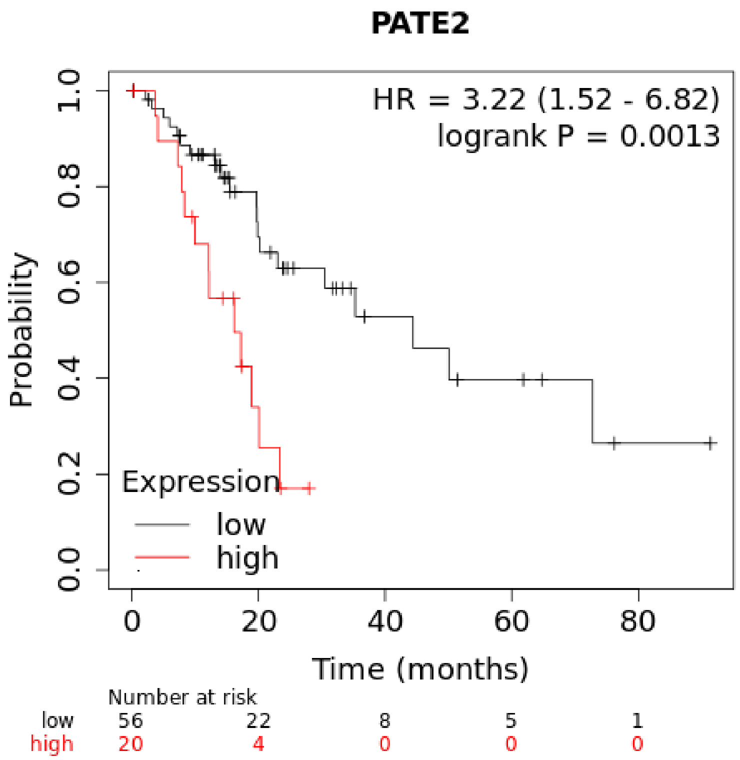

Click here to download the plot in TIFF format

Download plot as a PDF

Download p values vs. cutoff table

Median survival

Low expression cohort (months)	High expression cohort (months)
44.4	16.2

**RNAseq ID:**	PATE3 -
**Survival:**	OS
**Auto select best cutoff:**	checked
**Follow up threshold:**	all
**Censore at threshold:**	checked
**Compute median over entire database:**	false
**Cutoff value used in analysis:**	0
**Expression range of the probe:**	0 - 1
**Invert HR values below 1:**	not checked

Restrictions

Tumor type: Pancreatic ductal adenocarcinoma

Restrict analysis to subtypes...

Stage:	all
Gender:	all
Race:	all
Grade:	all
Mutation burden:	all

Restrict analysis based on cellular content...

Basophils:	all
B-cells:	all
CD4+ memory T-cells:	all
CD8+ T-cells:	enriched
Eosinophils:	all
Macrophages:	all
Mesenchymal stem cells:	all
Natural killer T-cells:	all
Regulatory T-cells:	all
Type 1 T-helper cells:	all
Type 2 T-helper cells:	all

Results

**P value:**	0.0157
**FDR:**	over 50%

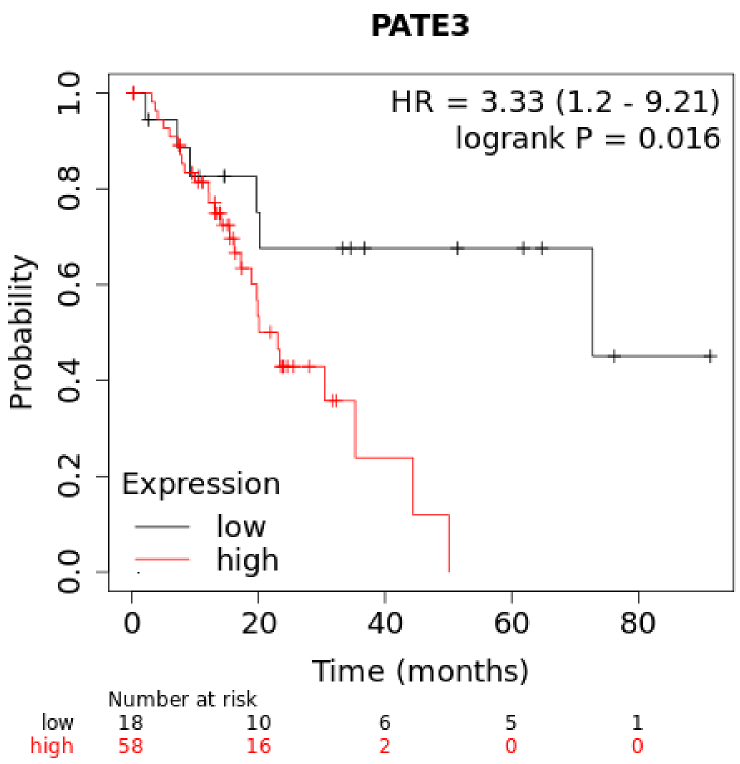

Click here to download the plot in TIFF format

Download plot as a PDF

Download p values vs. cutoff table

Median survival

Low expression cohort (months)	High expression cohort (months)
72.73	23.03

**RNAseq ID:**	PATE4 -
**Survival:**	OS
**Auto select best cutoff:**	checked
**Follow up threshold:**	all
**Censore at threshold:**	checked
**Compute median over entire database:**	false
**Cutoff value used in analysis:**	1
**Expression range of the probe:**	0 - 2
**Invert HR values below 1:**	not checked

Restrictions

Tumor type: Pancreatic ductal adenocarcinoma

Restrict analysis to subtypes...

Stage:	all
Gender:	all
Race:	all
Grade:	all
Mutation burden:	all

Restrict analysis based on cellular content...

Basophils:	all

B-cells:	all
CD4+ memory T-cells:	all
CD8+ T-cells:	enriched
Eosinophils:	all
Macrophages:	all
Mesenchymal stem cells:	all
Natural killer T-cells:	all
Regulatory T-cells:	all
Type 1 T-helper cells:	all
Type 2 T-helper cells:	all

Results

P value: 0.0598
FDR: 100%

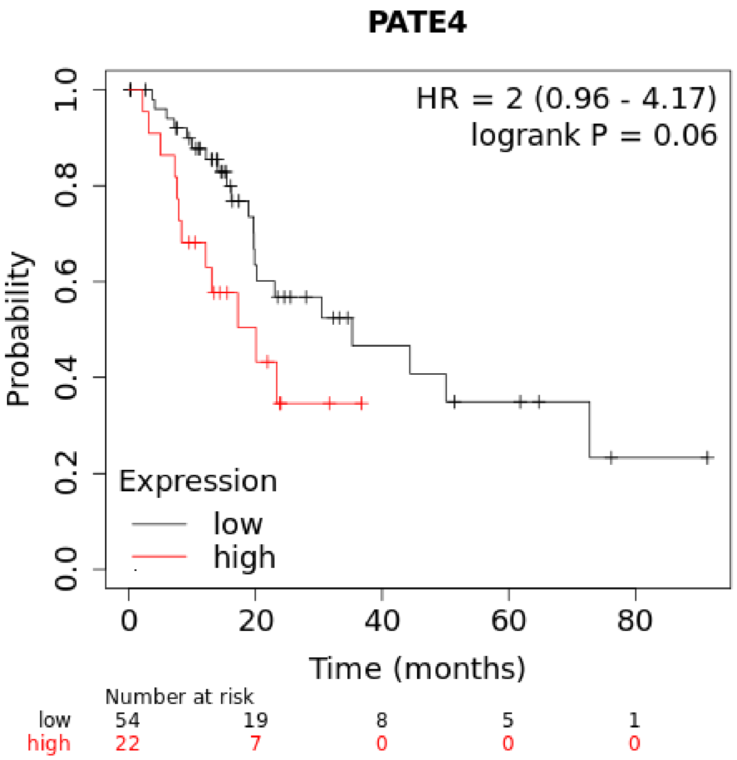

[Click here to download the plot in TIFF format]

[Download plot as a PDF]

[Download p values vs. cutoff table]

Median survival

Low expression cohort (months)	High expression cohort (months)
35.3	20.1

**RNAseq ID:**	CD59 -
**Survival:**	OS
**Auto select best cutoff:**	checked
**Follow up threshold:**	all
**Censore at threshold:**	checked
**Compute median over entire database:**	false
**Cutoff value used in analysis:**	16313
**Expression range of the probe:**	5163 - 32118
**Invert HR values below 1:**	not checked

Restrictions

Tumor type: Pancreatic ductal adenocarcinoma

Restrict analysis to subtypes...

Stage:	all
Gender:	all
Race:	all
Grade:	all
Mutation burden:	all

Restrict analysis based on cellular content...

Basophils:	all
B-cells:	all
CD4+ memory T-cells:	all
CD8+ T-cells:	enriched
Eosinophils:	all
Macrophages:	all
Mesenchymal stem cells:	all
Natural killer T-cells:	all
Regulatory T-cells:	all
Type 1 T-helper cells:	all
Type 2 T-helper cells:	all

Results

P value: 0.102
FDR: 100%

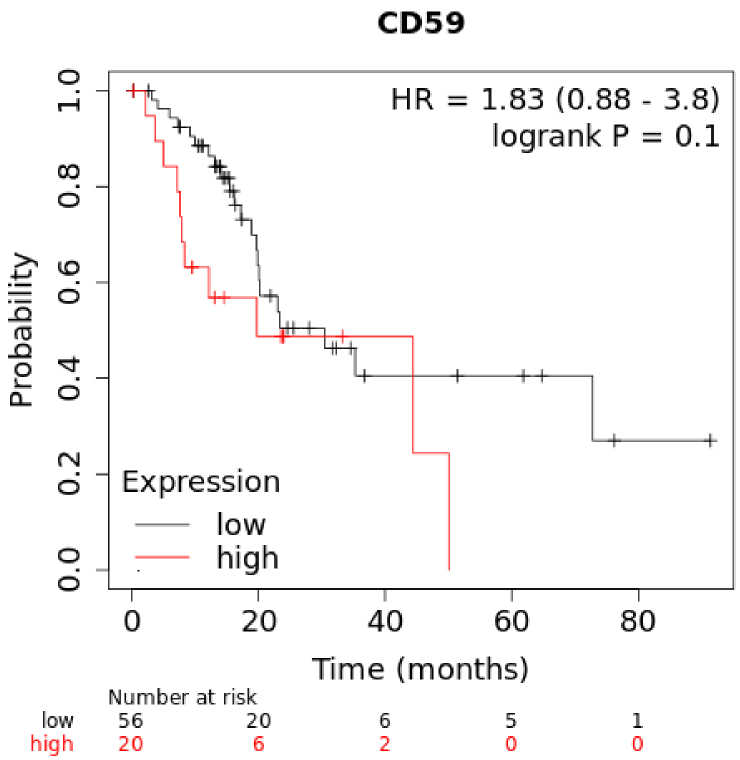

Click here to download the plot in TIFF format

Download plot as a PDF

Download p values vs. cutoff table

Median survival

Low expression cohort (months)	High expression cohort (months)
30.43	19.77

**RNAseq ID:**	LY6G6C -
**Survival:**	OS
**Auto select best cutoff:**	checked
**Follow up threshold:**	all
**Censore at threshold:**	checked
**Compute median over entire database:**	false
**Cutoff value used in analysis:**	1
**Expression range of the probe:**	0 - 251
**Invert HR values below 1:**	not checked

Restrictions

Tumor type: Pancreatic ductal adenocarcinoma

Restrict analysis to subtypes...

Stage:	all
Gender:	all
Race:	all
Grade:	all
Mutation burden:	all

Restrict analysis based on cellular content...

Basophils:	all
B-cells:	all
CD4+ memory T-cells:	all
CD8+ T-cells:	enriched
Eosinophils:	all
Macrophages:	all
Mesenchymal stem cells:	all
Natural killer T-cells:	all
Regulatory T-cells:	all
Type 1 T-helper cells:	all
Type 2 T-helper cells:	all

Results

**P value:**	0.0568
**FDR:**	100%

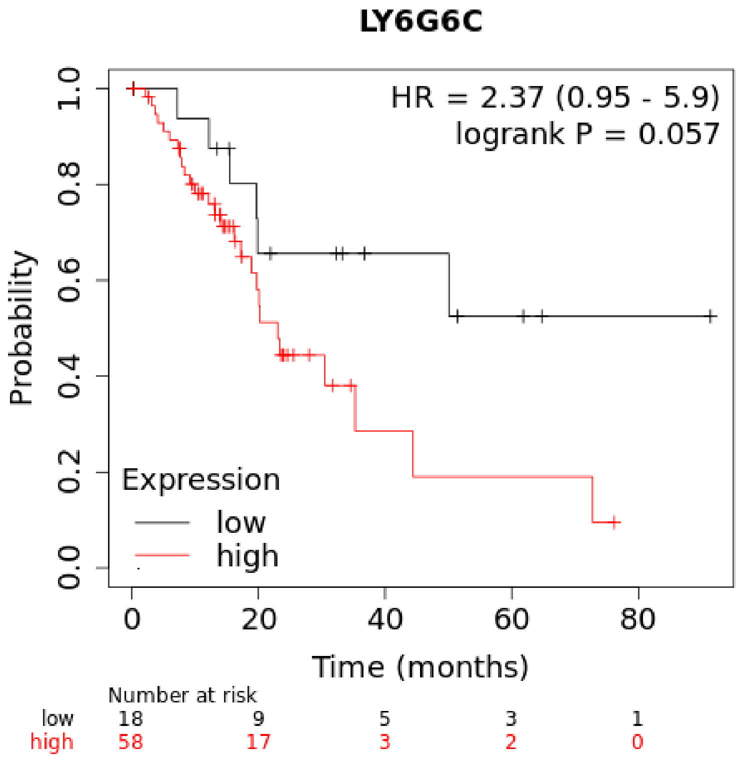

Click here to download the plot in TIFF format

Download plot as a PDF

Download p values vs. cutoff table

Upper quartile survival

Low expression cohort (months)	High expression cohort (months)
19.73	13.1

RNAseq ID: LY6G6D -
Survival: OS
Auto select best cutoff: checked
Follow up threshold: all
Censore at threshold: checked
Compute median over entire database: false
Cutoff value used in analysis: 0
Expression range of the probe: 0 - 2
Invert HR values below 1: not checked

Restrictions

Tumor type: Pancreatic ductal adenocarcinoma

Restrict analysis to subtypes...

Stage: all
Gender: all
Race: all
Grade: all
Mutation burden: all

Restrict analysis based on cellular content...

Basophils: all

B-cells:	all
CD4+ memory T-cells:	all
CD8+ T-cells:	enriched
Eosinophils:	all
Macrophages:	all
Mesenchymal stem cells:	all
Natural killer T-cells:	all
Regulatory T-cells:	all
Type 1 T-helper cells:	all
Type 2 T-helper cells:	all

Results

P value: 0.0701
FDR: 100%

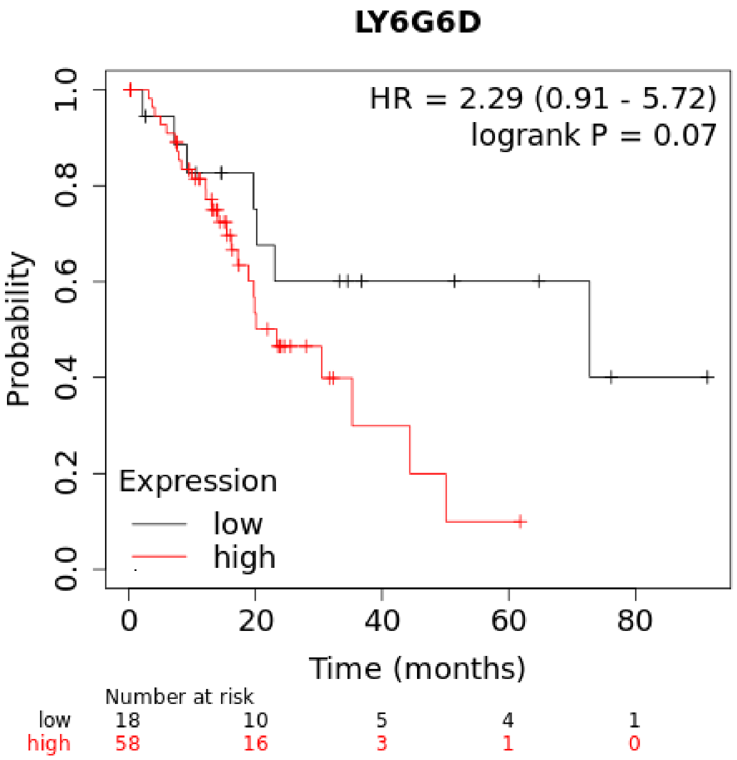

Click here to download the plot in TIFF format

Download plot as a PDF

Download p values vs. cutoff table

Median survival

Low expression cohort (months)	High expression cohort (months)
72.73	23.4

**RNAseq ID:**	LY6G6F -
**Survival:**	OS
**Auto select best cutoff:**	checked
**Follow up threshold:**	all
**Censore at threshold:**	checked
**Compute median over entire database:**	false
**Cutoff value used in analysis:**	0
**Expression range of the probe:**	0 - 5
**Invert HR values below 1:**	not checked

Restrictions

Tumor type: Pancreatic ductal adenocarcinoma

Restrict analysis to subtypes...

Stage:	all
Gender:	all
Race:	all
Grade:	all
Mutation burden:	all

Restrict analysis based on cellular content...

Basophils:	all
B-cells:	all
CD4+ memory T-cells:	all
CD8+ T-cells:	enriched
Eosinophils:	all
Macrophages:	all
Mesenchymal stem cells:	all
Natural killer T-cells:	all
Regulatory T-cells:	all
Type 1 T-helper cells:	all
Type 2 T-helper cells:	all

Results

P value: 0.0377
FDR: over 50%

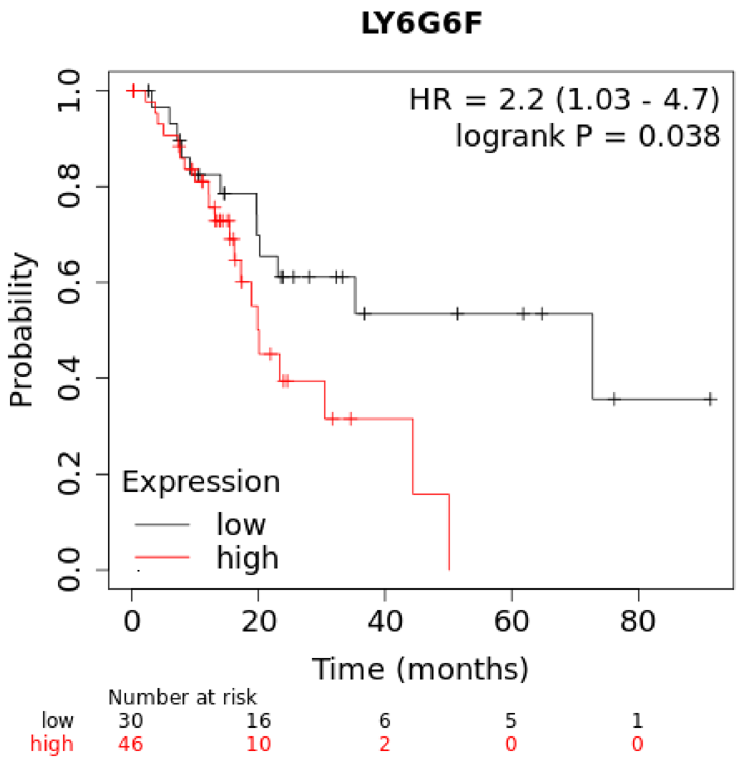

[Click here to download the plot in TIFF format]

[Download plot as a PDF]

[Download p values vs. cutoff table]

Median survival

Low expression cohort (months)	High expression cohort (months)
72.73	20.1

**RNAseq ID:**	LY6G5C -
**Survival:**	OS
**Auto select best cutoff:**	checked
**Follow up threshold:**	all
**Censore at threshold:**	checked
**Compute median over entire database:**	false
**Cutoff value used in analysis:**	67
**Expression range of the probe:**	27 - 253
**Invert HR values below 1:**	not checked

Restrictions

Tumor type: Pancreatic ductal adenocarcinoma

Restrict analysis to subtypes...

Stage:	all
Gender:	all
Race:	all
Grade:	all
Mutation burden:	all

Restrict analysis based on cellular content...

Basophils:	all
B-cells:	all
CD4+ memory T-cells:	all
CD8+ T-cells:	enriched
Eosinophils:	all
Macrophages:	all
Mesenchymal stem cells:	all
Natural killer T-cells:	all
Regulatory T-cells:	all
Type 1 T-helper cells:	all
Type 2 T-helper cells:	all

Results

**P value:**	0.0022
**FDR:**	10%

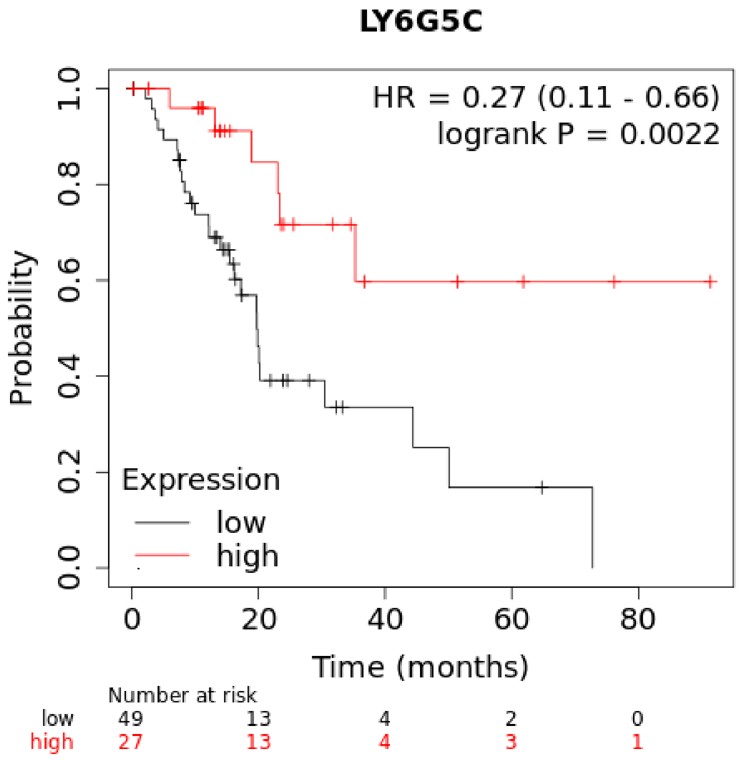

Click here to download the plot in TIFF format

Download plot as a PDF

Download p values vs. cutoff table

Upper quartile survival

Low expression cohort (months)	High expression cohort (months)
9.97	23.4

**RNAseq ID:**	LY6G5B -
**Survival:**	OS
**Auto select best cutoff:**	checked
**Follow up threshold:**	all
**Censore at threshold:**	checked
**Compute median over entire database:**	false
**Cutoff value used in analysis:**	38
**Expression range of the probe:**	17 - 173
**Invert HR values below 1:**	not checked

Restrictions

Tumor type: Pancreatic ductal adenocarcinoma

Restrict analysis to subtypes...

Stage:	all
Gender:	all
Race:	all
Grade:	all
Mutation burden:	all

Restrict analysis based on cellular content...

Basophils:	all

B-cells:	all
CD4+ memory T-cells:	all
CD8+ T-cells:	enriched
Eosinophils:	all
Macrophages:	all
Mesenchymal stem cells:	all
Natural killer T-cells:	all
Regulatory T-cells:	all
Type 1 T-helper cells:	all
Type 2 T-helper cells:	all

Results

P value: 0.0113
FDR: over 50%

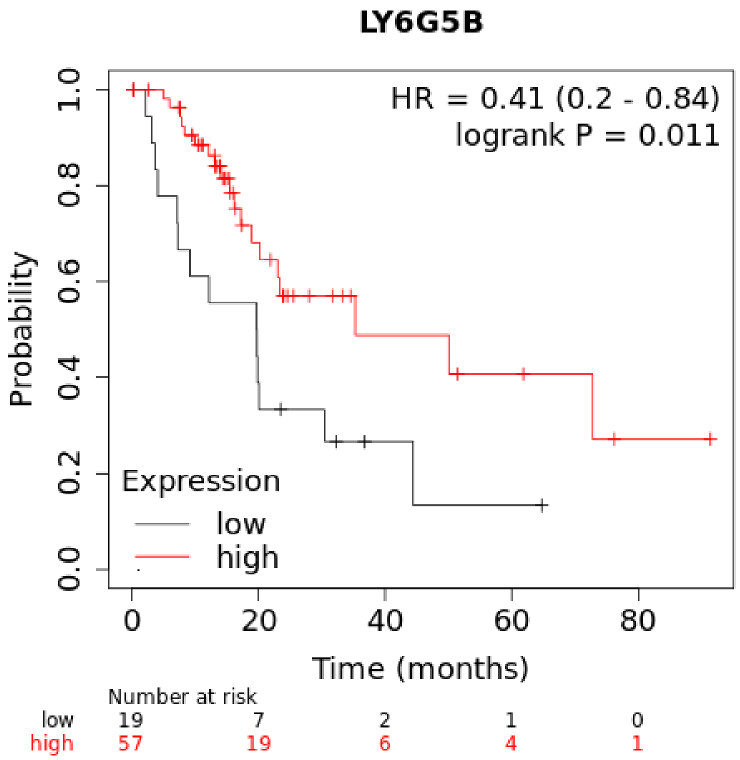

Click here to download the plot in TIFF format

Download plot as a PDF

Download p values vs. cutoff table

Median survival

Low expression cohort (months)	High expression cohort (months)
19.77	35.3

You can save the plots by right-clicking the image and then selecting "Save image as...". To generate a high resolution TIFF image, please adjust the "Settings" in the analysis page.

KMplot.com Copyright © 2009-2020 | Contact | Sitemap

Pan-cancer

KM plotter | Home | Vote | Download | Updates | Contact

The desired RNAseq ID is valid: PSCA (-), LY6K (-), SLURP1 (-), LYPD2 (-), LY6D (-), GML (-), LY6E (-), LY6L (-), LY6H (-), GPIHBP1 (-), LYPD4 (-), CD177 (-), TEX101 (-), LYPD3 (-), PINLYP (-), PLAUR (-), LYPD5 (-), SPACA4 (-), ACRV1 (-), PATE1 (-), PATE2 (-), PATE3 (-), PATE4 (-), CD59 (-), LY6G6C (-), LY6G6D (-), LY6G6F (-), LY6G5C (-), LY6G5B (-),

**RNAseq ID:**	PSCA -
**Survival:**	OS
**Auto select best cutoff:**	checked
**Follow up threshold:**	all
**Censore at threshold:**	checked
**Compute median over entire database:**	false
**Cutoff value used in analysis:**	350
**Expression range of the probe:**	0 - 65661
**Invert HR values below 1:**	not checked

Restrictions

Tumor type: Pancreatic ductal adenocarcinoma

Restrict analysis to subtypes...

Stage:	all
Gender:	all
Race:	all
Grade:	all
Mutation burden:	all

Restrict analysis based on cellular content...

Basophils:	all
B-cells:	all
CD4+ memory T-cells:	all
CD8+ T-cells:	decreased
Eosinophils:	all
Macrophages:	all
Mesenchymal stem cells:	all
Natural killer T-cells:	all
Regulatory T-cells:	all
Type 1 T-helper cells:	all
Type 2 T-helper cells:	all

Results

**P value:**	0.0034
**FDR:**	50%

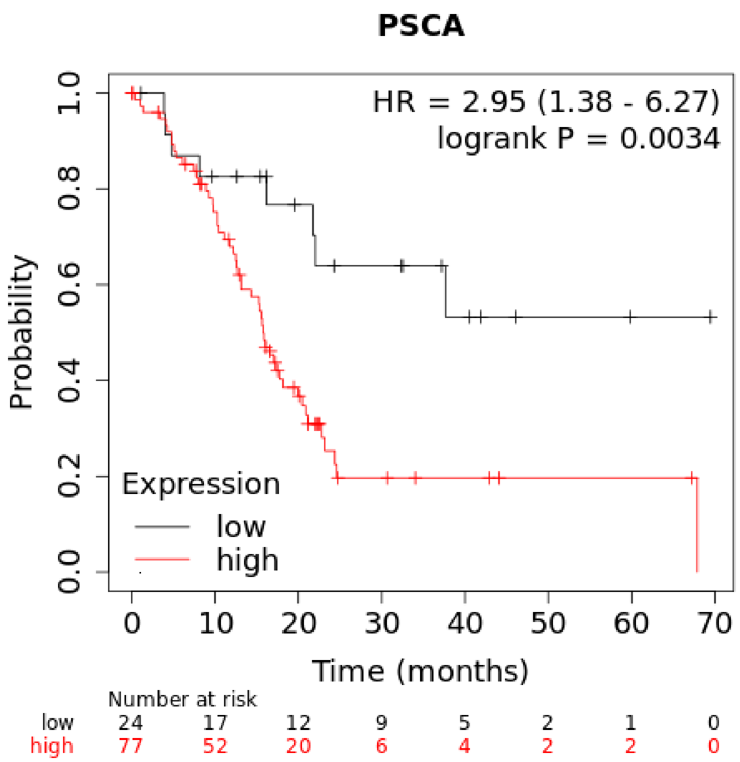

Click here to download the plot in TIFF format

Download plot as a PDF

Download p values vs. cutoff table

Upper quartile survival

Low expression cohort (months)	High expression cohort (months)
21.73	10.27

**RNAseq ID:**	LY6K -
**Survival:**	OS
**Auto select best cutoff:**	checked
**Follow up threshold:**	all
**Censore at threshold:**	checked
**Compute median over entire database:**	false
**Cutoff value used in analysis:**	5
**Expression range of the probe:**	0 - 1825
**Invert HR values below 1:**	not checked

Restrictions

Tumor type: Pancreatic ductal adenocarcinoma

Restrict analysis to subtypes...

Stage:	all
Gender:	all
Race:	all
Grade:	all
Mutation burden:	all

Restrict analysis based on cellular content...

Basophils:	all

B-cells:	all
CD4+ memory T-cells:	all
CD8+ T-cells:	decreased
Eosinophils:	all
Macrophages:	all
Mesenchymal stem cells:	all
Natural killer T-cells:	all
Regulatory T-cells:	all
Type 1 T-helper cells:	all
Type 2 T-helper cells:	all

Results

P value: 0.0743
FDR: 100%

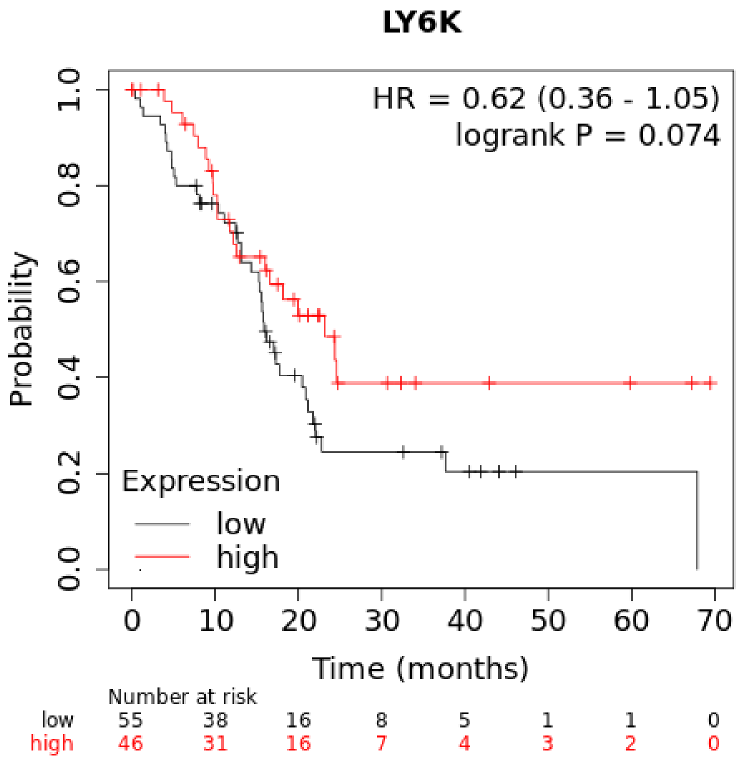

Click here to download the plot in TIFF format

Download plot as a PDF

Download p values vs. cutoff table

Median survival

Low expression cohort (months)	High expression cohort (months)
15.87	23.17

**RNAseq ID:**	SLURP1 -
**Survival:**	OS
**Auto select best cutoff:**	checked
**Follow up threshold:**	all
**Censore at threshold:**	checked
**Compute median over entire database:**	false
**Cutoff value used in analysis:**	1
**Expression range of the probe:**	0 - 279
**Invert HR values below 1:**	not checked

Restrictions

Tumor type: Pancreatic ductal adenocarcinoma

Restrict analysis to subtypes...

Stage:	all
Gender:	all
Race:	all
Grade:	all
Mutation burden:	all

Restrict analysis based on cellular content...

Basophils:	all
B-cells:	all
CD4+ memory T-cells:	all
CD8+ T-cells:	decreased
Eosinophils:	all
Macrophages:	all
Mesenchymal stem cells:	all
Natural killer T-cells:	all
Regulatory T-cells:	all
Type 1 T-helper cells:	all
Type 2 T-helper cells:	all

Results

P value: 0.0017
FDR: 50%

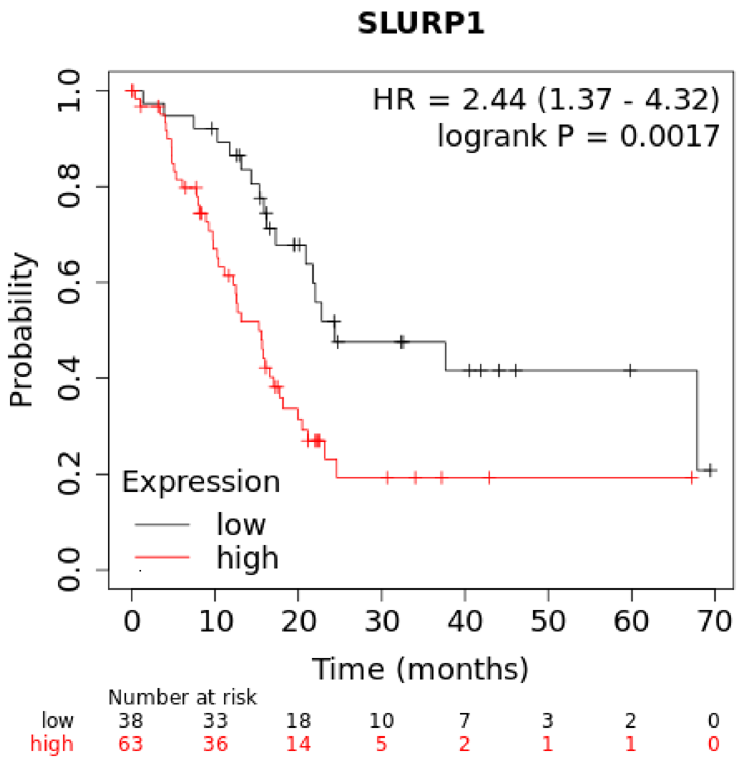

Click here to download the plot in TIFF format

Download plot as a PDF

Download p values vs. cutoff table

Median survival

Low expression cohort (months)	High expression cohort (months)
24.4	15.27

**RNAseq ID:**	LYPD2 -
**Survival:**	OS
**Auto select best cutoff:**	checked
**Follow up threshold:**	all
**Censore at threshold:**	checked
**Compute median over entire database:**	false
**Cutoff value used in analysis:**	4
**Expression range of the probe:**	0 - 4748
**Invert HR values below 1:**	not checked

Restrictions

Tumor type: Pancreatic ductal adenocarcinoma

Restrict analysis to subtypes...

Stage:	all
Gender:	all
Race:	all
Grade:	all
Mutation burden:	all

Restrict analysis based on cellular content...

Basophils:	all
B-cells:	all
CD4+ memory T-cells:	all
CD8+ T-cells:	decreased
Eosinophils:	all
Macrophages:	all
Mesenchymal stem cells:	all
Natural killer T-cells:	all
Regulatory T-cells:	all
Type 1 T-helper cells:	all
Type 2 T-helper cells:	all

Results

**P value:**	0.097
**FDR:**	100%

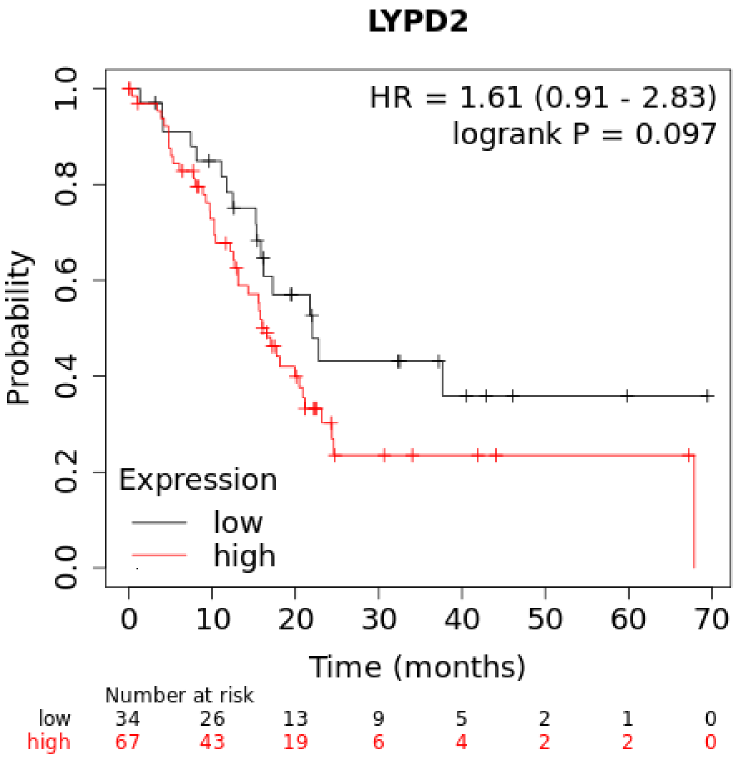

Click here to download the plot in TIFF format

Download plot as a PDF

Download p values vs. cutoff table

Median survival

Low expression cohort (months)	High expression cohort (months)
22.03	16.6

RNAseq ID: LY6D -
Survival: OS
Auto select best cutoff: checked
Follow up threshold: all
Censore at threshold: checked
Compute median over entire database: false
Cutoff value used in analysis: 598
Expression range of the probe: 0 - 18030
Invert HR values below 1: not checked

Restrictions

Tumor type: Pancreatic ductal adenocarcinoma

Restrict analysis to subtypes...

Stage: all
Gender: all
Race: all
Grade: all
Mutation burden: all

Restrict analysis based on cellular content...

Basophils: all

B-cells: all
CD4+ memory T-cells: all
CD8+ T-cells: decreased
Eosinophils: all
Macrophages: all
Mesenchymal stem cells: all
Natural killer T-cells: all
Regulatory T-cells: all
Type 1 T-helper cells: all
Type 2 T-helper cells: all

Results

P value: 0.0016
FDR: 20%

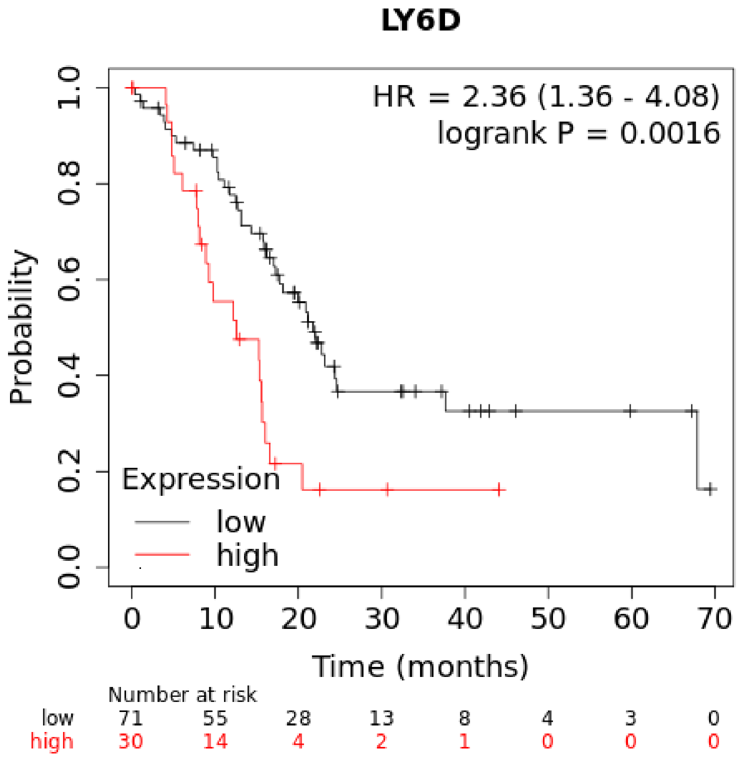

Click here to download the plot in TIFF format

Download plot as a PDF

Download p values vs. cutoff table

Median survival

Low expression cohort (months)	High expression cohort (months)
21.73	12.6

RNAseq ID: GML -
Survival: OS
Auto select best cutoff: checked
Follow up threshold: all
Censore at threshold: checked
Compute median over entire database: false
Cutoff value used in analysis: 0
Expression range of the probe: 0 - 2
Invert HR values below 1: not checked

Restrictions

Tumor type: Pancreatic ductal adenocarcinoma

Restrict analysis to subtypes...

Stage:	all
Gender:	all
Race:	all
Grade:	all
Mutation burden:	all

Restrict analysis based on cellular content...

Basophils:	all
B-cells:	all
CD4+ memory T-cells:	all
CD8+ T-cells:	decreased
Eosinophils:	all
Macrophages:	all
Mesenchymal stem cells:	all
Natural killer T-cells:	all
Regulatory T-cells:	all
Type 1 T-helper cells:	all
Type 2 T-helper cells:	all

Results

P value: 0.1333
FDR: 100%

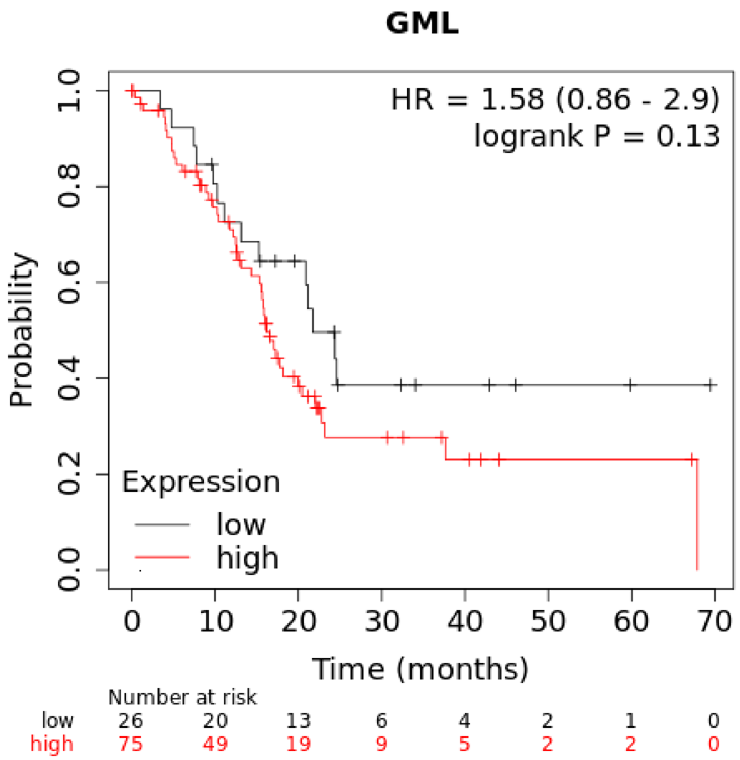

Click here to download the plot in TIFF format

Download plot as a PDF

Download p values vs. cutoff table

Median survival

Low expression cohort (months)	High expression cohort (months)
21.73	16.17

**RNAseq ID:**	LY6E	-
**Survival:**	OS	
**Auto select best cutoff:**	checked	
**Follow up threshold:**	all	
**Censore at threshold:**	checked	
**Compute median over entire database:**	false	
**Cutoff value used in analysis:**	10634	
**Expression range of the probe:**	504 - 56404	
**Invert HR values below 1:**	not checked	

Restrictions

Tumor type: Pancreatic ductal adenocarcinoma

Restrict analysis to subtypes...

Stage:	all
Gender:	all
Race:	all
Grade:	all
Mutation burden:	all

Restrict analysis based on cellular content...

Basophils:	all
B-cells:	all
CD4+ memory T-cells:	all
CD8+ T-cells:	decreased
Eosinophils:	all
Macrophages:	all
Mesenchymal stem cells:	all
Natural killer T-cells:	all
Regulatory T-cells:	all
Type 1 T-helper cells:	all
Type 2 T-helper cells:	all

Results

**P value:**	0.0815
**FDR:**	100%

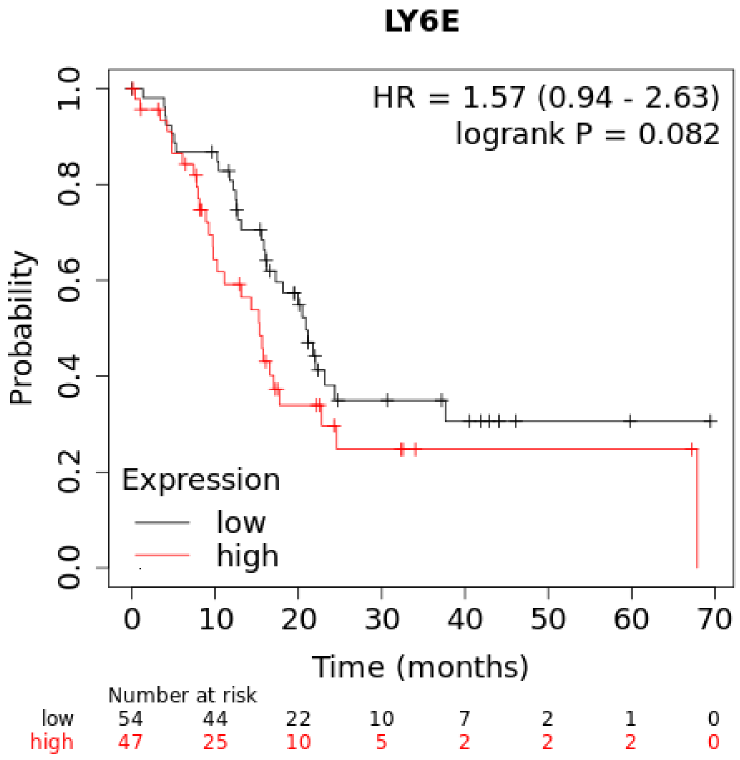

Click here to download the plot in TIFF format

Download plot as a PDF

Download p values vs. cutoff table

Median survival

Low expression cohort (months)	High expression cohort (months)
20.9	15.33

**RNAseq ID:**	LY6L -
**Survival:**	OS
**Auto select best cutoff:**	checked
**Follow up threshold:**	all
**Censore at threshold:**	checked
**Compute median over entire database:**	false
**Cutoff value used in analysis:**	0
**Expression range of the probe:**	0 - 6
**Invert HR values below 1:**	not checked

Restrictions

Tumor type: Pancreatic ductal adenocarcinoma

Restrict analysis to subtypes...

Stage:	all
Gender:	all
Race:	all
Grade:	all
Mutation burden:	all

Restrict analysis based on cellular content...

Basophils:	all

B-cells:	all
CD4+ memory T-cells:	all
CD8+ T-cells:	decreased
Eosinophils:	all
Macrophages:	all
Mesenchymal stem cells:	all
Natural killer T-cells:	all
Regulatory T-cells:	all
Type 1 T-helper cells:	all
Type 2 T-helper cells:	all

Results

P value: 0.1345
FDR: 100%

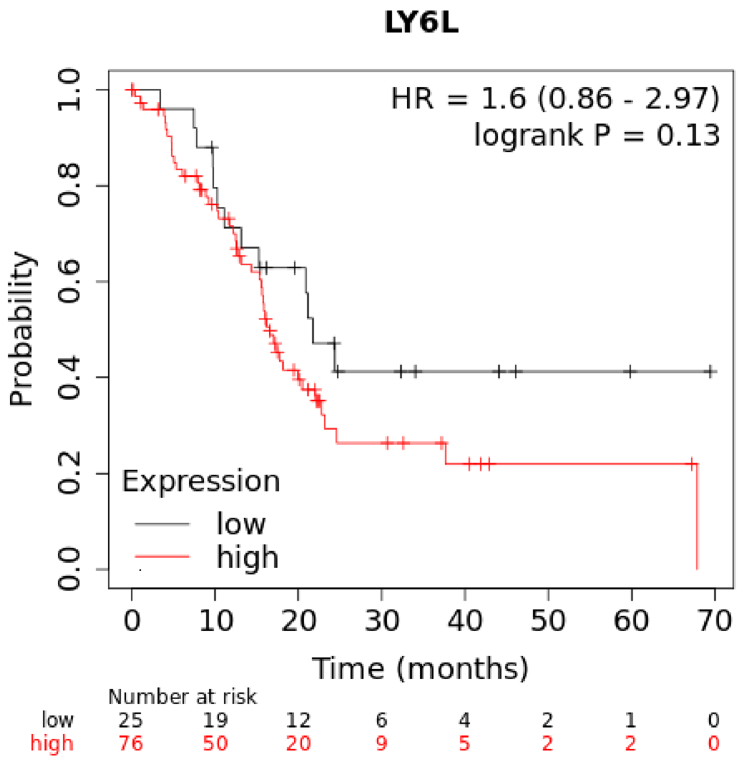

Click here to download the plot in TIFF format

Download plot as a PDF

Download p values vs. cutoff table

Median survival

Low expression cohort (months)	High expression cohort (months)
21.73	16.6

**RNAseq ID:**	LY6H -
**Survival:**	OS
**Auto select best cutoff:**	checked
**Follow up threshold:**	all
**Censore at threshold:**	checked
**Compute median over entire database:**	false
**Cutoff value used in analysis:**	43
**Expression range of the probe:**	1 - 866
**Invert HR values below 1:**	not checked

Restrictions

Tumor type: Pancreatic ductal adenocarcinoma

Restrict analysis to subtypes...

Stage:	all
Gender:	all
Race:	all
Grade:	all
Mutation burden:	all

Restrict analysis based on cellular content...

Basophils:	all
B-cells:	all
CD4+ memory T-cells:	all
CD8+ T-cells:	decreased
Eosinophils:	all
Macrophages:	all
Mesenchymal stem cells:	all
Natural killer T-cells:	all
Regulatory T-cells:	all
Type 1 T-helper cells:	all
Type 2 T-helper cells:	all

Results

P value: 0.0782
FDR: 100%

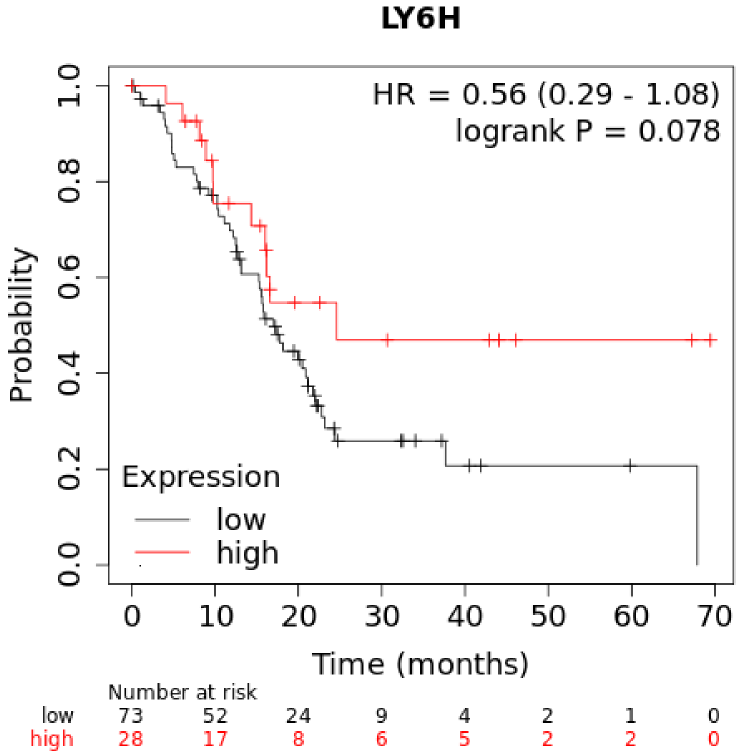

Click here to download the plot in TIFF format

Download plot as a PDF

Download p values vs. cutoff table

Median survival

Low expression cohort (months)	High expression cohort (months)
17.03	24.6

**RNAseq ID:**	GPIHBP1 -
**Survival:**	OS
**Auto select best cutoff:**	checked
**Follow up threshold:**	all
**Censore at threshold:**	checked
**Compute median over entire database:**	false
**Cutoff value used in analysis:**	23
**Expression range of the probe:**	5 - 344
**Invert HR values below 1:**	not checked

Restrictions

Tumor type: Pancreatic ductal adenocarcinoma

Restrict analysis to subtypes...

Stage:	all
Gender:	all
Race:	all
Grade:	all
Mutation burden:	all

Restrict analysis based on cellular content...

Basophils:	all
B-cells:	all
CD4+ memory T-cells:	all
CD8+ T-cells:	decreased
Eosinophils:	all
Macrophages:	all
Mesenchymal stem cells:	all
Natural killer T-cells:	all
Regulatory T-cells:	all
Type 1 T-helper cells:	all
Type 2 T-helper cells:	all

Results

**P value:**	0.3391
**FDR:**	100%

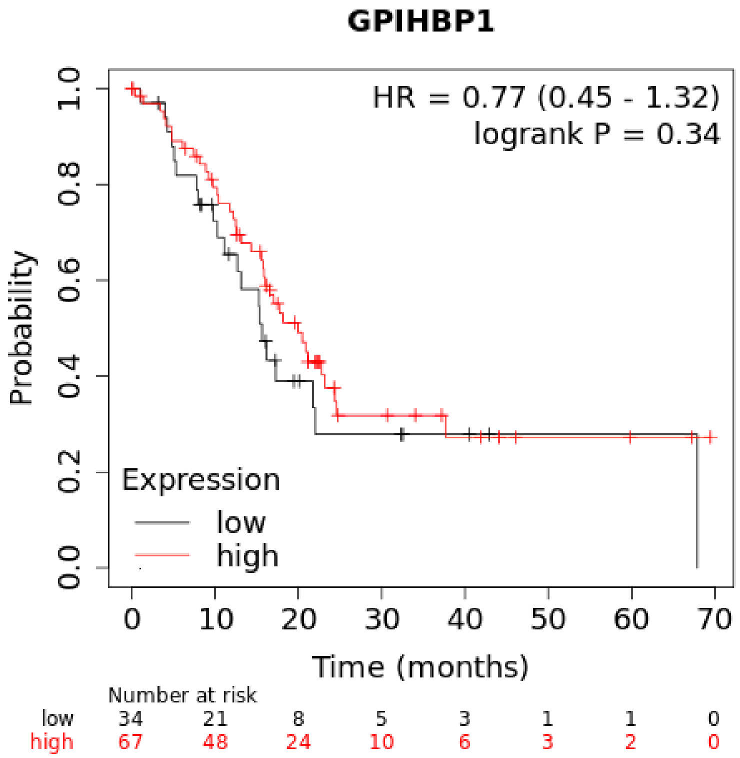

Click here to download the plot in TIFF format

Download plot as a PDF

Download p values vs. cutoff table

Median survival

Low expression cohort (months)	High expression cohort (months)
15.67	19.93

**RNAseq ID:**	LYPD4 -
**Survival:**	OS
**Auto select best cutoff:**	checked
**Follow up threshold:**	all
**Censore at threshold:**	checked
**Compute median over entire database:**	false
**Cutoff value used in analysis:**	0
**Expression range of the probe:**	0 - 18
**Invert HR values below 1:**	not checked

Restrictions

Tumor type: Pancreatic ductal adenocarcinoma

Restrict analysis to subtypes...

Stage:	all
Gender:	all
Race:	all
Grade:	all
Mutation burden:	all

Restrict analysis based on cellular content...

Basophils:	all

B-cells:	all
CD4+ memory T-cells:	all
CD8+ T-cells:	decreased
Eosinophils:	all
Macrophages:	all
Mesenchymal stem cells:	all
Natural killer T-cells:	all
Regulatory T-cells:	all
Type 1 T-helper cells:	all
Type 2 T-helper cells:	all

Results

P value: 0.044
FDR: over 50%

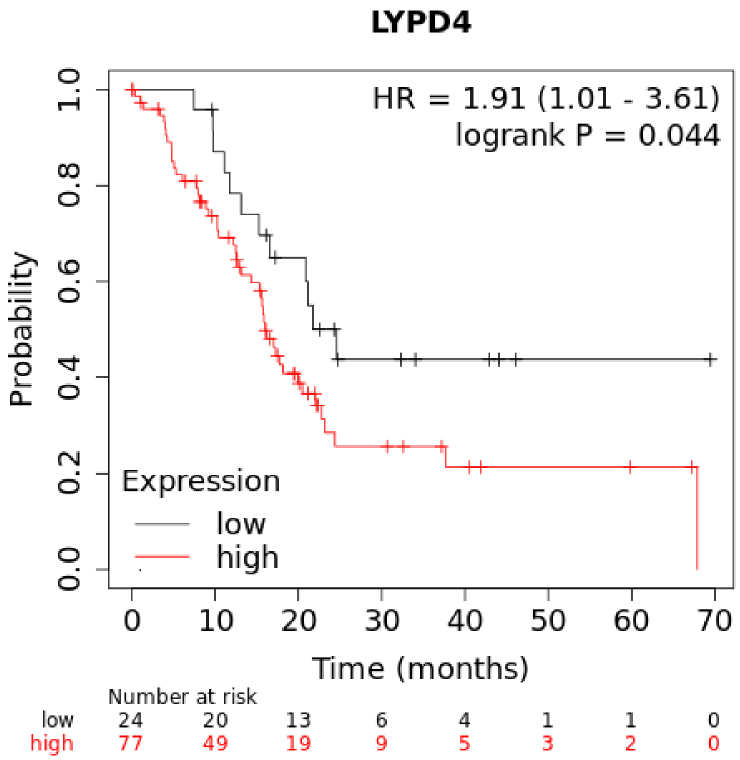

Click here to download the plot in TIFF format

Download plot as a PDF

Download p values vs. cutoff table

Median survival

Low expression cohort (months)	High expression cohort (months)
24.6	16.03

**RNAseq ID:**	CD177 -
**Survival:**	OS
**Auto select best cutoff:**	checked
**Follow up threshold:**	all
**Censore at threshold:**	checked
**Compute median over entire database:**	false
**Cutoff value used in analysis:**	31
**Expression range of the probe:**	0 - 3766
**Invert HR values below 1:**	not checked

Restrictions

Tumor type: Pancreatic ductal adenocarcinoma

Restrict analysis to subtypes...

Stage: all
Gender: all
Race: all
Grade: all
Mutation burden: all

Restrict analysis based on cellular content...

Basophils: all
B-cells: all
CD4+ memory T-cells: all
CD8+ T-cells: decreased
Eosinophils: all
Macrophages: all
Mesenchymal stem cells: all
Natural killer T-cells: all
Regulatory T-cells: all
Type 1 T-helper cells: all
Type 2 T-helper cells: all

Results

P value: 0.2691
FDR: 100%

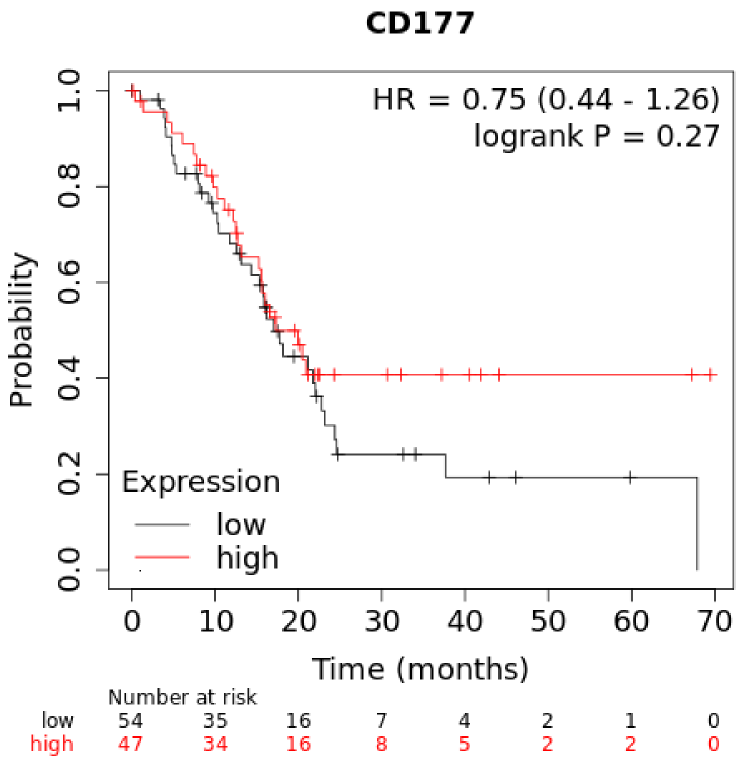

Click here to download the plot in TIFF format

Download plot as a PDF

Download p values vs. cutoff table

Median survival

Low expression cohort (months)	High expression cohort (months)
17.03	17.27

**RNAseq ID:**	TEX101 -
**Survival:**	OS
**Auto select best cutoff:**	checked
**Follow up threshold:**	all
**Censore at threshold:**	checked
**Compute median over entire database:**	false
**Cutoff value used in analysis:**	2
**Expression range of the probe:**	0 - 149
**Invert HR values below 1:**	not checked

Restrictions

Tumor type: Pancreatic ductal adenocarcinoma

Restrict analysis to subtypes...

Stage:	all
Gender:	all
Race:	all
Grade:	all
Mutation burden:	all

Restrict analysis based on cellular content...

Basophils:	all
B-cells:	all
CD4+ memory T-cells:	all
CD8+ T-cells:	decreased
Eosinophils:	all
Macrophages:	all
Mesenchymal stem cells:	all
Natural killer T-cells:	all
Regulatory T-cells:	all
Type 1 T-helper cells:	all
Type 2 T-helper cells:	all

Results

**P value:**	0.1413
**FDR:**	100%

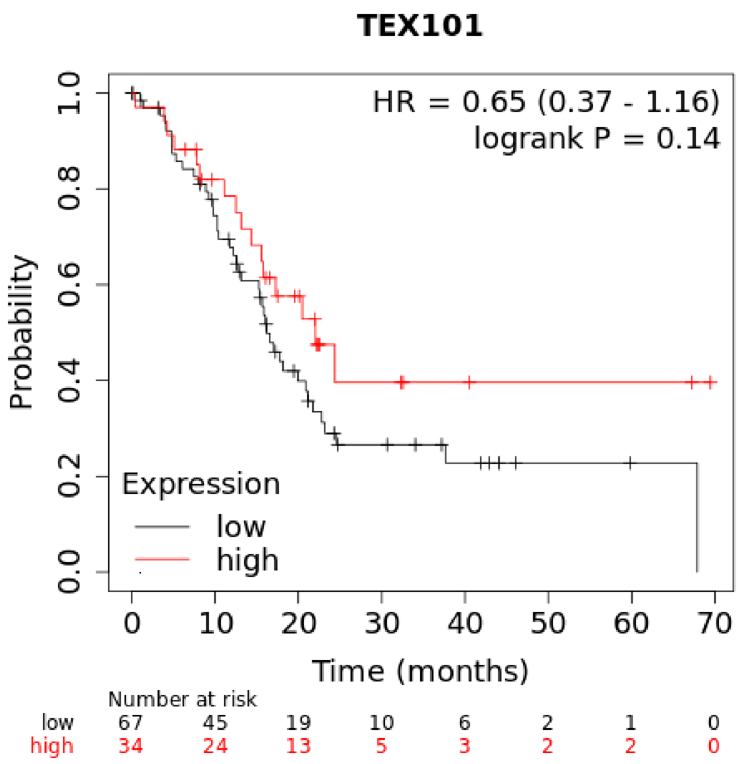

Click here to download the plot in TIFF format

Download plot as a PDF

Download p values vs. cutoff table

Median survival

Low expression cohort (months)	High expression cohort (months)
16.17	22.03

RNAseq ID: LYPD3 -
Survival: OS
Auto select best cutoff: checked
Follow up threshold: all
Censore at threshold: checked
Compute median over entire database: false
Cutoff value used in analysis: 279
Expression range of the probe: 9 - 7684
Invert HR values below 1: not checked

Restrictions

Tumor type: Pancreatic ductal adenocarcinoma

Restrict analysis to subtypes...

Stage: all
Gender: all
Race: all
Grade: all
Mutation burden: all

Restrict analysis based on cellular content...

Basophils: all

B-cells:	all
CD4+ memory T-cells:	all
CD8+ T-cells:	decreased
Eosinophils:	all
Macrophages:	all
Mesenchymal stem cells:	all
Natural killer T-cells:	all
Regulatory T-cells:	all
Type 1 T-helper cells:	all
Type 2 T-helper cells:	all

Results

P value: 0.009
FDR: 50%

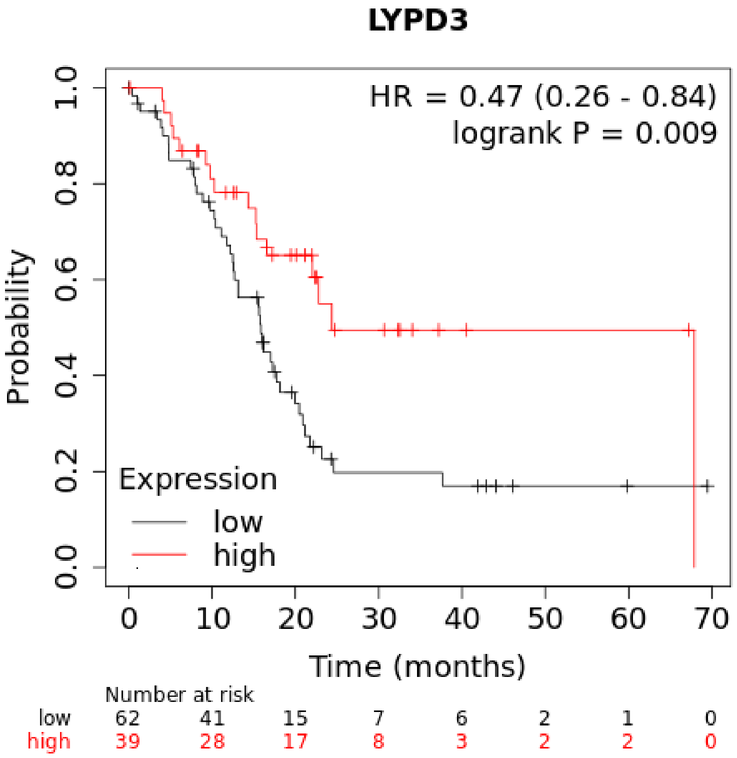

Click here to download the plot in TIFF format

Download plot as a PDF

Download p values vs. cutoff table

Median survival

Low expression cohort (months)	High expression cohort (months)
15.87	24.4

**RNAseq ID:**	PINLYP -
**Survival:**	OS
**Auto select best cutoff:**	checked
**Follow up threshold:**	all
**Censore at threshold:**	checked
**Compute median over entire database:**	false
**Cutoff value used in analysis:**	70
**Expression range of the probe:**	5 - 387
**Invert HR values below 1:**	not checked

Restrictions

Tumor type: Pancreatic ductal adenocarcinoma

Restrict analysis to subtypes...

Stage:	all
Gender:	all
Race:	all
Grade:	all
Mutation burden:	all

Restrict analysis based on cellular content...

Basophils:	all
B-cells:	all
CD4+ memory T-cells:	all
CD8+ T-cells:	decreased
Eosinophils:	all
Macrophages:	all
Mesenchymal stem cells:	all
Natural killer T-cells:	all
Regulatory T-cells:	all
Type 1 T-helper cells:	all
Type 2 T-helper cells:	all

Results

P value: 0.3196
FDR: 100%

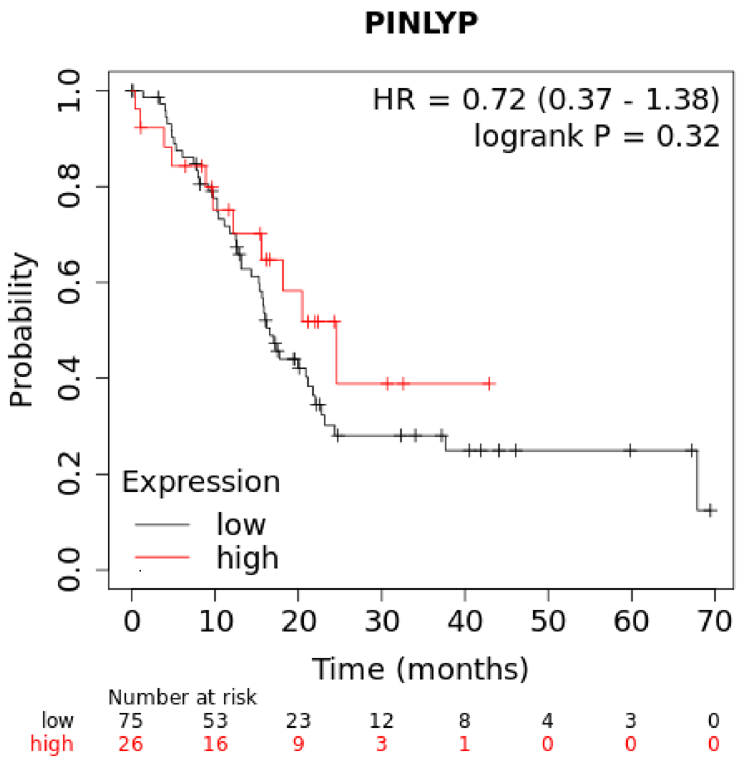

Click here to download the plot in TIFF format

Download plot as a PDF

Download p values vs. cutoff table

Median survival

Low expression cohort (months)	High expression cohort (months)
16.6	24.6

**RNAseq ID:**	PLAUR -
**Survival:**	OS
**Auto select best cutoff:**	checked
**Follow up threshold:**	all
**Censore at threshold:**	checked
**Compute median over entire database:**	false
**Cutoff value used in analysis:**	3016
**Expression range of the probe:**	55 - 18314
**Invert HR values below 1:**	not checked

Restrictions

Tumor type: Pancreatic ductal adenocarcinoma

Restrict analysis to subtypes...

Stage:	all
Gender:	all
Race:	all
Grade:	all
Mutation burden:	all

Restrict analysis based on cellular content...

Basophils:	all
B-cells:	all
CD4+ memory T-cells:	all
CD8+ T-cells:	decreased
Eosinophils:	all
Macrophages:	all
Mesenchymal stem cells:	all
Natural killer T-cells:	all
Regulatory T-cells:	all
Type 1 T-helper cells:	all
Type 2 T-helper cells:	all

Results

**P value:**	0.2066
**FDR:**	100%

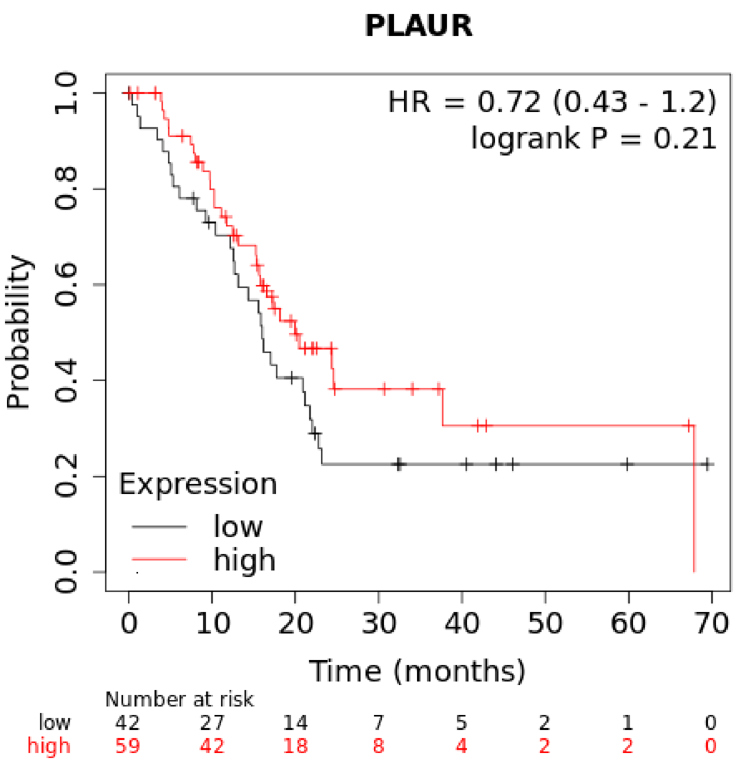

Click here to download the plot in TIFF format

Download plot as a PDF

Download p values vs. cutoff table

Median survival

Low expression cohort (months)	High expression cohort (months)
16.03	19.93

**RNAseq ID:**	LYPD5 -
**Survival:**	OS
**Auto select best cutoff:**	checked
**Follow up threshold:**	all
**Censore at threshold:**	checked
**Compute median over entire database:**	false
**Cutoff value used in analysis:**	76
**Expression range of the probe:**	5 - 419
**Invert HR values below 1:**	not checked

Restrictions

Tumor type: Pancreatic ductal adenocarcinoma

Restrict analysis to subtypes...

Stage:	all
Gender:	all
Race:	all
Grade:	all
Mutation burden:	all

Restrict analysis based on cellular content...

Basophils:	all

B-cells:	all
CD4+ memory T-cells:	all
CD8+ T-cells:	decreased
Eosinophils:	all
Macrophages:	all
Mesenchymal stem cells:	all
Natural killer T-cells:	all
Regulatory T-cells:	all
Type 1 T-helper cells:	all
Type 2 T-helper cells:	all

Results

P value: 0.0355
FDR: over 50%

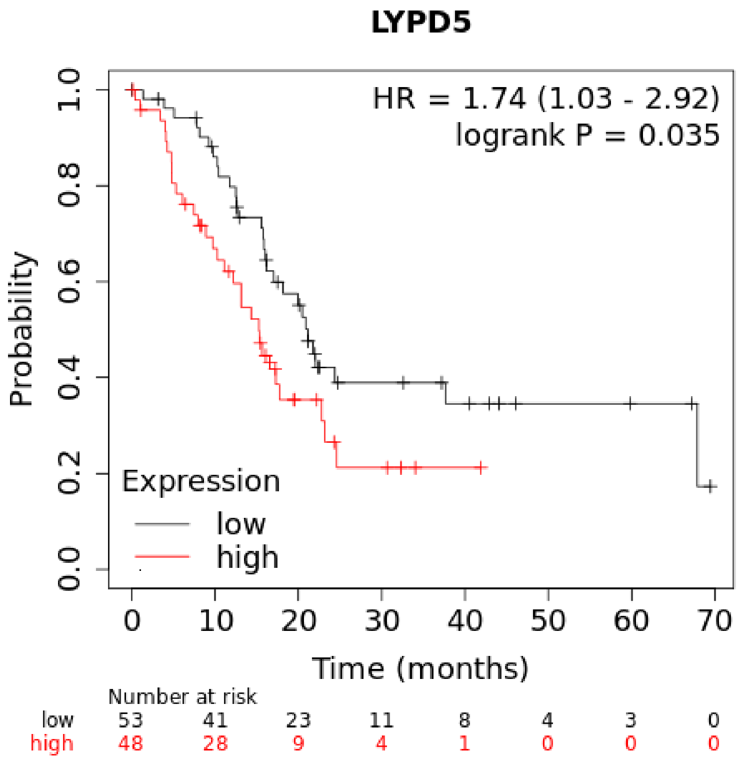

Click here to download the plot in TIFF format

Download plot as a PDF

Download p values vs. cutoff table

Median survival

Low expression cohort (months)	High expression cohort (months)
21.13	15.27

**RNAseq ID:**	SPACA4 -
**Survival:**	OS
**Auto select best cutoff:**	checked
**Follow up threshold:**	all
**Censore at threshold:**	checked
**Compute median over entire database:**	false
**Cutoff value used in analysis:**	12
**Expression range of the probe:**	0 - 206
**Invert HR values below 1:**	not checked

Restrictions

Tumor type: Pancreatic ductal adenocarcinoma

Restrict analysis to subtypes...

Stage: all
Gender: all
Race: all
Grade: all
Mutation burden: all

Restrict analysis based on cellular content...

Basophils: all
B-cells: all
CD4+ memory T-cells: all
CD8+ T-cells: decreased
Eosinophils: all
Macrophages: all
Mesenchymal stem cells: all
Natural killer T-cells: all
Regulatory T-cells: all
Type 1 T-helper cells: all
Type 2 T-helper cells: all

Results

P value: 0.3066
FDR: 100%

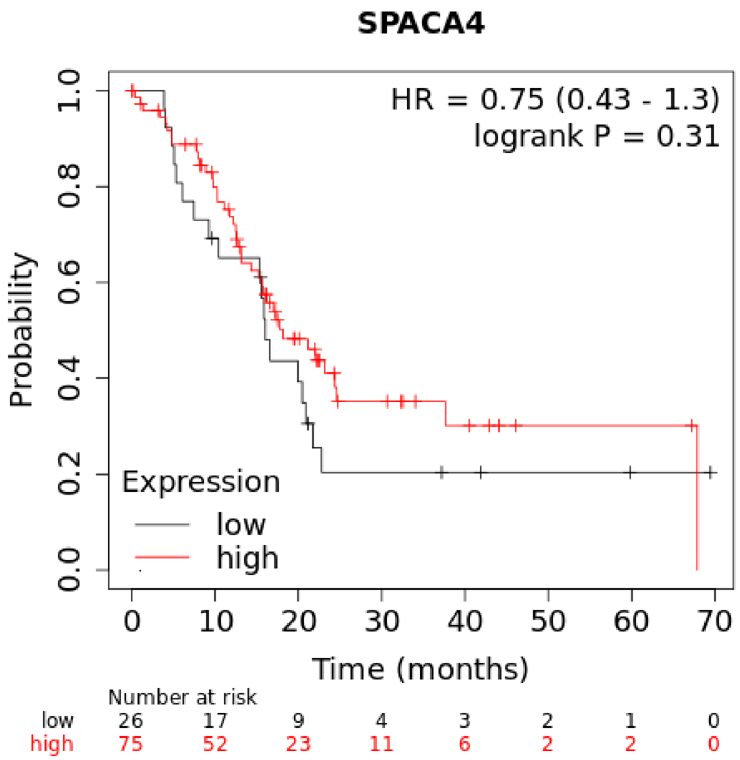

Click here to download the plot in TIFF format

Download plot as a PDF

Download p values vs. cutoff table

Median survival

Low expression cohort (months)	High expression cohort (months)
16.03	18.17

**RNAseq ID:**	ACRV1 -
**Survival:**	OS
**Auto select best cutoff:**	checked
**Follow up threshold:**	all
**Censore at threshold:**	checked
**Compute median over entire database:**	false
**Cutoff value used in analysis:**	4
**Expression range of the probe:**	0 - 71
**Invert HR values below 1:**	not checked

Restrictions

Tumor type: Pancreatic ductal adenocarcinoma

Restrict analysis to subtypes...

Stage:	all
Gender:	all
Race:	all
Grade:	all
Mutation burden:	all

Restrict analysis based on cellular content...

Basophils:	all
B-cells:	all
CD4+ memory T-cells:	all
CD8+ T-cells:	decreased
Eosinophils:	all
Macrophages:	all
Mesenchymal stem cells:	all
Natural killer T-cells:	all
Regulatory T-cells:	all
Type 1 T-helper cells:	all
Type 2 T-helper cells:	all

Results

**P value:**	0.2578
**FDR:**	100%

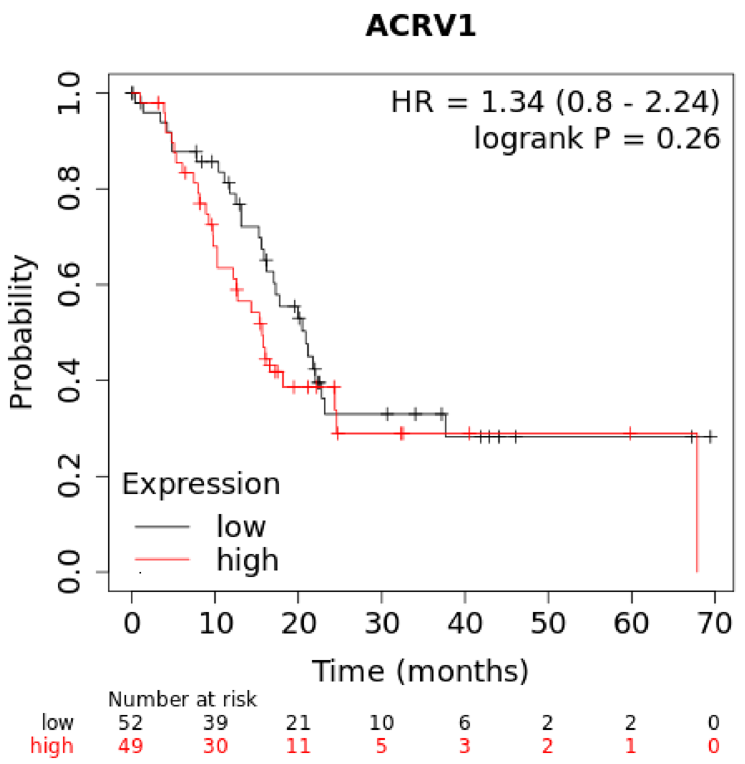

Click here to download the plot in TIFF format

Download plot as a PDF

Download p values vs. cutoff table

Median survival

Low expression cohort (months)	High expression cohort (months)
20.9	15.67

RNAseq ID: PATE1 -
Survival: OS
Auto select best cutoff: checked
Follow up threshold: all
Censore at threshold: checked
Compute median over entire database: false
Cutoff value used in analysis: 0
Expression range of the probe: 0 - 1
Invert HR values below 1: not checked

Restrictions

Tumor type: Pancreatic ductal adenocarcinoma

Restrict analysis to subtypes...

Stage: all
Gender: all
Race: all
Grade: all
Mutation burden: all

Restrict analysis based on cellular content...

Basophils: all

B-cells: all
CD4+ memory T-cells: all
CD8+ T-cells: decreased
Eosinophils: all
Macrophages: all
Mesenchymal stem cells: all
Natural killer T-cells: all
Regulatory T-cells: all
Type 1 T-helper cells: all
Type 2 T-helper cells: all

Results

P value: 0.0216
FDR: over 50%

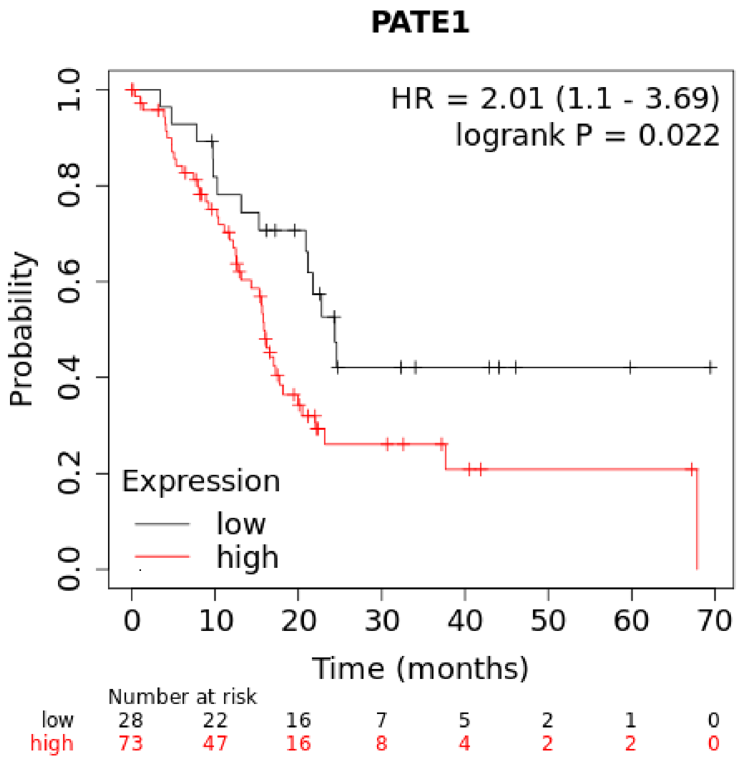

Click here to download the plot in TIFF format

Download plot as a PDF

Download p values vs. cutoff table

Median survival

Low expression cohort (months)	High expression cohort (months)
24.4	15.87

RNAseq ID: PATE2 -
Survival: OS
Auto select best cutoff: checked
Follow up threshold: all
Censore at threshold: checked
Compute median over entire database: false
Cutoff value used in analysis: 1
Expression range of the probe: 0 - 7
Invert HR values below 1: not checked

Restrictions

Tumor type: Pancreatic ductal adenocarcinoma

Restrict analysis to subtypes...

Stage: all
Gender: all
Race: all
Grade: all
Mutation burden: all

Restrict analysis based on cellular content...

Basophils: all
B-cells: all
CD4+ memory T-cells: all
CD8+ T-cells: decreased
Eosinophils: all
Macrophages: all
Mesenchymal stem cells: all
Natural killer T-cells: all
Regulatory T-cells: all
Type 1 T-helper cells: all
Type 2 T-helper cells: all

Results

P value: 0.0108
FDR: over 50%

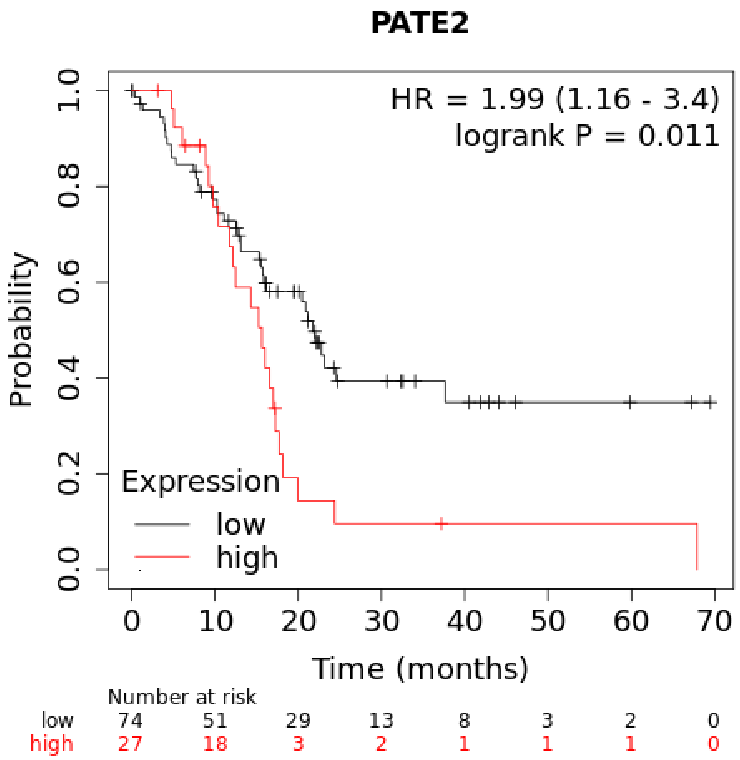

Click here to download the plot in TIFF format

Download plot as a PDF

Download p values vs. cutoff table

Median survival

Low expression cohort (months)	High expression cohort (months)
21.73	15.67

**RNAseq ID:**	PATE3 -
**Survival:**	OS
**Auto select best cutoff:**	checked
**Follow up threshold:**	all
**Censore at threshold:**	checked
**Compute median over entire database:**	false
**Cutoff value used in analysis:**	0
**Expression range of the probe:**	0 - 1
**Invert HR values below 1:**	not checked

Restrictions

Tumor type: Pancreatic ductal adenocarcinoma

Restrict analysis to subtypes...

Stage:	all
Gender:	all
Race:	all
Grade:	all
Mutation burden:	all

Restrict analysis based on cellular content...

Basophils:	all
B-cells:	all
CD4+ memory T-cells:	all
CD8+ T-cells:	decreased
Eosinophils:	all
Macrophages:	all
Mesenchymal stem cells:	all
Natural killer T-cells:	all
Regulatory T-cells:	all
Type 1 T-helper cells:	all
Type 2 T-helper cells:	all

Results

**P value:**	0.1009
**FDR:**	100%

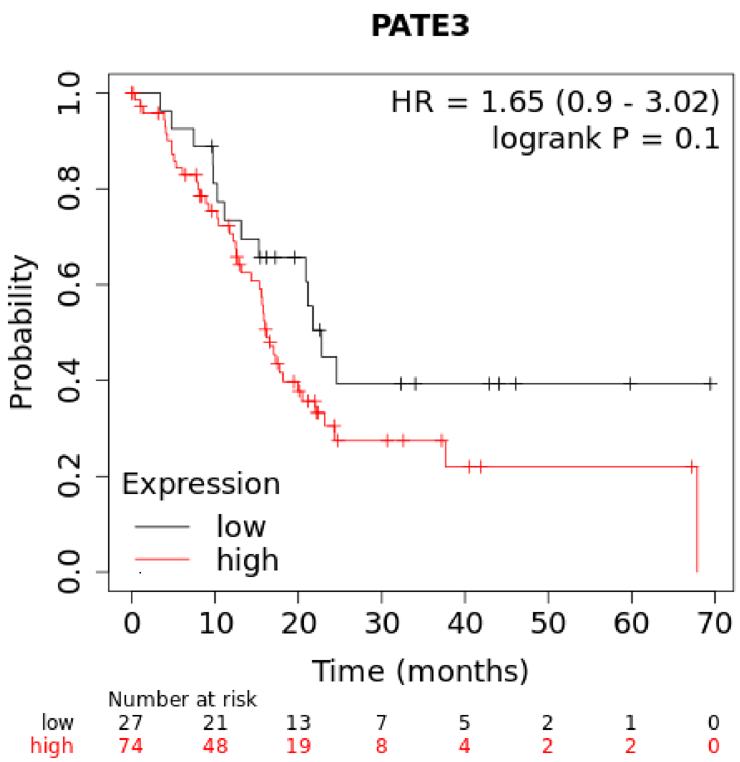

Click here to download the plot in TIFF format

Download plot as a PDF

Download p values vs. cutoff table

Median survival

Low expression cohort (months)	High expression cohort (months)
22.8	16.17

RNAseq ID: PATE4 -
Survival: OS
Auto select best cutoff: checked
Follow up threshold: all
Censore at threshold: checked
Compute median over entire database: false
Cutoff value used in analysis: 0
Expression range of the probe: 0 - 3
Invert HR values below 1: not checked

Restrictions

Tumor type: Pancreatic ductal adenocarcinoma

Restrict analysis to subtypes...

Stage: all
Gender: all
Race: all
Grade: all
Mutation burden: all

Restrict analysis based on cellular content...

Basophils: all

B-cells:	all
CD4+ memory T-cells:	all
CD8+ T-cells:	decreased
Eosinophils:	all
Macrophages:	all
Mesenchymal stem cells:	all
Natural killer T-cells:	all
Regulatory T-cells:	all
Type 1 T-helper cells:	all
Type 2 T-helper cells:	all

Results

P value: 0.0677
FDR: 100%

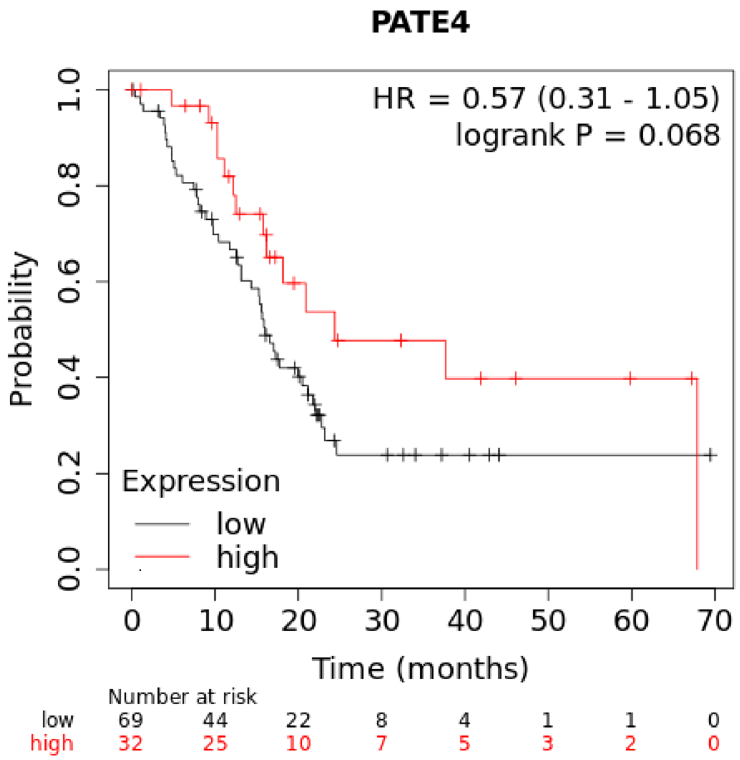

Click here to download the plot in TIFF format

Download plot as a PDF

Download p values vs. cutoff table

Median survival

Low expression cohort (months)	High expression cohort (months)
16.03	24.4

**RNAseq ID:**	CD59 -
**Survival:**	OS
**Auto select best cutoff:**	checked
**Follow up threshold:**	all
**Censore at threshold:**	checked
**Compute median over entire database:**	false
**Cutoff value used in analysis:**	12493
**Expression range of the probe:**	2610 - 39336
**Invert HR values below 1:**	not checked

Restrictions

Tumor type: Pancreatic ductal adenocarcinoma

Restrict analysis to subtypes...

Stage:	all
Gender:	all
Race:	all
Grade:	all
Mutation burden:	all

Restrict analysis based on cellular content...

Basophils:	all
B-cells:	all
CD4+ memory T-cells:	all
CD8+ T-cells:	decreased
Eosinophils:	all
Macrophages:	all
Mesenchymal stem cells:	all
Natural killer T-cells:	all
Regulatory T-cells:	all
Type 1 T-helper cells:	all
Type 2 T-helper cells:	all

Results

P value: 0.0014
FDR: 20%

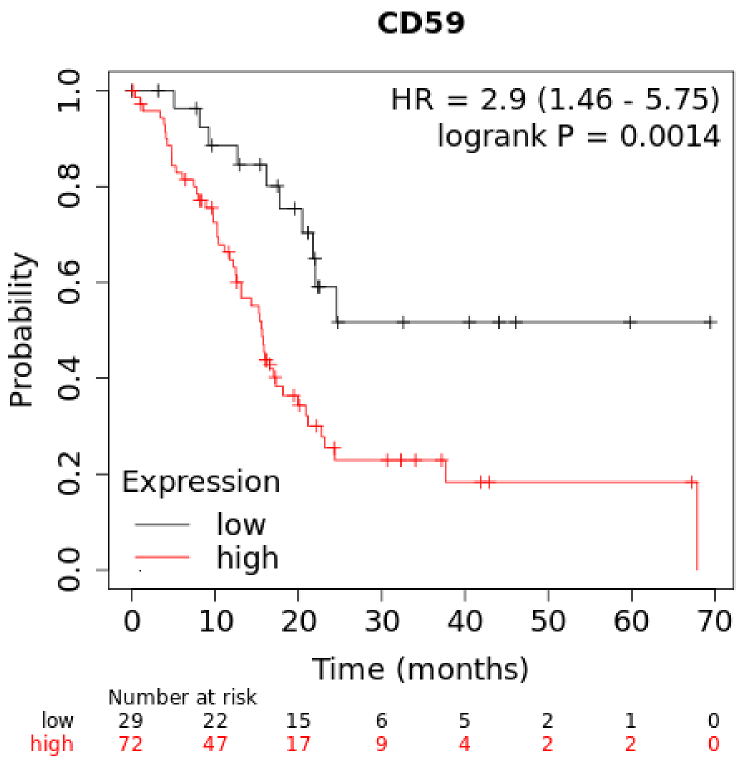

Click here to download the plot in TIFF format

Download plot as a PDF

Download p values vs. cutoff table

Upper quartile survival

Low expression cohort (months)	High expression cohort (months)
20.47	9.73

**RNAseq ID:**	LY6G6C -
**Survival:**	OS
**Auto select best cutoff:**	checked
**Follow up threshold:**	all
**Censore at threshold:**	checked
**Compute median over entire database:**	false
**Cutoff value used in analysis:**	21
**Expression range of the probe:**	0 - 142
**Invert HR values below 1:**	not checked

Restrictions

Tumor type: Pancreatic ductal adenocarcinoma

Restrict analysis to subtypes...

Stage:	all
Gender:	all
Race:	all
Grade:	all
Mutation burden:	all

Restrict analysis based on cellular content...

Basophils:	all
B-cells:	all
CD4+ memory T-cells:	all
CD8+ T-cells:	decreased
Eosinophils:	all
Macrophages:	all
Mesenchymal stem cells:	all
Natural killer T-cells:	all
Regulatory T-cells:	all
Type 1 T-helper cells:	all
Type 2 T-helper cells:	all

Results

**P value:**	0.0147
**FDR:**	over 50%

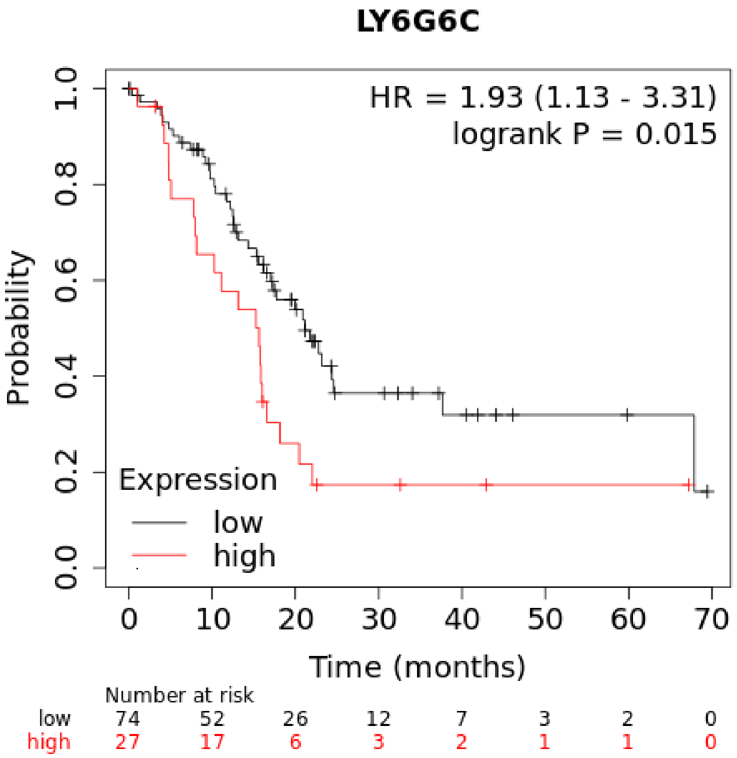

Click here to download the plot in TIFF format

Download plot as a PDF

Download p values vs. cutoff table

Median survival

Low expression cohort (months)	High expression cohort (months)
21.13	15.67

**RNAseq ID:**	LY6G6D -
**Survival:**	OS
**Auto select best cutoff:**	checked
**Follow up threshold:**	all
**Censore at threshold:**	checked
**Compute median over entire database:**	false
**Cutoff value used in analysis:**	0
**Expression range of the probe:**	0 - 1
**Invert HR values below 1:**	not checked

Restrictions

Tumor type: Pancreatic ductal adenocarcinoma

Restrict analysis to subtypes...

Stage:	all
Gender:	all
Race:	all
Grade:	all
Mutation burden:	all

Restrict analysis based on cellular content...

Basophils: all

B-cells:	all
CD4+ memory T-cells:	all
CD8+ T-cells:	decreased
Eosinophils:	all
Macrophages:	all
Mesenchymal stem cells:	all
Natural killer T-cells:	all
Regulatory T-cells:	all
Type 1 T-helper cells:	all
Type 2 T-helper cells:	all

Results

P value: 0.0141
FDR: 50%

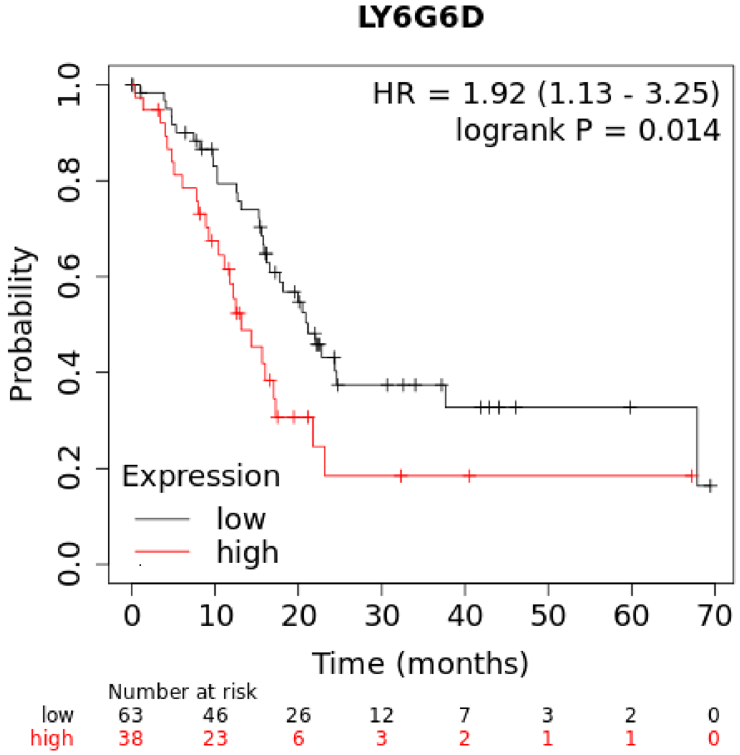

Click here to download the plot in TIFF format

Download plot as a PDF

Download p values vs. cutoff table

Median survival

Low expression cohort (months)	High expression cohort (months)
21.13	13.13

**RNAseq ID:**	LY6G6F -
**Survival:**	OS
**Auto select best cutoff:**	checked
**Follow up threshold:**	all
**Censore at threshold:**	checked
**Compute median over entire database:**	false
**Cutoff value used in analysis:**	0
**Expression range of the probe:**	0 - 2
**Invert HR values below 1:**	not checked

Restrictions

Tumor type: Pancreatic ductal adenocarcinoma

Restrict analysis to subtypes...

Stage:	all
Gender:	all
Race:	all
Grade:	all
Mutation burden:	all

Restrict analysis based on cellular content...

Basophils:	all
B-cells:	all
CD4+ memory T-cells:	all
CD8+ T-cells:	decreased
Eosinophils:	all
Macrophages:	all
Mesenchymal stem cells:	all
Natural killer T-cells:	all
Regulatory T-cells:	all
Type 1 T-helper cells:	all
Type 2 T-helper cells:	all

Results

P value: 0.0064
FDR: over 50%

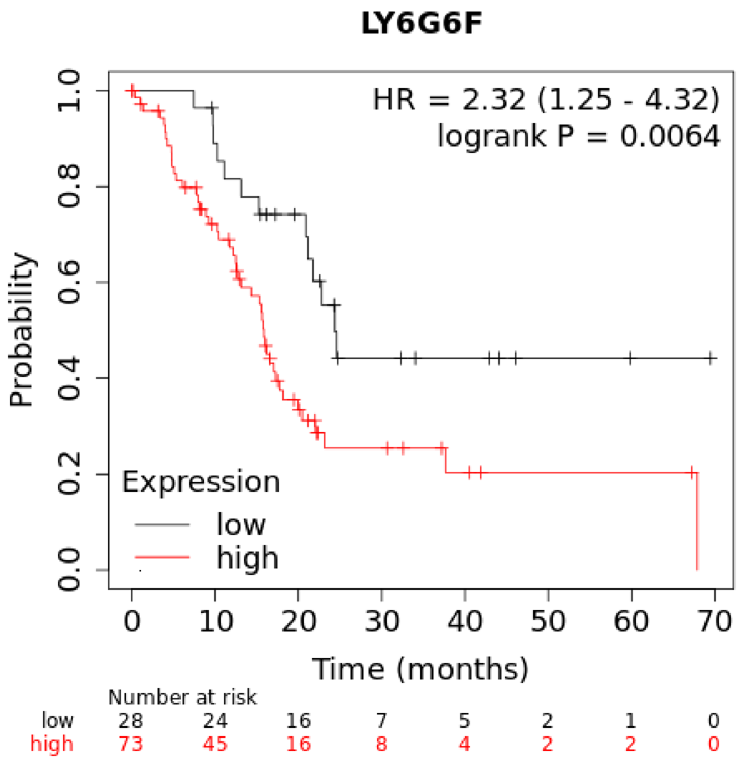

Click here to download the plot in TIFF format

Download plot as a PDF

Download p values vs. cutoff table

Median survival

Low expression cohort (months)	High expression cohort (months)
24.4	15.87

**RNAseq ID:**	LY6G5C -
**Survival:**	OS
**Auto select best cutoff:**	checked
**Follow up threshold:**	all
**Censore at threshold:**	checked
**Compute median over entire database:**	false
**Cutoff value used in analysis:**	51
**Expression range of the probe:**	15 - 586
**Invert HR values below 1:**	not checked

Restrictions

Tumor type: Pancreatic ductal adenocarcinoma

Restrict analysis to subtypes...

Stage:	all
Gender:	all
Race:	all
Grade:	all
Mutation burden:	all

Restrict analysis based on cellular content...

Basophils:	all
B-cells:	all
CD4+ memory T-cells:	all
CD8+ T-cells:	decreased
Eosinophils:	all
Macrophages:	all
Mesenchymal stem cells:	all
Natural killer T-cells:	all
Regulatory T-cells:	all
Type 1 T-helper cells:	all
Type 2 T-helper cells:	all

Results

**P value:**	0.0026
**FDR:**	20%

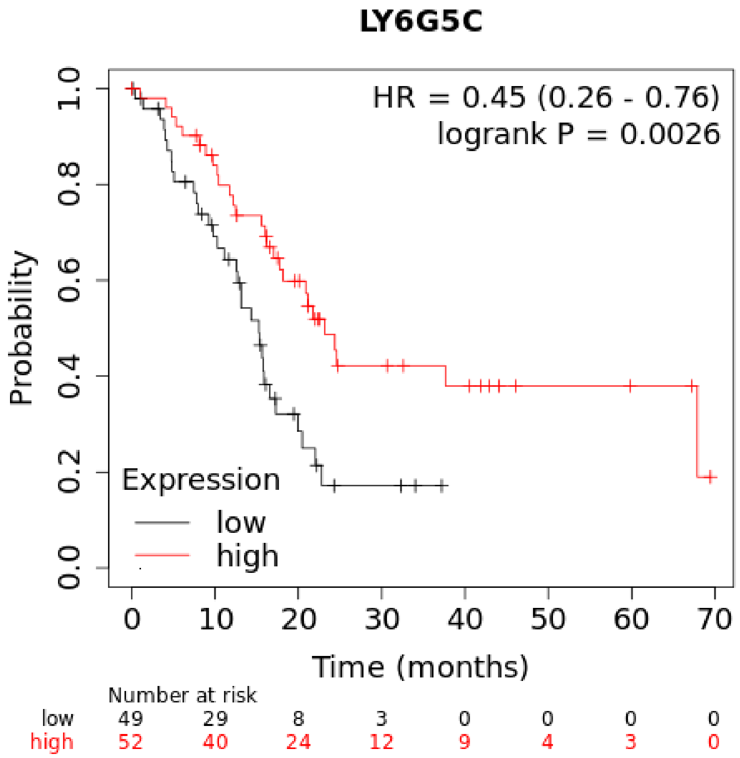

Click here to download the plot in TIFF format

Download plot as a PDF

Download p values vs. cutoff table

Median survival

Low expression cohort (months)	High expression cohort (months)
15.27	23.17

**RNAseq ID:**	LY6G5B -
**Survival:**	OS
**Auto select best cutoff:**	checked
**Follow up threshold:**	all
**Censore at threshold:**	checked
**Compute median over entire database:**	false
**Cutoff value used in analysis:**	61
**Expression range of the probe:**	3 - 192
**Invert HR values below 1:**	not checked

Restrictions

Tumor type: Pancreatic ductal adenocarcinoma

Restrict analysis to subtypes...

Stage:	all
Gender:	all
Race:	all
Grade:	all
Mutation burden:	all

Restrict analysis based on cellular content...

Basophils:	all

B-cells:	all
CD4+ memory T-cells:	all
CD8+ T-cells:	decreased
Eosinophils:	all
Macrophages:	all
Mesenchymal stem cells:	all
Natural killer T-cells:	all
Regulatory T-cells:	all
Type 1 T-helper cells:	all
Type 2 T-helper cells:	all

Results

P value: 0.0049
FDR: 50%

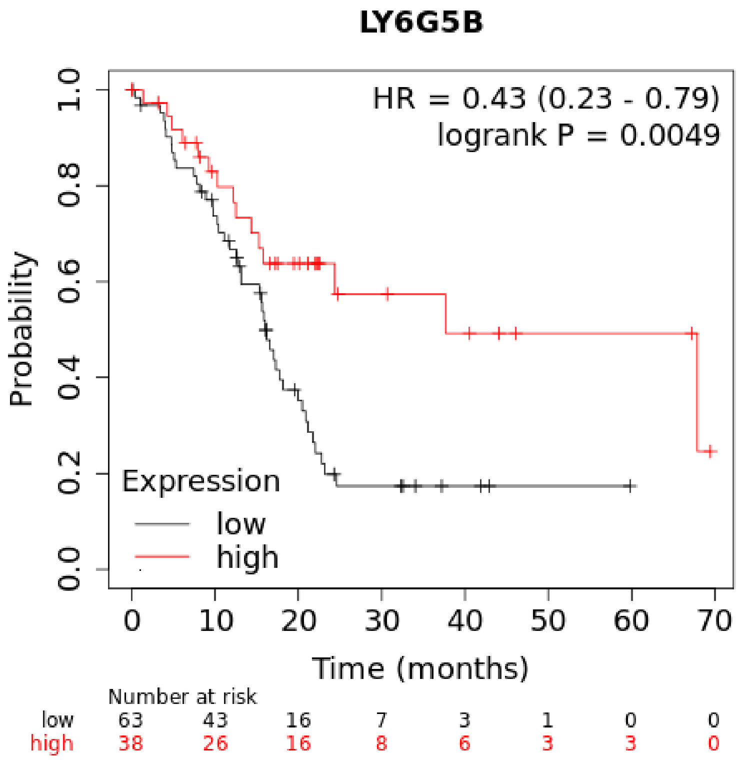

Click here to download the plot in TIFF format

Download plot as a PDF

Download p values vs. cutoff table

Median survival

Low expression cohort (months)	High expression cohort (months)
16.03	37.67

You can save the plots by right-clicking the image and then selecting "Save image as...". To generate a high resolution TIFF image, please adjust the "Settings" in the analysis page.

KMplot.com Copyright © 2009-2020 | Contact | Sitemap

Figure S5: KM plots and other raw data for the data depicted in Table 5

Pan-cancer

KM plotter | Home | Vote | Download | Updates | Contact

The desired RNAseq ID is valid: PSCA (-), LY6K (-), SLURP1 (-), LYPD2 (-), LY6D (-), GML (-), LY6E (-), LY6L (-), LY6H (-), GPIHBP1 (-), LYPD4 (-), CD177 (-), TEX101 (-), LYPD3 (-), PINLYP (-), PLAUR (-), LYPD5 (-), SPACA4 (-), ACRV1 (-), PATE1 (-), PATE2 (-), PATE3 (-), PATE4 (-), CD59 (-), LY6G6C (-), LY6G6D (-), LY6G6F (-), LY6G5C (-), LY6G5B (-),

**RNAseq ID:**	PSCA -
**Survival:**	OS
**Auto select best cutoff:**	checked
**Follow up threshold:**	all
**Censore at threshold:**	checked
**Compute median over entire database:**	false
**Cutoff value used in analysis:**	350
**Expression range of the probe:**	5 - 49321
**Invert HR values below 1:**	not checked

Restrictions

Tumor type: Pancreatic ductal adenocarcinoma

Restrict analysis to subtypes...

Stage:	all
Gender:	all
Race:	all
Grade:	all
Mutation burden:	all

Restrict analysis based on cellular content...

Basophils:	all
B-cells:	all
CD4+ memory T-cells:	all
CD8+ T-cells:	all
Eosinophils:	all
Macrophages:	enriched
Mesenchymal stem cells:	all
Natural killer T-cells:	all
Regulatory T-cells:	all
Type 1 T-helper cells:	all
Type 2 T-helper cells:	all

Results

P value: 0.0949
FDR: 100%

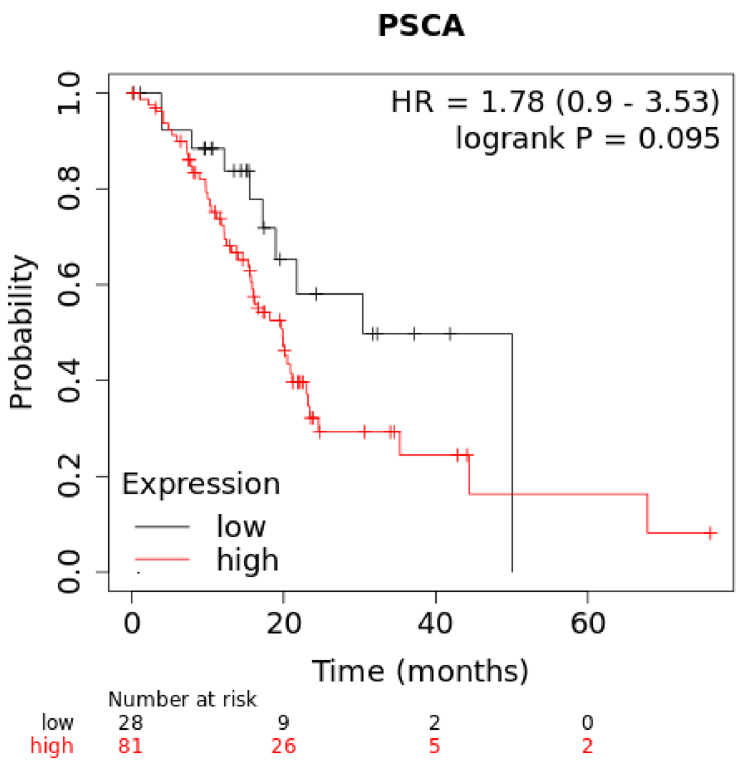

Click here to download the plot in TIFF format

Download plot as a PDF

Download p values vs. cutoff table

Median survival

Low expression cohort (months)	High expression cohort (months)
30.43	19.87

**RNAseq ID:**	LY6K -
**Survival:**	OS
**Auto select best cutoff:**	checked
**Follow up threshold:**	all
**Censore at threshold:**	checked
**Compute median over entire database:**	false
**Cutoff value used in analysis:**	24
**Expression range of the probe:**	0 - 1825
**Invert HR values below 1:**	not checked

Restrictions

Tumor type: Pancreatic ductal adenocarcinoma

Restrict analysis to subtypes...

Stage:	all
Gender:	all
Race:	all
Grade:	all
Mutation burden:	all

Restrict analysis based on cellular content...

Basophils:	all

B-cells:	all
CD4+ memory T-cells:	all
CD8+ T-cells:	all
Eosinophils:	all
Macrophages:	enriched
Mesenchymal stem cells:	all
Natural killer T-cells:	all
Regulatory T-cells:	all
Type 1 T-helper cells:	all
Type 2 T-helper cells:	all

Results

P value: 0.144
FDR: 100%

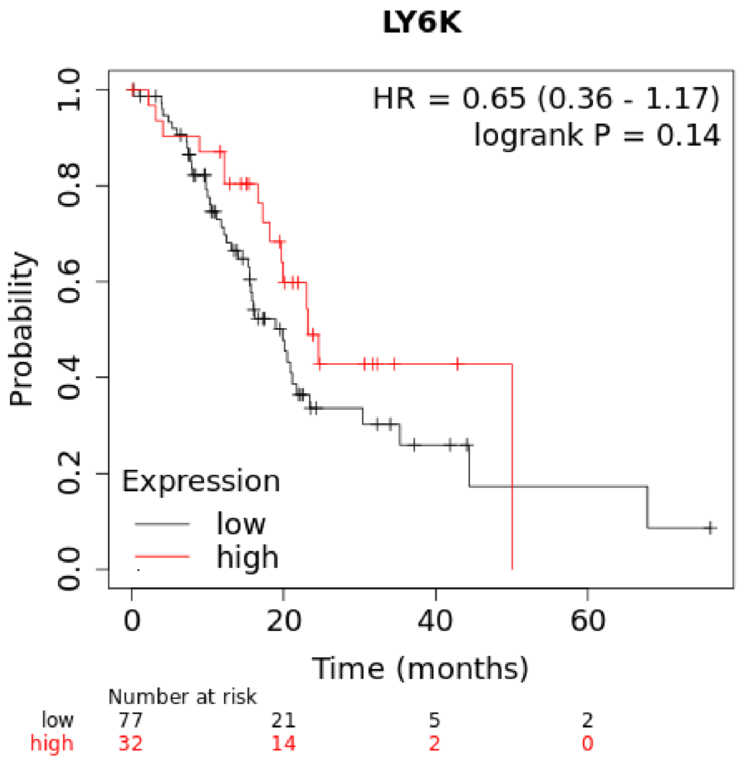

Click here to download the plot in TIFF format

Download plot as a PDF

Download p values vs. cutoff table

Median survival

Low expression cohort (months)	High expression cohort (months)
19.87	23.17

**RNAseq ID:**	SLURP1 -
**Survival:**	OS
**Auto select best cutoff:**	checked
**Follow up threshold:**	all
**Censore at threshold:**	checked
**Compute median over entire database:**	false
**Cutoff value used in analysis:**	1
**Expression range of the probe:**	0 - 105
**Invert HR values below 1:**	not checked

Restrictions

Tumor type: Pancreatic ductal adenocarcinoma

Restrict analysis to subtypes...

Stage: all
Gender: all
Race: all
Grade: all
Mutation burden: all

Restrict analysis based on cellular content...

Basophils: all
B-cells: all
CD4+ memory T-cells: all
CD8+ T-cells: all
Eosinophils: all
Macrophages: enriched
Mesenchymal stem cells: all
Natural killer T-cells: all
Regulatory T-cells: all
Type 1 T-helper cells: all
Type 2 T-helper cells: all

Results

P value: 0.017
FDR: over 50%

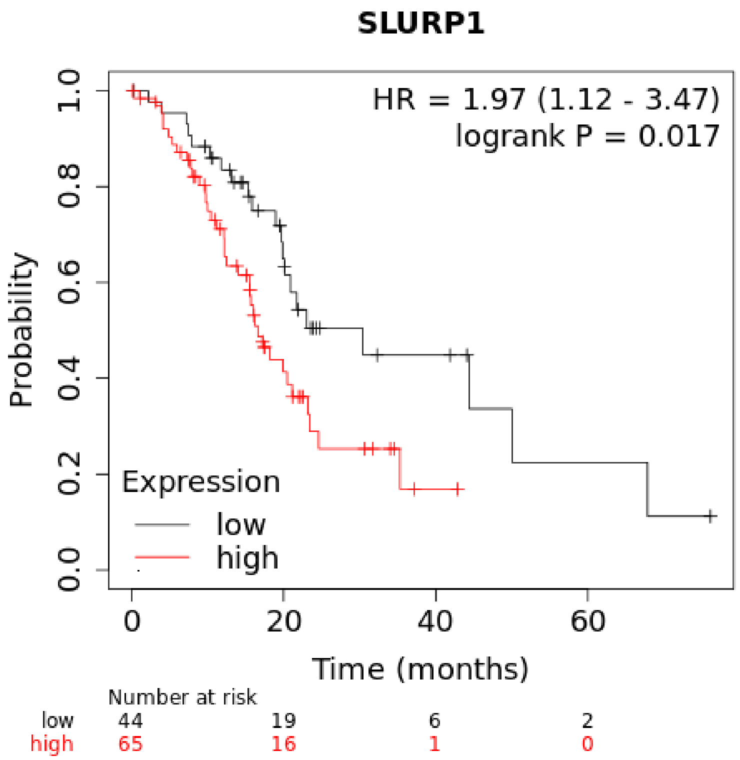

Click here to download the plot in TIFF format

Download plot as a PDF

Download p values vs. cutoff table

Median survival

Low expression cohort (months)	High expression cohort (months)
30.43	16.6

**RNAseq ID:**	LYPD2 -
**Survival:**	OS
**Auto select best cutoff:**	checked
**Follow up threshold:**	all
**Censore at threshold:**	checked
**Compute median over entire database:**	false
**Cutoff value used in analysis:**	2
**Expression range of the probe:**	0 - 4748
**Invert HR values below 1:**	not checked

Restrictions

Tumor type: Pancreatic ductal adenocarcinoma

Restrict analysis to subtypes...

Stage:	all
Gender:	all
Race:	all
Grade:	all
Mutation burden:	all

Restrict analysis based on cellular content...

Basophils:	all
B-cells:	all
CD4+ memory T-cells:	all
CD8+ T-cells:	all
Eosinophils:	all
Macrophages:	enriched
Mesenchymal stem cells:	all
Natural killer T-cells:	all
Regulatory T-cells:	all
Type 1 T-helper cells:	all
Type 2 T-helper cells:	all

Results

**P value:**	0.2728
**FDR:**	100%

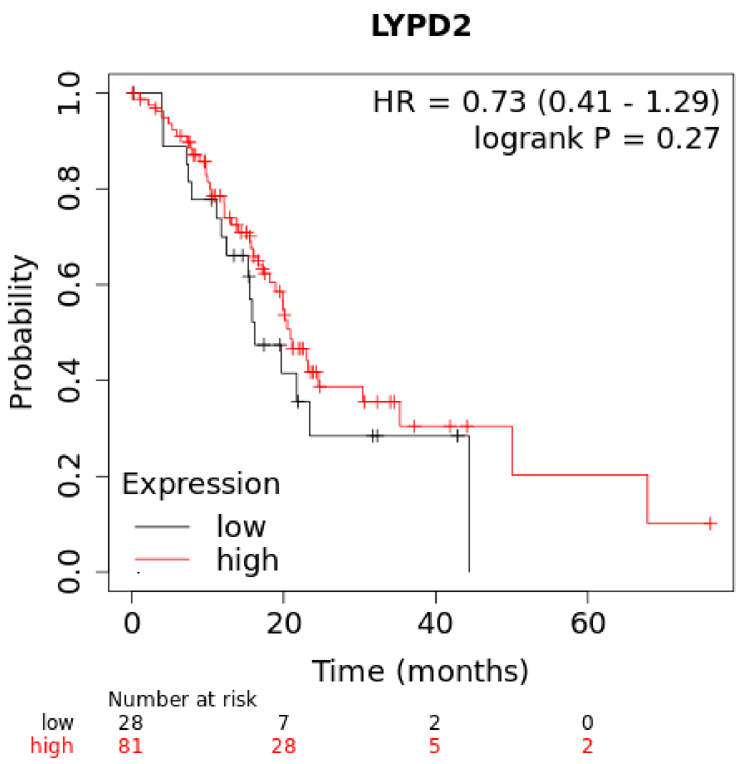

Click here to download the plot in TIFF format

Download plot as a PDF

Download p values vs. cutoff table

Median survival

Low expression cohort (months)	High expression cohort (months)
16.2	20.9

**RNAseq ID:**	LY6D -
**Survival:**	OS
**Auto select best cutoff:**	checked
**Follow up threshold:**	all
**Censore at threshold:**	checked
**Compute median over entire database:**	false
**Cutoff value used in analysis:**	188
**Expression range of the probe:**	0 - 12992
**Invert HR values below 1:**	not checked

Restrictions

Tumor type: Pancreatic ductal adenocarcinoma

Restrict analysis to subtypes...

Stage:	all
Gender:	all
Race:	all
Grade:	all
Mutation burden:	all

Restrict analysis based on cellular content...

Basophils:	all

B-cells:	all
CD4+ memory T-cells:	all
CD8+ T-cells:	all
Eosinophils:	all
Macrophages:	enriched
Mesenchymal stem cells:	all
Natural killer T-cells:	all
Regulatory T-cells:	all
Type 1 T-helper cells:	all
Type 2 T-helper cells:	all

Results

P value: 0.0002
FDR: 2%

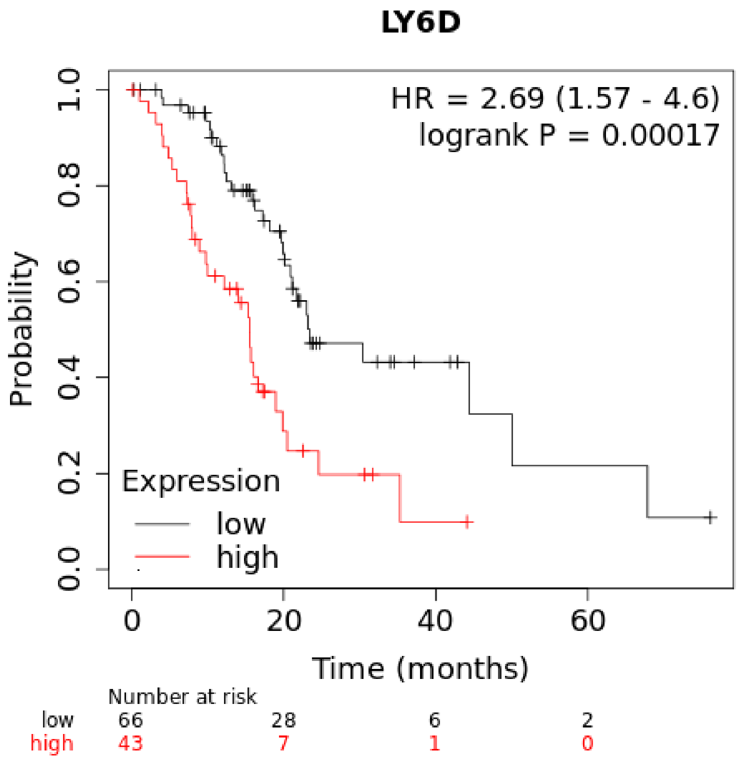

Click here to download the plot in TIFF format

Download plot as a PDF

Download p values vs. cutoff table

Median survival

Low expression cohort (months)	High expression cohort (months)
23.4	15.53

**RNAseq ID:**	GML -
**Survival:**	OS
**Auto select best cutoff:**	checked
**Follow up threshold:**	all
**Censore at threshold:**	checked
**Compute median over entire database:**	false
**Cutoff value used in analysis:**	0
**Expression range of the probe:**	0 - 3
**Invert HR values below 1:**	not checked

Restrictions

Tumor type: Pancreatic ductal adenocarcinoma

Restrict analysis to subtypes...

Stage:	all
Gender:	all
Race:	all
Grade:	all
Mutation burden:	all

Restrict analysis based on cellular content...

Basophils:	all
B-cells:	all
CD4+ memory T-cells:	all
CD8+ T-cells:	all
Eosinophils:	all
Macrophages:	enriched
Mesenchymal stem cells:	all
Natural killer T-cells:	all
Regulatory T-cells:	all
Type 1 T-helper cells:	all
Type 2 T-helper cells:	all

Results

P value: 0.4457
FDR: 100%

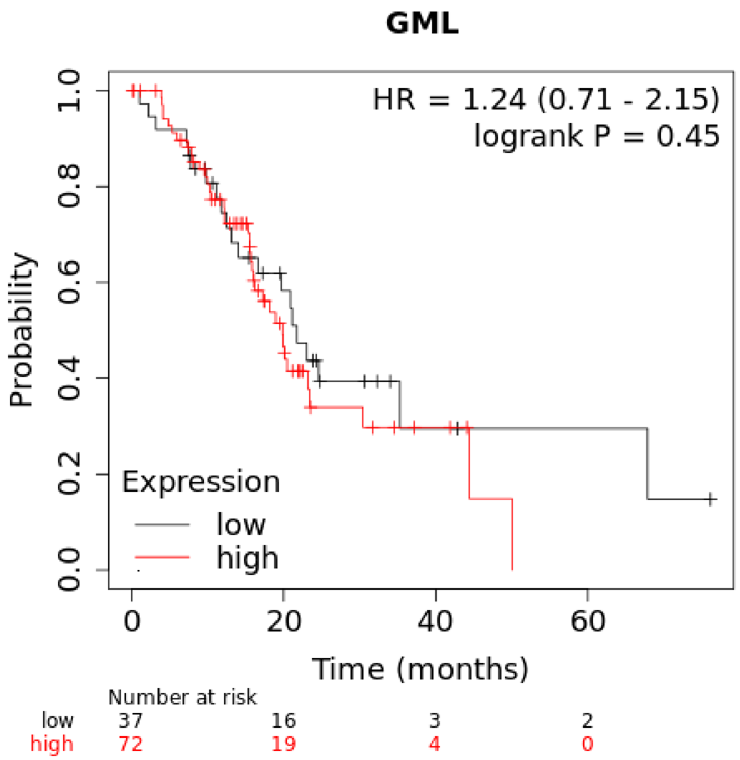

Click here to download the plot in TIFF format

Download plot as a PDF

Download p values vs. cutoff table

Median survival

Low expression cohort (months)	High expression cohort (months)
21.73	19.87

**RNAseq ID:**	LY6E -
**Survival:**	OS
**Auto select best cutoff:**	checked
**Follow up threshold:**	all
**Censore at threshold:**	checked
**Compute median over entire database:**	false
**Cutoff value used in analysis:**	12303
**Expression range of the probe:**	2373 - 49379
**Invert HR values below 1:**	not checked

Restrictions

Tumor type: Pancreatic ductal adenocarcinoma

Restrict analysis to subtypes...

Stage:	all
Gender:	all
Race:	all
Grade:	all
Mutation burden:	all

Restrict analysis based on cellular content...

Basophils:	all
B-cells:	all
CD4+ memory T-cells:	all
CD8+ T-cells:	all
Eosinophils:	all
Macrophages:	enriched
Mesenchymal stem cells:	all
Natural killer T-cells:	all
Regulatory T-cells:	all
Type 1 T-helper cells:	all
Type 2 T-helper cells:	all

Results

**P value:**	0.1422
**FDR:**	100%

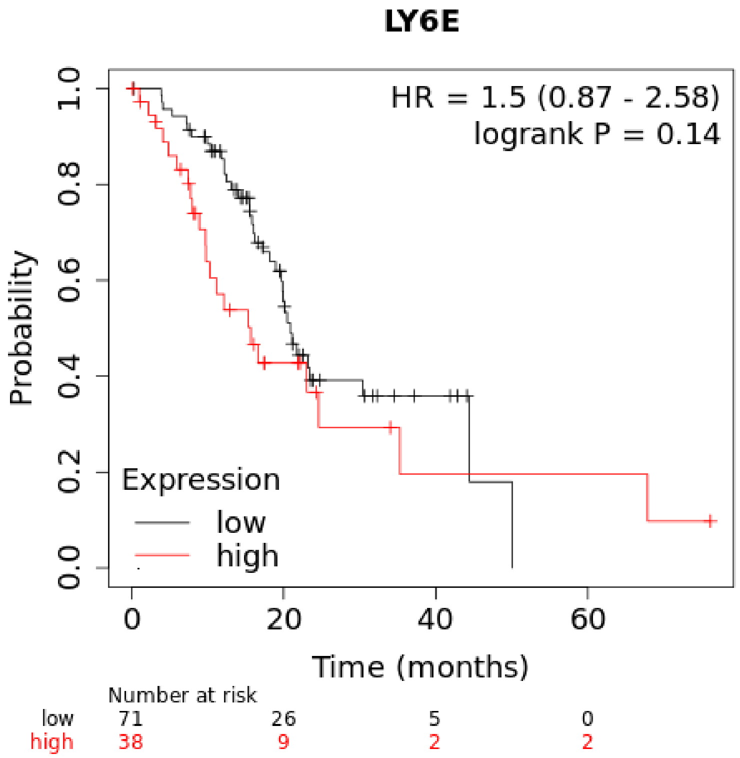

Click here to download the plot in TIFF format

Download plot as a PDF

Download p values vs. cutoff table

Median survival

Low expression cohort (months)	High expression cohort (months)
20.9	15.67

RNAseq ID: LY6L -
Survival: OS
Auto select best cutoff: checked
Follow up threshold: all
Censore at threshold: checked
Compute median over entire database: false
Cutoff value used in analysis: 0
Expression range of the probe: 0 - 2
Invert HR values below 1: not checked

Restrictions

Tumor type: Pancreatic ductal adenocarcinoma

Restrict analysis to subtypes...

Stage: all
Gender: all
Race: all
Grade: all
Mutation burden: all

Restrict analysis based on cellular content...

Basophils: all

B-cells:	all
CD4+ memory T-cells:	all
CD8+ T-cells:	all
Eosinophils:	all
Macrophages:	enriched
Mesenchymal stem cells:	all
Natural killer T-cells:	all
Regulatory T-cells:	all
Type 1 T-helper cells:	all
Type 2 T-helper cells:	all

Results

P value: 0.0412
FDR: over 50%

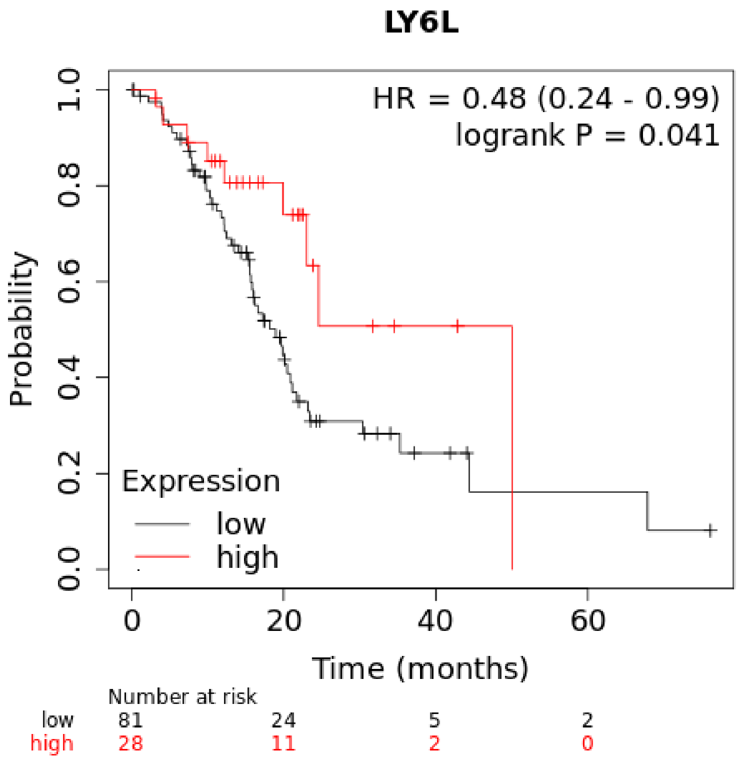

[Click here to download the plot in TIFF format]

[Download plot as a PDF]

[Download p values vs. cutoff table]

Median survival

Low expression cohort (months)	High expression cohort (months)
18.93	50.07

**RNAseq ID:**	LY6H -
**Survival:**	OS
**Auto select best cutoff:**	checked
**Follow up threshold:**	all
**Censore at threshold:**	checked
**Compute median over entire database:**	false
**Cutoff value used in analysis:**	28
**Expression range of the probe:**	4 - 250
**Invert HR values below 1:**	not checked

Restrictions

Tumor type: Pancreatic ductal adenocarcinoma

Restrict analysis to subtypes...

Stage:	all
Gender:	all
Race:	all
Grade:	all
Mutation burden:	all

Restrict analysis based on cellular content...

Basophils:	all
B-cells:	all
CD4+ memory T-cells:	all
CD8+ T-cells:	all
Eosinophils:	all
Macrophages:	enriched
Mesenchymal stem cells:	all
Natural killer T-cells:	all
Regulatory T-cells:	all
Type 1 T-helper cells:	all
Type 2 T-helper cells:	all

Results

P value: 0.0783
FDR: 100%

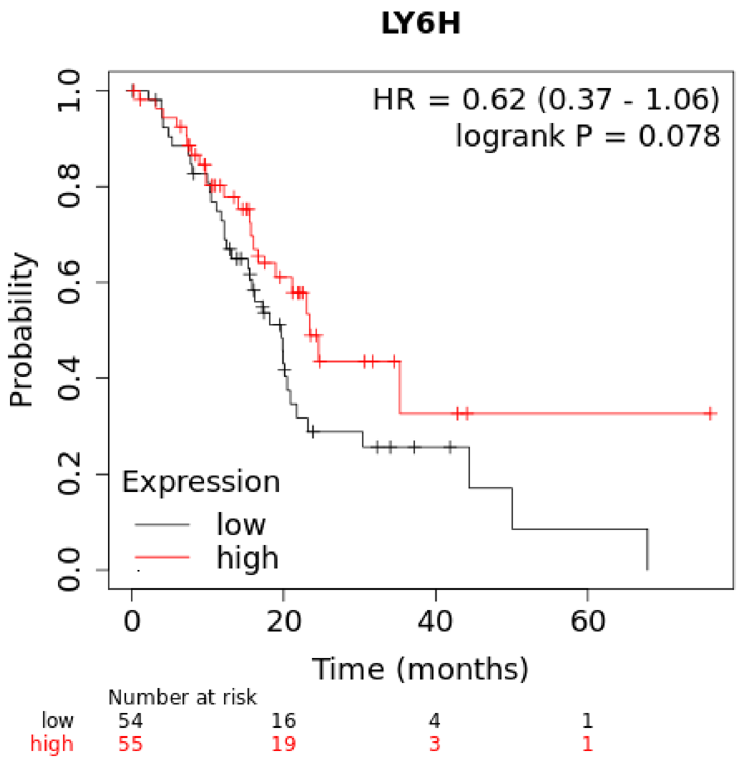

Click here to download the plot in TIFF format

Download plot as a PDF

Download p values vs. cutoff table

Median survival

Low expression cohort (months)	High expression cohort (months)
19.73	23.4

**RNAseq ID:**	GPIHBP1 -
**Survival:**	OS
**Auto select best cutoff:**	checked
**Follow up threshold:**	all
**Censore at threshold:**	checked
**Compute median over entire database:**	false
**Cutoff value used in analysis:**	54
**Expression range of the probe:**	5 - 323
**Invert HR values below 1:**	not checked

Restrictions

Tumor type: Pancreatic ductal adenocarcinoma

Restrict analysis to subtypes...

Stage:	all
Gender:	all
Race:	all
Grade:	all
Mutation burden:	all

Restrict analysis based on cellular content...

Basophils:	all
B-cells:	all
CD4+ memory T-cells:	all
CD8+ T-cells:	all
Eosinophils:	all
Macrophages:	enriched
Mesenchymal stem cells:	all
Natural killer T-cells:	all
Regulatory T-cells:	all
Type 1 T-helper cells:	all
Type 2 T-helper cells:	all

Results

**P value:**	0.3193
**FDR:**	100%

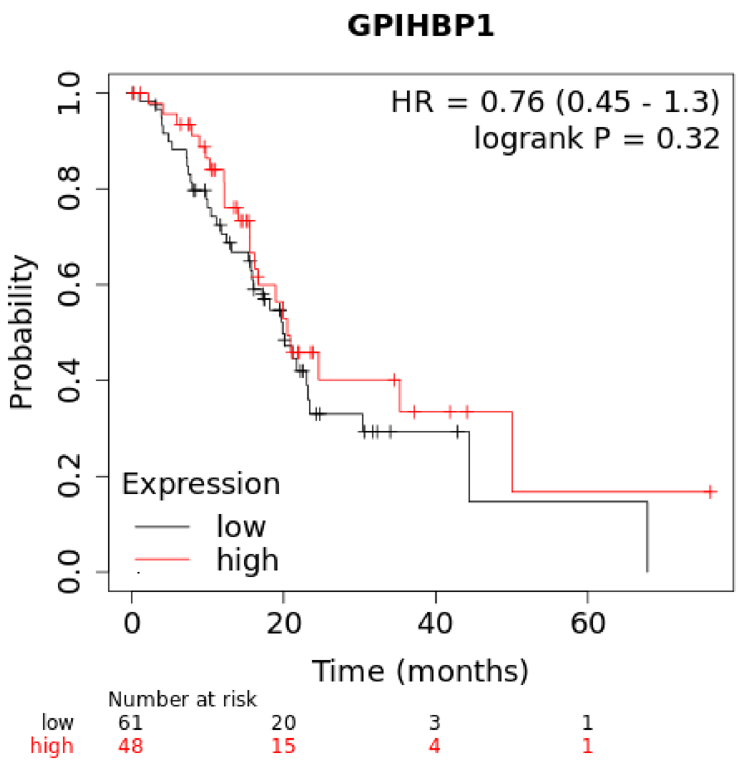

Click here to download the plot in TIFF format

Download plot as a PDF

Download p values vs. cutoff table

Median survival

Low expression cohort (months)	High expression cohort (months)
19.87	20.47

**RNAseq ID:**	LYPD4 -
**Survival:**	OS
**Auto select best cutoff:**	checked
**Follow up threshold:**	all
**Censore at threshold:**	checked
**Compute median over entire database:**	false
**Cutoff value used in analysis:**	0
**Expression range of the probe:**	0 - 11
**Invert HR values below 1:**	not checked

Restrictions

Tumor type: Pancreatic ductal adenocarcinoma

Restrict analysis to subtypes...

Stage:	all
Gender:	all
Race:	all
Grade:	all
Mutation burden:	all

Restrict analysis based on cellular content...

Basophils: all

B-cells:	all
CD4+ memory T-cells:	all
CD8+ T-cells:	all
Eosinophils:	all
Macrophages:	enriched
Mesenchymal stem cells:	all
Natural killer T-cells:	all
Regulatory T-cells:	all
Type 1 T-helper cells:	all
Type 2 T-helper cells:	all

Results

P value: 0.0215
FDR: over 50%

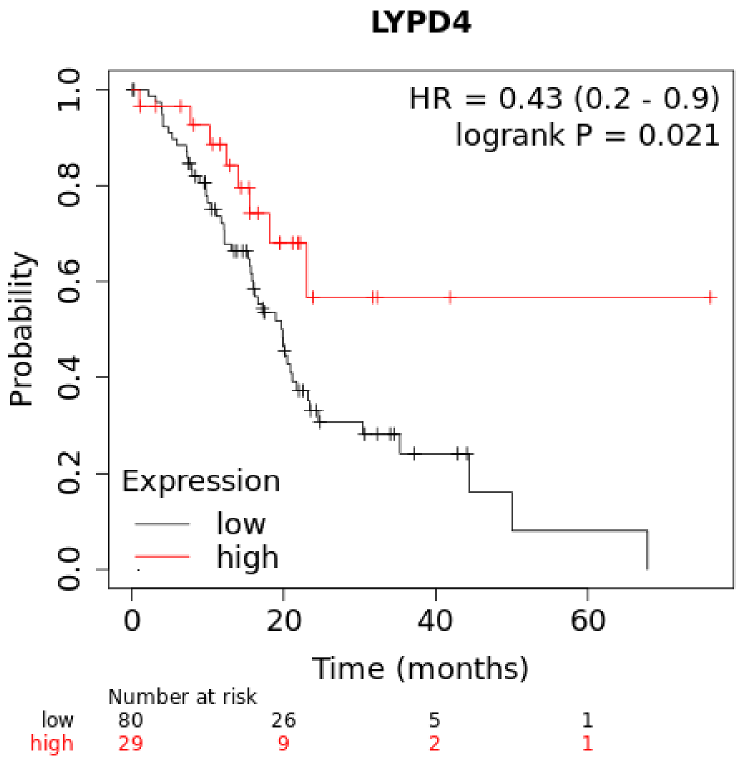

Click here to download the plot in TIFF format

Download plot as a PDF

Download p values vs. cutoff table

Upper quartile survival

Low expression cohort (months)	High expression cohort (months)
11.13	15.53

**RNAseq ID:**	CD177 -
**Survival:**	OS
**Auto select best cutoff:**	checked
**Follow up threshold:**	all
**Censore at threshold:**	checked
**Compute median over entire database:**	false
**Cutoff value used in analysis:**	31
**Expression range of the probe:**	1 - 6100
**Invert HR values below 1:**	not checked

Restrictions

Tumor type: Pancreatic ductal adenocarcinoma

Restrict analysis to subtypes...

Stage: all
Gender: all
Race: all
Grade: all
Mutation burden: all

Restrict analysis based on cellular content...

Basophils: all
B-cells: all
CD4+ memory T-cells: all
CD8+ T-cells: all
Eosinophils: all
Macrophages: enriched
Mesenchymal stem cells: all
Natural killer T-cells: all
Regulatory T-cells: all
Type 1 T-helper cells: all
Type 2 T-helper cells: all

Results

P value: 0.1163
FDR: 100%

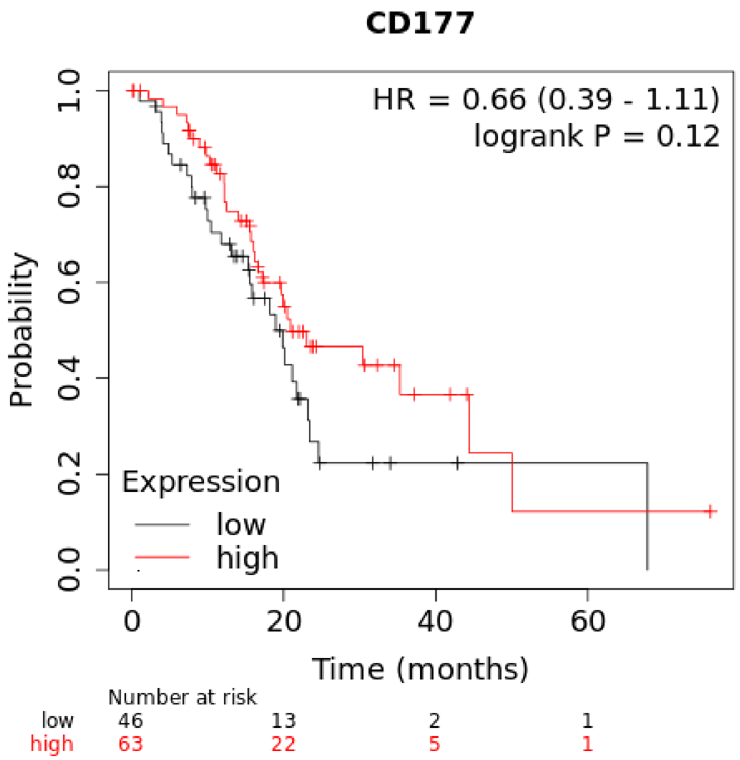

Click here to download the plot in TIFF format

Download plot as a PDF

Download p values vs. cutoff table

Median survival

Low expression cohort (months)	High expression cohort (months)
18.93	20.9

**RNAseq ID:**	TEX101 -
**Survival:**	OS
**Auto select best cutoff:**	checked
**Follow up threshold:**	all
**Censore at threshold:**	checked
**Compute median over entire database:**	false
**Cutoff value used in analysis:**	1
**Expression range of the probe:**	0 - 24
**Invert HR values below 1:**	not checked

Restrictions

Tumor type: Pancreatic ductal adenocarcinoma

Restrict analysis to subtypes...

Stage:	all
Gender:	all
Race:	all
Grade:	all
Mutation burden:	all

Restrict analysis based on cellular content...

Basophils:	all
B-cells:	all
CD4+ memory T-cells:	all
CD8+ T-cells:	all
Eosinophils:	all
Macrophages:	enriched
Mesenchymal stem cells:	all
Natural killer T-cells:	all
Regulatory T-cells:	all
Type 1 T-helper cells:	all
Type 2 T-helper cells:	all

Results

**P value:**	0.0957
**FDR:**	100%

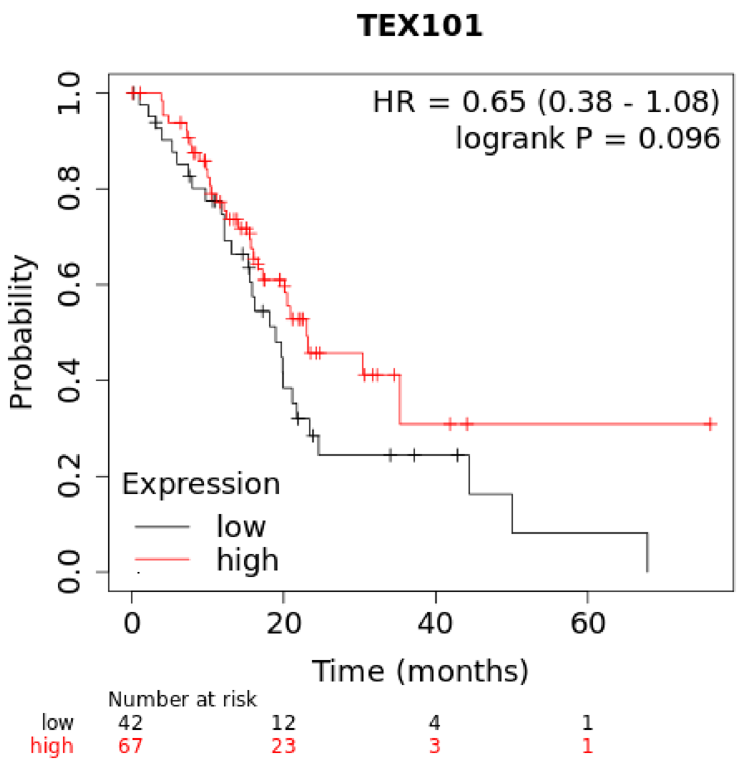

Click here to download the plot in TIFF format

Download plot as a PDF

Download p values vs. cutoff table

Median survival

Low expression cohort (months)	High expression cohort (months)
18.93	23.03

RNAseq ID: LYPD3 -
Survival: OS
Auto select best cutoff: checked
Follow up threshold: all
Censore at threshold: checked
Compute median over entire database: false
Cutoff value used in analysis: 279
Expression range of the probe: 14 - 7684
Invert HR values below 1: not checked

Restrictions

Tumor type: Pancreatic ductal adenocarcinoma

Restrict analysis to subtypes...

Stage: all
Gender: all
Race: all
Grade: all
Mutation burden: all

Restrict analysis based on cellular content...

Basophils: all

B-cells:	all
CD4+ memory T-cells:	all
CD8+ T-cells:	all
Eosinophils:	all
Macrophages:	enriched
Mesenchymal stem cells:	all
Natural killer T-cells:	all
Regulatory T-cells:	all
Type 1 T-helper cells:	all
Type 2 T-helper cells:	all

Results

P value: 0.0293
FDR: over 50%

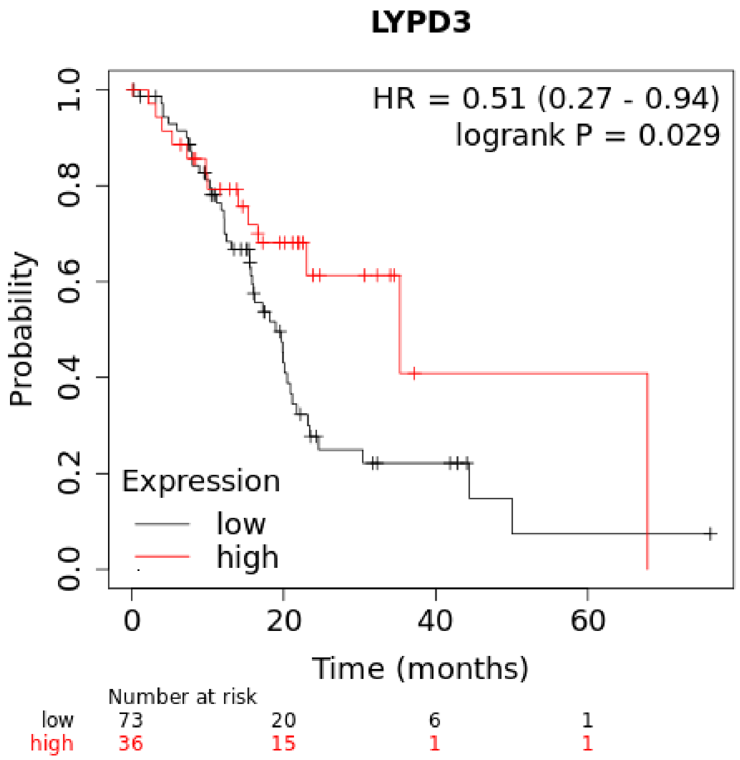

Click here to download the plot in TIFF format

Download plot as a PDF

Download p values vs. cutoff table

Median survival

Low expression cohort (months)	High expression cohort (months)
18.93	35.3

**RNAseq ID:**	PINLYP -
**Survival:**	OS
**Auto select best cutoff:**	checked
**Follow up threshold:**	all
**Censore at threshold:**	checked
**Compute median over entire database:**	false
**Cutoff value used in analysis:**	71
**Expression range of the probe:**	16 - 387
**Invert HR values below 1:**	not checked

Restrictions

Tumor type: Pancreatic ductal adenocarcinoma

Restrict analysis to subtypes...

Stage:	all
Gender:	all
Race:	all
Grade:	all
Mutation burden:	all

Restrict analysis based on cellular content...

Basophils:	all
B-cells:	all
CD4+ memory T-cells:	all
CD8+ T-cells:	all
Eosinophils:	all
Macrophages:	enriched
Mesenchymal stem cells:	all
Natural killer T-cells:	all
Regulatory T-cells:	all
Type 1 T-helper cells:	all
Type 2 T-helper cells:	all

Results

P value: 0.1577
FDR: 100%

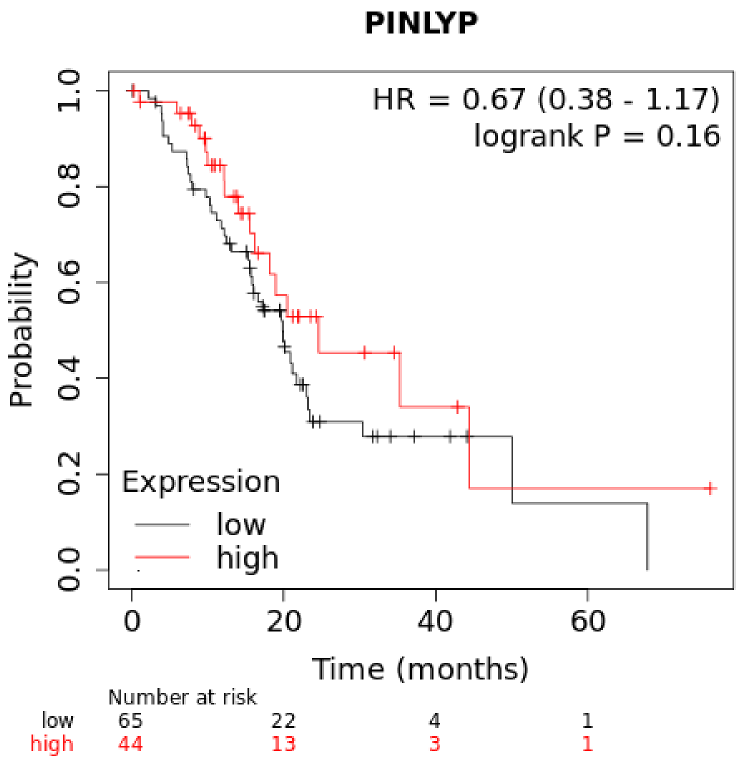

Click here to download the plot in TIFF format

Download plot as a PDF

Download p values vs. cutoff table

Median survival

Low expression cohort (months)	High expression cohort (months)
19.87	24.6

**RNAseq ID:**	PLAUR -
**Survival:**	OS
**Auto select best cutoff:**	checked
**Follow up threshold:**	all
**Censore at threshold:**	checked
**Compute median over entire database:**	false
**Cutoff value used in analysis:**	3873
**Expression range of the probe:**	553 - 18314
**Invert HR values below 1:**	not checked

Restrictions

Tumor type: Pancreatic ductal adenocarcinoma

Restrict analysis to subtypes...

Stage:	all
Gender:	all
Race:	all
Grade:	all
Mutation burden:	all

Restrict analysis based on cellular content...

Basophils:	all
B-cells:	all
CD4+ memory T-cells:	all
CD8+ T-cells:	all
Eosinophils:	all
Macrophages:	enriched
Mesenchymal stem cells:	all
Natural killer T-cells:	all
Regulatory T-cells:	all
Type 1 T-helper cells:	all
Type 2 T-helper cells:	all

Results

**P value:**	0.0693
**FDR:**	100%

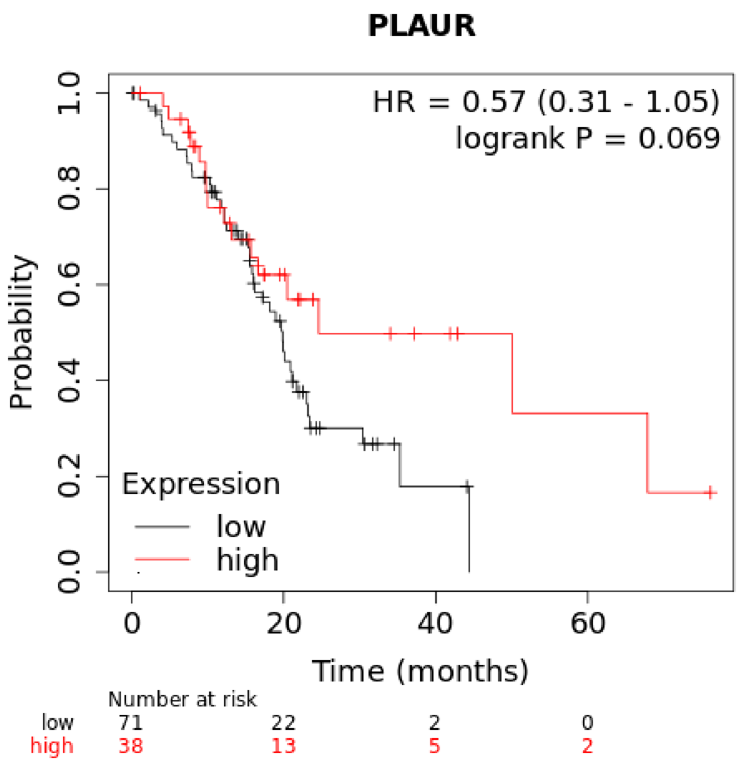

Click here to download the plot in TIFF format

Download plot as a PDF

Download p values vs. cutoff table

Median survival

Low expression cohort (months)	High expression cohort (months)
19.87	24.6

**RNAseq ID:**	LYPD5 -
**Survival:**	OS
**Auto select best cutoff:**	checked
**Follow up threshold:**	all
**Censore at threshold:**	checked
**Compute median over entire database:**	false
**Cutoff value used in analysis:**	66
**Expression range of the probe:**	24 - 419
**Invert HR values below 1:**	not checked

Restrictions

Tumor type: Pancreatic ductal adenocarcinoma

Restrict analysis to subtypes...

Stage:	all
Gender:	all
Race:	all
Grade:	all
Mutation burden:	all

Restrict analysis based on cellular content...

Basophils:	all

B-cells:	all
CD4+ memory T-cells:	all
CD8+ T-cells:	all
Eosinophils:	all
Macrophages:	enriched
Mesenchymal stem cells:	all
Natural killer T-cells:	all
Regulatory T-cells:	all
Type 1 T-helper cells:	all
Type 2 T-helper cells:	all

Results

P value: 0.3482
FDR: 100%

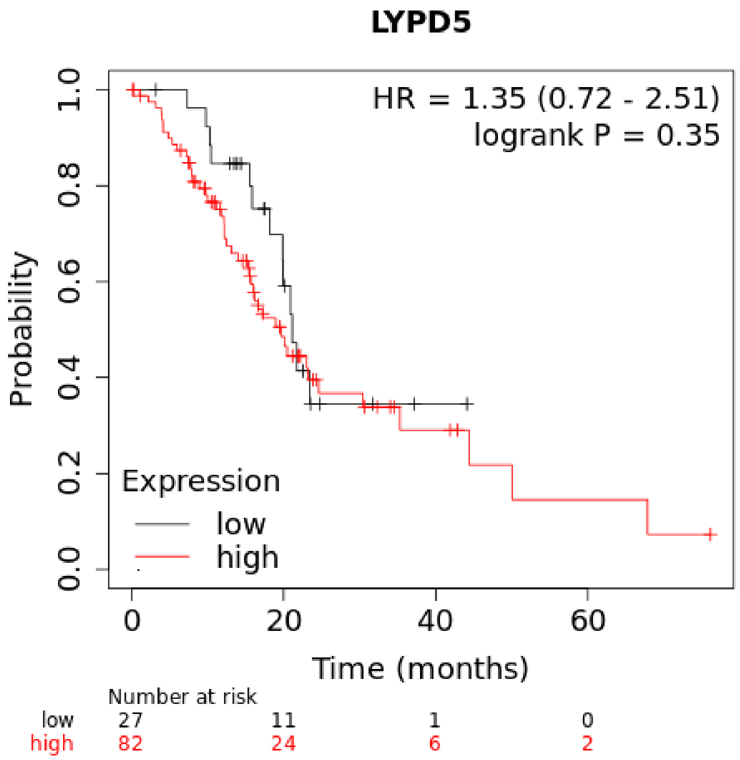

Click here to download the plot in TIFF format

Download plot as a PDF

Download p values vs. cutoff table

Median survival

Low expression cohort (months)	High expression cohort (months)
21.13	19.73

**RNAseq ID:**	SPACA4 -
**Survival:**	OS
**Auto select best cutoff:**	checked
**Follow up threshold:**	all
**Censore at threshold:**	checked
**Compute median over entire database:**	false
**Cutoff value used in analysis:**	8
**Expression range of the probe:**	0 - 206
**Invert HR values below 1:**	not checked

Restrictions

Tumor type: Pancreatic ductal adenocarcinoma

Restrict analysis to subtypes...

Stage:	all
Gender:	all
Race:	all
Grade:	all
Mutation burden:	all

Restrict analysis based on cellular content...

Basophils:	all
B-cells:	all
CD4+ memory T-cells:	all
CD8+ T-cells:	all
Eosinophils:	all
Macrophages:	enriched
Mesenchymal stem cells:	all
Natural killer T-cells:	all
Regulatory T-cells:	all
Type 1 T-helper cells:	all
Type 2 T-helper cells:	all

Results

P value: 0.0191
FDR: over 50%

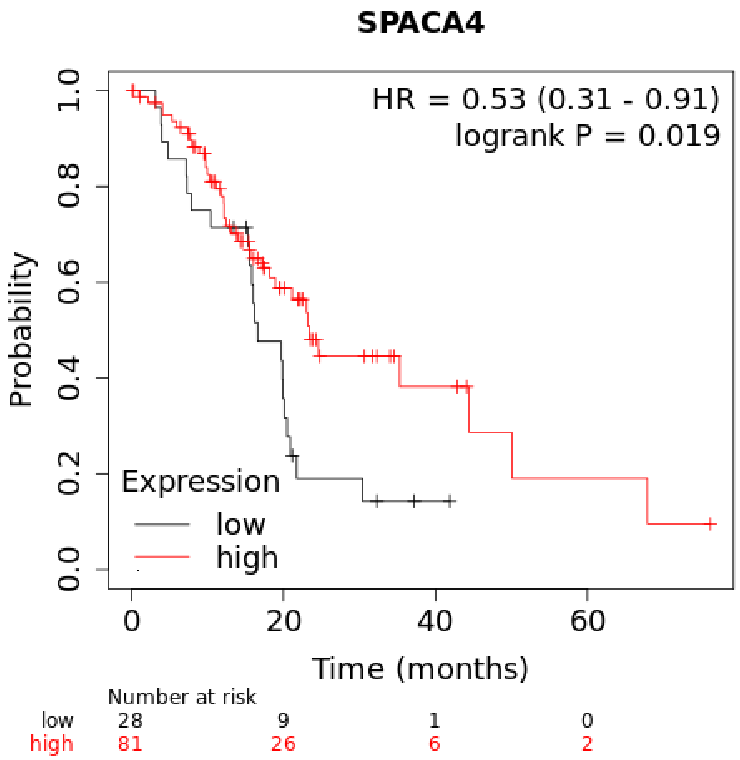

Click here to download the plot in TIFF format

Download plot as a PDF

Download p values vs. cutoff table

Median survival

Low expression cohort (months)	High expression cohort (months)
16.6	23.4

**RNAseq ID:**	ACRV1 -
**Survival:**	OS
**Auto select best cutoff:**	checked
**Follow up threshold:**	all
**Censore at threshold:**	checked
**Compute median over entire database:**	false
**Cutoff value used in analysis:**	4
**Expression range of the probe:**	0 - 71
**Invert HR values below 1:**	not checked

Restrictions

Tumor type: Pancreatic ductal adenocarcinoma

Restrict analysis to subtypes...

Stage:	all
Gender:	all
Race:	all
Grade:	all
Mutation burden:	all

Restrict analysis based on cellular content...

Basophils:	all
B-cells:	all
CD4+ memory T-cells:	all
CD8+ T-cells:	all
Eosinophils:	all
Macrophages:	enriched
Mesenchymal stem cells:	all
Natural killer T-cells:	all
Regulatory T-cells:	all
Type 1 T-helper cells:	all
Type 2 T-helper cells:	all

Results

P value: 0.0937
FDR: 100%

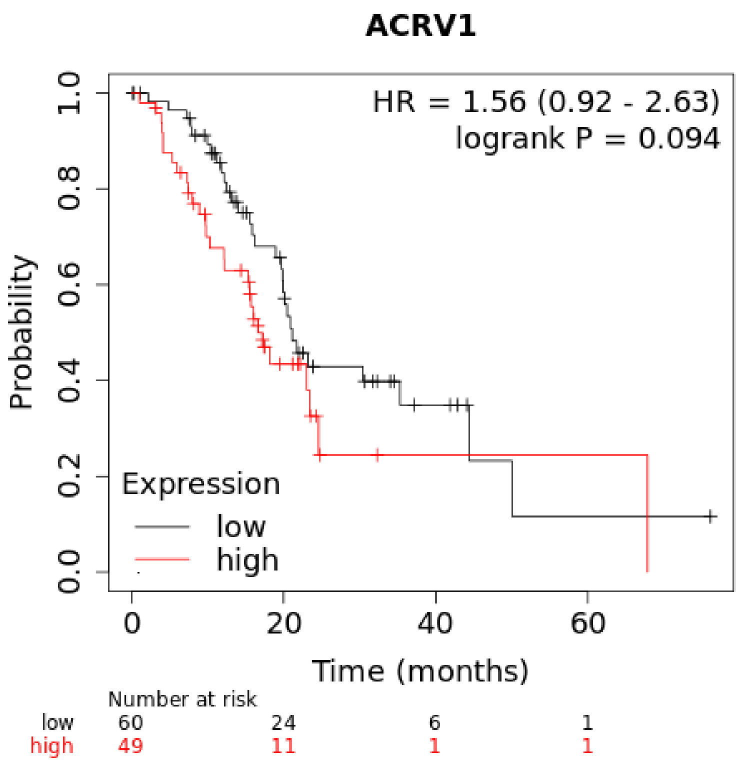

Click here to download the plot in TIFF format

Download plot as a PDF

Download p values vs. cutoff table

Median survival

Low expression cohort (months)	High expression cohort (months)
21.13	16.6

**RNAseq ID:**	PATE1 -
**Survival:**	OS
**Auto select best cutoff:**	checked
**Follow up threshold:**	all
**Censore at threshold:**	checked
**Compute median over entire database:**	false
**Cutoff value used in analysis:**	0
**Expression range of the probe:**	0 - 1
**Invert HR values below 1:**	not checked

Restrictions

Tumor type: Pancreatic ductal adenocarcinoma

Restrict analysis to subtypes...

Stage:	all
Gender:	all
Race:	all
Grade:	all
Mutation burden:	all

Restrict analysis based on cellular content...

Basophils:	all

B-cells:	all
CD4+ memory T-cells:	all
CD8+ T-cells:	all
Eosinophils:	all
Macrophages:	enriched
Mesenchymal stem cells:	all
Natural killer T-cells:	all
Regulatory T-cells:	all
Type 1 T-helper cells:	all
Type 2 T-helper cells:	all

Results

P value: 0.131
FDR: 100%

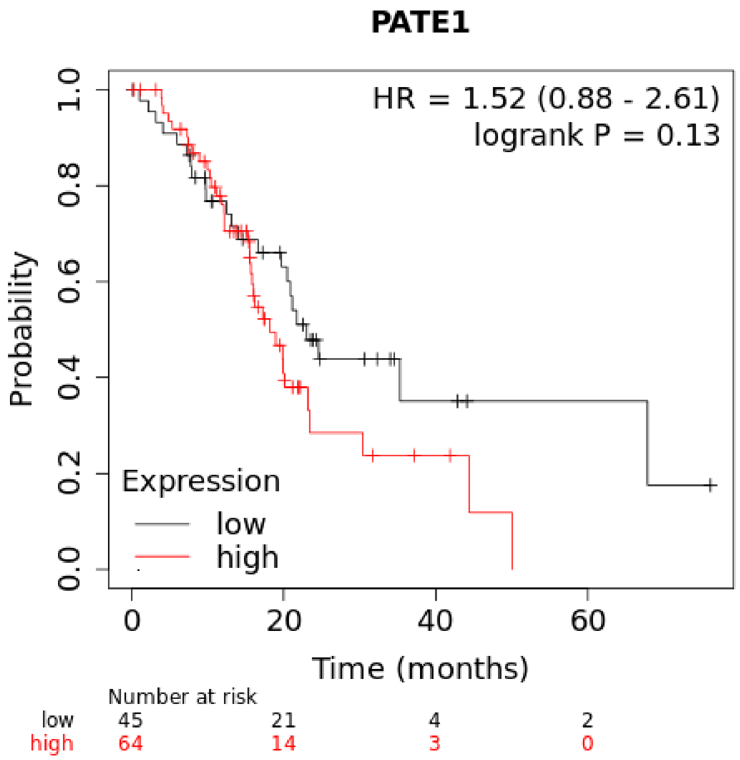

Click here to download the plot in TIFF format

Download plot as a PDF

Download p values vs. cutoff table

Median survival

Low expression cohort (months)	High expression cohort (months)
23.03	18.17

**RNAseq ID:**	PATE2 -
**Survival:**	OS
**Auto select best cutoff:**	checked
**Follow up threshold:**	all
**Censore at threshold:**	checked
**Compute median over entire database:**	false
**Cutoff value used in analysis:**	1
**Expression range of the probe:**	0 - 7
**Invert HR values below 1:**	not checked

Restrictions

Tumor type: Pancreatic ductal adenocarcinoma

Restrict analysis to subtypes...

Stage:	all
Gender:	all
Race:	all
Grade:	all
Mutation burden:	all

Restrict analysis based on cellular content...

Basophils:	all
B-cells:	all
CD4+ memory T-cells:	all
CD8+ T-cells:	all
Eosinophils:	all
Macrophages:	enriched
Mesenchymal stem cells:	all
Natural killer T-cells:	all
Regulatory T-cells:	all
Type 1 T-helper cells:	all
Type 2 T-helper cells:	all

Results

P value: 0.0097
FDR: over 50%

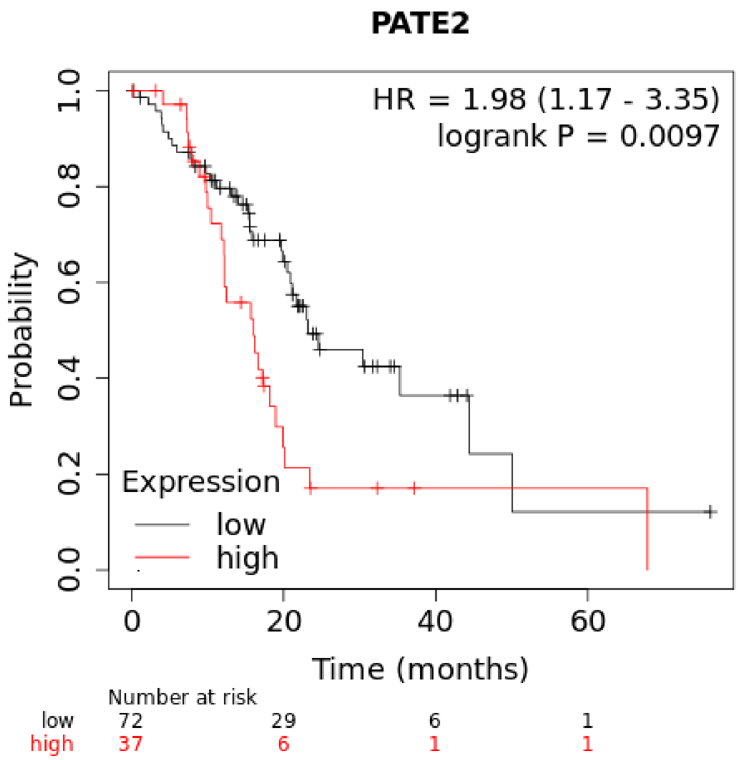

Click here to download the plot in TIFF format

Download plot as a PDF

Download p values vs. cutoff table

Median survival

Low expression cohort (months)	High expression cohort (months)
23.17	16.03

**RNAseq ID:**	PATE3 -
**Survival:**	OS
**Auto select best cutoff:**	checked
**Follow up threshold:**	all
**Censore at threshold:**	checked
**Compute median over entire database:**	false
**Cutoff value used in analysis:**	0
**Expression range of the probe:**	0 - 1
**Invert HR values below 1:**	not checked

Restrictions

Tumor type: Pancreatic ductal adenocarcinoma

Restrict analysis to subtypes...

Stage:	all
Gender:	all
Race:	all
Grade:	all
Mutation burden:	all

Restrict analysis based on cellular content...

Basophils:	all
B-cells:	all
CD4+ memory T-cells:	all
CD8+ T-cells:	all
Eosinophils:	all
Macrophages:	enriched
Mesenchymal stem cells:	all
Natural killer T-cells:	all
Regulatory T-cells:	all
Type 1 T-helper cells:	all
Type 2 T-helper cells:	all

Results

**P value:**	0.2861
**FDR:**	100%

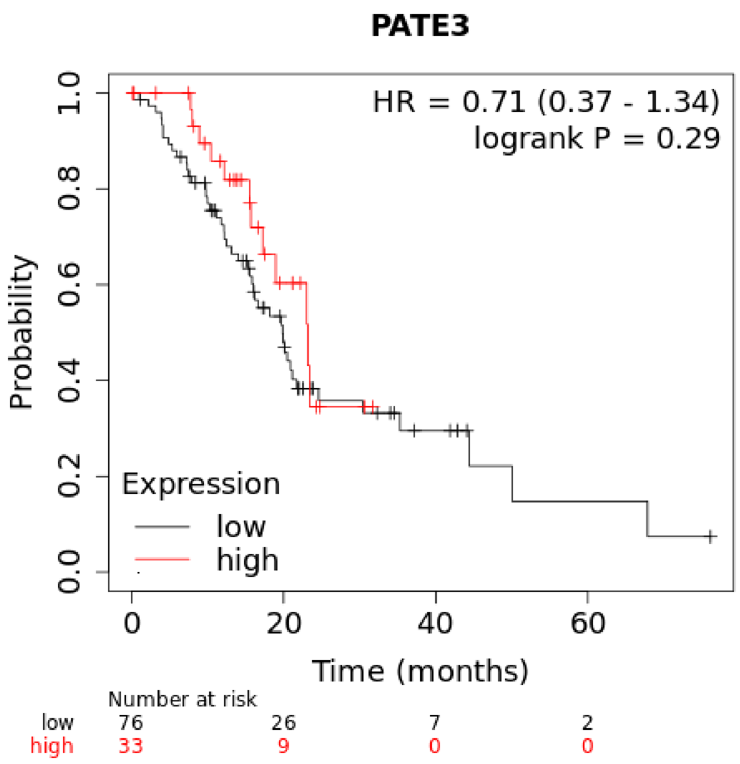

Click here to download the plot in TIFF format

Download plot as a PDF

Download p values vs. cutoff table

Median survival

Low expression cohort (months)	High expression cohort (months)
19.87	23.17

**RNAseq ID:**	PATE4 -
**Survival:**	OS
**Auto select best cutoff:**	checked
**Follow up threshold:**	all
**Censore at threshold:**	checked
**Compute median over entire database:**	false
**Cutoff value used in analysis:**	0
**Expression range of the probe:**	0 - 3
**Invert HR values below 1:**	not checked

Restrictions

Tumor type: Pancreatic ductal adenocarcinoma

Restrict analysis to subtypes...

Stage:	all
Gender:	all
Race:	all
Grade:	all
Mutation burden:	all

Restrict analysis based on cellular content...

Basophils: all

B-cells:	all
CD4+ memory T-cells:	all
CD8+ T-cells:	all
Eosinophils:	all
Macrophages:	enriched
Mesenchymal stem cells:	all
Natural killer T-cells:	all
Regulatory T-cells:	all
Type 1 T-helper cells:	all
Type 2 T-helper cells:	all

Results

P value: 0.4319
FDR: 100%

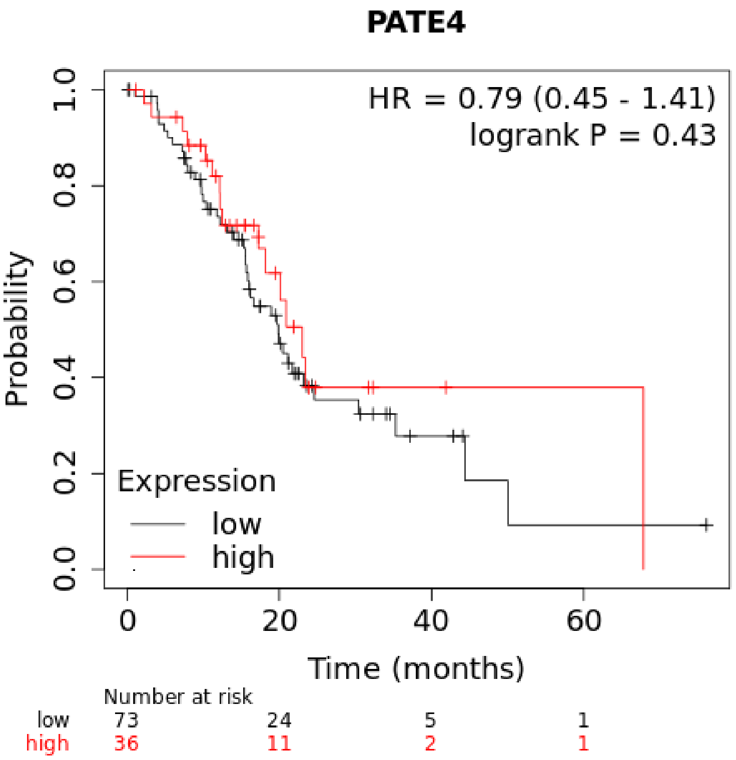

Click here to download the plot in TIFF format

Download plot as a PDF

Download p values vs. cutoff table

Median survival

Low expression cohort (months)	High expression cohort (months)
19.87	23.03

**RNAseq ID:**	CD59 -
**Survival:**	OS
**Auto select best cutoff:**	checked
**Follow up threshold:**	all
**Censore at threshold:**	checked
**Compute median over entire database:**	false
**Cutoff value used in analysis:**	17537
**Expression range of the probe:**	5502 - 33435
**Invert HR values below 1:**	not checked

Restrictions

Tumor type: Pancreatic ductal adenocarcinoma

Restrict analysis to subtypes...

Stage:	all
Gender:	all
Race:	all
Grade:	all
Mutation burden:	all

Restrict analysis based on cellular content...

Basophils:	all
B-cells:	all
CD4+ memory T-cells:	all
CD8+ T-cells:	all
Eosinophils:	all
Macrophages:	enriched
Mesenchymal stem cells:	all
Natural killer T-cells:	all
Regulatory T-cells:	all
Type 1 T-helper cells:	all
Type 2 T-helper cells:	all

Results

P value: 0.0089
FDR: over 50%

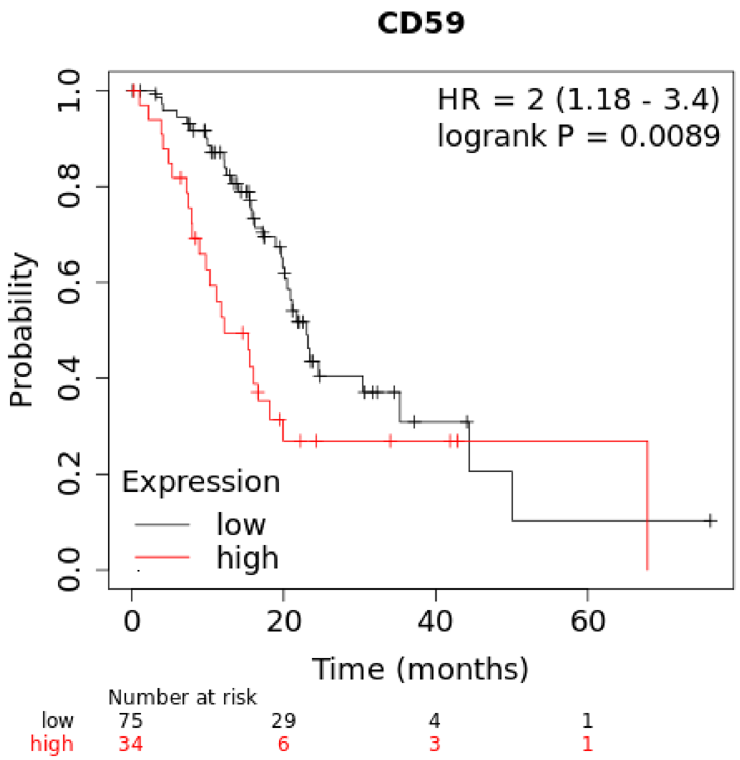

Click here to download the plot in TIFF format

Download plot as a PDF

Download p values vs. cutoff table

Median survival

Low expression cohort (months)	High expression cohort (months)
23.03	12.2

**RNAseq ID:**	LY6G6C -
**Survival:**	OS
**Auto select best cutoff:**	checked
**Follow up threshold:**	all
**Censore at threshold:**	checked
**Compute median over entire database:**	false
**Cutoff value used in analysis:**	3
**Expression range of the probe:**	0 - 85
**Invert HR values below 1:**	not checked

Restrictions

Tumor type: Pancreatic ductal adenocarcinoma

Restrict analysis to subtypes...

Stage:	all
Gender:	all
Race:	all
Grade:	all
Mutation burden:	all

Restrict analysis based on cellular content...

Basophils:	all
B-cells:	all
CD4+ memory T-cells:	all
CD8+ T-cells:	all
Eosinophils:	all
Macrophages:	enriched
Mesenchymal stem cells:	all
Natural killer T-cells:	all
Regulatory T-cells:	all
Type 1 T-helper cells:	all
Type 2 T-helper cells:	all

Results

**P value:**	0.4771
**FDR:**	100%

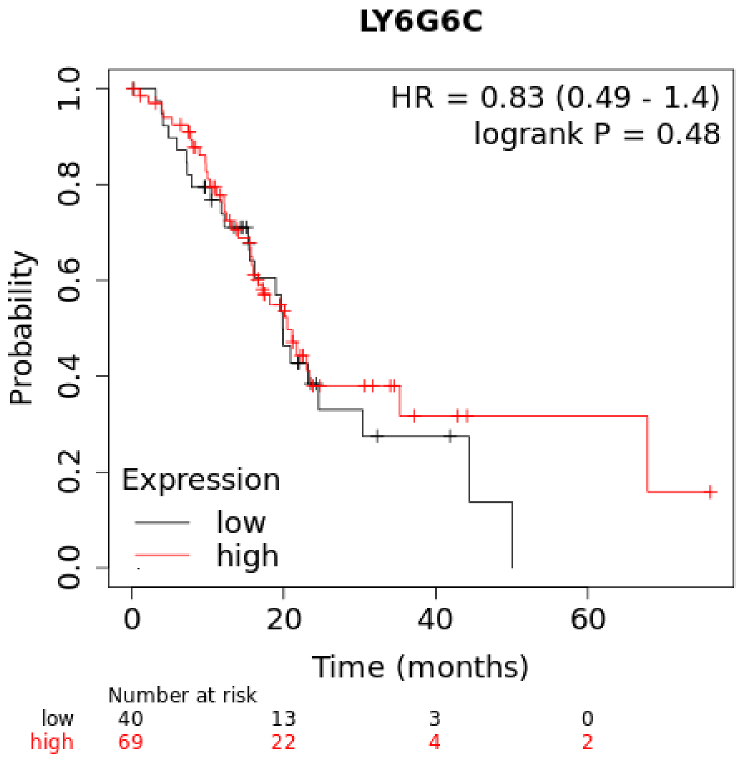

Click here to download the plot in TIFF format

Download plot as a PDF

Download p values vs. cutoff table

Median survival

Low expression cohort (months)	High expression cohort (months)
19.87	20.47

**RNAseq ID:**	LY6G6D -
**Survival:**	OS
**Auto select best cutoff:**	checked
**Follow up threshold:**	all
**Censore at threshold:**	checked
**Compute median over entire database:**	false
**Cutoff value used in analysis:**	0
**Expression range of the probe:**	0 - 1
**Invert HR values below 1:**	not checked

Restrictions

Tumor type: Pancreatic ductal adenocarcinoma

Restrict analysis to subtypes...

Stage:	all
Gender:	all
Race:	all
Grade:	all
Mutation burden:	all

Restrict analysis based on cellular content...

Basophils:	all

B-cells:	all
CD4+ memory T-cells:	all
CD8+ T-cells:	all
Eosinophils:	all
Macrophages:	enriched
Mesenchymal stem cells:	all
Natural killer T-cells:	all
Regulatory T-cells:	all
Type 1 T-helper cells:	all
Type 2 T-helper cells:	all

Results

P value: 0.1175
FDR: 100%

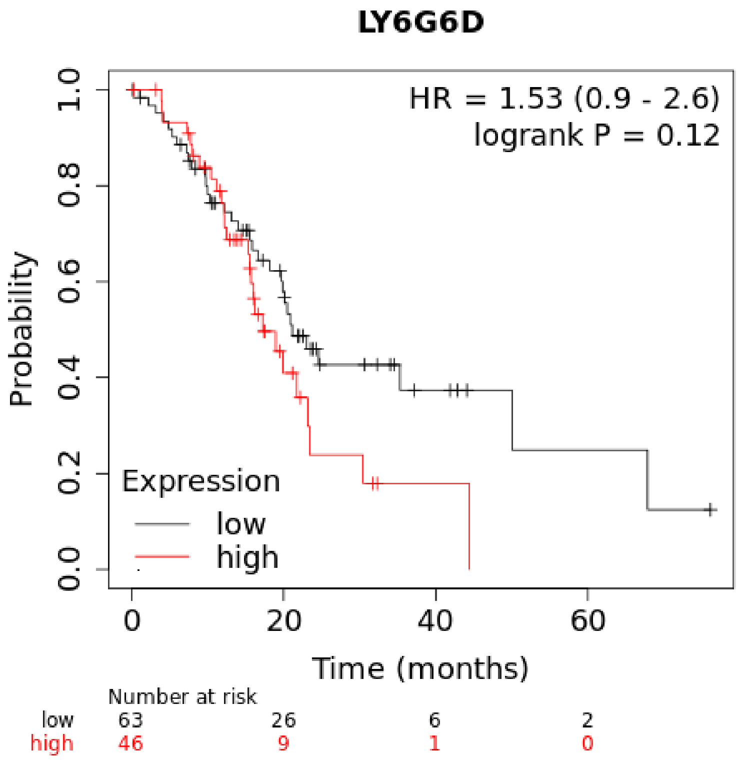

Click here to download the plot in TIFF format

Download plot as a PDF

Download p values vs. cutoff table

Median survival

Low expression cohort (months)	High expression cohort (months)
21.13	17.23

**RNAseq ID:**	LY6G6F -
**Survival:**	OS
**Auto select best cutoff:**	checked
**Follow up threshold:**	all
**Censore at threshold:**	checked
**Compute median over entire database:**	false
**Cutoff value used in analysis:**	0
**Expression range of the probe:**	0 - 2
**Invert HR values below 1:**	not checked

Restrictions

Tumor type: Pancreatic ductal adenocarcinoma

Restrict analysis to subtypes...

Stage: all
Gender: all
Race: all
Grade: all
Mutation burden: all

Restrict analysis based on cellular content...

Basophils: all
B-cells: all
CD4+ memory T-cells: all
CD8+ T-cells: all
Eosinophils: all
Macrophages: enriched
Mesenchymal stem cells: all
Natural killer T-cells: all
Regulatory T-cells: all
Type 1 T-helper cells: all
Type 2 T-helper cells: all

Results

P value: 0.1772
FDR: 100%

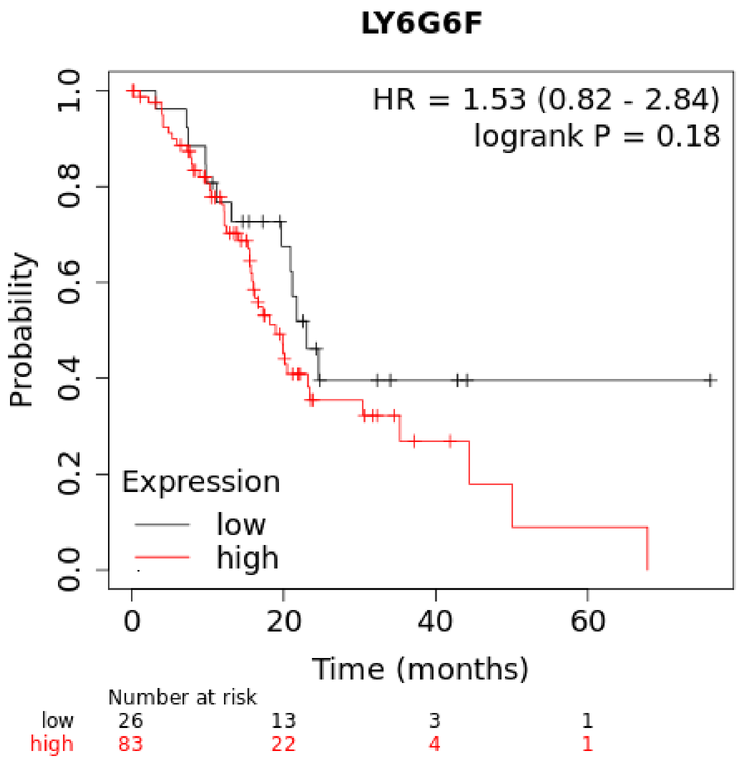

Click here to download the plot in TIFF format

Download plot as a PDF

Download p values vs. cutoff table

Median survival

Low expression cohort (months)	High expression cohort (months)
23.03	18.93

**RNAseq ID:**	LY6G5C -
**Survival:**	OS
**Auto select best cutoff:**	checked
**Follow up threshold:**	all
**Censore at threshold:**	checked
**Compute median over entire database:**	false
**Cutoff value used in analysis:**	38
**Expression range of the probe:**	17 - 116
**Invert HR values below 1:**	not checked

Restrictions

Tumor type: Pancreatic ductal adenocarcinoma

Restrict analysis to subtypes...

Stage:	all
Gender:	all
Race:	all
Grade:	all
Mutation burden:	all

Restrict analysis based on cellular content...

Basophils:	all
B-cells:	all
CD4+ memory T-cells:	all
CD8+ T-cells:	all
Eosinophils:	all
Macrophages:	enriched
Mesenchymal stem cells:	all
Natural killer T-cells:	all
Regulatory T-cells:	all
Type 1 T-helper cells:	all
Type 2 T-helper cells:	all

Results

**P value:**	0.0265
**FDR:**	over 50%

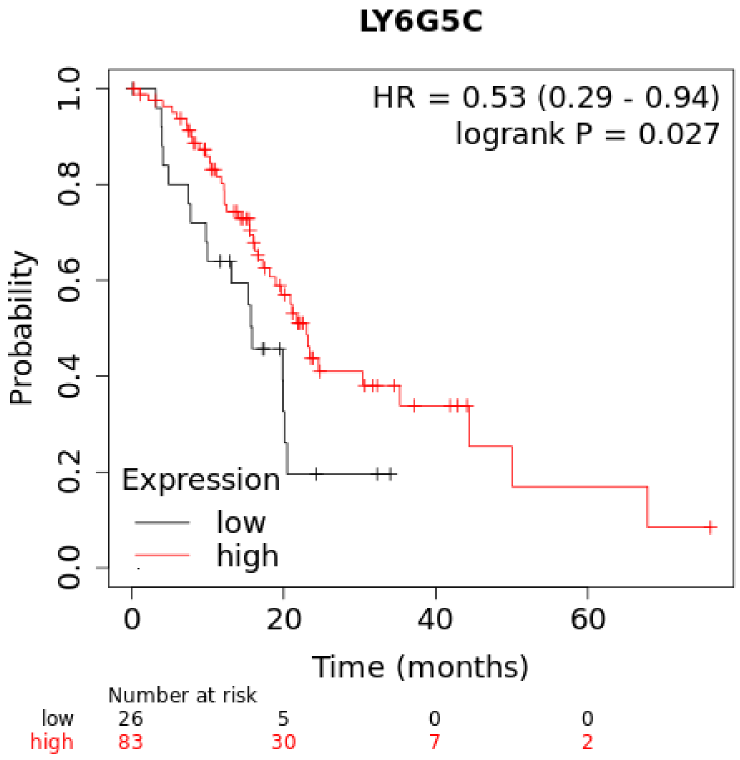

Click here to download the plot in TIFF format

Download plot as a PDF

Download p values vs. cutoff table

Median survival

Low expression cohort (months)	High expression cohort (months)
15.87	23.03

**RNAseq ID:**	LY6G5B -
**Survival:**	OS
**Auto select best cutoff:**	checked
**Follow up threshold:**	all
**Censore at threshold:**	checked
**Compute median over entire database:**	false
**Cutoff value used in analysis:**	56
**Expression range of the probe:**	4 - 173
**Invert HR values below 1:**	not checked

Restrictions

Tumor type: Pancreatic ductal adenocarcinoma

Restrict analysis to subtypes...

Stage:	all
Gender:	all
Race:	all
Grade:	all
Mutation burden:	all

Restrict analysis based on cellular content...

Basophils:	all

B-cells:	all
CD4+ memory T-cells:	all
CD8+ T-cells:	all
Eosinophils:	all
Macrophages:	enriched
Mesenchymal stem cells:	all
Natural killer T-cells:	all
Regulatory T-cells:	all
Type 1 T-helper cells:	all
Type 2 T-helper cells:	all

Results

P value: 0.0009
FDR: 10%

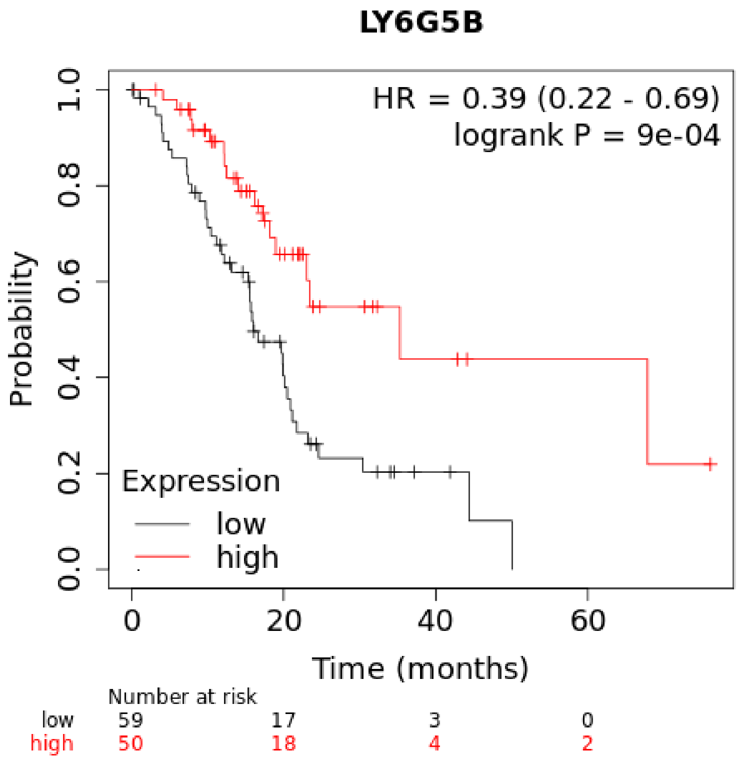

Click here to download the plot in TIFF format

Download plot as a PDF

Download p values vs. cutoff table

Median survival

Low expression cohort (months)	High expression cohort (months)
16.03	35.3

You can save the plots by right-clicking the image and then selecting "Save image as...". To generate a high resolution TIFF image, please adjust the "Settings" in the analysis page.

KMplot.com Copyright © 2009-2020 | Contact | Sitemap

Pan-cancer

KM plotter | Home | Vote | Download | Updates | Contact

The desired RNAseq ID is valid: PSCA (-), LY6K (-), SLURP1 (-), LYPD2 (-), LY6D (-), GML (-), LY6E (-), LY6L (-), LY6H (-), GPIHBP1 (-), LYPD4 (-), CD177 (-), TEX101 (-), LYPD3 (-), PINLYP (-), PLAUR (-), LYPD5 (-), SPACA4 (-), ACRV1 (-), PATE1 (-), PATE2 (-), PATE3 (-), PATE4 (-), CD59 (-), LY6G6C (-), LY6G6D (-), LY6G6F (-), LY6G5C (-), LY6G5B (-),

**RNAseq ID:**	PSCA -
**Survival:**	OS
**Auto select best cutoff:**	checked
**Follow up threshold:**	all
**Censore at threshold:**	checked
**Compute median over entire database:**	false
**Cutoff value used in analysis:**	2404
**Expression range of the probe:**	0 - 65661
**Invert HR values below 1:**	not checked

Restrictions

Tumor type: Pancreatic ductal adenocarcinoma

Restrict analysis to subtypes...

Stage:	all
Gender:	all
Race:	all
Grade:	all
Mutation burden:	all

Restrict analysis based on cellular content...

Basophils:	all
B-cells:	all
CD4+ memory T-cells:	all
CD8+ T-cells:	all
Eosinophils:	all
Macrophages:	decreased
Mesenchymal stem cells:	all
Natural killer T-cells:	all
Regulatory T-cells:	all
Type 1 T-helper cells:	all
Type 2 T-helper cells:	all

Results

P value: 1.9e-6
FDR: 1%

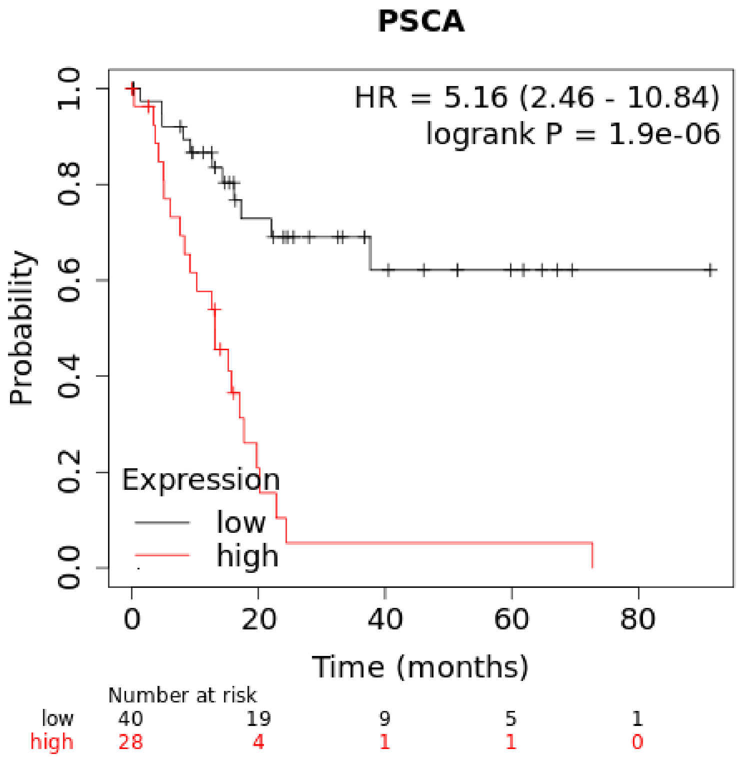

[Click here to download the plot in TIFF format]

[Download plot as a PDF]

[Download p values vs. cutoff table]

Upper quartile survival

Low expression cohort (months)	High expression cohort (months)
17.27	6.1

**RNAseq ID:**	LY6K -
**Survival:**	OS
**Auto select best cutoff:**	checked
**Follow up threshold:**	all
**Censore at threshold:**	checked
**Compute median over entire database:**	false
**Cutoff value used in analysis:**	6
**Expression range of the probe:**	0 - 1332
**Invert HR values below 1:**	not checked

Restrictions

Tumor type: Pancreatic ductal adenocarcinoma

Restrict analysis to subtypes...

Stage:	all
Gender:	all
Race:	all
Grade:	all
Mutation burden:	all

Restrict analysis based on cellular content...

Basophils:	all

B-cells:	all
CD4+ memory T-cells:	all
CD8+ T-cells:	all
Eosinophils:	all
Macrophages:	decreased
Mesenchymal stem cells:	all
Natural killer T-cells:	all
Regulatory T-cells:	all
Type 1 T-helper cells:	all
Type 2 T-helper cells:	all

Results

P value: 0.0047
FDR: over 50%

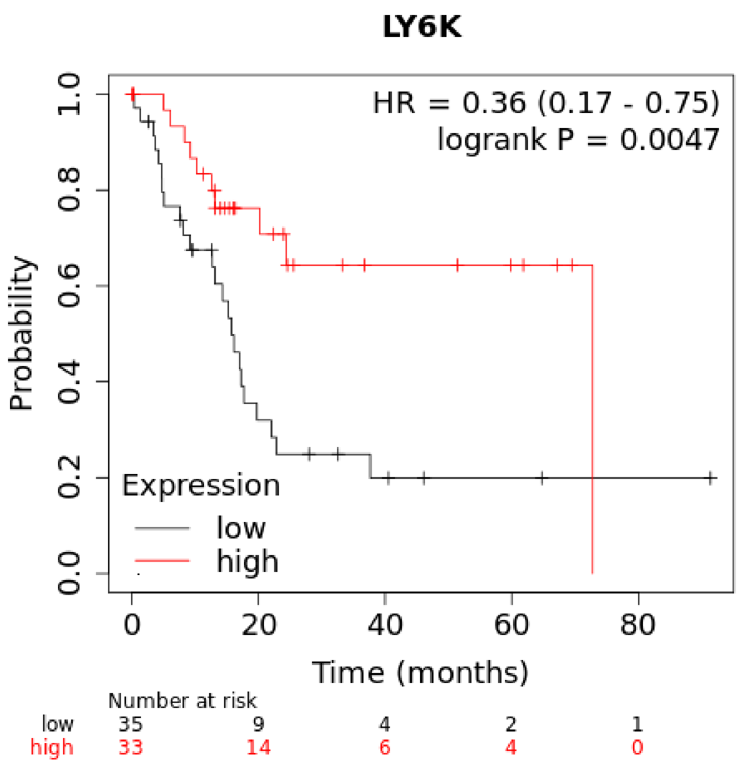

Click here to download the plot in TIFF format

Download plot as a PDF

Download p values vs. cutoff table

Median survival

Low expression cohort (months)	High expression cohort (months)
15.77	72.73

**RNAseq ID:**	SLURP1 -
**Survival:**	OS
**Auto select best cutoff:**	checked
**Follow up threshold:**	all
**Censore at threshold:**	checked
**Compute median over entire database:**	false
**Cutoff value used in analysis:**	1
**Expression range of the probe:**	0 - 279
**Invert HR values below 1:**	not checked

Restrictions

Tumor type: Pancreatic ductal adenocarcinoma

Restrict analysis to subtypes...

Stage:	all
Gender:	all
Race:	all
Grade:	all
Mutation burden:	all

Restrict analysis based on cellular content...

Basophils:	all
B-cells:	all
CD4+ memory T-cells:	all
CD8+ T-cells:	all
Eosinophils:	all
Macrophages:	decreased
Mesenchymal stem cells:	all
Natural killer T-cells:	all
Regulatory T-cells:	all
Type 1 T-helper cells:	all
Type 2 T-helper cells:	all

Results

P value: 9.9e-7
FDR: 1%

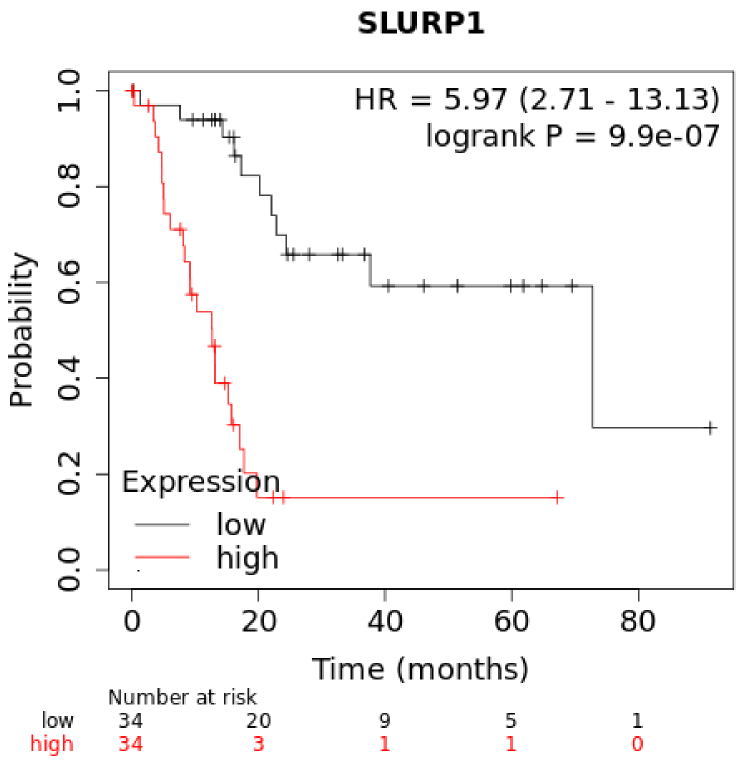

Click here to download the plot in TIFF format

Download plot as a PDF

Download p values vs. cutoff table

Median survival

Low expression cohort (months)	High expression cohort (months)
72.73	12.7

**RNAseq ID:**	LYPD2 -
**Survival:**	OS
**Auto select best cutoff:**	checked
**Follow up threshold:**	all
**Censore at threshold:**	checked
**Compute median over entire database:**	false
**Cutoff value used in analysis:**	2
**Expression range of the probe:**	0 - 2654
**Invert HR values below 1:**	not checked

Restrictions

Tumor type: Pancreatic ductal adenocarcinoma

Restrict analysis to subtypes...

Stage:	all
Gender:	all
Race:	all
Grade:	all
Mutation burden:	all

Restrict analysis based on cellular content...

Basophils:	all
B-cells:	all
CD4+ memory T-cells:	all
CD8+ T-cells:	all
Eosinophils:	all
Macrophages:	decreased
Mesenchymal stem cells:	all
Natural killer T-cells:	all
Regulatory T-cells:	all
Type 1 T-helper cells:	all
Type 2 T-helper cells:	all

Results

**P value:**	6.0e-5
**FDR:**	1%

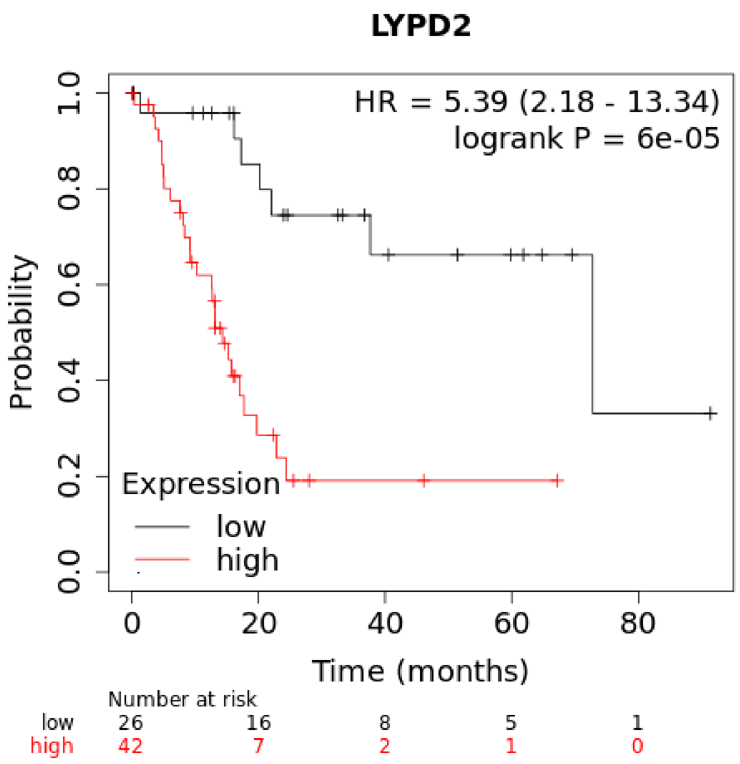

Click here to download the plot in TIFF format

Download plot as a PDF

Download p values vs. cutoff table

Median survival

Low expression cohort (months)	High expression cohort (months)
72.73	14.33

RNAseq ID: LY6D -
Survival: OS
Auto select best cutoff: checked
Follow up threshold: all
Censore at threshold: checked
Compute median over entire database: false
Cutoff value used in analysis: 9
Expression range of the probe: 0 - 18030
Invert HR values below 1: not checked

Restrictions

Tumor type: Pancreatic ductal adenocarcinoma

Restrict analysis to subtypes...

Stage: all
Gender: all
Race: all
Grade: all
Mutation burden: all

Restrict analysis based on cellular content...

Basophils: all

B-cells:	all
CD4+ memory T-cells:	all
CD8+ T-cells:	all
Eosinophils:	all
Macrophages:	decreased
Mesenchymal stem cells:	all
Natural killer T-cells:	all
Regulatory T-cells:	all
Type 1 T-helper cells:	all
Type 2 T-helper cells:	all

Results

P value: 8.3e-6
FDR: 1%

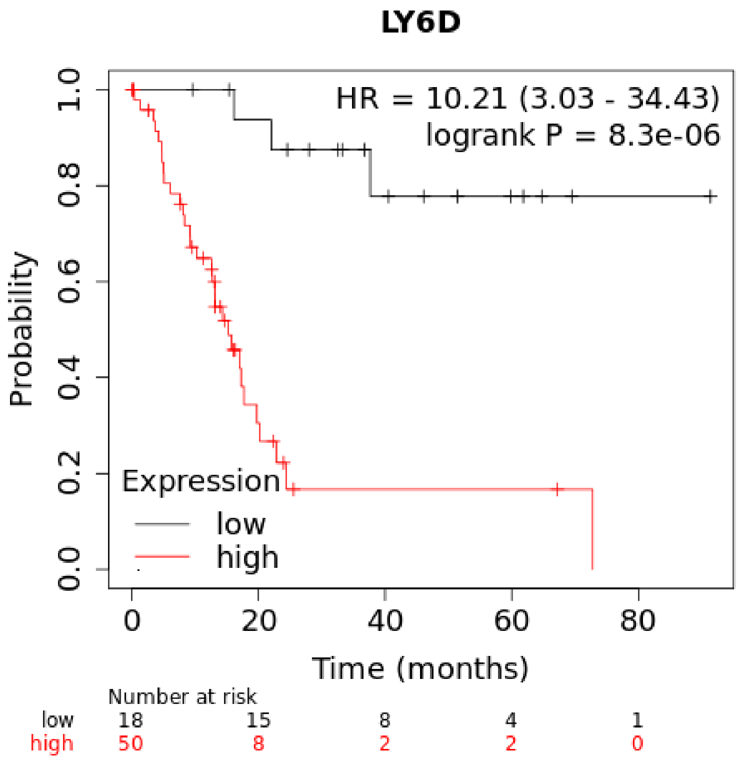

Click here to download the plot in TIFF format

Download plot as a PDF

Download p values vs. cutoff table

Median survival

Low expression cohort (months)	High expression cohort (months)
NA	NA

**RNAseq ID:**	GML -
**Survival:**	OS
**Auto select best cutoff:**	checked
**Follow up threshold:**	all
**Censore at threshold:**	checked
**Compute median over entire database:**	false
**Cutoff value used in analysis:**	0
**Expression range of the probe:**	0 - 2
**Invert HR values below 1:**	not checked

Restrictions

Tumor type: Pancreatic ductal adenocarcinoma

Restrict analysis to subtypes...

Stage:	all
Gender:	all
Race:	all
Grade:	all
Mutation burden:	all

Restrict analysis based on cellular content...

Basophils:	all
B-cells:	all
CD4+ memory T-cells:	all
CD8+ T-cells:	all
Eosinophils:	all
Macrophages:	decreased
Mesenchymal stem cells:	all
Natural killer T-cells:	all
Regulatory T-cells:	all
Type 1 T-helper cells:	all
Type 2 T-helper cells:	all

Results

P value: 0.0276
FDR: over 50%

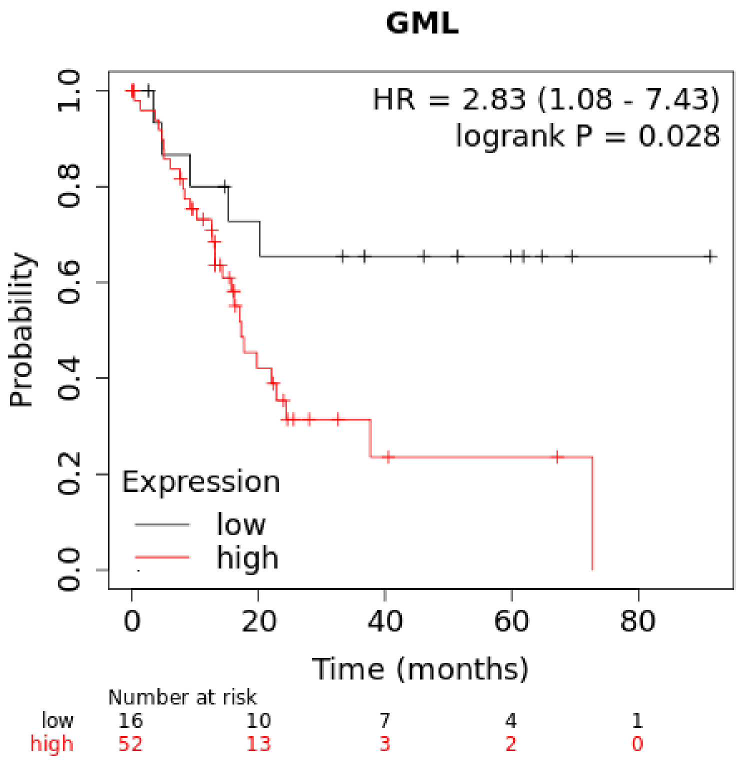

Click here to download the plot in TIFF format

Download plot as a PDF

Download p values vs. cutoff table

Upper quartile survival

Low expression cohort (months)	High expression cohort (months)
15.27	10.27

**RNAseq ID:**	LY6E -
**Survival:**	OS
**Auto select best cutoff:**	checked
**Follow up threshold:**	all
**Censore at threshold:**	checked
**Compute median over entire database:**	false
**Cutoff value used in analysis:**	10634
**Expression range of the probe:**	254 - 56404
**Invert HR values below 1:**	not checked

Restrictions

Tumor type: Pancreatic ductal adenocarcinoma

Restrict analysis to subtypes...

Stage:	all
Gender:	all
Race:	all
Grade:	all
Mutation burden:	all

Restrict analysis based on cellular content...

Basophils:	all
B-cells:	all
CD4+ memory T-cells:	all
CD8+ T-cells:	all
Eosinophils:	all
Macrophages:	decreased
Mesenchymal stem cells:	all
Natural killer T-cells:	all
Regulatory T-cells:	all
Type 1 T-helper cells:	all
Type 2 T-helper cells:	all

Results

**P value:**	0.0001
**FDR:**	1%

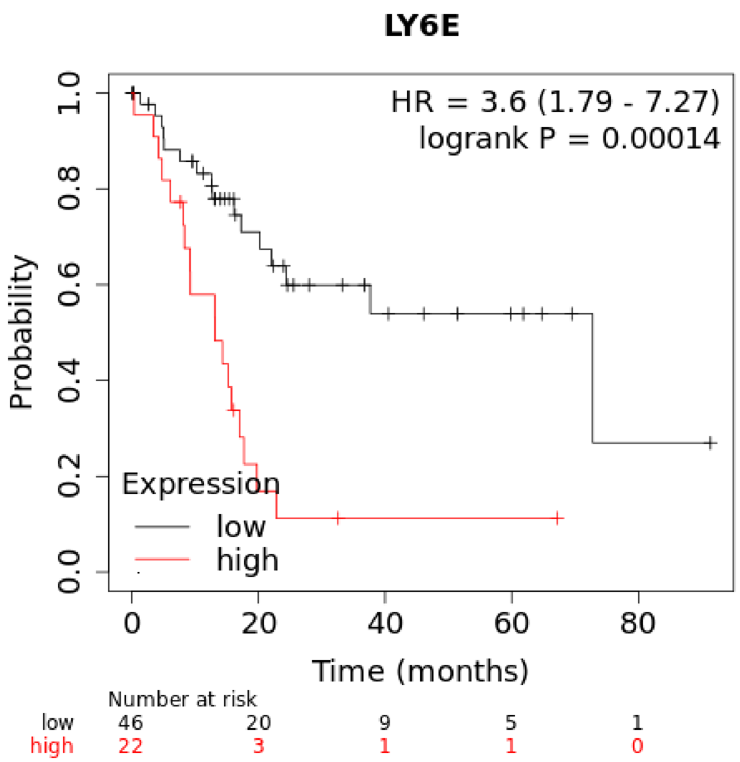

Click here to download the plot in TIFF format

Download plot as a PDF

Download p values vs. cutoff table

Median survival

Low expression cohort (months)	High expression cohort (months)
72.73	13.13

**RNAseq ID:**	LY6L -
**Survival:**	OS
**Auto select best cutoff:**	checked
**Follow up threshold:**	all
**Censore at threshold:**	checked
**Compute median over entire database:**	false
**Cutoff value used in analysis:**	0
**Expression range of the probe:**	0 - 8
**Invert HR values below 1:**	not checked

Restrictions

Tumor type: Pancreatic ductal adenocarcinoma

Restrict analysis to subtypes...

Stage:	all
Gender:	all
Race:	all
Grade:	all
Mutation burden:	all

Restrict analysis based on cellular content...

Basophils:	all

B-cells:	all
CD4+ memory T-cells:	all
CD8+ T-cells:	all
Eosinophils:	all
Macrophages:	decreased
Mesenchymal stem cells:	all
Natural killer T-cells:	all
Regulatory T-cells:	all
Type 1 T-helper cells:	all
Type 2 T-helper cells:	all

Results

P value: 0.0033
FDR: 20%

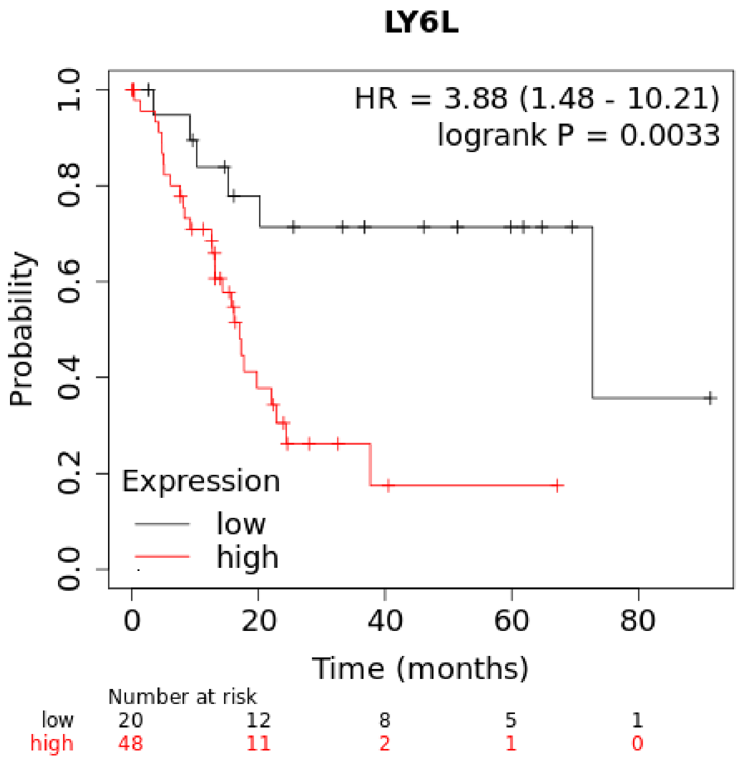

Click here to download the plot in TIFF format

Download plot as a PDF

Download p values vs. cutoff table

Median survival

Low expression cohort (months)	High expression cohort (months)
72.73	17.03

**RNAseq ID:**	LY6H -
**Survival:**	OS
**Auto select best cutoff:**	checked
**Follow up threshold:**	all
**Censore at threshold:**	checked
**Compute median over entire database:**	false
**Cutoff value used in analysis:**	60
**Expression range of the probe:**	1 - 9495
**Invert HR values below 1:**	not checked

Restrictions

Tumor type: Pancreatic ductal adenocarcinoma

Restrict analysis to subtypes...

Stage:	all
Gender:	all
Race:	all
Grade:	all
Mutation burden:	all

Restrict analysis based on cellular content...

Basophils:	all
B-cells:	all
CD4+ memory T-cells:	all
CD8+ T-cells:	all
Eosinophils:	all
Macrophages:	decreased
Mesenchymal stem cells:	all
Natural killer T-cells:	all
Regulatory T-cells:	all
Type 1 T-helper cells:	all
Type 2 T-helper cells:	all

Results

P value: 0.0007
FDR: 10%

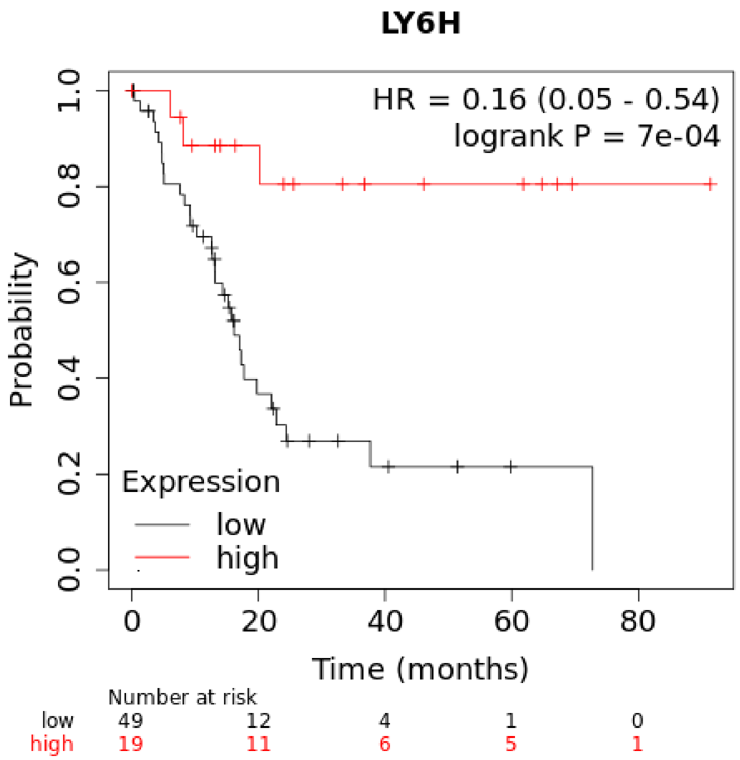

[Click here to download the plot in TIFF format](#)

[Download plot as a PDF](#)

[Download p values vs. cutoff table](#)

Median survival

Low expression cohort (months)	High expression cohort (months)
NA	NA

**RNAseq ID:**	GPIHBP1 -
**Survival:**	OS
**Auto select best cutoff:**	checked
**Follow up threshold:**	all
**Censore at threshold:**	checked
**Compute median over entire database:**	false
**Cutoff value used in analysis:**	27
**Expression range of the probe:**	7 - 344
**Invert HR values below 1:**	not checked

Restrictions

Tumor type: Pancreatic ductal adenocarcinoma

Restrict analysis to subtypes...

Stage:	all
Gender:	all
Race:	all
Grade:	all
Mutation burden:	all

Restrict analysis based on cellular content...

Basophils:	all
B-cells:	all
CD4+ memory T-cells:	all
CD8+ T-cells:	all
Eosinophils:	all
Macrophages:	decreased
Mesenchymal stem cells:	all
Natural killer T-cells:	all
Regulatory T-cells:	all
Type 1 T-helper cells:	all
Type 2 T-helper cells:	all

Results

**P value:**	0.0795
**FDR:**	100%

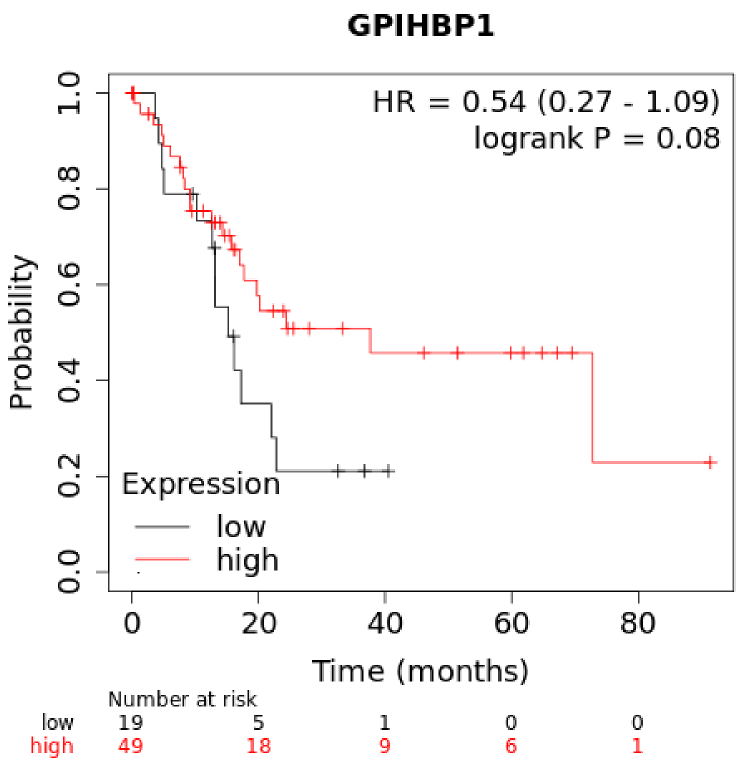

Click here to download the plot in TIFF format

Download plot as a PDF

Download p values vs. cutoff table

Median survival

Low expression cohort (months)	High expression cohort (months)
15.27	37.67

**RNAseq ID:**	LYPD4 -
**Survival:**	OS
**Auto select best cutoff:**	checked
**Follow up threshold:**	all
**Censore at threshold:**	checked
**Compute median over entire database:**	false
**Cutoff value used in analysis:**	0
**Expression range of the probe:**	0 - 18
**Invert HR values below 1:**	not checked

Restrictions

Tumor type: Pancreatic ductal adenocarcinoma

Restrict analysis to subtypes...

Stage:	all
Gender:	all
Race:	all
Grade:	all
Mutation burden:	all

Restrict analysis based on cellular content...

Basophils:	all

B-cells:	all
CD4+ memory T-cells:	all
CD8+ T-cells:	all
Eosinophils:	all
Macrophages:	decreased
Mesenchymal stem cells:	all
Natural killer T-cells:	all
Regulatory T-cells:	all
Type 1 T-helper cells:	all
Type 2 T-helper cells:	all

Results

P value: 0.0035
FDR: 50%

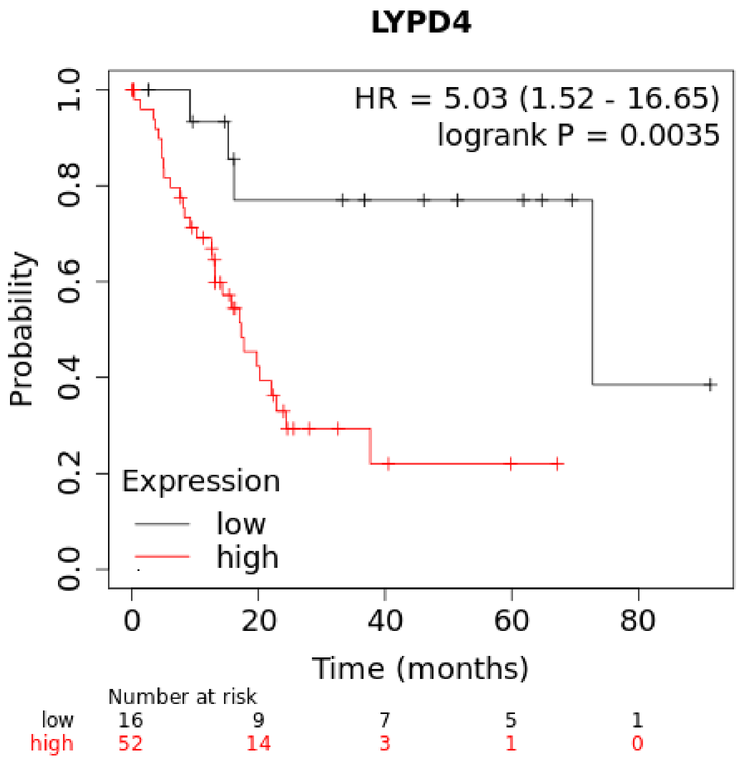

Click here to download the plot in TIFF format

Download plot as a PDF

Download p values vs. cutoff table

Median survival

Low expression cohort (months)	High expression cohort (months)
72.73	17.27

**RNAseq ID:**	CD177 -
**Survival:**	OS
**Auto select best cutoff:**	checked
**Follow up threshold:**	all
**Censore at threshold:**	checked
**Compute median over entire database:**	false
**Cutoff value used in analysis:**	9
**Expression range of the probe:**	0 - 1837
**Invert HR values below 1:**	not checked

Restrictions

Tumor type: Pancreatic ductal adenocarcinoma

Restrict analysis to subtypes...

Stage:	all
Gender:	all
Race:	all
Grade:	all
Mutation burden:	all

Restrict analysis based on cellular content...

Basophils:	all
B-cells:	all
CD4+ memory T-cells:	all
CD8+ T-cells:	all
Eosinophils:	all
Macrophages:	decreased
Mesenchymal stem cells:	all
Natural killer T-cells:	all
Regulatory T-cells:	all
Type 1 T-helper cells:	all
Type 2 T-helper cells:	all

Results

P value: 0.0079
FDR: 50%

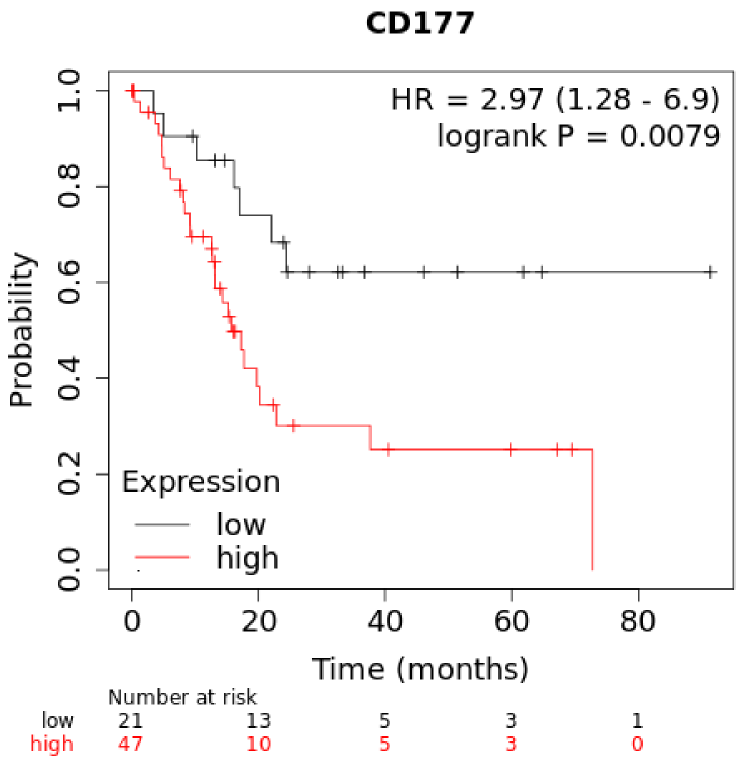

Click here to download the plot in TIFF format

Download plot as a PDF

Download p values vs. cutoff table

Upper quartile survival

Low expression cohort (months)	High expression cohort (months)
17.03	8.33

**RNAseq ID:**	TEX101 -
**Survival:**	OS
**Auto select best cutoff:**	checked
**Follow up threshold:**	all
**Censore at threshold:**	checked
**Compute median over entire database:**	false
**Cutoff value used in analysis:**	0
**Expression range of the probe:**	0 - 149
**Invert HR values below 1:**	not checked

Restrictions

Tumor type: Pancreatic ductal adenocarcinoma

Restrict analysis to subtypes...

Stage:	all
Gender:	all
Race:	all
Grade:	all
Mutation burden:	all

Restrict analysis based on cellular content...

Basophils:	all
B-cells:	all
CD4+ memory T-cells:	all
CD8+ T-cells:	all
Eosinophils:	all
Macrophages:	decreased
Mesenchymal stem cells:	all
Natural killer T-cells:	all
Regulatory T-cells:	all
Type 1 T-helper cells:	all
Type 2 T-helper cells:	all

Results

**P value:**	0.0099
**FDR:**	over 50%

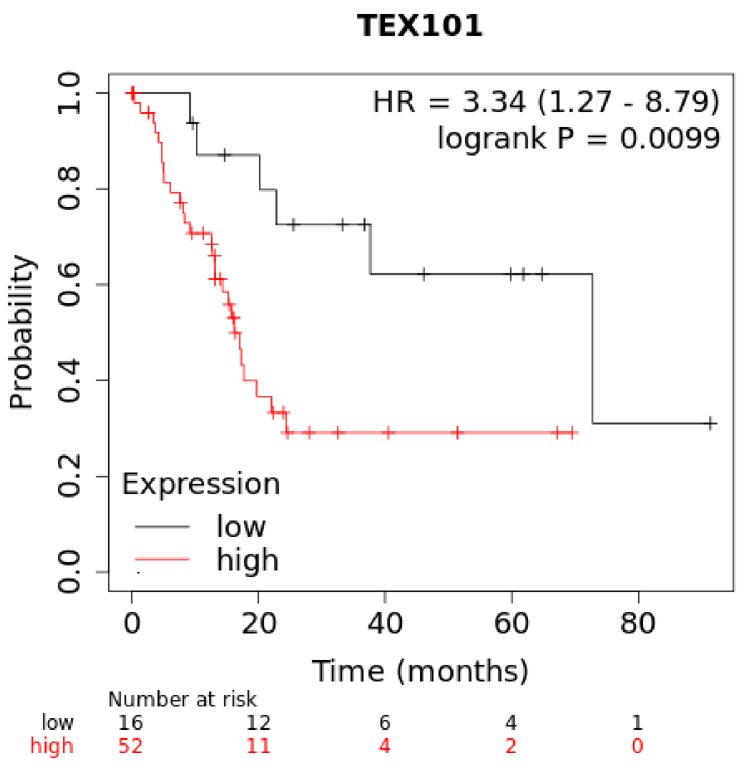

Click here to download the plot in TIFF format

Download plot as a PDF

Download p values vs. cutoff table

Median survival

Low expression cohort (months)	High expression cohort (months)
72.73	16.17

RNAseq ID: LYPD3 -
Survival: OS
Auto select best cutoff: checked
Follow up threshold: all
Censore at threshold: checked
Compute median over entire database: false
Cutoff value used in analysis: 148
Expression range of the probe: 9 - 3640
Invert HR values below 1: not checked

Restrictions

Tumor type: Pancreatic ductal adenocarcinoma

Restrict analysis to subtypes...

Stage: all
Gender: all
Race: all
Grade: all
Mutation burden: all

Restrict analysis based on cellular content...

Basophils: all

B-cells:	all
CD4+ memory T-cells:	all
CD8+ T-cells:	all
Eosinophils:	all
Macrophages:	decreased
Mesenchymal stem cells:	all
Natural killer T-cells:	all
Regulatory T-cells:	all
Type 1 T-helper cells:	all
Type 2 T-helper cells:	all

Results

P value: 0.0355
FDR: over 50%

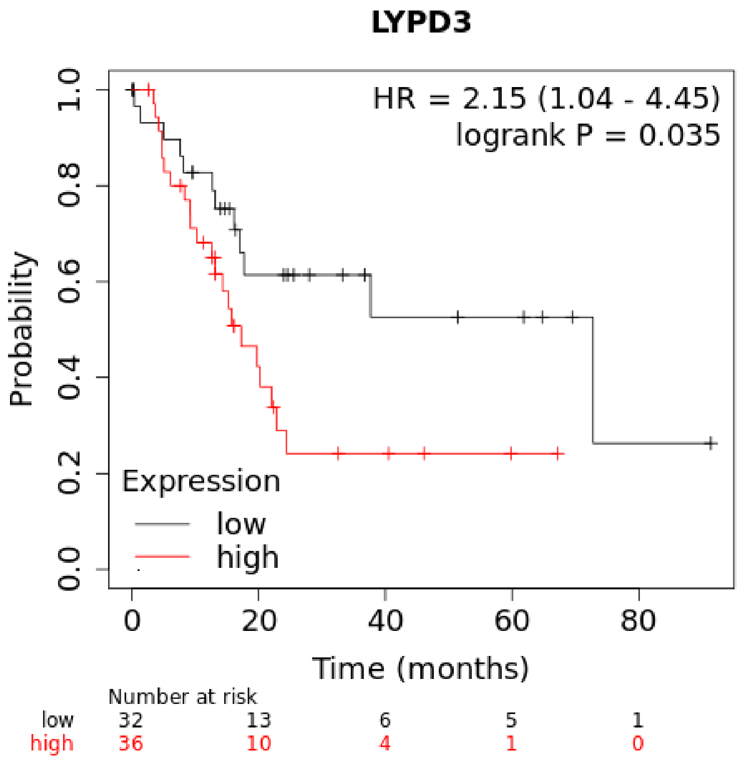

Click here to download the plot in TIFF format

Download plot as a PDF

Download p values vs. cutoff table

Median survival

Low expression cohort (months)	High expression cohort (months)
72.73	17.27

**RNAseq ID:**	PINLYP -
**Survival:**	OS
**Auto select best cutoff:**	checked
**Follow up threshold:**	all
**Censore at threshold:**	checked
**Compute median over entire database:**	false
**Cutoff value used in analysis:**	43
**Expression range of the probe:**	5 - 263
**Invert HR values below 1:**	not checked

Restrictions

Tumor type: Pancreatic ductal adenocarcinoma

Restrict analysis to subtypes...

Stage: all
Gender: all
Race: all
Grade: all
Mutation burden: all

Restrict analysis based on cellular content...

Basophils: all
B-cells: all
CD4+ memory T-cells: all
CD8+ T-cells: all
Eosinophils: all
Macrophages: decreased
Mesenchymal stem cells: all
Natural killer T-cells: all
Regulatory T-cells: all
Type 1 T-helper cells: all
Type 2 T-helper cells: all

Results

P value: 0.0949
FDR: 100%

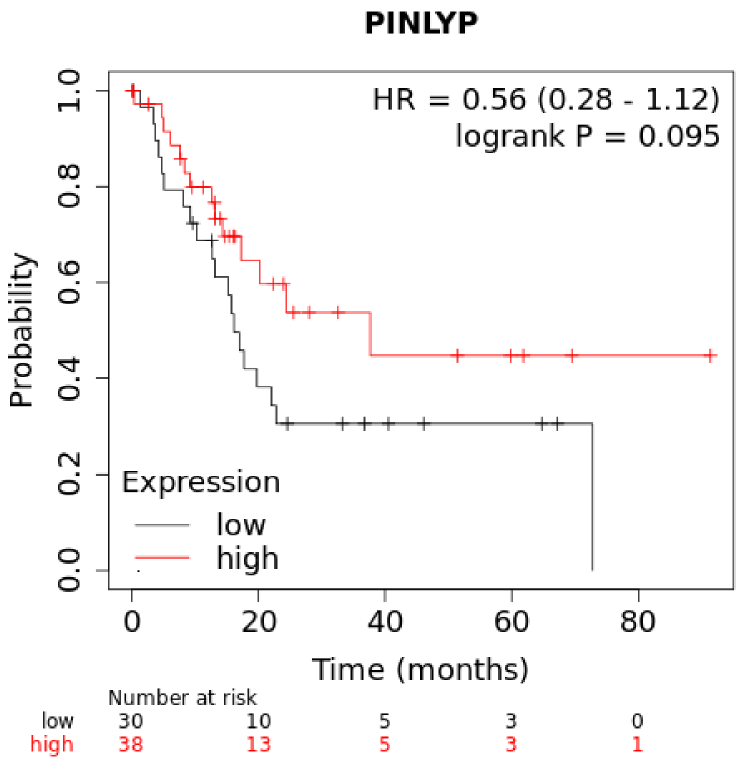

Click here to download the plot in TIFF format

Download plot as a PDF

Download p values vs. cutoff table

Median survival

Low expression cohort (months)	High expression cohort (months)
16.17	37.67

**RNAseq ID:**	PLAUR -
**Survival:**	OS
**Auto select best cutoff:**	checked
**Follow up threshold:**	all
**Censore at threshold:**	checked
**Compute median over entire database:**	false
**Cutoff value used in analysis:**	1592
**Expression range of the probe:**	47 - 7626
**Invert HR values below 1:**	not checked

Restrictions

Tumor type: Pancreatic ductal adenocarcinoma

Restrict analysis to subtypes...

Stage:	all
Gender:	all
Race:	all
Grade:	all
Mutation burden:	all

Restrict analysis based on cellular content...

Basophils:	all
B-cells:	all
CD4+ memory T-cells:	all
CD8+ T-cells:	all
Eosinophils:	all
Macrophages:	decreased
Mesenchymal stem cells:	all
Natural killer T-cells:	all
Regulatory T-cells:	all
Type 1 T-helper cells:	all
Type 2 T-helper cells:	all

Results

**P value:**	0.0009
**FDR:**	10%

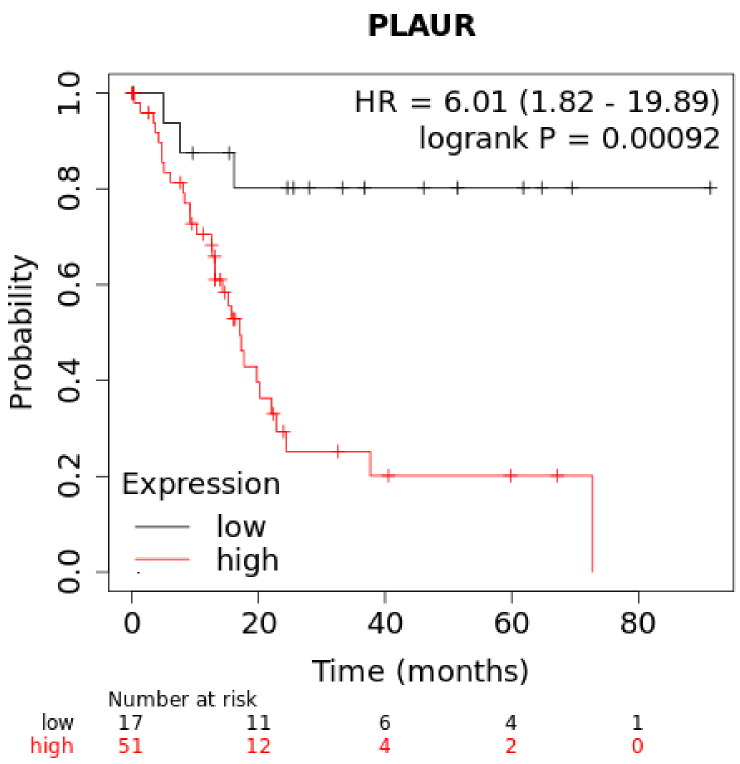

Click here to download the plot in TIFF format

Download plot as a PDF

Download p values vs. cutoff table

Median survival

Low expression cohort (months)	High expression cohort (months)
NA	NA

**RNAseq ID:**	LYPD5 -
**Survival:**	OS
**Auto select best cutoff:**	checked
**Follow up threshold:**	all
**Censore at threshold:**	checked
**Compute median over entire database:**	false
**Cutoff value used in analysis:**	78
**Expression range of the probe:**	1 - 578
**Invert HR values below 1:**	not checked

Restrictions

Tumor type: Pancreatic ductal adenocarcinoma

Restrict analysis to subtypes...

Stage:	all
Gender:	all
Race:	all
Grade:	all
Mutation burden:	all

Restrict analysis based on cellular content...

Basophils: all

B-cells:	all
CD4+ memory T-cells:	all
CD8+ T-cells:	all
Eosinophils:	all
Macrophages:	decreased
Mesenchymal stem cells:	all
Natural killer T-cells:	all
Regulatory T-cells:	all
Type 1 T-helper cells:	all
Type 2 T-helper cells:	all

Results

P value: 1.2e-5
FDR: 1%

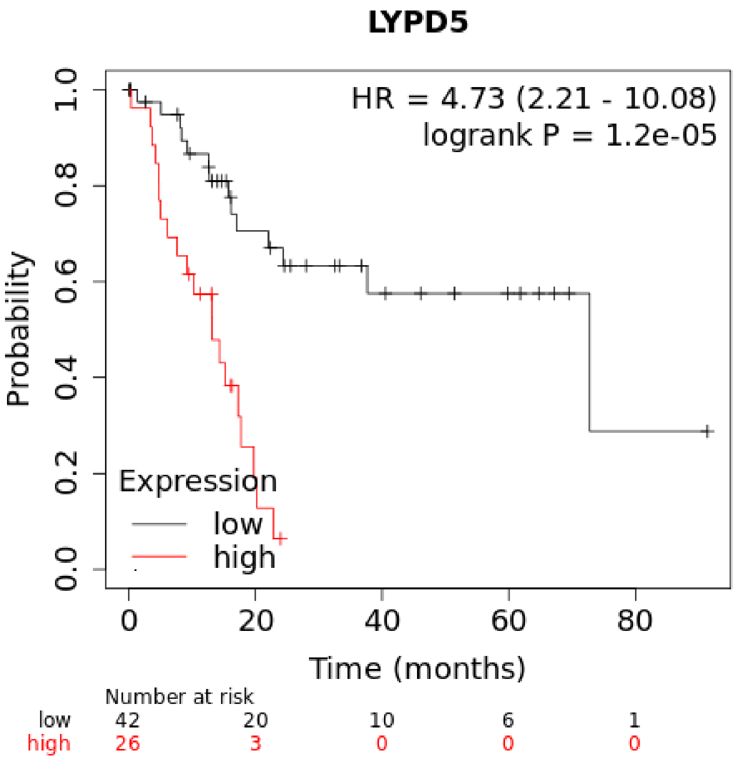

Click here to download the plot in TIFF format

Download plot as a PDF

Download p values vs. cutoff table

Median survival

Low expression cohort (months)	High expression cohort (months)
72.73	13.13

**RNAseq ID:**	SPACA4 -
**Survival:**	OS
**Auto select best cutoff:**	checked
**Follow up threshold:**	all
**Censore at threshold:**	checked
**Compute median over entire database:**	false
**Cutoff value used in analysis:**	13
**Expression range of the probe:**	0 - 204
**Invert HR values below 1:**	not checked

Restrictions

Tumor type: Pancreatic ductal adenocarcinoma

Restrict analysis to subtypes...

Stage:	all
Gender:	all
Race:	all
Grade:	all
Mutation burden:	all

Restrict analysis based on cellular content...

Basophils:	all
B-cells:	all
CD4+ memory T-cells:	all
CD8+ T-cells:	all
Eosinophils:	all
Macrophages:	decreased
Mesenchymal stem cells:	all
Natural killer T-cells:	all
Regulatory T-cells:	all
Type 1 T-helper cells:	all
Type 2 T-helper cells:	all

Results

P value: 0.0289
FDR: over 50%

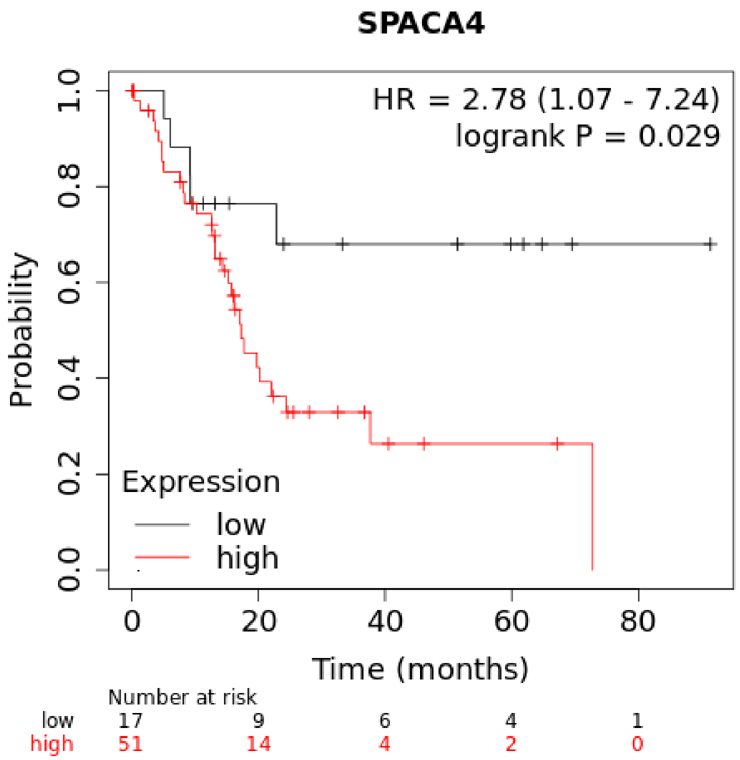

Click here to download the plot in TIFF format

Download plot as a PDF

Download p values vs. cutoff table

Upper quartile survival

Low expression cohort (months)	High expression cohort (months)
22.8	10.27

**RNAseq ID:**	ACRV1 -
**Survival:**	OS
**Auto select best cutoff:**	checked
**Follow up threshold:**	all
**Censore at threshold:**	checked
**Compute median over entire database:**	false
**Cutoff value used in analysis:**	3
**Expression range of the probe:**	0 - 23
**Invert HR values below 1:**	not checked

Restrictions

Tumor type: Pancreatic ductal adenocarcinoma

Restrict analysis to subtypes...

Stage:	all
Gender:	all
Race:	all
Grade:	all
Mutation burden:	all

Restrict analysis based on cellular content...

Basophils:	all
B-cells:	all
CD4+ memory T-cells:	all
CD8+ T-cells:	all
Eosinophils:	all
Macrophages:	decreased
Mesenchymal stem cells:	all
Natural killer T-cells:	all
Regulatory T-cells:	all
Type 1 T-helper cells:	all
Type 2 T-helper cells:	all

Results

**P value:**	0.1669
**FDR:**	100%

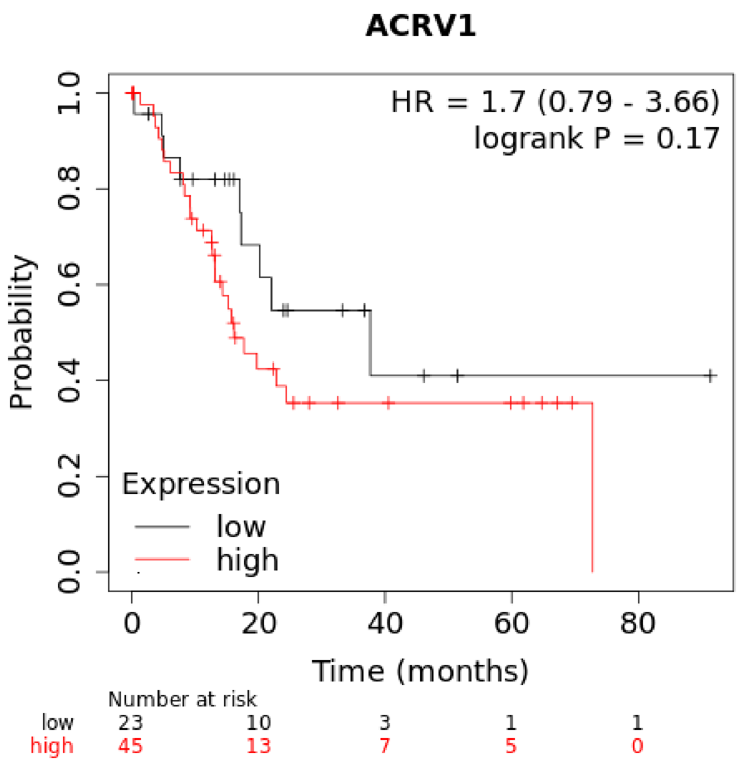

Click here to download the plot in TIFF format

Download plot as a PDF

Download p values vs. cutoff table

Median survival

Low expression cohort (months)	High expression cohort (months)
37.67	16.17

**RNAseq ID:**	PATE1 -
**Survival:**	OS
**Auto select best cutoff:**	checked
**Follow up threshold:**	all
**Censore at threshold:**	checked
**Compute median over entire database:**	false
**Cutoff value used in analysis:**	0
**Expression range of the probe:**	0 - 1
**Invert HR values below 1:**	not checked

Restrictions

Tumor type: Pancreatic ductal adenocarcinoma

Restrict analysis to subtypes...

Stage:	all
Gender:	all
Race:	all
Grade:	all
Mutation burden:	all

Restrict analysis based on cellular content...

Basophils:	all

B-cells:	all
CD4+ memory T-cells:	all
CD8+ T-cells:	all
Eosinophils:	all
Macrophages:	decreased
Mesenchymal stem cells:	all
Natural killer T-cells:	all
Regulatory T-cells:	all
Type 1 T-helper cells:	all
Type 2 T-helper cells:	all

Results

P value: 0.0119
FDR: over 50%

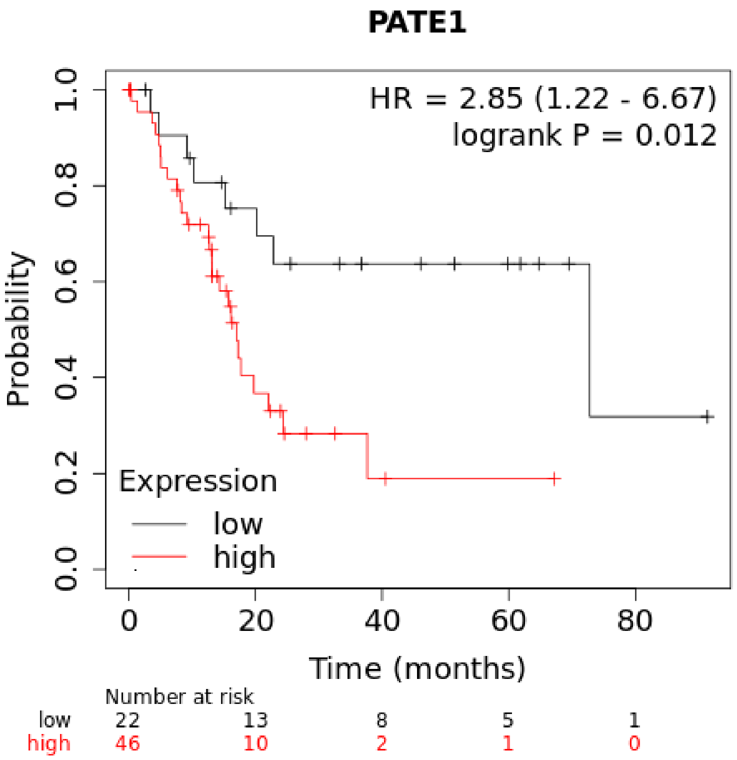

Click here to download the plot in TIFF format

Download plot as a PDF

Download p values vs. cutoff table

Median survival

Low expression cohort (months)	High expression cohort (months)
72.73	17.03

**RNAseq ID:**	PATE2 -
**Survival:**	OS
**Auto select best cutoff:**	checked
**Follow up threshold:**	all
**Censore at threshold:**	checked
**Compute median over entire database:**	false
**Cutoff value used in analysis:**	0
**Expression range of the probe:**	0 - 2
**Invert HR values below 1:**	not checked

Restrictions

Tumor type: Pancreatic ductal adenocarcinoma

Restrict analysis to subtypes...

Stage:	all
Gender:	all
Race:	all
Grade:	all
Mutation burden:	all

Restrict analysis based on cellular content...

Basophils:	all
B-cells:	all
CD4+ memory T-cells:	all
CD8+ T-cells:	all
Eosinophils:	all
Macrophages:	decreased
Mesenchymal stem cells:	all
Natural killer T-cells:	all
Regulatory T-cells:	all
Type 1 T-helper cells:	all
Type 2 T-helper cells:	all

Results

P value: 0.0998
FDR: 100%

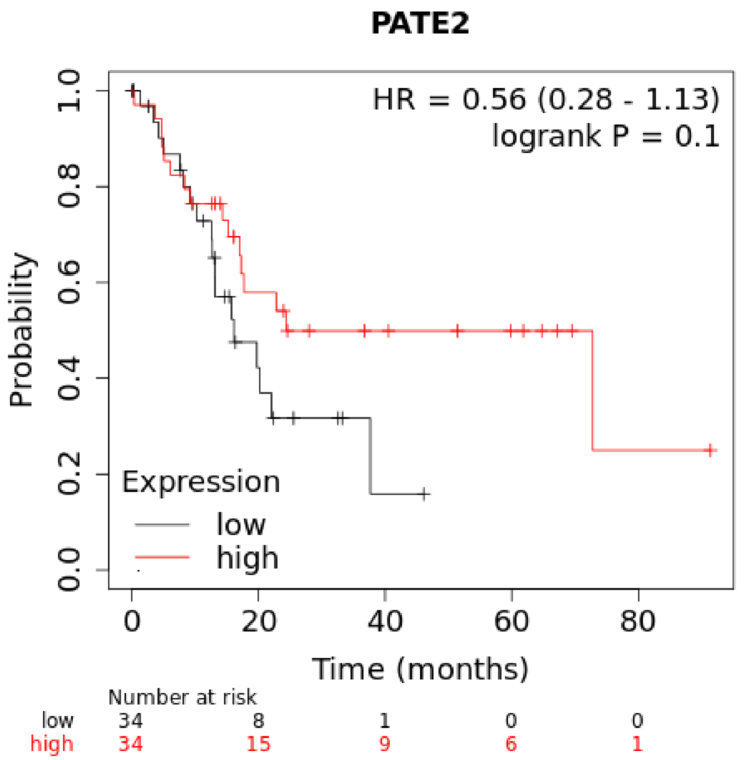

Click here to download the plot in TIFF format

Download plot as a PDF

Download p values vs. cutoff table

Median survival

Low expression cohort (months)	High expression cohort (months)
16.17	24.4

**RNAseq ID:**	PATE3 -
**Survival:**	OS
**Auto select best cutoff:**	checked
**Follow up threshold:**	all
**Censore at threshold:**	checked
**Compute median over entire database:**	false
**Cutoff value used in analysis:**	0
**Expression range of the probe:**	0 - 1
**Invert HR values below 1:**	not checked

Restrictions

Tumor type: Pancreatic ductal adenocarcinoma

Restrict analysis to subtypes...

Stage:	all
Gender:	all
Race:	all
Grade:	all
Mutation burden:	all

Restrict analysis based on cellular content...

Basophils:	all
B-cells:	all
CD4+ memory T-cells:	all
CD8+ T-cells:	all
Eosinophils:	all
Macrophages:	decreased
Mesenchymal stem cells:	all
Natural killer T-cells:	all
Regulatory T-cells:	all
Type 1 T-helper cells:	all
Type 2 T-helper cells:	all

Results

**P value:**	0.0119
**FDR:**	over 50%

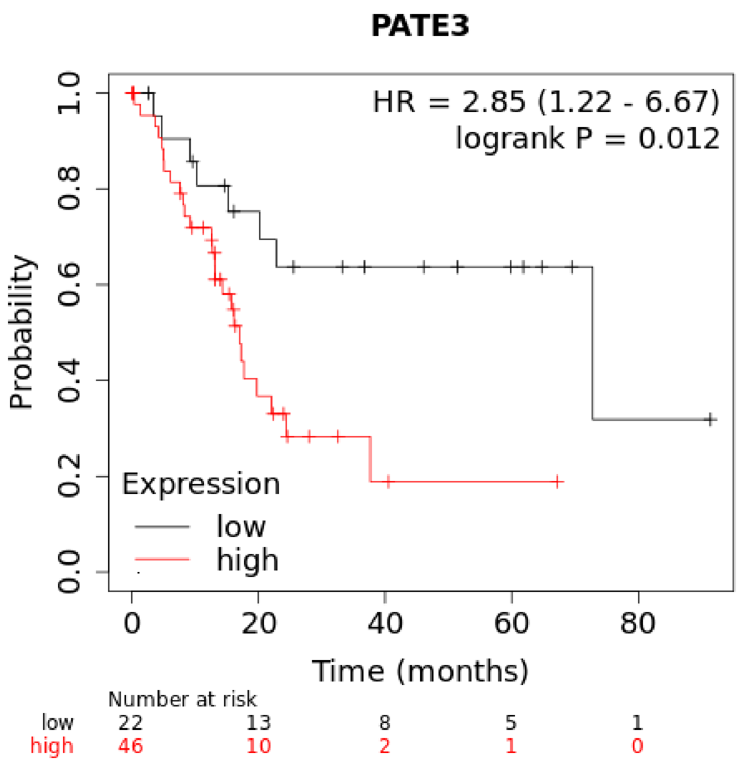

Click here to download the plot in TIFF format

Download plot as a PDF

Download p values vs. cutoff table

Median survival

Low expression cohort (months)	High expression cohort (months)
72.73	17.03

RNAseq ID: PATE4 -
Survival: OS
Auto select best cutoff: checked
Follow up threshold: all
Censore at threshold: checked
Compute median over entire database: false
Cutoff value used in analysis: 0
Expression range of the probe: 0 - 3
Invert HR values below 1: not checked

Restrictions

Tumor type: Pancreatic ductal adenocarcinoma

Restrict analysis to subtypes...

Stage: all
Gender: all
Race: all
Grade: all
Mutation burden: all

Restrict analysis based on cellular content...

Basophils: all

B-cells:	all
CD4+ memory T-cells:	all
CD8+ T-cells:	all
Eosinophils:	all
Macrophages:	decreased
Mesenchymal stem cells:	all
Natural killer T-cells:	all
Regulatory T-cells:	all
Type 1 T-helper cells:	all
Type 2 T-helper cells:	all

Results

P value: 0.4601
FDR: 100%

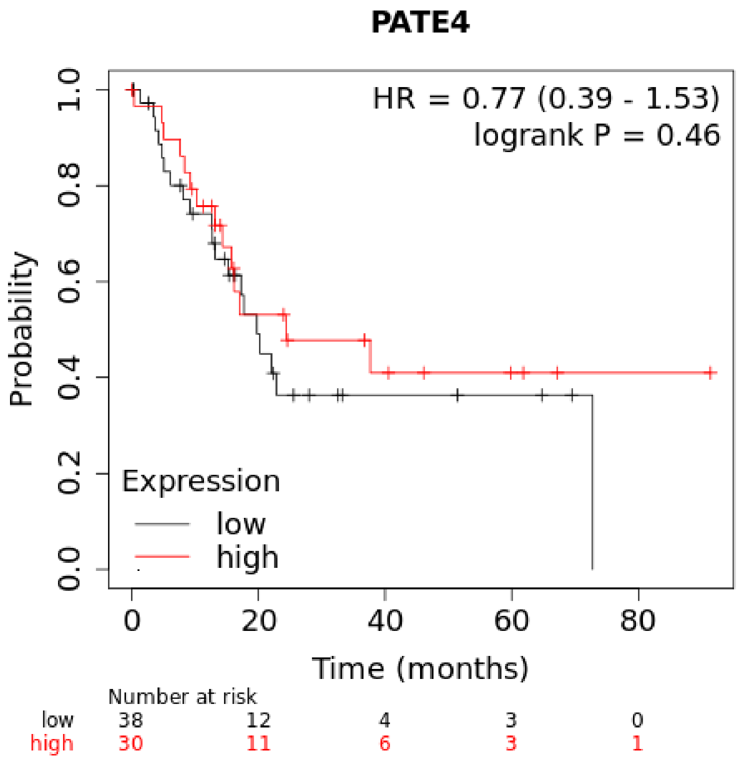

Click here to download the plot in TIFF format

Download plot as a PDF

Download p values vs. cutoff table

Median survival

Low expression cohort (months)	High expression cohort (months)
19.77	24.4

**RNAseq ID:**	CD59 -
**Survival:**	OS
**Auto select best cutoff:**	checked
**Follow up threshold:**	all
**Censore at threshold:**	checked
**Compute median over entire database:**	false
**Cutoff value used in analysis:**	15534
**Expression range of the probe:**	2610 - 39336
**Invert HR values below 1:**	not checked

Restrictions

Tumor type: Pancreatic ductal adenocarcinoma

Restrict analysis to subtypes...

Stage:	all
Gender:	all
Race:	all
Grade:	all
Mutation burden:	all

Restrict analysis based on cellular content...

Basophils:	all
B-cells:	all
CD4+ memory T-cells:	all
CD8+ T-cells:	all
Eosinophils:	all
Macrophages:	decreased
Mesenchymal stem cells:	all
Natural killer T-cells:	all
Regulatory T-cells:	all
Type 1 T-helper cells:	all
Type 2 T-helper cells:	all

Results

P value: 0.0003
FDR: 5%

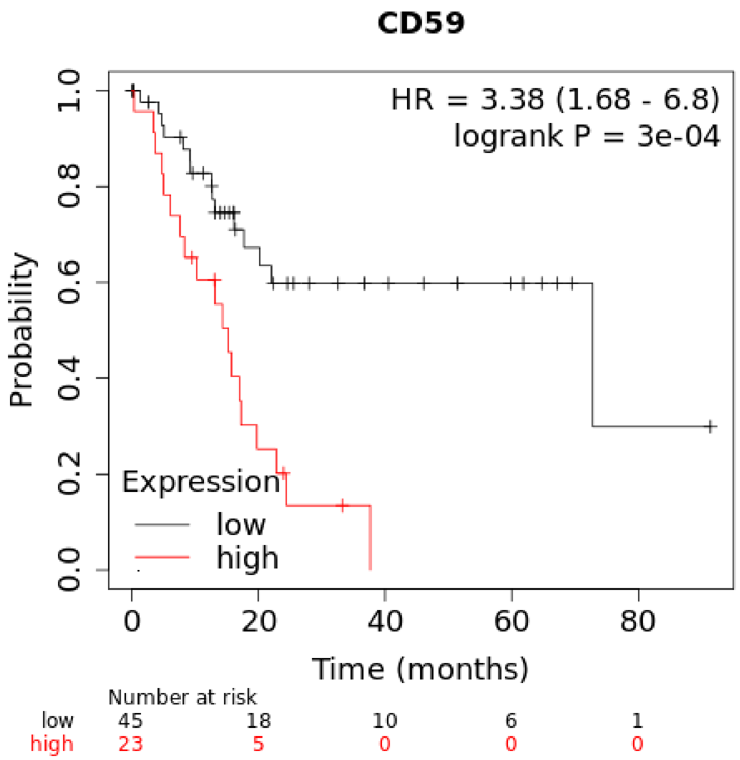

Click here to download the plot in TIFF format

Download plot as a PDF

Download p values vs. cutoff table

Median survival

Low expression cohort (months)	High expression cohort (months)
72.73	15.27

**RNAseq ID:**	LY6G6C -
**Survival:**	OS
**Auto select best cutoff:**	checked
**Follow up threshold:**	all
**Censore at threshold:**	checked
**Compute median over entire database:**	false
**Cutoff value used in analysis:**	14
**Expression range of the probe:**	0 - 251
**Invert HR values below 1:**	not checked

Restrictions

Tumor type: Pancreatic ductal adenocarcinoma

Restrict analysis to subtypes...

Stage:	all
Gender:	all
Race:	all
Grade:	all
Mutation burden:	all

Restrict analysis based on cellular content...

Basophils:	all
B-cells:	all
CD4+ memory T-cells:	all
CD8+ T-cells:	all
Eosinophils:	all
Macrophages:	decreased
Mesenchymal stem cells:	all
Natural killer T-cells:	all
Regulatory T-cells:	all
Type 1 T-helper cells:	all
Type 2 T-helper cells:	all

Results

P value: 0.0002
FDR: 2%

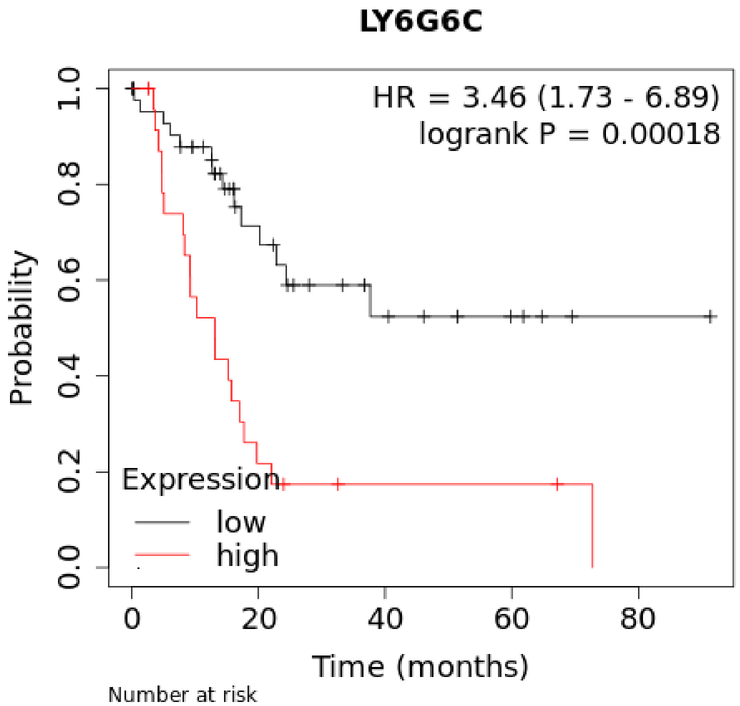

Number at risk

low	44	18	8	4	1
high	24	5	2	2	0

Click here to download the plot in TIFF format

Download plot as a PDF

Download p values vs. cutoff table

Upper quartile survival

Low expression cohort (months)	High expression cohort (months)
17.27	5.1

**RNAseq ID:**	LY6G6D -
**Survival:**	OS
**Auto select best cutoff:**	checked
**Follow up threshold:**	all
**Censore at threshold:**	checked
**Compute median over entire database:**	false
**Cutoff value used in analysis:**	0
**Expression range of the probe:**	0 - 2
**Invert HR values below 1:**	not checked

Restrictions

Tumor type: Pancreatic ductal adenocarcinoma

Restrict analysis to subtypes...

Stage:	all
Gender:	all
Race:	all
Grade:	all
Mutation burden:	all

Restrict analysis based on cellular content...

Basophils:	all

B-cells:	all
CD4+ memory T-cells:	all
CD8+ T-cells:	all
Eosinophils:	all
Macrophages:	decreased
Mesenchymal stem cells:	all
Natural killer T-cells:	all
Regulatory T-cells:	all
Type 1 T-helper cells:	all
Type 2 T-helper cells:	all

Results

P value: 0.0142
FDR: over 50%

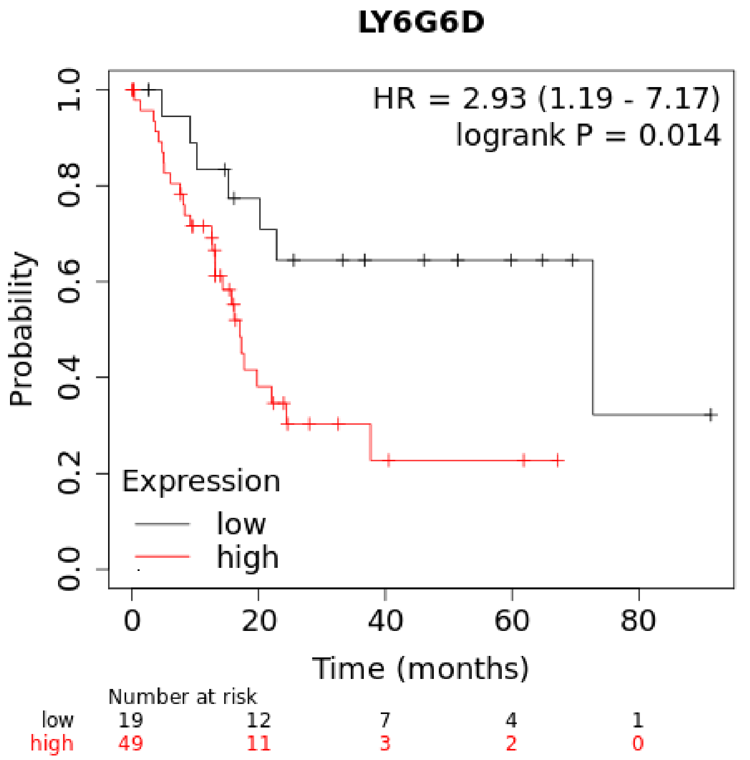

Click here to download the plot in TIFF format

Download plot as a PDF

Download p values vs. cutoff table

Median survival

Low expression cohort (months)	High expression cohort (months)
72.73	17.03

**RNAseq ID:**	LY6G6F -
**Survival:**	OS
**Auto select best cutoff:**	checked
**Follow up threshold:**	all
**Censore at threshold:**	checked
**Compute median over entire database:**	false
**Cutoff value used in analysis:**	0
**Expression range of the probe:**	0 - 5
**Invert HR values below 1:**	not checked

Restrictions

Tumor type: Pancreatic ductal adenocarcinoma

Restrict analysis to subtypes...

Stage:	all
Gender:	all
Race:	all
Grade:	all
Mutation burden:	all

Restrict analysis based on cellular content...

Basophils:	all
B-cells:	all
CD4+ memory T-cells:	all
CD8+ T-cells:	all
Eosinophils:	all
Macrophages:	decreased
Mesenchymal stem cells:	all
Natural killer T-cells:	all
Regulatory T-cells:	all
Type 1 T-helper cells:	all
Type 2 T-helper cells:	all

Results

P value: 0.0014
FDR: 20%

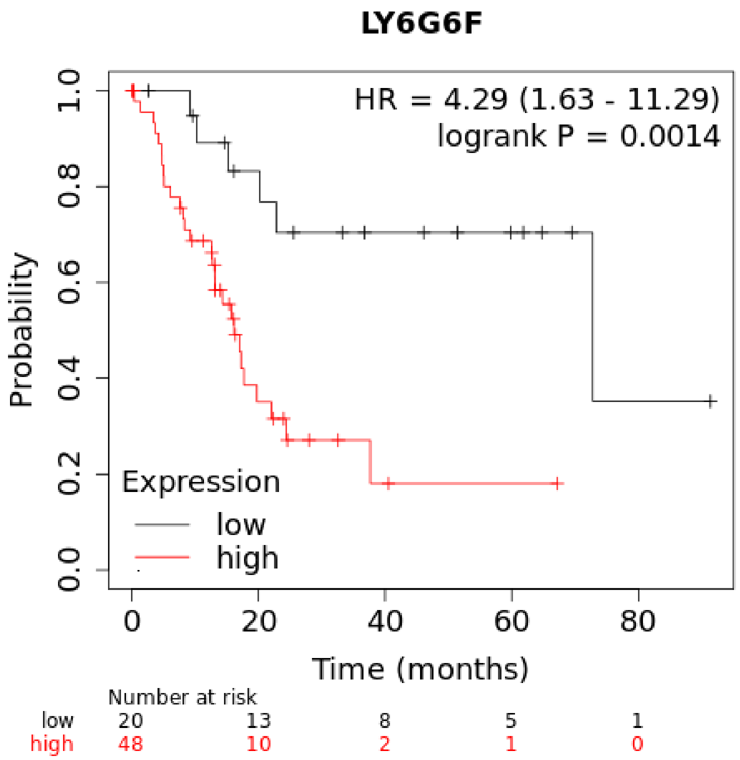

Click here to download the plot in TIFF format

Download plot as a PDF

Download p values vs. cutoff table

Median survival

Low expression cohort (months)	High expression cohort (months)
72.73	16.17

**RNAseq ID:**	LY6G5C -
**Survival:**	OS
**Auto select best cutoff:**	checked
**Follow up threshold:**	all
**Censore at threshold:**	checked
**Compute median over entire database:**	false
**Cutoff value used in analysis:**	50
**Expression range of the probe:**	15 - 586
**Invert HR values below 1:**	not checked

Restrictions

Tumor type: Pancreatic ductal adenocarcinoma

Restrict analysis to subtypes...

Stage:	all
Gender:	all
Race:	all
Grade:	all
Mutation burden:	all

Restrict analysis based on cellular content...

Basophils:	all
B-cells:	all
CD4+ memory T-cells:	all
CD8+ T-cells:	all
Eosinophils:	all
Macrophages:	decreased
Mesenchymal stem cells:	all
Natural killer T-cells:	all
Regulatory T-cells:	all
Type 1 T-helper cells:	all
Type 2 T-helper cells:	all

Results

**P value:**	1.6e-5
**FDR:**	1%

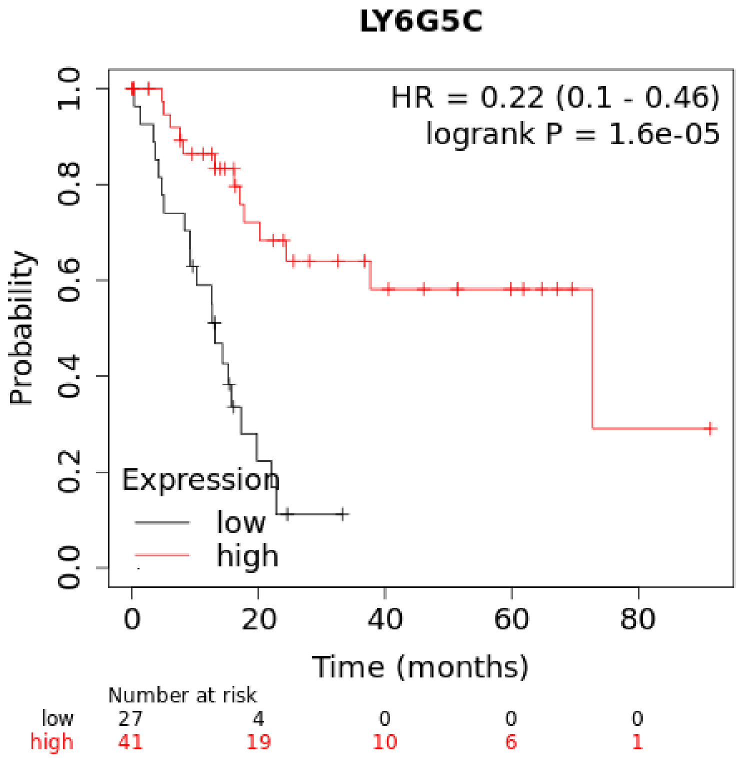

Click here to download the plot in TIFF format

Download plot as a PDF

Download p values vs. cutoff table

Median survival

Low expression cohort (months)	High expression cohort (months)
13.13	72.73

RNAseq ID: LY6G5B -
Survival: OS
Auto select best cutoff: checked
Follow up threshold: all
Censore at threshold: checked
Compute median over entire database: false
Cutoff value used in analysis: 81
Expression range of the probe: 3 - 192
Invert HR values below 1: not checked

Restrictions

Tumor type: Pancreatic ductal adenocarcinoma

Restrict analysis to subtypes...

Stage: all
Gender: all
Race: all
Grade: all
Mutation burden: all

Restrict analysis based on cellular content...

Basophils: all

B-cells:	all
CD4+ memory T-cells:	all
CD8+ T-cells:	all
Eosinophils:	all
Macrophages:	decreased
Mesenchymal stem cells:	all
Natural killer T-cells:	all
Regulatory T-cells:	all
Type 1 T-helper cells:	all
Type 2 T-helper cells:	all

Results

P value: 0.0441
FDR: over 50%

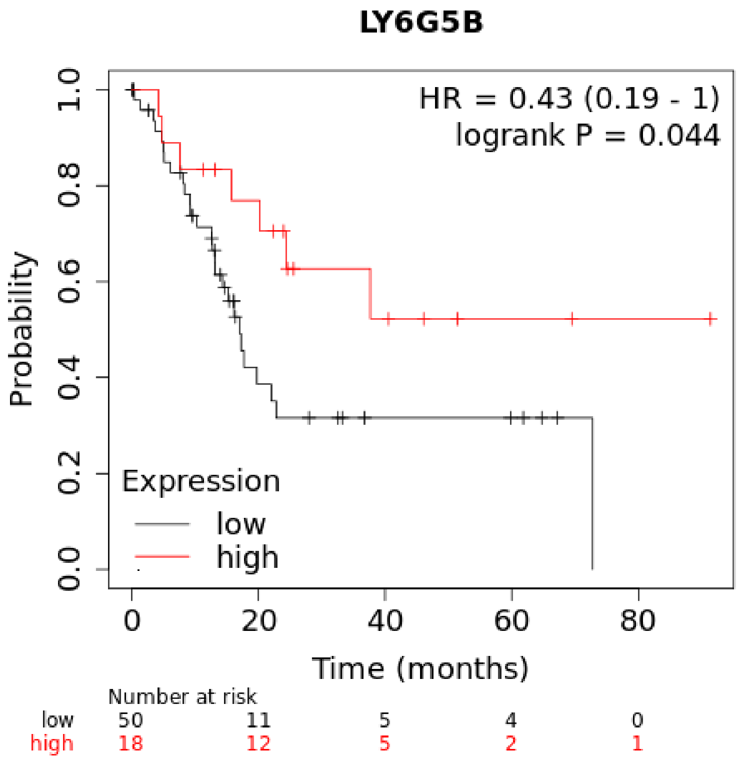

[Click here to download the plot in TIFF format]

[Download plot as a PDF]

[Download p values vs. cutoff table]

Upper quartile survival

Low expression cohort (months)	High expression cohort (months)
9.27	20.23

You can save the plots by right-clicking the image and then selecting "Save image as...". To generate a high resolution TIFF image, please adjust the "Settings" in the analysis page.

KMplot.com Copyright © 2009-2020 | Contact | Sitemap

Figure S6: KM plots and other raw data for the data depicted in Table 6

Pan-cancer

KM plotter | Home | Vote | Download | Updates | Contact

The desired RNAseq ID is valid: PSCA (-), LY6K (-), SLURP1 (-), LYPD2 (-), LY6D (-), GML (-), LY6E (-), LY6L (-), LY6H (-), GPIHBP1 (-), LYPD4 (-), CD177 (-), TEX101 (-), LYPD3 (-), PINLYP (-), PLAUR (-), LYPD5 (-), SPACA4 (-), ACRV1 (-), PATE1 (-), PATE2 (-), PATE3 (-), PATE4 (-), CD59 (-), LY6G6C (-), LY6G6D (-), LY6G6F (-), LY6G5C (-), LY6G5B (-),

**RNAseq ID:**	PSCA -
**Survival:**	OS
**Auto select best cutoff:**	checked
**Follow up threshold:**	all
**Censore at threshold:**	checked
**Compute median over entire database:**	false
**Cutoff value used in analysis:**	192
**Expression range of the probe:**	0 - 49321
**Invert HR values below 1:**	not checked

Restrictions

Tumor type: Pancreatic ductal adenocarcinoma

Restrict analysis to subtypes...

Stage:	all
Gender:	all
Race:	all
Grade:	all
Mutation burden:	all

Restrict analysis based on cellular content...

Basophils:	all
B-cells:	all
CD4+ memory T-cells:	all
CD8+ T-cells:	all
Eosinophils:	all
Macrophages:	all
Mesenchymal stem cells:	all
Natural killer T-cells:	enriched
Regulatory T-cells:	all
Type 1 T-helper cells:	all
Type 2 T-helper cells:	all

Results

**P value:**	0.0041
**FDR:**	20%

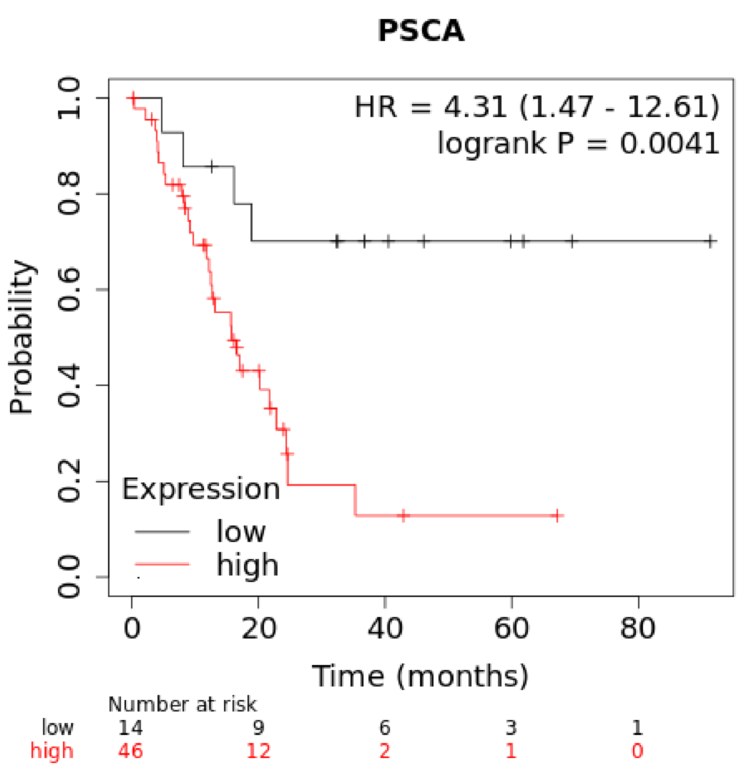

Click here to download the plot in TIFF format

Download plot as a PDF

Download p values vs. cutoff table

Upper quartile survival

Low expression cohort (months)	High expression cohort (months)
18.93	8.9

**RNAseq ID:**	LY6K -
**Survival:**	OS
**Auto select best cutoff:**	checked
**Follow up threshold:**	all
**Censore at threshold:**	checked
**Compute median over entire database:**	false
**Cutoff value used in analysis:**	6
**Expression range of the probe:**	0 - 1825
**Invert HR values below 1:**	not checked

Restrictions

Tumor type: Pancreatic ductal adenocarcinoma

Restrict analysis to subtypes...

Stage:	all
Gender:	all
Race:	all
Grade:	all
Mutation burden:	all

Restrict analysis based on cellular content...

Basophils:	all

B-cells:	all
CD4+ memory T-cells:	all
CD8+ T-cells:	all
Eosinophils:	all
Macrophages:	all
Mesenchymal stem cells:	all
Natural killer T-cells:	enriched
Regulatory T-cells:	all
Type 1 T-helper cells:	all
Type 2 T-helper cells:	all

Results

P value: 0.0107
FDR: over 50%

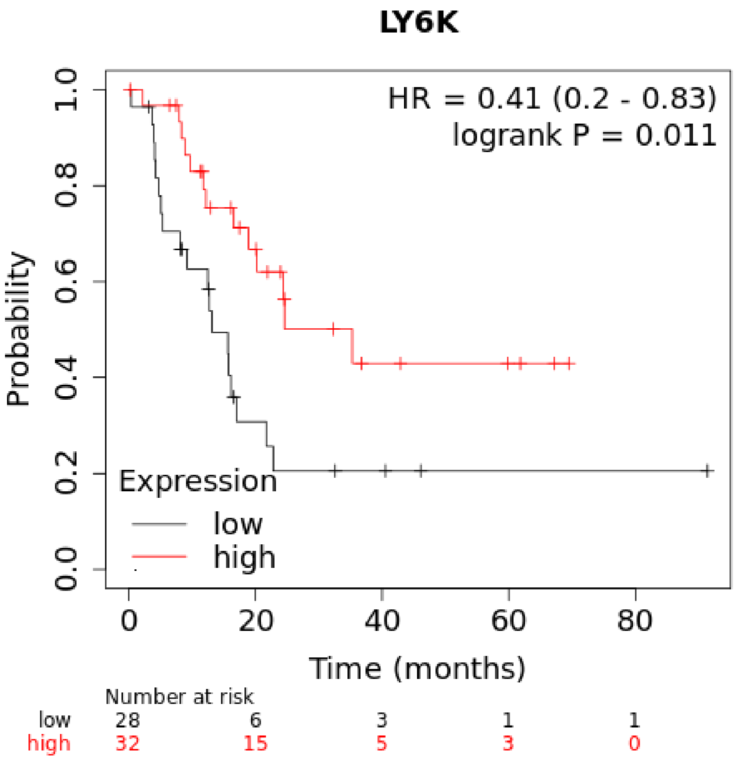

Click here to download the plot in TIFF format

Download plot as a PDF

Download p values vs. cutoff table

Median survival

Low expression cohort (months)	High expression cohort (months)
13.13	35.3

**RNAseq ID:**	SLURP1 -
**Survival:**	OS
**Auto select best cutoff:**	checked
**Follow up threshold:**	all
**Censore at threshold:**	checked
**Compute median over entire database:**	false
**Cutoff value used in analysis:**	1
**Expression range of the probe:**	0 - 279
**Invert HR values below 1:**	not checked

Restrictions

Tumor type: Pancreatic ductal adenocarcinoma

Restrict analysis to subtypes...

Stage:	all
Gender:	all
Race:	all
Grade:	all
Mutation burden:	all

Restrict analysis based on cellular content...

Basophils:	all
B-cells:	all
CD4+ memory T-cells:	all
CD8+ T-cells:	all
Eosinophils:	all
Macrophages:	all
Mesenchymal stem cells:	all
Natural killer T-cells:	enriched
Regulatory T-cells:	all
Type 1 T-helper cells:	all
Type 2 T-helper cells:	all

Results

P value: 9.6e-5
FDR: 2%

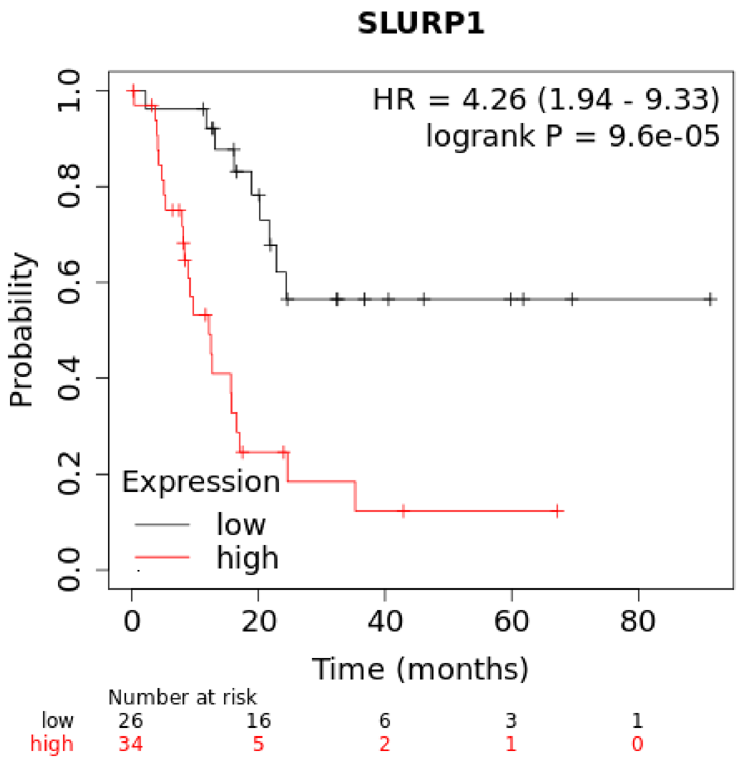

Click here to download the plot in TIFF format

Download plot as a PDF

Download p values vs. cutoff table

Upper quartile survival

Low expression cohort (months)	High expression cohort (months)
20.23	7.97

**RNAseq ID:**	LYPD2 -
**Survival:**	OS
**Auto select best cutoff:**	checked
**Follow up threshold:**	all
**Censore at threshold:**	checked
**Compute median over entire database:**	false
**Cutoff value used in analysis:**	29
**Expression range of the probe:**	0 - 2799
**Invert HR values below 1:**	not checked

Restrictions

Tumor type: Pancreatic ductal adenocarcinoma

Restrict analysis to subtypes...

Stage:	all
Gender:	all
Race:	all
Grade:	all
Mutation burden:	all

Restrict analysis based on cellular content...

Basophils:	all
B-cells:	all
CD4+ memory T-cells:	all
CD8+ T-cells:	all
Eosinophils:	all
Macrophages:	all
Mesenchymal stem cells:	all
Natural killer T-cells:	enriched
Regulatory T-cells:	all
Type 1 T-helper cells:	all
Type 2 T-helper cells:	all

Results

**P value:**	0.0004
**FDR:**	5%

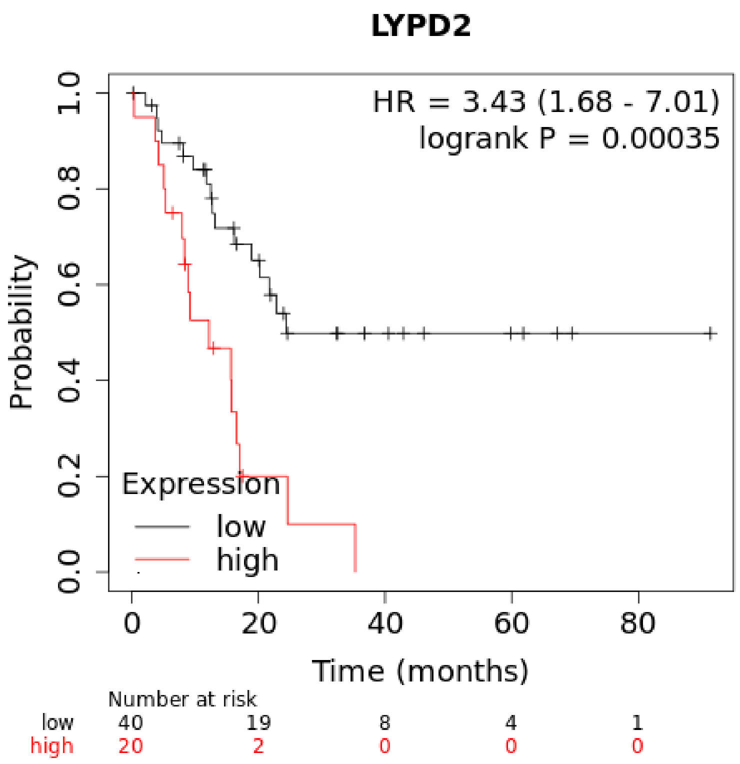

Click here to download the plot in TIFF format

Download plot as a PDF

Download p values vs. cutoff table

Median survival

Low expression cohort (months)	High expression cohort (months)
24.4	12.2

**RNAseq ID:**	LY6D -
**Survival:**	OS
**Auto select best cutoff:**	checked
**Follow up threshold:**	all
**Censore at threshold:**	checked
**Compute median over entire database:**	false
**Cutoff value used in analysis:**	305
**Expression range of the probe:**	0 - 18030
**Invert HR values below 1:**	not checked

Restrictions

Tumor type: Pancreatic ductal adenocarcinoma

Restrict analysis to subtypes...

Stage:	all
Gender:	all
Race:	all
Grade:	all
Mutation burden:	all

Restrict analysis based on cellular content...

Basophils:	all

B-cells: all
CD4+ memory T-cells: all
CD8+ T-cells: all
Eosinophils: all
Macrophages: all
Mesenchymal stem cells: all
Natural killer T-cells: enriched
Regulatory T-cells: all
Type 1 T-helper cells: all
Type 2 T-helper cells: all

Results

P value: 9.2e-5
FDR: 2%

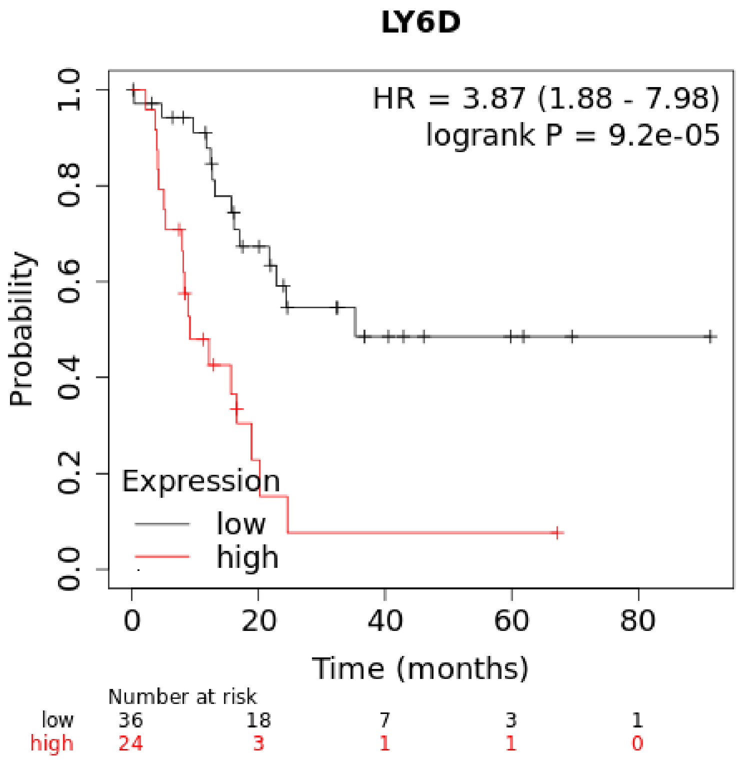

[Click here to download the plot in TIFF format]

[Download plot as a PDF]

[Download p values vs. cutoff table]

Median survival

Low expression cohort (months)	High expression cohort (months)
35.3	9.23

RNAseq ID: GML -
Survival: OS
Auto select best cutoff: checked
Follow up threshold: all
Censore at threshold: checked
Compute median over entire database: false
Cutoff value used in analysis: 0
Expression range of the probe: 0 - 2
Invert HR values below 1: not checked

Restrictions

Tumor type: Pancreatic ductal adenocarcinoma

Restrict analysis to subtypes...

Stage:	all
Gender:	all
Race:	all
Grade:	all
Mutation burden:	all

Restrict analysis based on cellular content...

Basophils:	all
B-cells:	all
CD4+ memory T-cells:	all
CD8+ T-cells:	all
Eosinophils:	all
Macrophages:	all
Mesenchymal stem cells:	all
Natural killer T-cells:	enriched
Regulatory T-cells:	all
Type 1 T-helper cells:	all
Type 2 T-helper cells:	all

Results

P value: 0.0587
FDR: 100%

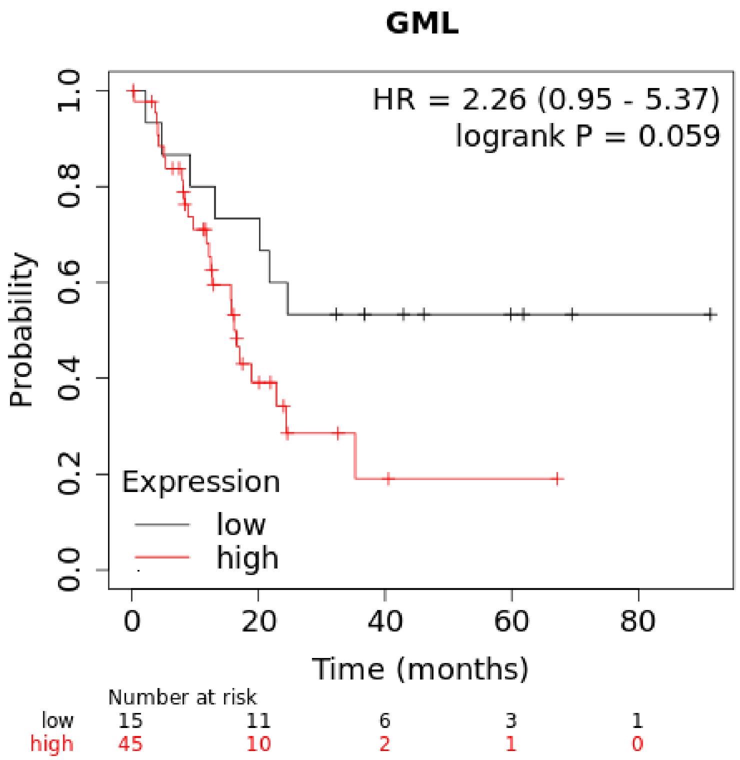

Click here to download the plot in TIFF format

Download plot as a PDF

Download p values vs. cutoff table

Upper quartile survival

Low expression cohort (months)	High expression cohort (months)
13.13	8.9

**RNAseq ID:**	LY6E -
**Survival:**	OS
**Auto select best cutoff:**	checked
**Follow up threshold:**	all
**Censore at threshold:**	checked
**Compute median over entire database:**	false
**Cutoff value used in analysis:**	5980
**Expression range of the probe:**	504 - 56404
**Invert HR values below 1:**	not checked

Restrictions

Tumor type: Pancreatic ductal adenocarcinoma

Restrict analysis to subtypes...

Stage:	all
Gender:	all
Race:	all
Grade:	all
Mutation burden:	all

Restrict analysis based on cellular content...

Basophils:	all
B-cells:	all
CD4+ memory T-cells:	all
CD8+ T-cells:	all
Eosinophils:	all
Macrophages:	all
Mesenchymal stem cells:	all
Natural killer T-cells:	enriched
Regulatory T-cells:	all
Type 1 T-helper cells:	all
Type 2 T-helper cells:	all

Results

**P value:**	0.0338
**FDR:**	over 50%

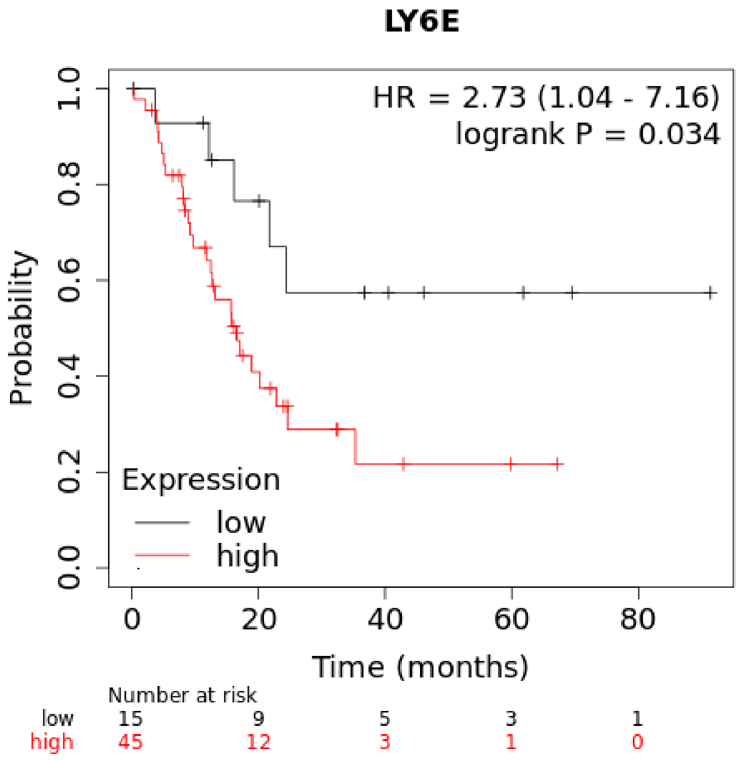

Click here to download the plot in TIFF format

Download plot as a PDF

Download p values vs. cutoff table

Upper quartile survival

Low expression cohort (months)	High expression cohort (months)
21.73	8.33

**RNAseq ID:**	LY6L -
**Survival:**	OS
**Auto select best cutoff:**	checked
**Follow up threshold:**	all
**Censore at threshold:**	checked
**Compute median over entire database:**	false
**Cutoff value used in analysis:**	0
**Expression range of the probe:**	0 - 4
**Invert HR values below 1:**	not checked

Restrictions

Tumor type: Pancreatic ductal adenocarcinoma

Restrict analysis to subtypes...

Stage:	all
Gender:	all
Race:	all
Grade:	all
Mutation burden:	all

Restrict analysis based on cellular content...

Basophils:	all

B-cells:	all
CD4+ memory T-cells:	all
CD8+ T-cells:	all
Eosinophils:	all
Macrophages:	all
Mesenchymal stem cells:	all
Natural killer T-cells:	enriched
Regulatory T-cells:	all
Type 1 T-helper cells:	all
Type 2 T-helper cells:	all

Results

P value: 0.0777
FDR: 100%

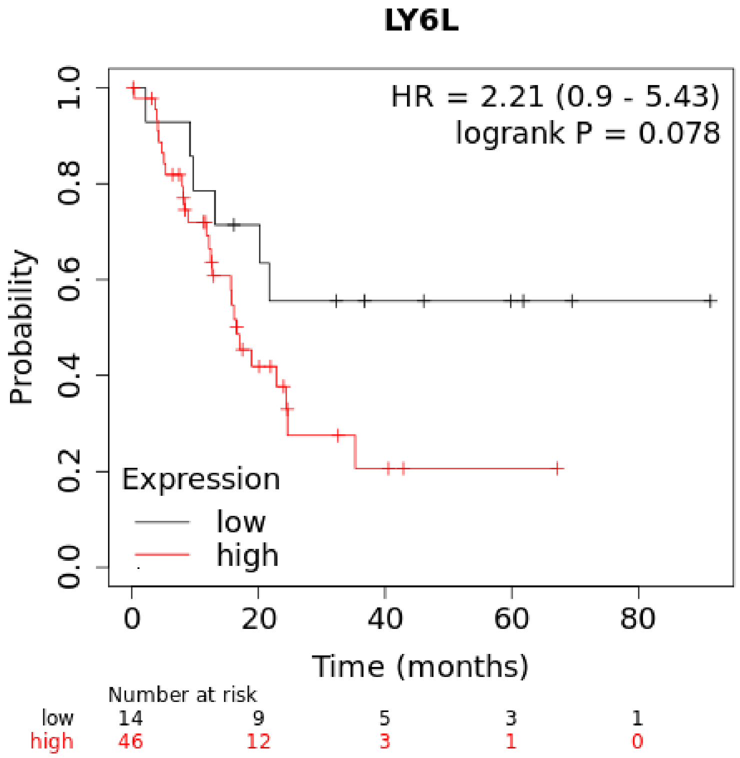

Click here to download the plot in TIFF format

Download plot as a PDF

Download p values vs. cutoff table

Upper quartile survival

Low expression cohort (months)	High expression cohort (months)
13.13	8.33

**RNAseq ID:**	LY6H -
**Survival:**	OS
**Auto select best cutoff:**	checked
**Follow up threshold:**	all
**Censore at threshold:**	checked
**Compute median over entire database:**	false
**Cutoff value used in analysis:**	43
**Expression range of the probe:**	4 - 9495
**Invert HR values below 1:**	not checked

Restrictions

Tumor type: Pancreatic ductal adenocarcinoma

Restrict analysis to subtypes...

Stage:	all
Gender:	all
Race:	all
Grade:	all
Mutation burden:	all

Restrict analysis based on cellular content...

Basophils:	all
B-cells:	all
CD4+ memory T-cells:	all
CD8+ T-cells:	all
Eosinophils:	all
Macrophages:	all
Mesenchymal stem cells:	all
Natural killer T-cells:	enriched
Regulatory T-cells:	all
Type 1 T-helper cells:	all
Type 2 T-helper cells:	all

Results

P value: 0.0085
FDR: 50%

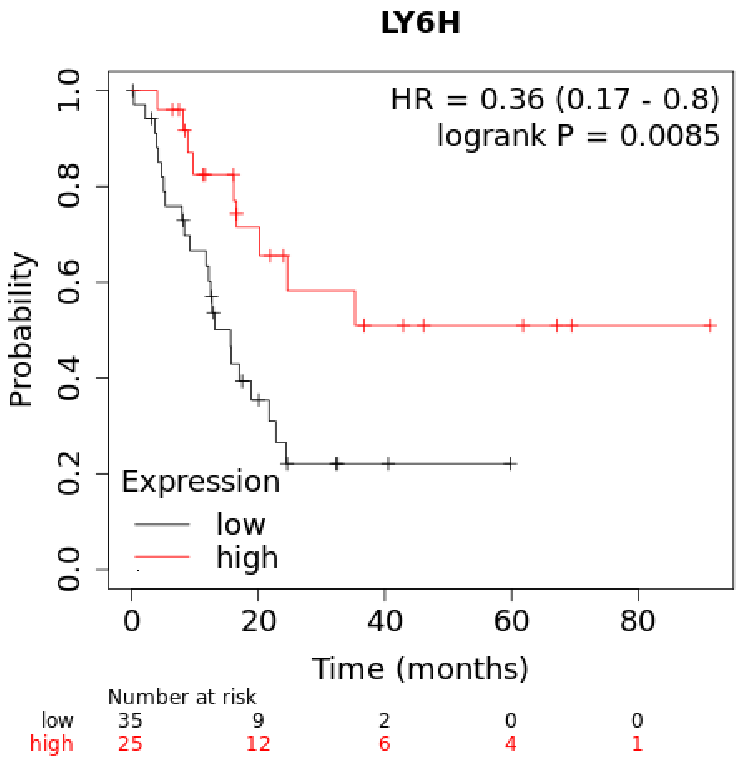

Click here to download the plot in TIFF format

Download plot as a PDF

Download p values vs. cutoff table

Upper quartile survival

Low expression cohort (months)	High expression cohort (months)
7.97	16.6

**RNAseq ID:**	GPIHBP1 -
**Survival:**	OS
**Auto select best cutoff:**	checked
**Follow up threshold:**	all
**Censore at threshold:**	checked
**Compute median over entire database:**	false
**Cutoff value used in analysis:**	19
**Expression range of the probe:**	5 - 344
**Invert HR values below 1:**	not checked

Restrictions

Tumor type: Pancreatic ductal adenocarcinoma

Restrict analysis to subtypes...

Stage:	all
Gender:	all
Race:	all
Grade:	all
Mutation burden:	all

Restrict analysis based on cellular content...

Basophils:	all
B-cells:	all
CD4+ memory T-cells:	all
CD8+ T-cells:	all
Eosinophils:	all
Macrophages:	all
Mesenchymal stem cells:	all
Natural killer T-cells:	enriched
Regulatory T-cells:	all
Type 1 T-helper cells:	all
Type 2 T-helper cells:	all

Results

**P value:**	0.474
**FDR:**	100%

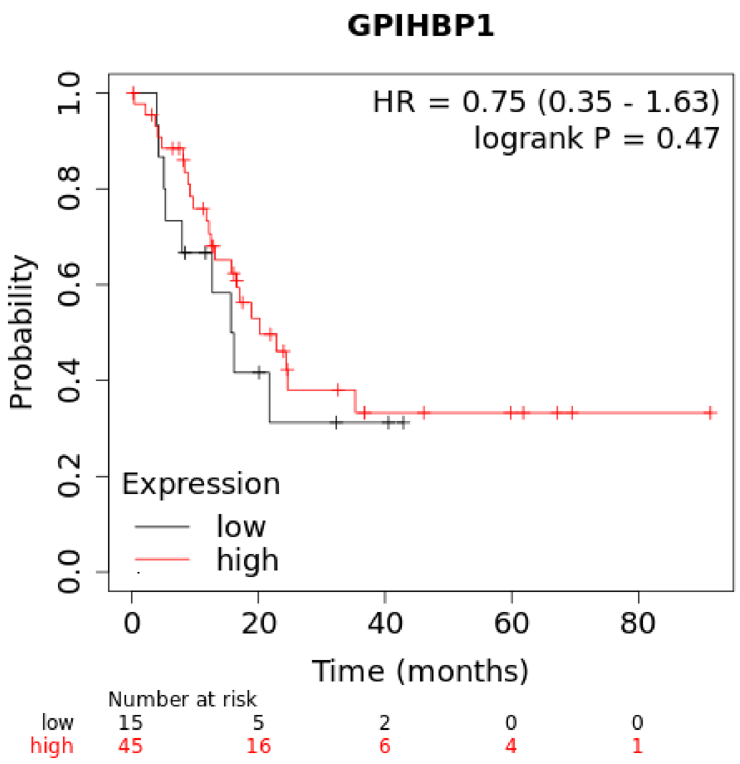

Click here to download the plot in TIFF format

Download plot as a PDF

Download p values vs. cutoff table

Median survival

Low expression cohort (months)	High expression cohort (months)
16.17	20.23

**RNAseq ID:**	LYPD4 -
**Survival:**	OS
**Auto select best cutoff:**	checked
**Follow up threshold:**	all
**Censore at threshold:**	checked
**Compute median over entire database:**	false
**Cutoff value used in analysis:**	0
**Expression range of the probe:**	0 - 18
**Invert HR values below 1:**	not checked

Restrictions

Tumor type: Pancreatic ductal adenocarcinoma

Restrict analysis to subtypes...

Stage:	all
Gender:	all
Race:	all
Grade:	all
Mutation burden:	all

Restrict analysis based on cellular content...

Basophils:	all

B-cells:	all
CD4+ memory T-cells:	all
CD8+ T-cells:	all
Eosinophils:	all
Macrophages:	all
Mesenchymal stem cells:	all
Natural killer T-cells:	enriched
Regulatory T-cells:	all
Type 1 T-helper cells:	all
Type 2 T-helper cells:	all

Results

P value: 0.0627
FDR: 100%

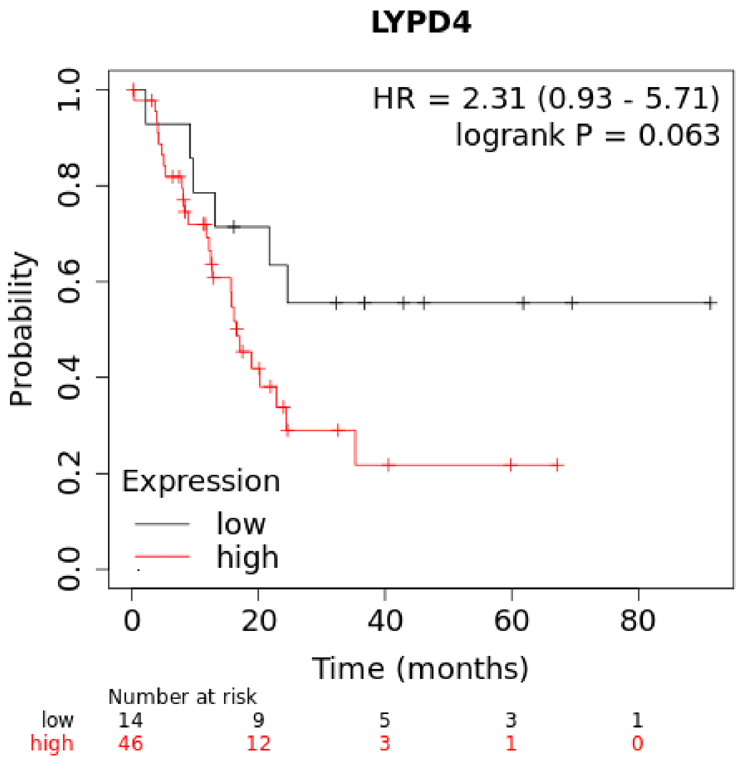

Click here to download the plot in TIFF format

Download plot as a PDF

Download p values vs. cutoff table

Upper quartile survival

Low expression cohort (months)	High expression cohort (months)
13.13	8.33

**RNAseq ID:**	CD177 -
**Survival:**	OS
**Auto select best cutoff:**	checked
**Follow up threshold:**	all
**Censore at threshold:**	checked
**Compute median over entire database:**	false
**Cutoff value used in analysis:**	13
**Expression range of the probe:**	0 - 1414
**Invert HR values below 1:**	not checked

Restrictions

Tumor type: Pancreatic ductal adenocarcinoma

Restrict analysis to subtypes...

Stage:	all
Gender:	all
Race:	all
Grade:	all
Mutation burden:	all

Restrict analysis based on cellular content...

Basophils:	all
B-cells:	all
CD4+ memory T-cells:	all
CD8+ T-cells:	all
Eosinophils:	all
Macrophages:	all
Mesenchymal stem cells:	all
Natural killer T-cells:	enriched
Regulatory T-cells:	all
Type 1 T-helper cells:	all
Type 2 T-helper cells:	all

Results

P value: 0.0169
FDR: over 50%

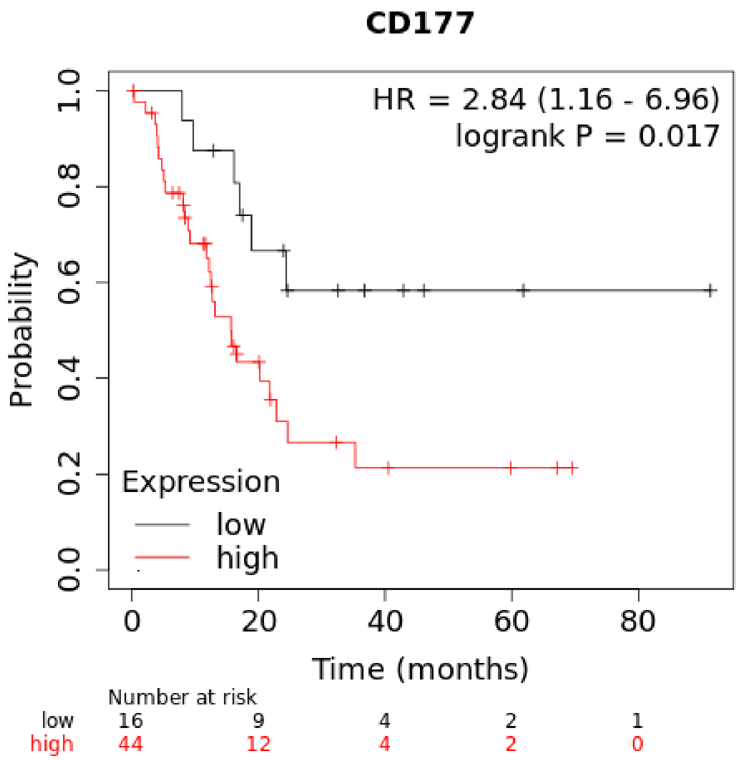

Click here to download the plot in TIFF format

Download plot as a PDF

Download p values vs. cutoff table

Upper quartile survival

Low expression cohort (months)	High expression cohort (months)
17.03	8.33

**RNAseq ID:**	TEX101 -
**Survival:**	OS
**Auto select best cutoff:**	checked
**Follow up threshold:**	all
**Censore at threshold:**	checked
**Compute median over entire database:**	false
**Cutoff value used in analysis:**	2
**Expression range of the probe:**	0 - 20
**Invert HR values below 1:**	not checked

Restrictions

Tumor type: Pancreatic ductal adenocarcinoma

Restrict analysis to subtypes...

Stage:	all
Gender:	all
Race:	all
Grade:	all
Mutation burden:	all

Restrict analysis based on cellular content...

Basophils:	all
B-cells:	all
CD4+ memory T-cells:	all
CD8+ T-cells:	all
Eosinophils:	all
Macrophages:	all
Mesenchymal stem cells:	all
Natural killer T-cells:	enriched
Regulatory T-cells:	all
Type 1 T-helper cells:	all
Type 2 T-helper cells:	all

Results

P value: 0.1515
FDR: 100%

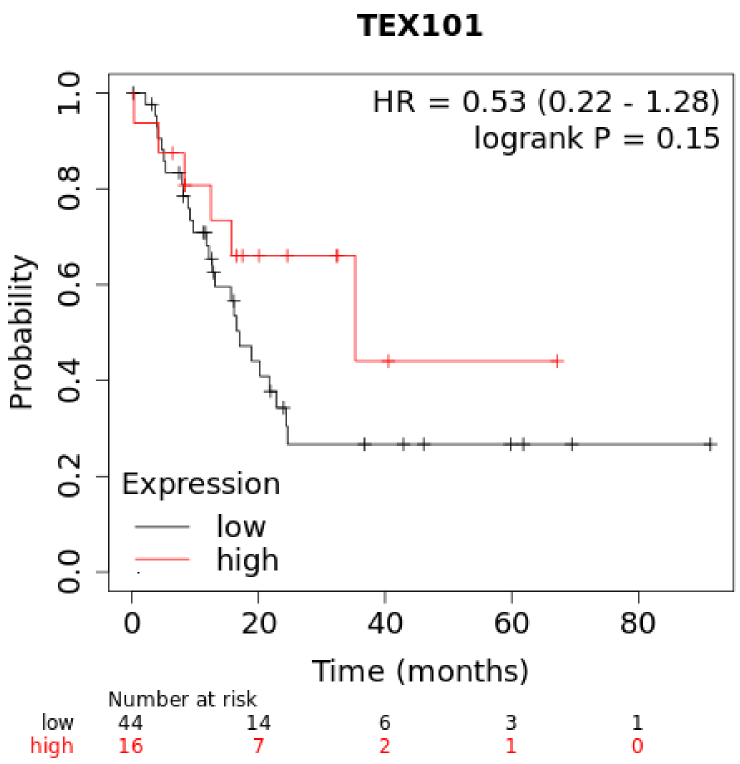

Click here to download the plot in TIFF format

Download plot as a PDF

Download p values vs. cutoff table

Median survival

Low expression cohort (months)	High expression cohort (months)
17.03	35.3

**RNAseq ID:**	LYPD3 -
**Survival:**	OS
**Auto select best cutoff:**	checked
**Follow up threshold:**	all
**Censore at threshold:**	checked
**Compute median over entire database:**	false
**Cutoff value used in analysis:**	269
**Expression range of the probe:**	14 - 3640
**Invert HR values below 1:**	not checked

Restrictions

Tumor type: Pancreatic ductal adenocarcinoma

Restrict analysis to subtypes...

Stage:	all
Gender:	all
Race:	all
Grade:	all
Mutation burden:	all

Restrict analysis based on cellular content...

Basophils:	all

B-cells:	all
CD4+ memory T-cells:	all
CD8+ T-cells:	all
Eosinophils:	all
Macrophages:	all
Mesenchymal stem cells:	all
Natural killer T-cells:	enriched
Regulatory T-cells:	all
Type 1 T-helper cells:	all
Type 2 T-helper cells:	all

Results

P value: 0.3179
FDR: 100%

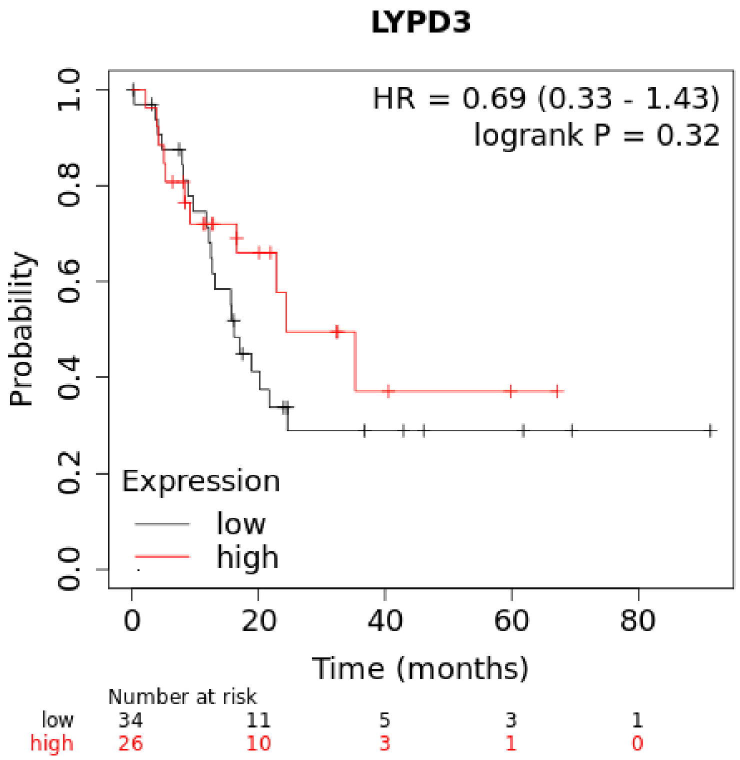

Click here to download the plot in TIFF format

Download plot as a PDF

Download p values vs. cutoff table

Median survival

Low expression cohort (months)	High expression cohort (months)
16.17	24.4

**RNAseq ID:**	PINLYP -
**Survival:**	OS
**Auto select best cutoff:**	checked
**Follow up threshold:**	all
**Censore at threshold:**	checked
**Compute median over entire database:**	false
**Cutoff value used in analysis:**	73
**Expression range of the probe:**	6 - 263
**Invert HR values below 1:**	not checked

Restrictions

Tumor type: Pancreatic ductal adenocarcinoma

Restrict analysis to subtypes...

Stage:	all
Gender:	all
Race:	all
Grade:	all
Mutation burden:	all

Restrict analysis based on cellular content...

Basophils:	all
B-cells:	all
CD4+ memory T-cells:	all
CD8+ T-cells:	all
Eosinophils:	all
Macrophages:	all
Mesenchymal stem cells:	all
Natural killer T-cells:	enriched
Regulatory T-cells:	all
Type 1 T-helper cells:	all
Type 2 T-helper cells:	all

Results

P value: 0.0828
FDR: 100%

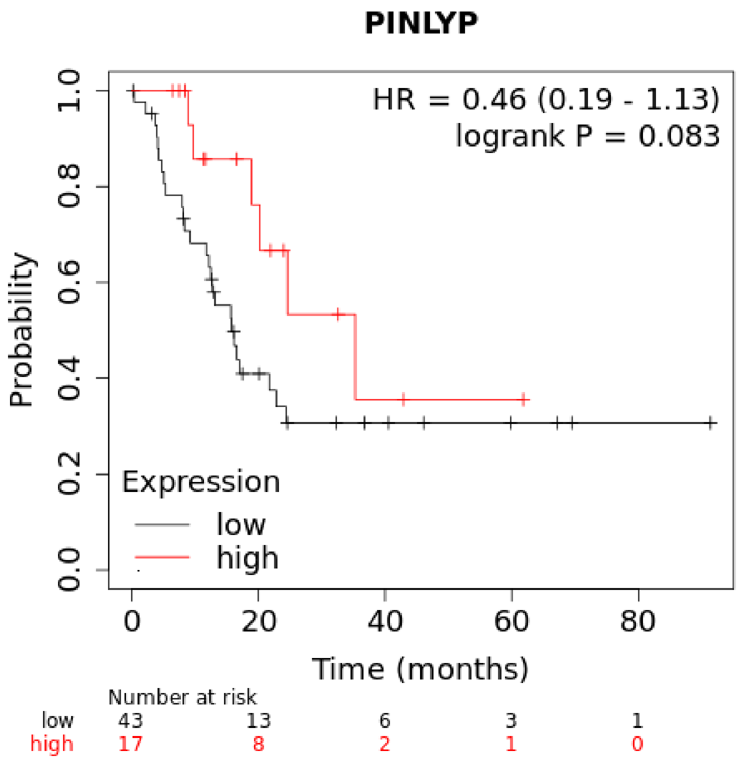

Click here to download the plot in TIFF format

Download plot as a PDF

Download p values vs. cutoff table

Median survival

Low expression cohort (months)	High expression cohort (months)
15.77	35.3

**RNAseq ID:**	PLAUR -
**Survival:**	OS
**Auto select best cutoff:**	checked
**Follow up threshold:**	all
**Censore at threshold:**	checked
**Compute median over entire database:**	false
**Cutoff value used in analysis:**	2003
**Expression range of the probe:**	164 - 14016
**Invert HR values below 1:**	not checked

Restrictions

Tumor type: Pancreatic ductal adenocarcinoma

Restrict analysis to subtypes...

Stage:	all
Gender:	all
Race:	all
Grade:	all
Mutation burden:	all

Restrict analysis based on cellular content...

Basophils:	all
B-cells:	all
CD4+ memory T-cells:	all
CD8+ T-cells:	all
Eosinophils:	all
Macrophages:	all
Mesenchymal stem cells:	all
Natural killer T-cells:	enriched
Regulatory T-cells:	all
Type 1 T-helper cells:	all
Type 2 T-helper cells:	all

Results

P value: 0.0099
FDR: 50%

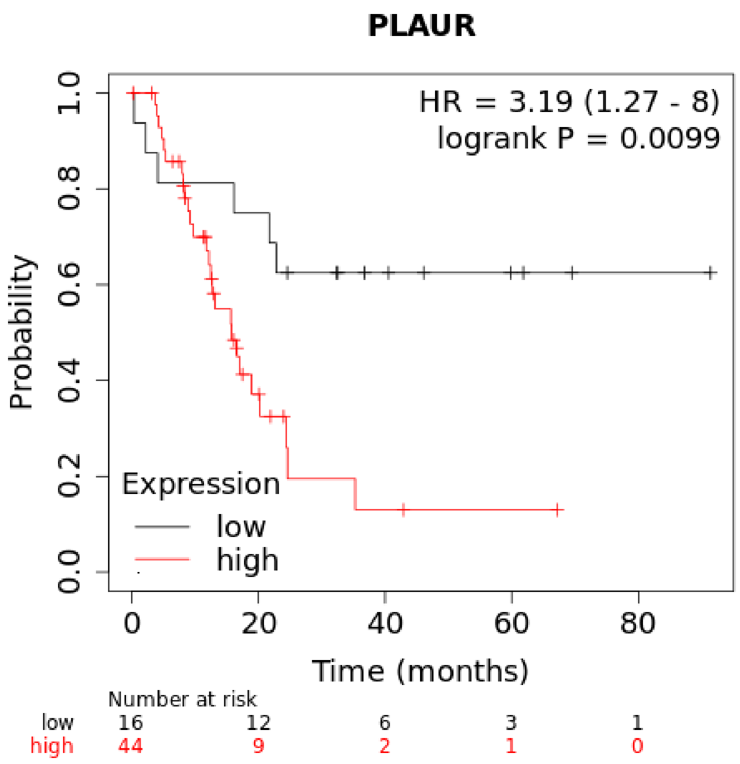

Click here to download the plot in TIFF format

Download plot as a PDF

Download p values vs. cutoff table

Upper quartile survival

Low expression cohort (months)	High expression cohort (months)
21.73	9.23

**RNAseq ID:**	LYPD5 -
**Survival:**	OS
**Auto select best cutoff:**	checked
**Follow up threshold:**	all
**Censore at threshold:**	checked
**Compute median over entire database:**	false
**Cutoff value used in analysis:**	76
**Expression range of the probe:**	5 - 419
**Invert HR values below 1:**	not checked

Restrictions

Tumor type: Pancreatic ductal adenocarcinoma

Restrict analysis to subtypes...

Stage:	all
Gender:	all
Race:	all
Grade:	all
Mutation burden:	all

Restrict analysis based on cellular content...

Basophils:	all

B-cells:	all
CD4+ memory T-cells:	all
CD8+ T-cells:	all
Eosinophils:	all
Macrophages:	all
Mesenchymal stem cells:	all
Natural killer T-cells:	enriched
Regulatory T-cells:	all
Type 1 T-helper cells:	all
Type 2 T-helper cells:	all

Results

P value: 0.0017
FDR: 20%

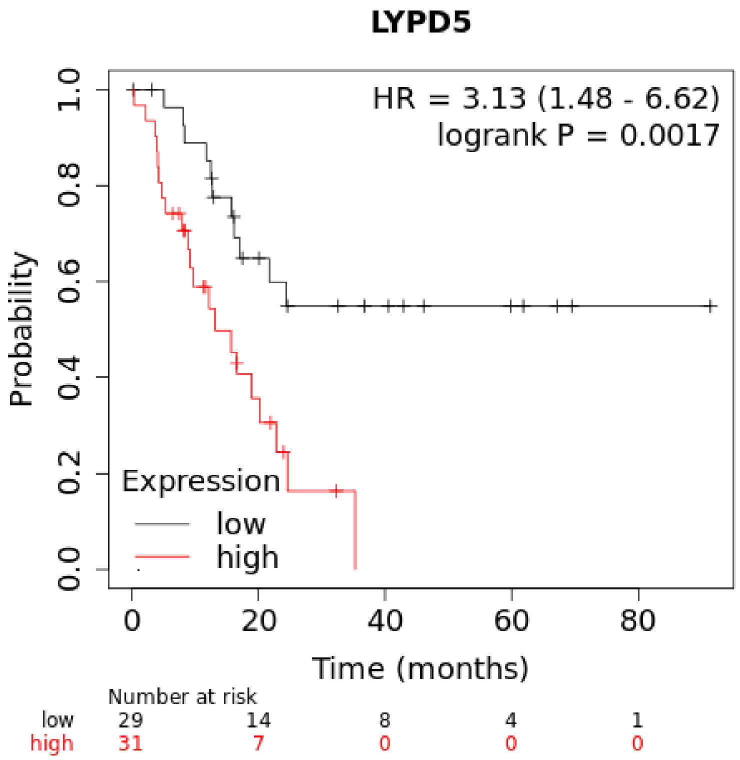

Click here to download the plot in TIFF format

Download plot as a PDF

Download p values vs. cutoff table

Upper quartile survival

Low expression cohort (months)	High expression cohort (months)
15.77	5.33

**RNAseq ID:**	SPACA4 -
**Survival:**	OS
**Auto select best cutoff:**	checked
**Follow up threshold:**	all
**Censore at threshold:**	checked
**Compute median over entire database:**	false
**Cutoff value used in analysis:**	21
**Expression range of the probe:**	0 - 206
**Invert HR values below 1:**	not checked

Restrictions

Tumor type: Pancreatic ductal adenocarcinoma

Restrict analysis to subtypes...

Stage: all
Gender: all
Race: all
Grade: all
Mutation burden: all

Restrict analysis based on cellular content...

Basophils: all
B-cells: all
CD4+ memory T-cells: all
CD8+ T-cells: all
Eosinophils: all
Macrophages: all
Mesenchymal stem cells: all
Natural killer T-cells: enriched
Regulatory T-cells: all
Type 1 T-helper cells: all
Type 2 T-helper cells: all

Results

P value: 0.3332
FDR: 100%

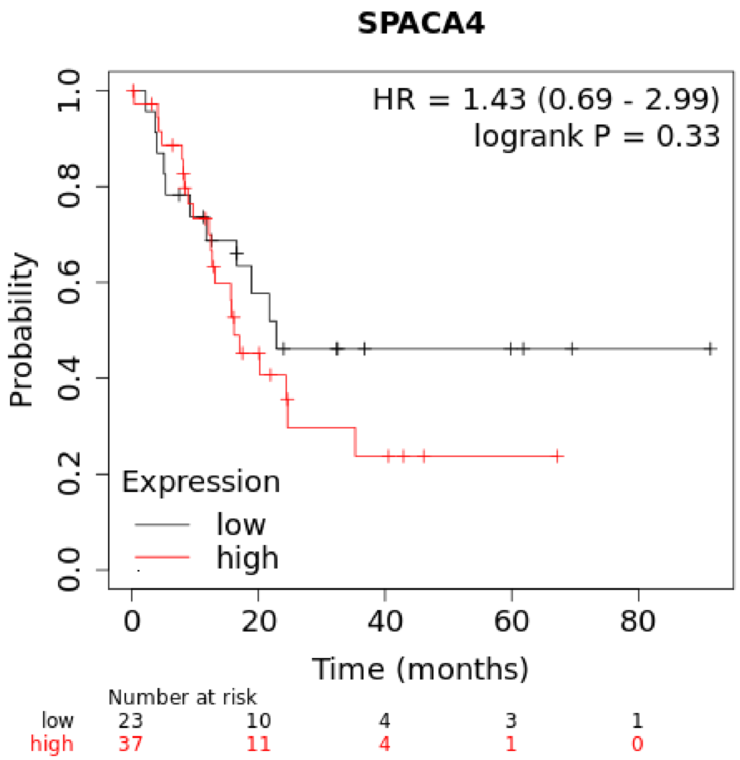

Click here to download the plot in TIFF format

Download plot as a PDF

Download p values vs. cutoff table

Median survival

Low expression cohort (months)	High expression cohort (months)
22.8	16.17

**RNAseq ID:**	ACRV1 -
**Survival:**	OS
**Auto select best cutoff:**	checked
**Follow up threshold:**	all
**Censore at threshold:**	checked
**Compute median over entire database:**	false
**Cutoff value used in analysis:**	4
**Expression range of the probe:**	0 - 26
**Invert HR values below 1:**	not checked

Restrictions

Tumor type: Pancreatic ductal adenocarcinoma

Restrict analysis to subtypes...

Stage:	all
Gender:	all
Race:	all
Grade:	all
Mutation burden:	all

Restrict analysis based on cellular content...

Basophils:	all
B-cells:	all
CD4+ memory T-cells:	all
CD8+ T-cells:	all
Eosinophils:	all
Macrophages:	all
Mesenchymal stem cells:	all
Natural killer T-cells:	enriched
Regulatory T-cells:	all
Type 1 T-helper cells:	all
Type 2 T-helper cells:	all

Results

**P value:**	0.2997
**FDR:**	100%

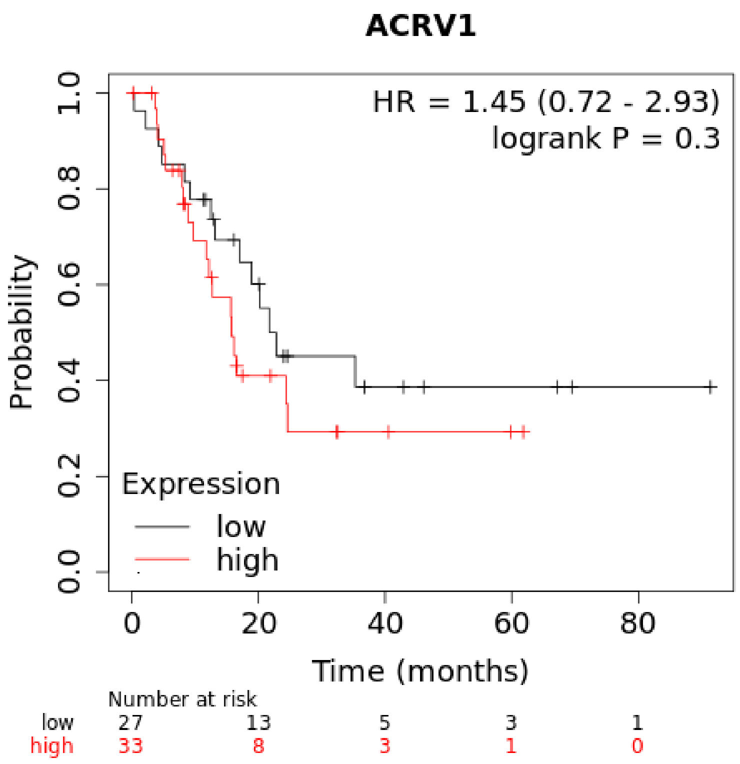

Click here to download the plot in TIFF format

Download plot as a PDF

Download p values vs. cutoff table

Median survival

Low expression cohort (months)	High expression cohort (months)
22.8	15.77

**RNAseq ID:**	PATE1 -
**Survival:**	OS
**Auto select best cutoff:**	checked
**Follow up threshold:**	all
**Censore at threshold:**	checked
**Compute median over entire database:**	false
**Cutoff value used in analysis:**	0
**Expression range of the probe:**	0 - 1
**Invert HR values below 1:**	not checked

Restrictions

Tumor type: Pancreatic ductal adenocarcinoma

Restrict analysis to subtypes...

Stage:	all
Gender:	all
Race:	all
Grade:	all
Mutation burden:	all

Restrict analysis based on cellular content...

Basophils:	all

B-cells:	all
CD4+ memory T-cells:	all
CD8+ T-cells:	all
Eosinophils:	all
Macrophages:	all
Mesenchymal stem cells:	all
Natural killer T-cells:	enriched
Regulatory T-cells:	all
Type 1 T-helper cells:	all
Type 2 T-helper cells:	all

Results

P value: 0.087
FDR: 100%

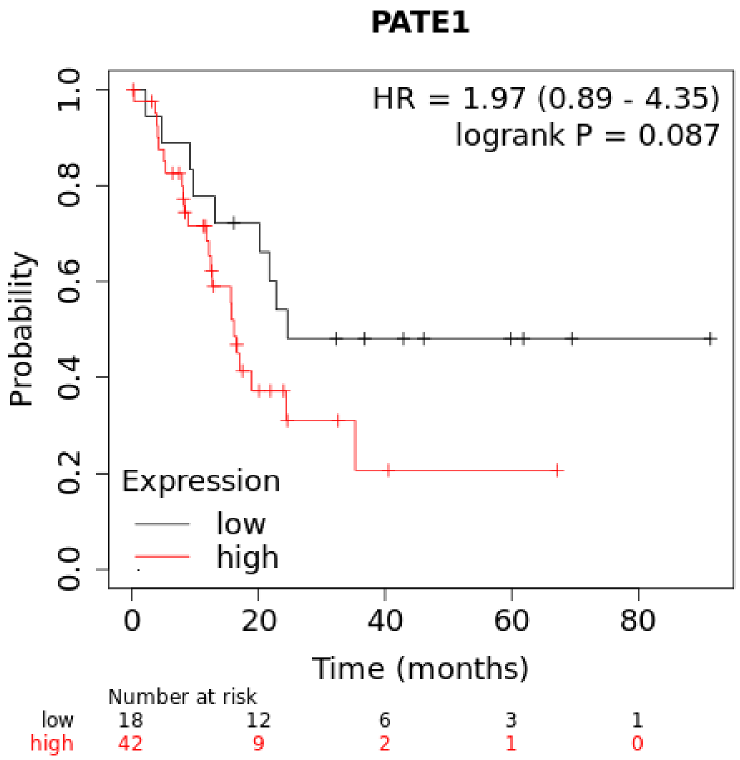

Click here to download the plot in TIFF format

Download plot as a PDF

Download p values vs. cutoff table

Median survival

Low expression cohort (months)	High expression cohort (months)
24.6	16.17

**RNAseq ID:**	PATE2 -
**Survival:**	OS
**Auto select best cutoff:**	checked
**Follow up threshold:**	all
**Censore at threshold:**	checked
**Compute median over entire database:**	false
**Cutoff value used in analysis:**	1
**Expression range of the probe:**	0 - 2
**Invert HR values below 1:**	not checked

Restrictions

Tumor type: Pancreatic ductal adenocarcinoma

Restrict analysis to subtypes...

Stage:	all
Gender:	all
Race:	all
Grade:	all
Mutation burden:	all

Restrict analysis based on cellular content...

Basophils:	all
B-cells:	all
CD4+ memory T-cells:	all
CD8+ T-cells:	all
Eosinophils:	all
Macrophages:	all
Mesenchymal stem cells:	all
Natural killer T-cells:	enriched
Regulatory T-cells:	all
Type 1 T-helper cells:	all
Type 2 T-helper cells:	all

Results

P value: 0.0163
FDR: over 50%

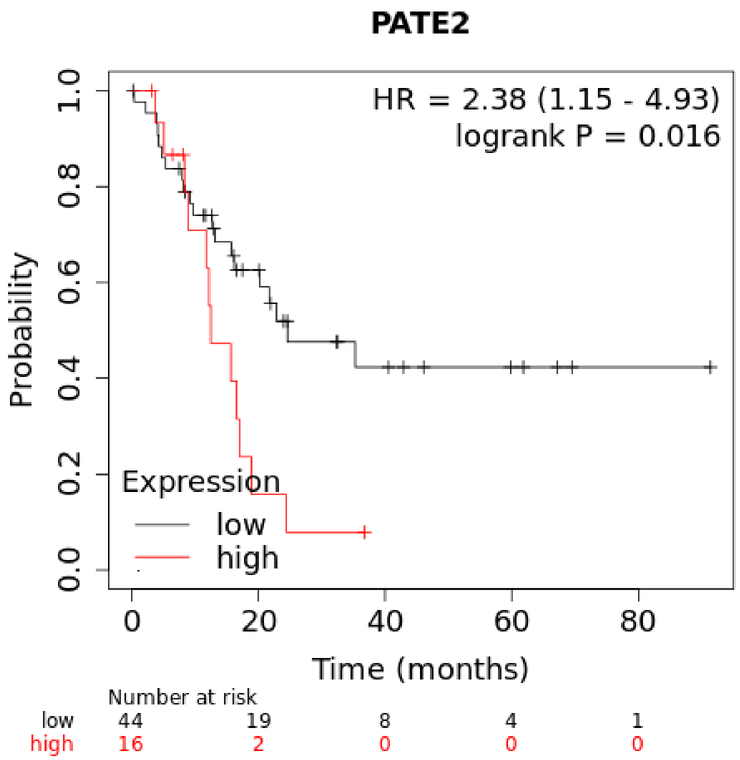

Click here to download the plot in TIFF format

Download plot as a PDF

Download p values vs. cutoff table

Median survival

Low expression cohort (months)	High expression cohort (months)
24.6	12.5

**RNAseq ID:**	PATE3 -
**Survival:**	OS
**Auto select best cutoff:**	checked
**Follow up threshold:**	all
**Censore at threshold:**	checked
**Compute median over entire database:**	false
**Cutoff value used in analysis:**	0
**Expression range of the probe:**	0 - 1
**Invert HR values below 1:**	not checked

Restrictions

Tumor type: Pancreatic ductal adenocarcinoma

Restrict analysis to subtypes...

Stage:	all
Gender:	all
Race:	all
Grade:	all
Mutation burden:	all

Restrict analysis based on cellular content...

Basophils:	all
B-cells:	all
CD4+ memory T-cells:	all
CD8+ T-cells:	all
Eosinophils:	all
Macrophages:	all
Mesenchymal stem cells:	all
Natural killer T-cells:	enriched
Regulatory T-cells:	all
Type 1 T-helper cells:	all
Type 2 T-helper cells:	all

Results

**P value:**	0.0686
**FDR:**	100%

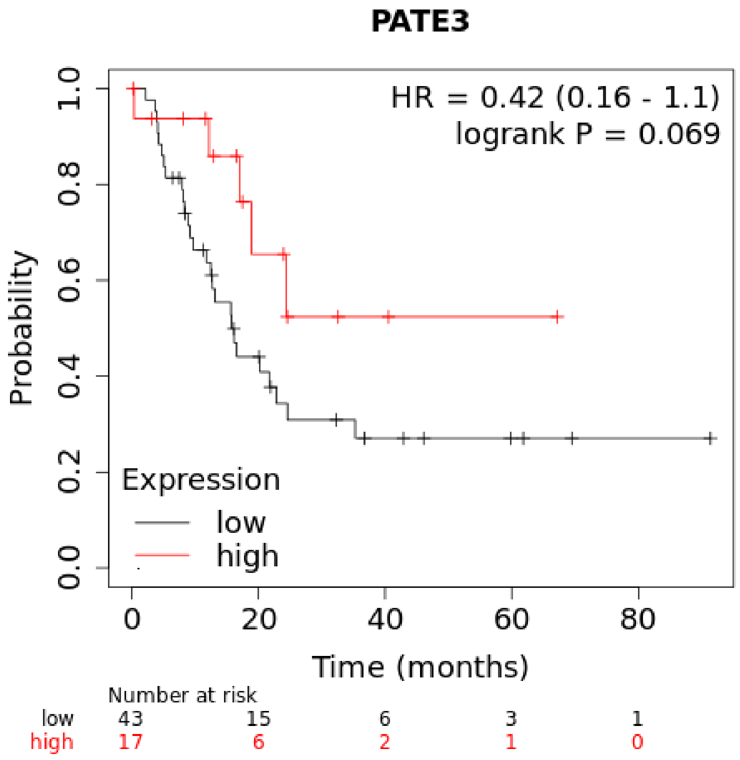

[Click here to download the plot in TIFF format]

[Download plot as a PDF]

[Download p values vs. cutoff table]

Upper quartile survival

Low expression cohort (months)	High expression cohort (months)
8.33	18.93

**RNAseq ID:**	PATE4 -
**Survival:**	OS
**Auto select best cutoff:**	checked
**Follow up threshold:**	all
**Censore at threshold:**	checked
**Compute median over entire database:**	false
**Cutoff value used in analysis:**	0
**Expression range of the probe:**	0 - 3
**Invert HR values below 1:**	not checked

Restrictions

Tumor type: Pancreatic ductal adenocarcinoma

Restrict analysis to subtypes...

Stage:	all
Gender:	all
Race:	all
Grade:	all
Mutation burden:	all

Restrict analysis based on cellular content...

Basophils:	all

B-cells:	all
CD4+ memory T-cells:	all
CD8+ T-cells:	all
Eosinophils:	all
Macrophages:	all
Mesenchymal stem cells:	all
Natural killer T-cells:	enriched
Regulatory T-cells:	all
Type 1 T-helper cells:	all
Type 2 T-helper cells:	all

Results

P value: 0.007
FDR: 50%

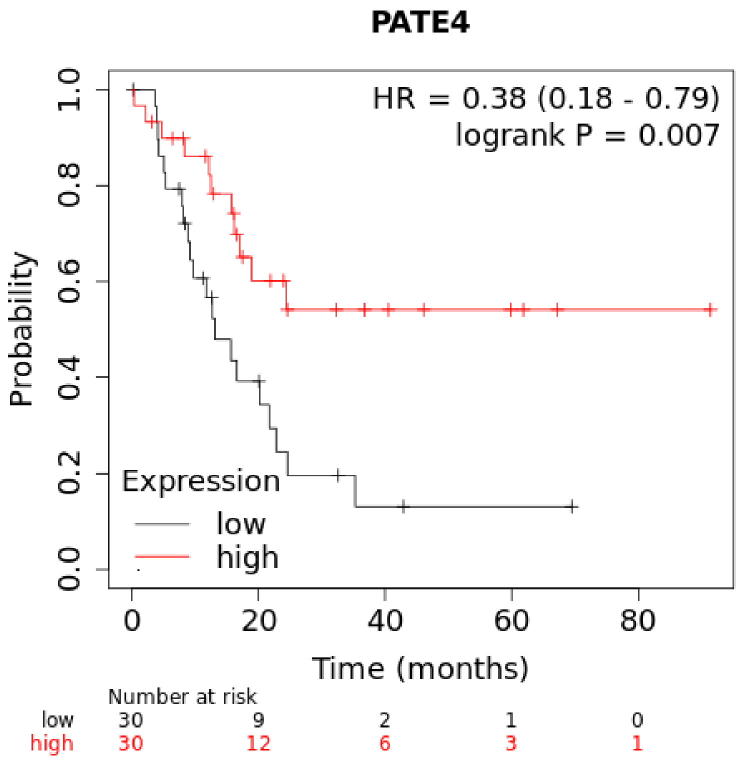

Click here to download the plot in TIFF format

Download plot as a PDF

Download p values vs. cutoff table

Upper quartile survival

Low expression cohort (months)	High expression cohort (months)
8.13	15.77

**RNAseq ID:**	CD59 -
**Survival:**	OS
**Auto select best cutoff:**	checked
**Follow up threshold:**	all
**Censore at threshold:**	checked
**Compute median over entire database:**	false
**Cutoff value used in analysis:**	17604
**Expression range of the probe:**	2610 - 39336
**Invert HR values below 1:**	not checked

Restrictions

Tumor type: Pancreatic ductal adenocarcinoma

Restrict analysis to subtypes...

Stage: all
Gender: all
Race: all
Grade: all
Mutation burden: all

Restrict analysis based on cellular content...

Basophils: all
B-cells: all
CD4+ memory T-cells: all
CD8+ T-cells: all
Eosinophils: all
Macrophages: all
Mesenchymal stem cells: all
Natural killer T-cells: enriched
Regulatory T-cells: all
Type 1 T-helper cells: all
Type 2 T-helper cells: all

Results

P value: 0.0075
FDR: over 50%

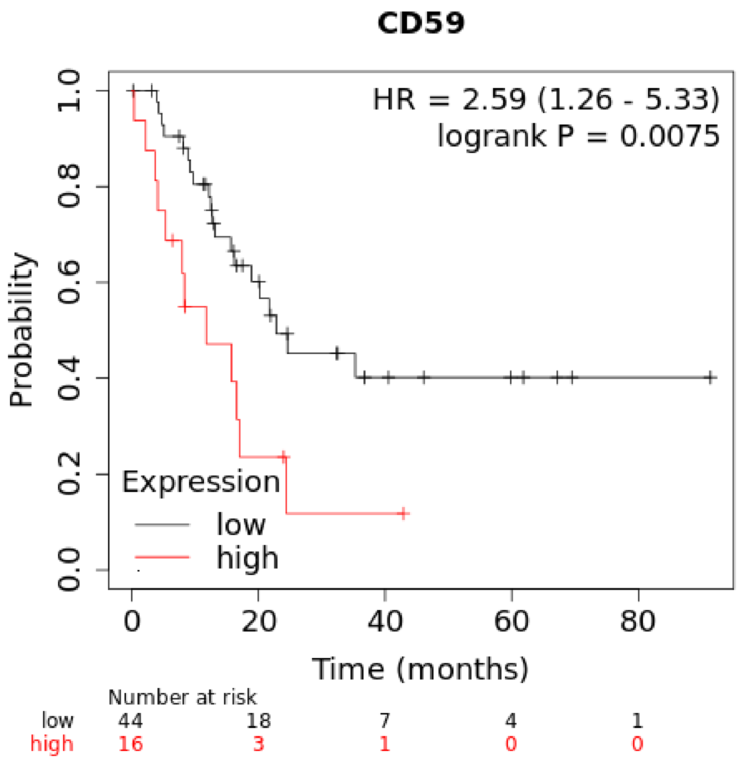

Click here to download the plot in TIFF format

Download plot as a PDF

Download p values vs. cutoff table

Median survival

Low expression cohort (months)	High expression cohort (months)
22.8	11.77

**RNAseq ID:**	LY6G6C -
**Survival:**	OS
**Auto select best cutoff:**	checked
**Follow up threshold:**	all
**Censore at threshold:**	checked
**Compute median over entire database:**	false
**Cutoff value used in analysis:**	13
**Expression range of the probe:**	0 - 142
**Invert HR values below 1:**	not checked

Restrictions

Tumor type: Pancreatic ductal adenocarcinoma

Restrict analysis to subtypes...

Stage:	all
Gender:	all
Race:	all
Grade:	all
Mutation burden:	all

Restrict analysis based on cellular content...

Basophils:	all
B-cells:	all
CD4+ memory T-cells:	all
CD8+ T-cells:	all
Eosinophils:	all
Macrophages:	all
Mesenchymal stem cells:	all
Natural killer T-cells:	enriched
Regulatory T-cells:	all
Type 1 T-helper cells:	all
Type 2 T-helper cells:	all

Results

**P value:**	0.0148
**FDR:**	50%

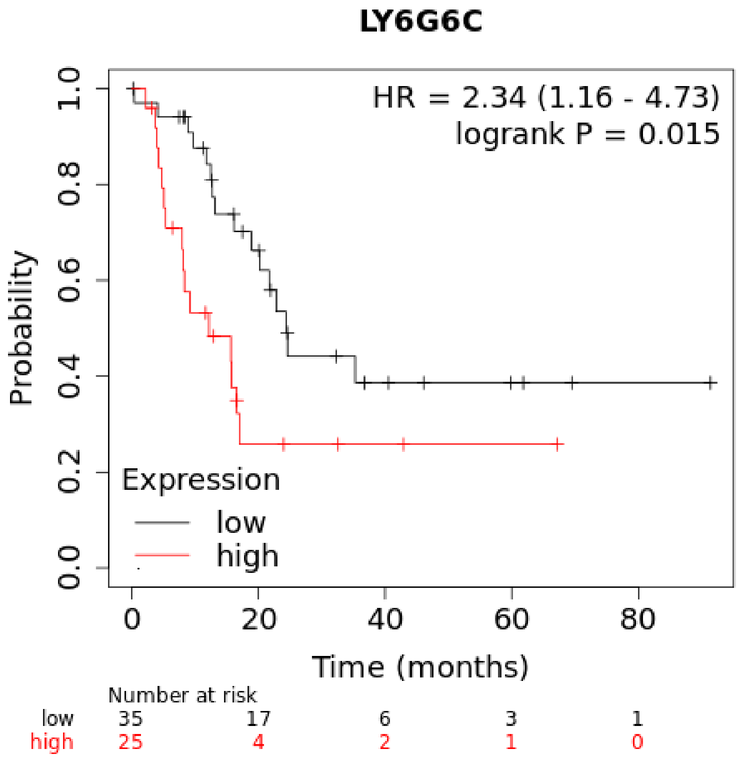

[Click here to download the plot in TIFF format]

[Download plot as a PDF]

[Download p values vs. cutoff table]

Median survival

Low expression cohort (months)	High expression cohort (months)
24.4	12.2

**RNAseq ID:**	LY6G6D -
**Survival:**	OS
**Auto select best cutoff:**	checked
**Follow up threshold:**	all
**Censore at threshold:**	checked
**Compute median over entire database:**	false
**Cutoff value used in analysis:**	0
**Expression range of the probe:**	0 - 1
**Invert HR values below 1:**	not checked

Restrictions

Tumor type: Pancreatic ductal adenocarcinoma

Restrict analysis to subtypes...

Stage:	all
Gender:	all
Race:	all
Grade:	all
Mutation burden:	all

Restrict analysis based on cellular content...

Basophils: all

B-cells:	all
CD4+ memory T-cells:	all
CD8+ T-cells:	all
Eosinophils:	all
Macrophages:	all
Mesenchymal stem cells:	all
Natural killer T-cells:	enriched
Regulatory T-cells:	all
Type 1 T-helper cells:	all
Type 2 T-helper cells:	all

Results

P value: 0.2869
FDR: 100%

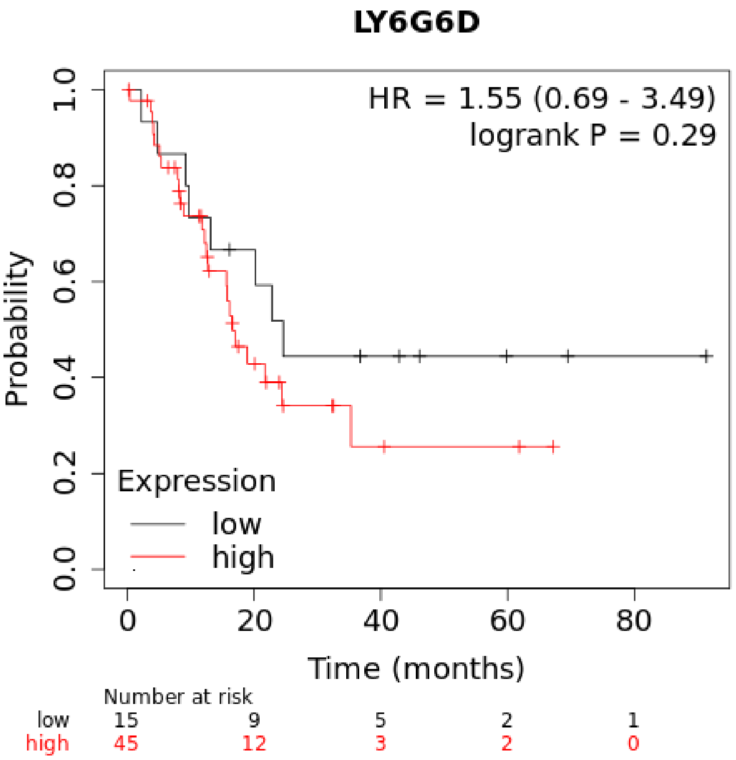

Click here to download the plot in TIFF format

Download plot as a PDF

Download p values vs. cutoff table

Median survival

Low expression cohort (months)	High expression cohort (months)
24.6	16.6

**RNAseq ID:**	LY6G6F -
**Survival:**	OS
**Auto select best cutoff:**	checked
**Follow up threshold:**	all
**Censore at threshold:**	checked
**Compute median over entire database:**	false
**Cutoff value used in analysis:**	0
**Expression range of the probe:**	0 - 1
**Invert HR values below 1:**	not checked

Restrictions

Tumor type: Pancreatic ductal adenocarcinoma

Restrict analysis to subtypes...

Stage:	all
Gender:	all
Race:	all
Grade:	all
Mutation burden:	all

Restrict analysis based on cellular content...

Basophils:	all
B-cells:	all
CD4+ memory T-cells:	all
CD8+ T-cells:	all
Eosinophils:	all
Macrophages:	all
Mesenchymal stem cells:	all
Natural killer T-cells:	enriched
Regulatory T-cells:	all
Type 1 T-helper cells:	all
Type 2 T-helper cells:	all

Results

P value: 0.0165
FDR: over 50%

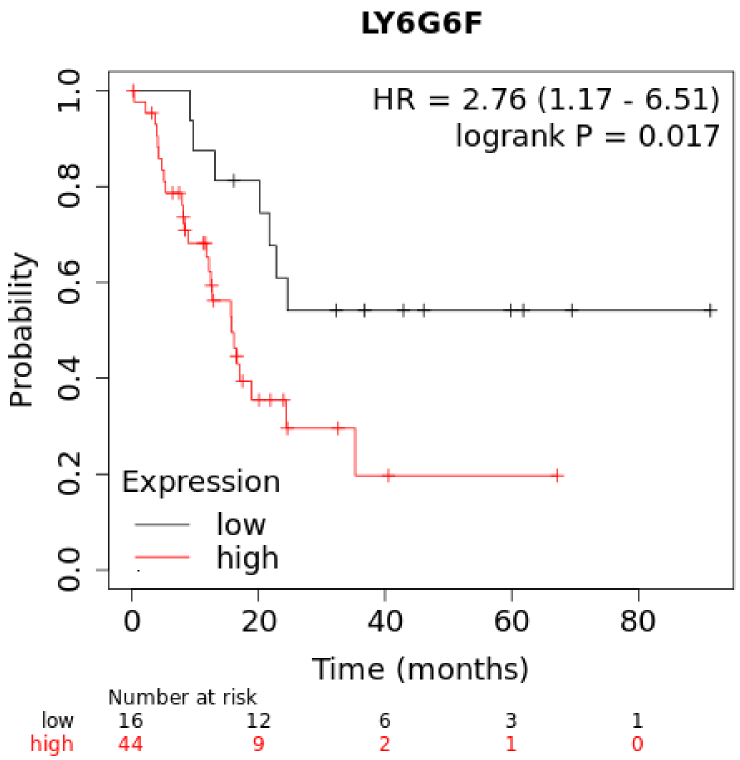

Click here to download the plot in TIFF format

Download plot as a PDF

Download p values vs. cutoff table

Upper quartile survival

Low expression cohort (months)	High expression cohort (months)
20.23	8.13

**RNAseq ID:**	LY6G5C -
**Survival:**	OS
**Auto select best cutoff:**	checked
**Follow up threshold:**	all
**Censore at threshold:**	checked
**Compute median over entire database:**	false
**Cutoff value used in analysis:**	52
**Expression range of the probe:**	15 - 586
**Invert HR values below 1:**	not checked

Restrictions

Tumor type: Pancreatic ductal adenocarcinoma

Restrict analysis to subtypes...

Stage:	all
Gender:	all
Race:	all
Grade:	all
Mutation burden:	all

Restrict analysis based on cellular content...

Basophils:	all
B-cells:	all
CD4+ memory T-cells:	all
CD8+ T-cells:	all
Eosinophils:	all
Macrophages:	all
Mesenchymal stem cells:	all
Natural killer T-cells:	enriched
Regulatory T-cells:	all
Type 1 T-helper cells:	all
Type 2 T-helper cells:	all

Results

**P value:**	0.0003
**FDR:**	2%

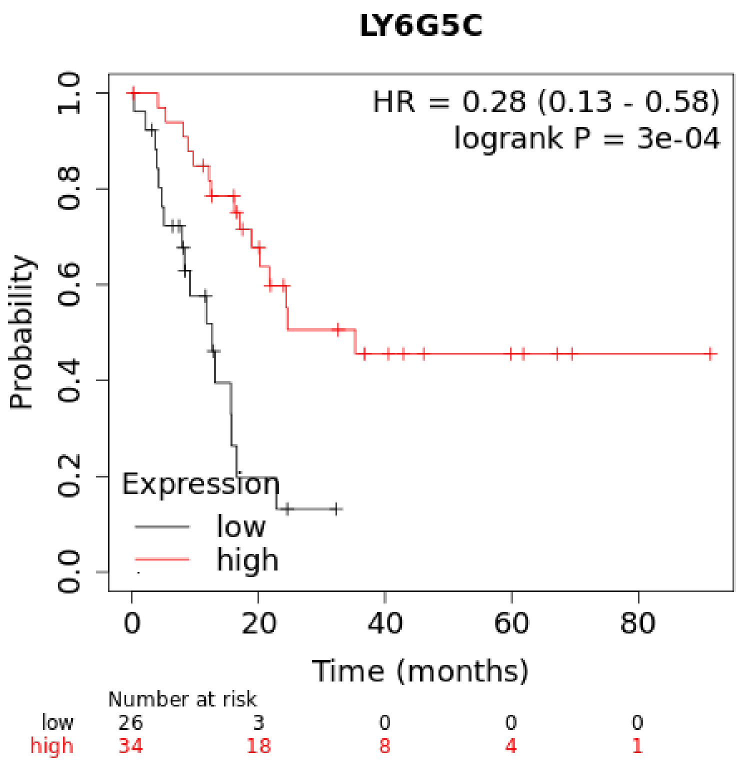

Click here to download the plot in TIFF format

Download plot as a PDF

Download p values vs. cutoff table

Median survival

Low expression cohort (months)	High expression cohort (months)
12.7	35.3

**RNAseq ID:**	LY6G5B -
**Survival:**	OS
**Auto select best cutoff:**	checked
**Follow up threshold:**	all
**Censore at threshold:**	checked
**Compute median over entire database:**	false
**Cutoff value used in analysis:**	51
**Expression range of the probe:**	4 - 192
**Invert HR values below 1:**	not checked

Restrictions

Tumor type: Pancreatic ductal adenocarcinoma

Restrict analysis to subtypes...

Stage:	all
Gender:	all
Race:	all
Grade:	all
Mutation burden:	all

Restrict analysis based on cellular content...

Basophils:	all

B-cells:	all
CD4+ memory T-cells:	all
CD8+ T-cells:	all
Eosinophils:	all
Macrophages:	all
Mesenchymal stem cells:	all
Natural killer T-cells:	enriched
Regulatory T-cells:	all
Type 1 T-helper cells:	all
Type 2 T-helper cells:	all

Results

P value: 0.0143
FDR: over 50%

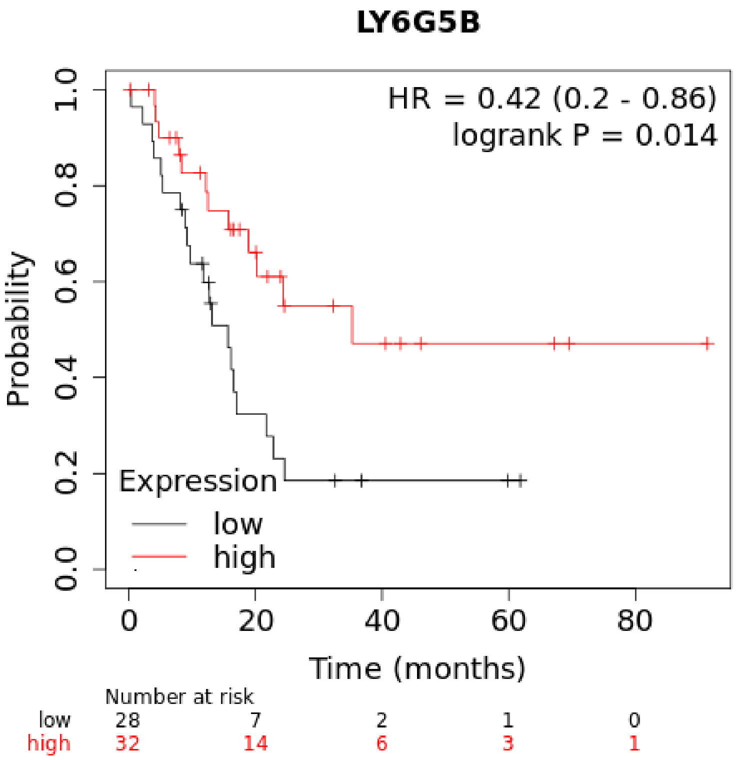

Click here to download the plot in TIFF format

Download plot as a PDF

Download p values vs. cutoff table

Median survival

Low expression cohort (months)	High expression cohort (months)
15.67	35.3

You can save the plots by right-clicking the image and then selecting "Save image as...". To generate a high resolution TIFF image, please adjust the "Settings" in the analysis page.

KMplot.com Copyright © 2009-2020 | Contact | Sitemap

Pan-cancer

KM plotter | Home | Vote | Download | Updates | Contact

The desired RNAseq ID is valid: PSCA (-), LY6K (-), SLURP1 (-), LYPD2 (-), LY6D (-), GML (-), LY6E (-), LY6L (-), LY6H (-), GPIHBP1 (-), LYPD4 (-), CD177 (-), TEX101 (-), LYPD3 (-), PINLYP (-), PLAUR (-), LYPD5 (-), SPACA4 (-), ACRV1 (-), PATE1 (-), PATE2 (-), PATE3 (-), PATE4 (-), CD59 (-), LY6G6C (-), LY6G6D (-), LY6G6F (-), LY6G5C (-), LY6G5B (-),

**RNAseq ID:**	PSCA -
**Survival:**	OS
**Auto select best cutoff:**	checked
**Follow up threshold:**	all
**Censore at threshold:**	checked
**Compute median over entire database:**	false
**Cutoff value used in analysis:**	506
**Expression range of the probe:**	1 - 65661
**Invert HR values below 1:**	not checked

Restrictions

Tumor type: Pancreatic ductal adenocarcinoma

Restrict analysis to subtypes...

Stage:	all
Gender:	all
Race:	all
Grade:	all
Mutation burden:	all

Restrict analysis based on cellular content...

Basophils:	all
B-cells:	all
CD4+ memory T-cells:	all
CD8+ T-cells:	all
Eosinophils:	all
Macrophages:	all
Mesenchymal stem cells:	all
Natural killer T-cells:	decreased
Regulatory T-cells:	all
Type 1 T-helper cells:	all
Type 2 T-helper cells:	all

Results

**P value:**	0.0026
**FDR:**	50%

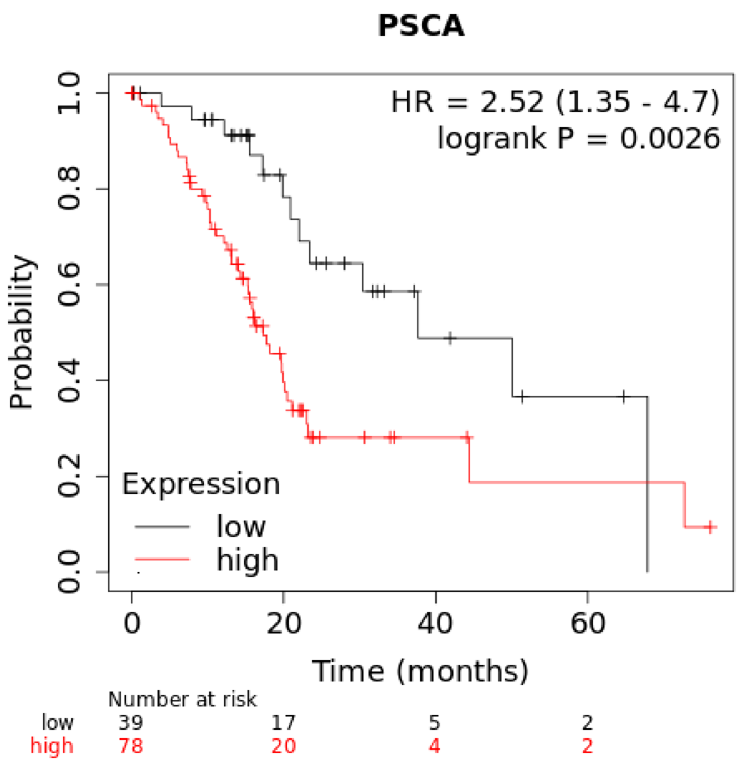

Click here to download the plot in TIFF format

Download plot as a PDF

Download p values vs. cutoff table

Median survival

Low expression cohort (months)	High expression cohort (months)
37.67	17.27

**RNAseq ID:**	LY6K	-
**Survival:**	OS	
**Auto select best cutoff:**	checked	
**Follow up threshold:**	all	
**Censore at threshold:**	checked	
**Compute median over entire database:**	false	
**Cutoff value used in analysis:**	3	
**Expression range of the probe:**	0 - 1332	
**Invert HR values below 1:**	not checked	

Restrictions

Tumor type: Pancreatic ductal adenocarcinoma

Restrict analysis to subtypes...

Stage:	all
Gender:	all
Race:	all
Grade:	all
Mutation burden:	all

Restrict analysis based on cellular content...

Basophils:	all

B-cells:	all
CD4+ memory T-cells:	all
CD8+ T-cells:	all
Eosinophils:	all
Macrophages:	all
Mesenchymal stem cells:	all
Natural killer T-cells:	decreased
Regulatory T-cells:	all
Type 1 T-helper cells:	all
Type 2 T-helper cells:	all

Results

P value: 0.3954
FDR: 100%

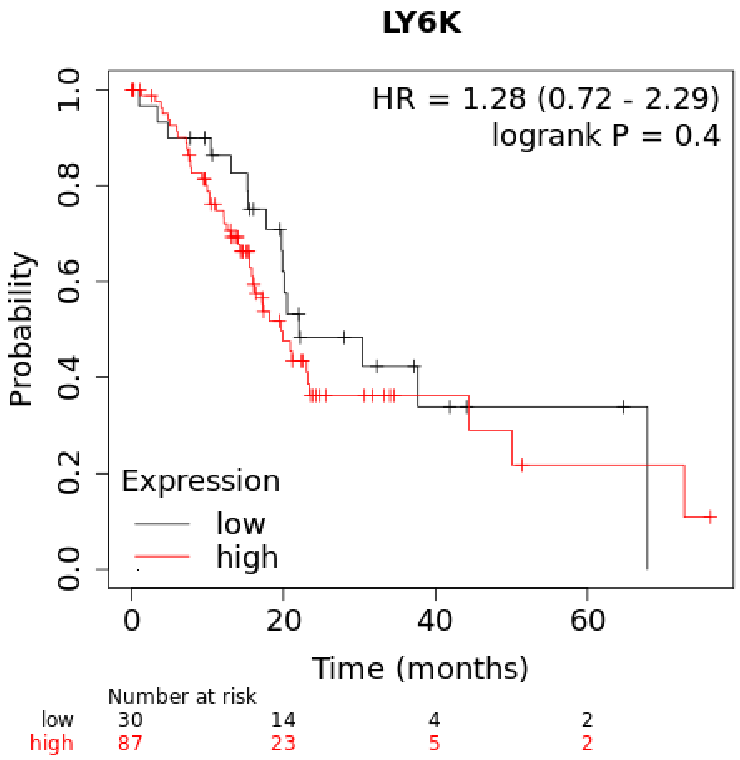

Click here to download the plot in TIFF format

Download plot as a PDF

Download p values vs. cutoff table

Median survival

Low expression cohort (months)	High expression cohort (months)
22.03	19.73

**RNAseq ID:**	SLURP1 -
**Survival:**	OS
**Auto select best cutoff:**	checked
**Follow up threshold:**	all
**Censore at threshold:**	checked
**Compute median over entire database:**	false
**Cutoff value used in analysis:**	1
**Expression range of the probe:**	0 - 150
**Invert HR values below 1:**	not checked

Restrictions

Tumor type: Pancreatic ductal adenocarcinoma

Restrict analysis to subtypes...

Stage:	all
Gender:	all
Race:	all
Grade:	all
Mutation burden:	all

Restrict analysis based on cellular content...

Basophils:	all
B-cells:	all
CD4+ memory T-cells:	all
CD8+ T-cells:	all
Eosinophils:	all
Macrophages:	all
Mesenchymal stem cells:	all
Natural killer T-cells:	decreased
Regulatory T-cells:	all
Type 1 T-helper cells:	all
Type 2 T-helper cells:	all

Results

P value: 0.0033
FDR: over 50%

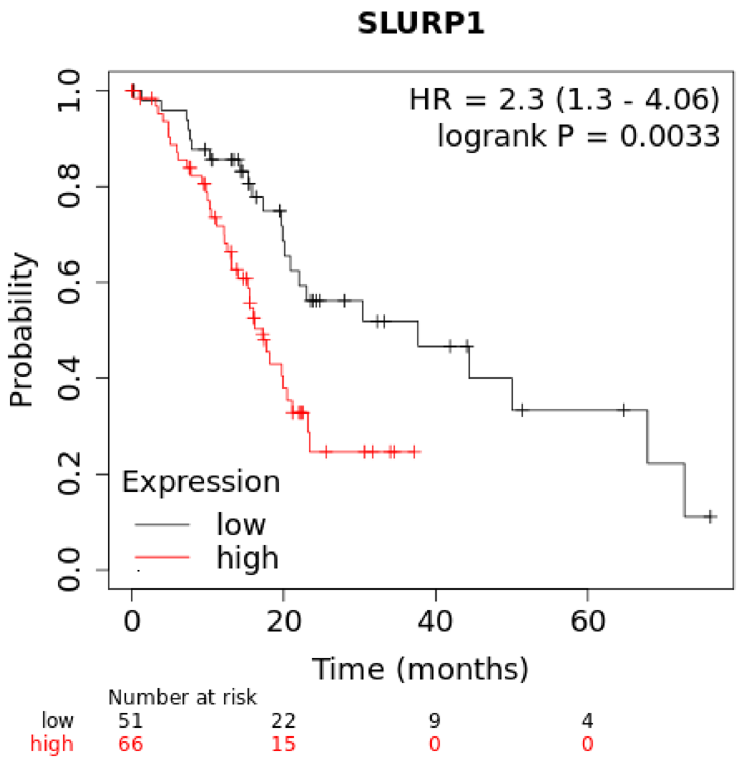

Click here to download the plot in TIFF format

Download plot as a PDF

Download p values vs. cutoff table

Median survival

Low expression cohort (months)	High expression cohort (months)
37.67	17.23

**RNAseq ID:**	LYPD2 -
**Survival:**	OS
**Auto select best cutoff:**	checked
**Follow up threshold:**	all
**Censore at threshold:**	checked
**Compute median over entire database:**	false
**Cutoff value used in analysis:**	4
**Expression range of the probe:**	0 - 4748
**Invert HR values below 1:**	not checked

Restrictions

Tumor type: Pancreatic ductal adenocarcinoma

Restrict analysis to subtypes...

Stage:	all
Gender:	all
Race:	all
Grade:	all
Mutation burden:	all

Restrict analysis based on cellular content...

Basophils:	all
B-cells:	all
CD4+ memory T-cells:	all
CD8+ T-cells:	all
Eosinophils:	all
Macrophages:	all
Mesenchymal stem cells:	all
Natural killer T-cells:	decreased
Regulatory T-cells:	all
Type 1 T-helper cells:	all
Type 2 T-helper cells:	all

Results

**P value:**	0.2594
**FDR:**	100%

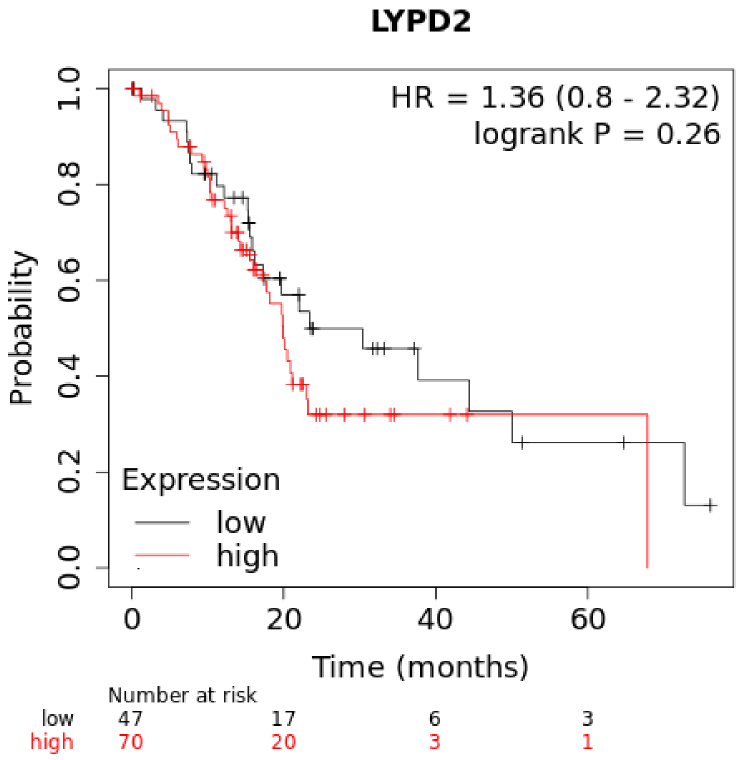

[Click here to download the plot in TIFF format]

[Download plot as a PDF]

[Download p values vs. cutoff table]

Median survival

Low expression cohort (months)	High expression cohort (months)
23.4	19.93

**RNAseq ID:**	LY6D -
**Survival:**	OS
**Auto select best cutoff:**	checked
**Follow up threshold:**	all
**Censore at threshold:**	checked
**Compute median over entire database:**	false
**Cutoff value used in analysis:**	160
**Expression range of the probe:**	0 - 6226
**Invert HR values below 1:**	not checked

Restrictions

Tumor type: Pancreatic ductal adenocarcinoma

Restrict analysis to subtypes...

Stage:	all
Gender:	all
Race:	all
Grade:	all
Mutation burden:	all

Restrict analysis based on cellular content...

Basophils:	all

B-cells:	all
CD4+ memory T-cells:	all
CD8+ T-cells:	all
Eosinophils:	all
Macrophages:	all
Mesenchymal stem cells:	all
Natural killer T-cells:	decreased
Regulatory T-cells:	all
Type 1 T-helper cells:	all
Type 2 T-helper cells:	all

Results

P value: 0.001
FDR: 20%

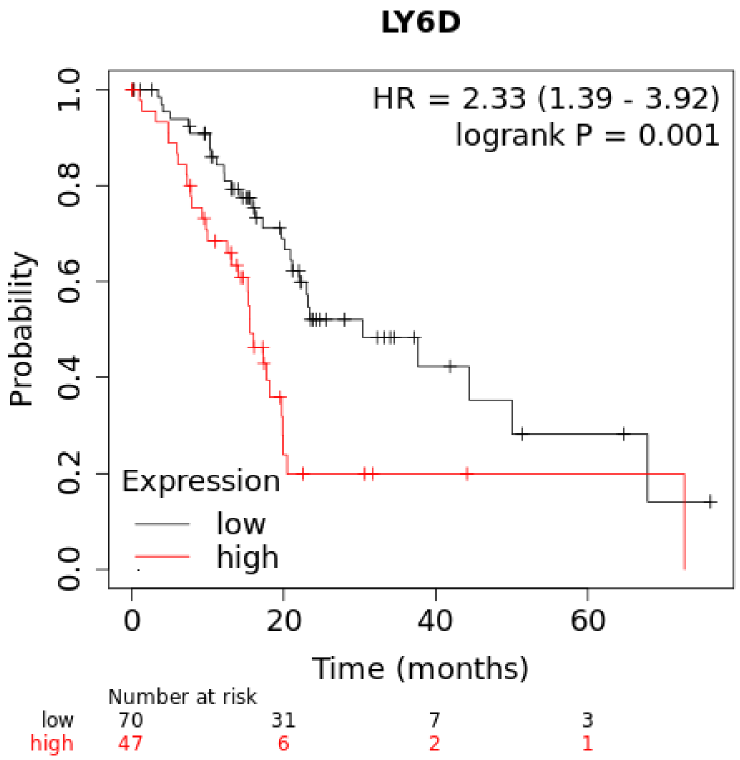

Click here to download the plot in TIFF format

Download plot as a PDF

Download p values vs. cutoff table

Median survival

Low expression cohort (months)	High expression cohort (months)
30.43	15.57

**RNAseq ID:**	GML -
**Survival:**	OS
**Auto select best cutoff:**	checked
**Follow up threshold:**	all
**Censore at threshold:**	checked
**Compute median over entire database:**	false
**Cutoff value used in analysis:**	0
**Expression range of the probe:**	0 - 3
**Invert HR values below 1:**	not checked

Restrictions

Tumor type: Pancreatic ductal adenocarcinoma

Restrict analysis to subtypes...

Stage: all
Gender: all
Race: all
Grade: all
Mutation burden: all

Restrict analysis based on cellular content...

Basophils: all
B-cells: all
CD4+ memory T-cells: all
CD8+ T-cells: all
Eosinophils: all
Macrophages: all
Mesenchymal stem cells: all
Natural killer T-cells: decreased
Regulatory T-cells: all
Type 1 T-helper cells: all
Type 2 T-helper cells: all

Results

P value: 0.0822
FDR: 100%

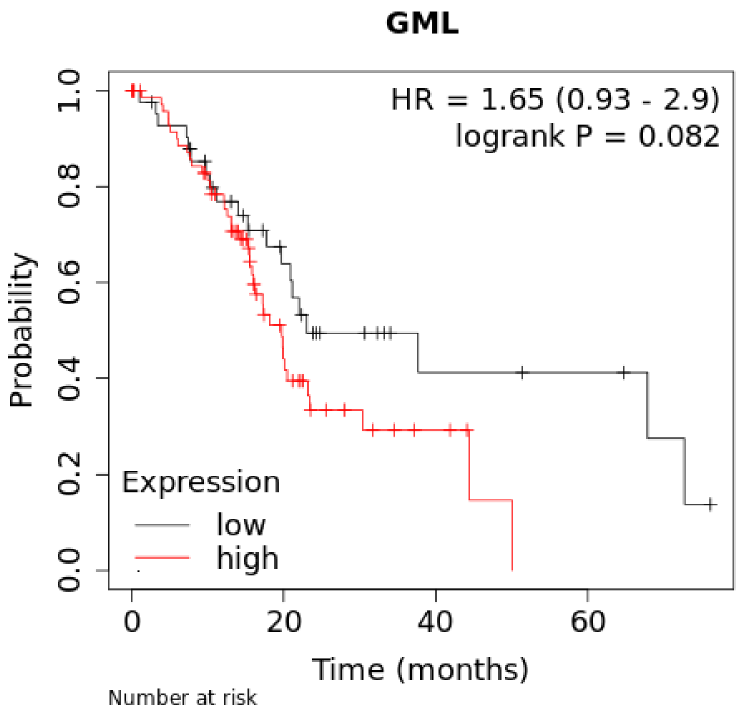

Number at risk

low	42	18	5	4
high	75	19	4	0

Click here to download the plot in TIFF format

Download plot as a PDF

Download p values vs. cutoff table

Median survival

Low expression cohort (months)	High expression cohort (months)
23.03	19.77

**RNAseq ID:**	LY6E -
**Survival:**	OS
**Auto select best cutoff:**	checked
**Follow up threshold:**	all
**Censore at threshold:**	checked
**Compute median over entire database:**	false
**Cutoff value used in analysis:**	10634
**Expression range of the probe:**	254 - 52613
**Invert HR values below 1:**	not checked

Restrictions

Tumor type: Pancreatic ductal adenocarcinoma

Restrict analysis to subtypes...

Stage:	all
Gender:	all
Race:	all
Grade:	all
Mutation burden:	all

Restrict analysis based on cellular content...

Basophils:	all
B-cells:	all
CD4+ memory T-cells:	all
CD8+ T-cells:	all
Eosinophils:	all
Macrophages:	all
Mesenchymal stem cells:	all
Natural killer T-cells:	decreased
Regulatory T-cells:	all
Type 1 T-helper cells:	all
Type 2 T-helper cells:	all

Results

**P value:**	0.007
**FDR:**	over 50%

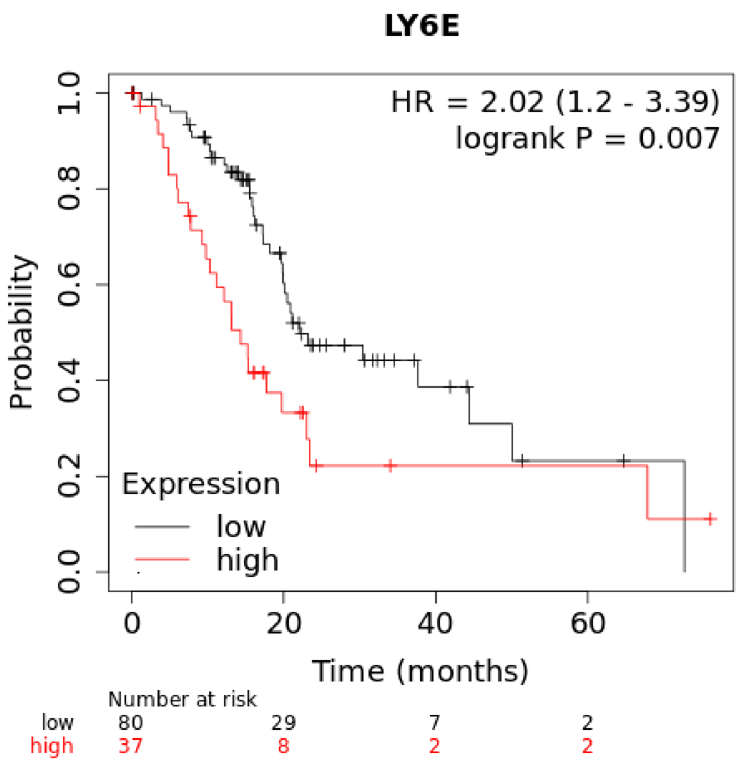

Click here to download the plot in TIFF format

Download plot as a PDF

Download p values vs. cutoff table

Median survival

Low expression cohort (months)	High expression cohort (months)
22.03	14.33

RNAseq ID: LY6L -
Survival: OS
Auto select best cutoff: checked
Follow up threshold: all
Censore at threshold: checked
Compute median over entire database: false
Cutoff value used in analysis: 0
Expression range of the probe: 0 - 8
Invert HR values below 1: not checked

Restrictions

Tumor type: Pancreatic ductal adenocarcinoma

Restrict analysis to subtypes...

Stage: all
Gender: all
Race: all
Grade: all
Mutation burden: all

Restrict analysis based on cellular content...

Basophils: all

B-cells:	all
CD4+ memory T-cells:	all
CD8+ T-cells:	all
Eosinophils:	all
Macrophages:	all
Mesenchymal stem cells:	all
Natural killer T-cells:	decreased
Regulatory T-cells:	all
Type 1 T-helper cells:	all
Type 2 T-helper cells:	all

Results

P value: 0.0781
FDR: 100%

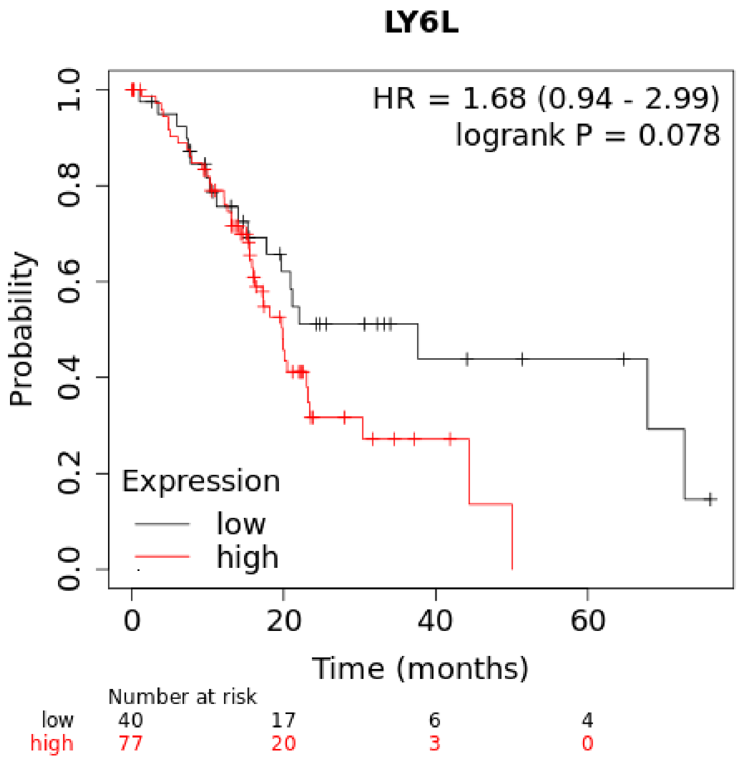

Click here to download the plot in TIFF format

Download plot as a PDF

Download p values vs. cutoff table

Median survival

Low expression cohort (months)	High expression cohort (months)
37.67	19.87

**RNAseq ID:**	LY6H -
**Survival:**	OS
**Auto select best cutoff:**	checked
**Follow up threshold:**	all
**Censore at threshold:**	checked
**Compute median over entire database:**	false
**Cutoff value used in analysis:**	21
**Expression range of the probe:**	1 - 6634
**Invert HR values below 1:**	not checked

Restrictions

Tumor type: Pancreatic ductal adenocarcinoma

Restrict analysis to subtypes...

Stage:	all
Gender:	all
Race:	all
Grade:	all
Mutation burden:	all

Restrict analysis based on cellular content...

Basophils:	all
B-cells:	all
CD4+ memory T-cells:	all
CD8+ T-cells:	all
Eosinophils:	all
Macrophages:	all
Mesenchymal stem cells:	all
Natural killer T-cells:	decreased
Regulatory T-cells:	all
Type 1 T-helper cells:	all
Type 2 T-helper cells:	all

Results

P value: 0.009
FDR: over 50%

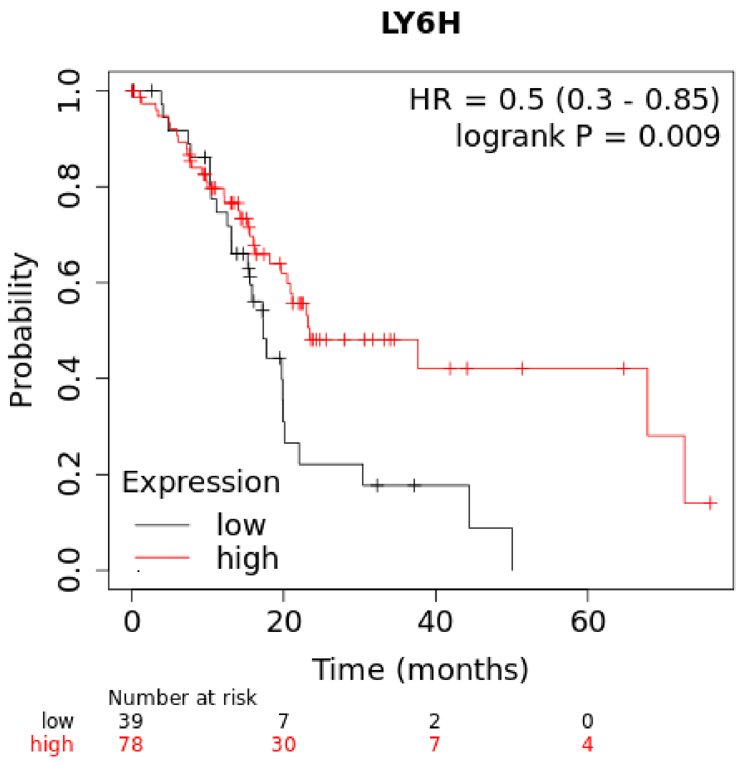

Click here to download the plot in TIFF format

Download plot as a PDF

Download p values vs. cutoff table

Median survival

Low expression cohort (months)	High expression cohort (months)
17.27	23.4

**RNAseq ID:**	GPIHBP1 -
**Survival:**	OS
**Auto select best cutoff:**	checked
**Follow up threshold:**	all
**Censore at threshold:**	checked
**Compute median over entire database:**	false
**Cutoff value used in analysis:**	32
**Expression range of the probe:**	7 - 323
**Invert HR values below 1:**	not checked

Restrictions

Tumor type: Pancreatic ductal adenocarcinoma

Restrict analysis to subtypes...

Stage:	all
Gender:	all
Race:	all
Grade:	all
Mutation burden:	all

Restrict analysis based on cellular content...

Basophils:	all
B-cells:	all
CD4+ memory T-cells:	all
CD8+ T-cells:	all
Eosinophils:	all
Macrophages:	all
Mesenchymal stem cells:	all
Natural killer T-cells:	decreased
Regulatory T-cells:	all
Type 1 T-helper cells:	all
Type 2 T-helper cells:	all

Results

**P value:**	0.0629
**FDR:**	100%

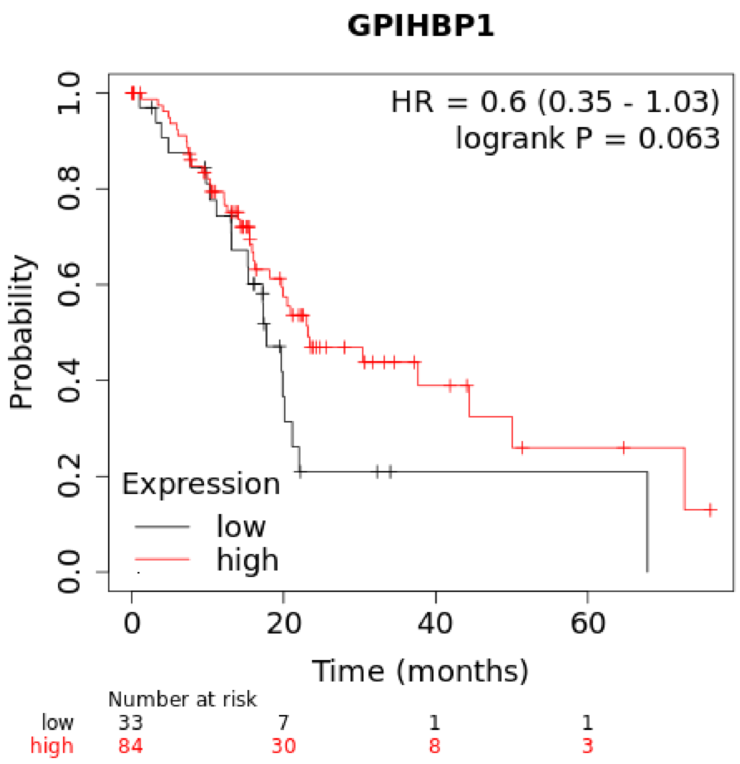

Click here to download the plot in TIFF format

Download plot as a PDF

Download p values vs. cutoff table

Median survival

Low expression cohort (months)	High expression cohort (months)
17.73	23.17

**RNAseq ID:**	LYPD4 -
**Survival:**	OS
**Auto select best cutoff:**	checked
**Follow up threshold:**	all
**Censore at threshold:**	checked
**Compute median over entire database:**	false
**Cutoff value used in analysis:**	0
**Expression range of the probe:**	0 - 11
**Invert HR values below 1:**	not checked

Restrictions

Tumor type: Pancreatic ductal adenocarcinoma

Restrict analysis to subtypes...

Stage:	all
Gender:	all
Race:	all
Grade:	all
Mutation burden:	all

Restrict analysis based on cellular content...

Basophils:	all

B-cells:	all
CD4+ memory T-cells:	all
CD8+ T-cells:	all
Eosinophils:	all
Macrophages:	all
Mesenchymal stem cells:	all
Natural killer T-cells:	decreased
Regulatory T-cells:	all
Type 1 T-helper cells:	all
Type 2 T-helper cells:	all

Results

P value: 0.0191
FDR: over 50%

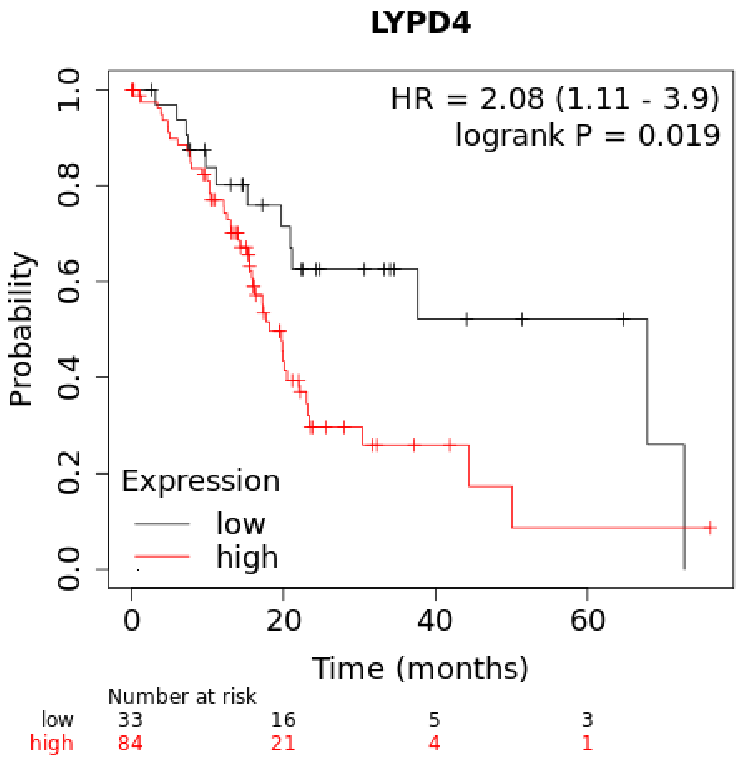

Click here to download the plot in TIFF format

Download plot as a PDF

Download p values vs. cutoff table

Median survival

Low expression cohort (months)	High expression cohort (months)
67.87	18.17

**RNAseq ID:**	CD177 -
**Survival:**	OS
**Auto select best cutoff:**	checked
**Follow up threshold:**	all
**Censore at threshold:**	checked
**Compute median over entire database:**	false
**Cutoff value used in analysis:**	58
**Expression range of the probe:**	0 - 6100
**Invert HR values below 1:**	not checked

Restrictions

Tumor type: Pancreatic ductal adenocarcinoma

Restrict analysis to subtypes...

Stage: all
Gender: all
Race: all
Grade: all
Mutation burden: all

Restrict analysis based on cellular content...

Basophils: all
B-cells: all
CD4+ memory T-cells: all
CD8+ T-cells: all
Eosinophils: all
Macrophages: all
Mesenchymal stem cells: all
Natural killer T-cells: decreased
Regulatory T-cells: all
Type 1 T-helper cells: all
Type 2 T-helper cells: all

Results

P value: 0.3255
FDR: 100%

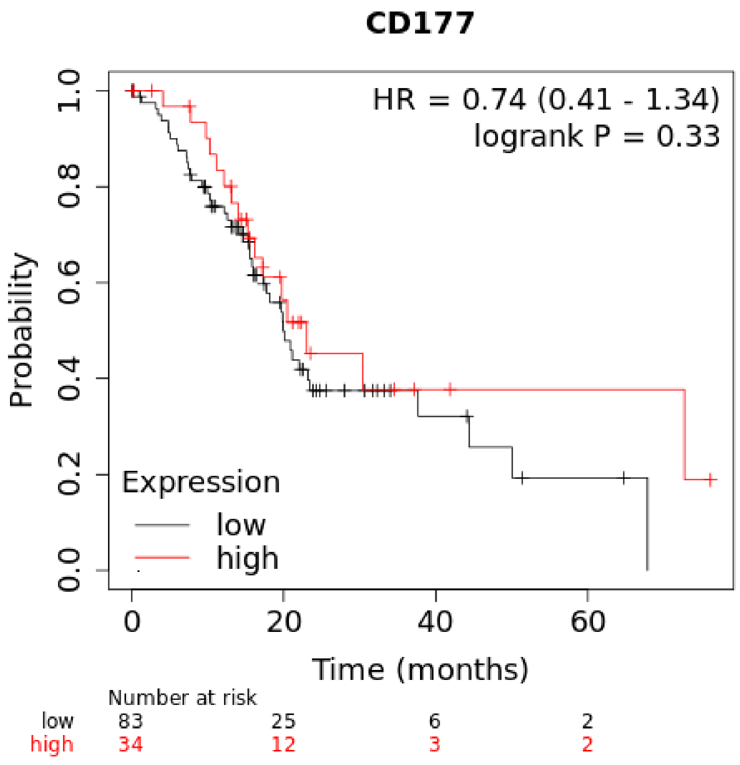

Click here to download the plot in TIFF format

Download plot as a PDF

Download p values vs. cutoff table

Median survival

Low expression cohort (months)	High expression cohort (months)
19.93	23.03

**RNAseq ID:**	TEX101 -
**Survival:**	OS
**Auto select best cutoff:**	checked
**Follow up threshold:**	all
**Censore at threshold:**	checked
**Compute median over entire database:**	false
**Cutoff value used in analysis:**	1
**Expression range of the probe:**	0 - 149
**Invert HR values below 1:**	not checked

Restrictions

Tumor type: Pancreatic ductal adenocarcinoma

Restrict analysis to subtypes...

Stage:	all
Gender:	all
Race:	all
Grade:	all
Mutation burden:	all

Restrict analysis based on cellular content...

Basophils:	all
B-cells:	all
CD4+ memory T-cells:	all
CD8+ T-cells:	all
Eosinophils:	all
Macrophages:	all
Mesenchymal stem cells:	all
Natural killer T-cells:	decreased
Regulatory T-cells:	all
Type 1 T-helper cells:	all
Type 2 T-helper cells:	all

Results

**P value:**	0.2829
**FDR:**	100%

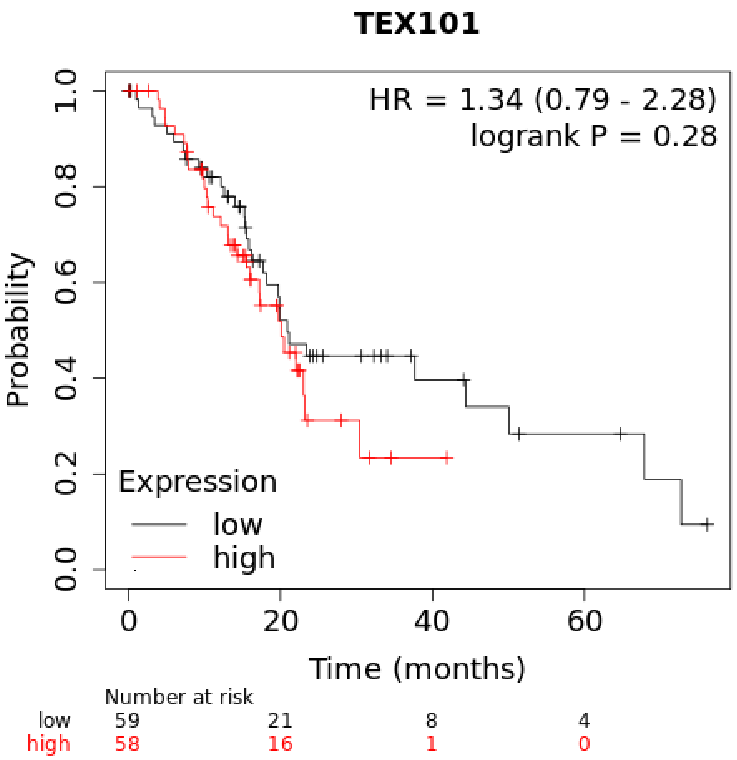

Click here to download the plot in TIFF format

Download plot as a PDF

Download p values vs. cutoff table

Median survival

Low expression cohort (months)	High expression cohort (months)
20.9	20.1

**RNAseq ID:**	LYPD3 -
**Survival:**	OS
**Auto select best cutoff:**	checked
**Follow up threshold:**	all
**Censore at threshold:**	checked
**Compute median over entire database:**	false
**Cutoff value used in analysis:**	148
**Expression range of the probe:**	9 - 7684
**Invert HR values below 1:**	not checked

Restrictions

Tumor type: Pancreatic ductal adenocarcinoma

Restrict analysis to subtypes...

Stage:	all
Gender:	all
Race:	all
Grade:	all
Mutation burden:	all

Restrict analysis based on cellular content...

Basophils:	all

B-cells:	all
CD4+ memory T-cells:	all
CD8+ T-cells:	all
Eosinophils:	all
Macrophages:	all
Mesenchymal stem cells:	all
Natural killer T-cells:	decreased
Regulatory T-cells:	all
Type 1 T-helper cells:	all
Type 2 T-helper cells:	all

Results

P value: 0.1744
FDR: 100%

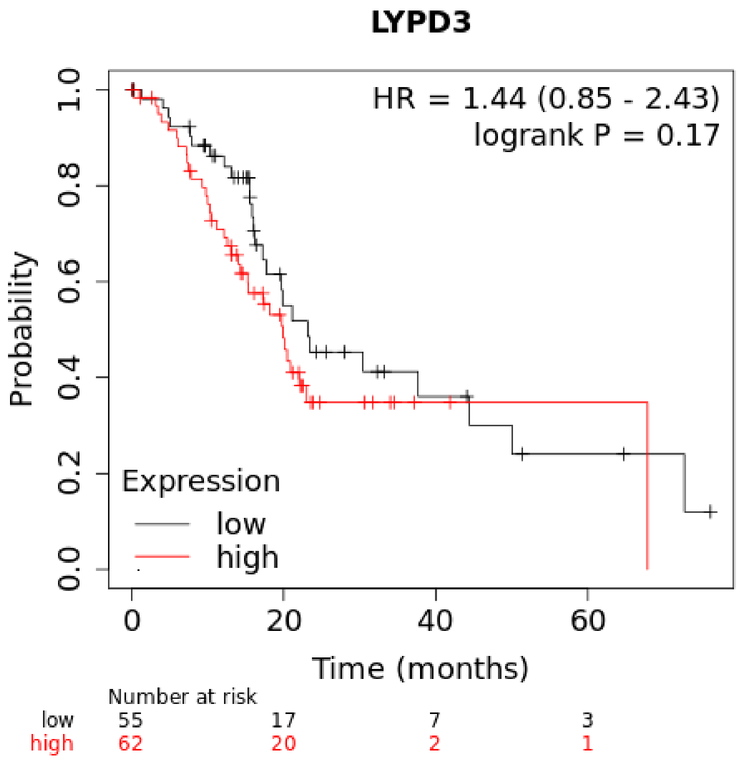

Click here to download the plot in TIFF format

Download plot as a PDF

Download p values vs. cutoff table

Median survival

Low expression cohort (months)	High expression cohort (months)
23.17	19.93

**RNAseq ID:**	PINLYP -
**Survival:**	OS
**Auto select best cutoff:**	checked
**Follow up threshold:**	all
**Censore at threshold:**	checked
**Compute median over entire database:**	false
**Cutoff value used in analysis:**	76
**Expression range of the probe:**	5 - 387
**Invert HR values below 1:**	not checked

Restrictions

Tumor type: Pancreatic ductal adenocarcinoma

Restrict analysis to subtypes...

Stage:	all
Gender:	all
Race:	all
Grade:	all
Mutation burden:	all

Restrict analysis based on cellular content...

Basophils:	all
B-cells:	all
CD4+ memory T-cells:	all
CD8+ T-cells:	all
Eosinophils:	all
Macrophages:	all
Mesenchymal stem cells:	all
Natural killer T-cells:	decreased
Regulatory T-cells:	all
Type 1 T-helper cells:	all
Type 2 T-helper cells:	all

Results

P value: 0.1192
FDR: 100%

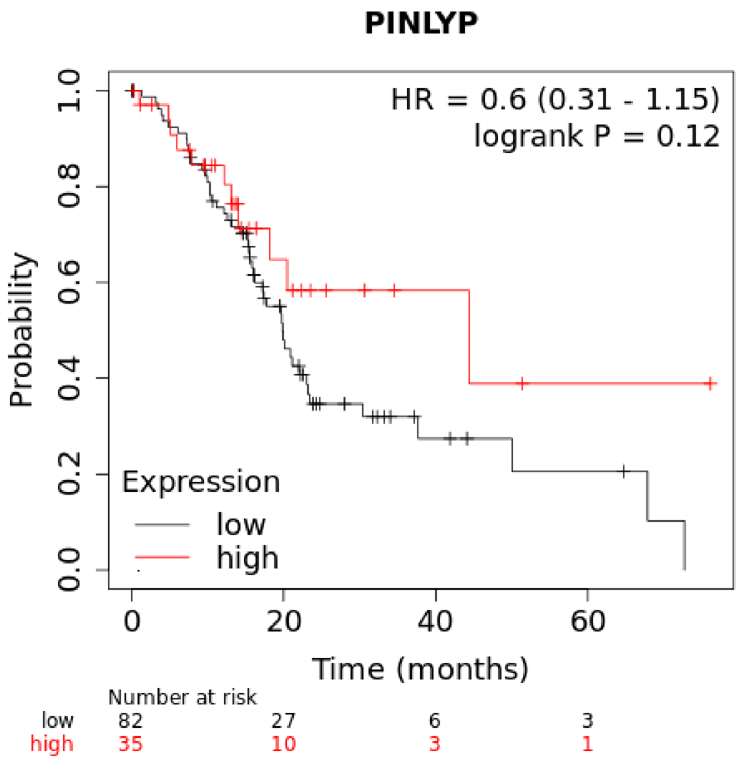

Click here to download the plot in TIFF format

Download plot as a PDF

Download p values vs. cutoff table

Median survival

Low expression cohort (months)	High expression cohort (months)
19.87	44.4

**RNAseq ID:**	PLAUR -
**Survival:**	OS
**Auto select best cutoff:**	checked
**Follow up threshold:**	all
**Censore at threshold:**	checked
**Compute median over entire database:**	false
**Cutoff value used in analysis:**	3205
**Expression range of the probe:**	47 - 18314
**Invert HR values below 1:**	not checked

Restrictions

Tumor type: Pancreatic ductal adenocarcinoma

Restrict analysis to subtypes...

Stage:	all
Gender:	all
Race:	all
Grade:	all
Mutation burden:	all

Restrict analysis based on cellular content...

Basophils:	all
B-cells:	all
CD4+ memory T-cells:	all
CD8+ T-cells:	all
Eosinophils:	all
Macrophages:	all
Mesenchymal stem cells:	all
Natural killer T-cells:	decreased
Regulatory T-cells:	all
Type 1 T-helper cells:	all
Type 2 T-helper cells:	all

Results

**P value:**	0.4231
**FDR:**	100%

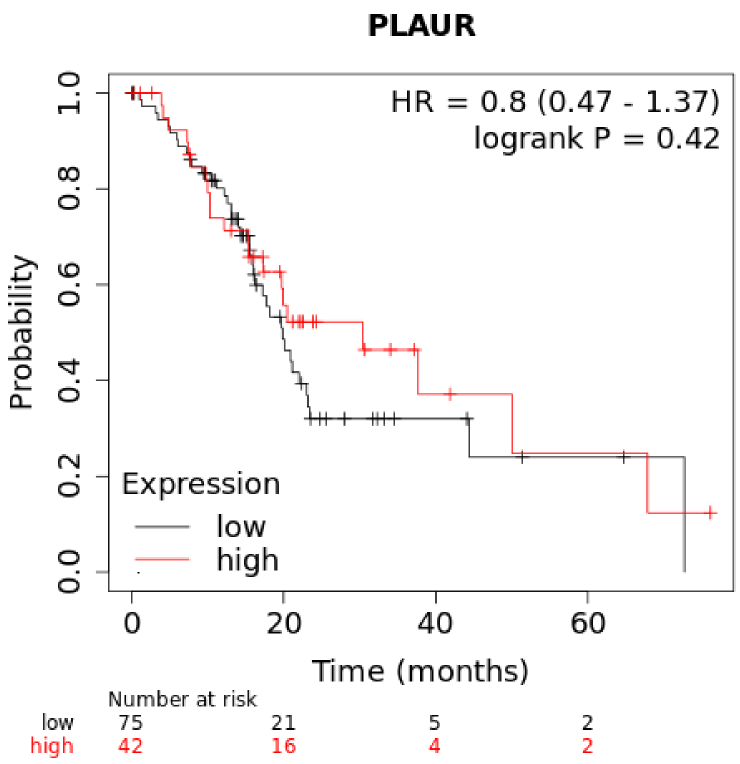

Click here to download the plot in TIFF format

Download plot as a PDF

Download p values vs. cutoff table

Median survival

Low expression cohort (months)	High expression cohort (months)
19.87	30.43

**RNAseq ID:**	LYPD5 -
**Survival:**	OS
**Auto select best cutoff:**	checked
**Follow up threshold:**	all
**Censore at threshold:**	checked
**Compute median over entire database:**	false
**Cutoff value used in analysis:**	107
**Expression range of the probe:**	1 - 578
**Invert HR values below 1:**	not checked

Restrictions

Tumor type: Pancreatic ductal adenocarcinoma

Restrict analysis to subtypes...

Stage:	all
Gender:	all
Race:	all
Grade:	all
Mutation burden:	all

Restrict analysis based on cellular content...

Basophils:	all

B-cells:	all
CD4+ memory T-cells:	all
CD8+ T-cells:	all
Eosinophils:	all
Macrophages:	all
Mesenchymal stem cells:	all
Natural killer T-cells:	decreased
Regulatory T-cells:	all
Type 1 T-helper cells:	all
Type 2 T-helper cells:	all

Results

P value: 0.0471
FDR: over 50%

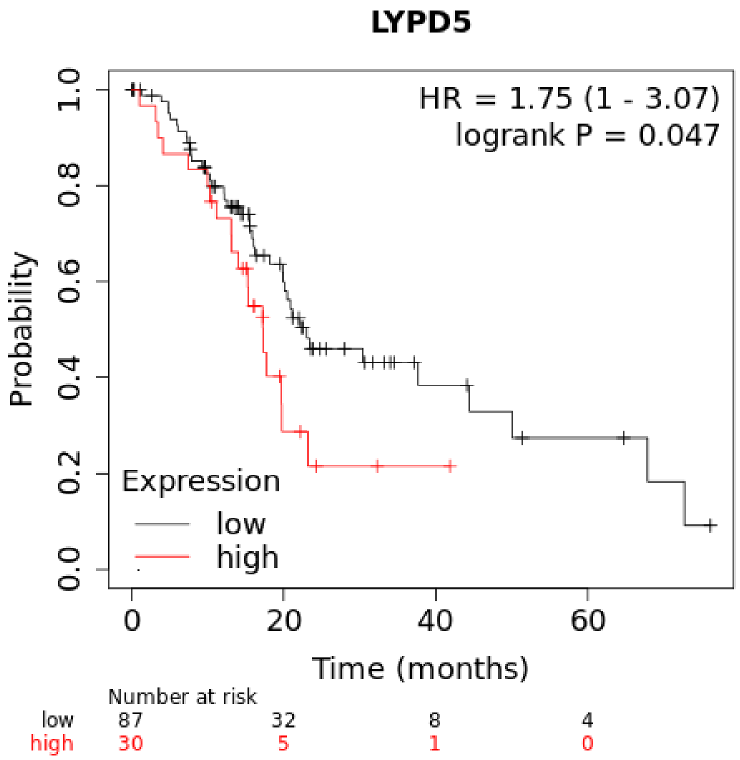

Click here to download the plot in TIFF format

Download plot as a PDF

Download p values vs. cutoff table

Median survival

Low expression cohort (months)	High expression cohort (months)
23.03	17.27

**RNAseq ID:**	SPACA4 -
**Survival:**	OS
**Auto select best cutoff:**	checked
**Follow up threshold:**	all
**Censore at threshold:**	checked
**Compute median over entire database:**	false
**Cutoff value used in analysis:**	8
**Expression range of the probe:**	1 - 120
**Invert HR values below 1:**	not checked

Restrictions

Tumor type: Pancreatic ductal adenocarcinoma

Restrict analysis to subtypes...

Stage:	all
Gender:	all
Race:	all
Grade:	all
Mutation burden:	all

Restrict analysis based on cellular content...

Basophils:	all
B-cells:	all
CD4+ memory T-cells:	all
CD8+ T-cells:	all
Eosinophils:	all
Macrophages:	all
Mesenchymal stem cells:	all
Natural killer T-cells:	decreased
Regulatory T-cells:	all
Type 1 T-helper cells:	all
Type 2 T-helper cells:	all

Results

P value: 0.3044
FDR: 100%

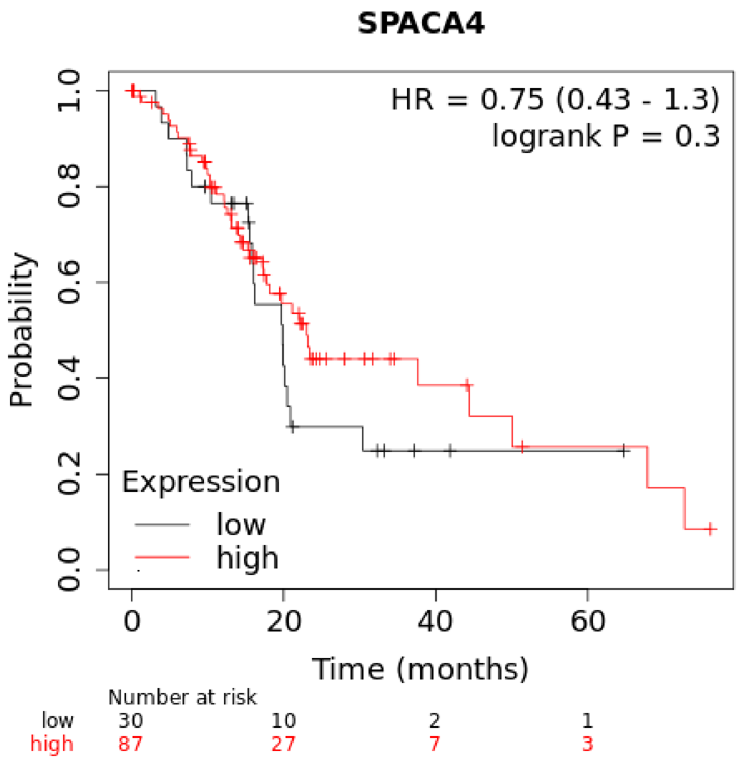

Click here to download the plot in TIFF format

Download plot as a PDF

Download p values vs. cutoff table

Median survival

Low expression cohort (months)	High expression cohort (months)
19.87	23.03

**RNAseq ID:**	ACRV1 -
**Survival:**	OS
**Auto select best cutoff:**	checked
**Follow up threshold:**	all
**Censore at threshold:**	checked
**Compute median over entire database:**	false
**Cutoff value used in analysis:**	6
**Expression range of the probe:**	0 - 71
**Invert HR values below 1:**	not checked

Restrictions

Tumor type: Pancreatic ductal adenocarcinoma

Restrict analysis to subtypes...

Stage:	all
Gender:	all
Race:	all
Grade:	all
Mutation burden:	all

Restrict analysis based on cellular content...

Basophils:	all
B-cells:	all
CD4+ memory T-cells:	all
CD8+ T-cells:	all
Eosinophils:	all
Macrophages:	all
Mesenchymal stem cells:	all
Natural killer T-cells:	decreased
Regulatory T-cells:	all
Type 1 T-helper cells:	all
Type 2 T-helper cells:	all

Results

**P value:**	0.046
**FDR:**	over 50%

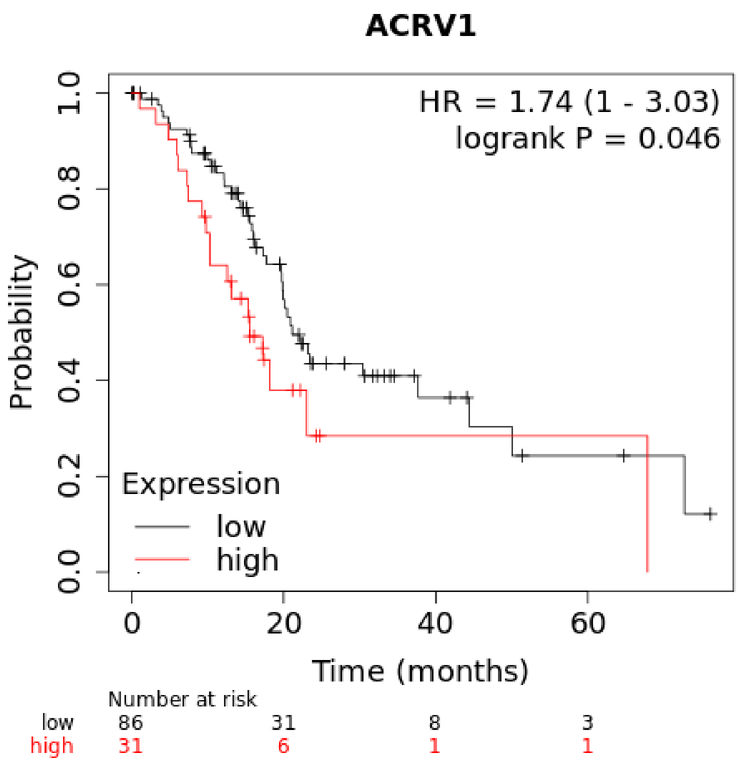

Click here to download the plot in TIFF format

Download plot as a PDF

Download p values vs. cutoff table

Median survival

Low expression cohort (months)	High expression cohort (months)
21.13	15.53

**RNAseq ID:**	PATE1 -
**Survival:**	OS
**Auto select best cutoff:**	checked
**Follow up threshold:**	all
**Censore at threshold:**	checked
**Compute median over entire database:**	false
**Cutoff value used in analysis:**	0
**Expression range of the probe:**	0 - 1
**Invert HR values below 1:**	not checked

Restrictions

Tumor type: Pancreatic ductal adenocarcinoma

Restrict analysis to subtypes...

Stage:	all
Gender:	all
Race:	all
Grade:	all
Mutation burden:	all

Restrict analysis based on cellular content...

Basophils:	all

B-cells:	all
CD4+ memory T-cells:	all
CD8+ T-cells:	all
Eosinophils:	all
Macrophages:	all
Mesenchymal stem cells:	all
Natural killer T-cells:	decreased
Regulatory T-cells:	all
Type 1 T-helper cells:	all
Type 2 T-helper cells:	all

Results

P value: 0.0065
FDR: 50%

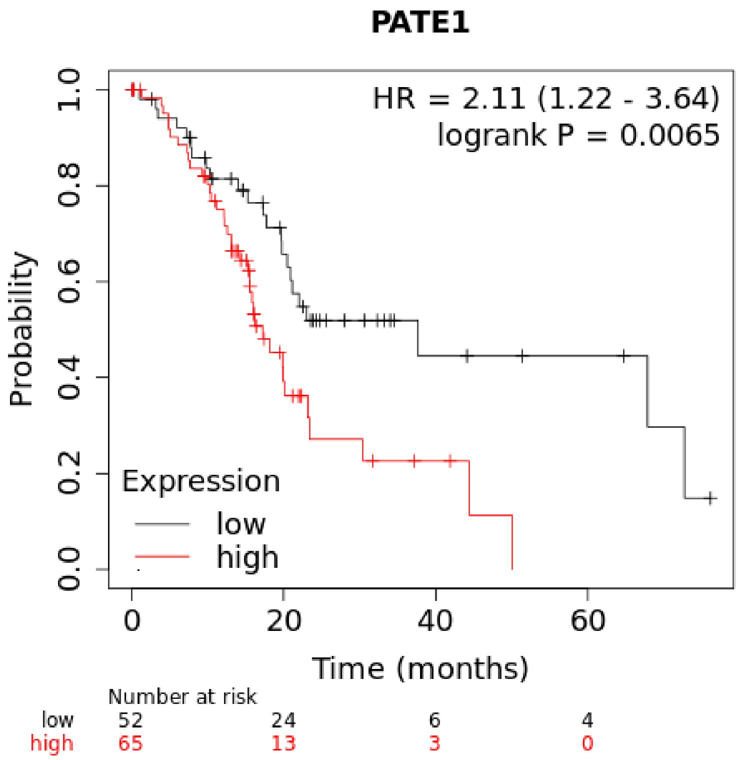

Click here to download the plot in TIFF format

Download plot as a PDF

Download p values vs. cutoff table

Median survival

Low expression cohort (months)	High expression cohort (months)
37.67	17.23

**RNAseq ID:**	PATE2 -
**Survival:**	OS
**Auto select best cutoff:**	checked
**Follow up threshold:**	all
**Censore at threshold:**	checked
**Compute median over entire database:**	false
**Cutoff value used in analysis:**	1
**Expression range of the probe:**	0 - 7
**Invert HR values below 1:**	not checked

Restrictions

Tumor type: Pancreatic ductal adenocarcinoma

Restrict analysis to subtypes...

Stage: all
Gender: all
Race: all
Grade: all
Mutation burden: all

Restrict analysis based on cellular content...

Basophils: all
B-cells: all
CD4+ memory T-cells: all
CD8+ T-cells: all
Eosinophils: all
Macrophages: all
Mesenchymal stem cells: all
Natural killer T-cells: decreased
Regulatory T-cells: all
Type 1 T-helper cells: all
Type 2 T-helper cells: all

Results

P value: 0.0106
FDR: over 50%

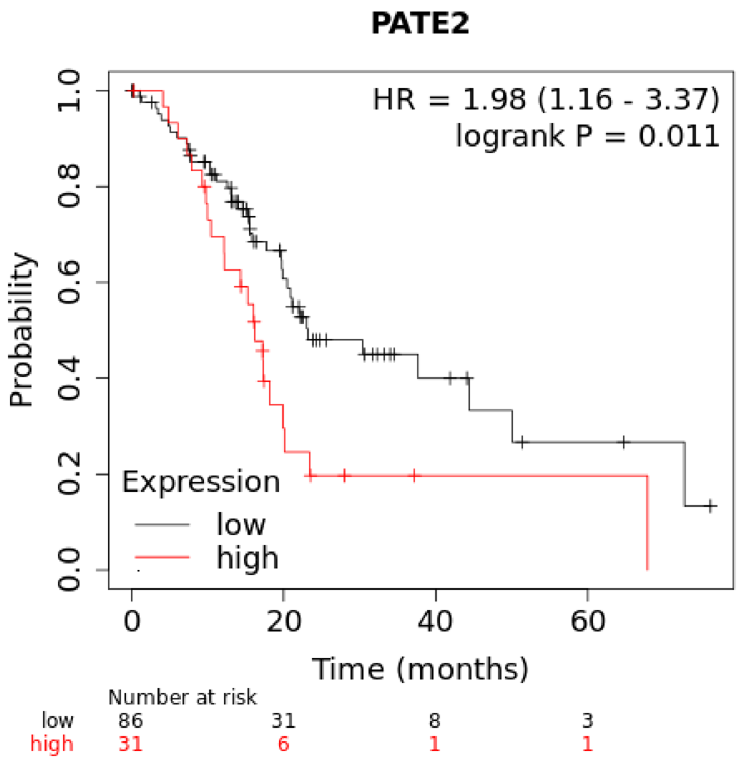

Click here to download the plot in TIFF format

Download plot as a PDF

Download p values vs. cutoff table

Median survival

Low expression cohort (months)	High expression cohort (months)
23.17	16.2

**RNAseq ID:**	PATE3 -
**Survival:**	OS
**Auto select best cutoff:**	checked
**Follow up threshold:**	all
**Censore at threshold:**	checked
**Compute median over entire database:**	false
**Cutoff value used in analysis:**	0
**Expression range of the probe:**	0 - 1
**Invert HR values below 1:**	not checked

Restrictions

Tumor type: Pancreatic ductal adenocarcinoma

Restrict analysis to subtypes...

Stage:	all
Gender:	all
Race:	all
Grade:	all
Mutation burden:	all

Restrict analysis based on cellular content...

Basophils:	all
B-cells:	all
CD4+ memory T-cells:	all
CD8+ T-cells:	all
Eosinophils:	all
Macrophages:	all
Mesenchymal stem cells:	all
Natural killer T-cells:	decreased
Regulatory T-cells:	all
Type 1 T-helper cells:	all
Type 2 T-helper cells:	all

Results

**P value:**	0.0345
**FDR:**	over 50%

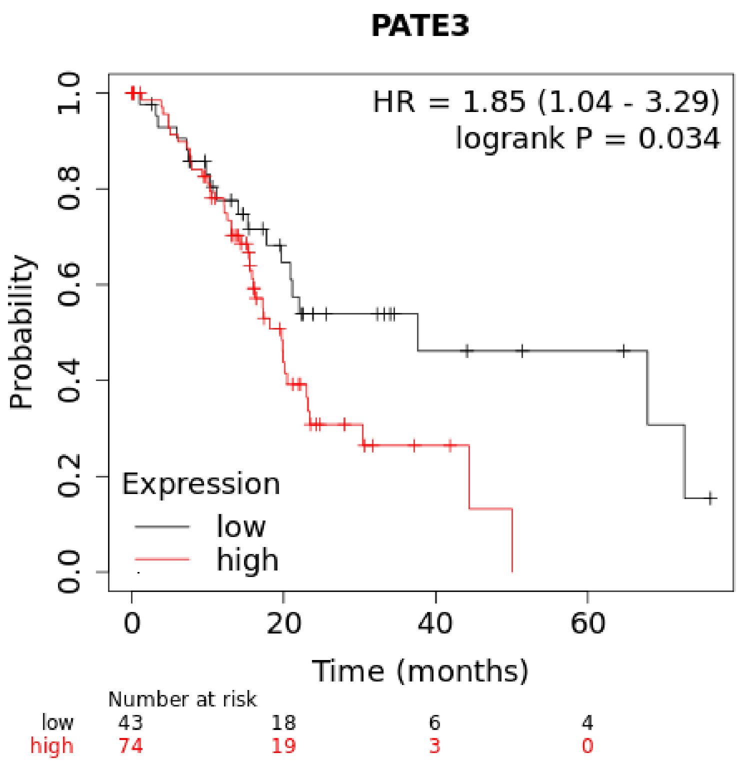

Click here to download the plot in TIFF format

Download plot as a PDF

Download p values vs. cutoff table

Median survival

Low expression cohort (months)	High expression cohort (months)
37.67	19.77

**RNAseq ID:**	PATE4 -
**Survival:**	OS
**Auto select best cutoff:**	checked
**Follow up threshold:**	all
**Censore at threshold:**	checked
**Compute median over entire database:**	false
**Cutoff value used in analysis:**	0
**Expression range of the probe:**	0 - 3
**Invert HR values below 1:**	not checked

Restrictions

Tumor type: Pancreatic ductal adenocarcinoma

Restrict analysis to subtypes...

Stage:	all
Gender:	all
Race:	all
Grade:	all
Mutation burden:	all

Restrict analysis based on cellular content...

Basophils: all

B-cells:	all
CD4+ memory T-cells:	all
CD8+ T-cells:	all
Eosinophils:	all
Macrophages:	all
Mesenchymal stem cells:	all
Natural killer T-cells:	decreased
Regulatory T-cells:	all
Type 1 T-helper cells:	all
Type 2 T-helper cells:	all

Results

P value: 0.0109
FDR: 50%

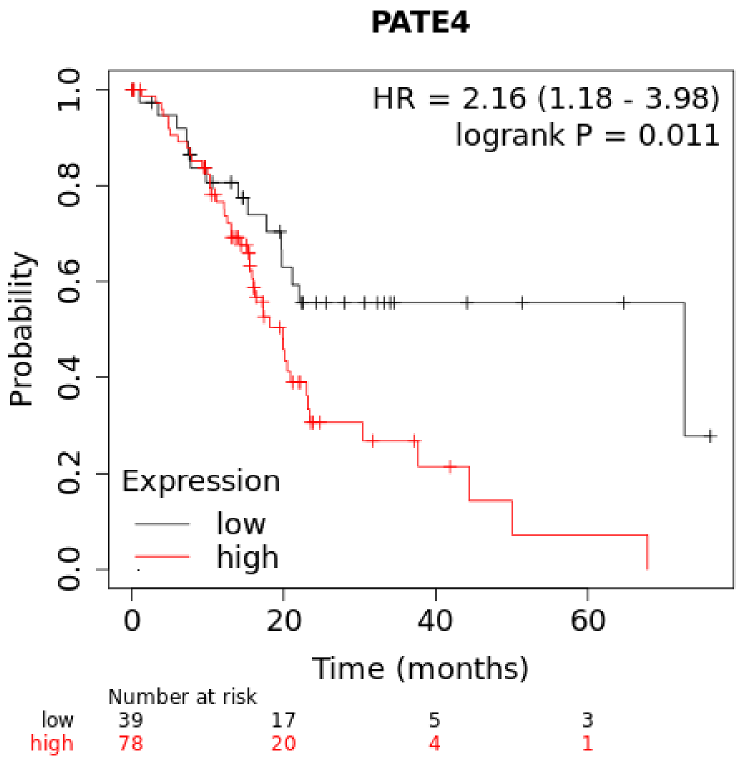

Click here to download the plot in TIFF format

Download plot as a PDF

Download p values vs. cutoff table

Median survival

Low expression cohort (months)	High expression cohort (months)
72.73	19.87

**RNAseq ID:**	CD59 -
**Survival:**	OS
**Auto select best cutoff:**	checked
**Follow up threshold:**	all
**Censore at threshold:**	checked
**Compute median over entire database:**	false
**Cutoff value used in analysis:**	17322
**Expression range of the probe:**	4436 - 32118
**Invert HR values below 1:**	not checked

Restrictions

Tumor type: Pancreatic ductal adenocarcinoma

Restrict analysis to subtypes...

Stage:	all
Gender:	all
Race:	all
Grade:	all
Mutation burden:	all

Restrict analysis based on cellular content...

Basophils:	all
B-cells:	all
CD4+ memory T-cells:	all
CD8+ T-cells:	all
Eosinophils:	all
Macrophages:	all
Mesenchymal stem cells:	all
Natural killer T-cells:	decreased
Regulatory T-cells:	all
Type 1 T-helper cells:	all
Type 2 T-helper cells:	all

Results

P value: 0.0031
FDR: 20%

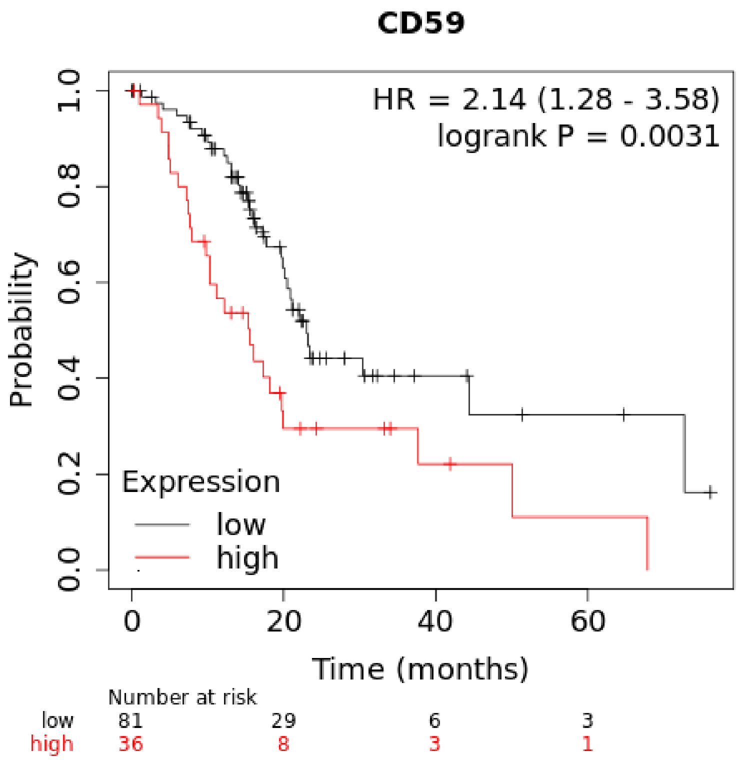

Click here to download the plot in TIFF format

Download plot as a PDF

Download p values vs. cutoff table

Median survival

Low expression cohort (months)	High expression cohort (months)
23.03	15.57

**RNAseq ID:**	LY6G6C -
**Survival:**	OS
**Auto select best cutoff:**	checked
**Follow up threshold:**	all
**Censore at threshold:**	checked
**Compute median over entire database:**	false
**Cutoff value used in analysis:**	14
**Expression range of the probe:**	0 - 251
**Invert HR values below 1:**	not checked

Restrictions

Tumor type: Pancreatic ductal adenocarcinoma

Restrict analysis to subtypes...

Stage:	all
Gender:	all
Race:	all
Grade:	all
Mutation burden:	all

Restrict analysis based on cellular content...

Basophils:	all
B-cells:	all
CD4+ memory T-cells:	all
CD8+ T-cells:	all
Eosinophils:	all
Macrophages:	all
Mesenchymal stem cells:	all
Natural killer T-cells:	decreased
Regulatory T-cells:	all
Type 1 T-helper cells:	all
Type 2 T-helper cells:	all

Results

**P value:**	0.1708
**FDR:**	100%

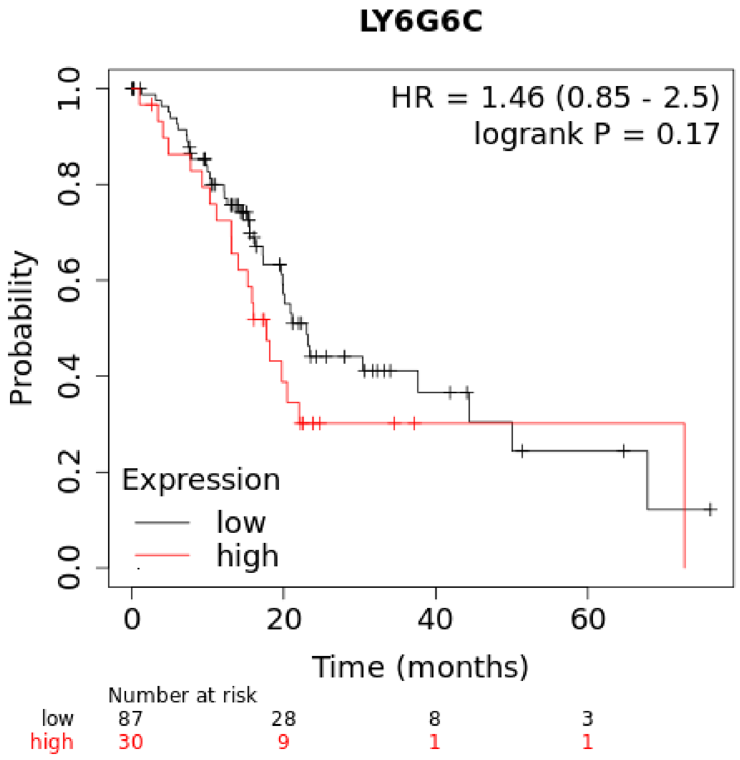

Click here to download the plot in TIFF format

Download plot as a PDF

Download p values vs. cutoff table

Median survival

Low expression cohort (months)	High expression cohort (months)
23.03	17.73

RNAseq ID: LY6G6D -
Survival: OS
Auto select best cutoff: checked
Follow up threshold: all
Censore at threshold: checked
Compute median over entire database: false
Cutoff value used in analysis: 0
Expression range of the probe: 0 - 2
Invert HR values below 1: not checked

Restrictions

Tumor type: Pancreatic ductal adenocarcinoma

Restrict analysis to subtypes...

Stage: all
Gender: all
Race: all
Grade: all
Mutation burden: all

Restrict analysis based on cellular content...

Basophils: all

B-cells:	all
CD4+ memory T-cells:	all
CD8+ T-cells:	all
Eosinophils:	all
Macrophages:	all
Mesenchymal stem cells:	all
Natural killer T-cells:	decreased
Regulatory T-cells:	all
Type 1 T-helper cells:	all
Type 2 T-helper cells:	all

Results

P value: 0.0016
FDR: 20%

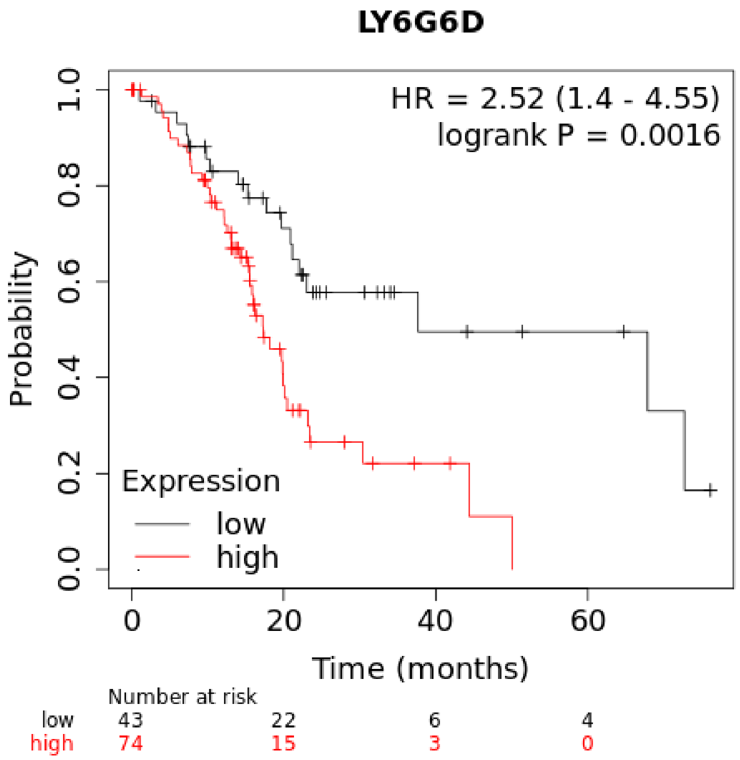

Click here to download the plot in TIFF format

Download plot as a PDF

Download p values vs. cutoff table

Median survival

Low expression cohort (months)	High expression cohort (months)
37.67	17.27

**RNAseq ID:**	LY6G6F -
**Survival:**	OS
**Auto select best cutoff:**	checked
**Follow up threshold:**	all
**Censore at threshold:**	checked
**Compute median over entire database:**	false
**Cutoff value used in analysis:**	0
**Expression range of the probe:**	0 - 5
**Invert HR values below 1:**	not checked

Restrictions

Tumor type: Pancreatic ductal adenocarcinoma

Restrict analysis to subtypes...

Stage:	all
Gender:	all
Race:	all
Grade:	all
Mutation burden:	all

Restrict analysis based on cellular content...

Basophils:	all
B-cells:	all
CD4+ memory T-cells:	all
CD8+ T-cells:	all
Eosinophils:	all
Macrophages:	all
Mesenchymal stem cells:	all
Natural killer T-cells:	decreased
Regulatory T-cells:	all
Type 1 T-helper cells:	all
Type 2 T-helper cells:	all

Results

P value: 0.0066
FDR: 50%

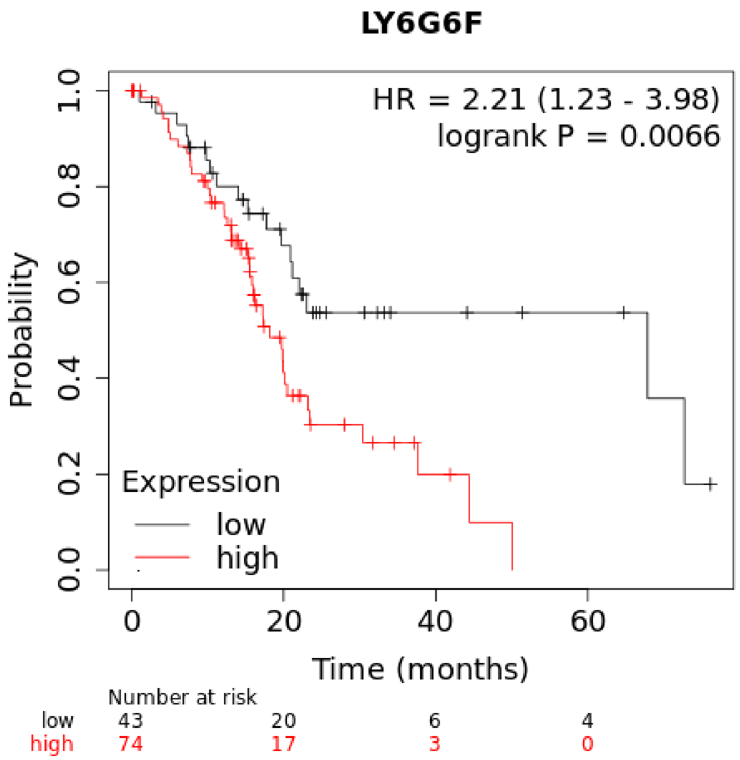

Click here to download the plot in TIFF format

Download plot as a PDF

Download p values vs. cutoff table

Median survival

Low expression cohort (months)	High expression cohort (months)
67.87	18.17

**RNAseq ID:**	LY6G5C -
**Survival:**	OS
**Auto select best cutoff:**	checked
**Follow up threshold:**	all
**Censore at threshold:**	checked
**Compute median over entire database:**	false
**Cutoff value used in analysis:**	36
**Expression range of the probe:**	17 - 253
**Invert HR values below 1:**	not checked

Restrictions

Tumor type: Pancreatic ductal adenocarcinoma

Restrict analysis to subtypes...

Stage:	all
Gender:	all
Race:	all
Grade:	all
Mutation burden:	all

Restrict analysis based on cellular content...

Basophils:	all
B-cells:	all
CD4+ memory T-cells:	all
CD8+ T-cells:	all
Eosinophils:	all
Macrophages:	all
Mesenchymal stem cells:	all
Natural killer T-cells:	decreased
Regulatory T-cells:	all
Type 1 T-helper cells:	all
Type 2 T-helper cells:	all

Results

**P value:**	0.0049
**FDR:**	50%

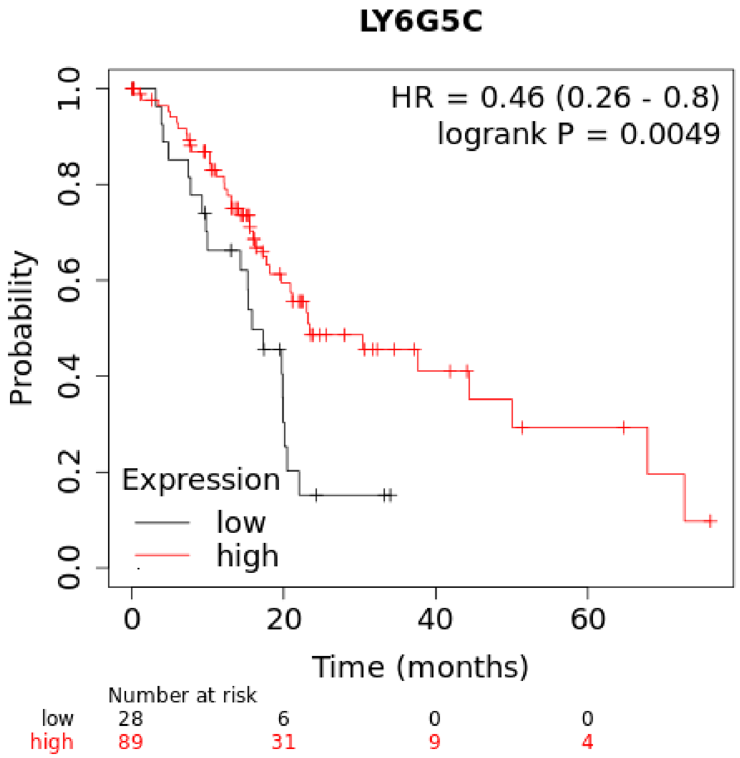

Click here to download the plot in TIFF format

Download plot as a PDF

Download p values vs. cutoff table

Median survival

Low expression cohort (months)	High expression cohort (months)
15.87	23.4

**RNAseq ID:**	LY6G5B -
**Survival:**	OS
**Auto select best cutoff:**	checked
**Follow up threshold:**	all
**Censore at threshold:**	checked
**Compute median over entire database:**	false
**Cutoff value used in analysis:**	37
**Expression range of the probe:**	3 - 173
**Invert HR values below 1:**	not checked

Restrictions

Tumor type: Pancreatic ductal adenocarcinoma

Restrict analysis to subtypes...

Stage:	all
Gender:	all
Race:	all
Grade:	all
Mutation burden:	all

Restrict analysis based on cellular content...

Basophils:	all

B-cells:	all
CD4+ memory T-cells:	all
CD8+ T-cells:	all
Eosinophils:	all
Macrophages:	all
Mesenchymal stem cells:	all
Natural killer T-cells:	decreased
Regulatory T-cells:	all
Type 1 T-helper cells:	all
Type 2 T-helper cells:	all

Results

P value: 0.0034
FDR: 50%

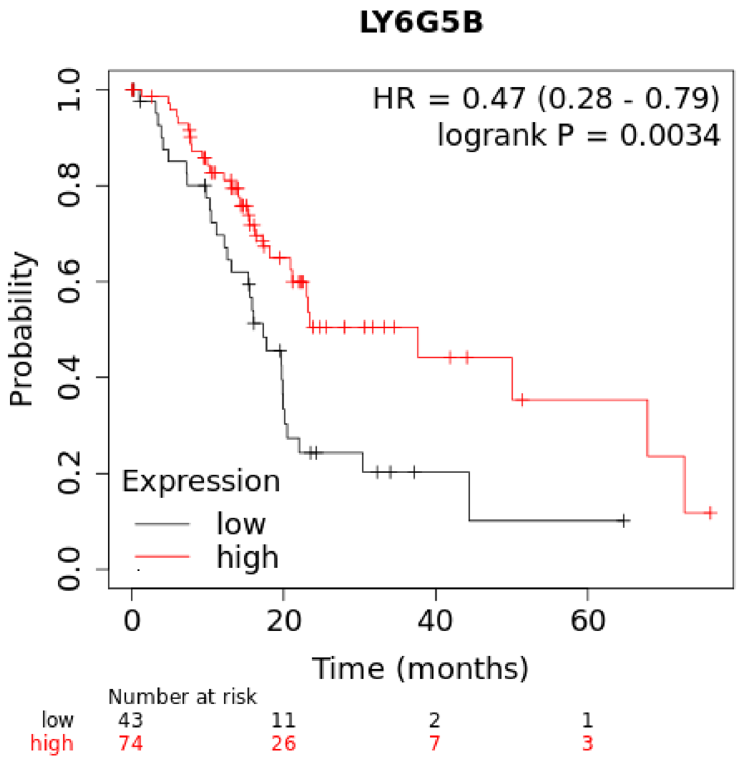

Click here to download the plot in TIFF format

Download plot as a PDF

Download p values vs. cutoff table

Median survival

Low expression cohort (months)	High expression cohort (months)
17.27	37.67

You can save the plots by right-clicking the image and then selecting "Save image as...". To generate a high resolution TIFF image, please adjust the "Settings" in the analysis page.

KMplot.com Copyright © 2009-2020 | Contact | Sitemap

Figure S7: KM plots and other raw data for the data depicted in Table 7

Pan-cancer

KM plotter | Home | Vote | Download | Updates | Contact

The desired RNAseq ID is valid: PSCA (-), LY6K (-), SLURP1 (-), LYPD2 (-), LY6D (-), GML (-), LY6E (-), LY6L (-), LY6H (-), GPIHBP1 (-), LYPD4 (-), CD177 (-), TEX101 (-), LYPD3 (-), PINLYP (-), PLAUR (-), LYPD5 (-), SPACA4 (-), ACRV1 (-), PATE1 (-), PATE2 (-), PATE3 (-), PATE4 (-), CD59 (-), LY6G6C (-), LY6G6D (-), LY6G6F (-), LY6G5C (-), LY6G5B (-),

**RNAseq ID:**	PSCA -
**Survival:**	OS
**Auto select best cutoff:**	checked
**Follow up threshold:**	all
**Censore at threshold:**	checked
**Compute median over entire database:**	false
**Cutoff value used in analysis:**	821
**Expression range of the probe:**	5 - 17395
**Invert HR values below 1:**	not checked

Restrictions

Tumor type: Pancreatic ductal adenocarcinoma

Restrict analysis to subtypes...

Stage:	all
Gender:	all
Race:	all
Grade:	all
Mutation burden:	all

Restrict analysis based on cellular content...

Basophils:	all
B-cells:	all
CD4+ memory T-cells:	enriched
CD8+ T-cells:	all
Eosinophils:	all
Macrophages:	all
Mesenchymal stem cells:	all
Natural killer T-cells:	all
Regulatory T-cells:	all
Type 1 T-helper cells:	all
Type 2 T-helper cells:	all

Results

P value: 0.1014
FDR: 100%

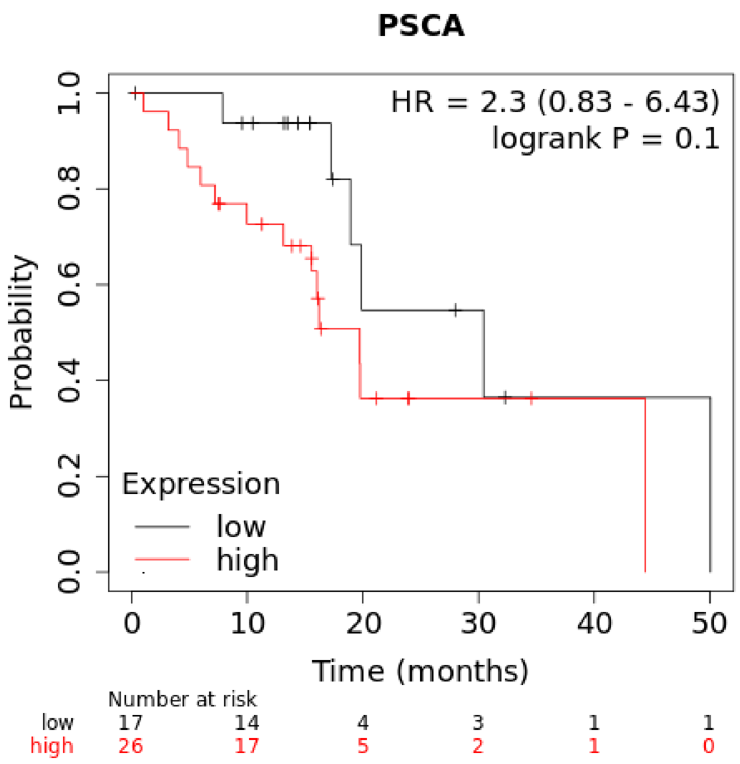

Click here to download the plot in TIFF format

Download plot as a PDF

Download p values vs. cutoff table

Median survival

Low expression cohort (months)	High expression cohort (months)
30.43	19.73

**RNAseq ID:**	LY6K -
**Survival:**	OS
**Auto select best cutoff:**	checked
**Follow up threshold:**	all
**Censore at threshold:**	checked
**Compute median over entire database:**	false
**Cutoff value used in analysis:**	12
**Expression range of the probe:**	0 - 1332
**Invert HR values below 1:**	not checked

Restrictions

Tumor type: Pancreatic ductal adenocarcinoma

Restrict analysis to subtypes...

Stage: all
Gender: all
Race: all
Grade: all
Mutation burden: all

Restrict analysis based on cellular content...

Basophils: all

B-cells:	all
CD4+ memory T-cells:	enriched
CD8+ T-cells:	all
Eosinophils:	all
Macrophages:	all
Mesenchymal stem cells:	all
Natural killer T-cells:	all
Regulatory T-cells:	all
Type 1 T-helper cells:	all
Type 2 T-helper cells:	all

Results

P value: 0.1345
FDR: 100%

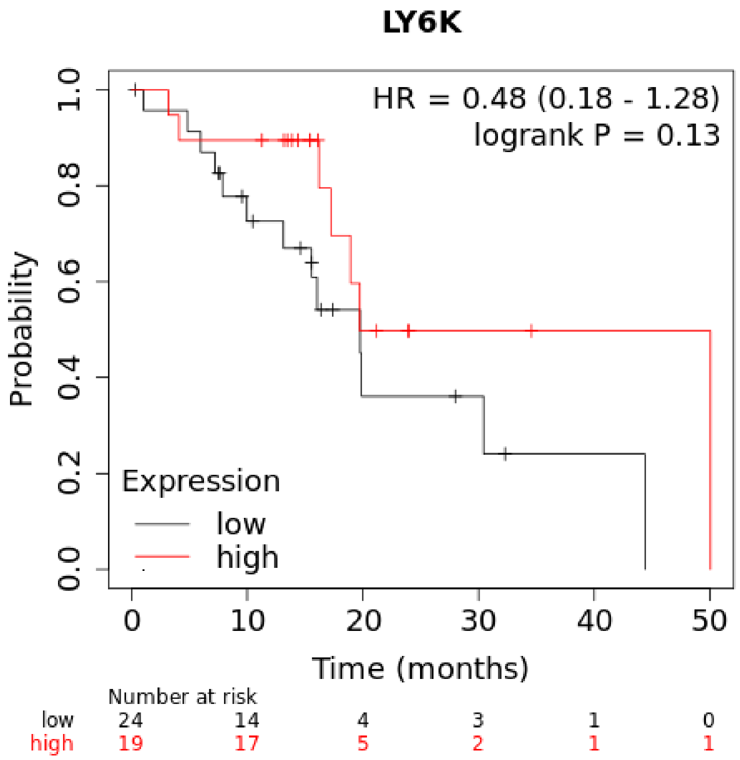

[Click here to download the plot in TIFF format]

[Download plot as a PDF]

[Download p values vs. cutoff table]

Median survival

Low expression cohort (months)	High expression cohort (months)
19.77	19.73

**RNAseq ID:**	SLURP1 -
**Survival:**	OS
**Auto select best cutoff:**	checked
**Follow up threshold:**	all
**Censore at threshold:**	checked
**Compute median over entire database:**	false
**Cutoff value used in analysis:**	1
**Expression range of the probe:**	0 - 7
**Invert HR values below 1:**	not checked

Restrictions

Tumor type: Pancreatic ductal adenocarcinoma

Restrict analysis to subtypes...

Stage:	all
Gender:	all
Race:	all
Grade:	all
Mutation burden:	all

Restrict analysis based on cellular content...

Basophils:	all
B-cells:	all
CD4+ memory T-cells:	enriched
CD8+ T-cells:	all
Eosinophils:	all
Macrophages:	all
Mesenchymal stem cells:	all
Natural killer T-cells:	all
Regulatory T-cells:	all
Type 1 T-helper cells:	all
Type 2 T-helper cells:	all

Results

P value: 0.0328
FDR: over 50%

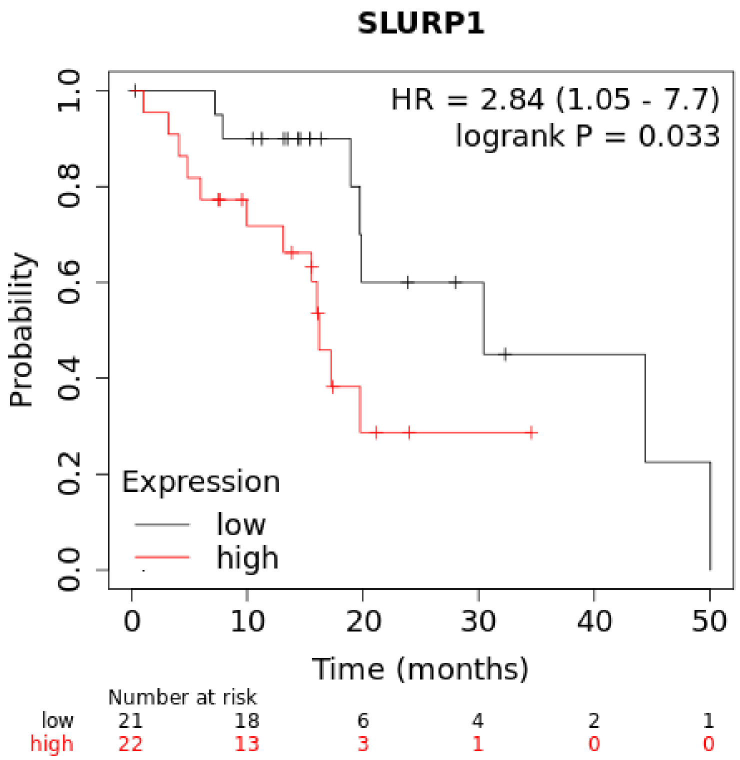

Click here to download the plot in TIFF format

Download plot as a PDF

Download p values vs. cutoff table

Median survival

Low expression cohort (months)	High expression cohort (months)
30.43	16.2

**RNAseq ID:**	LYPD2 -
**Survival:**	OS
**Auto select best cutoff:**	checked
**Follow up threshold:**	all
**Censore at threshold:**	checked
**Compute median over entire database:**	false
**Cutoff value used in analysis:**	28
**Expression range of the probe:**	0 - 1859
**Invert HR values below 1:**	not checked

Restrictions

Tumor type: Pancreatic ductal adenocarcinoma

Restrict analysis to subtypes...

Stage:	all
Gender:	all
Race:	all
Grade:	all
Mutation burden:	all

Restrict analysis based on cellular content...

Basophils:	all
B-cells:	all
CD4+ memory T-cells:	enriched
CD8+ T-cells:	all
Eosinophils:	all
Macrophages:	all
Mesenchymal stem cells:	all
Natural killer T-cells:	all
Regulatory T-cells:	all
Type 1 T-helper cells:	all
Type 2 T-helper cells:	all

Results

P value: 0.0854
FDR: 100%

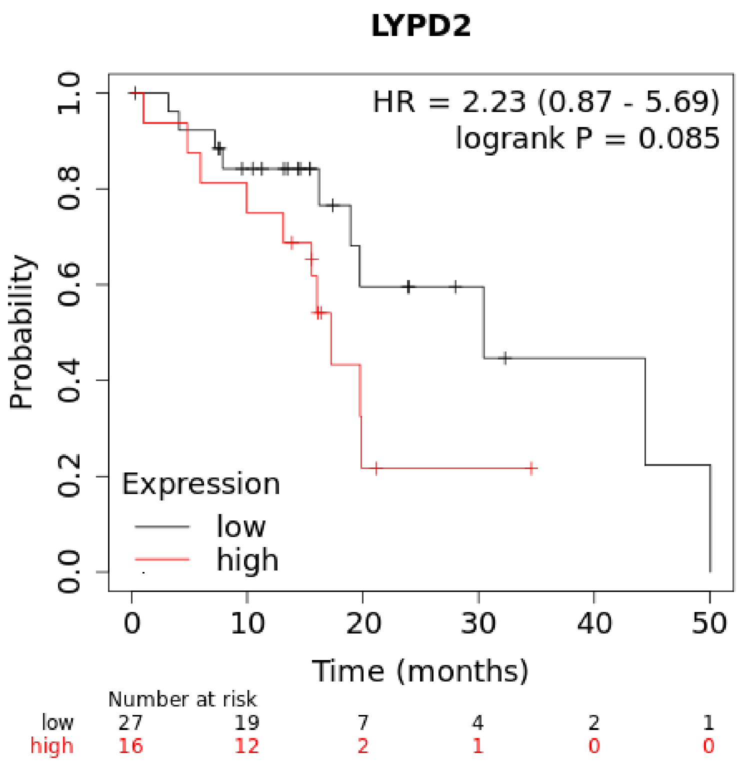

Click here to download the plot in TIFF format

Download plot as a PDF

Download p values vs. cutoff table

Median survival

Low expression cohort (months)	High expression cohort (months)
30.43	17.23

**RNAseq ID:**	LY6D -
**Survival:**	OS
**Auto select best cutoff:**	checked
**Follow up threshold:**	all
**Censore at threshold:**	checked
**Compute median over entire database:**	false
**Cutoff value used in analysis:**	188
**Expression range of the probe:**	0 - 4067
**Invert HR values below 1:**	not checked

Restrictions

Tumor type: Pancreatic ductal adenocarcinoma

Restrict analysis to subtypes...

Stage:	all
Gender:	all
Race:	all
Grade:	all
Mutation burden:	all

Restrict analysis based on cellular content...

Basophils:	all

B-cells:	all
CD4+ memory T-cells:	enriched
CD8+ T-cells:	all
Eosinophils:	all
Macrophages:	all
Mesenchymal stem cells:	all
Natural killer T-cells:	all
Regulatory T-cells:	all
Type 1 T-helper cells:	all
Type 2 T-helper cells:	all

Results

P value: 4.8e-5
FDR: 1%

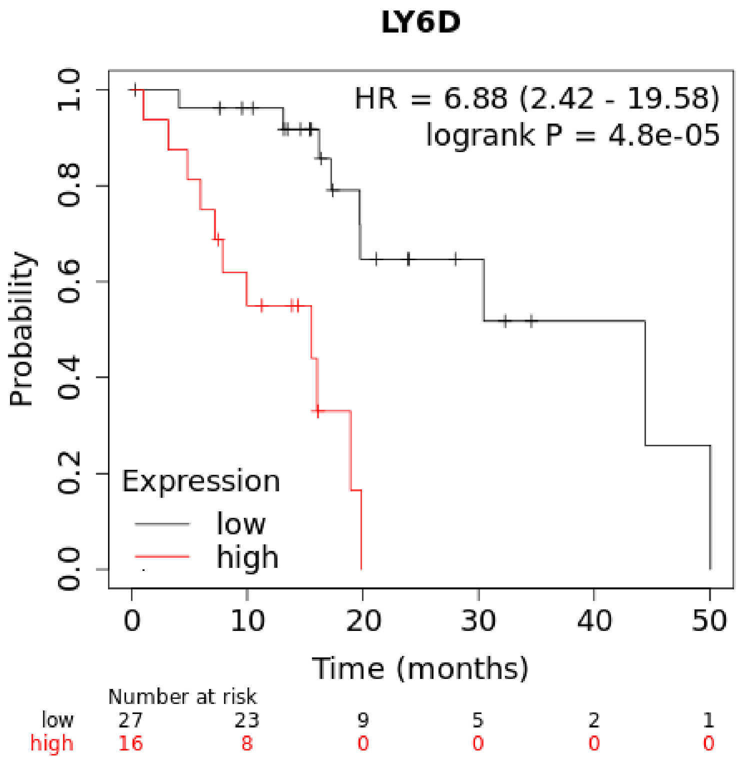

Click here to download the plot in TIFF format

Download plot as a PDF

Download p values vs. cutoff table

Median survival

Low expression cohort (months)	High expression cohort (months)
44.4	15.57

**RNAseq ID:**	GML -
**Survival:**	OS
**Auto select best cutoff:**	checked
**Follow up threshold:**	all
**Censore at threshold:**	checked
**Compute median over entire database:**	false
**Cutoff value used in analysis:**	0
**Expression range of the probe:**	0 - 3
**Invert HR values below 1:**	not checked

Restrictions

Tumor type: Pancreatic ductal adenocarcinoma

Restrict analysis to subtypes...

Stage:	all
Gender:	all
Race:	all
Grade:	all
Mutation burden:	all

Restrict analysis based on cellular content...

Basophils:	all
B-cells:	all
CD4+ memory T-cells:	enriched
CD8+ T-cells:	all
Eosinophils:	all
Macrophages:	all
Mesenchymal stem cells:	all
Natural killer T-cells:	all
Regulatory T-cells:	all
Type 1 T-helper cells:	all
Type 2 T-helper cells:	all

Results

P value: 0.1008
FDR: 100%

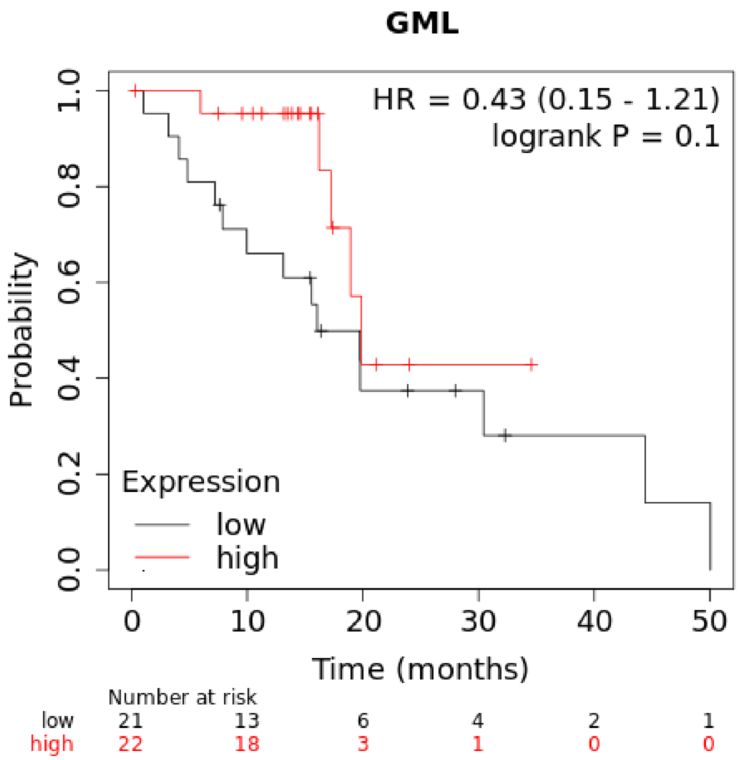

Click here to download the plot in TIFF format

Download plot as a PDF

Download p values vs. cutoff table

Median survival

Low expression cohort (months)	High expression cohort (months)
16.03	19.87

**RNAseq ID:**	LY6E -
**Survival:**	OS
**Auto select best cutoff:**	checked
**Follow up threshold:**	all
**Censore at threshold:**	checked
**Compute median over entire database:**	false
**Cutoff value used in analysis:**	7890
**Expression range of the probe:**	1984 - 32064
**Invert HR values below 1:**	not checked

Restrictions

Tumor type: Pancreatic ductal adenocarcinoma

Restrict analysis to subtypes...

Stage:	all
Gender:	all
Race:	all
Grade:	all
Mutation burden:	all

Restrict analysis based on cellular content...

Basophils:	all
B-cells:	all
CD4+ memory T-cells:	enriched
CD8+ T-cells:	all
Eosinophils:	all
Macrophages:	all
Mesenchymal stem cells:	all
Natural killer T-cells:	all
Regulatory T-cells:	all
Type 1 T-helper cells:	all
Type 2 T-helper cells:	all

Results

P value: 0.0012
FDR: 10%

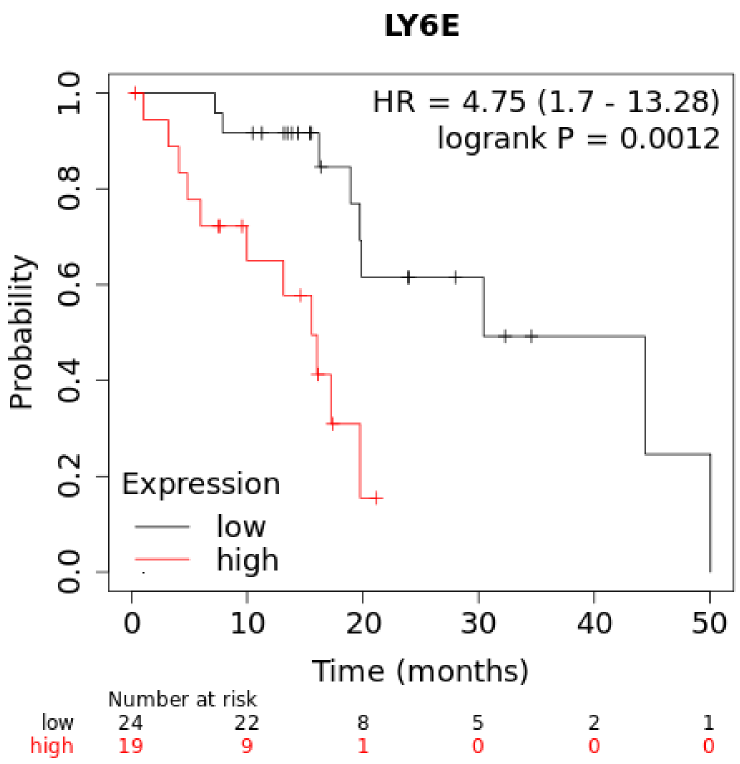

Click here to download the plot in TIFF format

Download plot as a PDF

Download p values vs. cutoff table

Median survival

Low expression cohort (months)	High expression cohort (months)
30.43	15.57

RNAseq ID: LY6L -
Survival: OS
Auto select best cutoff: checked
Follow up threshold: all
Censore at threshold: checked
Compute median over entire database: false
Cutoff value used in analysis: 0
Expression range of the probe: 0 - 8
Invert HR values below 1: not checked

Restrictions

Tumor type: Pancreatic ductal adenocarcinoma

Restrict analysis to subtypes...

Stage: all
Gender: all
Race: all
Grade: all
Mutation burden: all

Restrict analysis based on cellular content...

Basophils: all

B-cells:	all
CD4+ memory T-cells:	enriched
CD8+ T-cells:	all
Eosinophils:	all
Macrophages:	all
Mesenchymal stem cells:	all
Natural killer T-cells:	all
Regulatory T-cells:	all
Type 1 T-helper cells:	all
Type 2 T-helper cells:	all

Results

P value: 0.0037
FDR: 20%

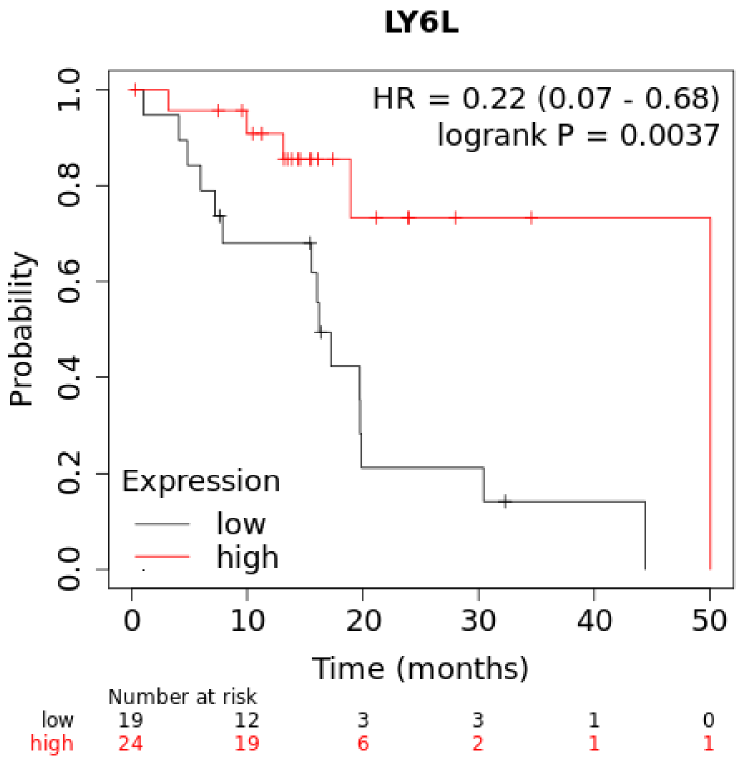

Click here to download the plot in TIFF format

Download plot as a PDF

Download p values vs. cutoff table

Median survival

Low expression cohort (months)	High expression cohort (months)
16.2	50.07

**RNAseq ID:**	LY6H -
**Survival:**	OS
**Auto select best cutoff:**	checked
**Follow up threshold:**	all
**Censore at threshold:**	checked
**Compute median over entire database:**	false
**Cutoff value used in analysis:**	14
**Expression range of the probe:**	2 - 147
**Invert HR values below 1:**	not checked

Restrictions

Tumor type: Pancreatic ductal adenocarcinoma

Restrict analysis to subtypes...

Stage:	all
Gender:	all
Race:	all
Grade:	all
Mutation burden:	all

Restrict analysis based on cellular content...

Basophils:	all
B-cells:	all
CD4+ memory T-cells:	enriched
CD8+ T-cells:	all
Eosinophils:	all
Macrophages:	all
Mesenchymal stem cells:	all
Natural killer T-cells:	all
Regulatory T-cells:	all
Type 1 T-helper cells:	all
Type 2 T-helper cells:	all

Results

P value: 0.214
FDR: 100%

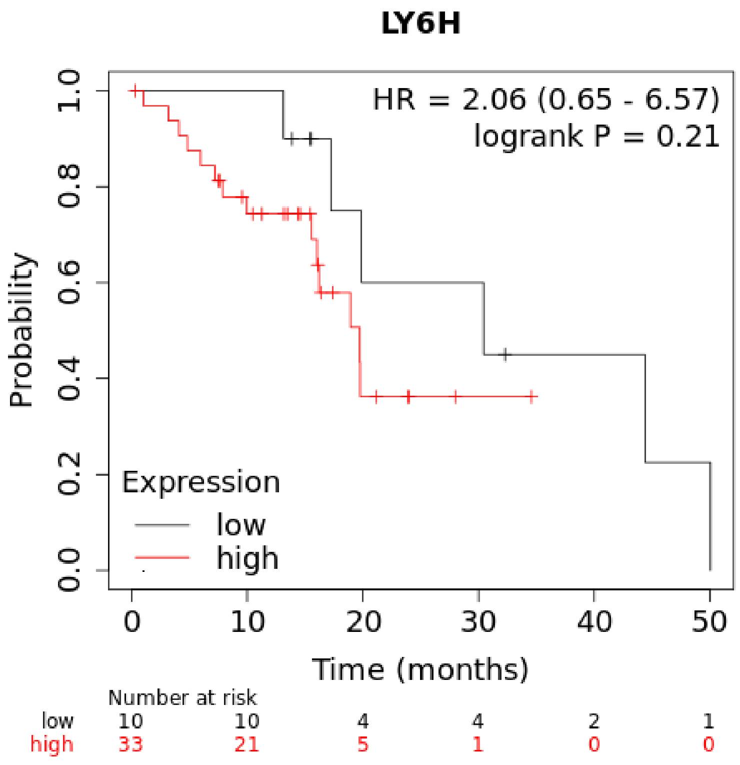

Click here to download the plot in TIFF format

Download plot as a PDF

Download p values vs. cutoff table

Median survival

Low expression cohort (months)	High expression cohort (months)
30.43	19.73

**RNAseq ID:**	GPIHBP1 -
**Survival:**	OS
**Auto select best cutoff:**	checked
**Follow up threshold:**	all
**Censore at threshold:**	checked
**Compute median over entire database:**	false
**Cutoff value used in analysis:**	125
**Expression range of the probe:**	11 - 323
**Invert HR values below 1:**	not checked

Restrictions

Tumor type: Pancreatic ductal adenocarcinoma

Restrict analysis to subtypes...

Stage:	all
Gender:	all
Race:	all
Grade:	all
Mutation burden:	all

Restrict analysis based on cellular content...

Basophils:	all
B-cells:	all
CD4+ memory T-cells:	enriched
CD8+ T-cells:	all
Eosinophils:	all
Macrophages:	all
Mesenchymal stem cells:	all
Natural killer T-cells:	all
Regulatory T-cells:	all
Type 1 T-helper cells:	all
Type 2 T-helper cells:	all

Results

**P value:**	0.0269
**FDR:**	over 50%

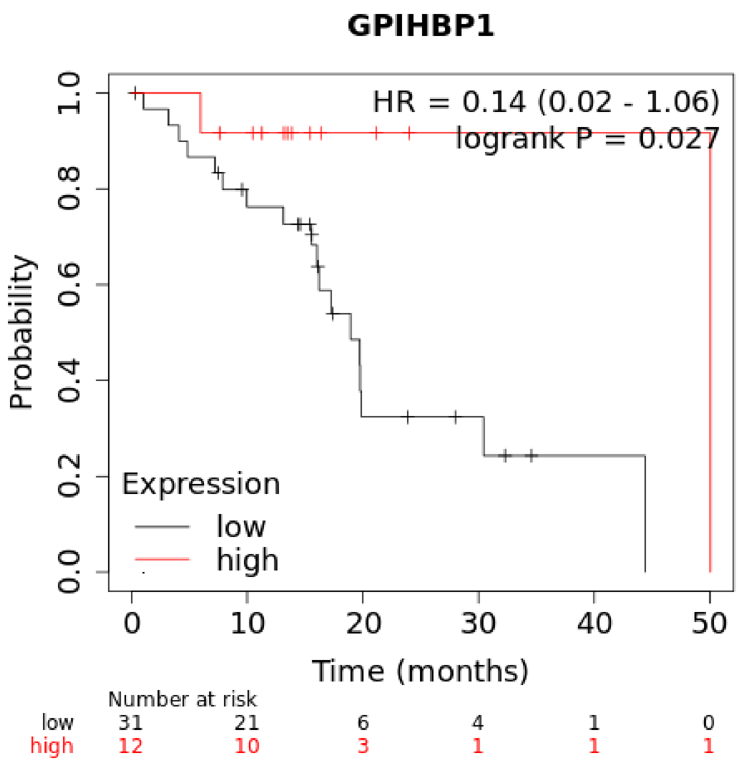

Click here to download the plot in TIFF format

Download plot as a PDF

Download p values vs. cutoff table

Median survival

Low expression cohort (months)	High expression cohort (months)
18.93	50.07

RNAseq ID: LYPD4 -
Survival: OS
Auto select best cutoff: checked
Follow up threshold: all
Censore at threshold: checked
Compute median over entire database: false
Cutoff value used in analysis: 0
Expression range of the probe: 0 - 8
Invert HR values below 1: not checked

Restrictions

Tumor type: Pancreatic ductal adenocarcinoma

Restrict analysis to subtypes...

Stage: all
Gender: all
Race: all
Grade: all
Mutation burden: all

Restrict analysis based on cellular content...

Basophils: all

B-cells:	all
CD4+ memory T-cells:	enriched
CD8+ T-cells:	all
Eosinophils:	all
Macrophages:	all
Mesenchymal stem cells:	all
Natural killer T-cells:	all
Regulatory T-cells:	all
Type 1 T-helper cells:	all
Type 2 T-helper cells:	all

Results

P value: 0.0038
FDR: 20%

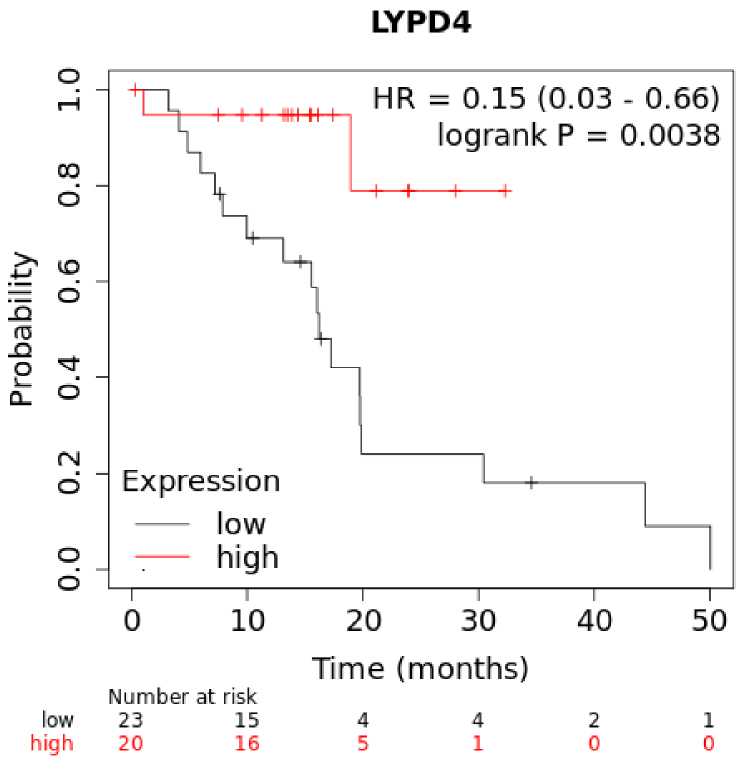

Click here to download the plot in TIFF format

Download plot as a PDF

Download p values vs. cutoff table

Median survival

Low expression cohort (months)	High expression cohort (months)
NA	NA

**RNAseq ID:**	CD177 -
**Survival:**	OS
**Auto select best cutoff:**	checked
**Follow up threshold:**	all
**Censore at threshold:**	checked
**Compute median over entire database:**	false
**Cutoff value used in analysis:**	54
**Expression range of the probe:**	1 - 1205
**Invert HR values below 1:**	not checked

Restrictions

Tumor type: Pancreatic ductal adenocarcinoma

Restrict analysis to subtypes...

Stage:	all
Gender:	all
Race:	all
Grade:	all
Mutation burden:	all

Restrict analysis based on cellular content...

Basophils:	all
B-cells:	all
CD4+ memory T-cells:	enriched
CD8+ T-cells:	all
Eosinophils:	all
Macrophages:	all
Mesenchymal stem cells:	all
Natural killer T-cells:	all
Regulatory T-cells:	all
Type 1 T-helper cells:	all
Type 2 T-helper cells:	all

Results

P value: 0.3226
FDR: 100%

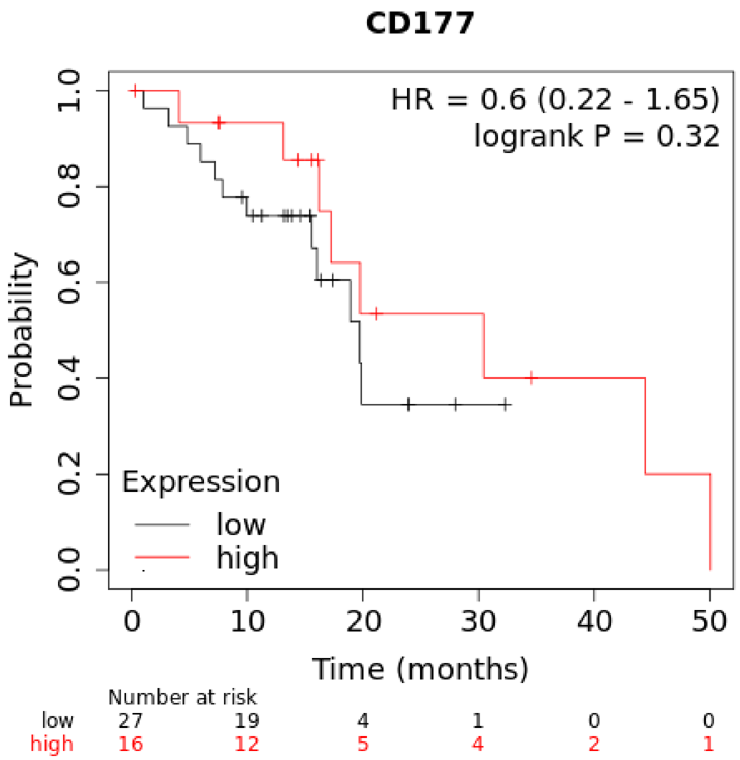

Click here to download the plot in TIFF format

Download plot as a PDF

Download p values vs. cutoff table

Median survival

Low expression cohort (months)	High expression cohort (months)
19.73	30.43

**RNAseq ID:**	TEX101 -
**Survival:**	OS
**Auto select best cutoff:**	checked
**Follow up threshold:**	all
**Censore at threshold:**	checked
**Compute median over entire database:**	false
**Cutoff value used in analysis:**	1
**Expression range of the probe:**	0 - 20
**Invert HR values below 1:**	not checked

Restrictions

Tumor type: Pancreatic ductal adenocarcinoma

Restrict analysis to subtypes...

Stage:	all
Gender:	all
Race:	all
Grade:	all
Mutation burden:	all

Restrict analysis based on cellular content...

Basophils:	all
B-cells:	all
CD4+ memory T-cells:	enriched
CD8+ T-cells:	all
Eosinophils:	all
Macrophages:	all
Mesenchymal stem cells:	all
Natural killer T-cells:	all
Regulatory T-cells:	all
Type 1 T-helper cells:	all
Type 2 T-helper cells:	all

Results

**P value:**	0.2285
**FDR:**	100%

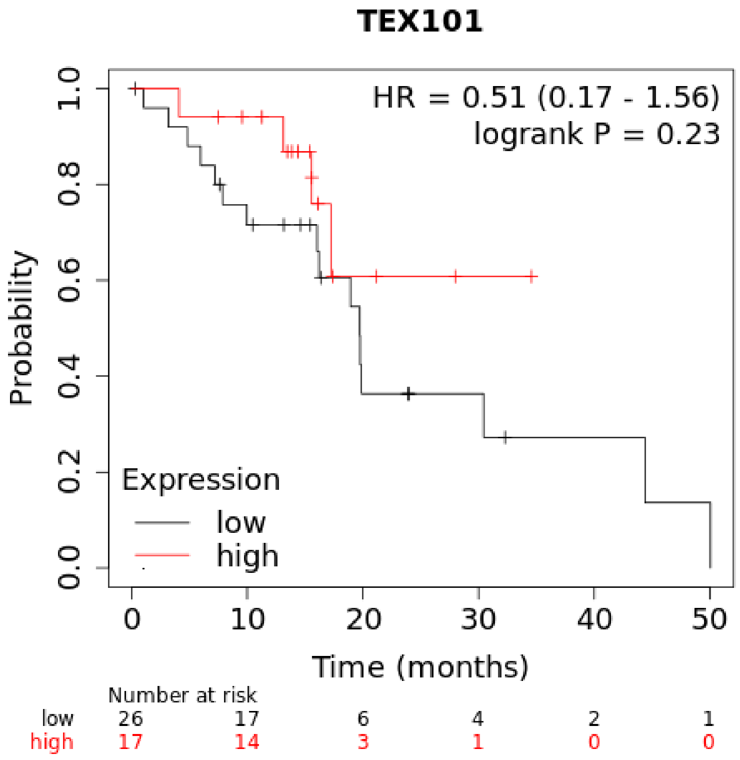

Click here to download the plot in TIFF format

Download plot as a PDF

Download p values vs. cutoff table

Upper quartile survival

Low expression cohort (months)	High expression cohort (months)
9.97	17.23

**RNAseq ID:**	LYPD3 -
**Survival:**	OS
**Auto select best cutoff:**	checked
**Follow up threshold:**	all
**Censore at threshold:**	checked
**Compute median over entire database:**	false
**Cutoff value used in analysis:**	125
**Expression range of the probe:**	19 - 1869
**Invert HR values below 1:**	not checked

Restrictions

Tumor type: Pancreatic ductal adenocarcinoma

Restrict analysis to subtypes...

Stage:	all
Gender:	all
Race:	all
Grade:	all
Mutation burden:	all

Restrict analysis based on cellular content...

Basophils:	all

B-cells:	all
CD4+ memory T-cells:	enriched
CD8+ T-cells:	all
Eosinophils:	all
Macrophages:	all
Mesenchymal stem cells:	all
Natural killer T-cells:	all
Regulatory T-cells:	all
Type 1 T-helper cells:	all
Type 2 T-helper cells:	all

Results

P value: 0.4669
FDR: 100%

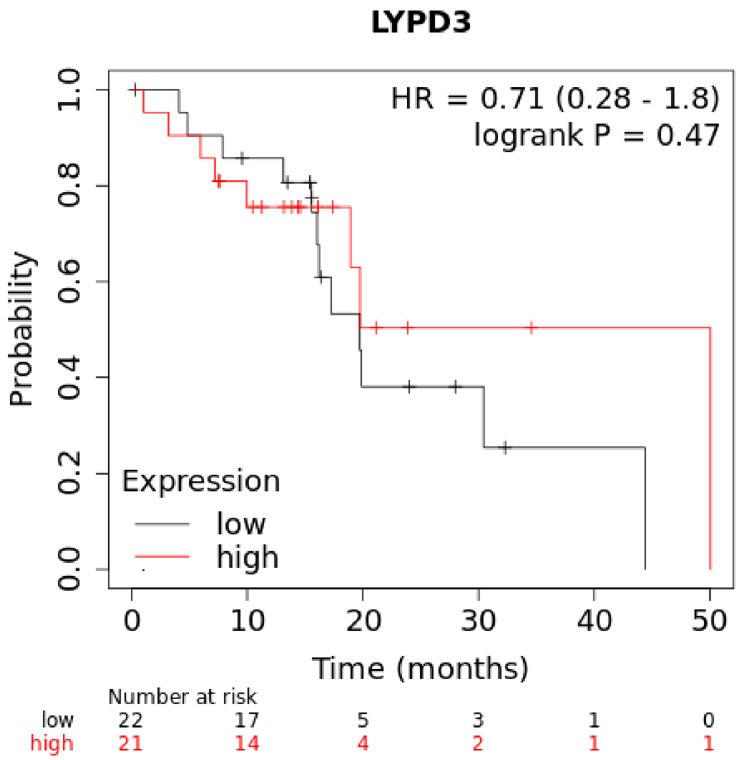

Click here to download the plot in TIFF format

Download plot as a PDF

Download p values vs. cutoff table

Median survival

Low expression cohort (months)	High expression cohort (months)
19.73	50.07

**RNAseq ID:**	PINLYP -
**Survival:**	OS
**Auto select best cutoff:**	checked
**Follow up threshold:**	all
**Censore at threshold:**	checked
**Compute median over entire database:**	false
**Cutoff value used in analysis:**	48
**Expression range of the probe:**	22 - 387
**Invert HR values below 1:**	not checked

Restrictions

Tumor type: Pancreatic ductal adenocarcinoma

Restrict analysis to subtypes...

Stage: all
Gender: all
Race: all
Grade: all
Mutation burden: all

Restrict analysis based on cellular content...

Basophils: all
B-cells: all
CD4+ memory T-cells: enriched
CD8+ T-cells: all
Eosinophils: all
Macrophages: all
Mesenchymal stem cells: all
Natural killer T-cells: all
Regulatory T-cells: all
Type 1 T-helper cells: all
Type 2 T-helper cells: all

Results

P value: 0.0942
FDR: 100%

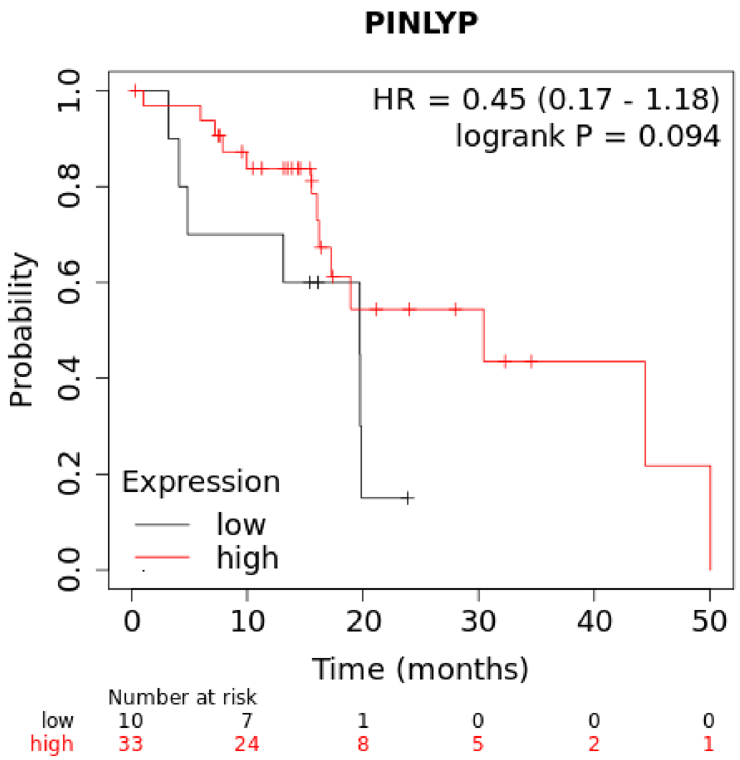

Click here to download the plot in TIFF format

Download plot as a PDF

Download p values vs. cutoff table

Median survival

Low expression cohort (months)	High expression cohort (months)
19.73	30.43

**RNAseq ID:**	PLAUR -
**Survival:**	OS
**Auto select best cutoff:**	checked
**Follow up threshold:**	all
**Censore at threshold:**	checked
**Compute median over entire database:**	false
**Cutoff value used in analysis:**	2625
**Expression range of the probe:**	753 - 7680
**Invert HR values below 1:**	not checked

Restrictions

Tumor type: Pancreatic ductal adenocarcinoma

Restrict analysis to subtypes...

Stage:	all
Gender:	all
Race:	all
Grade:	all
Mutation burden:	all

Restrict analysis based on cellular content...

Basophils:	all
B-cells:	all
CD4+ memory T-cells:	enriched
CD8+ T-cells:	all
Eosinophils:	all
Macrophages:	all
Mesenchymal stem cells:	all
Natural killer T-cells:	all
Regulatory T-cells:	all
Type 1 T-helper cells:	all
Type 2 T-helper cells:	all

Results

**P value:**	0.1655
**FDR:**	100%

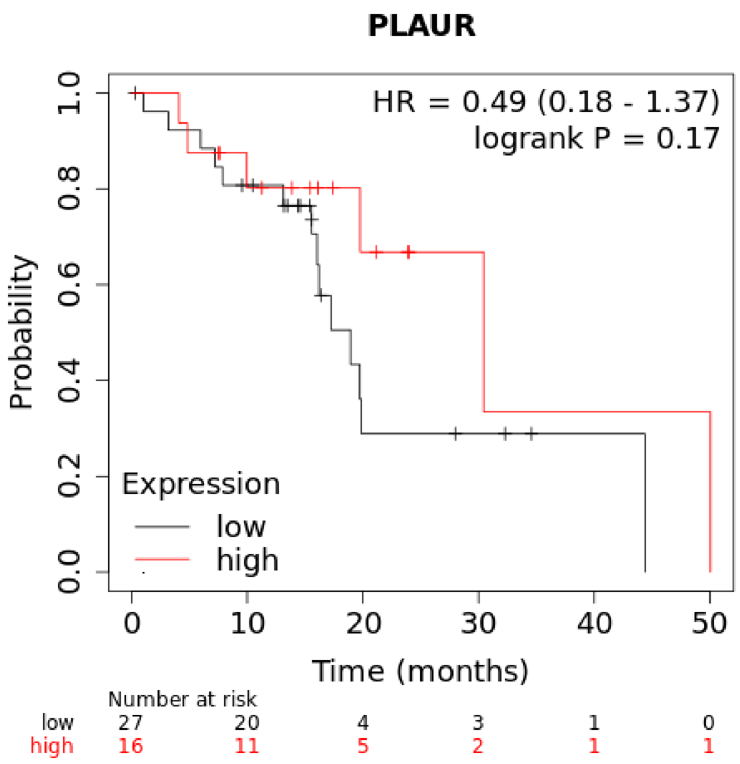

Click here to download the plot in TIFF format

Download plot as a PDF

Download p values vs. cutoff table

Median survival

Low expression cohort (months)	High expression cohort (months)
18.93	30.43

**RNAseq ID:**	LYPD5 -
**Survival:**	OS
**Auto select best cutoff:**	checked
**Follow up threshold:**	all
**Censore at threshold:**	checked
**Compute median over entire database:**	false
**Cutoff value used in analysis:**	102
**Expression range of the probe:**	31 - 578
**Invert HR values below 1:**	not checked

Restrictions

Tumor type: Pancreatic ductal adenocarcinoma

Restrict analysis to subtypes...

Stage:	all
Gender:	all
Race:	all
Grade:	all
Mutation burden:	all

Restrict analysis based on cellular content...

Basophils:	all

B-cells:	all
CD4+ memory T-cells:	enriched
CD8+ T-cells:	all
Eosinophils:	all
Macrophages:	all
Mesenchymal stem cells:	all
Natural killer T-cells:	all
Regulatory T-cells:	all
Type 1 T-helper cells:	all
Type 2 T-helper cells:	all

Results

P value: 0.0142
FDR: over 50%

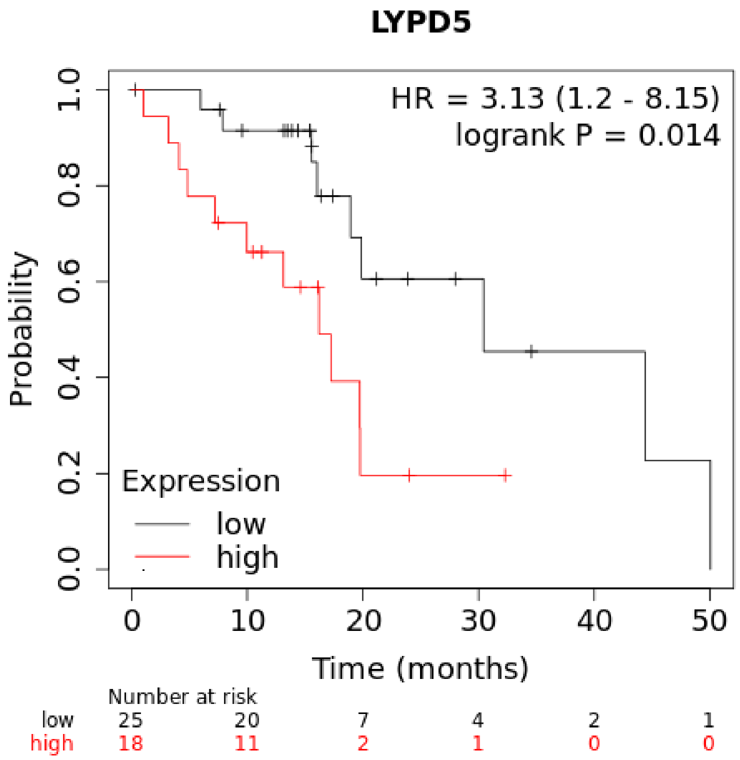

Click here to download the plot in TIFF format

Download plot as a PDF

Download p values vs. cutoff table

Median survival

Low expression cohort (months)	High expression cohort (months)
30.43	16.2

**RNAseq ID:**	SPACA4 -
**Survival:**	OS
**Auto select best cutoff:**	checked
**Follow up threshold:**	all
**Censore at threshold:**	checked
**Compute median over entire database:**	false
**Cutoff value used in analysis:**	6
**Expression range of the probe:**	1 - 34
**Invert HR values below 1:**	not checked

Restrictions

Tumor type: Pancreatic ductal adenocarcinoma

Restrict analysis to subtypes...

Stage:	all
Gender:	all
Race:	all
Grade:	all
Mutation burden:	all

Restrict analysis based on cellular content...

Basophils:	all
B-cells:	all
CD4+ memory T-cells:	enriched
CD8+ T-cells:	all
Eosinophils:	all
Macrophages:	all
Mesenchymal stem cells:	all
Natural killer T-cells:	all
Regulatory T-cells:	all
Type 1 T-helper cells:	all
Type 2 T-helper cells:	all

Results

P value: 0.0327
FDR: over 50%

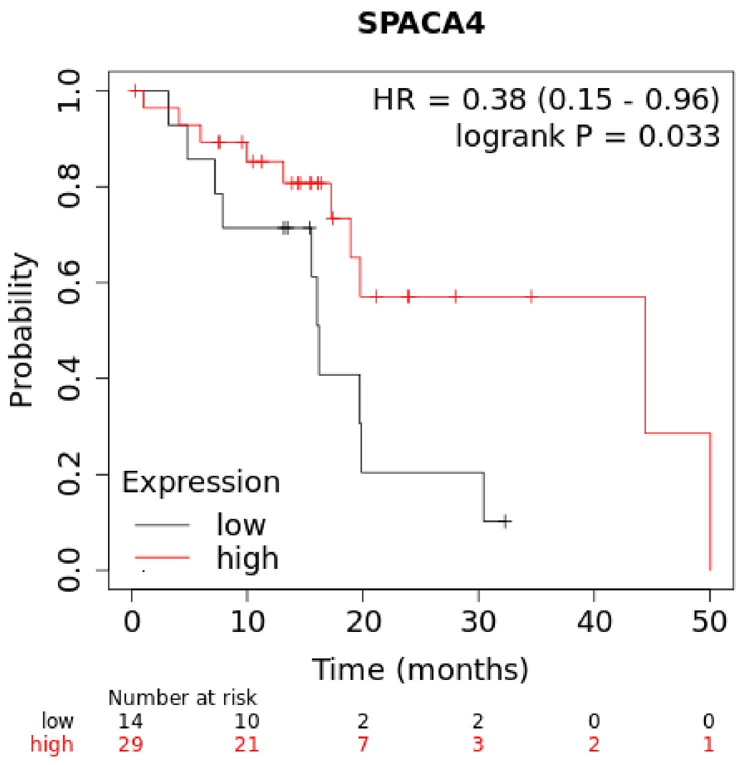

Click here to download the plot in TIFF format

Download plot as a PDF

Download p values vs. cutoff table

Median survival

Low expression cohort (months)	High expression cohort (months)
16.2	44.4

**RNAseq ID:**	ACRV1	-
**Survival:**	OS	
**Auto select best cutoff:**	checked	
**Follow up threshold:**	all	
**Censore at threshold:**	checked	
**Compute median over entire database:**	false	
**Cutoff value used in analysis:**	3	
**Expression range of the probe:**	0 - 15	
**Invert HR values below 1:**	not checked	

Restrictions

Tumor type: Pancreatic ductal adenocarcinoma

Restrict analysis to subtypes...

Stage:	all
Gender:	all
Race:	all
Grade:	all
Mutation burden:	all

Restrict analysis based on cellular content...

Basophils:	all
B-cells:	all
CD4+ memory T-cells:	enriched
CD8+ T-cells:	all
Eosinophils:	all
Macrophages:	all
Mesenchymal stem cells:	all
Natural killer T-cells:	all
Regulatory T-cells:	all
Type 1 T-helper cells:	all
Type 2 T-helper cells:	all

Results

P value: 0.1074
FDR: 100%

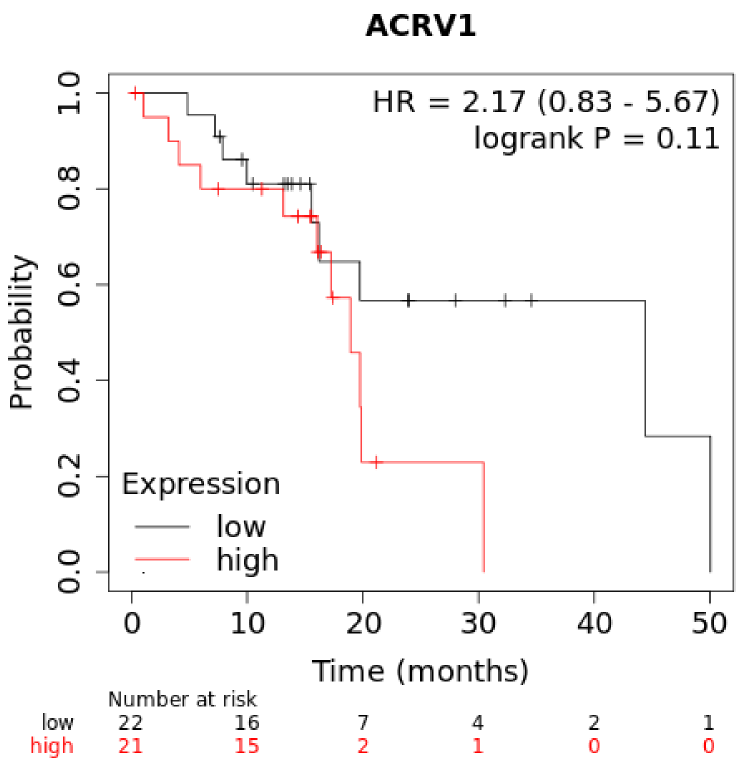

Click here to download the plot in TIFF format

Download plot as a PDF

Download p values vs. cutoff table

Median survival

Low expression cohort (months)	High expression cohort (months)
44.4	18.93

**RNAseq ID:**	PATE1 -
**Survival:**	OS
**Auto select best cutoff:**	checked
**Follow up threshold:**	all
**Censore at threshold:**	checked
**Compute median over entire database:**	false
**Cutoff value used in analysis:**	0
**Expression range of the probe:**	0 - 1
**Invert HR values below 1:**	not checked

Restrictions

Tumor type: Pancreatic ductal adenocarcinoma

Restrict analysis to subtypes...

Stage:	all
Gender:	all
Race:	all
Grade:	all
Mutation burden:	all

Restrict analysis based on cellular content...

Basophils:	all

B-cells:	all
CD4+ memory T-cells:	enriched
CD8+ T-cells:	all
Eosinophils:	all
Macrophages:	all
Mesenchymal stem cells:	all
Natural killer T-cells:	all
Regulatory T-cells:	all
Type 1 T-helper cells:	all
Type 2 T-helper cells:	all

Results

P value: 0.0465
FDR: over 50%

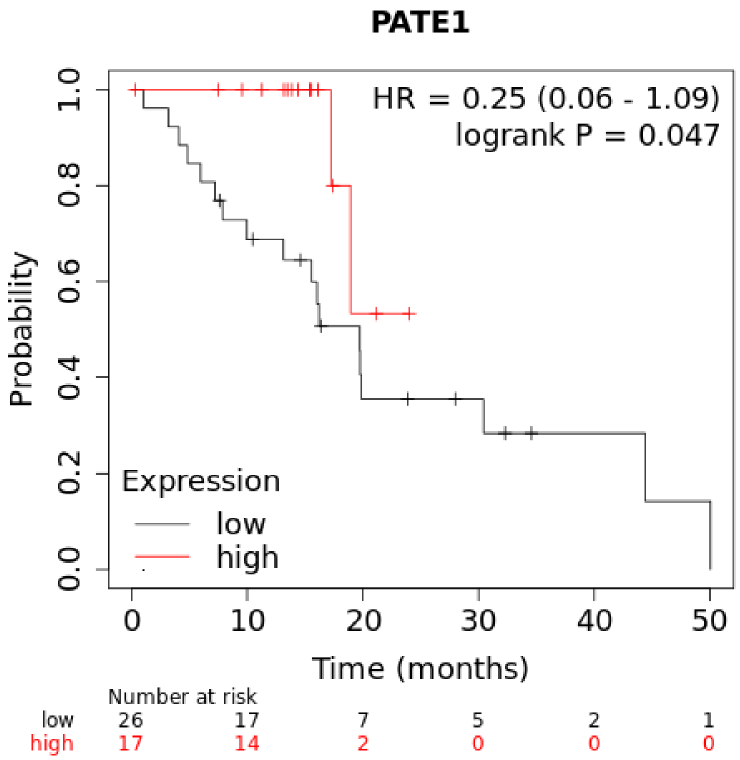

Click here to download the plot in TIFF format

Download plot as a PDF

Download p values vs. cutoff table

Upper quartile survival

Low expression cohort (months)	High expression cohort (months)
7.87	18.93

**RNAseq ID:**	PATE2 -
**Survival:**	OS
**Auto select best cutoff:**	checked
**Follow up threshold:**	all
**Censore at threshold:**	checked
**Compute median over entire database:**	false
**Cutoff value used in analysis:**	1
**Expression range of the probe:**	0 - 3
**Invert HR values below 1:**	not checked

Restrictions

Tumor type: Pancreatic ductal adenocarcinoma

Restrict analysis to subtypes...

Stage:	all
Gender:	all
Race:	all
Grade:	all
Mutation burden:	all

Restrict analysis based on cellular content...

Basophils:	all
B-cells:	all
CD4+ memory T-cells:	enriched
CD8+ T-cells:	all
Eosinophils:	all
Macrophages:	all
Mesenchymal stem cells:	all
Natural killer T-cells:	all
Regulatory T-cells:	all
Type 1 T-helper cells:	all
Type 2 T-helper cells:	all

Results

P value: 0.1275
FDR: 100%

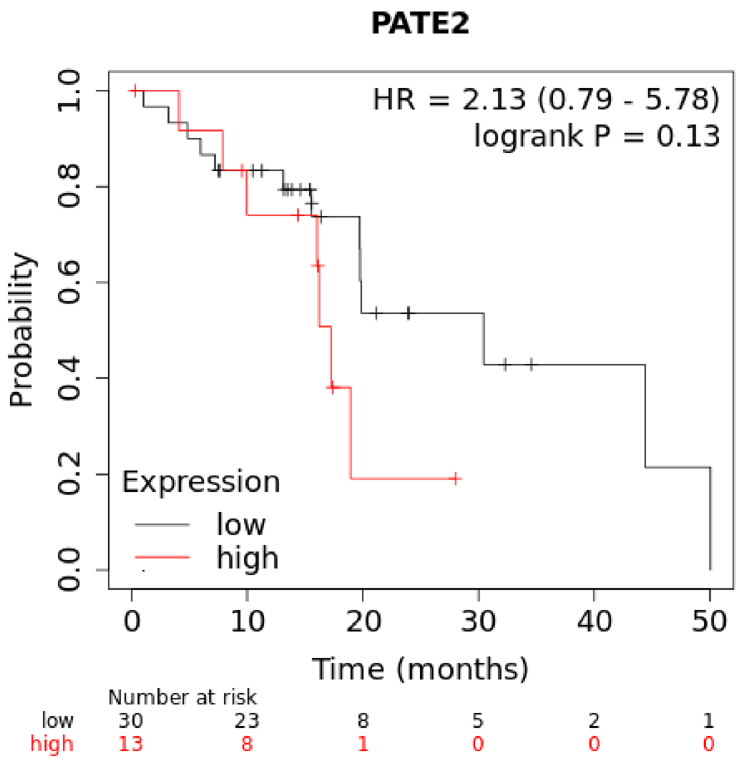

Click here to download the plot in TIFF format

Download plot as a PDF

Download p values vs. cutoff table

Median survival

Low expression cohort (months)	High expression cohort (months)
30.43	17.23

**RNAseq ID:**	PATE3 -
**Survival:**	OS
**Auto select best cutoff:**	checked
**Follow up threshold:**	all
**Censore at threshold:**	checked
**Compute median over entire database:**	false
**Cutoff value used in analysis:**	0
**Expression range of the probe:**	0 - 1
**Invert HR values below 1:**	not checked

Restrictions

Tumor type: Pancreatic ductal adenocarcinoma

Restrict analysis to subtypes...

Stage:	all
Gender:	all
Race:	all
Grade:	all
Mutation burden:	all

Restrict analysis based on cellular content...

Basophils:	all
B-cells:	all
CD4+ memory T-cells:	enriched
CD8+ T-cells:	all
Eosinophils:	all
Macrophages:	all
Mesenchymal stem cells:	all
Natural killer T-cells:	all
Regulatory T-cells:	all
Type 1 T-helper cells:	all
Type 2 T-helper cells:	all

Results

**P value:**	0.061
**FDR:**	100%

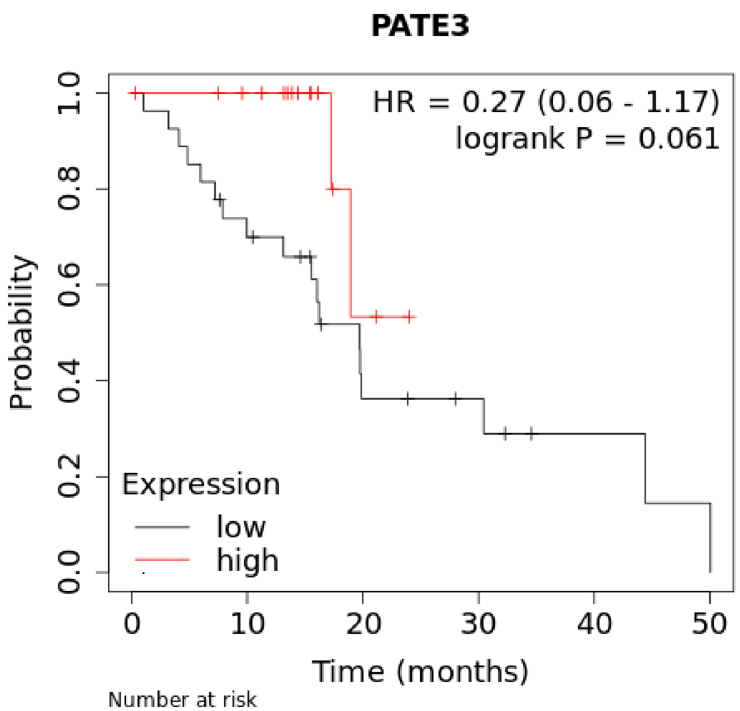

Number at risk

low	27	18	7	5	2	1
high	16	13	2	0	0	0

Click here to download the plot in TIFF format

Download plot as a PDF

Download p values vs. cutoff table

Upper quartile survival

Low expression cohort (months)	High expression cohort (months)
7.87	18.93

**RNAseq ID:**	PATE4 -
**Survival:**	OS
**Auto select best cutoff:**	checked
**Follow up threshold:**	all
**Censore at threshold:**	checked
**Compute median over entire database:**	false
**Cutoff value used in analysis:**	0
**Expression range of the probe:**	0 - 2
**Invert HR values below 1:**	not checked

Restrictions

Tumor type: Pancreatic ductal adenocarcinoma

Restrict analysis to subtypes...

Stage:	all
Gender:	all
Race:	all
Grade:	all
Mutation burden:	all

Restrict analysis based on cellular content...

Basophils:	all

B-cells:	all
CD4+ memory T-cells:	enriched
CD8+ T-cells:	all
Eosinophils:	all
Macrophages:	all
Mesenchymal stem cells:	all
Natural killer T-cells:	all
Regulatory T-cells:	all
Type 1 T-helper cells:	all
Type 2 T-helper cells:	all

Results

P value: 0.1227
FDR: 100%

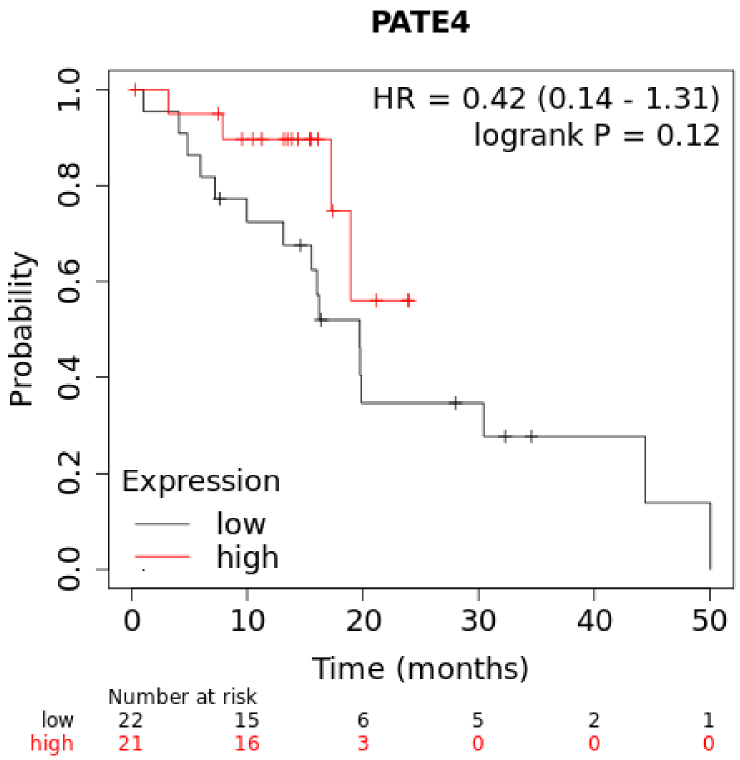

Click here to download the plot in TIFF format

Download plot as a PDF

Download p values vs. cutoff table

Upper quartile survival

Low expression cohort (months)	High expression cohort (months)
9.97	17.23

**RNAseq ID:**	CD59 -
**Survival:**	OS
**Auto select best cutoff:**	checked
**Follow up threshold:**	all
**Censore at threshold:**	checked
**Compute median over entire database:**	false
**Cutoff value used in analysis:**	17172
**Expression range of the probe:**	5969 - 27211
**Invert HR values below 1:**	not checked

Restrictions

Tumor type: Pancreatic ductal adenocarcinoma

Restrict analysis to subtypes...

Stage:	all
Gender:	all
Race:	all
Grade:	all
Mutation burden:	all

Restrict analysis based on cellular content...

Basophils:	all
B-cells:	all
CD4+ memory T-cells:	enriched
CD8+ T-cells:	all
Eosinophils:	all
Macrophages:	all
Mesenchymal stem cells:	all
Natural killer T-cells:	all
Regulatory T-cells:	all
Type 1 T-helper cells:	all
Type 2 T-helper cells:	all

Results

P value: 0.1934
FDR: 100%

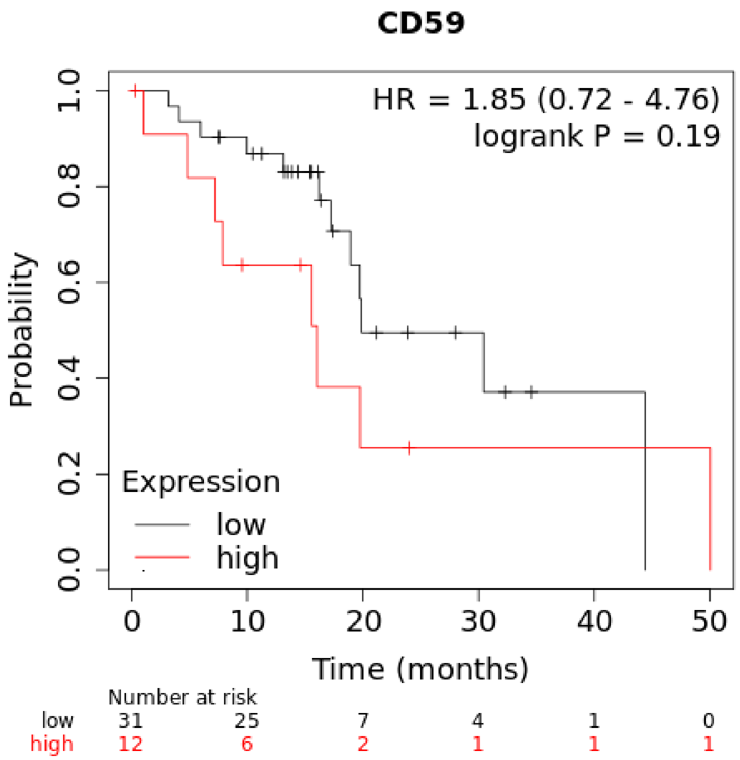

Click here to download the plot in TIFF format

Download plot as a PDF

Download p values vs. cutoff table

Median survival

Low expression cohort (months)	High expression cohort (months)
19.87	16.03

**RNAseq ID:**	LY6G6C -
**Survival:**	OS
**Auto select best cutoff:**	checked
**Follow up threshold:**	all
**Censore at threshold:**	checked
**Compute median over entire database:**	false
**Cutoff value used in analysis:**	2
**Expression range of the probe:**	0 - 140
**Invert HR values below 1:**	not checked

Restrictions

Tumor type: Pancreatic ductal adenocarcinoma

Restrict analysis to subtypes...

Stage:	all
Gender:	all
Race:	all
Grade:	all
Mutation burden:	all

Restrict analysis based on cellular content...

Basophils:	all
B-cells:	all
CD4+ memory T-cells:	enriched
CD8+ T-cells:	all
Eosinophils:	all
Macrophages:	all
Mesenchymal stem cells:	all
Natural killer T-cells:	all
Regulatory T-cells:	all
Type 1 T-helper cells:	all
Type 2 T-helper cells:	all

Results

**P value:**	0.4561
**FDR:**	100%

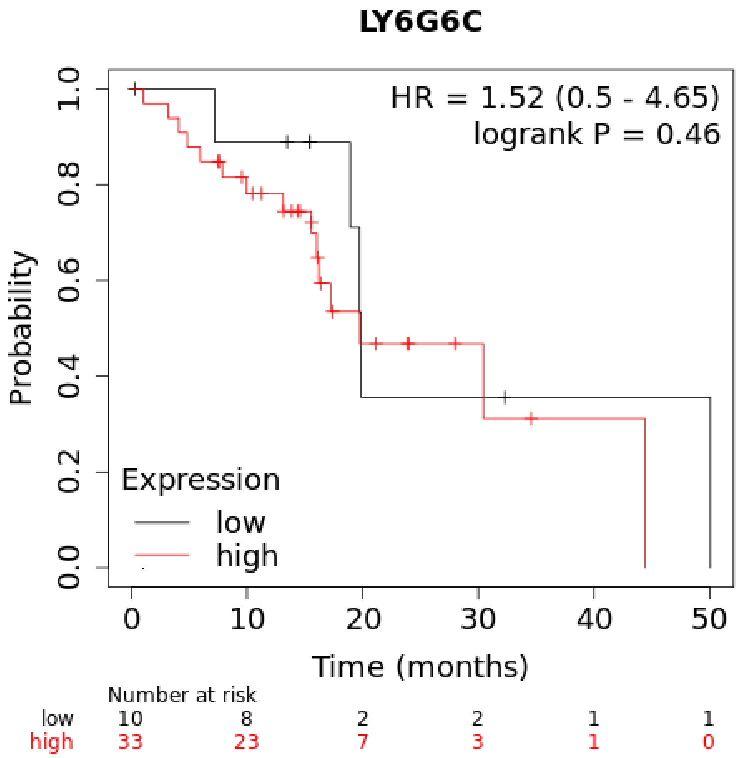

Click here to download the plot in TIFF format

Download plot as a PDF

Download p values vs. cutoff table

Median survival

Low expression cohort (months)	High expression cohort (months)
19.87	19.77

**RNAseq ID:**	LY6G6D -
**Survival:**	OS
**Auto select best cutoff:**	checked
**Follow up threshold:**	all
**Censore at threshold:**	checked
**Compute median over entire database:**	false
**Cutoff value used in analysis:**	0
**Expression range of the probe:**	0 - 1
**Invert HR values below 1:**	not checked

Restrictions

Tumor type: Pancreatic ductal adenocarcinoma

Restrict analysis to subtypes...

Stage:	all
Gender:	all
Race:	all
Grade:	all
Mutation burden:	all

Restrict analysis based on cellular content...

Basophils:	all

B-cells:	all
CD4+ memory T-cells:	enriched
CD8+ T-cells:	all
Eosinophils:	all
Macrophages:	all
Mesenchymal stem cells:	all
Natural killer T-cells:	all
Regulatory T-cells:	all
Type 1 T-helper cells:	all
Type 2 T-helper cells:	all

Results

P value: 0.1351
FDR: 100%

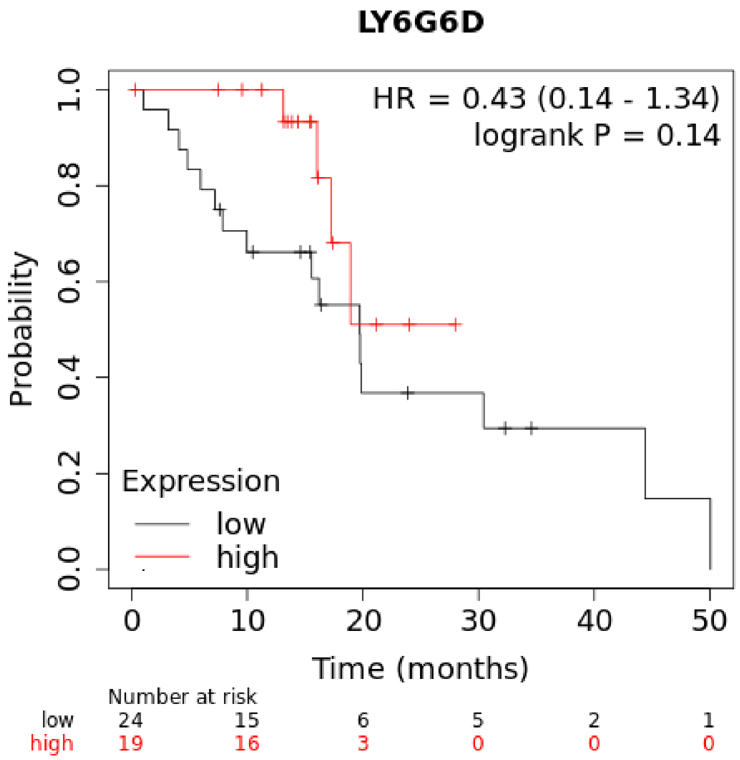

Click here to download the plot in TIFF format

Download plot as a PDF

Download p values vs. cutoff table

Upper quartile survival

Low expression cohort (months)	High expression cohort (months)
7.2	17.23

**RNAseq ID:**	LY6G6F -
**Survival:**	OS
**Auto select best cutoff:**	checked
**Follow up threshold:**	all
**Censore at threshold:**	checked
**Compute median over entire database:**	false
**Cutoff value used in analysis:**	0
**Expression range of the probe:**	0 - 1
**Invert HR values below 1:**	not checked

Restrictions

Tumor type: Pancreatic ductal adenocarcinoma

Restrict analysis to subtypes...

Stage:	all
Gender:	all
Race:	all
Grade:	all
Mutation burden:	all

Restrict analysis based on cellular content...

Basophils:	all
B-cells:	all
CD4+ memory T-cells:	enriched
CD8+ T-cells:	all
Eosinophils:	all
Macrophages:	all
Mesenchymal stem cells:	all
Natural killer T-cells:	all
Regulatory T-cells:	all
Type 1 T-helper cells:	all
Type 2 T-helper cells:	all

Results

P value: 0.0102
FDR: 50%

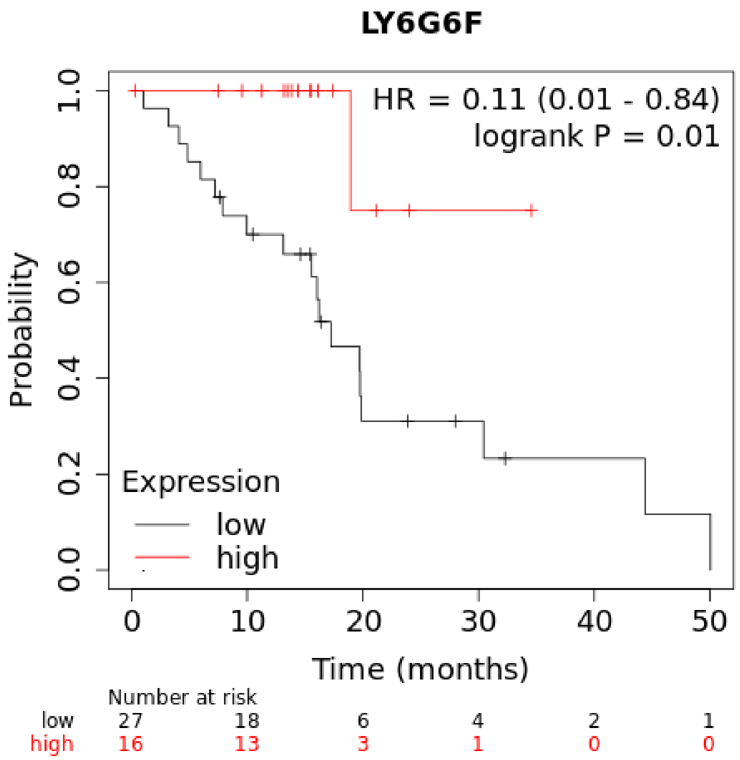

Click here to download the plot in TIFF format

Download plot as a PDF

Download p values vs. cutoff table

Median survival

Low expression cohort (months)	High expression cohort (months)
NA	NA

**RNAseq ID:**	LY6G5C -
**Survival:**	OS
**Auto select best cutoff:**	checked
**Follow up threshold:**	all
**Censore at threshold:**	checked
**Compute median over entire database:**	false
**Cutoff value used in analysis:**	42
**Expression range of the probe:**	24 - 93
**Invert HR values below 1:**	not checked

Restrictions

Tumor type: Pancreatic ductal adenocarcinoma

Restrict analysis to subtypes...

Stage:	all
Gender:	all
Race:	all
Grade:	all
Mutation burden:	all

Restrict analysis based on cellular content...

Basophils:	all
B-cells:	all
CD4+ memory T-cells:	enriched
CD8+ T-cells:	all
Eosinophils:	all
Macrophages:	all
Mesenchymal stem cells:	all
Natural killer T-cells:	all
Regulatory T-cells:	all
Type 1 T-helper cells:	all
Type 2 T-helper cells:	all

Results

**P value:**	0.277
**FDR:**	100%

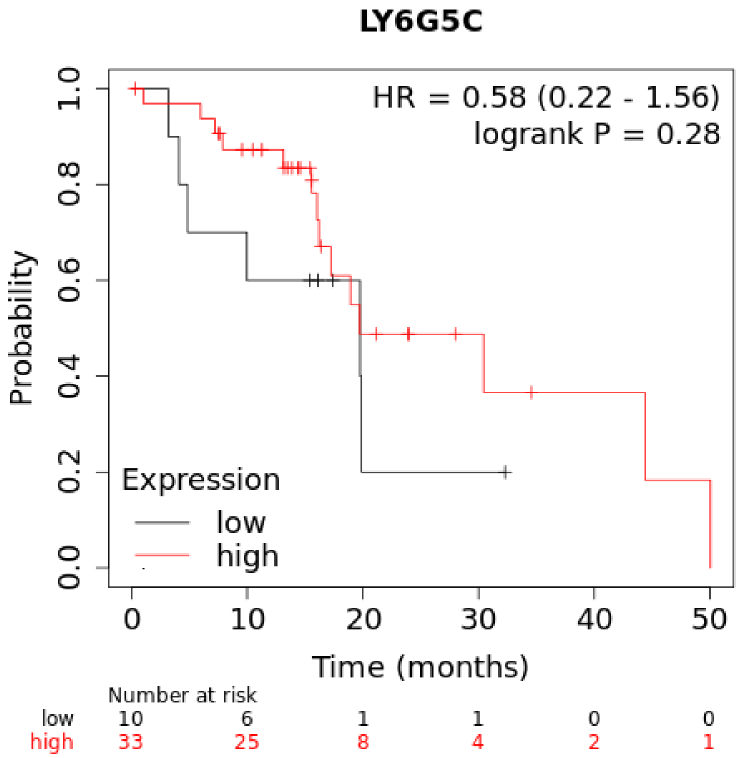

Click here to download the plot in TIFF format

Download plot as a PDF

Download p values vs. cutoff table

Median survival

Low expression cohort (months)	High expression cohort (months)
19.77	19.73

**RNAseq ID:**	LY6G5B -
**Survival:**	OS
**Auto select best cutoff:**	checked
**Follow up threshold:**	all
**Censore at threshold:**	checked
**Compute median over entire database:**	false
**Cutoff value used in analysis:**	38
**Expression range of the probe:**	10 - 173
**Invert HR values below 1:**	not checked

Restrictions

Tumor type: Pancreatic ductal adenocarcinoma

Restrict analysis to subtypes...

Stage:	all
Gender:	all
Race:	all
Grade:	all
Mutation burden:	all

Restrict analysis based on cellular content...

Basophils:	all

B-cells:	all
CD4+ memory T-cells:	enriched
CD8+ T-cells:	all
Eosinophils:	all
Macrophages:	all
Mesenchymal stem cells:	all
Natural killer T-cells:	all
Regulatory T-cells:	all
Type 1 T-helper cells:	all
Type 2 T-helper cells:	all

Results

P value: 0.0062
FDR: over 50%

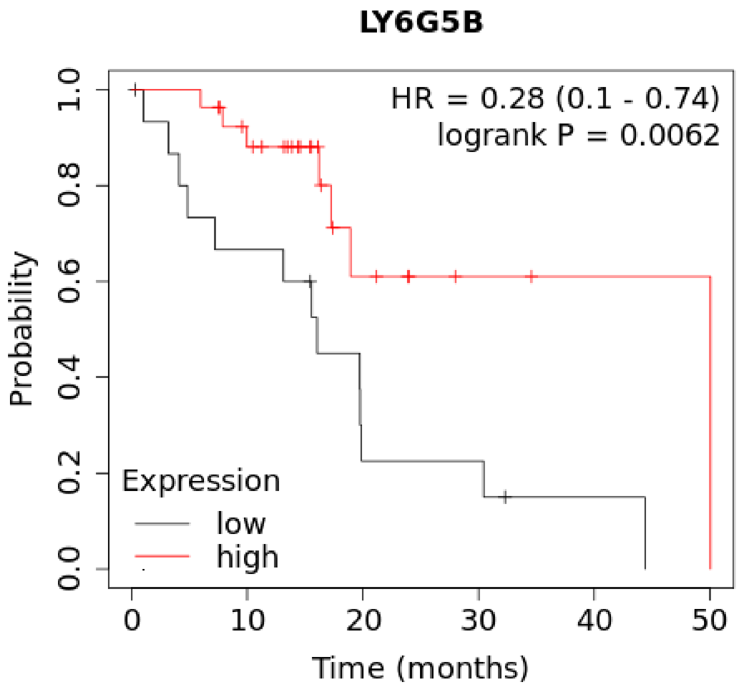

Number at risk

low	16	10	3	3	1	0
high	27	21	6	2	1	1

Click here to download the plot in TIFF format

Download plot as a PDF

Download p values vs. cutoff table

Median survival

Low expression cohort (months)	High expression cohort (months)
16.03	50.07

You can save the plots by right-clicking the image and then selecting "Save image as...". To generate a high resolution TIFF image, please adjust the "Settings" in the analysis page.

KMplot.com Copyright © 2009-2020 | Contact | Sitemap

Pan-cancer

KM plotter | Home | Vote | Download | Updates | Contact

The desired RNAseq ID is valid: PSCA (-), LY6K (-), SLURP1 (-), LYPD2 (-), LY6D (-), GML (-), LY6E (-), LY6L (-), LY6H (-), GPIHBP1 (-), LYPD4 (-), CD177 (-), TEX101 (-), LYPD3 (-), PINLYP (-), PLAUR (-), LYPD5 (-), SPACA4 (-), ACRV1 (-), PATE1 (-), PATE2 (-), PATE3 (-), PATE4 (-), CD59 (-), LY6G6C (-), LY6G6D (-), LY6G6F (-), LY6G5C (-), LY6G5B (-),

**RNAseq ID:**	PSCA -
**Survival:**	OS
**Auto select best cutoff:**	checked
**Follow up threshold:**	all
**Censore at threshold:**	checked
**Compute median over entire database:**	false
**Cutoff value used in analysis:**	222
**Expression range of the probe:**	0 - 65661
**Invert HR values below 1:**	not checked

Restrictions

Tumor type: Pancreatic ductal adenocarcinoma

Restrict analysis to subtypes...

Stage:	all
Gender:	all
Race:	all
Grade:	all
Mutation burden:	all

Restrict analysis based on cellular content...

Basophils:	all
B-cells:	all
CD4+ memory T-cells:	decreased
CD8+ T-cells:	all
Eosinophils:	all
Macrophages:	all
Mesenchymal stem cells:	all
Natural killer T-cells:	all
Regulatory T-cells:	all
Type 1 T-helper cells:	all
Type 2 T-helper cells:	all

Results

P value: 8.4e-5
FDR: 2%

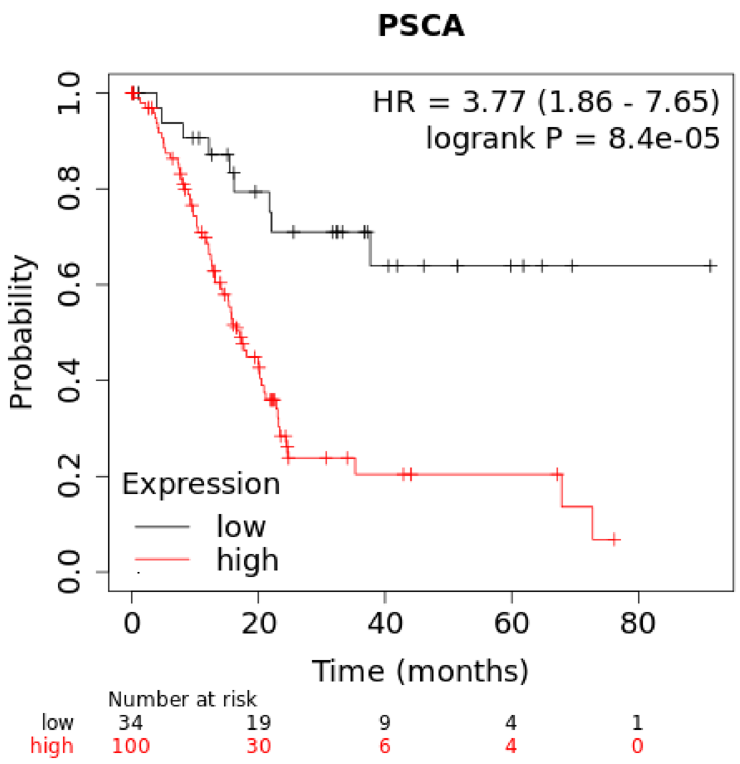

Click here to download the plot in TIFF format

Download plot as a PDF

Download p values vs. cutoff table

Upper quartile survival

Low expression cohort (months)	High expression cohort (months)
22.03	9.77

**RNAseq ID:**	LY6K -
**Survival:**	OS
**Auto select best cutoff:**	checked
**Follow up threshold:**	all
**Censore at threshold:**	checked
**Compute median over entire database:**	false
**Cutoff value used in analysis:**	6
**Expression range of the probe:**	0 - 1825
**Invert HR values below 1:**	not checked

Restrictions

Tumor type: Pancreatic ductal adenocarcinoma

Restrict analysis to subtypes...

Stage:	all
Gender:	all
Race:	all
Grade:	all
Mutation burden:	all

Restrict analysis based on cellular content...

Basophils:	all

B-cells:	all
CD4+ memory T-cells:	decreased
CD8+ T-cells:	all
Eosinophils:	all
Macrophages:	all
Mesenchymal stem cells:	all
Natural killer T-cells:	all
Regulatory T-cells:	all
Type 1 T-helper cells:	all
Type 2 T-helper cells:	all

Results

P value: 0.08
FDR: 100%

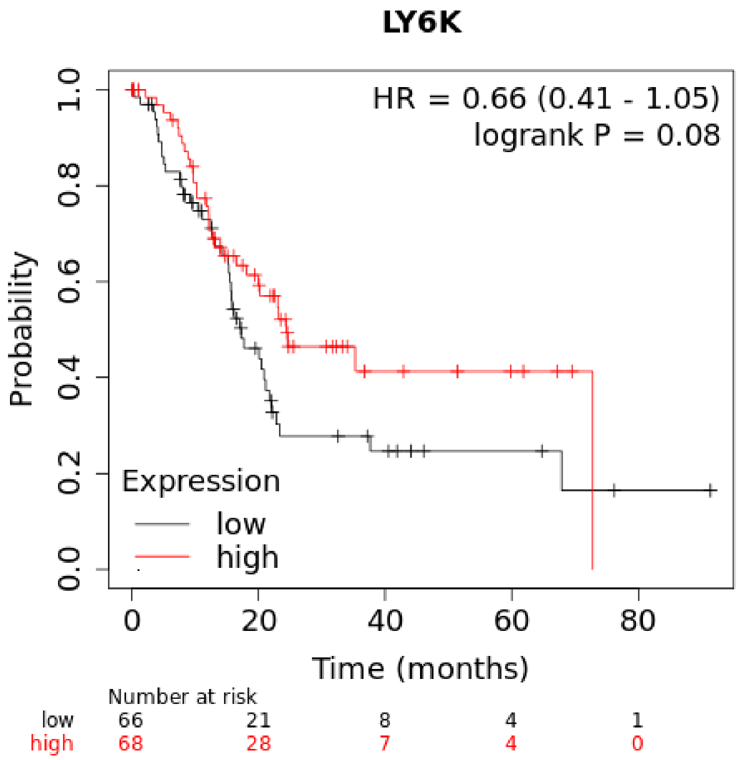

Click here to download the plot in TIFF format

Download plot as a PDF

Download p values vs. cutoff table

Median survival

Low expression cohort (months)	High expression cohort (months)
17.27	24.4

**RNAseq ID:**	SLURP1 -
**Survival:**	OS
**Auto select best cutoff:**	checked
**Follow up threshold:**	all
**Censore at threshold:**	checked
**Compute median over entire database:**	false
**Cutoff value used in analysis:**	1
**Expression range of the probe:**	0 - 279
**Invert HR values below 1:**	not checked

Restrictions

Tumor type: Pancreatic ductal adenocarcinoma

Restrict analysis to subtypes...

Stage:	all
Gender:	all
Race:	all
Grade:	all
Mutation burden:	all

Restrict analysis based on cellular content...

Basophils:	all
B-cells:	all
CD4+ memory T-cells:	decreased
CD8+ T-cells:	all
Eosinophils:	all
Macrophages:	all
Mesenchymal stem cells:	all
Natural killer T-cells:	all
Regulatory T-cells:	all
Type 1 T-helper cells:	all
Type 2 T-helper cells:	all

Results

P value: 1.3e-5
FDR: 1%

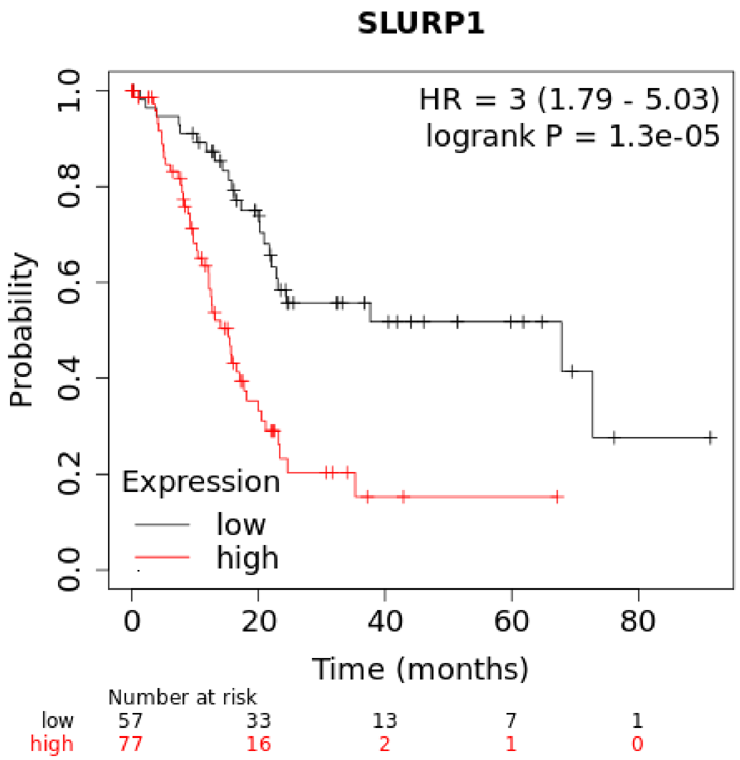

Click here to download the plot in TIFF format

Download plot as a PDF

Download p values vs. cutoff table

Median survival

Low expression cohort (months)	High expression cohort (months)
67.87	15.27

**RNAseq ID:**	LYPD2 -
**Survival:**	OS
**Auto select best cutoff:**	checked
**Follow up threshold:**	all
**Censore at threshold:**	checked
**Compute median over entire database:**	false
**Cutoff value used in analysis:**	4
**Expression range of the probe:**	0 - 4748
**Invert HR values below 1:**	not checked

Restrictions

Tumor type: Pancreatic ductal adenocarcinoma

Restrict analysis to subtypes...

Stage:	all
Gender:	all
Race:	all
Grade:	all
Mutation burden:	all

Restrict analysis based on cellular content...

Basophils:	all
B-cells:	all
CD4+ memory T-cells:	decreased
CD8+ T-cells:	all
Eosinophils:	all
Macrophages:	all
Mesenchymal stem cells:	all
Natural killer T-cells:	all
Regulatory T-cells:	all
Type 1 T-helper cells:	all
Type 2 T-helper cells:	all

Results

**P value:**	0.0041
**FDR:**	over 50%

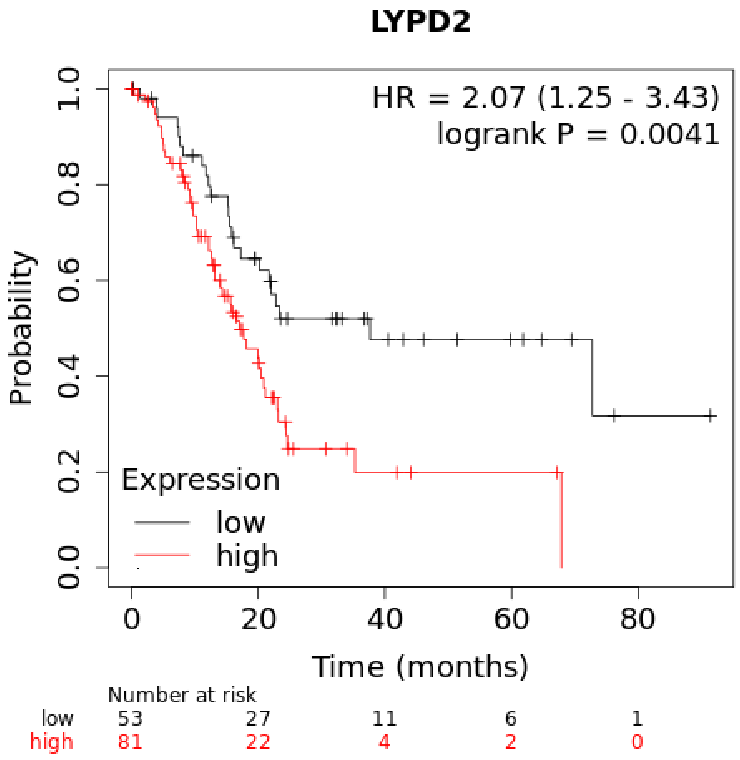

Click here to download the plot in TIFF format

Download plot as a PDF

Download p values vs. cutoff table

Median survival

Low expression cohort (months)	High expression cohort (months)
37.67	17.03

**RNAseq ID:**	LY6D -
**Survival:**	OS
**Auto select best cutoff:**	checked
**Follow up threshold:**	all
**Censore at threshold:**	checked
**Compute median over entire database:**	false
**Cutoff value used in analysis:**	642
**Expression range of the probe:**	0 - 18030
**Invert HR values below 1:**	not checked

Restrictions

Tumor type: Pancreatic ductal adenocarcinoma

Restrict analysis to subtypes...

Stage:	all
Gender:	all
Race:	all
Grade:	all
Mutation burden:	all

Restrict analysis based on cellular content...

Basophils:	all

B-cells:	all
CD4+ memory T-cells:	decreased
CD8+ T-cells:	all
Eosinophils:	all
Macrophages:	all
Mesenchymal stem cells:	all
Natural killer T-cells:	all
Regulatory T-cells:	all
Type 1 T-helper cells:	all
Type 2 T-helper cells:	all

Results

P value: 0.0001
FDR: 5%

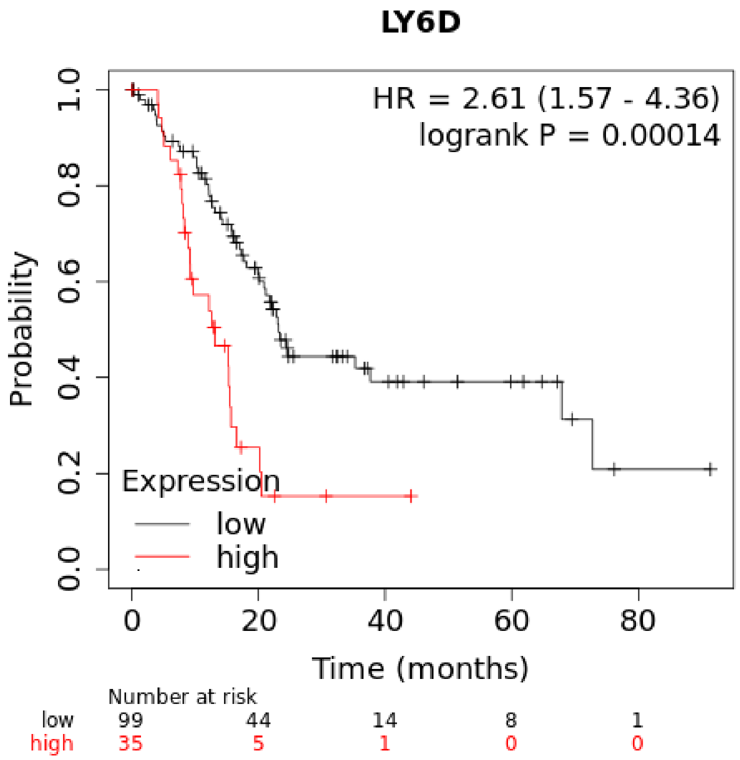

Click here to download the plot in TIFF format

Download plot as a PDF

Download p values vs. cutoff table

Median survival

Low expression cohort (months)	High expression cohort (months)
23.17	13.1

**RNAseq ID:**	GML -
**Survival:**	OS
**Auto select best cutoff:**	checked
**Follow up threshold:**	all
**Censore at threshold:**	checked
**Compute median over entire database:**	false
**Cutoff value used in analysis:**	0
**Expression range of the probe:**	0 - 2
**Invert HR values below 1:**	not checked

Restrictions

Tumor type: Pancreatic ductal adenocarcinoma

Restrict analysis to subtypes...

Stage: all
Gender: all
Race: all
Grade: all
Mutation burden: all

Restrict analysis based on cellular content...

Basophils: all
B-cells: all
CD4+ memory T-cells: decreased
CD8+ T-cells: all
Eosinophils: all
Macrophages: all
Mesenchymal stem cells: all
Natural killer T-cells: all
Regulatory T-cells: all
Type 1 T-helper cells: all
Type 2 T-helper cells: all

Results

P value: 0.0112
FDR: over 50%

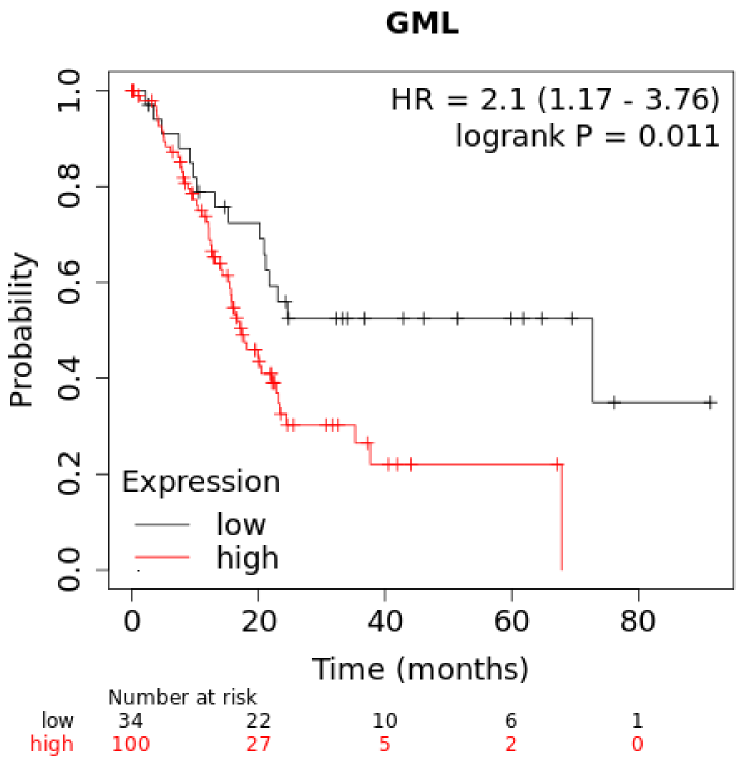

Click here to download the plot in TIFF format

Download plot as a PDF

Download p values vs. cutoff table

Median survival

Low expression cohort (months)	High expression cohort (months)
72.73	17.27

**RNAseq ID:**	LY6E -
**Survival:**	OS
**Auto select best cutoff:**	checked
**Follow up threshold:**	all
**Censore at threshold:**	checked
**Compute median over entire database:**	false
**Cutoff value used in analysis:**	10191
**Expression range of the probe:**	254 - 56404
**Invert HR values below 1:**	not checked

Restrictions

Tumor type: Pancreatic ductal adenocarcinoma

Restrict analysis to subtypes...

Stage:	all
Gender:	all
Race:	all
Grade:	all
Mutation burden:	all

Restrict analysis based on cellular content...

Basophils:	all
B-cells:	all
CD4+ memory T-cells:	decreased
CD8+ T-cells:	all
Eosinophils:	all
Macrophages:	all
Mesenchymal stem cells:	all
Natural killer T-cells:	all
Regulatory T-cells:	all
Type 1 T-helper cells:	all
Type 2 T-helper cells:	all

Results

**P value:**	0.0248
**FDR:**	over 50%

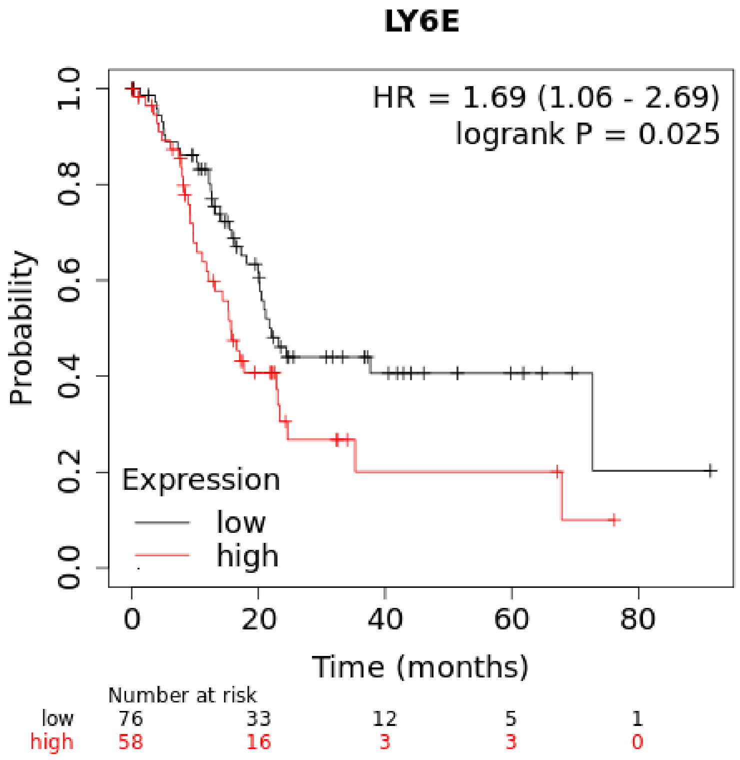

Click here to download the plot in TIFF format

Download plot as a PDF

Download p values vs. cutoff table

Median survival

Low expression cohort (months)	High expression cohort (months)
22.03	15.67

RNAseq ID: LY6L -
Survival: OS
Auto select best cutoff: checked
Follow up threshold: all
Censore at threshold: checked
Compute median over entire database: false
Cutoff value used in analysis: 0
Expression range of the probe: 0 - 6
Invert HR values below 1: not checked

Restrictions

Tumor type: Pancreatic ductal adenocarcinoma

Restrict analysis to subtypes...

Stage: all
Gender: all
Race: all
Grade: all
Mutation burden: all

Restrict analysis based on cellular content...

Basophils: all

B-cells: all
CD4+ memory T-cells: decreased
CD8+ T-cells: all
Eosinophils: all
Macrophages: all
Mesenchymal stem cells: all
Natural killer T-cells: all
Regulatory T-cells: all
Type 1 T-helper cells: all
Type 2 T-helper cells: all

Results

P value: 0.0056
FDR: over 50%

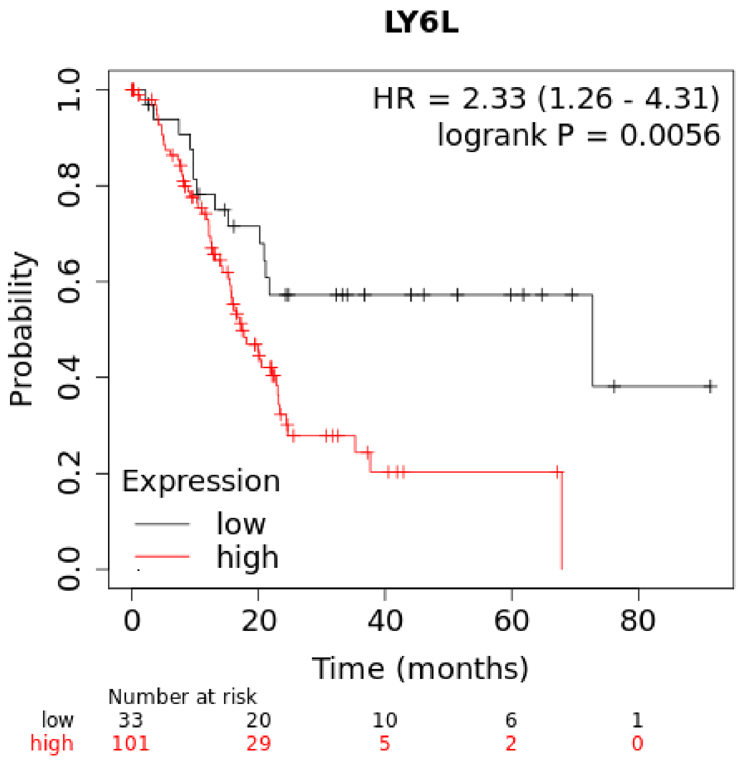

Click here to download the plot in TIFF format

Download plot as a PDF

Download p values vs. cutoff table

Median survival

Low expression cohort (months)	High expression cohort (months)
72.73	17.27

RNAseq ID: LY6H -
Survival: OS
Auto select best cutoff: checked
Follow up threshold: all
Censore at threshold: checked
Compute median over entire database: false
Cutoff value used in analysis: 39
Expression range of the probe: 1 - 9495
Invert HR values below 1: not checked

Restrictions

Tumor type: Pancreatic ductal adenocarcinoma

Restrict analysis to subtypes...

Stage:	all
Gender:	all
Race:	all
Grade:	all
Mutation burden:	all

Restrict analysis based on cellular content...

Basophils:	all
B-cells:	all
CD4+ memory T-cells:	decreased
CD8+ T-cells:	all
Eosinophils:	all
Macrophages:	all
Mesenchymal stem cells:	all
Natural killer T-cells:	all
Regulatory T-cells:	all
Type 1 T-helper cells:	all
Type 2 T-helper cells:	all

Results

P value: 0.006
FDR: 50%

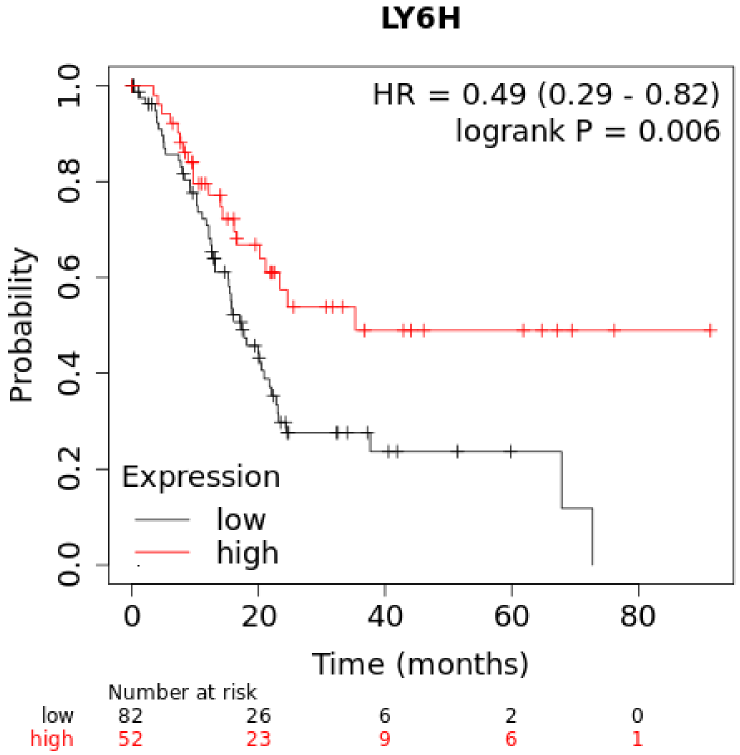

Click here to download the plot in TIFF format

Download plot as a PDF

Download p values vs. cutoff table

Median survival

Low expression cohort (months)	High expression cohort (months)
17.27	35.3

**RNAseq ID:**	GPIHBP1 -
**Survival:**	OS
**Auto select best cutoff:**	checked
**Follow up threshold:**	all
**Censore at threshold:**	checked
**Compute median over entire database:**	false
**Cutoff value used in analysis:**	23
**Expression range of the probe:**	5 - 344
**Invert HR values below 1:**	not checked

Restrictions

Tumor type: Pancreatic ductal adenocarcinoma

Restrict analysis to subtypes...

Stage:	all
Gender:	all
Race:	all
Grade:	all
Mutation burden:	all

Restrict analysis based on cellular content...

Basophils:	all
B-cells:	all
CD4+ memory T-cells:	decreased
CD8+ T-cells:	all
Eosinophils:	all
Macrophages:	all
Mesenchymal stem cells:	all
Natural killer T-cells:	all
Regulatory T-cells:	all
Type 1 T-helper cells:	all
Type 2 T-helper cells:	all

Results

**P value:**	0.1511
**FDR:**	100%

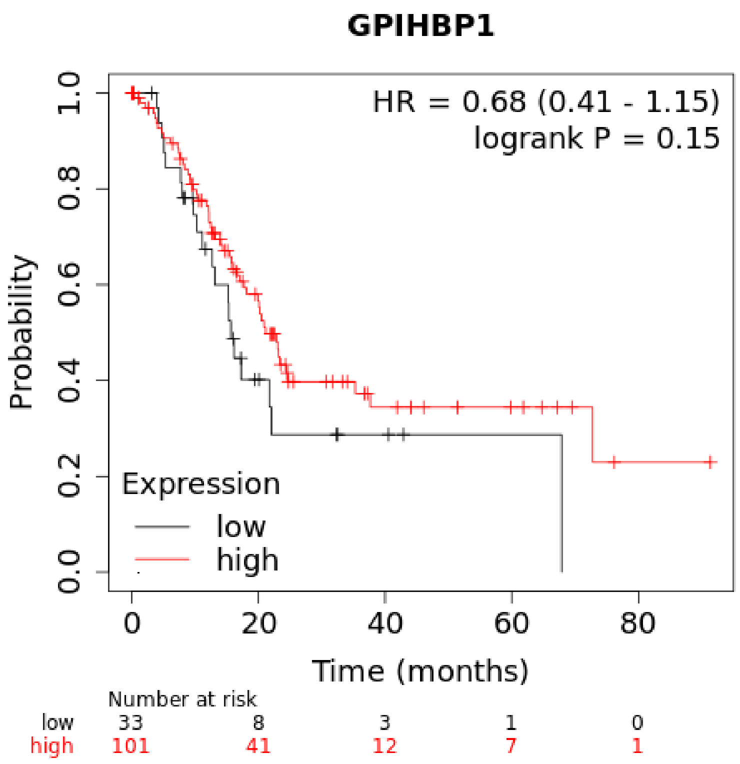

Click here to download the plot in TIFF format

Download plot as a PDF

Download p values vs. cutoff table

Median survival

Low expression cohort (months)	High expression cohort (months)
15.67	21.13

**RNAseq ID:**	LYPD4 -
**Survival:**	OS
**Auto select best cutoff:**	checked
**Follow up threshold:**	all
**Censore at threshold:**	checked
**Compute median over entire database:**	false
**Cutoff value used in analysis:**	0
**Expression range of the probe:**	0 - 18
**Invert HR values below 1:**	not checked

Restrictions

Tumor type: Pancreatic ductal adenocarcinoma

Restrict analysis to subtypes...

Stage:	all
Gender:	all
Race:	all
Grade:	all
Mutation burden:	all

Restrict analysis based on cellular content...

Basophils:	all

B-cells:	all
CD4+ memory T-cells:	decreased
CD8+ T-cells:	all
Eosinophils:	all
Macrophages:	all
Mesenchymal stem cells:	all
Natural killer T-cells:	all
Regulatory T-cells:	all
Type 1 T-helper cells:	all
Type 2 T-helper cells:	all

Results

P value: 0.003
FDR: over 50%

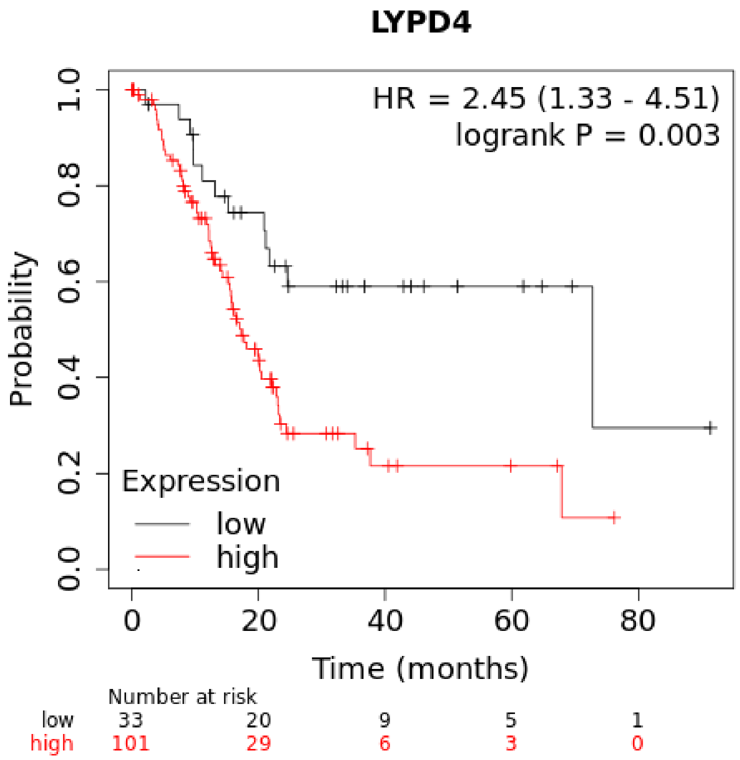

Click here to download the plot in TIFF format

Download plot as a PDF

Download p values vs. cutoff table

Median survival

Low expression cohort (months)	High expression cohort (months)
72.73	17.27

**RNAseq ID:**	CD177 -
**Survival:**	OS
**Auto select best cutoff:**	checked
**Follow up threshold:**	all
**Censore at threshold:**	checked
**Compute median over entire database:**	false
**Cutoff value used in analysis:**	20
**Expression range of the probe:**	0 - 6100
**Invert HR values below 1:**	not checked

Restrictions

Tumor type: Pancreatic ductal adenocarcinoma

Restrict analysis to subtypes...

Stage:	all
Gender:	all
Race:	all
Grade:	all
Mutation burden:	all

Restrict analysis based on cellular content...

Basophils:	all
B-cells:	all
CD4+ memory T-cells:	decreased
CD8+ T-cells:	all
Eosinophils:	all
Macrophages:	all
Mesenchymal stem cells:	all
Natural killer T-cells:	all
Regulatory T-cells:	all
Type 1 T-helper cells:	all
Type 2 T-helper cells:	all

Results

P value: 0.0952
FDR: 100%

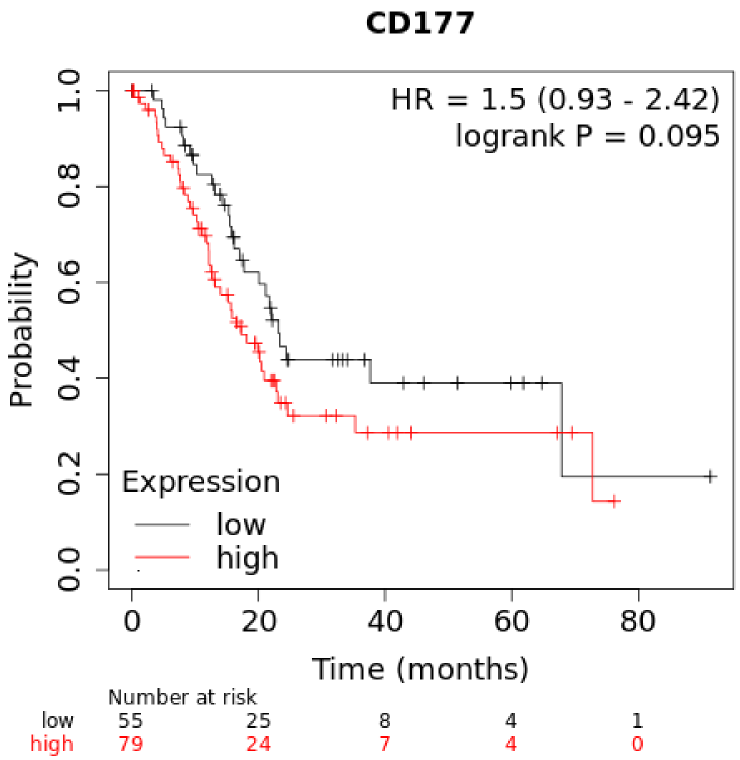

Click here to download the plot in TIFF format

Download plot as a PDF

Download p values vs. cutoff table

Median survival

Low expression cohort (months)	High expression cohort (months)
23.17	17.27

**RNAseq ID:**	TEX101 -
**Survival:**	OS
**Auto select best cutoff:**	checked
**Follow up threshold:**	all
**Censore at threshold:**	checked
**Compute median over entire database:**	false
**Cutoff value used in analysis:**	0
**Expression range of the probe:**	0 - 149
**Invert HR values below 1:**	not checked

Restrictions

Tumor type: Pancreatic ductal adenocarcinoma

Restrict analysis to subtypes...

Stage:	all
Gender:	all
Race:	all
Grade:	all
Mutation burden:	all

Restrict analysis based on cellular content...

Basophils:	all
B-cells:	all
CD4+ memory T-cells:	decreased
CD8+ T-cells:	all
Eosinophils:	all
Macrophages:	all
Mesenchymal stem cells:	all
Natural killer T-cells:	all
Regulatory T-cells:	all
Type 1 T-helper cells:	all
Type 2 T-helper cells:	all

Results

**P value:**	0.2005
**FDR:**	100%

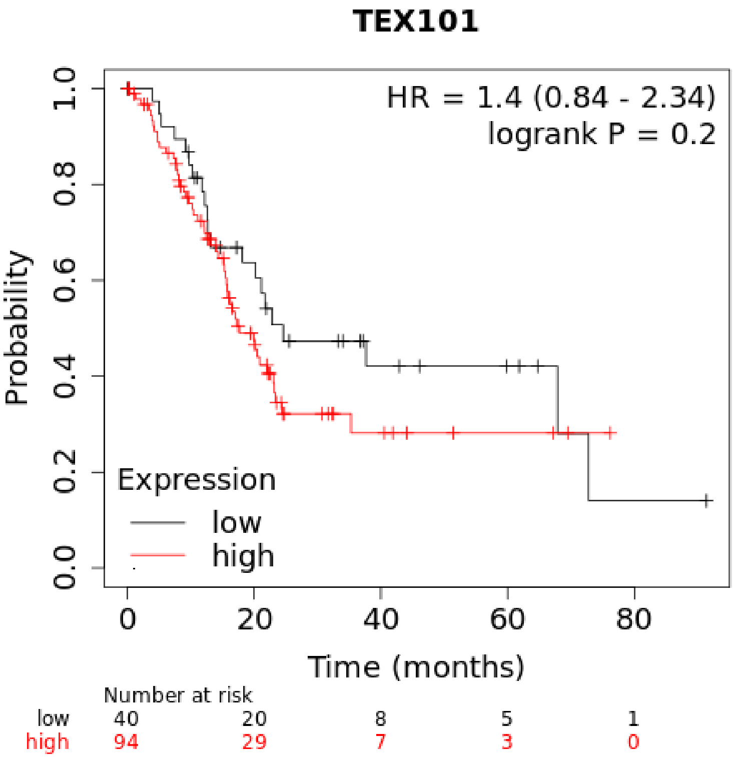

Click here to download the plot in TIFF format

Download plot as a PDF

Download p values vs. cutoff table

Median survival

Low expression cohort (months)	High expression cohort (months)
24.6	17.73

RNAseq ID: LYPD3 -
Survival: OS
Auto select best cutoff: checked
Follow up threshold: all
Censore at threshold: checked
Compute median over entire database: false
Cutoff value used in analysis: 144
Expression range of the probe: 9 - 7684
Invert HR values below 1: not checked

Restrictions

Tumor type: Pancreatic ductal adenocarcinoma

Restrict analysis to subtypes...

Stage: all
Gender: all
Race: all
Grade: all
Mutation burden: all

Restrict analysis based on cellular content...

Basophils: all

B-cells:	all
CD4+ memory T-cells:	decreased
CD8+ T-cells:	all
Eosinophils:	all
Macrophages:	all
Mesenchymal stem cells:	all
Natural killer T-cells:	all
Regulatory T-cells:	all
Type 1 T-helper cells:	all
Type 2 T-helper cells:	all

Results

P value: 0.1121
FDR: 100%

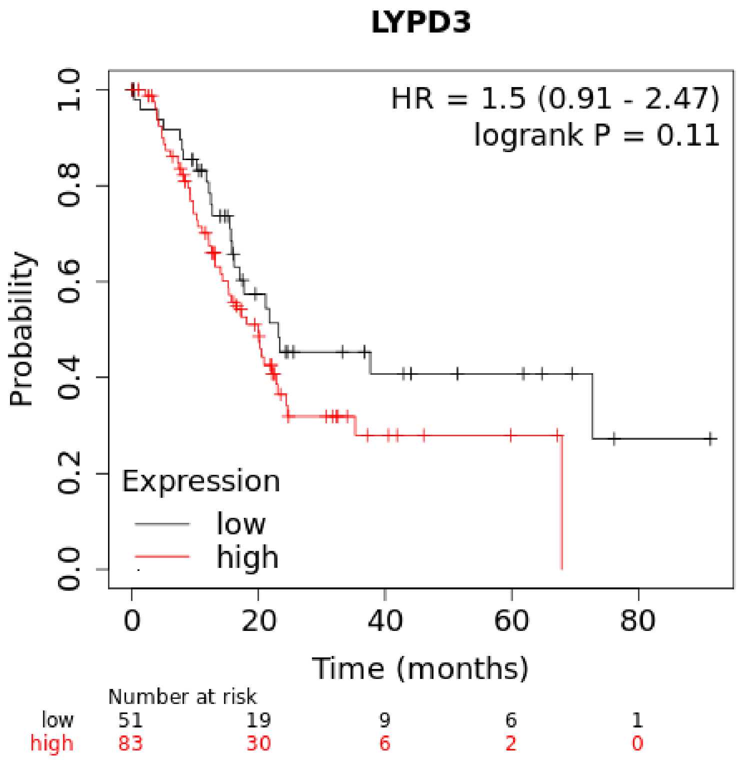

Click here to download the plot in TIFF format

Download plot as a PDF

Download p values vs. cutoff table

Median survival

Low expression cohort (months)	High expression cohort (months)
23.17	19.93

**RNAseq ID:**	PINLYP -
**Survival:**	OS
**Auto select best cutoff:**	checked
**Follow up threshold:**	all
**Censore at threshold:**	checked
**Compute median over entire database:**	false
**Cutoff value used in analysis:**	71
**Expression range of the probe:**	5 - 263
**Invert HR values below 1:**	not checked

Restrictions

Tumor type: Pancreatic ductal adenocarcinoma

Restrict analysis to subtypes...

Stage:	all
Gender:	all
Race:	all
Grade:	all
Mutation burden:	all

Restrict analysis based on cellular content...

Basophils:	all
B-cells:	all
CD4+ memory T-cells:	decreased
CD8+ T-cells:	all
Eosinophils:	all
Macrophages:	all
Mesenchymal stem cells:	all
Natural killer T-cells:	all
Regulatory T-cells:	all
Type 1 T-helper cells:	all
Type 2 T-helper cells:	all

Results

P value: 0.0458
FDR: over 50%

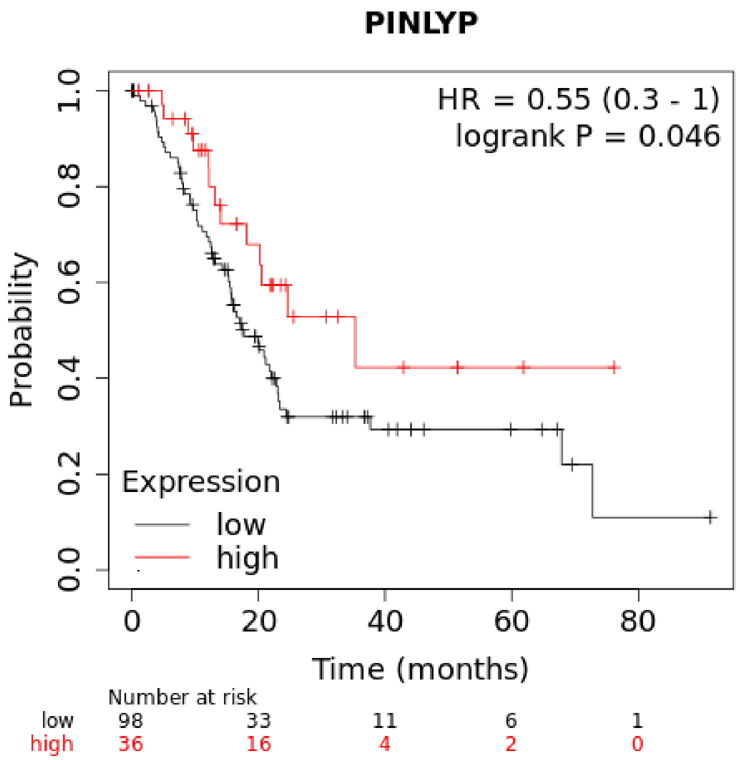

Click here to download the plot in TIFF format

Download plot as a PDF

Download p values vs. cutoff table

Median survival

Low expression cohort (months)	High expression cohort (months)
17.73	35.3

**RNAseq ID:**	PLAUR -
**Survival:**	OS
**Auto select best cutoff:**	checked
**Follow up threshold:**	all
**Censore at threshold:**	checked
**Compute median over entire database:**	false
**Cutoff value used in analysis:**	1937
**Expression range of the probe:**	47 - 18314
**Invert HR values below 1:**	not checked

Restrictions

Tumor type: Pancreatic ductal adenocarcinoma

Restrict analysis to subtypes...

Stage:	all
Gender:	all
Race:	all
Grade:	all
Mutation burden:	all

Restrict analysis based on cellular content...

Basophils:	all
B-cells:	all
CD4+ memory T-cells:	decreased
CD8+ T-cells:	all
Eosinophils:	all
Macrophages:	all
Mesenchymal stem cells:	all
Natural killer T-cells:	all
Regulatory T-cells:	all
Type 1 T-helper cells:	all
Type 2 T-helper cells:	all

Results

**P value:**	0.0976
**FDR:**	100%

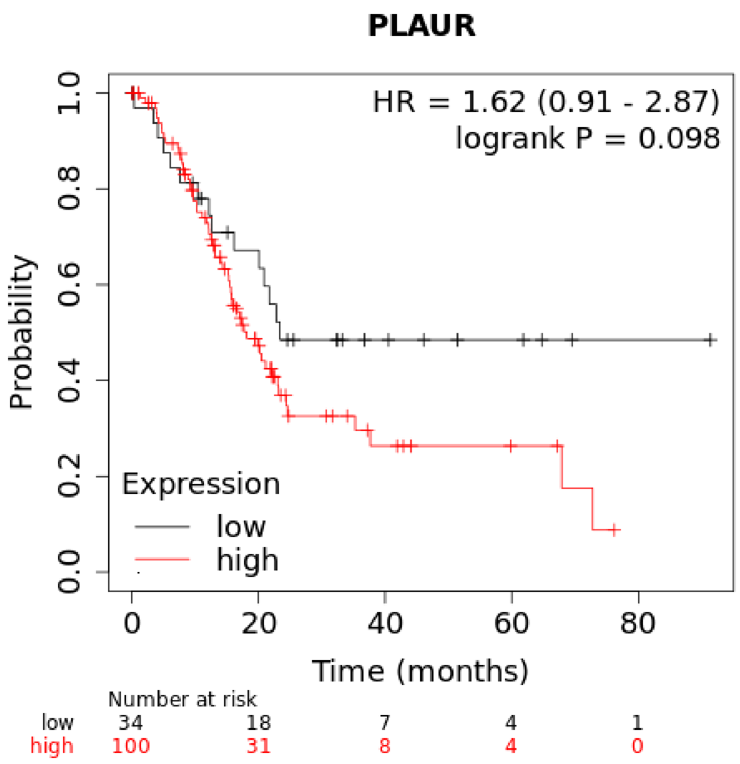

Click here to download the plot in TIFF format

Download plot as a PDF

Download p values vs. cutoff table

Median survival

Low expression cohort (months)	High expression cohort (months)
23.4	18.17

**RNAseq ID:**	LYPD5 -
**Survival:**	OS
**Auto select best cutoff:**	checked
**Follow up threshold:**	all
**Censore at threshold:**	checked
**Compute median over entire database:**	false
**Cutoff value used in analysis:**	70
**Expression range of the probe:**	1 - 419
**Invert HR values below 1:**	not checked

Restrictions

Tumor type: Pancreatic ductal adenocarcinoma

Restrict analysis to subtypes...

Stage:	all
Gender:	all
Race:	all
Grade:	all
Mutation burden:	all

Restrict analysis based on cellular content...

Basophils:	all

B-cells:	all
CD4+ memory T-cells:	decreased
CD8+ T-cells:	all
Eosinophils:	all
Macrophages:	all
Mesenchymal stem cells:	all
Natural killer T-cells:	all
Regulatory T-cells:	all
Type 1 T-helper cells:	all
Type 2 T-helper cells:	all

Results

P value: 0.0044
FDR: 50%

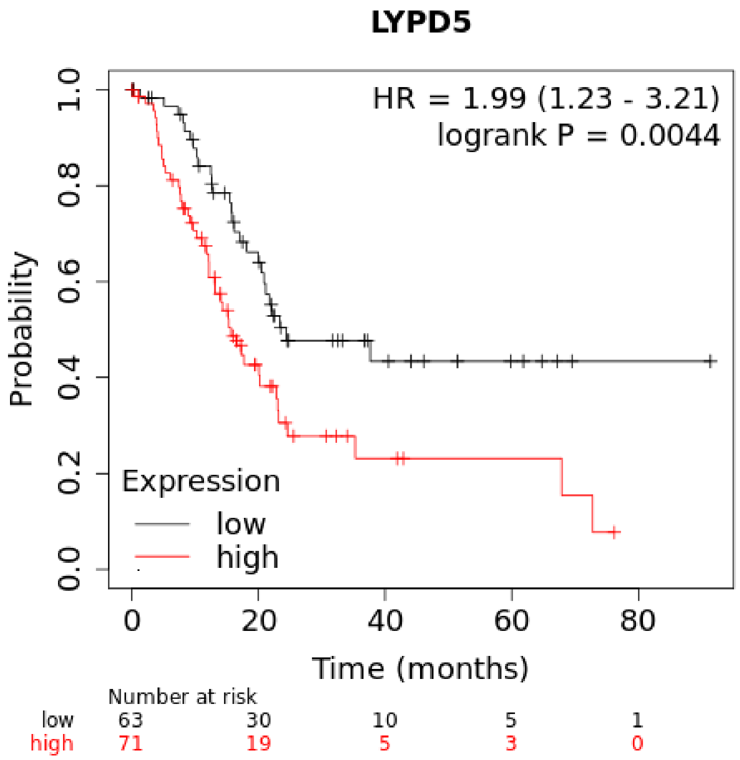

Click here to download the plot in TIFF format

Download plot as a PDF

Download p values vs. cutoff table

Median survival

Low expression cohort (months)	High expression cohort (months)
24.4	15.67

**RNAseq ID:**	SPACA4 -
**Survival:**	OS
**Auto select best cutoff:**	checked
**Follow up threshold:**	all
**Censore at threshold:**	checked
**Compute median over entire database:**	false
**Cutoff value used in analysis:**	19
**Expression range of the probe:**	0 - 206
**Invert HR values below 1:**	not checked

Restrictions

Tumor type: Pancreatic ductal adenocarcinoma

Restrict analysis to subtypes...

Stage:	all
Gender:	all
Race:	all
Grade:	all
Mutation burden:	all

Restrict analysis based on cellular content...

Basophils:	all
B-cells:	all
CD4+ memory T-cells:	decreased
CD8+ T-cells:	all
Eosinophils:	all
Macrophages:	all
Mesenchymal stem cells:	all
Natural killer T-cells:	all
Regulatory T-cells:	all
Type 1 T-helper cells:	all
Type 2 T-helper cells:	all

Results

P value: 0.3037
FDR: 100%

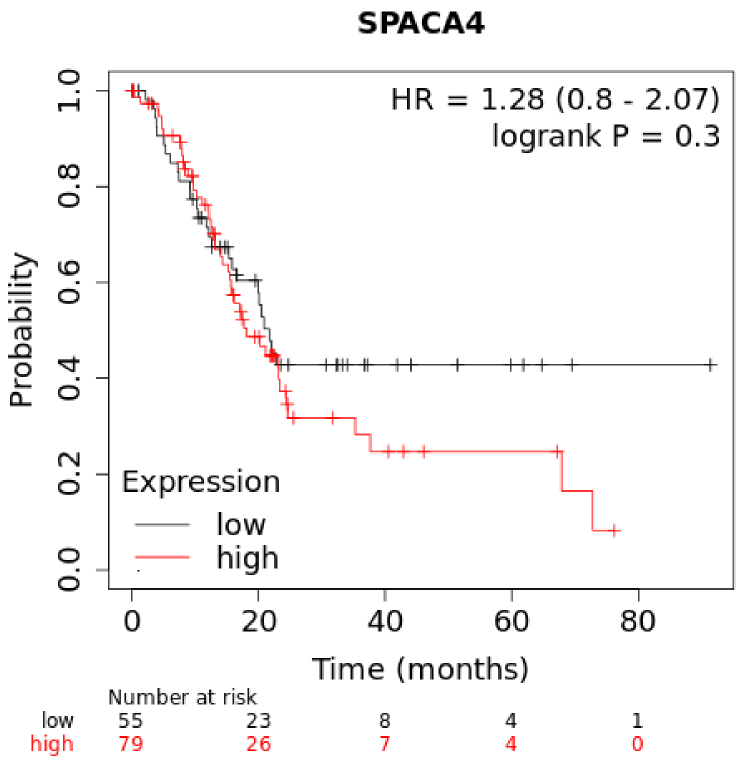

Click here to download the plot in TIFF format

Download plot as a PDF

Download p values vs. cutoff table

Median survival

Low expression cohort (months)	High expression cohort (months)
21.73	18.17

**RNAseq ID:**	ACRV1 -
**Survival:**	OS
**Auto select best cutoff:**	checked
**Follow up threshold:**	all
**Censore at threshold:**	checked
**Compute median over entire database:**	false
**Cutoff value used in analysis:**	6
**Expression range of the probe:**	0 - 71
**Invert HR values below 1:**	not checked

Restrictions

Tumor type: Pancreatic ductal adenocarcinoma

Restrict analysis to subtypes...

Stage:	all
Gender:	all
Race:	all
Grade:	all
Mutation burden:	all

Restrict analysis based on cellular content...

Basophils:	all
B-cells:	all
CD4+ memory T-cells:	decreased
CD8+ T-cells:	all
Eosinophils:	all
Macrophages:	all
Mesenchymal stem cells:	all
Natural killer T-cells:	all
Regulatory T-cells:	all
Type 1 T-helper cells:	all
Type 2 T-helper cells:	all

Results

**P value:**	0.0635
**FDR:**	100%

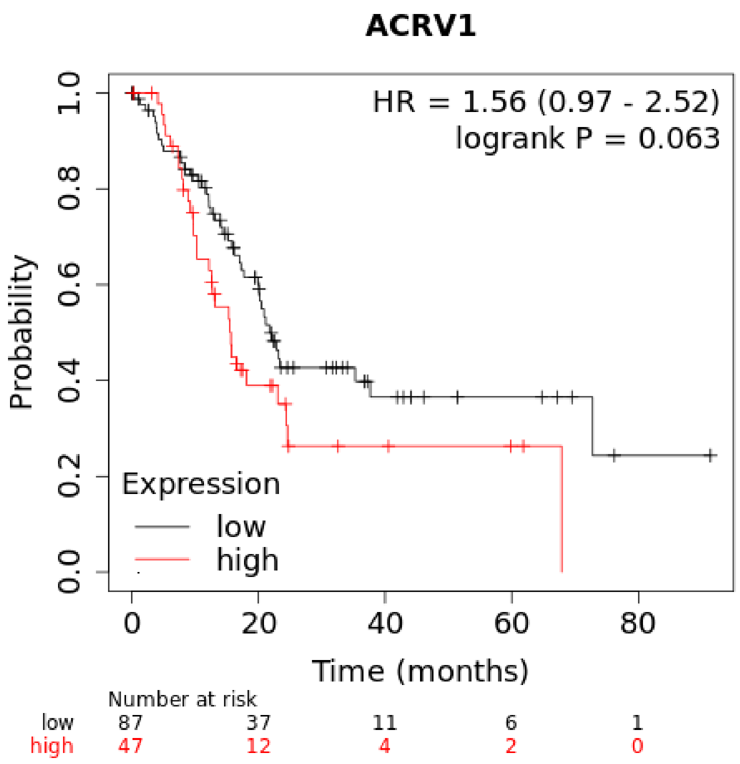

Click here to download the plot in TIFF format

Download plot as a PDF

Download p values vs. cutoff table

Median survival

Low expression cohort (months)	High expression cohort (months)
21.73	15.67

**RNAseq ID:**	PATE1 -
**Survival:**	OS
**Auto select best cutoff:**	checked
**Follow up threshold:**	all
**Censore at threshold:**	checked
**Compute median over entire database:**	false
**Cutoff value used in analysis:**	0
**Expression range of the probe:**	0 - 1
**Invert HR values below 1:**	not checked

Restrictions

Tumor type: Pancreatic ductal adenocarcinoma

Restrict analysis to subtypes...

Stage: all
Gender: all
Race: all
Grade: all
Mutation burden: all

Restrict analysis based on cellular content...

Basophils: all

B-cells:	all
CD4+ memory T-cells:	decreased
CD8+ T-cells:	all
Eosinophils:	all
Macrophages:	all
Mesenchymal stem cells:	all
Natural killer T-cells:	all
Regulatory T-cells:	all
Type 1 T-helper cells:	all
Type 2 T-helper cells:	all

Results

P value: 0.0007
FDR: 10%

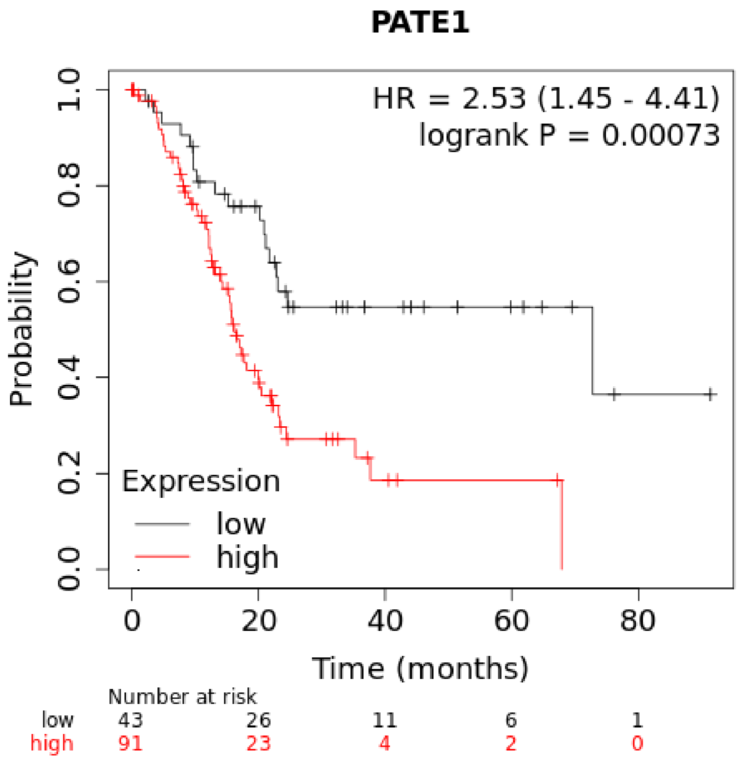

Click here to download the plot in TIFF format

Download plot as a PDF

Download p values vs. cutoff table

Median survival

Low expression cohort (months)	High expression cohort (months)
72.73	16.17

**RNAseq ID:**	PATE2 -
**Survival:**	OS
**Auto select best cutoff:**	checked
**Follow up threshold:**	all
**Censore at threshold:**	checked
**Compute median over entire database:**	false
**Cutoff value used in analysis:**	1
**Expression range of the probe:**	0 - 7
**Invert HR values below 1:**	not checked

Restrictions

Tumor type: Pancreatic ductal adenocarcinoma

Restrict analysis to subtypes...

Stage: all
Gender: all
Race: all
Grade: all
Mutation burden: all

Restrict analysis based on cellular content...

Basophils: all
B-cells: all
CD4+ memory T-cells: decreased
CD8+ T-cells: all
Eosinophils: all
Macrophages: all
Mesenchymal stem cells: all
Natural killer T-cells: all
Regulatory T-cells: all
Type 1 T-helper cells: all
Type 2 T-helper cells: all

Results

P value: 0.0004
FDR: 20%

PATE2

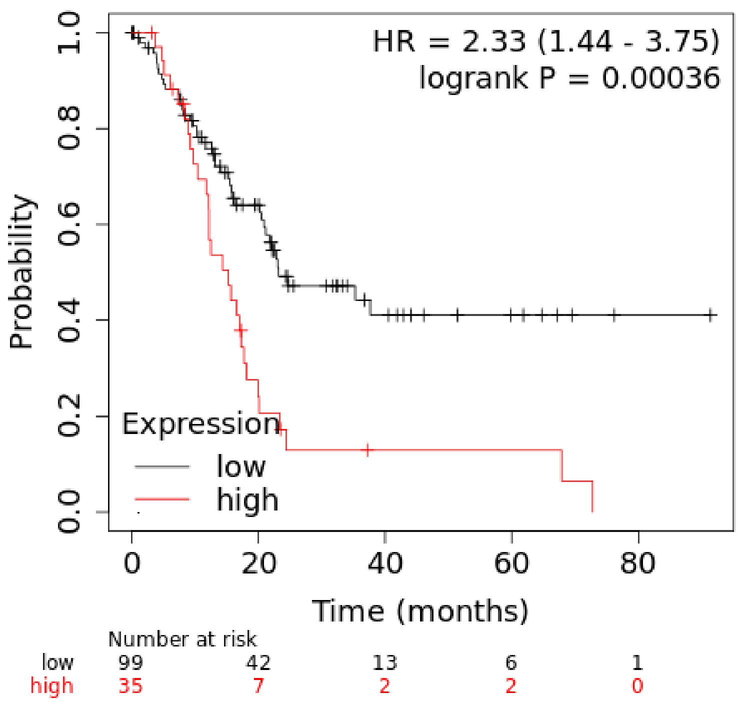

Click here to download the plot in TIFF format

Download plot as a PDF

Download p values vs. cutoff table

Median survival

Low expression cohort (months)	High expression cohort (months)
23.17	15.27

**RNAseq ID:**	PATE3 -
**Survival:**	OS
**Auto select best cutoff:**	checked
**Follow up threshold:**	all
**Censore at threshold:**	checked
**Compute median over entire database:**	false
**Cutoff value used in analysis:**	0
**Expression range of the probe:**	0 - 1
**Invert HR values below 1:**	not checked

Restrictions

Tumor type: Pancreatic ductal adenocarcinoma

Restrict analysis to subtypes...

Stage:	all
Gender:	all
Race:	all
Grade:	all
Mutation burden:	all

Restrict analysis based on cellular content...

Basophils:	all
B-cells:	all
CD4+ memory T-cells:	decreased
CD8+ T-cells:	all
Eosinophils:	all
Macrophages:	all
Mesenchymal stem cells:	all
Natural killer T-cells:	all
Regulatory T-cells:	all
Type 1 T-helper cells:	all
Type 2 T-helper cells:	all

Results

**P value:**	0.0052
**FDR:**	over 50%

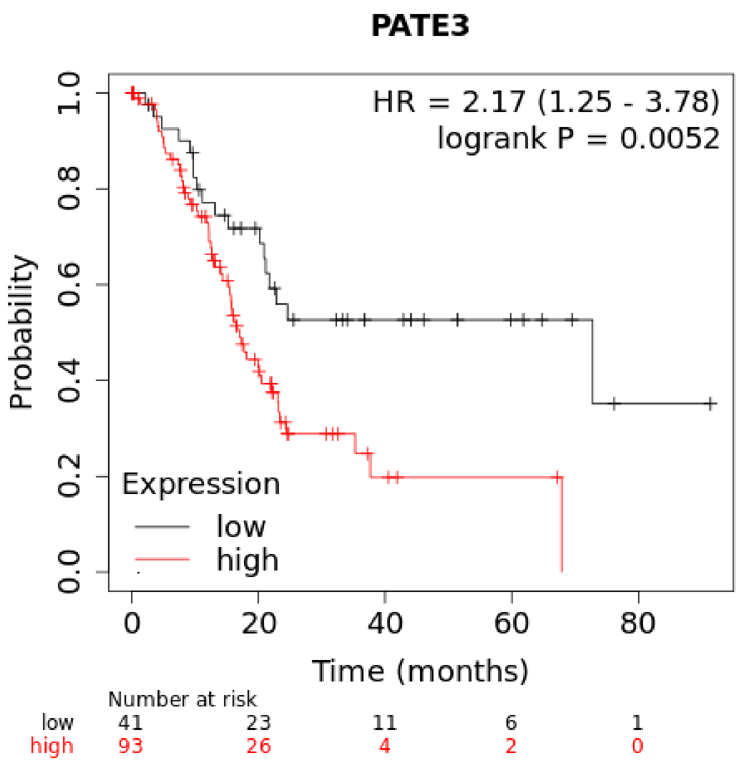

Click here to download the plot in TIFF format

Download plot as a PDF

Download p values vs. cutoff table

Median survival

Low expression cohort (months)	High expression cohort (months)
72.73	17.03

**RNAseq ID:**	PATE4 -
**Survival:**	OS
**Auto select best cutoff:**	checked
**Follow up threshold:**	all
**Censore at threshold:**	checked
**Compute median over entire database:**	false
**Cutoff value used in analysis:**	0
**Expression range of the probe:**	0 - 3
**Invert HR values below 1:**	not checked

Restrictions

Tumor type: Pancreatic ductal adenocarcinoma

Restrict analysis to subtypes...

Stage:	all
Gender:	all
Race:	all
Grade:	all
Mutation burden:	all

Restrict analysis based on cellular content...

Basophils:	all

B-cells:	all
CD4+ memory T-cells:	decreased
CD8+ T-cells:	all
Eosinophils:	all
Macrophages:	all
Mesenchymal stem cells:	all
Natural killer T-cells:	all
Regulatory T-cells:	all
Type 1 T-helper cells:	all
Type 2 T-helper cells:	all

Results

P value: 0.1777
FDR: 100%

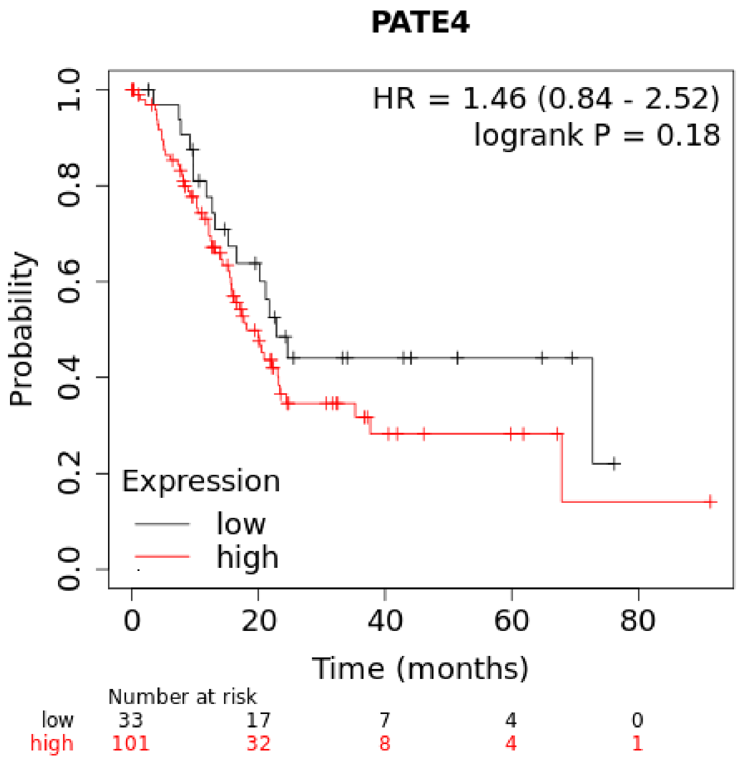

[Click here to download the plot in TIFF format]()

[Download plot as a PDF]()

[Download p values vs. cutoff table]()

Median survival

Low expression cohort (months)	High expression cohort (months)
22.8	18.17

**RNAseq ID:**	CD59	-
**Survival:**	OS	
**Auto select best cutoff:**	checked	
**Follow up threshold:**	all	
**Censore at threshold:**	checked	
**Compute median over entire database:**	false	
**Cutoff value used in analysis:**	17322	
**Expression range of the probe:**	2610 - 39336	
**Invert HR values below 1:**	not checked	

Restrictions

Tumor type: Pancreatic ductal adenocarcinoma

Restrict analysis to subtypes...

Stage:	all
Gender:	all
Race:	all
Grade:	all
Mutation burden:	all

Restrict analysis based on cellular content...

Basophils:	all
B-cells:	all
CD4+ memory T-cells:	decreased
CD8+ T-cells:	all
Eosinophils:	all
Macrophages:	all
Mesenchymal stem cells:	all
Natural killer T-cells:	all
Regulatory T-cells:	all
Type 1 T-helper cells:	all
Type 2 T-helper cells:	all

Results

P value: 0.0006
FDR: 10%

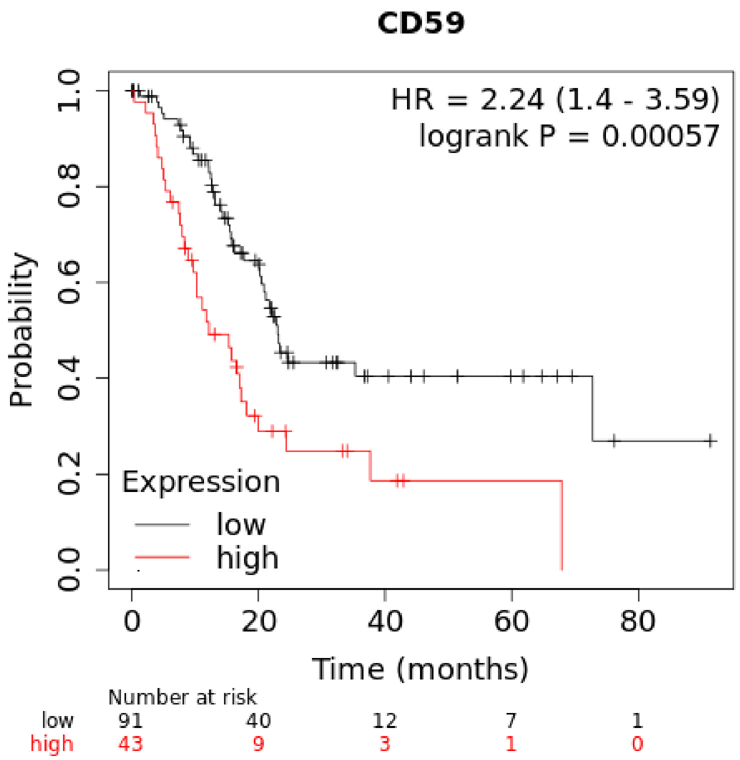

Click here to download the plot in TIFF format

Download plot as a PDF

Download p values vs. cutoff table

Median survival

Low expression cohort (months)	High expression cohort (months)
23.03	12.2

**RNAseq ID:**	LY6G6C -
**Survival:**	OS
**Auto select best cutoff:**	checked
**Follow up threshold:**	all
**Censore at threshold:**	checked
**Compute median over entire database:**	false
**Cutoff value used in analysis:**	13
**Expression range of the probe:**	0 - 251
**Invert HR values below 1:**	not checked

Restrictions

Tumor type: Pancreatic ductal adenocarcinoma

Restrict analysis to subtypes...

Stage:	all
Gender:	all
Race:	all
Grade:	all
Mutation burden:	all

Restrict analysis based on cellular content...

Basophils:	all
B-cells:	all
CD4+ memory T-cells:	decreased
CD8+ T-cells:	all
Eosinophils:	all
Macrophages:	all
Mesenchymal stem cells:	all
Natural killer T-cells:	all
Regulatory T-cells:	all
Type 1 T-helper cells:	all
Type 2 T-helper cells:	all

Results

**P value:**	0.0037
**FDR:**	50%

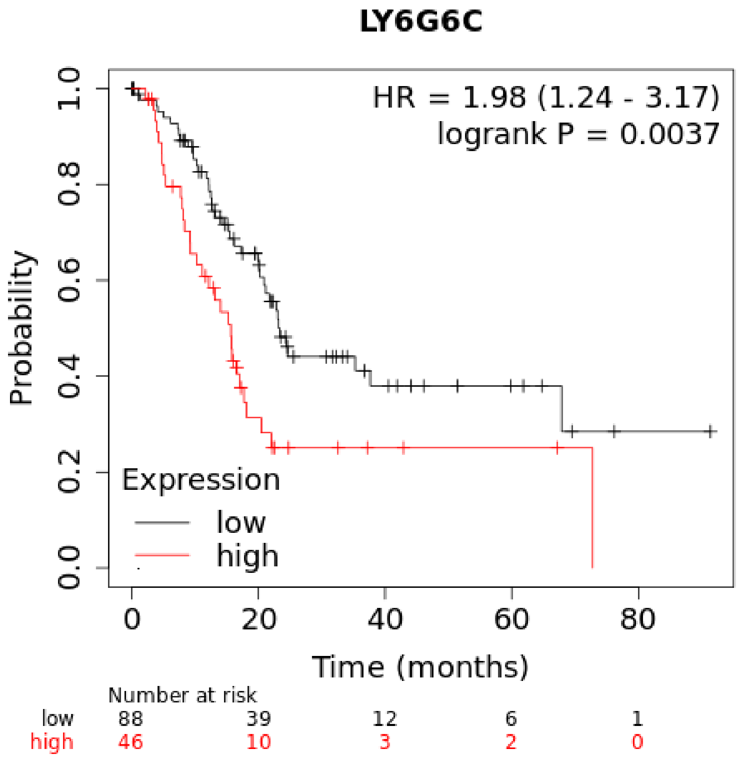

Click here to download the plot in TIFF format

Download plot as a PDF

Download p values vs. cutoff table

Median survival

Low expression cohort (months)	High expression cohort (months)
23.4	15.67

RNAseq ID: LY6G6D -
Survival: OS
Auto select best cutoff: checked
Follow up threshold: all
Censore at threshold: checked
Compute median over entire database: false
Cutoff value used in analysis: 0
Expression range of the probe: 0 - 2
Invert HR values below 1: not checked

Restrictions

Tumor type: Pancreatic ductal adenocarcinoma

Restrict analysis to subtypes...

Stage: all
Gender: all
Race: all
Grade: all
Mutation burden: all

Restrict analysis based on cellular content...

Basophils: all

B-cells:	all
CD4+ memory T-cells:	decreased
CD8+ T-cells:	all
Eosinophils:	all
Macrophages:	all
Mesenchymal stem cells:	all
Natural killer T-cells:	all
Regulatory T-cells:	all
Type 1 T-helper cells:	all
Type 2 T-helper cells:	all

Results

P value: 0.0016
FDR: 10%

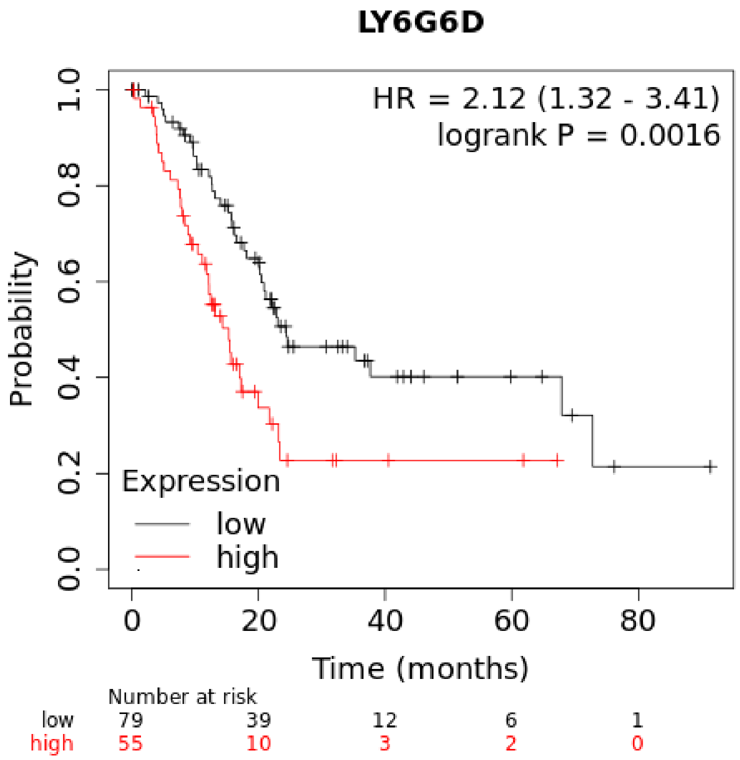

Click here to download the plot in TIFF format

Download plot as a PDF

Download p values vs. cutoff table

Median survival

Low expression cohort (months)	High expression cohort (months)
24.4	15.33

**RNAseq ID:**	LY6G6F -
**Survival:**	OS
**Auto select best cutoff:**	checked
**Follow up threshold:**	all
**Censore at threshold:**	checked
**Compute median over entire database:**	false
**Cutoff value used in analysis:**	0
**Expression range of the probe:**	0 - 5
**Invert HR values below 1:**	not checked

Restrictions

Tumor type: Pancreatic ductal adenocarcinoma

Restrict analysis to subtypes...

Stage:	all
Gender:	all
Race:	all
Grade:	all
Mutation burden:	all

Restrict analysis based on cellular content...

Basophils:	all
B-cells:	all
CD4+ memory T-cells:	decreased
CD8+ T-cells:	all
Eosinophils:	all
Macrophages:	all
Mesenchymal stem cells:	all
Natural killer T-cells:	all
Regulatory T-cells:	all
Type 1 T-helper cells:	all
Type 2 T-helper cells:	all

Results

P value: 9.8e-5
FDR: 2%

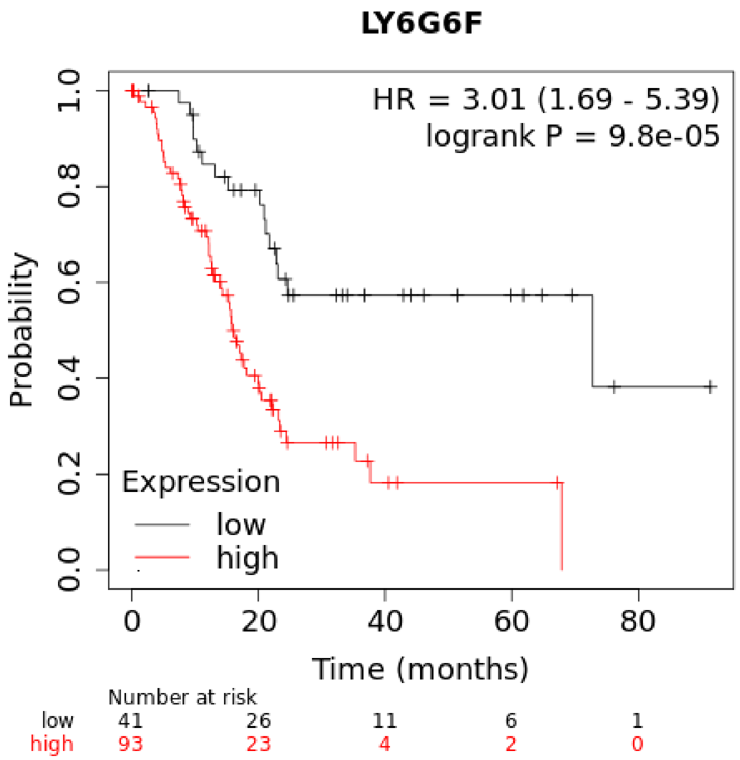

Click here to download the plot in TIFF format

Download plot as a PDF

Download p values vs. cutoff table

Median survival

Low expression cohort (months)	High expression cohort (months)
72.73	15.87

**RNAseq ID:**	LY6G5C -
**Survival:**	OS
**Auto select best cutoff:**	checked
**Follow up threshold:**	all
**Censore at threshold:**	checked
**Compute median over entire database:**	false
**Cutoff value used in analysis:**	50
**Expression range of the probe:**	15 - 586
**Invert HR values below 1:**	not checked

Restrictions

Tumor type: Pancreatic ductal adenocarcinoma

Restrict analysis to subtypes...

Stage:	all
Gender:	all
Race:	all
Grade:	all
Mutation burden:	all

Restrict analysis based on cellular content...

Basophils:	all
B-cells:	all
CD4+ memory T-cells:	decreased
CD8+ T-cells:	all
Eosinophils:	all
Macrophages:	all
Mesenchymal stem cells:	all
Natural killer T-cells:	all
Regulatory T-cells:	all
Type 1 T-helper cells:	all
Type 2 T-helper cells:	all

Results

**P value:**	6.0e-5
**FDR:**	2%

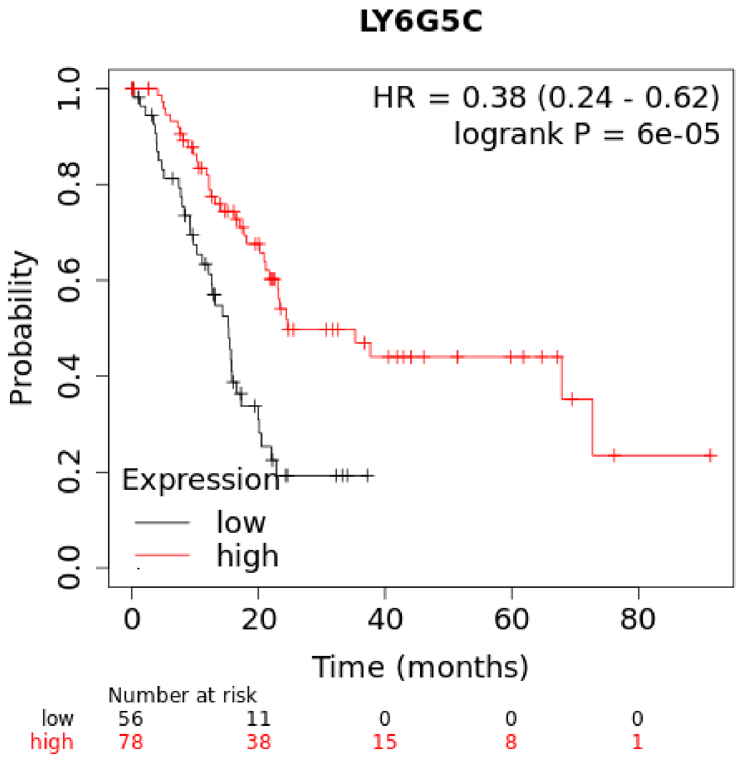

Click here to download the plot in TIFF format

Download plot as a PDF

Download p values vs. cutoff table

Median survival

Low expression cohort (months)	High expression cohort (months)
15.33	24.6

**RNAseq ID:**	LY6G5B -
**Survival:**	OS
**Auto select best cutoff:**	checked
**Follow up threshold:**	all
**Censore at threshold:**	checked
**Compute median over entire database:**	false
**Cutoff value used in analysis:**	37
**Expression range of the probe:**	3 - 192
**Invert HR values below 1:**	not checked

Restrictions

Tumor type: Pancreatic ductal adenocarcinoma

Restrict analysis to subtypes...

Stage:	all
Gender:	all
Race:	all
Grade:	all
Mutation burden:	all

Restrict analysis based on cellular content...

Basophils:	all

B-cells:	all
CD4+ memory T-cells:	decreased
CD8+ T-cells:	all
Eosinophils:	all
Macrophages:	all
Mesenchymal stem cells:	all
Natural killer T-cells:	all
Regulatory T-cells:	all
Type 1 T-helper cells:	all
Type 2 T-helper cells:	all

Results

P value: 0.009
FDR: 50%

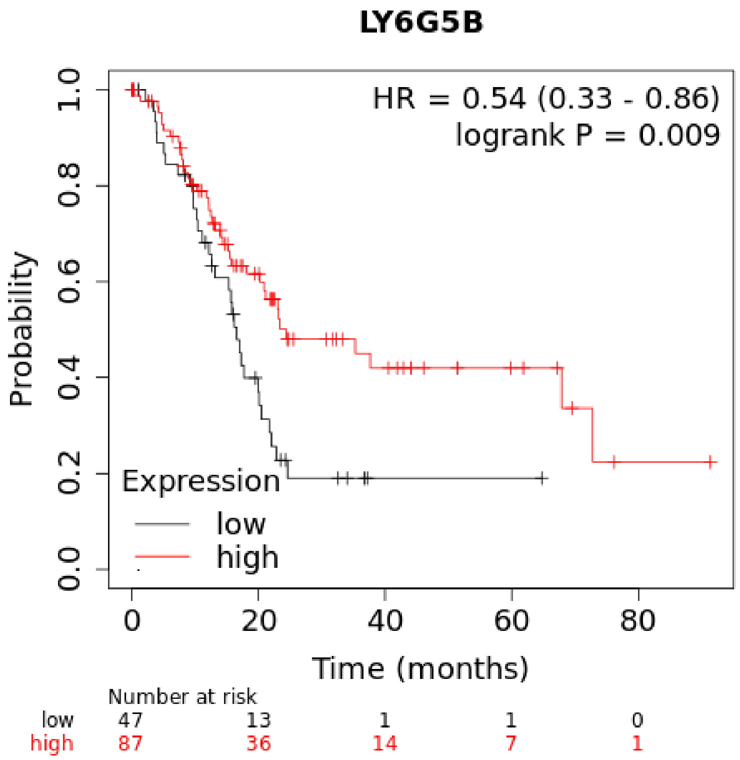

Click here to download the plot in TIFF format

Download plot as a PDF

Download p values vs. cutoff table

Median survival

Low expression cohort (months)	High expression cohort (months)
16.6	24.4

You can save the plots by right-clicking the image and then selecting "Save image as...". To generate a high resolution TIFF image, please adjust the "Settings" in the analysis page.

KMplot.com Copyright © 2009-2020 | Contact | Sitemap

Figure S8: KM plots and other raw data for the data depicted in Table 8

Pan-cancer

KM plotter | Home | Vote | Download | Updates | Contact

The desired RNAseq ID is valid: PSCA (-), LY6K (-), SLURP1 (-), LYPD2 (-), LY6D (-), GML (-), LY6E (-), LY6L (-), LY6H (-), GPIHBP1 (-), LYPD4 (-), CD177 (-), TEX101 (-), LYPD3 (-), PINLYP (-), PLAUR (-), LYPD5 (-), SPACA4 (-), ACRV1 (-), PATE1 (-), PATE2 (-), PATE3 (-), PATE4 (-), CD59 (-), LY6G6C (-), LY6G6D (-), LY6G6F (-), LY6G5C (-), LY6G5B (-),

**RNAseq ID:**	PSCA -
**Survival:**	OS
**Auto select best cutoff:**	checked
**Follow up threshold:**	all
**Censore at threshold:**	checked
**Compute median over entire database:**	false
**Cutoff value used in analysis:**	195
**Expression range of the probe:**	4 - 46575
**Invert HR values below 1:**	not checked

Restrictions

Tumor type: Pancreatic ductal adenocarcinoma

Restrict analysis to subtypes...

Stage:	all
Gender:	all
Race:	all
Grade:	all
Mutation burden:	all

Restrict analysis based on cellular content...

Basophils:	all
B-cells:	enriched
CD4+ memory T-cells:	all
CD8+ T-cells:	all
Eosinophils:	all
Macrophages:	all
Mesenchymal stem cells:	all
Natural killer T-cells:	all
Regulatory T-cells:	all
Type 1 T-helper cells:	all
Type 2 T-helper cells:	all

Results

**P value:**	0.0152
**FDR:**	over 50%

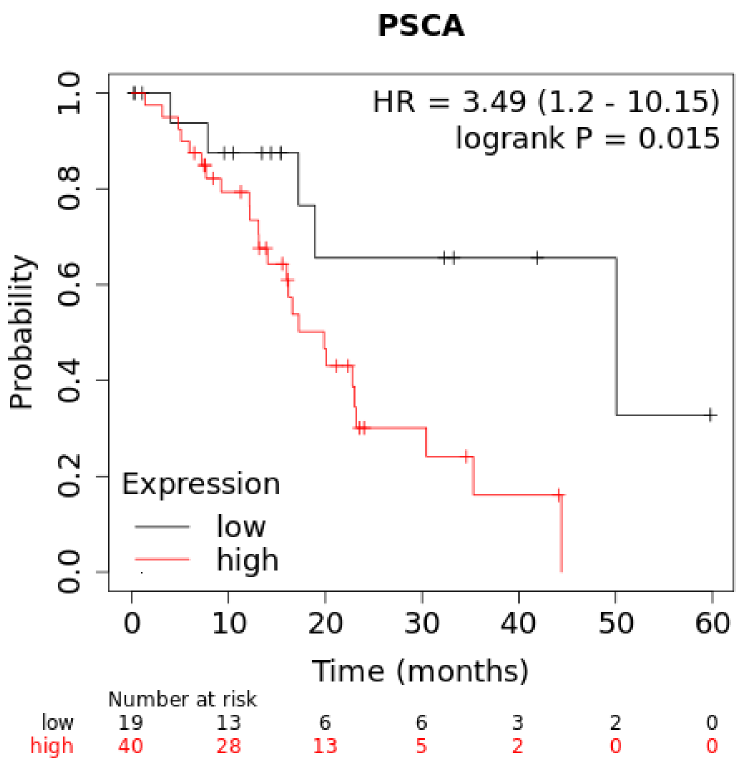

Click here to download the plot in TIFF format

Download plot as a PDF

Download p values vs. cutoff table

Median survival

Low expression cohort (months)	High expression cohort (months)
50.07	19.87

**RNAseq ID:**	LY6K -
**Survival:**	OS
**Auto select best cutoff:**	checked
**Follow up threshold:**	all
**Censore at threshold:**	checked
**Compute median over entire database:**	false
**Cutoff value used in analysis:**	10
**Expression range of the probe:**	0 - 1332
**Invert HR values below 1:**	not checked

Restrictions

Tumor type: Pancreatic ductal adenocarcinoma

Restrict analysis to subtypes...

Stage:	all
Gender:	all
Race:	all
Grade:	all
Mutation burden:	all

Restrict analysis based on cellular content...

Basophils:	all

B-cells:	enriched
CD4+ memory T-cells:	all
CD8+ T-cells:	all
Eosinophils:	all
Macrophages:	all
Mesenchymal stem cells:	all
Natural killer T-cells:	all
Regulatory T-cells:	all
Type 1 T-helper cells:	all
Type 2 T-helper cells:	all

Results

P value: 0.1842
FDR: 100%

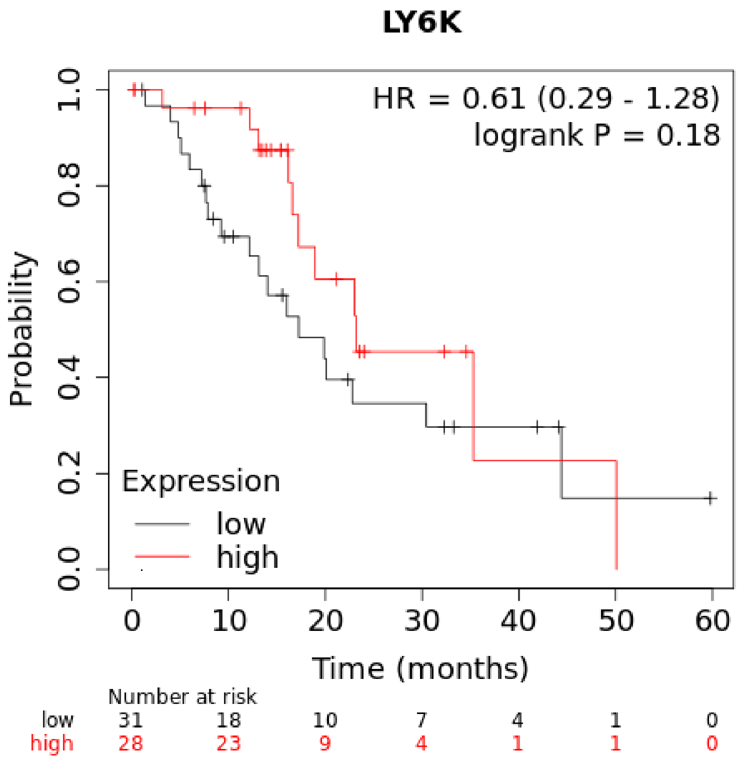

Click here to download the plot in TIFF format

Download plot as a PDF

Download p values vs. cutoff table

Median survival

Low expression cohort (months)	High expression cohort (months)
17.27	23.17

**RNAseq ID:**	SLURP1 -
**Survival:**	OS
**Auto select best cutoff:**	checked
**Follow up threshold:**	all
**Censore at threshold:**	checked
**Compute median over entire database:**	false
**Cutoff value used in analysis:**	1
**Expression range of the probe:**	0 - 279
**Invert HR values below 1:**	not checked

Restrictions

Tumor type: Pancreatic ductal adenocarcinoma

Restrict analysis to subtypes...

Stage:	all
Gender:	all
Race:	all
Grade:	all
Mutation burden:	all

Restrict analysis based on cellular content...

Basophils:	all
B-cells:	enriched
CD4+ memory T-cells:	all
CD8+ T-cells:	all
Eosinophils:	all
Macrophages:	all
Mesenchymal stem cells:	all
Natural killer T-cells:	all
Regulatory T-cells:	all
Type 1 T-helper cells:	all
Type 2 T-helper cells:	all

Results

P value: 0.0242
FDR: over 50%

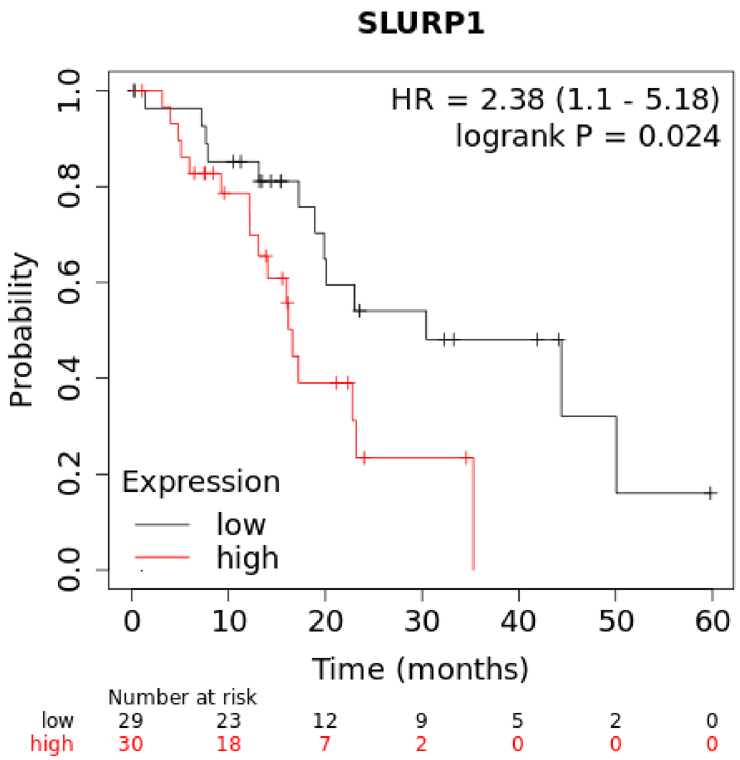

Click here to download the plot in TIFF format

Download plot as a PDF

Download p values vs. cutoff table

Median survival

Low expression cohort (months)	High expression cohort (months)
30.43	16.6

**RNAseq ID:**	LYPD2 -
**Survival:**	OS
**Auto select best cutoff:**	checked
**Follow up threshold:**	all
**Censore at threshold:**	checked
**Compute median over entire database:**	false
**Cutoff value used in analysis:**	35
**Expression range of the probe:**	0 - 2799
**Invert HR values below 1:**	not checked

Restrictions

Tumor type: Pancreatic ductal adenocarcinoma

Restrict analysis to subtypes...

Stage:	all
Gender:	all
Race:	all
Grade:	all
Mutation burden:	all

Restrict analysis based on cellular content...

Basophils:	all
B-cells:	enriched
CD4+ memory T-cells:	all
CD8+ T-cells:	all
Eosinophils:	all
Macrophages:	all
Mesenchymal stem cells:	all
Natural killer T-cells:	all
Regulatory T-cells:	all
Type 1 T-helper cells:	all
Type 2 T-helper cells:	all

Results

**P value:**	0.0967
**FDR:**	100%

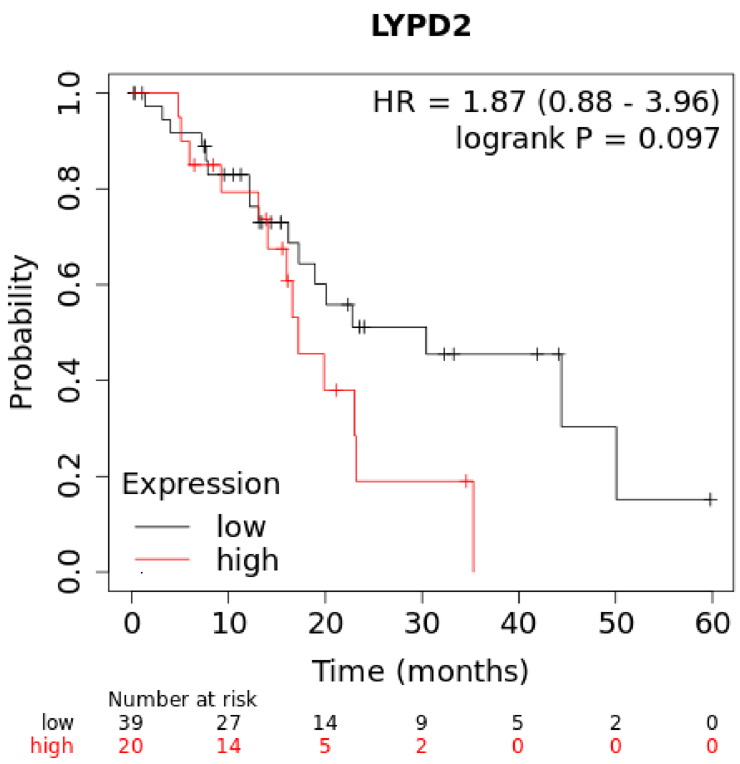

Click here to download the plot in TIFF format

Download plot as a PDF

Download p values vs. cutoff table

Median survival

Low expression cohort (months)	High expression cohort (months)
30.43	17.23

RNAseq ID: LY6D -
Survival: OS
Auto select best cutoff: checked
Follow up threshold: all
Censore at threshold: checked
Compute median over entire database: false
Cutoff value used in analysis: 160
Expression range of the probe: 0 - 18030
Invert HR values below 1: not checked

Restrictions

Tumor type: Pancreatic ductal adenocarcinoma

Restrict analysis to subtypes...

Stage: all
Gender: all
Race: all
Grade: all
Mutation burden: all

Restrict analysis based on cellular content...

Basophils: all

B-cells:	enriched
CD4+ memory T-cells:	all
CD8+ T-cells:	all
Eosinophils:	all
Macrophages:	all
Mesenchymal stem cells:	all
Natural killer T-cells:	all
Regulatory T-cells:	all
Type 1 T-helper cells:	all
Type 2 T-helper cells:	all

Results

P value: 3.0e-5
FDR: 1%

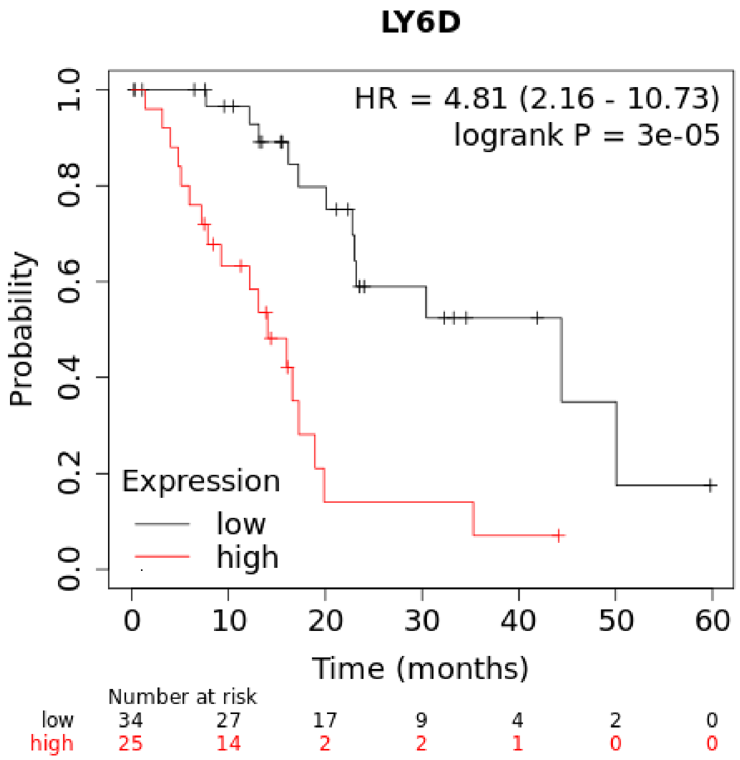

Click here to download the plot in TIFF format

Download plot as a PDF

Download p values vs. cutoff table

Median survival

Low expression cohort (months)	High expression cohort (months)
44.4	14.03

**RNAseq ID:**	GML -
**Survival:**	OS
**Auto select best cutoff:**	checked
**Follow up threshold:**	all
**Censore at threshold:**	checked
**Compute median over entire database:**	false
**Cutoff value used in analysis:**	0
**Expression range of the probe:**	0 - 3
**Invert HR values below 1:**	not checked

Restrictions

Tumor type: Pancreatic ductal adenocarcinoma

Restrict analysis to subtypes...

Stage:	all
Gender:	all
Race:	all
Grade:	all
Mutation burden:	all

Restrict analysis based on cellular content...

Basophils:	all
B-cells:	enriched
CD4+ memory T-cells:	all
CD8+ T-cells:	all
Eosinophils:	all
Macrophages:	all
Mesenchymal stem cells:	all
Natural killer T-cells:	all
Regulatory T-cells:	all
Type 1 T-helper cells:	all
Type 2 T-helper cells:	all

Results

P value: 0.3734
FDR: 100%

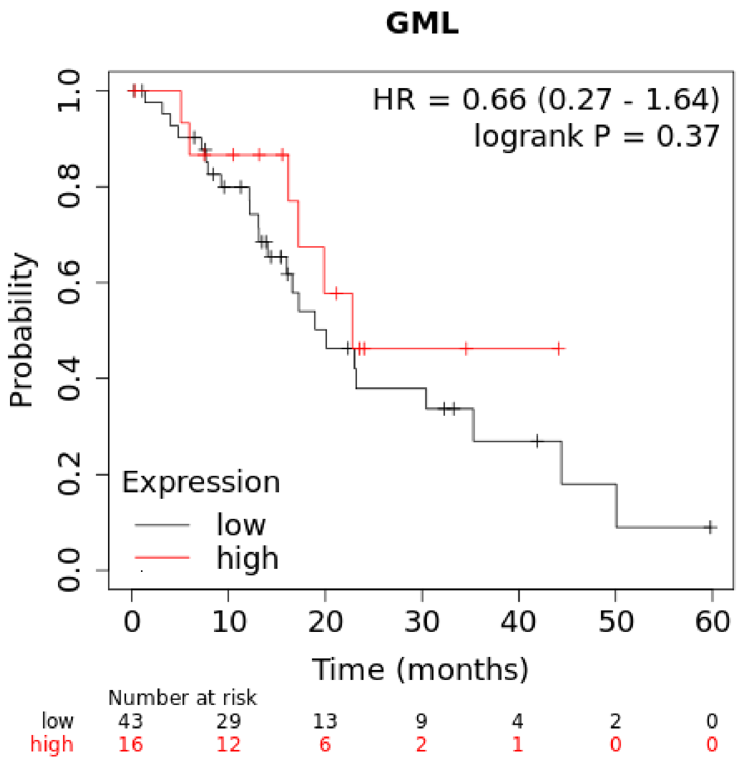

Click here to download the plot in TIFF format

Download plot as a PDF

Download p values vs. cutoff table

Median survival

Low expression cohort (months)	High expression cohort (months)
20.1	22.8

**RNAseq ID:**	LY6E -
**Survival:**	OS
**Auto select best cutoff:**	checked
**Follow up threshold:**	all
**Censore at threshold:**	checked
**Compute median over entire database:**	false
**Cutoff value used in analysis:**	9941
**Expression range of the probe:**	254 - 56404
**Invert HR values below 1:**	not checked

Restrictions

Tumor type: Pancreatic ductal adenocarcinoma

Restrict analysis to subtypes...

Stage:	all
Gender:	all
Race:	all
Grade:	all
Mutation burden:	all

Restrict analysis based on cellular content...

Basophils:	all
B-cells:	enriched
CD4+ memory T-cells:	all
CD8+ T-cells:	all
Eosinophils:	all
Macrophages:	all
Mesenchymal stem cells:	all
Natural killer T-cells:	all
Regulatory T-cells:	all
Type 1 T-helper cells:	all
Type 2 T-helper cells:	all

Results

**P value:**	0.006
**FDR:**	over 50%

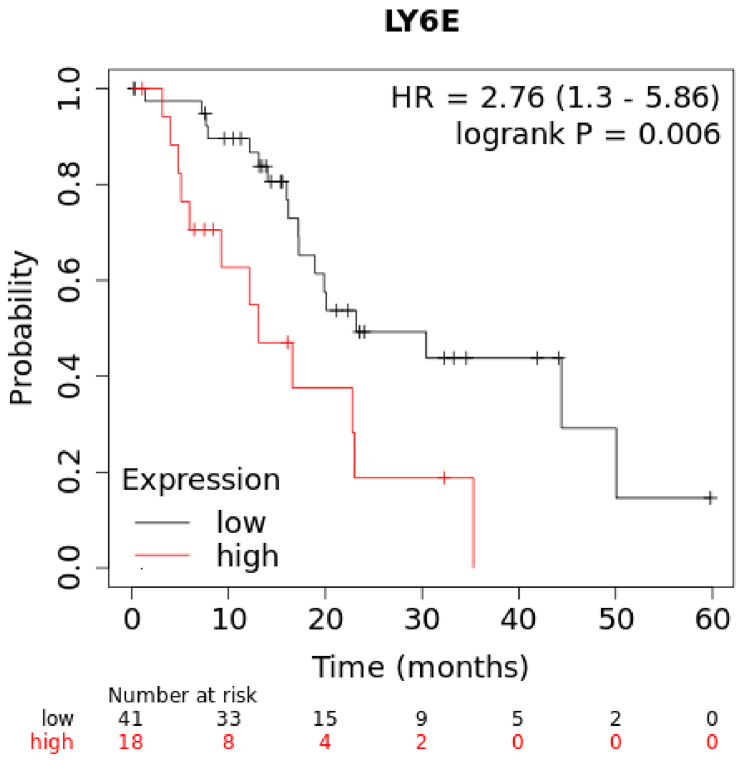

Click here to download the plot in TIFF format

Download plot as a PDF

Download p values vs. cutoff table

Median survival

Low expression cohort (months)	High expression cohort (months)
23.17	13.1

RNAseq ID: LY6L -
Survival: OS
Auto select best cutoff: checked
Follow up threshold: all
Censore at threshold: checked
Compute median over entire database: false
Cutoff value used in analysis: 0
Expression range of the probe: 0 - 6
Invert HR values below 1: not checked

Restrictions

Tumor type: Pancreatic ductal adenocarcinoma

Restrict analysis to subtypes...

Stage: all
Gender: all
Race: all
Grade: all
Mutation burden: all

Restrict analysis based on cellular content...

Basophils: all

B-cells:	enriched
CD4+ memory T-cells:	all
CD8+ T-cells:	all
Eosinophils:	all
Macrophages:	all
Mesenchymal stem cells:	all
Natural killer T-cells:	all
Regulatory T-cells:	all
Type 1 T-helper cells:	all
Type 2 T-helper cells:	all

Results

P value: 0.2433
FDR: 100%

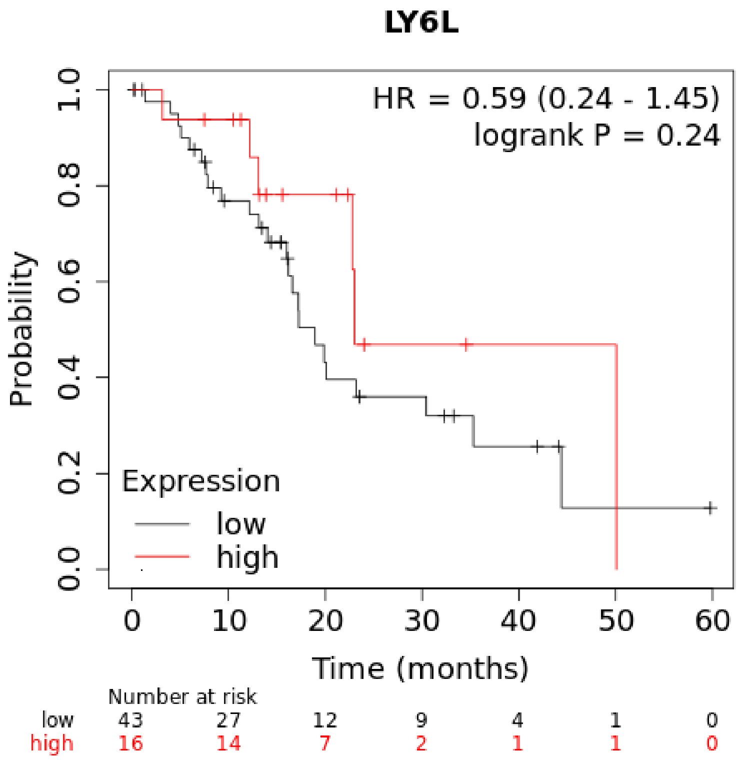

Click here to download the plot in TIFF format

Download plot as a PDF

Download p values vs. cutoff table

Median survival

Low expression cohort (months)	High expression cohort (months)
18.93	23.03

**RNAseq ID:**	LY6H -
**Survival:**	OS
**Auto select best cutoff:**	checked
**Follow up threshold:**	all
**Censore at threshold:**	checked
**Compute median over entire database:**	false
**Cutoff value used in analysis:**	34
**Expression range of the probe:**	4 - 6037
**Invert HR values below 1:**	not checked

Restrictions

Tumor type: Pancreatic ductal adenocarcinoma

Restrict analysis to subtypes...

Stage:	all
Gender:	all
Race:	all
Grade:	all
Mutation burden:	all

Restrict analysis based on cellular content...

Basophils:	all
B-cells:	enriched
CD4+ memory T-cells:	all
CD8+ T-cells:	all
Eosinophils:	all
Macrophages:	all
Mesenchymal stem cells:	all
Natural killer T-cells:	all
Regulatory T-cells:	all
Type 1 T-helper cells:	all
Type 2 T-helper cells:	all

Results

P value: 0.3599
FDR: 100%

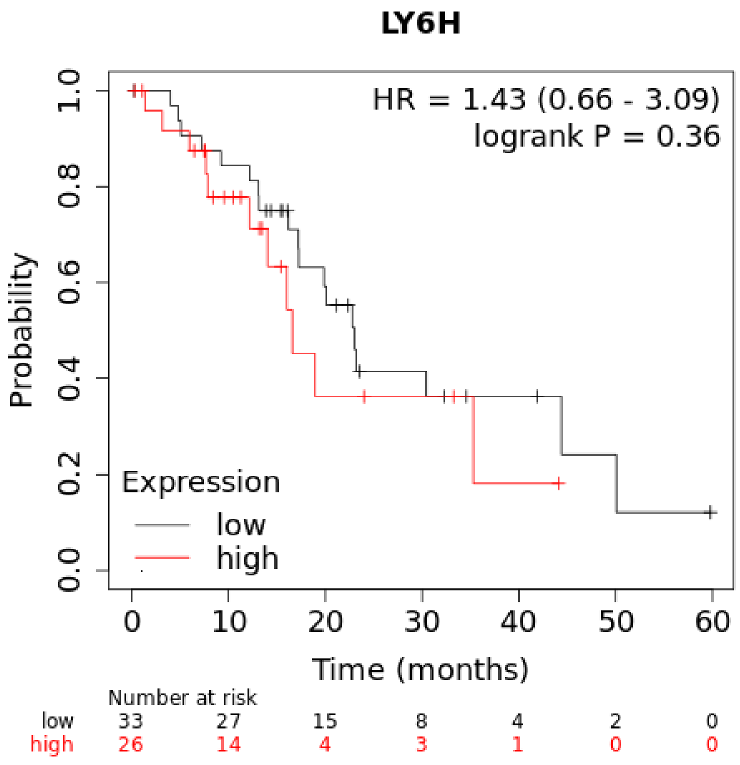

Click here to download the plot in TIFF format

Download plot as a PDF

Download p values vs. cutoff table

Median survival

Low expression cohort (months)	High expression cohort (months)
23.03	16.6

**RNAseq ID:**	GPIHBP1 -
**Survival:**	OS
**Auto select best cutoff:**	checked
**Follow up threshold:**	all
**Censore at threshold:**	checked
**Compute median over entire database:**	false
**Cutoff value used in analysis:**	32
**Expression range of the probe:**	8 - 323
**Invert HR values below 1:**	not checked

Restrictions

Tumor type: Pancreatic ductal adenocarcinoma

Restrict analysis to subtypes...

Stage:	all
Gender:	all
Race:	all
Grade:	all
Mutation burden:	all

Restrict analysis based on cellular content...

Basophils:	all
B-cells:	enriched
CD4+ memory T-cells:	all
CD8+ T-cells:	all
Eosinophils:	all
Macrophages:	all
Mesenchymal stem cells:	all
Natural killer T-cells:	all
Regulatory T-cells:	all
Type 1 T-helper cells:	all
Type 2 T-helper cells:	all

Results

**P value:**	0.0942
**FDR:**	100%

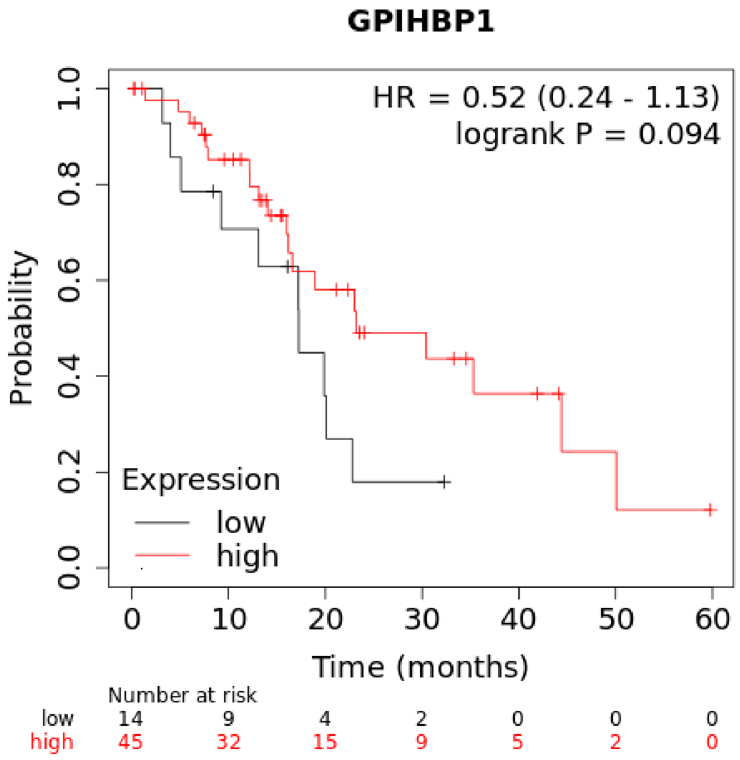

Click here to download the plot in TIFF format

Download plot as a PDF

Download p values vs. cutoff table

Median survival

Low expression cohort (months)	High expression cohort (months)
17.27	23.17

**RNAseq ID:**	LYPD4 -
**Survival:**	OS
**Auto select best cutoff:**	checked
**Follow up threshold:**	all
**Censore at threshold:**	checked
**Compute median over entire database:**	false
**Cutoff value used in analysis:**	0
**Expression range of the probe:**	0 - 8
**Invert HR values below 1:**	not checked

Restrictions

Tumor type: Pancreatic ductal adenocarcinoma

Restrict analysis to subtypes...

Stage:	all
Gender:	all
Race:	all
Grade:	all
Mutation burden:	all

Restrict analysis based on cellular content...

Basophils:	all

B-cells:	enriched
CD4+ memory T-cells:	all
CD8+ T-cells:	all
Eosinophils:	all
Macrophages:	all
Mesenchymal stem cells:	all
Natural killer T-cells:	all
Regulatory T-cells:	all
Type 1 T-helper cells:	all
Type 2 T-helper cells:	all

Results

P value: 0.0525
FDR: 100%

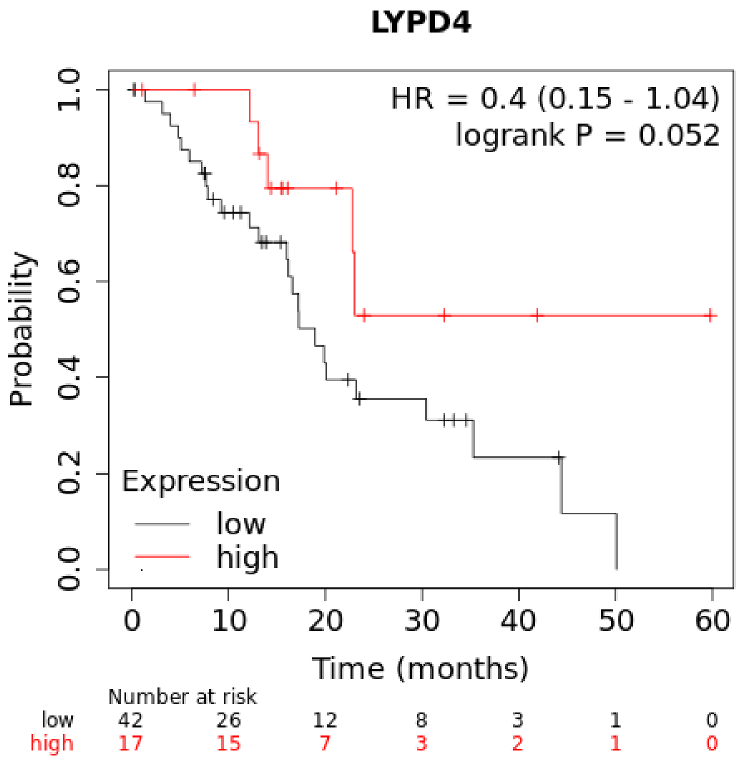

Click here to download the plot in TIFF format

Download plot as a PDF

Download p values vs. cutoff table

Upper quartile survival

Low expression cohort (months)	High expression cohort (months)
9.23	22.8

**RNAseq ID:**	CD177 -
**Survival:**	OS
**Auto select best cutoff:**	checked
**Follow up threshold:**	all
**Censore at threshold:**	checked
**Compute median over entire database:**	false
**Cutoff value used in analysis:**	54
**Expression range of the probe:**	0 - 6100
**Invert HR values below 1:**	not checked

Restrictions

Tumor type: Pancreatic ductal adenocarcinoma

Restrict analysis to subtypes...

Stage:	all
Gender:	all
Race:	all
Grade:	all
Mutation burden:	all

Restrict analysis based on cellular content...

Basophils:	all
B-cells:	enriched
CD4+ memory T-cells:	all
CD8+ T-cells:	all
Eosinophils:	all
Macrophages:	all
Mesenchymal stem cells:	all
Natural killer T-cells:	all
Regulatory T-cells:	all
Type 1 T-helper cells:	all
Type 2 T-helper cells:	all

Results

P value: 0.1114
FDR: 100%

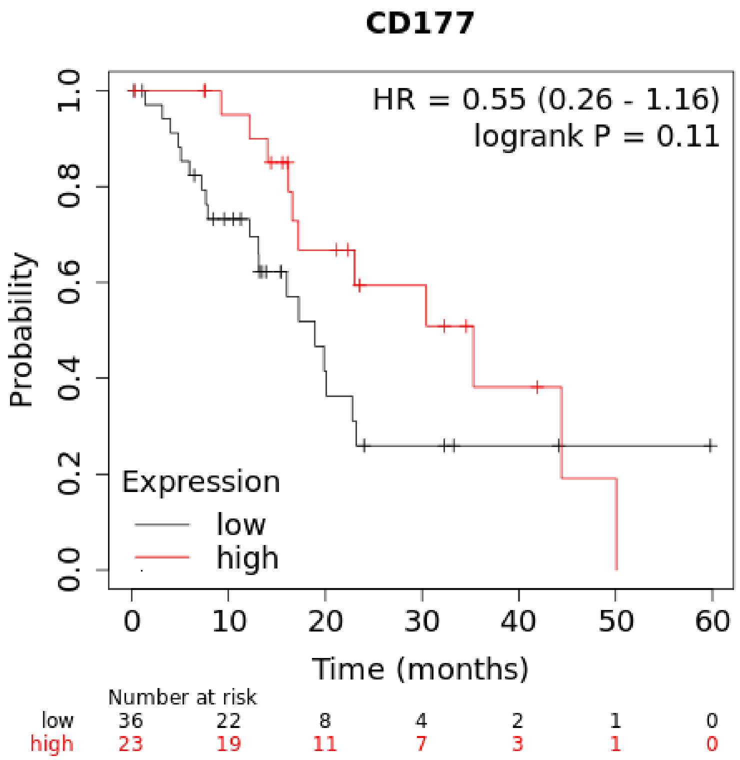

Click here to download the plot in TIFF format

Download plot as a PDF

Download p values vs. cutoff table

Median survival

Low expression cohort (months)	High expression cohort (months)
18.93	35.3

**RNAseq ID:**	TEX101 -
**Survival:**	OS
**Auto select best cutoff:**	checked
**Follow up threshold:**	all
**Censore at threshold:**	checked
**Compute median over entire database:**	false
**Cutoff value used in analysis:**	1
**Expression range of the probe:**	0 - 149
**Invert HR values below 1:**	not checked

Restrictions

Tumor type: Pancreatic ductal adenocarcinoma

Restrict analysis to subtypes...

Stage:	all
Gender:	all
Race:	all
Grade:	all
Mutation burden:	all

Restrict analysis based on cellular content...

Basophils:	all
B-cells:	enriched
CD4+ memory T-cells:	all
CD8+ T-cells:	all
Eosinophils:	all
Macrophages:	all
Mesenchymal stem cells:	all
Natural killer T-cells:	all
Regulatory T-cells:	all
Type 1 T-helper cells:	all
Type 2 T-helper cells:	all

Results

**P value:**	0.2584
**FDR:**	100%

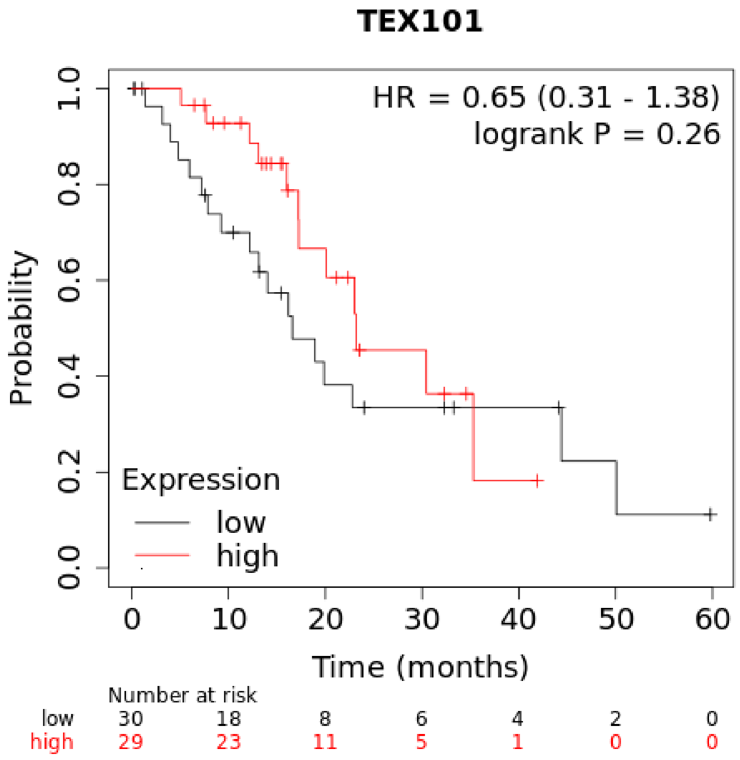

Click here to download the plot in TIFF format

Download plot as a PDF

Download p values vs. cutoff table

Median survival

Low expression cohort (months)	High expression cohort (months)
16.6	23.17

**RNAseq ID:**	LYPD3 -
**Survival:**	OS
**Auto select best cutoff:**	checked
**Follow up threshold:**	all
**Censore at threshold:**	checked
**Compute median over entire database:**	false
**Cutoff value used in analysis:**	290
**Expression range of the probe:**	19 - 2702
**Invert HR values below 1:**	not checked

Restrictions

Tumor type: Pancreatic ductal adenocarcinoma

Restrict analysis to subtypes...

Stage:	all
Gender:	all
Race:	all
Grade:	all
Mutation burden:	all

Restrict analysis based on cellular content...

Basophils:	all

B-cells:	enriched
CD4+ memory T-cells:	all
CD8+ T-cells:	all
Eosinophils:	all
Macrophages:	all
Mesenchymal stem cells:	all
Natural killer T-cells:	all
Regulatory T-cells:	all
Type 1 T-helper cells:	all
Type 2 T-helper cells:	all

Results

P value: 0.4414
FDR: 100%

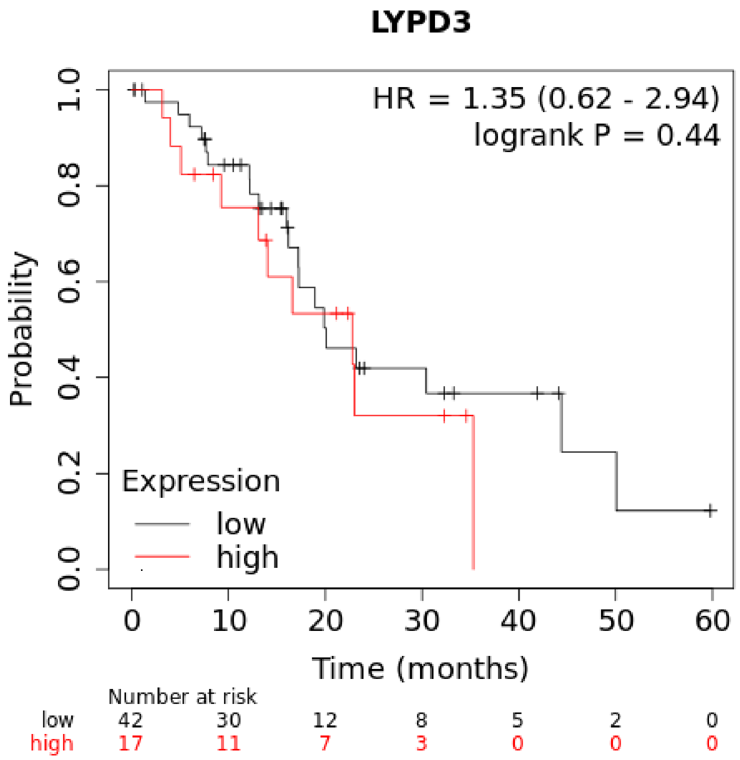

Click here to download the plot in TIFF format

Download plot as a PDF

Download p values vs. cutoff table

Median survival

Low expression cohort (months)	High expression cohort (months)
20.1	22.8

**RNAseq ID:**	PINLYP -
**Survival:**	OS
**Auto select best cutoff:**	checked
**Follow up threshold:**	all
**Censore at threshold:**	checked
**Compute median over entire database:**	false
**Cutoff value used in analysis:**	76
**Expression range of the probe:**	22 - 157
**Invert HR values below 1:**	not checked

Restrictions

Tumor type: Pancreatic ductal adenocarcinoma

Restrict analysis to subtypes...

Stage:	all
Gender:	all
Race:	all
Grade:	all
Mutation burden:	all

Restrict analysis based on cellular content...

Basophils:	all
B-cells:	enriched
CD4+ memory T-cells:	all
CD8+ T-cells:	all
Eosinophils:	all
Macrophages:	all
Mesenchymal stem cells:	all
Natural killer T-cells:	all
Regulatory T-cells:	all
Type 1 T-helper cells:	all
Type 2 T-helper cells:	all

Results

P value: 0.1182
FDR: 100%

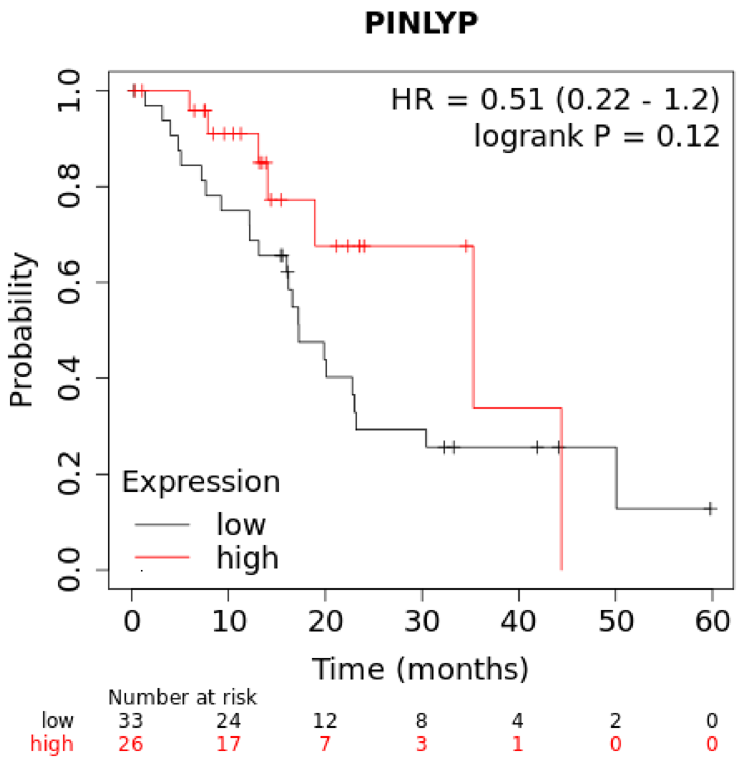

Click here to download the plot in TIFF format

Download plot as a PDF

Download p values vs. cutoff table

Median survival

Low expression cohort (months)	High expression cohort (months)
17.27	35.3

**RNAseq ID:**	PLAUR -
**Survival:**	OS
**Auto select best cutoff:**	checked
**Follow up threshold:**	all
**Censore at threshold:**	checked
**Compute median over entire database:**	false
**Cutoff value used in analysis:**	2095
**Expression range of the probe:**	47 - 14016
**Invert HR values below 1:**	not checked

Restrictions

Tumor type: Pancreatic ductal adenocarcinoma

Restrict analysis to subtypes...

Stage:	all
Gender:	all
Race:	all
Grade:	all
Mutation burden:	all

Restrict analysis based on cellular content...

Basophils:	all
B-cells:	enriched
CD4+ memory T-cells:	all
CD8+ T-cells:	all
Eosinophils:	all
Macrophages:	all
Mesenchymal stem cells:	all
Natural killer T-cells:	all
Regulatory T-cells:	all
Type 1 T-helper cells:	all
Type 2 T-helper cells:	all

Results

**P value:**	0.0433
**FDR:**	over 50%

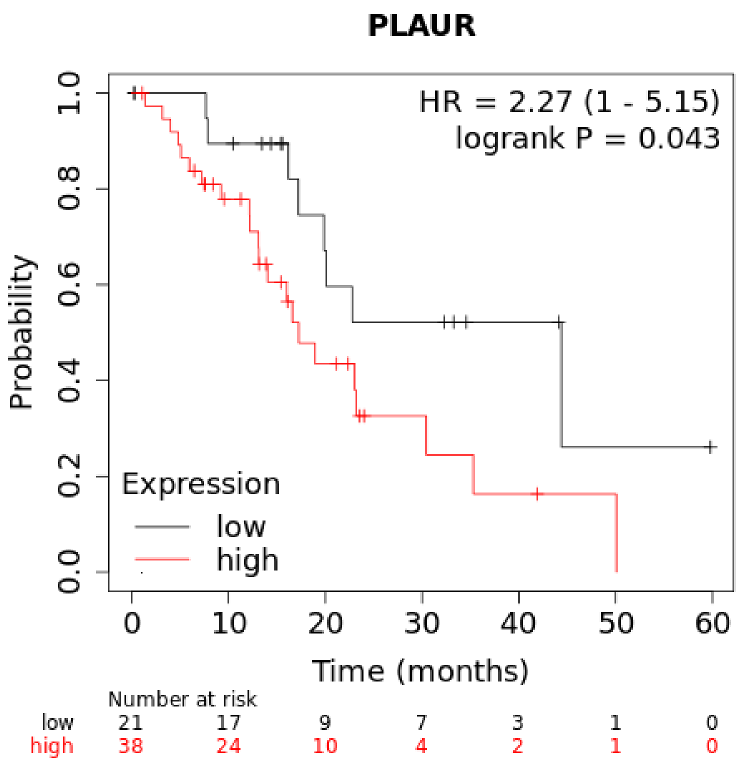

Click here to download the plot in TIFF format

Download plot as a PDF

Download p values vs. cutoff table

Median survival

Low expression cohort (months)	High expression cohort (months)
44.4	17.27

**RNAseq ID:**	LYPD5 -
**Survival:**	OS
**Auto select best cutoff:**	checked
**Follow up threshold:**	all
**Censore at threshold:**	checked
**Compute median over entire database:**	false
**Cutoff value used in analysis:**	74
**Expression range of the probe:**	1 - 578
**Invert HR values below 1:**	not checked

Restrictions

Tumor type: Pancreatic ductal adenocarcinoma

Restrict analysis to subtypes...

Stage:	all
Gender:	all
Race:	all
Grade:	all
Mutation burden:	all

Restrict analysis based on cellular content...

Basophils:	all

B-cells:	enriched
CD4+ memory T-cells:	all
CD8+ T-cells:	all
Eosinophils:	all
Macrophages:	all
Mesenchymal stem cells:	all
Natural killer T-cells:	all
Regulatory T-cells:	all
Type 1 T-helper cells:	all
Type 2 T-helper cells:	all

Results

P value: 0.0355
FDR: over 50%

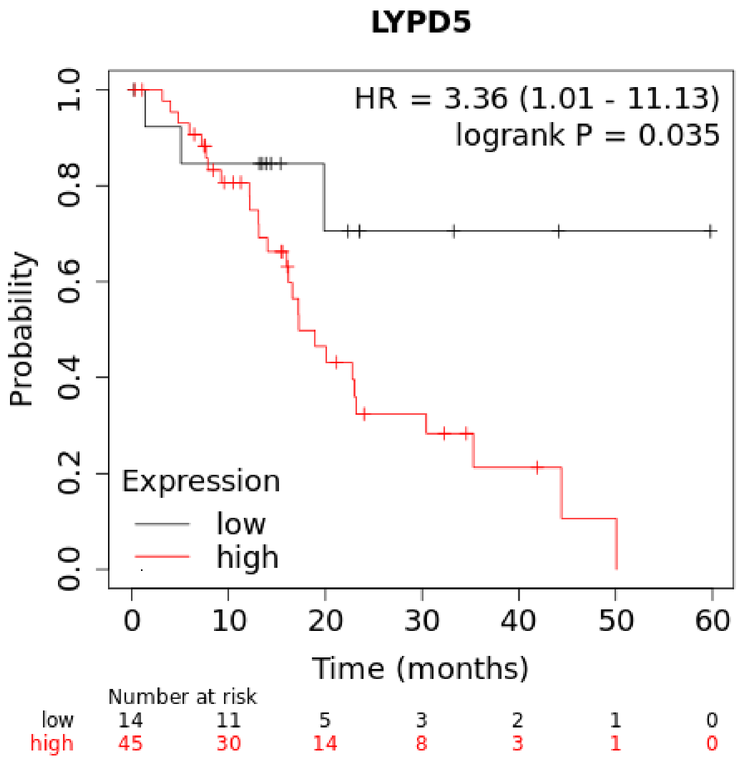

Click here to download the plot in TIFF format

Download plot as a PDF

Download p values vs. cutoff table

Upper quartile survival

Low expression cohort (months)	High expression cohort (months)
19.87	12.2

**RNAseq ID:**	SPACA4 -
**Survival:**	OS
**Auto select best cutoff:**	checked
**Follow up threshold:**	all
**Censore at threshold:**	checked
**Compute median over entire database:**	false
**Cutoff value used in analysis:**	19
**Expression range of the probe:**	1 - 120
**Invert HR values below 1:**	not checked

Restrictions

Tumor type: Pancreatic ductal adenocarcinoma

Restrict analysis to subtypes...

Stage:	all
Gender:	all
Race:	all
Grade:	all
Mutation burden:	all

Restrict analysis based on cellular content...

Basophils:	all
B-cells:	enriched
CD4+ memory T-cells:	all
CD8+ T-cells:	all
Eosinophils:	all
Macrophages:	all
Mesenchymal stem cells:	all
Natural killer T-cells:	all
Regulatory T-cells:	all
Type 1 T-helper cells:	all
Type 2 T-helper cells:	all

Results

P value: 0.0115
FDR: over 50%

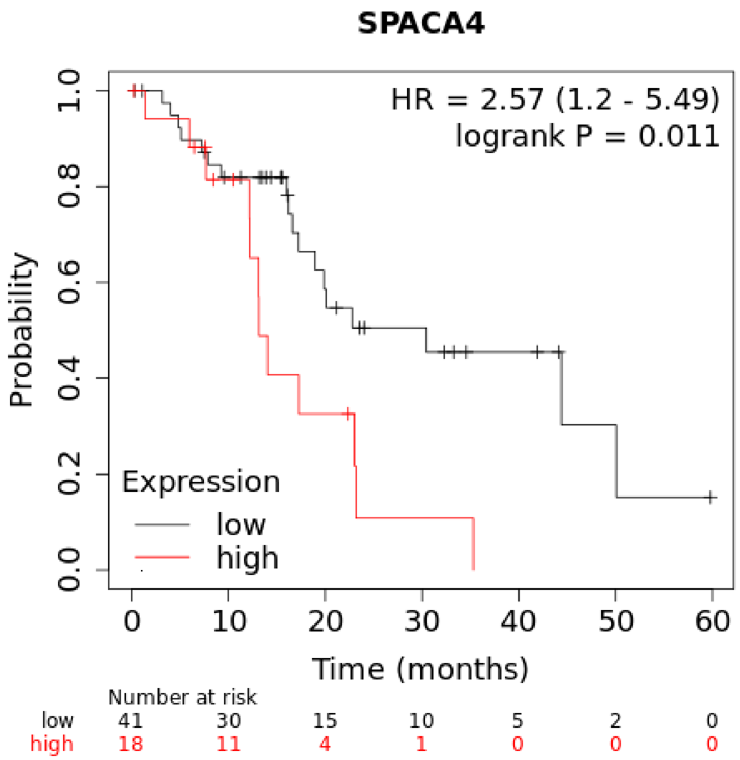

Click here to download the plot in TIFF format

Download plot as a PDF

Download p values vs. cutoff table

Median survival

Low expression cohort (months)	High expression cohort (months)
30.43	13.13

**RNAseq ID:**	ACRV1 -
**Survival:**	OS
**Auto select best cutoff:**	checked
**Follow up threshold:**	all
**Censore at threshold:**	checked
**Compute median over entire database:**	false
**Cutoff value used in analysis:**	2
**Expression range of the probe:**	0 - 15
**Invert HR values below 1:**	not checked

Restrictions

Tumor type: Pancreatic ductal adenocarcinoma

Restrict analysis to subtypes...

Stage:	all
Gender:	all
Race:	all
Grade:	all
Mutation burden:	all

Restrict analysis based on cellular content...

Basophils:	all
B-cells:	enriched
CD4+ memory T-cells:	all
CD8+ T-cells:	all
Eosinophils:	all
Macrophages:	all
Mesenchymal stem cells:	all
Natural killer T-cells:	all
Regulatory T-cells:	all
Type 1 T-helper cells:	all
Type 2 T-helper cells:	all

Results

**P value:**	0.236
**FDR:**	100%

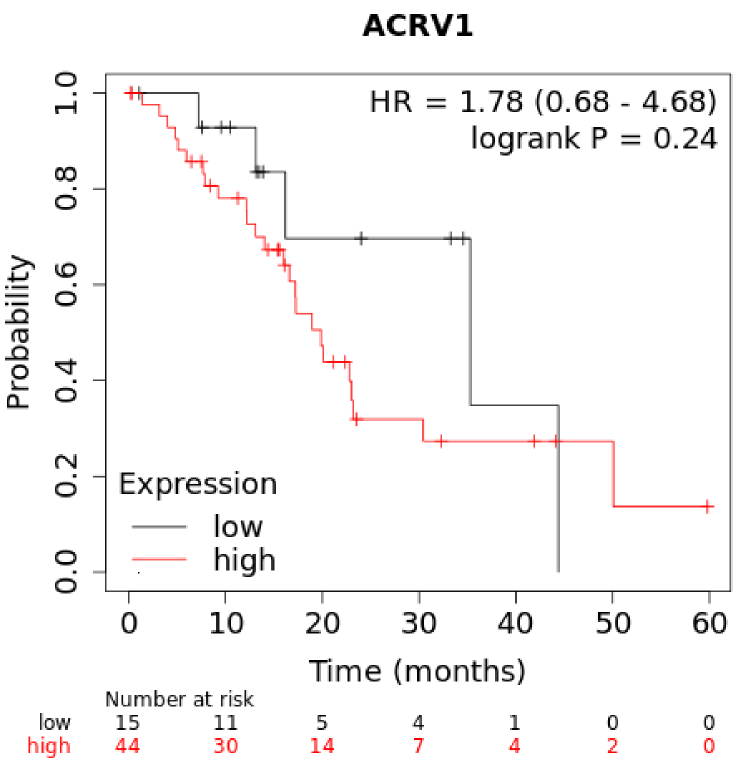

Click here to download the plot in TIFF format

Download plot as a PDF

Download p values vs. cutoff table

Median survival

Low expression cohort (months)	High expression cohort (months)
35.3	19.87

RNAseq ID: PATE1 -
Survival: OS
Auto select best cutoff: checked
Follow up threshold: all
Censore at threshold: checked
Compute median over entire database: false
Cutoff value used in analysis: 0
Expression range of the probe: 0 - 1
Invert HR values below 1: not checked

Restrictions

Tumor type: Pancreatic ductal adenocarcinoma

Restrict analysis to subtypes...

Stage: all
Gender: all
Race: all
Grade: all
Mutation burden: all

Restrict analysis based on cellular content...

Basophils: all

B-cells:	enriched
CD4+ memory T-cells:	all
CD8+ T-cells:	all
Eosinophils:	all
Macrophages:	all
Mesenchymal stem cells:	all
Natural killer T-cells:	all
Regulatory T-cells:	all
Type 1 T-helper cells:	all
Type 2 T-helper cells:	all

Results

P value: 0.6189
FDR: 100%

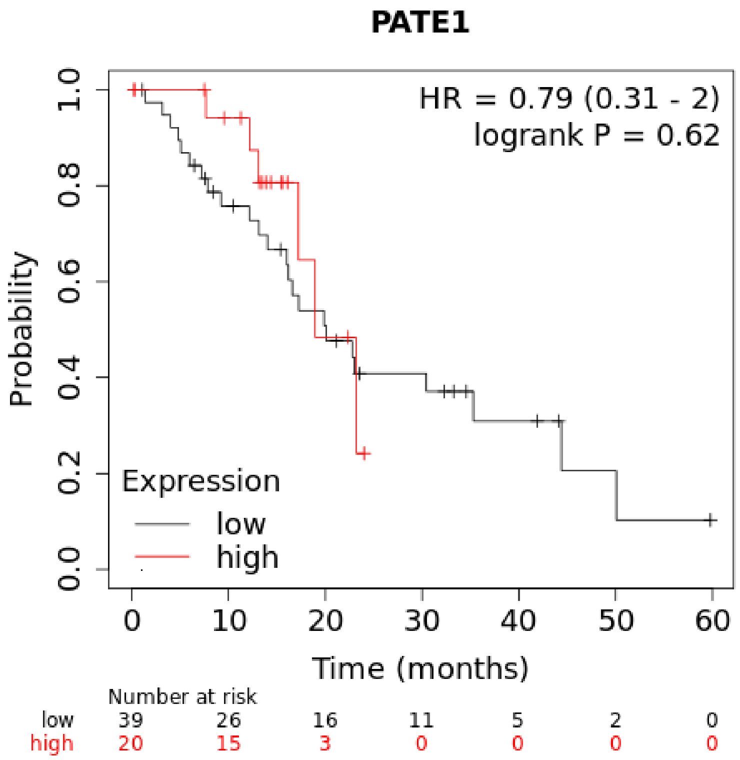

Click here to download the plot in TIFF format

Download plot as a PDF

Download p values vs. cutoff table

Median survival

Low expression cohort (months)	High expression cohort (months)
20.1	18.93

**RNAseq ID:**	PATE2 -
**Survival:**	OS
**Auto select best cutoff:**	checked
**Follow up threshold:**	all
**Censore at threshold:**	checked
**Compute median over entire database:**	false
**Cutoff value used in analysis:**	1
**Expression range of the probe:**	0 - 3
**Invert HR values below 1:**	not checked

Restrictions

Tumor type: Pancreatic ductal adenocarcinoma

Restrict analysis to subtypes...

Stage:	all
Gender:	all
Race:	all
Grade:	all
Mutation burden:	all

Restrict analysis based on cellular content...

Basophils:	all
B-cells:	enriched
CD4+ memory T-cells:	all
CD8+ T-cells:	all
Eosinophils:	all
Macrophages:	all
Mesenchymal stem cells:	all
Natural killer T-cells:	all
Regulatory T-cells:	all
Type 1 T-helper cells:	all
Type 2 T-helper cells:	all

Results

P value: 0.0488
FDR: over 50%

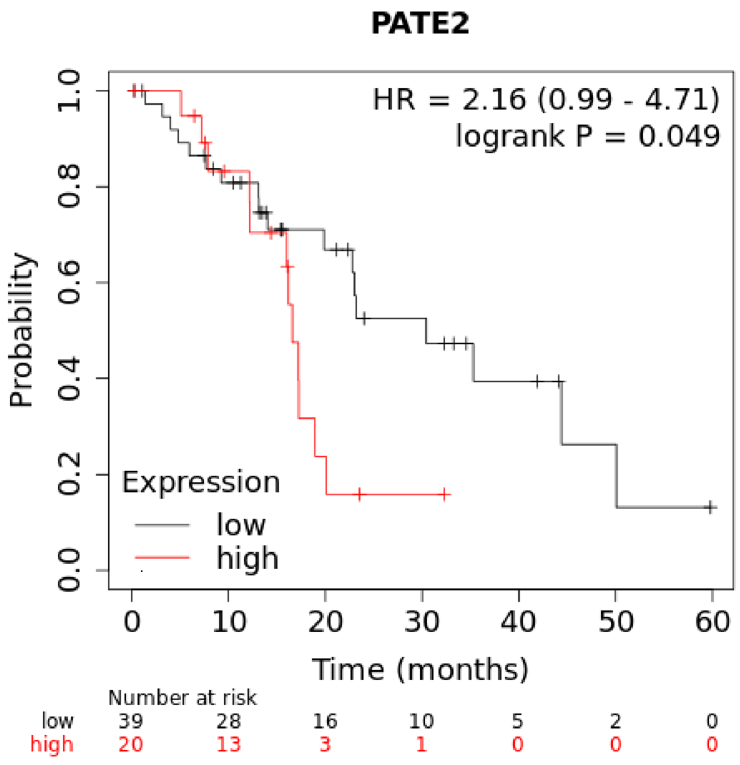

Click here to download the plot in TIFF format

Download plot as a PDF

Download p values vs. cutoff table

Median survival

Low expression cohort (months)	High expression cohort (months)
30.43	16.6

**RNAseq ID:**	PATE3	-
**Survival:**	OS	
**Auto select best cutoff:**	checked	
**Follow up threshold:**	all	
**Censore at threshold:**	checked	
**Compute median over entire database:**	false	
**Cutoff value used in analysis:**	0	
**Expression range of the probe:**	0 - 1	
**Invert HR values below 1:**	not checked	

Restrictions

Tumor type: Pancreatic ductal adenocarcinoma

Restrict analysis to subtypes...

Stage:	all
Gender:	all
Race:	all
Grade:	all
Mutation burden:	all

Restrict analysis based on cellular content...

Basophils:	all
B-cells:	enriched
CD4+ memory T-cells:	all
CD8+ T-cells:	all
Eosinophils:	all
Macrophages:	all
Mesenchymal stem cells:	all
Natural killer T-cells:	all
Regulatory T-cells:	all
Type 1 T-helper cells:	all
Type 2 T-helper cells:	all

Results

**P value:**	0.4032
**FDR:**	100%

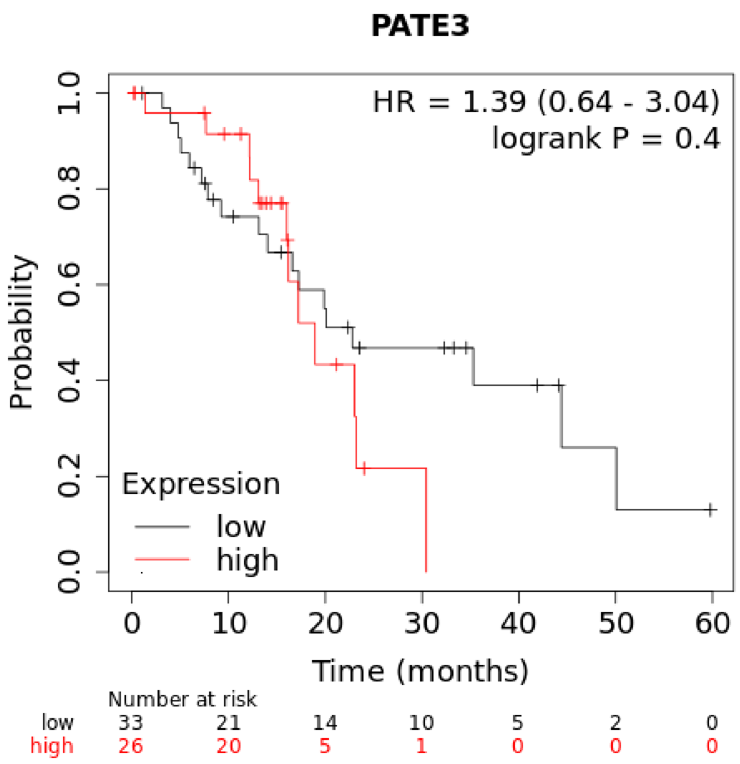

Click here to download the plot in TIFF format

Download plot as a PDF

Download p values vs. cutoff table

Median survival

Low expression cohort (months)	High expression cohort (months)
22.8	18.93

RNAseq ID: PATE4 -
Survival: OS
Auto select best cutoff: checked
Follow up threshold: all
Censore at threshold: checked
Compute median over entire database: false
Cutoff value used in analysis: 1
Expression range of the probe: 0 - 2
Invert HR values below 1: not checked

Restrictions

Tumor type: Pancreatic ductal adenocarcinoma

Restrict analysis to subtypes...

Stage: all
Gender: all
Race: all
Grade: all
Mutation burden: all

Restrict analysis based on cellular content...

Basophils: all

B-cells:	enriched
CD4+ memory T-cells:	all
CD8+ T-cells:	all
Eosinophils:	all
Macrophages:	all
Mesenchymal stem cells:	all
Natural killer T-cells:	all
Regulatory T-cells:	all
Type 1 T-helper cells:	all
Type 2 T-helper cells:	all

Results

P value: 0.2667
FDR: 100%

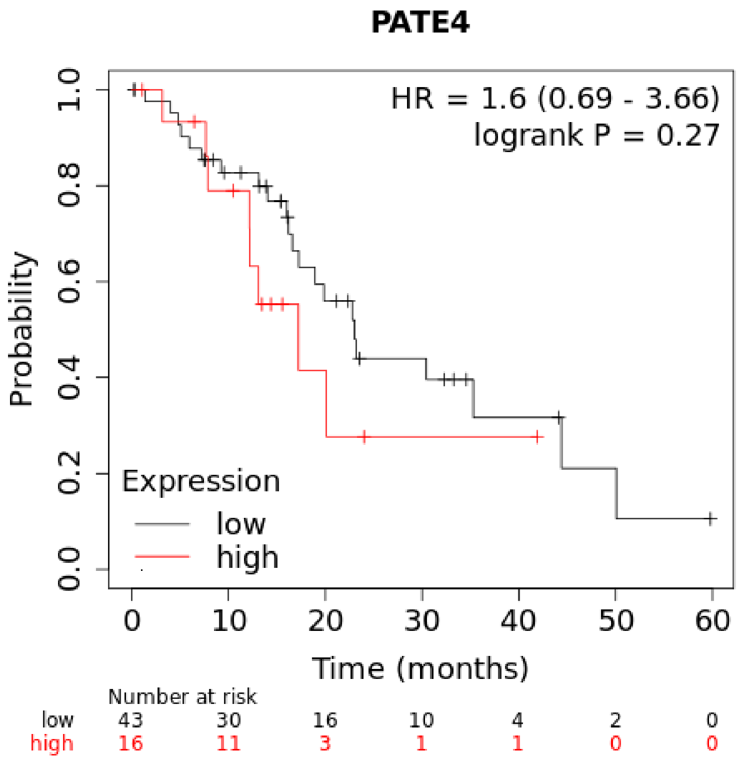

Click here to download the plot in TIFF format

Download plot as a PDF

Download p values vs. cutoff table

Median survival

Low expression cohort (months)	High expression cohort (months)
23.03	17.23

**RNAseq ID:**	CD59 -
**Survival:**	OS
**Auto select best cutoff:**	checked
**Follow up threshold:**	all
**Censore at threshold:**	checked
**Compute median over entire database:**	false
**Cutoff value used in analysis:**	13119
**Expression range of the probe:**	5163 - 27211
**Invert HR values below 1:**	not checked

Restrictions

Tumor type: Pancreatic ductal adenocarcinoma

Restrict analysis to subtypes...

Stage:	all
Gender:	all
Race:	all
Grade:	all
Mutation burden:	all

Restrict analysis based on cellular content...

Basophils:	all
B-cells:	enriched
CD4+ memory T-cells:	all
CD8+ T-cells:	all
Eosinophils:	all
Macrophages:	all
Mesenchymal stem cells:	all
Natural killer T-cells:	all
Regulatory T-cells:	all
Type 1 T-helper cells:	all
Type 2 T-helper cells:	all

Results

P value: 0.1004
FDR: 100%

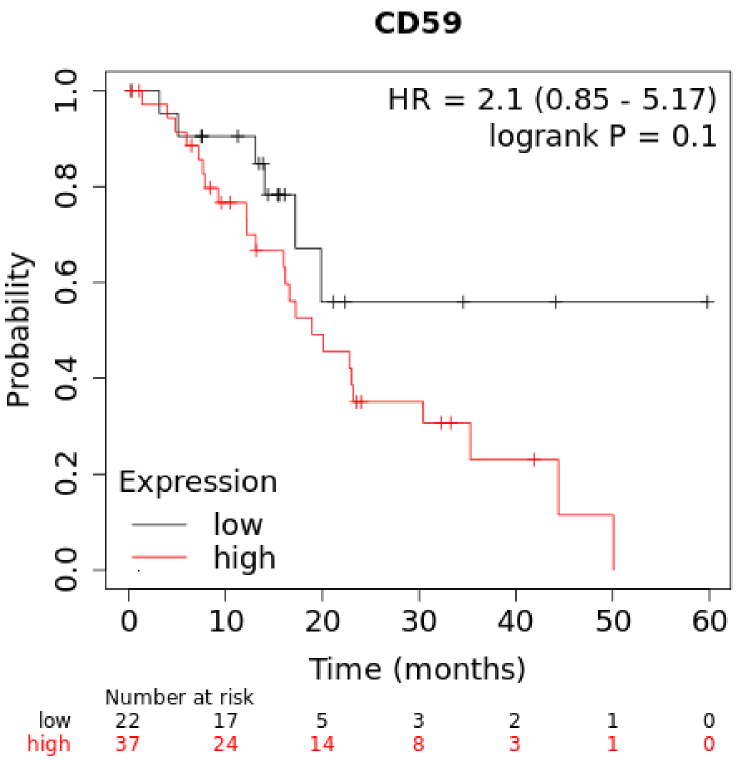

Click here to download the plot in TIFF format

Download plot as a PDF

Download p values vs. cutoff table

Upper quartile survival

Low expression cohort (months)	High expression cohort (months)
17.23	12.17

**RNAseq ID:**	LY6G6C -
**Survival:**	OS
**Auto select best cutoff:**	checked
**Follow up threshold:**	all
**Censore at threshold:**	checked
**Compute median over entire database:**	false
**Cutoff value used in analysis:**	2
**Expression range of the probe:**	0 - 251
**Invert HR values below 1:**	not checked

Restrictions

Tumor type: Pancreatic ductal adenocarcinoma

Restrict analysis to subtypes...

Stage:	all
Gender:	all
Race:	all
Grade:	all
Mutation burden:	all

Restrict analysis based on cellular content...

Basophils:	all
B-cells:	enriched
CD4+ memory T-cells:	all
CD8+ T-cells:	all
Eosinophils:	all
Macrophages:	all
Mesenchymal stem cells:	all
Natural killer T-cells:	all
Regulatory T-cells:	all
Type 1 T-helper cells:	all
Type 2 T-helper cells:	all

Results

**P value:**	0.055
**FDR:**	100%

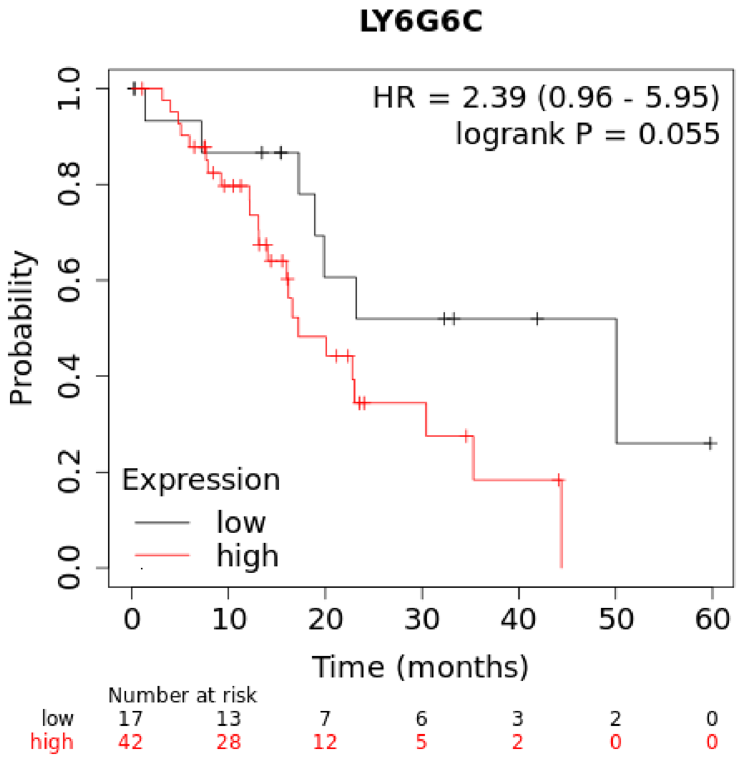

Click here to download the plot in TIFF format

Download plot as a PDF

Download p values vs. cutoff table

Median survival

Low expression cohort (months)	High expression cohort (months)
50.07	17.23

**RNAseq ID:**	LY6G6D -
**Survival:**	OS
**Auto select best cutoff:**	checked
**Follow up threshold:**	all
**Censore at threshold:**	checked
**Compute median over entire database:**	false
**Cutoff value used in analysis:**	0
**Expression range of the probe:**	0 - 2
**Invert HR values below 1:**	not checked

Restrictions

Tumor type: Pancreatic ductal adenocarcinoma

Restrict analysis to subtypes...

Stage:	all
Gender:	all
Race:	all
Grade:	all
Mutation burden:	all

Restrict analysis based on cellular content...

Basophils:	all

B-cells:	enriched
CD4+ memory T-cells:	all
CD8+ T-cells:	all
Eosinophils:	all
Macrophages:	all
Mesenchymal stem cells:	all
Natural killer T-cells:	all
Regulatory T-cells:	all
Type 1 T-helper cells:	all
Type 2 T-helper cells:	all

Results

P value: 0.1892
FDR: 100%

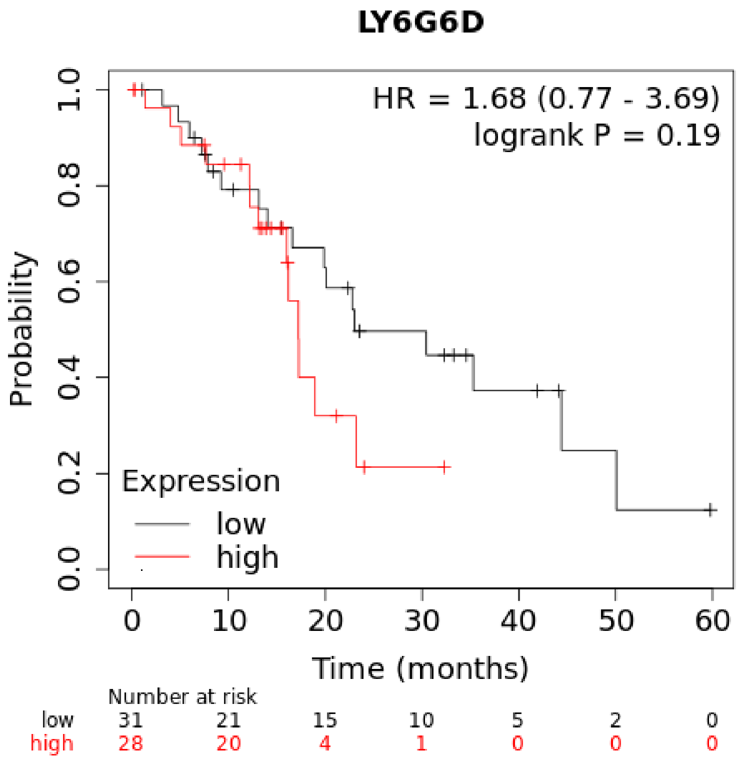

Click here to download the plot in TIFF format

Download plot as a PDF

Download p values vs. cutoff table

Median survival

Low expression cohort (months)	High expression cohort (months)
23.03	17.23

**RNAseq ID:**	LY6G6F -
**Survival:**	OS
**Auto select best cutoff:**	checked
**Follow up threshold:**	all
**Censore at threshold:**	checked
**Compute median over entire database:**	false
**Cutoff value used in analysis:**	0
**Expression range of the probe:**	0 - 5
**Invert HR values below 1:**	not checked

Restrictions

Tumor type: Pancreatic ductal adenocarcinoma

Restrict analysis to subtypes...

Stage:	all
Gender:	all
Race:	all
Grade:	all
Mutation burden:	all

Restrict analysis based on cellular content...

Basophils:	all
B-cells:	enriched
CD4+ memory T-cells:	all
CD8+ T-cells:	all
Eosinophils:	all
Macrophages:	all
Mesenchymal stem cells:	all
Natural killer T-cells:	all
Regulatory T-cells:	all
Type 1 T-helper cells:	all
Type 2 T-helper cells:	all

Results

P value: 0.4419
FDR: 100%

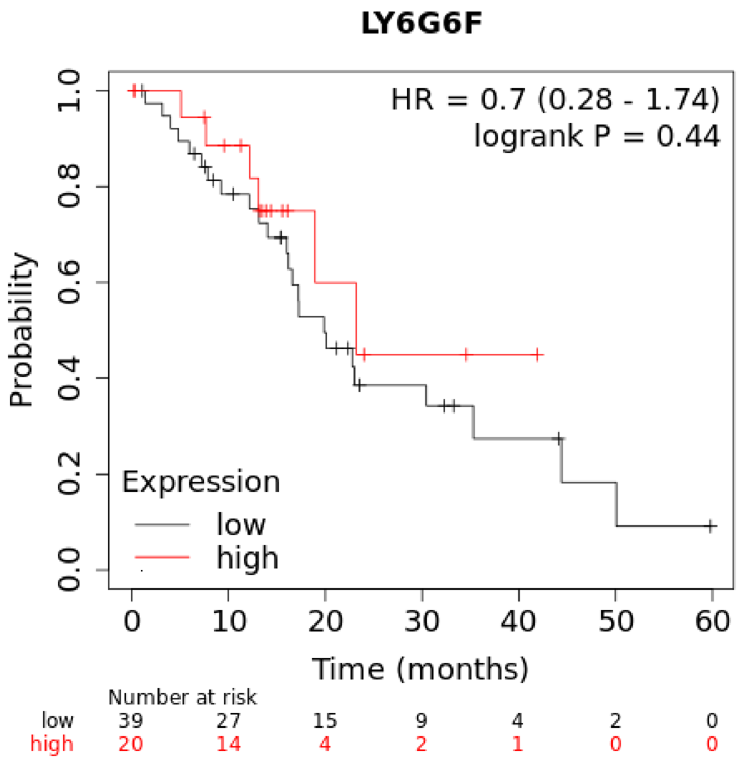

Click here to download the plot in TIFF format

Download plot as a PDF

Download p values vs. cutoff table

Median survival

Low expression cohort (months)	High expression cohort (months)
19.87	23.17

**RNAseq ID:**	LY6G5C -
**Survival:**	OS
**Auto select best cutoff:**	checked
**Follow up threshold:**	all
**Censore at threshold:**	checked
**Compute median over entire database:**	false
**Cutoff value used in analysis:**	68
**Expression range of the probe:**	23 - 93
**Invert HR values below 1:**	not checked

Restrictions

Tumor type: Pancreatic ductal adenocarcinoma

Restrict analysis to subtypes...

Stage:	all
Gender:	all
Race:	all
Grade:	all
Mutation burden:	all

Restrict analysis based on cellular content...

Basophils:	all
B-cells:	enriched
CD4+ memory T-cells:	all
CD8+ T-cells:	all
Eosinophils:	all
Macrophages:	all
Mesenchymal stem cells:	all
Natural killer T-cells:	all
Regulatory T-cells:	all
Type 1 T-helper cells:	all
Type 2 T-helper cells:	all

Results

**P value:**	0.0283
**FDR:**	over 50%

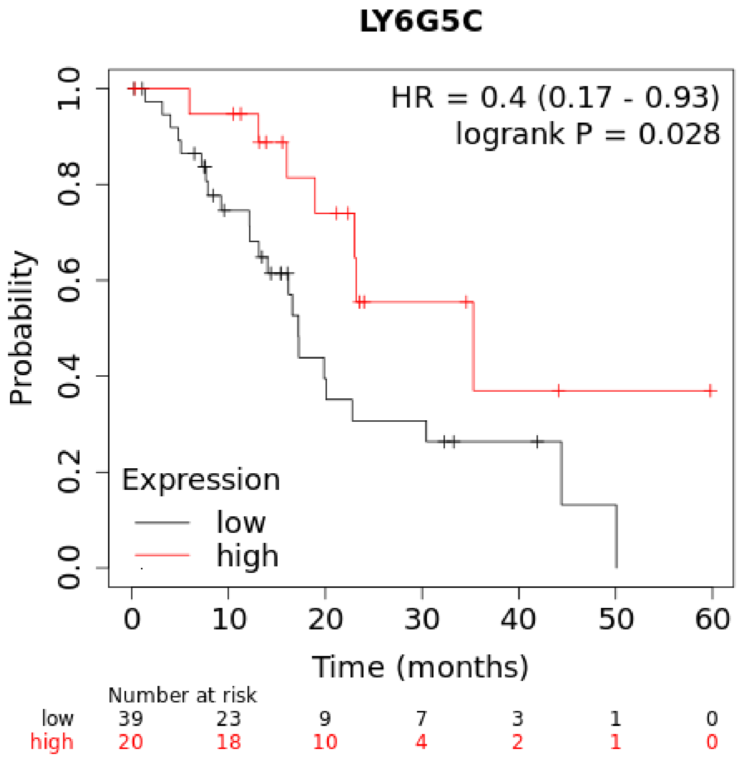

Click here to download the plot in TIFF format

Download plot as a PDF

Download p values vs. cutoff table

Median survival

Low expression cohort (months)	High expression cohort (months)
17.23	35.3

**RNAseq ID:**	LY6G5B -
**Survival:**	OS
**Auto select best cutoff:**	checked
**Follow up threshold:**	all
**Censore at threshold:**	checked
**Compute median over entire database:**	false
**Cutoff value used in analysis:**	37
**Expression range of the probe:**	10 - 173
**Invert HR values below 1:**	not checked

Restrictions

Tumor type: Pancreatic ductal adenocarcinoma

Restrict analysis to subtypes...

Stage:	all
Gender:	all
Race:	all
Grade:	all
Mutation burden:	all

Restrict analysis based on cellular content...

Basophils:	all

B-cells:	enriched
CD4+ memory T-cells:	all
CD8+ T-cells:	all
Eosinophils:	all
Macrophages:	all
Mesenchymal stem cells:	all
Natural killer T-cells:	all
Regulatory T-cells:	all
Type 1 T-helper cells:	all
Type 2 T-helper cells:	all

Results

P value: 0.0213
FDR: over 50%

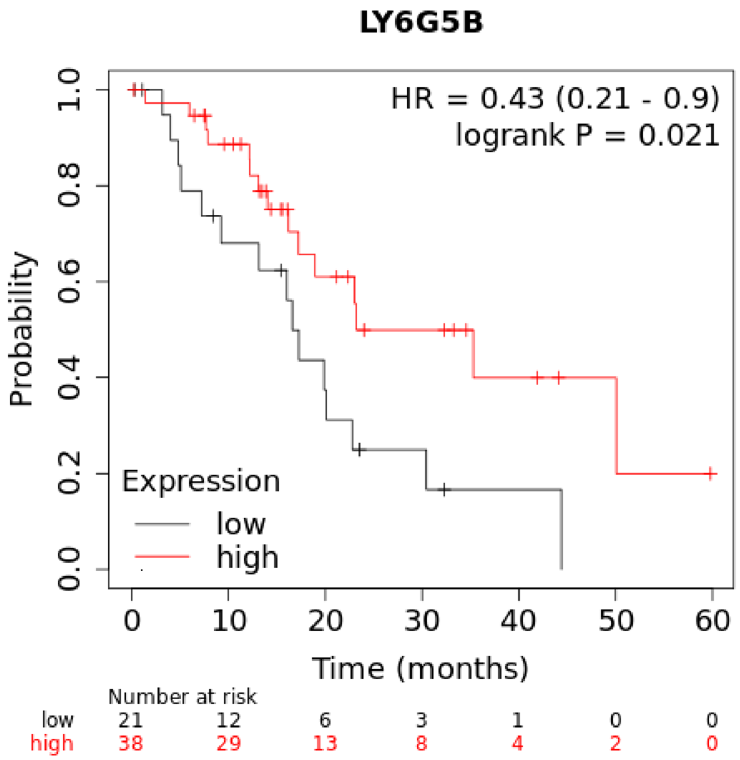

Click here to download the plot in TIFF format

Download plot as a PDF

Download p values vs. cutoff table

Median survival

Low expression cohort (months)	High expression cohort (months)
16.6	23.17

You can save the plots by right-clicking the image and then selecting "Save image as...". To generate a high resolution TIFF image, please adjust the "Settings" in the analysis page.

KMplot.com Copyright © 2009-2020 | Contact | Sitemap

Pan-cancer

KM plotter | Home | Vote | Download | Updates | Contact

The desired RNAseq ID is valid: PSCA (-), LY6K (-), SLURP1 (-), LYPD2 (-), LY6D (-), GML (-), LY6E (-), LY6L (-), LY6H (-), GPIHBP1 (-), LYPD4 (-), CD177 (-), TEX101 (-), LYPD3 (-), PINLYP (-), PLAUR (-), LYPD5 (-), SPACA4 (-), ACRV1 (-), PATE1 (-), PATE2 (-), PATE3 (-), PATE4 (-), CD59 (-), LY6G6C (-), LY6G6D (-), LY6G6F (-), LY6G5C (-), LY6G5B (-),

**RNAseq ID:**	PSCA -
**Survival:**	OS
**Auto select best cutoff:**	checked
**Follow up threshold:**	all
**Censore at threshold:**	checked
**Compute median over entire database:**	false
**Cutoff value used in analysis:**	621
**Expression range of the probe:**	0 - 65661
**Invert HR values below 1:**	not checked

Restrictions

Tumor type: Pancreatic ductal adenocarcinoma

Restrict analysis to subtypes...

Stage:	all
Gender:	all
Race:	all
Grade:	all
Mutation burden:	all

Restrict analysis based on cellular content...

Basophils:	all
B-cells:	decreased
CD4+ memory T-cells:	all
CD8+ T-cells:	all
Eosinophils:	all
Macrophages:	all
Mesenchymal stem cells:	all
Natural killer T-cells:	all
Regulatory T-cells:	all
Type 1 T-helper cells:	all
Type 2 T-helper cells:	all

Results

P value: 0.0005
FDR: 5%

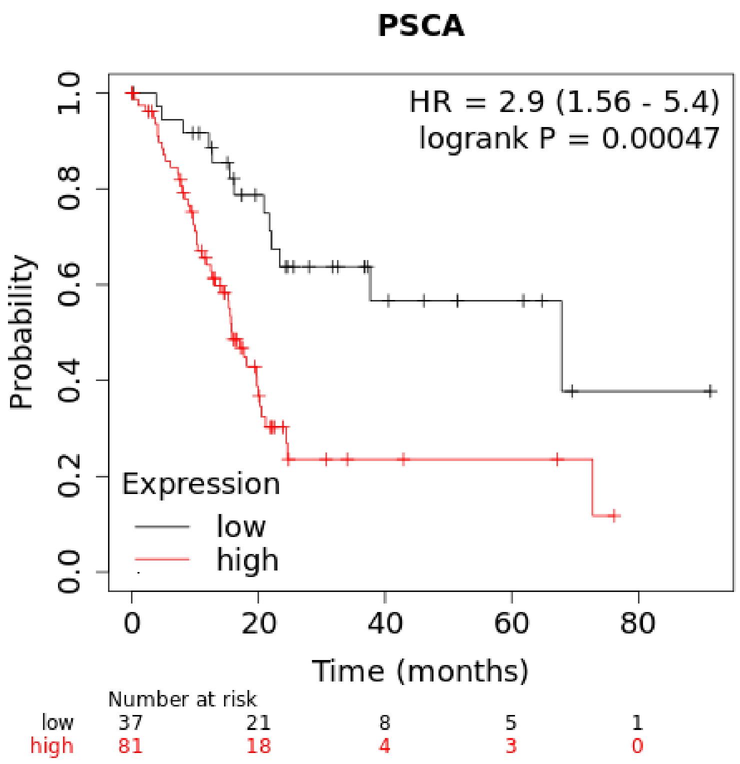

Click here to download the plot in TIFF format

Download plot as a PDF

Download p values vs. cutoff table

Median survival

Low expression cohort (months)	High expression cohort (months)
67.87	15.87

**RNAseq ID:**	LY6K -
**Survival:**	OS
**Auto select best cutoff:**	checked
**Follow up threshold:**	all
**Censore at threshold:**	checked
**Compute median over entire database:**	false
**Cutoff value used in analysis:**	3
**Expression range of the probe:**	0 - 1825
**Invert HR values below 1:**	not checked

Restrictions

Tumor type: Pancreatic ductal adenocarcinoma

Restrict analysis to subtypes...

Stage: all
Gender: all
Race: all
Grade: all
Mutation burden: all

Restrict analysis based on cellular content...

Basophils: all

B-cells:	decreased
CD4+ memory T-cells:	all
CD8+ T-cells:	all
Eosinophils:	all
Macrophages:	all
Mesenchymal stem cells:	all
Natural killer T-cells:	all
Regulatory T-cells:	all
Type 1 T-helper cells:	all
Type 2 T-helper cells:	all

Results

P value: 0.0966
FDR: 100%

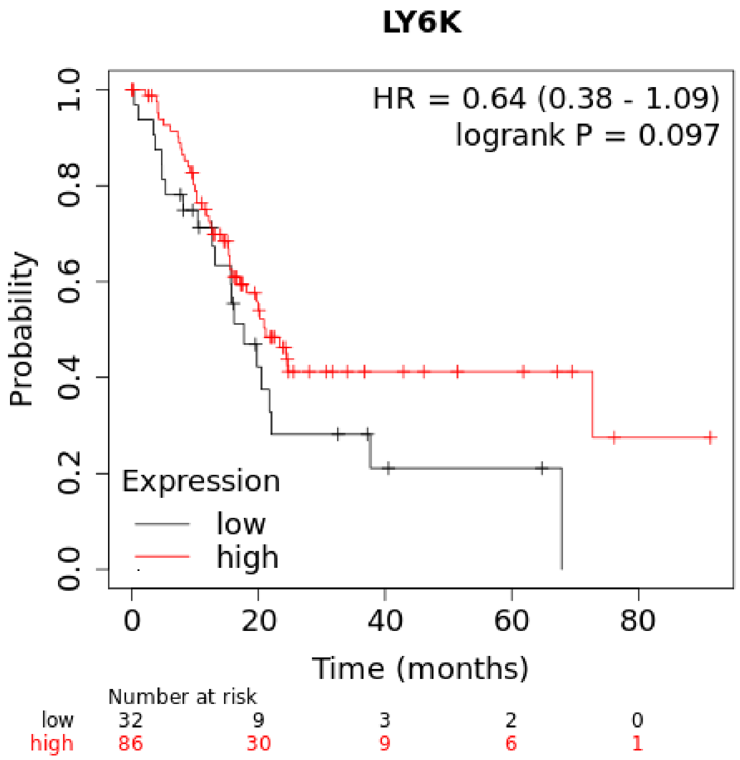

Click here to download the plot in TIFF format

Download plot as a PDF

Download p values vs. cutoff table

Median survival

Low expression cohort (months)	High expression cohort (months)
17.73	21.13

**RNAseq ID:**	SLURP1 -
**Survival:**	OS
**Auto select best cutoff:**	checked
**Follow up threshold:**	all
**Censore at threshold:**	checked
**Compute median over entire database:**	false
**Cutoff value used in analysis:**	1
**Expression range of the probe:**	0 - 185
**Invert HR values below 1:**	not checked

Restrictions

Tumor type: Pancreatic ductal adenocarcinoma

Restrict analysis to subtypes...

Stage:	all
Gender:	all
Race:	all
Grade:	all
Mutation burden:	all

Restrict analysis based on cellular content...

Basophils:	all
B-cells:	decreased
CD4+ memory T-cells:	all
CD8+ T-cells:	all
Eosinophils:	all
Macrophages:	all
Mesenchymal stem cells:	all
Natural killer T-cells:	all
Regulatory T-cells:	all
Type 1 T-helper cells:	all
Type 2 T-helper cells:	all

Results

P value: 9.2e-6
FDR: 1%

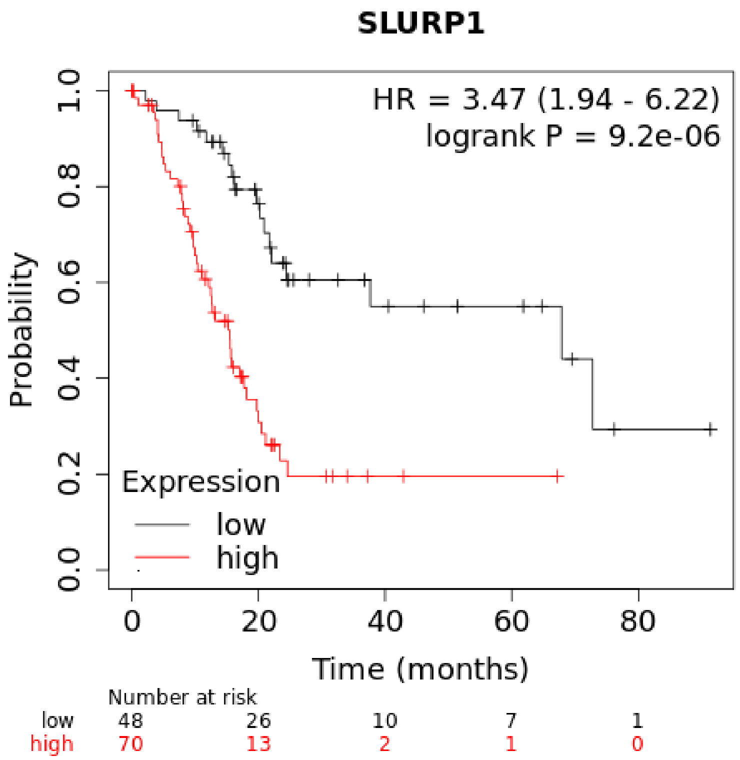

Click here to download the plot in TIFF format

Download plot as a PDF

Download p values vs. cutoff table

Median survival

Low expression cohort (months)	High expression cohort (months)
67.87	15.53

**RNAseq ID:**	LYPD2 -
**Survival:**	OS
**Auto select best cutoff:**	checked
**Follow up threshold:**	all
**Censore at threshold:**	checked
**Compute median over entire database:**	false
**Cutoff value used in analysis:**	4
**Expression range of the probe:**	0 - 4748
**Invert HR values below 1:**	not checked

Restrictions

Tumor type: Pancreatic ductal adenocarcinoma

Restrict analysis to subtypes...

Stage:	all
Gender:	all
Race:	all
Grade:	all
Mutation burden:	all

Restrict analysis based on cellular content...

Basophils:	all
B-cells:	decreased
CD4+ memory T-cells:	all
CD8+ T-cells:	all
Eosinophils:	all
Macrophages:	all
Mesenchymal stem cells:	all
Natural killer T-cells:	all
Regulatory T-cells:	all
Type 1 T-helper cells:	all
Type 2 T-helper cells:	all

Results

**P value:**	0.0021
**FDR:**	50%

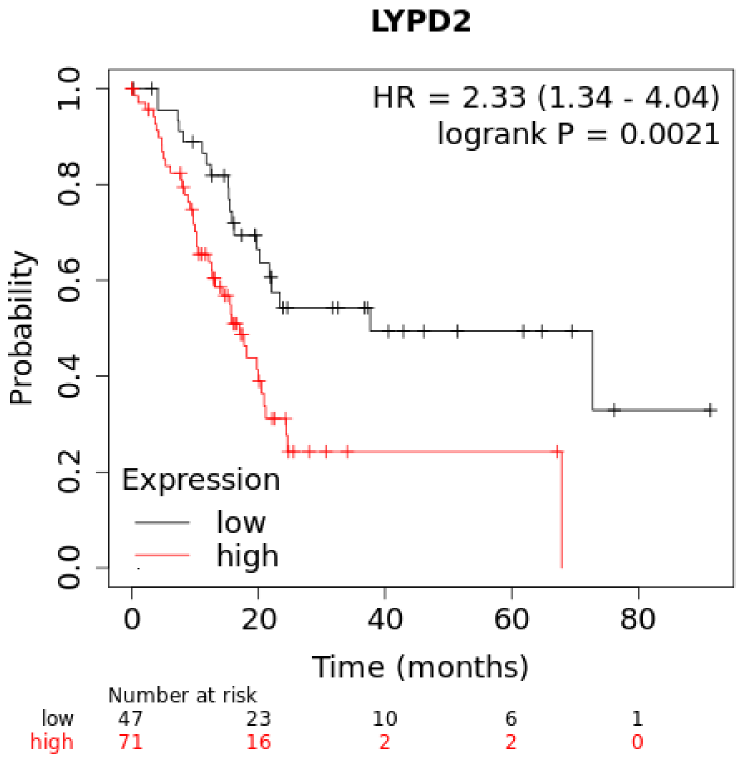

Click here to download the plot in TIFF format

Download plot as a PDF

Download p values vs. cutoff table

Median survival

Low expression cohort (months)	High expression cohort (months)
37.67	17.03

**RNAseq ID:**	LY6D -
**Survival:**	OS
**Auto select best cutoff:**	checked
**Follow up threshold:**	all
**Censore at threshold:**	checked
**Compute median over entire database:**	false
**Cutoff value used in analysis:**	181
**Expression range of the probe:**	0 - 16422
**Invert HR values below 1:**	not checked

Restrictions

Tumor type: Pancreatic ductal adenocarcinoma

Restrict analysis to subtypes...

Stage:	all
Gender:	all
Race:	all
Grade:	all
Mutation burden:	all

Restrict analysis based on cellular content...

Basophils:	all

B-cells:	decreased
CD4+ memory T-cells:	all
CD8+ T-cells:	all
Eosinophils:	all
Macrophages:	all
Mesenchymal stem cells:	all
Natural killer T-cells:	all
Regulatory T-cells:	all
Type 1 T-helper cells:	all
Type 2 T-helper cells:	all

Results

P value: 0.0009
FDR: 10%

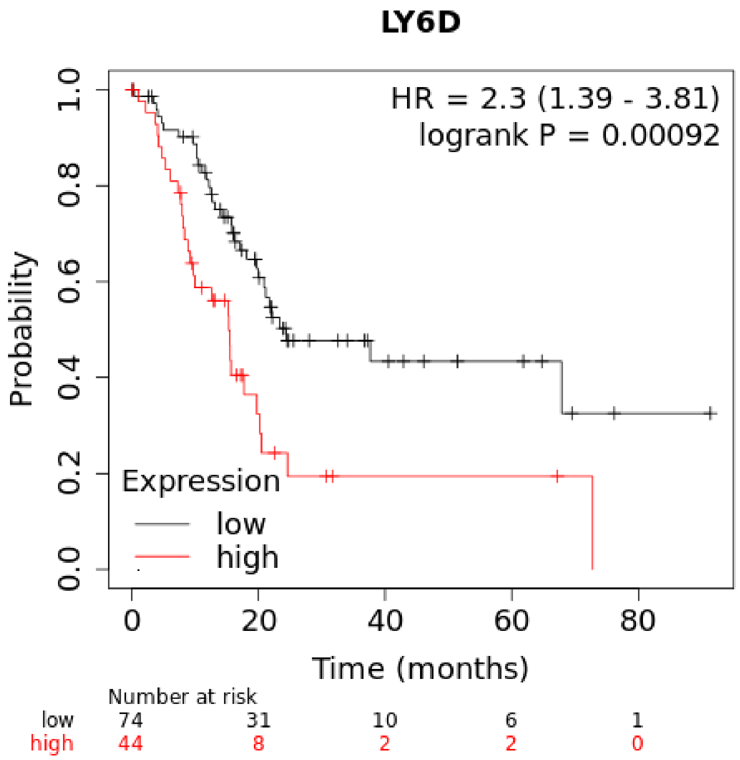

Click here to download the plot in TIFF format

Download plot as a PDF

Download p values vs. cutoff table

Median survival

Low expression cohort (months)	High expression cohort (months)
24.4	15.33

**RNAseq ID:**	GML -
**Survival:**	OS
**Auto select best cutoff:**	checked
**Follow up threshold:**	all
**Censore at threshold:**	checked
**Compute median over entire database:**	false
**Cutoff value used in analysis:**	0
**Expression range of the probe:**	0 - 2
**Invert HR values below 1:**	not checked

Restrictions

Tumor type: Pancreatic ductal adenocarcinoma

Restrict analysis to subtypes...

Stage:	all
Gender:	all
Race:	all
Grade:	all
Mutation burden:	all

Restrict analysis based on cellular content...

Basophils:	all
B-cells:	decreased
CD4+ memory T-cells:	all
CD8+ T-cells:	all
Eosinophils:	all
Macrophages:	all
Mesenchymal stem cells:	all
Natural killer T-cells:	all
Regulatory T-cells:	all
Type 1 T-helper cells:	all
Type 2 T-helper cells:	all

Results

P value: 0.0385
FDR: over 50%

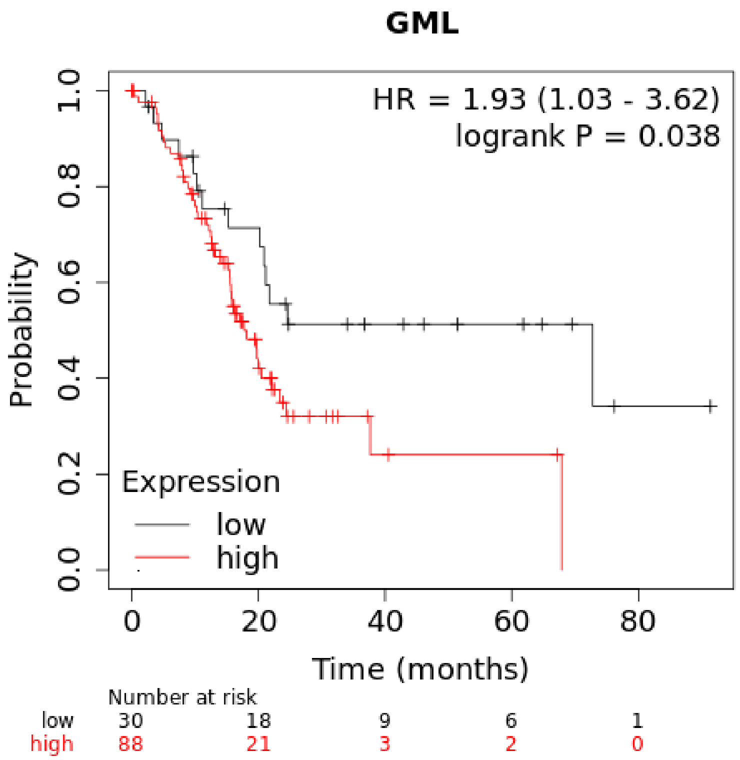

Click here to download the plot in TIFF format

Download plot as a PDF

Download p values vs. cutoff table

Median survival

Low expression cohort (months)	High expression cohort (months)
72.73	17.73

**RNAseq ID:**	LY6E	-
**Survival:**	OS	
**Auto select best cutoff:**	checked	
**Follow up threshold:**	all	
**Censore at threshold:**	checked	
**Compute median over entire database:**	false	
**Cutoff value used in analysis:**	10634	
**Expression range of the probe:**	504 - 52613	
**Invert HR values below 1:**	not checked	

Restrictions

Tumor type: Pancreatic ductal adenocarcinoma

Restrict analysis to subtypes...

Stage:	all
Gender:	all
Race:	all
Grade:	all
Mutation burden:	all

Restrict analysis based on cellular content...

Basophils:	all
B-cells:	decreased
CD4+ memory T-cells:	all
CD8+ T-cells:	all
Eosinophils:	all
Macrophages:	all
Mesenchymal stem cells:	all
Natural killer T-cells:	all
Regulatory T-cells:	all
Type 1 T-helper cells:	all
Type 2 T-helper cells:	all

Results

**P value:**	0.0078
**FDR:**	over 50%

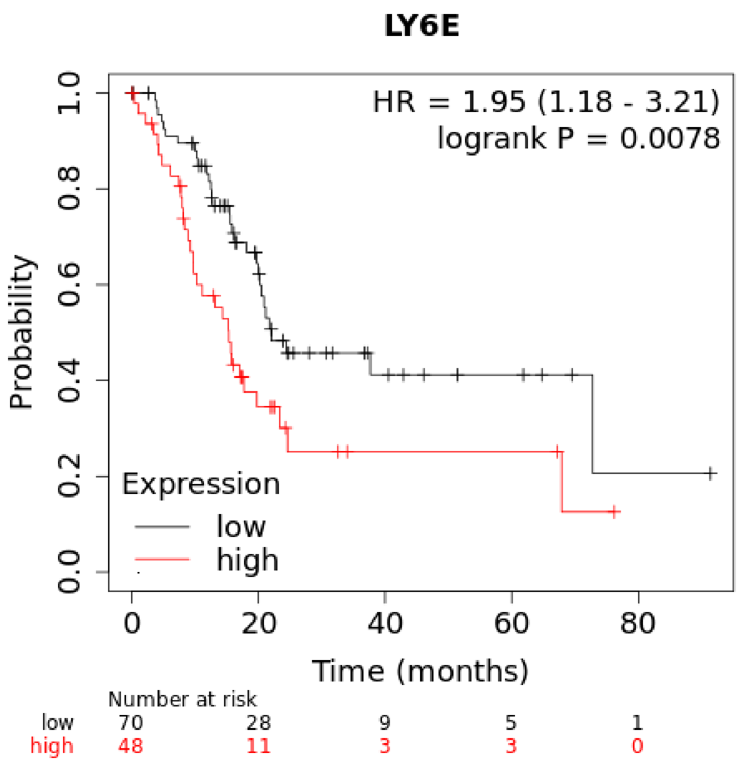

Click here to download the plot in TIFF format

Download plot as a PDF

Download p values vs. cutoff table

Median survival

Low expression cohort (months)	High expression cohort (months)
22.03	15.33

**RNAseq ID:**	LY6L -
**Survival:**	OS
**Auto select best cutoff:**	checked
**Follow up threshold:**	all
**Censore at threshold:**	checked
**Compute median over entire database:**	false
**Cutoff value used in analysis:**	0
**Expression range of the probe:**	0 - 8
**Invert HR values below 1:**	not checked

Restrictions

Tumor type: Pancreatic ductal adenocarcinoma

Restrict analysis to subtypes...

Stage:	all
Gender:	all
Race:	all
Grade:	all
Mutation burden:	all

Restrict analysis based on cellular content...

Basophils: all

B-cells:	decreased
CD4+ memory T-cells:	all
CD8+ T-cells:	all
Eosinophils:	all
Macrophages:	all
Mesenchymal stem cells:	all
Natural killer T-cells:	all
Regulatory T-cells:	all
Type 1 T-helper cells:	all
Type 2 T-helper cells:	all

Results

P value: 0.043
FDR: over 50%

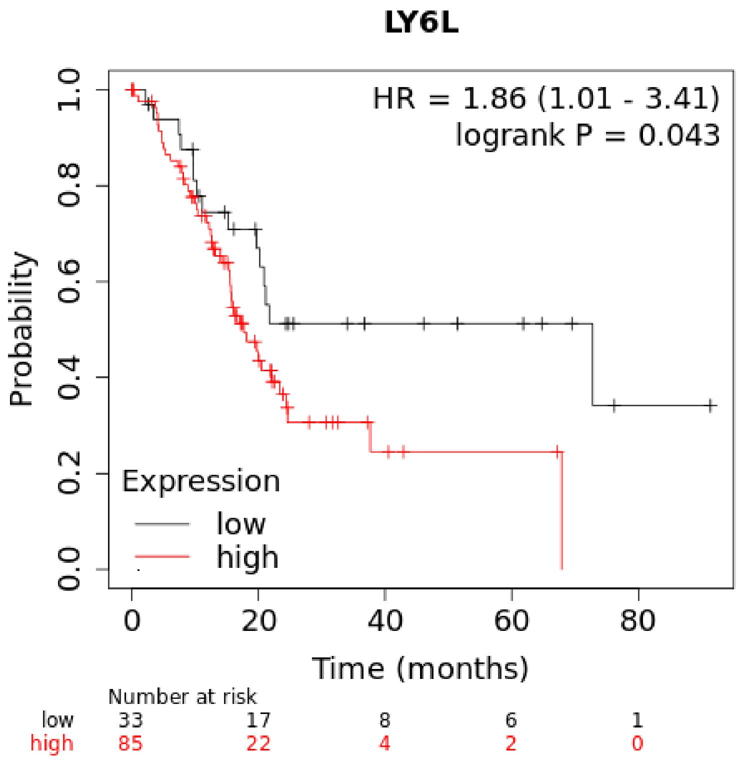

Click here to download the plot in TIFF format

Download plot as a PDF

Download p values vs. cutoff table

Median survival

Low expression cohort (months)	High expression cohort (months)
72.73	17.73

**RNAseq ID:**	LY6H -
**Survival:**	OS
**Auto select best cutoff:**	checked
**Follow up threshold:**	all
**Censore at threshold:**	checked
**Compute median over entire database:**	false
**Cutoff value used in analysis:**	39
**Expression range of the probe:**	1 - 9495
**Invert HR values below 1:**	not checked

Restrictions

Tumor type: Pancreatic ductal adenocarcinoma

Restrict analysis to subtypes...

Stage:	all
Gender:	all
Race:	all
Grade:	all
Mutation burden:	all

Restrict analysis based on cellular content...

Basophils:	all
B-cells:	decreased
CD4+ memory T-cells:	all
CD8+ T-cells:	all
Eosinophils:	all
Macrophages:	all
Mesenchymal stem cells:	all
Natural killer T-cells:	all
Regulatory T-cells:	all
Type 1 T-helper cells:	all
Type 2 T-helper cells:	all

Results

P value: 0.0007
FDR: 20%

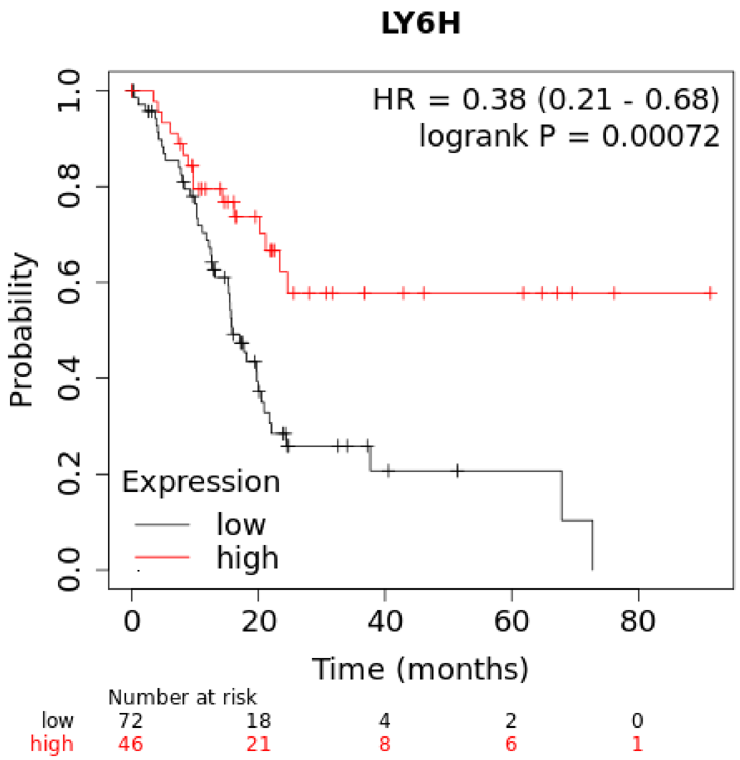

Click here to download the plot in TIFF format

Download plot as a PDF

Download p values vs. cutoff table

Upper quartile survival

Low expression cohort (months)	High expression cohort (months)
10.27	16.17

**RNAseq ID:**	GPIHBP1 -
**Survival:**	OS
**Auto select best cutoff:**	checked
**Follow up threshold:**	all
**Censore at threshold:**	checked
**Compute median over entire database:**	false
**Cutoff value used in analysis:**	23
**Expression range of the probe:**	5 - 344
**Invert HR values below 1:**	not checked

Restrictions

Tumor type: Pancreatic ductal adenocarcinoma

Restrict analysis to subtypes...

Stage:	all
Gender:	all
Race:	all
Grade:	all
Mutation burden:	all

Restrict analysis based on cellular content...

Basophils:	all
B-cells:	decreased
CD4+ memory T-cells:	all
CD8+ T-cells:	all
Eosinophils:	all
Macrophages:	all
Mesenchymal stem cells:	all
Natural killer T-cells:	all
Regulatory T-cells:	all
Type 1 T-helper cells:	all
Type 2 T-helper cells:	all

Results

**P value:**	0.1802
**FDR:**	100%

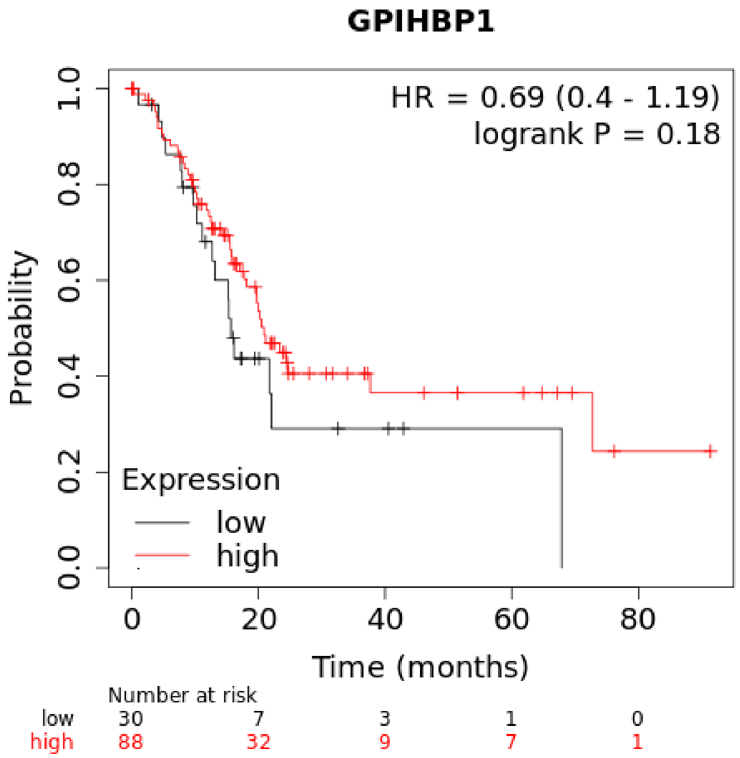

Click here to download the plot in TIFF format

Download plot as a PDF

Download p values vs. cutoff table

Median survival

Low expression cohort (months)	High expression cohort (months)
15.67	20.9

**RNAseq ID:**	LYPD4 -
**Survival:**	OS
**Auto select best cutoff:**	checked
**Follow up threshold:**	all
**Censore at threshold:**	checked
**Compute median over entire database:**	false
**Cutoff value used in analysis:**	0
**Expression range of the probe:**	0 - 18
**Invert HR values below 1:**	not checked

Restrictions

Tumor type: Pancreatic ductal adenocarcinoma

Restrict analysis to subtypes...

Stage:	all
Gender:	all
Race:	all
Grade:	all
Mutation burden:	all

Restrict analysis based on cellular content...

Basophils:	all

B-cells:	decreased
CD4+ memory T-cells:	all
CD8+ T-cells:	all
Eosinophils:	all
Macrophages:	all
Mesenchymal stem cells:	all
Natural killer T-cells:	all
Regulatory T-cells:	all
Type 1 T-helper cells:	all
Type 2 T-helper cells:	all

Results

P value: 0.009
FDR: over 50%

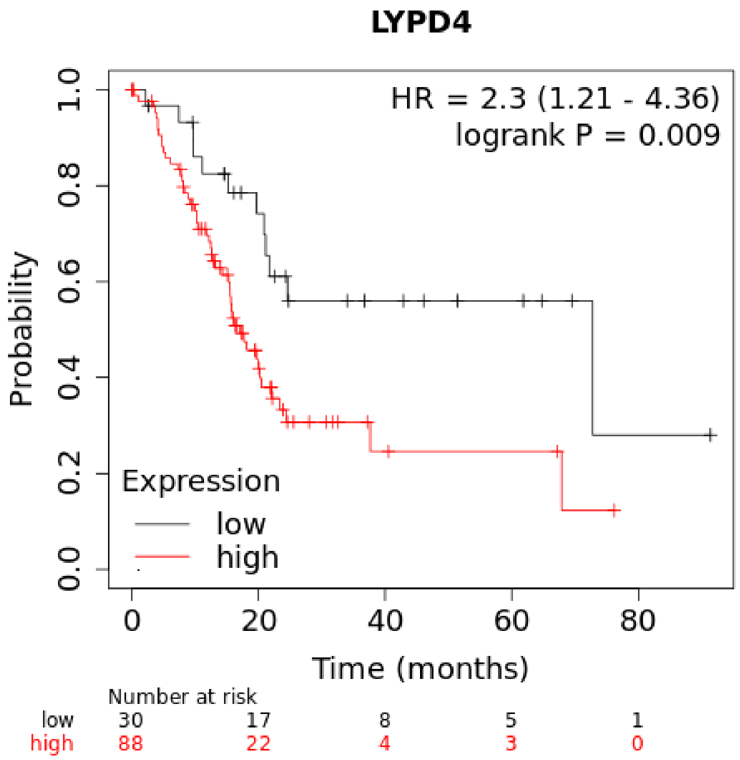

Click here to download the plot in TIFF format

Download plot as a PDF

Download p values vs. cutoff table

Median survival

Low expression cohort (months)	High expression cohort (months)
72.73	17.03

**RNAseq ID:**	CD177 -
**Survival:**	OS
**Auto select best cutoff:**	checked
**Follow up threshold:**	all
**Censore at threshold:**	checked
**Compute median over entire database:**	false
**Cutoff value used in analysis:**	11
**Expression range of the probe:**	0 - 3766
**Invert HR values below 1:**	not checked

Restrictions

Tumor type: Pancreatic ductal adenocarcinoma

Restrict analysis to subtypes...

Stage:	all
Gender:	all
Race:	all
Grade:	all
Mutation burden:	all

Restrict analysis based on cellular content...

Basophils:	all
B-cells:	decreased
CD4+ memory T-cells:	all
CD8+ T-cells:	all
Eosinophils:	all
Macrophages:	all
Mesenchymal stem cells:	all
Natural killer T-cells:	all
Regulatory T-cells:	all
Type 1 T-helper cells:	all
Type 2 T-helper cells:	all

Results

P value: 0.1185
FDR: 100%

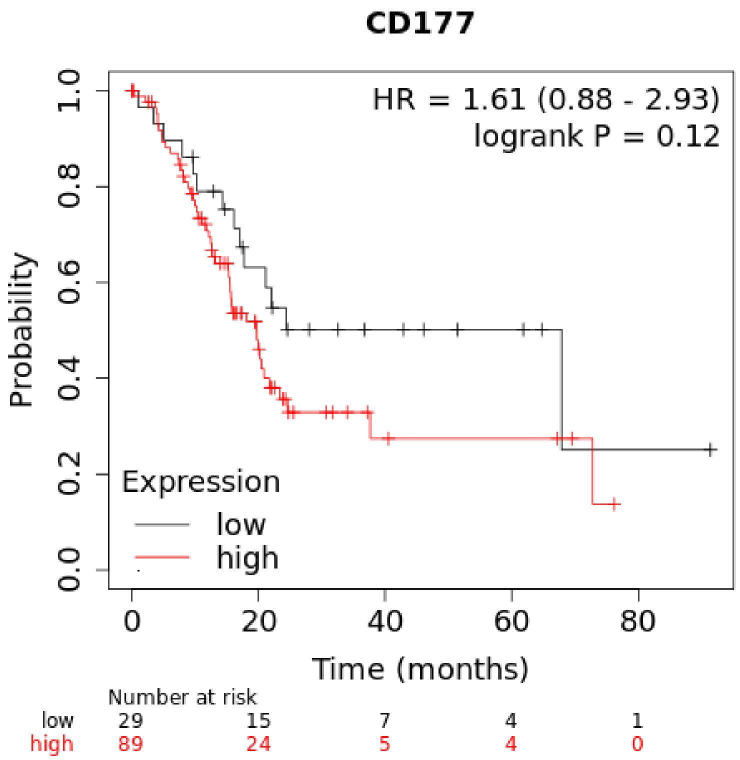

Click here to download the plot in TIFF format

Download plot as a PDF

Download p values vs. cutoff table

Median survival

Low expression cohort (months)	High expression cohort (months)
67.87	19.73

**RNAseq ID:**	TEX101 -
**Survival:**	OS
**Auto select best cutoff:**	checked
**Follow up threshold:**	all
**Censore at threshold:**	checked
**Compute median over entire database:**	false
**Cutoff value used in analysis:**	0
**Expression range of the probe:**	0 - 24
**Invert HR values below 1:**	not checked

Restrictions

Tumor type: Pancreatic ductal adenocarcinoma

Restrict analysis to subtypes...

Stage:	all
Gender:	all
Race:	all
Grade:	all
Mutation burden:	all

Restrict analysis based on cellular content...

Basophils:	all
B-cells:	decreased
CD4+ memory T-cells:	all
CD8+ T-cells:	all
Eosinophils:	all
Macrophages:	all
Mesenchymal stem cells:	all
Natural killer T-cells:	all
Regulatory T-cells:	all
Type 1 T-helper cells:	all
Type 2 T-helper cells:	all

Results

**P value:**	0.1132
**FDR:**	100%

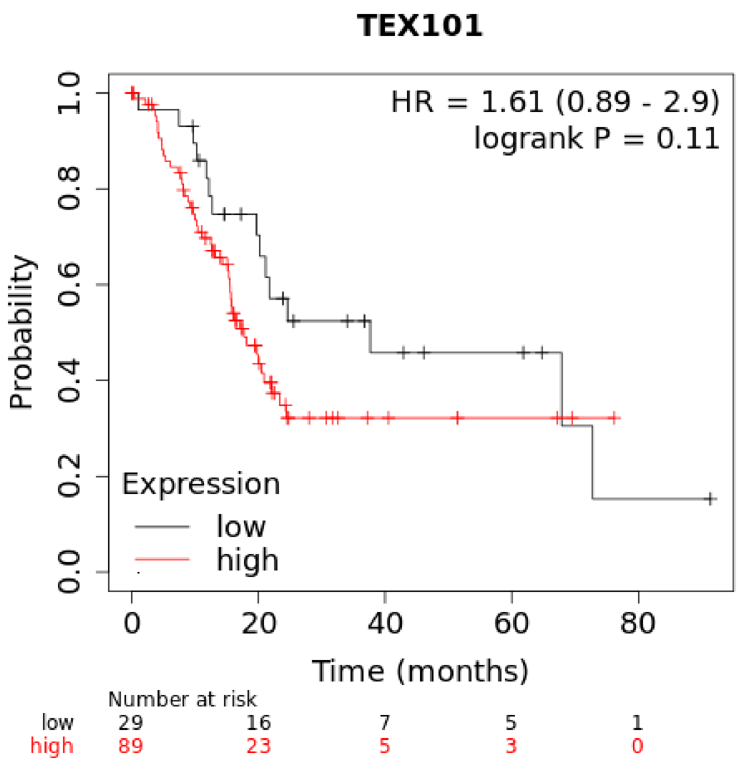

Click here to download the plot in TIFF format

Download plot as a PDF

Download p values vs. cutoff table

Median survival

Low expression cohort (months)	High expression cohort (months)
37.67	17.73

**RNAseq ID:**	LYPD3 -
**Survival:**	OS
**Auto select best cutoff:**	checked
**Follow up threshold:**	all
**Censore at threshold:**	checked
**Compute median over entire database:**	false
**Cutoff value used in analysis:**	279
**Expression range of the probe:**	9 - 7684
**Invert HR values below 1:**	not checked

Restrictions

Tumor type: Pancreatic ductal adenocarcinoma

Restrict analysis to subtypes...

Stage:	all
Gender:	all
Race:	all
Grade:	all
Mutation burden:	all

Restrict analysis based on cellular content...

Basophils:	all

B-cells: decreased
CD4+ memory T-cells: all
CD8+ T-cells: all
Eosinophils: all
Macrophages: all
Mesenchymal stem cells: all
Natural killer T-cells: all
Regulatory T-cells: all
Type 1 T-helper cells: all
Type 2 T-helper cells: all

Results

P value: 0.2181
FDR: 100%

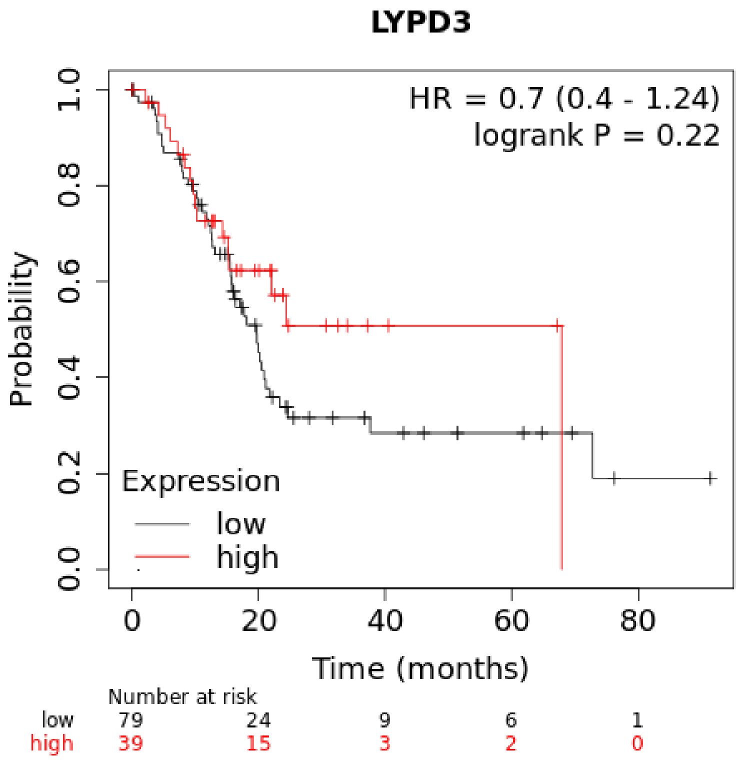

Click here to download the plot in TIFF format

Download plot as a PDF

Download p values vs. cutoff table

Median survival

Low expression cohort (months)	High expression cohort (months)
19.73	67.87

RNAseq ID: PINLYP -
Survival: OS
Auto select best cutoff: checked
Follow up threshold: all
Censore at threshold: checked
Compute median over entire database: false
Cutoff value used in analysis: 71
Expression range of the probe: 5 - 387
Invert HR values below 1: not checked

Restrictions

Tumor type: Pancreatic ductal adenocarcinoma

Restrict analysis to subtypes...

Stage: all
Gender: all
Race: all
Grade: all
Mutation burden: all

Restrict analysis based on cellular content...

Basophils: all
B-cells: decreased
CD4+ memory T-cells: all
CD8+ T-cells: all
Eosinophils: all
Macrophages: all
Mesenchymal stem cells: all
Natural killer T-cells: all
Regulatory T-cells: all
Type 1 T-helper cells: all
Type 2 T-helper cells: all

Results

P value: 0.0939
FDR: 100%

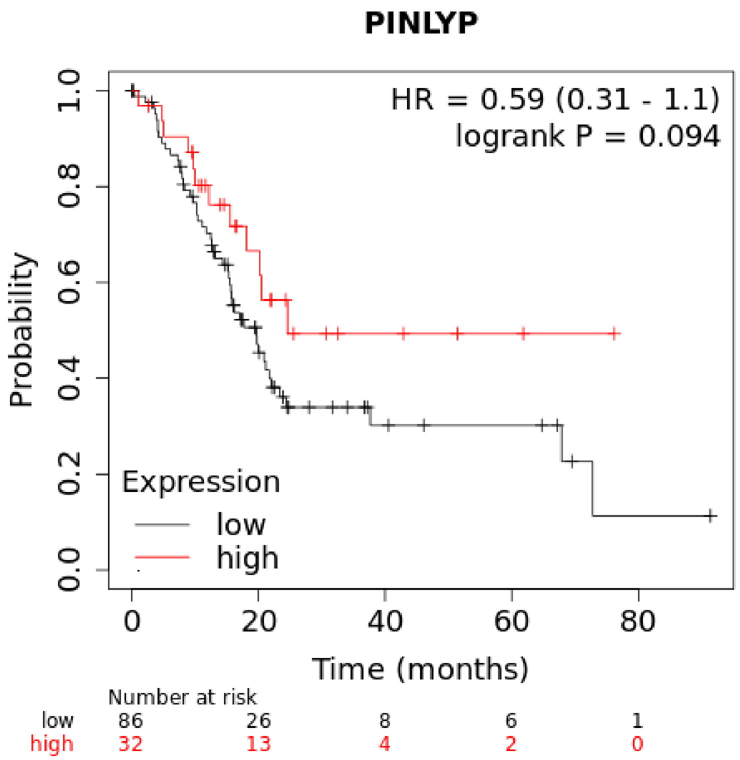

Click here to download the plot in TIFF format

Download plot as a PDF

Download p values vs. cutoff table

Median survival

Low expression cohort (months)	High expression cohort (months)
19.73	24.6

**RNAseq ID:**	PLAUR -
**Survival:**	OS
**Auto select best cutoff:**	checked
**Follow up threshold:**	all
**Censore at threshold:**	checked
**Compute median over entire database:**	false
**Cutoff value used in analysis:**	1861
**Expression range of the probe:**	55 - 18314
**Invert HR values below 1:**	not checked

Restrictions

Tumor type: Pancreatic ductal adenocarcinoma

Restrict analysis to subtypes...

Stage:	all
Gender:	all
Race:	all
Grade:	all
Mutation burden:	all

Restrict analysis based on cellular content...

Basophils:	all
B-cells:	decreased
CD4+ memory T-cells:	all
CD8+ T-cells:	all
Eosinophils:	all
Macrophages:	all
Mesenchymal stem cells:	all
Natural killer T-cells:	all
Regulatory T-cells:	all
Type 1 T-helper cells:	all
Type 2 T-helper cells:	all

Results

**P value:**	0.0644
**FDR:**	100%

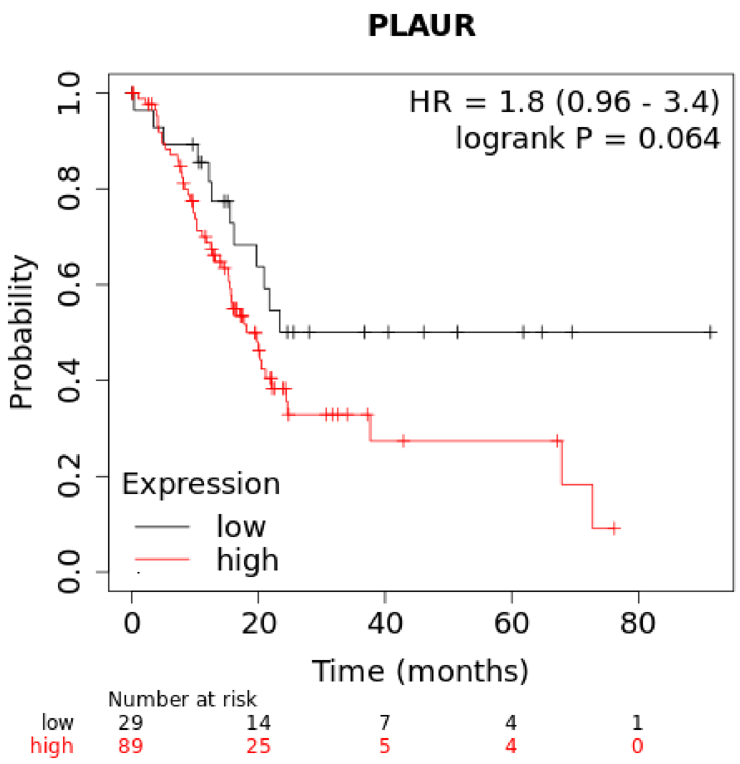

Click here to download the plot in TIFF format

Download plot as a PDF

Download p values vs. cutoff table

Upper quartile survival

Low expression cohort (months)	High expression cohort (months)
15.57	9.97

**RNAseq ID:**	LYPD5 -
**Survival:**	OS
**Auto select best cutoff:**	checked
**Follow up threshold:**	all
**Censore at threshold:**	checked
**Compute median over entire database:**	false
**Cutoff value used in analysis:**	106
**Expression range of the probe:**	5 - 293
**Invert HR values below 1:**	not checked

Restrictions

Tumor type: Pancreatic ductal adenocarcinoma

Restrict analysis to subtypes...

Stage:	all
Gender:	all
Race:	all
Grade:	all
Mutation burden:	all

Restrict analysis based on cellular content...

Basophils:	all

B-cells:	decreased
CD4+ memory T-cells:	all
CD8+ T-cells:	all
Eosinophils:	all
Macrophages:	all
Mesenchymal stem cells:	all
Natural killer T-cells:	all
Regulatory T-cells:	all
Type 1 T-helper cells:	all
Type 2 T-helper cells:	all

Results

P value: 0.003
FDR: 50%

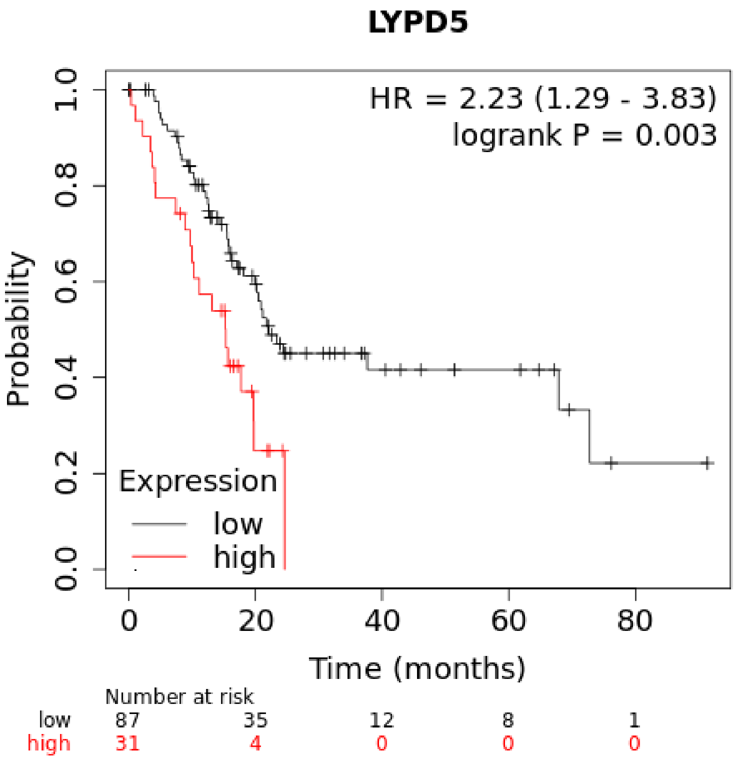

Click here to download the plot in TIFF format

Download plot as a PDF

Download p values vs. cutoff table

Median survival

Low expression cohort (months)	High expression cohort (months)
22.03	15.33

**RNAseq ID:**	SPACA4 -
**Survival:**	OS
**Auto select best cutoff:**	checked
**Follow up threshold:**	all
**Censore at threshold:**	checked
**Compute median over entire database:**	false
**Cutoff value used in analysis:**	17
**Expression range of the probe:**	0 - 206
**Invert HR values below 1:**	not checked

Restrictions

Tumor type: Pancreatic ductal adenocarcinoma

Restrict analysis to subtypes...

Stage: all
Gender: all
Race: all
Grade: all
Mutation burden: all

Restrict analysis based on cellular content...

Basophils: all
B-cells: decreased
CD4+ memory T-cells: all
CD8+ T-cells: all
Eosinophils: all
Macrophages: all
Mesenchymal stem cells: all
Natural killer T-cells: all
Regulatory T-cells: all
Type 1 T-helper cells: all
Type 2 T-helper cells: all

Results

P value: 0.2212
FDR: 100%

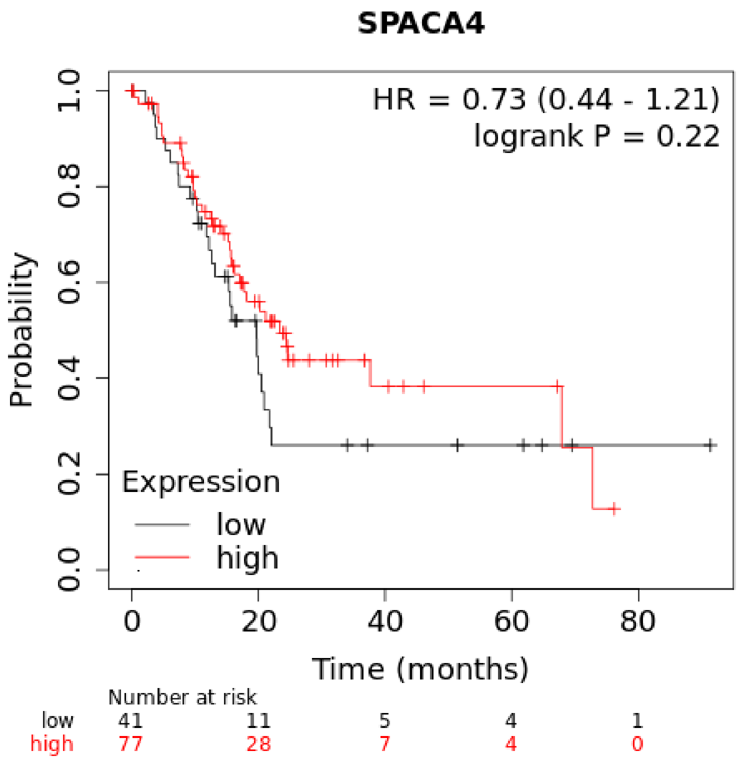

Click here to download the plot in TIFF format

Download plot as a PDF

Download p values vs. cutoff table

Median survival

Low expression cohort (months)	High expression cohort (months)
19.73	23.4

**RNAseq ID:**	ACRV1 -
**Survival:**	OS
**Auto select best cutoff:**	checked
**Follow up threshold:**	all
**Censore at threshold:**	checked
**Compute median over entire database:**	false
**Cutoff value used in analysis:**	4
**Expression range of the probe:**	0 - 71
**Invert HR values below 1:**	not checked

Restrictions

Tumor type: Pancreatic ductal adenocarcinoma

Restrict analysis to subtypes...

Stage:	all
Gender:	all
Race:	all
Grade:	all
Mutation burden:	all

Restrict analysis based on cellular content...

Basophils:	all
B-cells:	decreased
CD4+ memory T-cells:	all
CD8+ T-cells:	all
Eosinophils:	all
Macrophages:	all
Mesenchymal stem cells:	all
Natural killer T-cells:	all
Regulatory T-cells:	all
Type 1 T-helper cells:	all
Type 2 T-helper cells:	all

Results

**P value:**	0.028
**FDR:**	over 50%

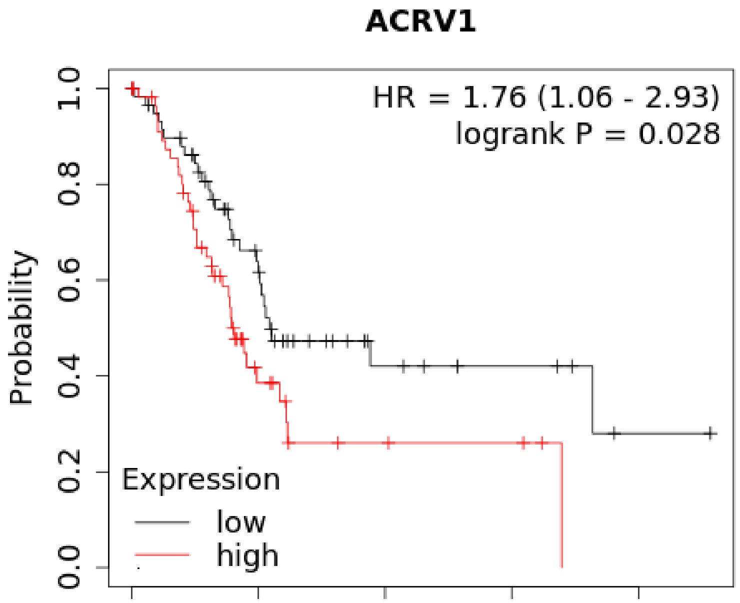

Click here to download the plot in TIFF format

Download plot as a PDF

Download p values vs. cutoff table

Median survival

Low expression cohort (months)	High expression cohort (months)
21.73	16.17

**RNAseq ID:**	PATE1 -
**Survival:**	OS
**Auto select best cutoff:**	checked
**Follow up threshold:**	all
**Censore at threshold:**	checked
**Compute median over entire database:**	false
**Cutoff value used in analysis:**	0
**Expression range of the probe:**	0 - 1
**Invert HR values below 1:**	not checked

Restrictions

Tumor type: Pancreatic ductal adenocarcinoma

Restrict analysis to subtypes...

Stage:	all
Gender:	all
Race:	all
Grade:	all
Mutation burden:	all

Restrict analysis based on cellular content...

Basophils:	all

B-cells:	decreased
CD4+ memory T-cells:	all
CD8+ T-cells:	all
Eosinophils:	all
Macrophages:	all
Mesenchymal stem cells:	all
Natural killer T-cells:	all
Regulatory T-cells:	all
Type 1 T-helper cells:	all
Type 2 T-helper cells:	all

Results

P value: 0.0039
FDR: 50%

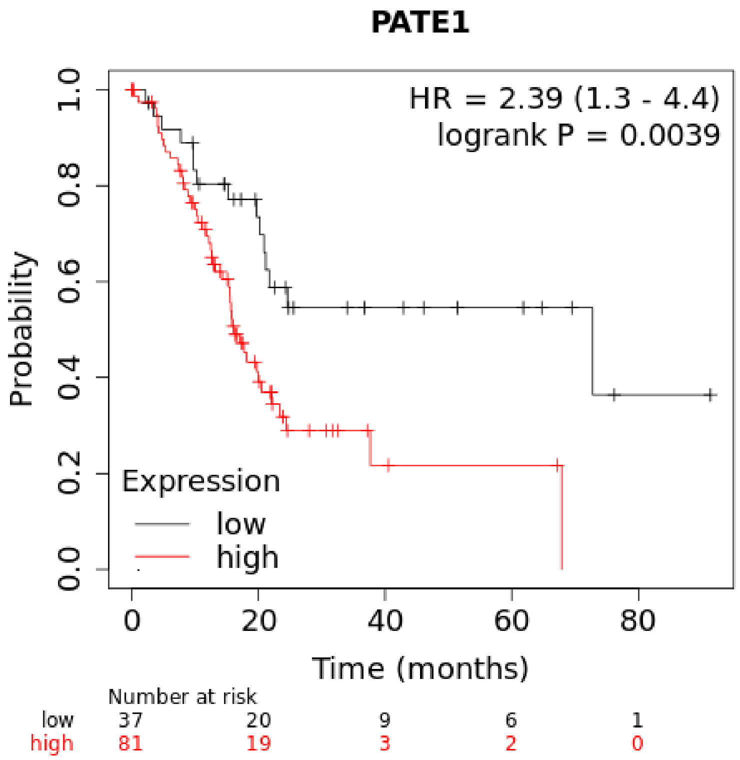

Click here to download the plot in TIFF format

Download plot as a PDF

Download p values vs. cutoff table

Median survival

Low expression cohort (months)	High expression cohort (months)
72.73	16.17

**RNAseq ID:**	PATE2 -
**Survival:**	OS
**Auto select best cutoff:**	checked
**Follow up threshold:**	all
**Censore at threshold:**	checked
**Compute median over entire database:**	false
**Cutoff value used in analysis:**	1
**Expression range of the probe:**	0 - 7
**Invert HR values below 1:**	not checked

Restrictions

Tumor type: Pancreatic ductal adenocarcinoma

Restrict analysis to subtypes...

Stage:	all
Gender:	all
Race:	all
Grade:	all
Mutation burden:	all

Restrict analysis based on cellular content...

Basophils:	all
B-cells:	decreased
CD4+ memory T-cells:	all
CD8+ T-cells:	all
Eosinophils:	all
Macrophages:	all
Mesenchymal stem cells:	all
Natural killer T-cells:	all
Regulatory T-cells:	all
Type 1 T-helper cells:	all
Type 2 T-helper cells:	all

Results

P value: 0.0014
FDR: 50%

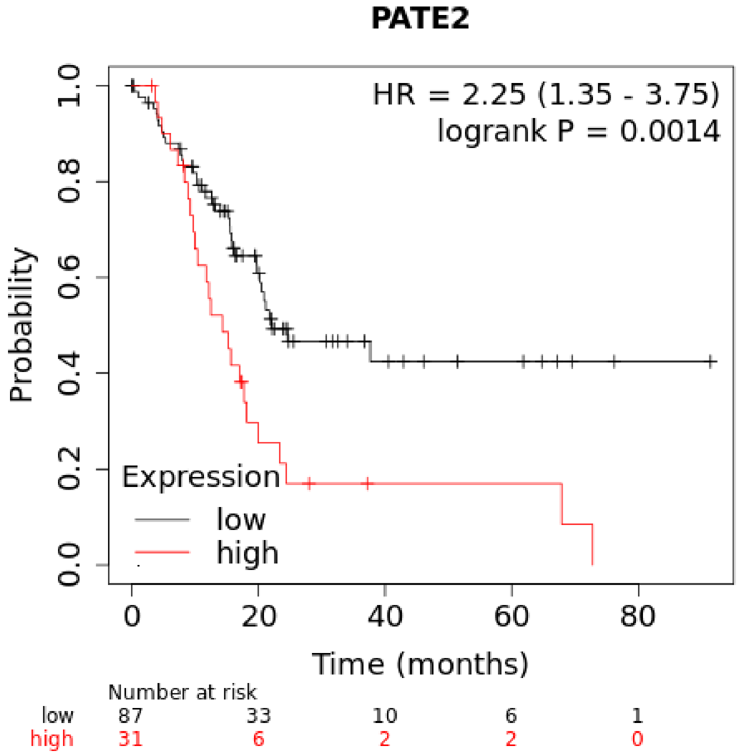

Click here to download the plot in TIFF format

Download plot as a PDF

Download p values vs. cutoff table

Median survival

Low expression cohort (months)	High expression cohort (months)
22.03	14.33

**RNAseq ID:**	PATE3	-
**Survival:**	OS	
**Auto select best cutoff:**	checked	
**Follow up threshold:**	all	
**Censore at threshold:**	checked	
**Compute median over entire database:**	false	
**Cutoff value used in analysis:**	0	
**Expression range of the probe:**	0 - 1	
**Invert HR values below 1:**	not checked	

Restrictions

Tumor type: Pancreatic ductal adenocarcinoma

Restrict analysis to subtypes...

Stage:	all
Gender:	all
Race:	all
Grade:	all
Mutation burden:	all

Restrict analysis based on cellular content...

Basophils:	all
B-cells:	decreased
CD4+ memory T-cells:	all
CD8+ T-cells:	all
Eosinophils:	all
Macrophages:	all
Mesenchymal stem cells:	all
Natural killer T-cells:	all
Regulatory T-cells:	all
Type 1 T-helper cells:	all
Type 2 T-helper cells:	all

Results

**P value:**	0.0261
**FDR:**	over 50%

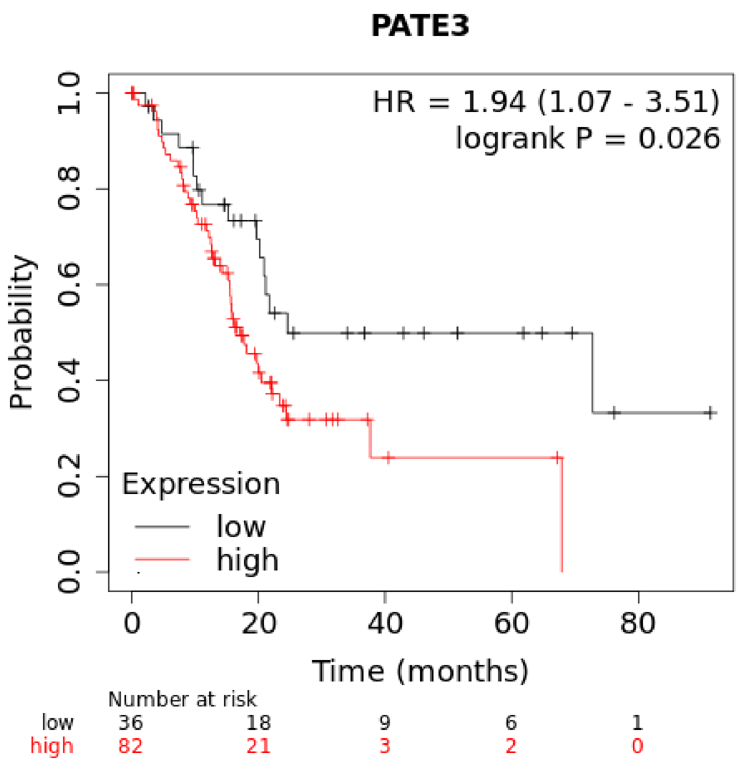

Click here to download the plot in TIFF format

Download plot as a PDF

Download p values vs. cutoff table

Median survival

Low expression cohort (months)	High expression cohort (months)
24.6	17.03

RNAseq ID: PATE4 -
Survival: OS
Auto select best cutoff: checked
Follow up threshold: all
Censore at threshold: checked
Compute median over entire database: false
Cutoff value used in analysis: 0
Expression range of the probe: 0 - 3
Invert HR values below 1: not checked

Restrictions

Tumor type: Pancreatic ductal adenocarcinoma

Restrict analysis to subtypes...

Stage: all
Gender: all
Race: all
Grade: all
Mutation burden: all

Restrict analysis based on cellular content...

Basophils: all

B-cells:	decreased
CD4+ memory T-cells:	all
CD8+ T-cells:	all
Eosinophils:	all
Macrophages:	all
Mesenchymal stem cells:	all
Natural killer T-cells:	all
Regulatory T-cells:	all
Type 1 T-helper cells:	all
Type 2 T-helper cells:	all

Results

P value: 0.1719
FDR: 100%

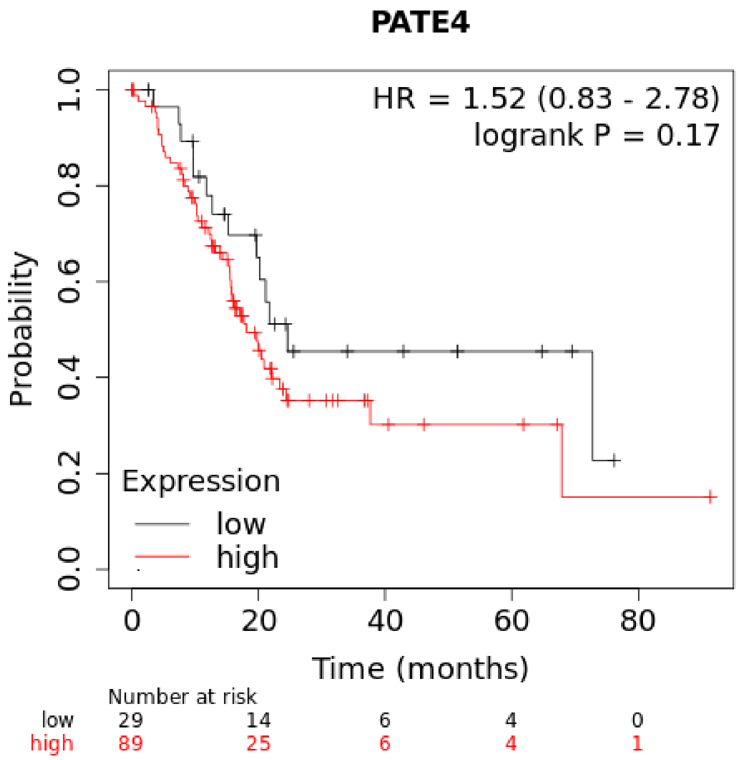

[Click here to download the plot in TIFF format]

[Download plot as a PDF]

[Download p values vs. cutoff table]

Median survival

Low expression cohort (months)	High expression cohort (months)
24.6	18.17

**RNAseq ID:**	CD59 -
**Survival:**	OS
**Auto select best cutoff:**	checked
**Follow up threshold:**	all
**Censore at threshold:**	checked
**Compute median over entire database:**	false
**Cutoff value used in analysis:**	17150
**Expression range of the probe:**	2610 - 39336
**Invert HR values below 1:**	not checked

Restrictions

Tumor type: Pancreatic ductal adenocarcinoma

Restrict analysis to subtypes...

Stage:	all
Gender:	all
Race:	all
Grade:	all
Mutation burden:	all

Restrict analysis based on cellular content...

Basophils:	all
B-cells:	decreased
CD4+ memory T-cells:	all
CD8+ T-cells:	all
Eosinophils:	all
Macrophages:	all
Mesenchymal stem cells:	all
Natural killer T-cells:	all
Regulatory T-cells:	all
Type 1 T-helper cells:	all
Type 2 T-helper cells:	all

Results

P value: 6.8e-6
FDR: 1%

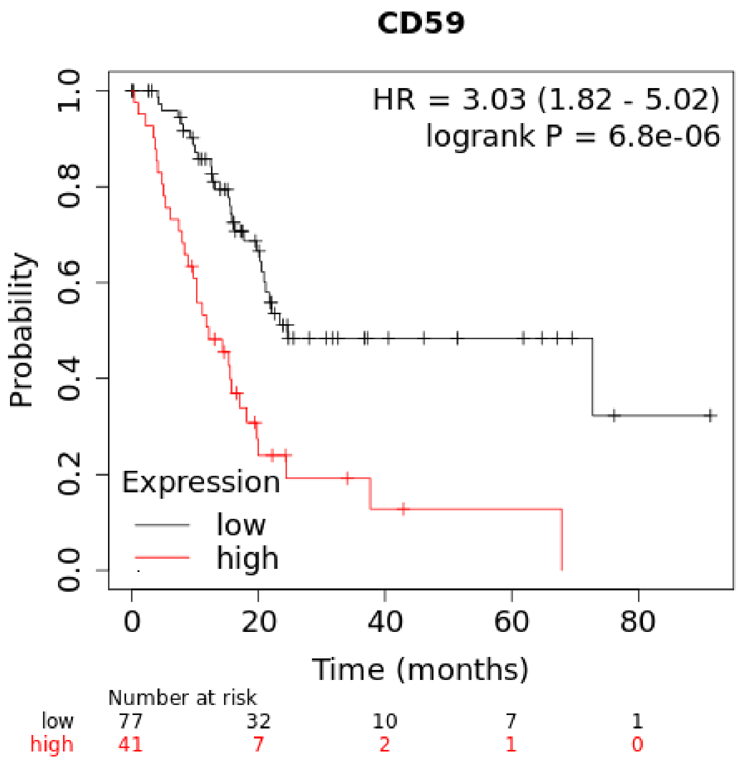

Click here to download the plot in TIFF format

Download plot as a PDF

Download p values vs. cutoff table

Median survival

Low expression cohort (months)	High expression cohort (months)
24.6	12.2

**RNAseq ID:**	LY6G6C	-
**Survival:**	OS	
**Auto select best cutoff:**	checked	
**Follow up threshold:**	all	
**Censore at threshold:**	checked	
**Compute median over entire database:**	false	
**Cutoff value used in analysis:**	18	
**Expression range of the probe:**	0 - 142	
**Invert HR values below 1:**	not checked	

Restrictions

Tumor type: Pancreatic ductal adenocarcinoma

Restrict analysis to subtypes...

Stage:	all
Gender:	all
Race:	all
Grade:	all
Mutation burden:	all

Restrict analysis based on cellular content...

Basophils:	all
B-cells:	decreased
CD4+ memory T-cells:	all
CD8+ T-cells:	all
Eosinophils:	all
Macrophages:	all
Mesenchymal stem cells:	all
Natural killer T-cells:	all
Regulatory T-cells:	all
Type 1 T-helper cells:	all
Type 2 T-helper cells:	all

Results

**P value:**	0.0078
**FDR:**	over 50%

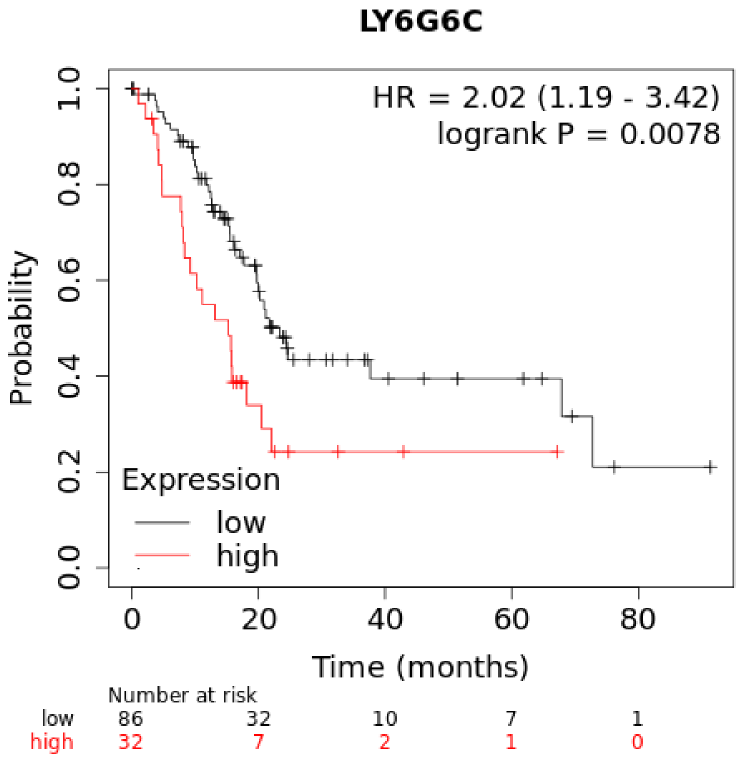

Click here to download the plot in TIFF format

Download plot as a PDF

Download p values vs. cutoff table

Median survival

Low expression cohort (months)	High expression cohort (months)
23.4	15.27

**RNAseq ID:**	LY6G6D -
**Survival:**	OS
**Auto select best cutoff:**	checked
**Follow up threshold:**	all
**Censore at threshold:**	checked
**Compute median over entire database:**	false
**Cutoff value used in analysis:**	0
**Expression range of the probe:**	0 - 1
**Invert HR values below 1:**	not checked

Restrictions

Tumor type: Pancreatic ductal adenocarcinoma

Restrict analysis to subtypes...

Stage:	all
Gender:	all
Race:	all
Grade:	all
Mutation burden:	all

Restrict analysis based on cellular content...

Basophils:	all

B-cells:	decreased
CD4+ memory T-cells:	all
CD8+ T-cells:	all
Eosinophils:	all
Macrophages:	all
Mesenchymal stem cells:	all
Natural killer T-cells:	all
Regulatory T-cells:	all
Type 1 T-helper cells:	all
Type 2 T-helper cells:	all

Results

P value: 0.0038
FDR: 50%

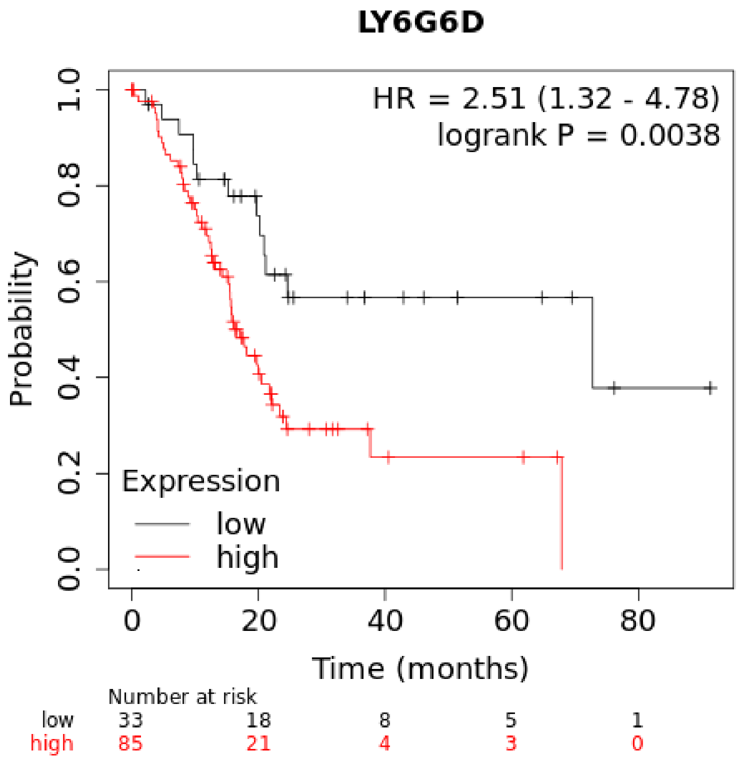

Click here to download the plot in TIFF format

Download plot as a PDF

Download p values vs. cutoff table

Median survival

Low expression cohort (months)	High expression cohort (months)
72.73	17.03

**RNAseq ID:**	LY6G6F -
**Survival:**	OS
**Auto select best cutoff:**	checked
**Follow up threshold:**	all
**Censore at threshold:**	checked
**Compute median over entire database:**	false
**Cutoff value used in analysis:**	0
**Expression range of the probe:**	0 - 2
**Invert HR values below 1:**	not checked

Restrictions

Tumor type: Pancreatic ductal adenocarcinoma

Restrict analysis to subtypes...

Stage:	all
Gender:	all
Race:	all
Grade:	all
Mutation burden:	all

Restrict analysis based on cellular content...

Basophils:	all
B-cells:	decreased
CD4+ memory T-cells:	all
CD8+ T-cells:	all
Eosinophils:	all
Macrophages:	all
Mesenchymal stem cells:	all
Natural killer T-cells:	all
Regulatory T-cells:	all
Type 1 T-helper cells:	all
Type 2 T-helper cells:	all

Results

P value: 0.0006
FDR: 10%

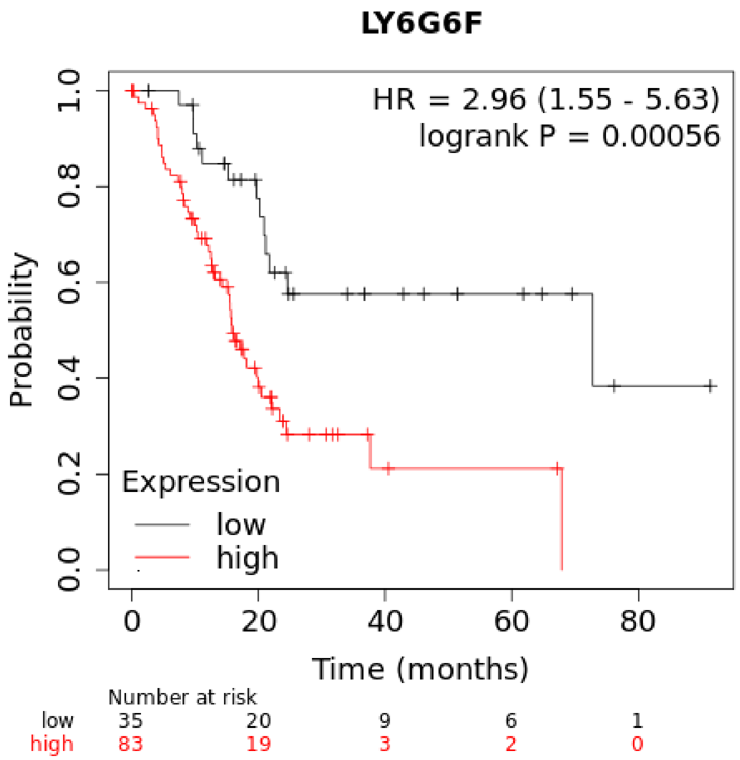

Click here to download the plot in TIFF format

Download plot as a PDF

Download p values vs. cutoff table

Median survival

Low expression cohort (months)	High expression cohort (months)
72.73	15.87

**RNAseq ID:**	LY6G5C -
**Survival:**	OS
**Auto select best cutoff:**	checked
**Follow up threshold:**	all
**Censore at threshold:**	checked
**Compute median over entire database:**	false
**Cutoff value used in analysis:**	49
**Expression range of the probe:**	15 - 586
**Invert HR values below 1:**	not checked

Restrictions

Tumor type: Pancreatic ductal adenocarcinoma

Restrict analysis to subtypes...

Stage:	all
Gender:	all
Race:	all
Grade:	all
Mutation burden:	all

Restrict analysis based on cellular content...

Basophils:	all
B-cells:	decreased
CD4+ memory T-cells:	all
CD8+ T-cells:	all
Eosinophils:	all
Macrophages:	all
Mesenchymal stem cells:	all
Natural killer T-cells:	all
Regulatory T-cells:	all
Type 1 T-helper cells:	all
Type 2 T-helper cells:	all

Results

**P value:**	0.0001
**FDR:**	5%

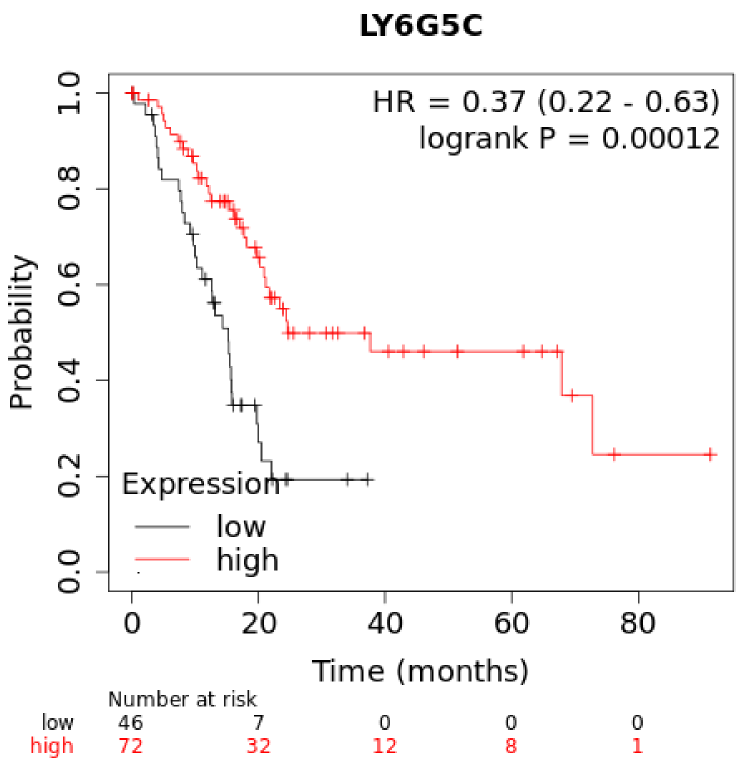

Click here to download the plot in TIFF format

Download plot as a PDF

Download p values vs. cutoff table

Median survival

Low expression cohort (months)	High expression cohort (months)
15.27	24.6

**RNAseq ID:**	LY6G5B -
**Survival:**	OS
**Auto select best cutoff:**	checked
**Follow up threshold:**	all
**Censore at threshold:**	checked
**Compute median over entire database:**	false
**Cutoff value used in analysis:**	41
**Expression range of the probe:**	3 - 192
**Invert HR values below 1:**	not checked

Restrictions

Tumor type: Pancreatic ductal adenocarcinoma

Restrict analysis to subtypes...

Stage:	all
Gender:	all
Race:	all
Grade:	all
Mutation burden:	all

Restrict analysis based on cellular content...

Basophils:	all

B-cells:	decreased
CD4+ memory T-cells:	all
CD8+ T-cells:	all
Eosinophils:	all
Macrophages:	all
Mesenchymal stem cells:	all
Natural killer T-cells:	all
Regulatory T-cells:	all
Type 1 T-helper cells:	all
Type 2 T-helper cells:	all

Results

P value: 0.0017
FDR: 10%

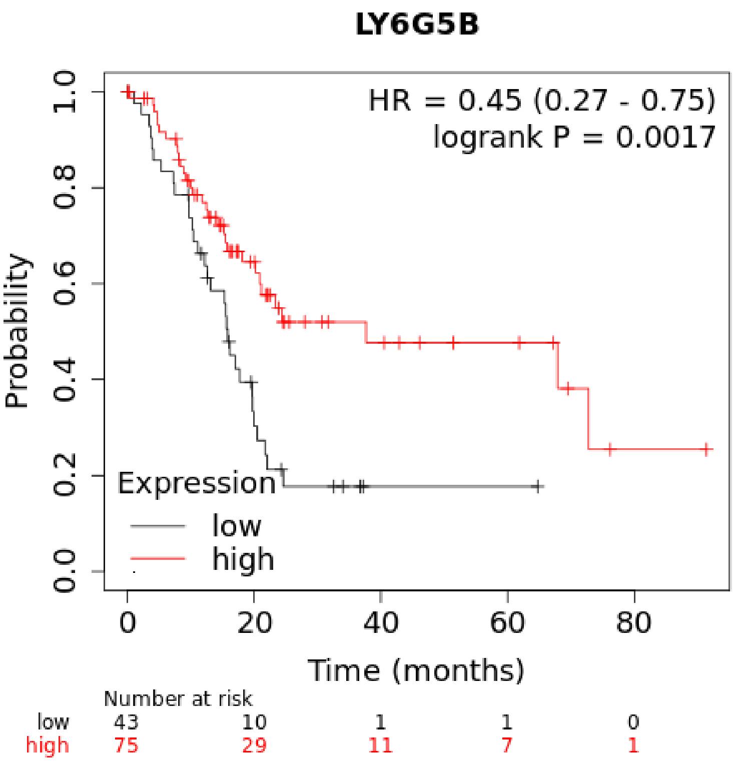

Click here to download the plot in TIFF format

Download plot as a PDF

Download p values vs. cutoff table

Median survival

Low expression cohort (months)	High expression cohort (months)
15.87	37.67

You can save the plots by right-clicking the image and then selecting "Save image as...". To generate a high resolution TIFF image, please adjust the "Settings" in the analysis page.

KMplot.com Copyright © 2009-2020 | Contact | Sitemap
